# Revisiting the taxonomical classification of Porcine Circovirus type 2 (PCV2): still a real challenge

**DOI:** 10.1186/s12985-015-0361-x

**Published:** 2015-08-28

**Authors:** Giovanni Franzo, Martí Cortey, Alex Olvera, Dinko Novosel, Alessandra Marnie Martins Gomes De Castro, Philippe Biagini, Joaquim Segalés, Michele Drigo

**Affiliations:** Department of Animal Medicine, Production and Health (MAPS), University of Padua, Viale dell’Università 16, Legnaro, PD 35020 Italy; The Pirbright Institute, Pirbright, Woking UK; IrsiCaixa - HIVACAT, Badalona, Spain; Department of Pathology, Croatian Veterinary Institute, Zagreb, Croatia; Department of Preventive Veterinary Medicine and Animal Health, College of Veterinary Medicine, University of Sao Paulo, Brazil/College of Veterinary Medicine, United Metropolitan College complex (FMU), Sao Paulo, Brazil; Unité de Virologie Moléculaire, Emergence et Co-évolution Virale UMR CNRS, Marseille, 7268 France; UAB, Centre de Recerca en Sanitat Animal (CReSA, IRTA-UAB), Campus de la Universitat Autònoma de Barcelona, Barcelona, Bellaterra 08193 Spain; Departament de Sanitat i Anatomia Animals, Universitat Autònoma de Barcelona (UAB), Barcelona, Bellaterra 08193 Spain

## Abstract

**Background:**

PCV2 has emerged as one of the most devastating viral infections of swine farming, causing a relevant economic impact due to direct losses and control strategies expenses. Epidemiological and experimental studies have evidenced that genetic diversity is potentially affecting the virulence of PVC2. The growing number of PCV2 complete genomes and partial sequences available at GenBank questioned the accepted PCV2 classification.

**Methods:**

Nine hundred seventy five PCV2 complete genomes and 1,270 ORF2 sequences available from GenBank were subjected to recombination, PASC and phylogenetic analyses and results were used for comparison with previous classification scheme.

**Results:**

The outcome of these analyses favors the recognition of four genotypes on the basis of ORF2 sequences, namely PCV2a, PCV2b, PCV2c and PCV2d-mPCV2b. To deal with the difficulty of founding an unambiguous classification and accounting the impossibility to define a p-distance cut-off, a set of reference sequences that could be used in further phylogenetic studies for PCV2 genotyping was established. Being aware that extensive phylogenetic analyses are time-consuming and often impracticable during routine diagnostic activity, ORF2 nucleotide positions adequately conserved in the reference sequences were identified and reported to allow a quick genotype differentiation.

**Conclusions:**

Globally, the present work provides an updated scenario of PCV2 genotypes distribution and, based on the limits of the previous classification criteria, proposes new rapid and effective schemes for differentiating the four defined PCV2 genotypes.

**Electronic supplementary material:**

The online version of this article (doi:10.1186/s12985-015-0361-x) contains supplementary material, which is available to authorized users.

## Background

Members of the family *Circoviridae*, genus *Circovirus*, are icosahedral, non-enveloped viruses with single-stranded ambisense circular genomes. Two members of this genus have been reported to infect mammals; *Porcine circovirus type 1* (PCV1) and *Porcine circovirus type 2* (PCV2) [1]. PCV1, initially designated as porcine circovirus (PCV), was first discovered in 1974 as a permanent contaminant of continuous cell culture PK15 and is considered non-pathogenic [2]. At the beginning of 1990s a new syndrome, named postweaning multisistemic wasting syndrome (PMWS) and nowadays designated as PCV2-systemic disease (PCV2-SD) [3], was described by Clark and Harding [4, 5] and the etiological agent, recognized as a *Circovirus* different from PCV1, was first isolated in 1998 [6]. Since the first reports, PCV2 infection was reported all over the world and PMWS showed epidemical proportion in Europe and South East Asia by the late 1990’s and in the Americas by 2004–05 [7]. Progressively, several clinical manifestations, collectively named porcine circovirus diseases (PCVD), have been associated with PCV2 infection and are responsible of a relevant economic impact to the pig industry due to direct losses and control measures’ costs. PCV2 display a simple ambisense genome ranging from 1766 to 1768 nucleotides. Three major Open Reading Frames (ORF1, ORF2 and ORF3) have been recognized [8], encoding for proteins involved respectively in viral genome replication (Rep and Rep’), capsid protein (Cap) and possibly in pathogenesis (ORF3) [9–11].

At the intraspecific level, two major PCV2 groups were initially defined (i.e. PCV2a and PCV2b) [12, 13]. Applying PASC analyses [14, 15] and linearized phylogenetic trees [16] under the assumption of strict molecular clock, two nucleotide diversity cut-offs for ORF2 (3.5 %) and the complete genome (2.0 %) were proposed [17]. Accordingly, PCV2 genotypes a and b were designated under the European project no 513928 of the Sixth Framework Programme (www.pcvd.eu) [18]. A third genotype named PCV2c was reported from Denmark in the 1980s [19] and has recently been identified in the Brazilian Pantanal [20]. Two additional genotypes were proposed after analyzing several sequences from the People’s Republic of China [21]; although subsequent analyses did not support the genotype status for those strains [22]. Another study, restricted to Chinese sequences, also proposed a fourth group, which fitted with the existing definition of genotype and was named PCV2d [8]. This late clade, apparently more virulent [23], was also detected in the USA (designated as mPCV2b) and in other countries [24, 25]. The mPCV2b-PCV2d genotype will be referred in this paper as PCV2d. A genotype shift from the older PCV2a to the new variant PCV2b has been reported during a time window that coincided with an increase in severe outbreaks of PCVD worldwide: Canada [26, 27], China [21], Denmark [19], Spain [28], Sweden [29], Switzerland [30] and USA [31]. Interestingly, the oldest PCV2b strains were reported at the beginning of 90’s [32], contemporaneously to the first report of PCV2-SD. All these elements suggest that the viral genotype plays a major role in the appearance of clinical disease [7].

A growing number of PCV2 complete genomes and partial sequences are available at GenBank (more than 3,300 in July 2014), most of them updated after 2008 when the PCV2 genotype proposal was published. Since then, several new genotypes have been proposed, but also highly divergent sequences have been reported elsewhere (i.e. [33] .) Therefore, the aim of this paper was to revisit the intraspecific taxonomy of PCV2 and the genotype definition to check its current validity, to unify nomenclature and to avoid further misconceptions.

## Results

### Dataset

After removal of poor quality sequences and exclusion of predicted recombinant sequences detected by RDP3, 595 complete genomes and 954 ORF2 were maintained (Additional file 1). Recombination traces were reported in a substantial proportion of the PCV2 whole genome (37.7 %) and ORF2 (24.3 %) sequences downloaded from GenBank. Recombination was pervasive and affected several genome fragments, intra- and inter-genes, involving strains belonging to both closely and distantly related clades (i.e. different genotypes according to previous classification). The majority of the positions in the alignments was variable in the complete PCV2 genomes (52.8 %) and especially in the ORF2 (76.4 % nucleotide, 87.2 % amino acid).

### Phylogenetic analysis and genotype definition

**Fig. 1:**
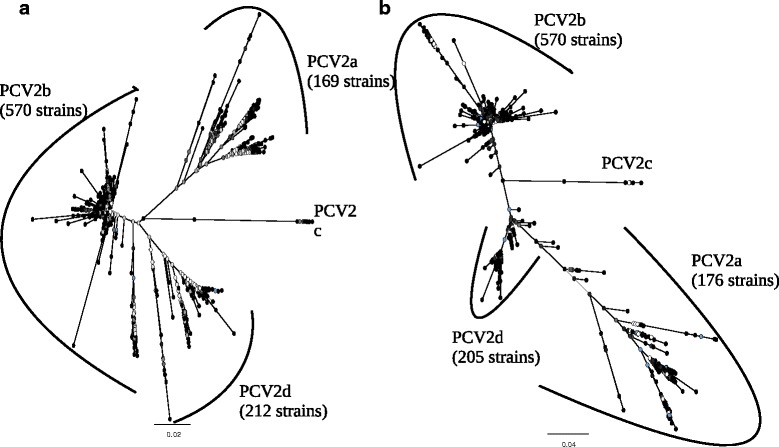
Phylogenetic trees reconstructed using Neighbor Joining (**a**) and Maximum likelihood (**b**) methods based on the ORF2 database after removing the recombinant strains detected by RDP. Bootstrap support has been reported using gray scale ranging from white (i.e. bootstrap support = 0) to black (i.e. bootstrap support = 1)

Phylogenetic trees reconstructed from PCV2 ORF2 using NJ (Fig. 1a) and ML (Fig. 1b) methods displayed very similar topologies and four main clades were identified. These four clades substantially corresponded to the previously defined PCV2a, PCV2b, PCV2c and PCV2d genotypes. Very few strains (n = 7) showed contradictory clustering between PCV2a and PCV2d clades in the ORF2 trees (Additional file 1). The same clustering in four major groups was obtained rooting the tree using PCV1 sequences as outgroup (data not shown). Similarly, phylogenetic trees reconstructed using complete genome showed a similar topology even if with a closer relationship between remaining PCV2d and PCV2a strains (named according to ORF2 classification).

### PASC analysis

**Fig. 2:**
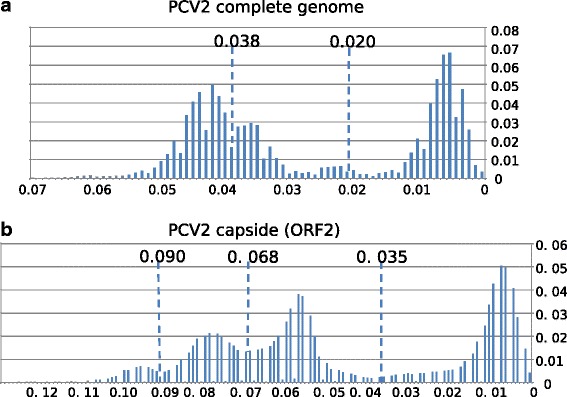
PASC analysis of complete PCV2 genome database (**a**) and of ORF2 sequences (**b**). The percentage of pairwise p-distances comprised within a 0.01 p-distance interval is reported

PASC analyses based on the PCV2 complete genome and the ORF2 (Fig. 2) displayed a multimodal curve. In both cases the definition of a single cut-off value to define PCV2 genotypes appeared complicated. Applying the previously reported cut-off values to classify complete genomes (0.02) and ORF2 (0.035) to the PASC analyses, 27 and 18 genotypes should be defined in PCV2 considering whole genomes and ORF2, respectively. For complete genomes (Fig. 2a), a threshold between 0.034 and 0.042 would separate PCV2a and PCV2c from PCV2b/PCV2d, but a second threshold of 0.038 would be necessary to differentiate PCV2b and PCV2d. The picture is even more complicated for ORF2 (Fig. 2b). The first value pointed by the PASC analysis (around 0.068) would be meaningless for the genotype definition and a second value around 0.090 would only differentiate PCV2c from PCV2a/PCV2b/PCV2d. Alternatively, a theoretical cut-off around 0.078 would separate PCV2a, PCV2c and PCV2b/PCV2d, but this value was not robust according to the pairwise distribution. Consequently, a clear overlap between the p-distances calculated using sequences belonging to the same genotype and the sequences belonging to different genotypes was reported (Additional file 2). Even the sequences corresponding to the highly divergent PCV2c genotype displayed a p-distance with those belonging to the PCV2a and PCV2b comprised between 0.087 and 0.165, within the range of intra-genotype distances of the PCV2a (0–0.103) and PCV2b (0–0.12).

### Rate of substitution estimation

**Fig. 3:**
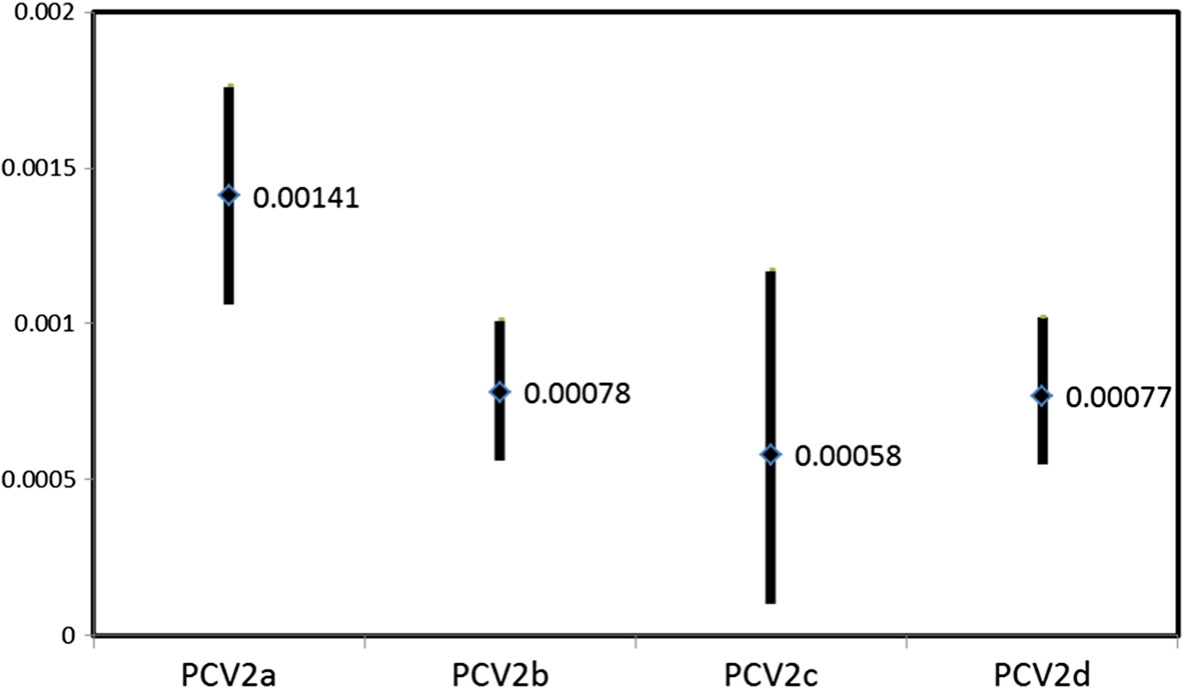
Substitution rates and 95 % CI for the PCV2 genotypes obtained using BEAST

The substitution rates and the 95 % CI for the previously defined PCV2a, PCV2b, PCV2c and PCV2d genotypes based on the ORF2 are shown in Fig. 3. The estimated rate for PCV2a (1.41°10 ^−3^ subs · site ^−1^ · year ^−1^ ) is higher compared with PCV2b (7.8°10 ^−4^ ) and PCV2d (7.7°10 ^−4^ ). The same picture is reported when the whole PCV2 genome is used, with PCV2a sowing higher substitution rates than the others. Actually, when all genotypes were analyzed together, the marginal likelihood estimation indicated that a relaxed molecular clock fit much better in the model than a strict one (data not shown), pointing that the variation among genotypes and among lineages is affecting the goodness of the model.

### Reference dataset

In view of the confused results reported in the PASC analysis, an alternative methodology for genotyping PCV2 based on marker positions is proposed. Table 1 summarizes the ORF2 marker positions that are consistently (>95 %) different among PCV2 genotypes. Taking as a reference genotype PCV2b, 9 unique positions were identified to define genotype PCV2a, thirty-five for PCV2c and five for PCV2d (Table 1). In addition, a more robust ORF2 reference dataset was built selecting only those sequences unequivocally part of one genotype on the basis of the topology of both ML and NJ trees (Additional file 3).

**Table 1:** Marker nucleotide positions (95 % CI) in the ORF2 among PCV2 proposed genotypes are shown in boldface. The marker positions are not reported for PCV2b since this genotype was used as the reference genotype. The number of reference strains for each genotype and the number of sequences not fitting these marker positions are reported in the header and between brackets, respectively

Genotype/Position	PCV2a (169)	PCV2b (532)	PCV2c (3)	PCV2d (201)
129	C (4)	C (3)	**A (0)**	C (0)
138	C (2)	C (2)	**T (0)**	C (0)
154	A (0)	A (0)	**T (0)**	A (1)
157	T (1)	T (4)	T (0)	**A (3)**
159	C (0)	C (2)	**T (0)**	C (5)
161	G (0)	G (0)	**T (0)**	G (0)
162	A (4)	A (6)	**T (0)**	**T (5)**
168	T (1)	T (1)	**C (0)**	T (0)
178	A (0)	A (1)	**T (0)**	A (0)
180	C (1)	C (2)	**A (0)**	C (2)
181	A (0)	A (0)	**C (0)**	A (0)
182	C (0)	C (0)	**A (0)**	C (0)
187	A (3)	A (1)	**T (0)**	A (0)
190	A (2)	A (0)	**C (0)**	A (0)
198	C (2)	C (8)	**T (0)**	C (0)
232	G (2)	G (0)	**C (0)**	G (0)
255	**G (1)**	C (5)	C (0)	C (2)
256	**A (2)**	T (3)	T (0)	T (0)
258	**C (2)**	A (4)	A (0)	A (0)
262	**A (1)**	C (3)	C (0)	C (0)
263	**A (0)**	C (1)	C (0)	C (0)
264	**A (0)**	C (1)	C (0)	C (0)
265	**A (2)**	C (1)	C (0)	C (0)
271	**A (5)**	G (0)	G (0)	G (0)
273	**A (1)**	G (9)	G (0)	G (10)
306	G (0)	G (1)	**A (0)**	G (2)
309	T (2)	T (2)	**G (0)**	T (0)
317	G (0)	G (0)	**T (0)**	G (0)
318	G (0)	G (2)	**T (0)**	G (0)
319	C (0)	C(0)	**G (0)**	C (0)
322	T (0)	T (0)	**A (0)**	T (0)
324	C (4)	C (4)	**A (0)**	C (0)
339	G (1)	G (7)	**A (0)**	G (3)
513	T (0)	T (4)	T (0)	**C (0)**
585	C (1)	C (1)	**T (0)**	**T (2)**
597	C (2)	C (10)	**A (0)**	C (0)
598	A (2)	A (4)	**C (0)**	A (0)
599	C (8)	C (4)	**A (0)**	C (0)
600	T (3)	T (1)	**C (0)**	T (0)
606	C (1)	C (3)	**T (0)**	C (0)
607	G (0)	G (1)	**C (0)**	G (0)
619	T (0)	T (1)	**A (0)**	T (0)
621	C (0)	C (2)	**T (0)**	C (0)
623	A (0)	A (2)	**C (0)**	A (0)
629	A (0)	A (2)	**C (0)**	A (0)
637	A (1)	A (2)	**G (0)**	A (0)
643	G (2)	G (2)	G (0)	**A (4)**

## Discussion

A unified criterion for PCV2 genotyping is paramount to allow the comparison of molecular epidemiology data worldwide [22]. Historically, the intraspecific classification of PCV2 has been controversial [22, 23, 34, 35]. In 2008, the EU consortium on Porcine Circovirus Diseases proposed a standardized nomenclature for PCV2 genotype definition based on pairwise sequence comparisons [18]. The PASC analyses applied to PCV2 complete and capsid (ORF2) nucleotide sequences defined two distance thresholds at 0.020 and 0.035, respectively [17]. Since then, a huge number of PCV2 sequences has been deposited in the GenBank, and several new genotypes were proposed (i.e. 7) though they were not always validated [21, 22, 35]. A significant proportion of those sequences have a recombinant origin and in some cases they have been circulating with increasing prevalence in several Asian countries and USA [36–38]. Bearing this in mind, the intraspecific taxonomy of PCV2 and the genotype definition have been revised to check their current validity in order to unify the nomenclature and avoid further misconceptions.

Based on the obtained results , one of the main PASC and linearized phylogenetic trees assumptions is unequivocally violated. These techniques assume equal rates of evolution among clades and, according to the BEAST estimations for the four main groups of the phylogenetic trees (Fig. 1), they are significantly different (Fig. 3). These evidences, coupled with the enormous amount of new sequences available, have important implications for PCV2 intraspecific classification. Mostly, the thresholds applied since 2008 to define PCV2 genotypes using PASC and linearized phylogenetic trees are currently not applicable to all PCV2 strains and therefore these methods should be changed. Since the accepted scheme is no longer valid, an alternative method to genotype PCV2 strains in an unambiguous way is proposed. The suggested approach tries to account for different theoretical and practical issues. The first challenge when dealing with PCV2 classification is related to the presence of several recombinant strains that, as a matter of fact, belongs to more than one genotype. Considering the high recombination frequency reported in this study and by other authors [38–41] and given the tendency to display a higher frequency of breakpoints between or at the periphery of the genes [42], complete genome sequences pose indubitably a greater challenge for PCV2 classification. In fact, despite the attempt to exclude all recombinants sequences from the generated databases using a combination of different approaches with RDP software, current methods are still perfectible and their results are somewhat dependent on database features, initial settings and subjective refinement of the results. Additionally, other evolutionary phenomena may lead to the identification of putative recombination, including lineage-specific rate variation, convergent evolution and natural selection. So, it cannot be excluded that some recombinant sequences were not identified or misclassified, affecting the results. In practice it is infeasible to perform an extensive recombination analysis on routinely basis and results would probably be quite different among different analysis and operators. Moreover, the higher percentage of identity of ORF1 gene provides lower phylogenetic signal, limiting its applicability to phylogenetic inference and recombination detection. Finally, complete genome sequencing is laborious and expensive, so many laboratories currently base their analysis on ORF2 sequences. Taking into account all these factors, a classification approach based on the ORF2 gene was preferred. Aiming at offering an unambiguous classification scheme, several reference sequences, whose classification was clear and concordant for the two phylogenetic reconstruction methods, were selected (Additional files 1 and 3). This allows to define four genotypes using both phylogenetic reconstruction and genotype-specific marker positions. The substantial agreement between the more rapid NJ method and the more accurate ML tree reconstruction method represent a remarkable advantage. In addition to substantiate the robustness of this classification, it suggests the possibility to use a quicker approach still obtaining reliable results. Being aware that extensive phylogenetic analysis are time consuming and often impracticable during routine diagnostic activity, conserved nucleotide marker positions in the ORF2 are proposed to perform a quick genotype differentiation. These markers, consistently (>95 %) present in each of the four accepted genotypes ORF2, are depicted in Table 1 and can be used as a reference to assign a certain sequence to one of the newly proposed genotypes. Noteworthy, classification offered in the present study substantially agrees with that proposed by Olvera et al. [13], with the remarkable difference that strains previously classified into PCV2b Clade 1C are now part of the PCV2d genotype.

## Conclusion

The present study confirms and validates the variability of viral sequences and the high intra- and inter-genotype recombination frequency between PCV2 strains, and highlights the difficulty to pinpoint an unequivocal genotype definition of this virus. Considering that the method based on genetic distance seems to be no longer valid and has generated some misclassification through time, it is suggested to use an approach based on the reference sequences and/or identification of marker positions.

## Materials and methods

### Dataset

Nine hundred seventy five PCV2 complete genomes and 1,270 ORF2 sequences available from GenBank (www.ncbi.nlm.nih.gov) were downloaded in January 2014. Complete genome sequences were aligned at nucleotide level. Considering the coding nature of ORF2 sequences, multiple sequence alignment was carried out at amino acid level and was then used to generate the corresponding nucleotide sequences. The MUSCLE algorithm [43] implemented in MEGA6 [44] was employed in both cases. Alignments were visually inspected and edited to remove poorly aligned sequences or those with modification in the reading frame or premature stop codon, highly suggestive of sequencing errors. The alignments robustness was evaluated using Guidance [45] assuming MAFFT [46] as multiple alignment algorithms.

### Recombination analyses

Recombination analysis was performed on both complete genome and ORF2 alignments using RDP3 [47]. The RDP, GENECONV, MaxChi and 3Seq methods were selected as primary scan, while all the methods implemented in RDP3 were used for recombination detection refinement. Settings for each method were adjusted considering the database features following the recommendations of the RDP3 manual. A recombination event was accepted if detected by more than two methods with a significance p-value of p < 0.01 with Bonferroni’s correction. All sequences identified as recombinant where excluded from further analysis.

### Phylogenetic analyses

Phylogenetic trees for both complete genome and ORF2 were reconstructed using the maximum likelihood (ML) method implemented in PhyML [48]. Substitution model was selected according to Bayesian Information Criterion (BIC), calculated using Jmodeltest 2.1.2 [49]. A combination of Nearest Neighbor Interchange (NNI) and Sub-tree Pruning and Regrafting (SPR) was used as tree rearrangement strategy. The phylogenetic tree reliability was evaluated using the Shimodaira–Hasegawa [SH]-aLRT [50] likelihood-based measures of branch supports implemented in PhyML. Phylogenetic trees were also reconstructed with the Neighbor Joining (NJ) method and MEGA6 using the substitution model with the better BIC score given by the same MEGA software. The confidence of the internal branches was evaluated performing 1000 bootstrap pseudo-replicates of the aligned dataset.

### PASC analyses

Pairwise p-distances among sequences for every dataset were calculated with MEGA6. Distances were ordered and a histogram of pairwised differences was constructed to perform a PASC analysis using Microsoft Excel 2010.

### Rates of substitution

Estimations for the rate of substitution were calculated for the nucleotide sequences of the PCV2 genomes and the ORF2 using a Bayesian Markov chain Monte Carlo (MCMC) approach implemented in BEAST v.1.8.0 package [51]. Three independent runs of MCMC per dataset were performed under a strict and a relaxed molecular clock model, using the General Time Reversible model of sequence evolution and the remaining default parameters in the prior’s panel. To account for different population dynamics through time a Bayesian Skygrid [52] was chosen as tree prior. The MCMC run was 5 × 10 ^7^ steps long and the posterior probability distribution of the chains was sampled every 1000 steps. Convergence was assessed by visually inspecting the runs’ trace plot and on the basis of an effective sampling size greater than 200 after a 10  % burn-in using Tracer software, version 1.6 [53]. The estimations are the mean values obtained for the three runs, combined using LogCombiner v1.8.0 (part of the BEAST 1.8. package). The mean substitution rate and the 95  % CI were calculated, and the best-fitting models were selected by a Bayes factor using marginal likelihoods estimator tools implemented in Tracer and the Stepping Stone and Path Sampling approaches [54] .

## Additional files

Additional file 1:
**Spreadsheet containing the datasets used in the present study.** For ORF2, the clustering according to NJ and ML phylogenetic trees is included. The list of Acc.Num of reference strains is also reported. (XLS 313 kb) Additional file 2:
**Intra and inter genotype pairwise p-distances obtained from complete ORF2 Database (a) and on reference sequences (b).** (PDF 2920 kb) Additional file 3.
**ORF2 reference dataset built selecting only those sequences unequivocally part of one genotype on the basis of the topology of both ML and NJ trees.** (PDF 921 kb)
