# Supplementary material for: Revisiting the taxonomical classification of Porcine Circovirus type 2 (PCV2): still a real challenge
Source: Virol J. 2015 Aug 28;12:131. doi: 10.1186/s12985-015-0361-x (PMC4551364; doi:10.1186/s12985-015-0361-x)
Supplement: Additional file 2: — Intra and inter genotype pairwise p-distances obtained from complete ORF2 Database (a) and on reference sequences (b). (PDF 2920 kb) [file 12985_2015_361_MOESM2_ESM.pdf]

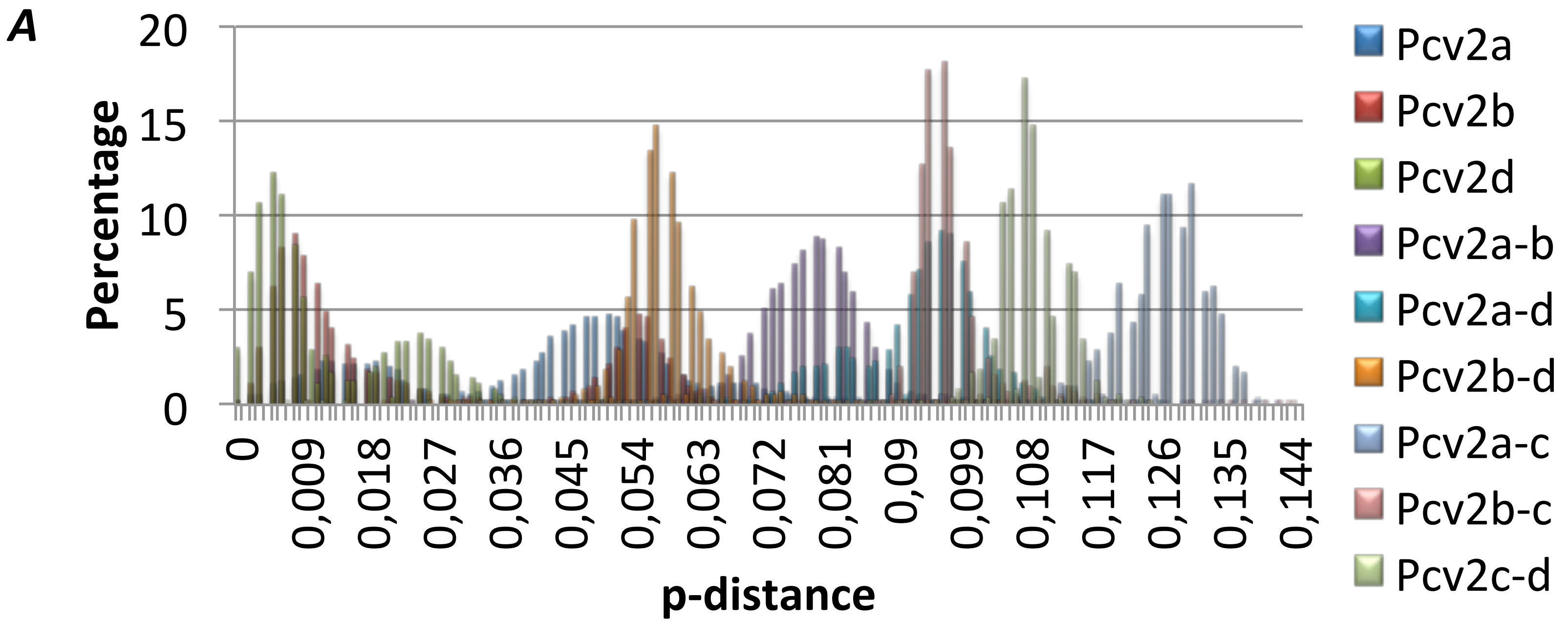

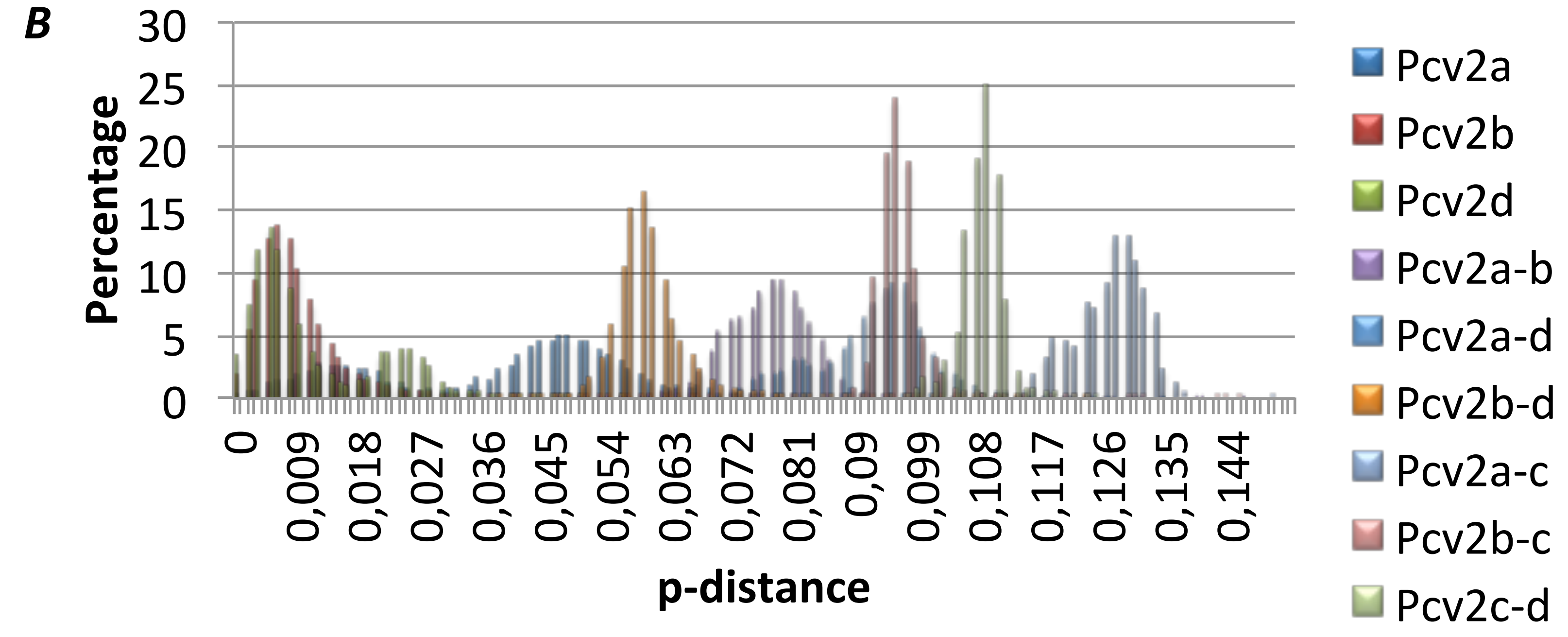

Appendix III. Intra and inter genotype pairwise p-distances obtained from complete ORF2 database (a) and on reference sequences (b).
