# Supplementary material for: Revisiting the taxonomical classification of Porcine Circovirus type 2 (PCV2): still a real challenge
Source: Virol J. 2015 Aug 28;12:131. doi: 10.1186/s12985-015-0361-x (PMC4551364; doi:10.1186/s12985-015-0361-x)
Supplement: Additional file 3. — ORF2 reference dataset built selecting only those sequences unequivocally part of one genotype on the basis of the topology of both ML and NJ trees. (PDF 921 kb) [file 12985_2015_361_MOESM3_ESM.pdf]

>EF184225_pcv2a

ATGACGTATCCAAGGAGGCGTTACCGGAGAAGAAGACACCGCCCCCGCAGCCATCTTGGCCATATCCTCCGCCGCCG
CCCCTGGCTCGTCCACCCCCGCCACCGTTACCGCTGGAGAAGGAAAAATGGCATTTTCAACAGCCGCCTCTCCCGCAC
CTTCGGATATACTGTCAAAGCTACCACAGTCACAACGCCCTCCTGGGCGGTGGACATGCTGAGATTTAATATTAACGA
CTTTCTTCCCCCGGGAGGGGGGACCAACAAAATCTCTATACCCTTTGAATACTACAGAATAAGAAAGGTTAAGGTTGA
ATTCTGGCCCTGCTCCCCAATCACCCAGGGTGACAGGGGAGTTGGATCCAGTGCTGTTATTCTAGATGATAACTTTGTA
ACTAAGGCCACAGCCCTAACCTATGACCCCTACGTAAACTACTCCTCCCGCCATACCATAACCCAGCCCTTCTCCTACCA
CTCCCGGTACTTTACCCCCAAACCTGTCCTTGATTCCACCATTGATTACTTCCAACCAAATAACAAAAGGAATCAGTTGT
GGATGAGACTTCAAACCACTGGAAATGTAGACCACGTAGGCCTCGGCACTGCGTTCGAAAACAGTAAATACGACCAG
GACTACAATATCCGTGTAACCCTGTATGTACAATTCAGAGAATTTAATCTTAAAGACCCCCCACTTAAACCC--------

>FJ660969_pcv2a

ATGACGTATCCAAGGAGGCGTTACCGGAGAAGAAGACACCGCCCCCGCAGCCATCTTGGCTCCATCCTCCGCCGCCG
CCCCTGGCTCGTCCACCCACGCCACCGCTACCGCTGGAGAAGGAAAAATGGAATCTTCAATGCCCGCCTCTCCCGCAC
CTTCGGATATACTGTCAAGGCTACCACAGTCAGAACGCCCTCCTGGGCGGTGGACATGATGAGATTTAATATTGACGA
CTTCCTTCCCCCGGGAGGGGGGACCAACAAAATCTCTATACCCTTTGAATACTACAGAATAAGAAAGGTTAAGGTTGA
ATTCTGGCCCTGCTCCCCAATCACCCAGGGTGACAGGGGAGTTGGATCCAGTGCTGTTATTCTAGATGATAACTTTTTC
CCTAAGTCCACAGCCCTAACCTATGACCCCTACGTAAACTACTCCTCCCGCCACACCATACCCCAGCCCTTCTCCTACCA
CTCCCGGTACTTTACCCCCAAACCTGTCCTTGATTCCACCATTGATTACTTCCAACCAAATAACAAAAGGAATCAGCTGT
GGATGAGAATTCAAACCAGTAAAAATGTAGACCACGTAGGCCTCGGCACTGCGTTCGAAAACAGTAAATACGACCAG
GACTACAATATCCGTGTAACCATGTATGTACAATTCAGAGAATTTAATCTTAAAGACCCCCCACTTAACCCTAAG-----

>EF184220_pcv2a

ATGACGTATCCAAGGAGGCGTTTCCGCAGACGAAGACACCGCCCCCGCAGCCATCTTGGCCAGATCCTCCGCCGCCGC
CCCTGGCTCGTCCACCCCCGCCACCGTTACCGCTGGAGAAGGAAAAATGGCATCTTCAACACCCGCCTCTCCCGCACCT
TCGGATATACTGTCAAGGCTACCACAGTCACAACGCCCTCCTGGGCGGTGGACATGATGAGATTTAATATTAACGACT
TTGTTCCCCCGGGAGGGGGGACCAACAAAATCTCTATACCCTTTGAATACTACAGAATAAGAAAGGTTAAGGTTGAAT
TCTGGCCCTGCTCCCCAATCACCCAGGGTGACAGGGGAGTGGGCTCCACTGCTGTTATTCTAGATGATAACTTTGTAA
CAAAGGCCACTGCCCTAACCTATGACCCCTATGTAAACTACTCCTCCCGCCATACAATCCCCCAACCCTTCTCCTACCAC
TCCCGTTACTTCACACCCAAACCTGTACTGGATAGAACTATTGATTACTTCCAGCCAAACAACAAAAAAAAATCAGCTTT
GGCTGAGGCTACAAACCTCTGCAAATGTAGACCACGTAGGCCTCGGCACTGCGTTCGAAAACAGTAAATACGACCAG
GACTACAATATCCGTGTAACCATGTATGTACAATTCAGAGAATTTAATCTTAAAGACCCCCCACTTAAACCC--------

>EU386606_pcv2a

ATGACGTATCCAAGGAGGCGTTTCCGCAGACGAAGACACCGCCCCCGCAGCCATCTTGGCCAGATCCTCCGCCGCCGC
CCCTGGCTCGTCCACCCCCGCCACCGTTACCGCTGGAGAAGGAAAAATGGCATCTTCAACACCCGCCTCTCCCGCACCT
TCGGATATACTGTCAAGGCTACCACAGTCACAACGCCCTCCTGGGCGGTGGACAGGATGAGATTTAATATTAACGACT
TTGTTCCCCCGGGAGGGGGGACCAACAAAATCTCTATACCCTTTGAATACTACAGAATAAGAAAGGTTAAGGTTGAAT
TCTGGCCCTGCTCCCCAATCACCCAGGGTGACAGGGGAGTGGGCTCCACTGCTGTTATTCTAGATGATAACTTTGTAA
CAAAGGCCACTGCCCTAACCTATGACCCCTATGTAAACTACTCCTCCCGCCATACAATCCCCCAACCCTTCTCCTACCAC
TCCCGTTACTTCACACCCAAACCTGTACTGGATAGAACTATTGATTACTTCCAGCCAAACAACAAAAAAAAATCAGCTTT
GGCTGAGGCTACAAACCTCTGCAAATGTAGACCACGTAGGCCTCGGCACTGCGTTCGAAAACAGTAAATACGACCAG
GACTACAATATCCGTGTAACCATGTATGTACAATTCAGAGAATTTAATCTTAAAGACCCCCCACTTAAACCC--------

>EF371551_pcv2a

ATGACGTATCCAAGGAGGCGTTTCCGCAGACGAAGACACCGCCCCCGCAGCCATCTTGGCCAGATCCTCCGCCGCCGC
CCCTGGCTCGTCCACCCCCGCCACCGTTACCGCTGGAGAAGGAAAAATGGCATCTTCAACACCCGCCTCTCCCGCACCT
TCGGATATACTGTCAAGGCTACCACAGTCACAACGCCCTCCTGGGCGGTGGACATGATGAGATTTAATATTAACGACT
TTGTTCCCCCGGGAGGGGGGACCAACAAAATCTCTATACCCTTTGAATACTACAGAATAAGAAAGGTTAAGGTTGAAT
TCTGGCCCTGCTCCCCAATCACCCAGGGTGACAGGGGAGTGGGCTCCACTGCTGTTATTCTAGATGATAACTTTGTAA
CAAAGGCCACTGCCCTAACCTATGACCCCTATGTAAACTACTCCTCCCGCCATACAATCCCCCAACCCTTCTCCTACCAC
TCCCGTTACTTCACACCCAAACCTGTACTGGATAGAACTATTGATTACTTCCAGCCAAACAACAAAAAAAATCAGCTTT
GGCTGAGGCTACAAACCTCTGCAAATGTAGACCACGTAGGCCTCGGCACTGCGTTCGAAAACAGTAAATACGACCAG
GACTACAATATCCGTGTAACCATGTATGTACAATTCAGAGAATTTAATCTTAAAGACCCCCCACTTAAACCC--------

>EF184221_pcv2a

ATGACGTATCCAAGGAGGCGTTTCCGCAGACGAAGACACCGCCCCCGCAGCCATCTTGGCCAGATCCTCCGCCGCCGC
CCCTGGCTCGTCCACCCCCGCCACCGTTACCGCTGGAGAAGGAAAAATGGCATCTTCAACACCCGCCTCTCCCGCACCT
TCGGATATACTGTCAAGGCTACCACAGTCACAACGCCCTCCTGGGCGGTGGACATGATGAGATTTAATATTAACGACT
TTGTTCCCCCGGGAGGGGGGACCAACAAAATCTCTATACCCTTTGAATACTACAGAATAAGAAAGGTTAAGGTTGAAT
TCTGGCCCTGCTCCCCAATCACCCAGGGTGACAGGGGAGTGGGCTCCACTGCTGTTATTCTAGATGATAACTTTGTAA
CAAAGGCCAATGCCCTAACCTATGACCCCTATGTAAACTACTCCTCCCGCCATACAATCCCCCAACCCTTCTCCTACCAC
TCCCGTTACTTCACACCCAAACCTGTACTGGATAGAACTATTGATTACTTCCAGCCAAACAACAAAAAAAATCAGCTTT
GGCTGAGGCTACAAACCTCTGCAAATGTAGACCACGTAGGCCTCGGCACTGCGTTCGAAAACAGTAAATACGACCAG
GACTACAATATCCGTGTAACCATGTATGTACAATTCAGAGAATTTAATCTTAAAGACCCCCCACTTAAACCC--------

>EF371519_pcv2a

ATGACGTATCCAAGGAGGCGTTTCCGCAGAAGAAGACACCGCCCCCGCAGCCATCTTGGCCAGATCCTCCGCCGCCG
CCCCTGGCTCGTCCACCCCCGCCACCGTTACCGCTGGAGAAGGAAAAATGGCATCTTCAACACCCGCCTCTCCCGCACC
TTCGGATATACTGTCAAGGCTACCACAGTCACAACGCCCTCCTGGGCGGTGGACATGATGAGATTTAATATTAACGAC
TTTGTTCCCCCGGGAGGGGGGACCAACAAAATCTCTATACCCTTTGAATACTACAGAATAAGAAAGGTTAAGGTTGAA
TTCTGGCCCTGCTCCCCAATCACCCAGGGTGACAGGGGAGTGGGCTCCACTGCTGTTATTCTAGATGATAACTTTGTAA
CAAAGGCCAATGCCCTAACCTATGACCCCTATGTAAACTACTCCTCCCGCCATACAATCCCCCAACCCTTCTCCTACCAC
TCCCGTTACTTCACACCCAAACCTGTACTGGATAGAACTATTGATTACTTCCAGCCAAACAACAAAAAAAATCAGGTTT
GGCTGAGGCTACAAACCTCTGCAAATGTAGACCACGTAGGCCTCGGCACTGCGTTCGAAAACAGTAAATACGACCAG
GACTACAATATCCGTGTAACCATGTATGTACAATTCAGAGAATTTAATCTTAAAGACCCCCCACTTAAACCC--------

>EU136711_pcv2a

ATGACGTATCCAAGGAGGCGTTTCCGCCGACGAAGACACCGCCCCCGCAGCCATCTTGGCCAGATCCTCCGCCGCCGC
CCCTGGCTCGTCCACCCCCGCCTCCGTTACCGATGGAGAAGGAAAAATGGCATCTTCAACACCCGCCTCTCCCGCACCT
TCGGATATACTGTCAAGGCTACCACAGTCACAACGCCCTCCTGGGCTGTGGACATGATGAGATTTAATATTAACGACT
TTGTTCCCCCGGGAGGGGGGACCAACAAAATCTCTATACCCTTTGAATACTACAGAATAAGAAAGGTTAAGGTTGAAT
TCTGGCCCTGCTCCCCAATCACCCAGGGTGACAGGGGAGTGGGCTCCACTGCTGTTATTCTAGATGATAACTTTGTAA
CAAAGGCCACAGCCCTAACCTATGACCCCTATGTAAACTACTCCTCCCGCCATACAATCCCCCAACCCTTCTCCTACCAC
TCCCGTTACTTCACACCCAAACCTGTACTGGATAGAACTATTGATTACTTCCAGCCAAACAACAAAAAAAATCAGCTTT
GGCTGAGGCTACAAACCTCTGCAAATGTAGACCACGTAGGCCTCGGCACTGCATTCGAAAACAGTAAATACGACCAG
GACTACAATATCCGTGTAACCATGTATGTACAATTCAGAGAATTTAATCTTAAAGACCCCCCACTTAAACCC--------

>AF201307_pcv2a

ATGACGTATCCAAGGAGGCGTTTCCGCAGACGAAGACACCGCCCCCGCAGCCATCTTGGCCAGATCCTCCGCCGCCGC
CCCTGGCTCGTCCACCCCCGCCACCGTTACCGCTGGAGAAGGAAAAATGGCATCTTCAACACCCGCCTCTCCCGCACCT
TCGGATATACTGTCAAGGCTACCACAGTCACAACGCCCTCCTGGGCGGTGGACATGATGAGATTTAATATTAACGACT
TTGTTCCCCCGGGAGGGGGGACCAACAAAATCTCTATACCCTTTGAATACTACAGAATAAGAAAGGTTAAGGTTGAAT
TCTGGCCCTGCTCCCCAATCACCCAGGGTGACAGGGGAGTGGGCTCCACTGCTGTTATTCTAGATGATAACTTTGTAA
CAAAGGCCACAGCCCTAACCTATGACCCCTATGTAAACTACTCCTCCCGCCATACAATCCCCCAACCCTTCTCCTACCAC
TCCCGTTACTTCACACCCAAACCTGTACTGGATAGAACTATTGATTACTTCCAGCCAAACAACAAAAAAAATCAGCTTT
GGCTGAGGCTACAAACCTCTGCAAATGTAGACCACGTAGGCCTCGGCACTGCGTTCGAAAACAGTAAATACGACCAG
GACTACAATATCCGTGTAACCATGTATGTACAATTCAGAGAATTTAATCTTAAAGACCCCCCACTTAAACCC--------

>AY146992_pcv2a

ATGACGTATCCAAGGAGGCGTTTCCGCAGACGAAGACACCGCCCCCGCAGCCATCTTGGCCAGATCCTCCGCCGCCGC
CCCTGGCTCGTCCACCCCCGCCACCGTTACCGCTGGAGAAGGAAAAATGGCATCTTCAACACCCGCCTCTCCCGCACCT
TCGGATATACGGTAAAGGCTAGCACAGTCAGAACGCCCTCCTGGGCGGTGGACATGATGAGATTTAATATTAACGAC
TTTGTTCCCCCGGGAGGGGGGACCAACAAAATCTCTATACCCTTTGAATACTACCGAATAAGAAAGGTGAAGGTTGAA
TTCTGGCCCTGCTCCCCAATCACCCAGGGTGACAGGGGAATTGGATCCAGTGCTATTATTCTAGATGACAACTTTGTGC
CAAAGGTAGGGAACCAAACCTATGACCCATATGTAAACTACTCCTCCCGCCATACAATCCCCCAACCCTTCTCCTACCA
CTCCCGTTACTTCACACCCAAACCTGTACTGGATAGAACTATTGATTACTTCCAGCCAAACAACAAAAAAAATCAGCTT
TGGCTGAGGCTACAAACCTCTGGAAATGTAGACCACGTAGGCCTCGGCACTGCATTCGAAAACAGTAAATACGACCA
GGACTACAATATCCGTGTAACCATGTATGTACAATTCAGAGAATTTAATCTTAAAGACCCCCCACTTCACCCC--------

>JF683391_pcv2a

ATGACGTATCCAAGGAGGCGTTTCCGCAGACGAAGACACCGCCCCCGCAGCCATCTTGGCCAGATCCTCCGCCGCCGC
CCCTGGCTCGTCCACCCCCGCCACCGTTACCGCTGGAGAAGGAAAAATGGCATCTTCAACACCCGCCTCTCCCGCACCT
TCGGATATACTGTCGAGCGTACCACAGTCAAACCGCCCTCCTGGGCGGTGGACATGCTGAGATTTAACATTGACAACT
TTGTTCCCCCGGGAGGGGGGACCAACAAAATCTCTATACCCTTTGAATACTACCGAATAAGAAAGGTGAAGGTTGAAT
TCTGGCCCTGCTCCCCAATCACCCAGGGTGACAGGGGAGTTGGATCCAGTGCTATTATTCTAGATGACAACTTTGTACC
AAAGGTAGGGAAGCAAACCTATGACCCATATGTAAACTACTCCTCCCGCCATACAATCCCCCAACCCTTCTCCTACCAC
TCCCGTTACTTCACACCCAAACCTGTACTGGATAGAACTATTGATTACTTCCAGCCAAACAACAAAAAAAATCAGCTTT
GGCTGAGGCTACAAACCTCTGGAAATGTAGACCACGTAGGCCTCGGCACTGCATTCGAAAACAGTATATACGACCAG
GAGTACAATATCCGTGTAACCATGTATGTACAATTCAGAGAATTTAATCTTAAAGACCCCCCACTTCACCCC--------

>EU148507_pcv2a

ATGACGTATCCAAGGAGGCGTTTCCGACGACGACGAAACCGCCCCCGCAGCCATCTTGGCCAGATCCTCCGCCGCCGC
CCCTGGCTCGTCCACCCCCGCCACCGTTACCGCTGGAGAAGGAAAAATGGCATCTTCAACACCCGCCTCTCCCGCACCT
TCGGATATACTGTCAAGCGTACCACAGTCACACCGCCCTCCTGGGCGGTGGACATGCTGAGATTTAACATTGACAACT
TTGTTCCCCCGGGAGGGGGGACCAACAAAATCTCTATACCCTTTGAATACTACCGAATAAGAAAGGTGAAGGTTGAAT
TCTGGCCCTGTTCCCCAATCACCCAGGGTGACAGGGGAATTGGATCCAGTGCTATTATTCTAGATGACAACTTTGTGCC
AAAGGTAGGGAACCAAACCTATGACCCATATGTAAACTACTCCTCCCGCCATACAATCCCCCAACCCTTCTCCTACCAC
TCCCGTTACTTCACACCCAAACCTGTACTGGATAGAACTATTGATTACTTCCAGCCAAACAACAAAAAAAATCAGCTTT
GGCTGAGGCTACAAACCTCTGGAAATGTGGACCACGTAGGCCTCGGCACTGCGTTCGAAAACAGTAAATACGACCAG
GACTACAATATCCGTGTAACCATGTATGTACAATTCAGAGAATTTAATCTTAAAGACCCCCCACTTCACCCC--------

>EF184222_pcv2a

ATGACGTATCCAAGGAGGCGTTTCCGCAGACGAAGACACCGCCCCCGCAGCCATCTTGGCCAGATCCTCCGCCGCCGC
CCCTGGCTCGTCCACCCCCGCCACCGTTACCGCTGGAGAAGGAAAAATGGCATCTTCAACACCCGCCTCTCCCGCACCT
TCGGATATACTGTCAAGGCTACCACAGTCACAACGCCCTCCTGGGCGGTGGACATGATGAGATTTAATATTAACGACT
TTGTTCCCCCGGGAGGGGGGACCAACAAAATCTCTATACCCTTTGAATACTACAGAATAAGAAAGGTTAAGGTTGAAT
TCTGGCCCTGCTCCCCAATCACCCAGGGTGACAGGGGAGTGGGCTCCACTGCTGTTATTCTAGATGATAACTTTGTAA
CAAAGGCCAATGCCCTAACCTATGACCCCTATGTAAACTACTCCTCCCGCCATACAATCCCCCAACCCTTCTCCTACCAC
TCCCGTTACTTCACACCCAAACCTGTACTGGATAGAACTATTGATTACTTCCAGCCAAACAACAAAAAAAATCAGCTTT
GGCTGAGGCTACAAACCTCTGCAAATGTAGACCACGTAGGCCTCGGCACTGCGTTCGAAAACAGTAAATACGACCAG
GACTACAATATCCGTGTAACCATGTATGTACAATTCAGAGAATTTAATCTTAAAGACCCCCCACTTAACCCT--------

>EF371518_pcv2a

ATGACGTATCCAAGGAGGCGTTTCCGCAGACGAAGACACCGCCCCCGCAGCCATCTTGGCCAGATCCTCCGCCGCCGC
CCCTGGCTCGTCCACCCCCGCCACCGTTACCGCTGGAGAAGGAAAAATGGCATCTTCAACACCCGCCTCTCCCGCACCT
TCGGATATACTGTCAAGGCTACCACAGTCACAACGCCCTCCTGGGCGGTGGACAGGATGAGATTTAATATTAACGACT
TTGTTCCCCCGGGAGGGGGGACCAACAAAATCTCTATACCCTTTGAATACTACAGAATAAGAAAGGTTAAGGTTGAAT
TCTGGCCCTGCTCCCCAATCACCCAGGGTGACAGGGGAGTGGGCTCCACTGCTGTTATTCTAGATGATAACTTTGTAA
CAAAGGCCACTGCCCTAACCTATGACCCCTATGTAAACTACTCCTCCCGCCATACAATCCCCCAACCCTTCTCCTACCAC
TCCCGTTACTTCACACCCAAACCTGTACTGGATAGAACTATTGATTACTTCCAGCCAAACAACAAAAAAAATCAGCTTT
GGCTGAGGCTACAAACCTCTGCAAATGTAGACCACGTAGGCCTCGGCACTGCGTTCGAAAACAGTAAATACGACCAG
GACTACAATATCCGTGTAACCATGTATGTACAATTCAGAGAATTTAATCTTAAAGACCCCCCACTTAACCCT--------

>AY874164_pcv2a

ATGACGTATCCAAGGAGGCGTTTCCGCAGACGAAGACACCGCCCCCGCAGCCATCTTGGCCAGATCCTCCGCCGCCGC
CCCTGGCTCGTCCACCCCCGCCACCGTTACCGCTGGAGAAGGAAAAATGGCATCTTCAACACCCGCCTCTCCCGCACCT
TCGGATATACTGTCAAGGCTACCACAGTCACAACGCCCTCCTGGGCGGTGGACATGCTGAGATTTAATATTAATGACT
TTGTTCCCCCGGGAGGGGGGACCAACAAAATCTCTATACCCTTTGAATACTACAGAATAAGAAAAGTTAAGGTTGAAT
TCTGGCCCTGCTCCCCAATCACCCAGGGTGACAGGGGAGTTGGATCCAGTGCTGTTATTCTAGATGATAATTTTATACC
AAAGGCCACAGCCCTAACCTATGACCCCTATGTAAACTACTCCTCCCGCCATACCATCACCCAACCCTTCTCCTACCACT
CCCGTTACTTCACCCCCAAACCTGTGCTGGATTCCACTATTGATTACTTCCAGCCAAACAACAAAAGAAATCAGATTTG
GCTGAGGCTACAAACCTCTGCAAATGTGGACCATGTAGGACTCGGCACTGCGTTCGAAAACAGTAAATACGACCAGG
ACTACAATATCCGTGTAACCATGTATGTACAATTCAGAGAATTTAATCTGAAAGACCCCCCACTTAAACCC--------

>JF927978_pcv2a

ATGACGTATCCAAGGAGGCGTTTCCGCAGACGGAGACACCGCCCCCGCAGCCATCTTGGCCAGATCCTCCGCCGCCG
CCCCTGGCTCGTCCACCCCCGCCACCGTTACCGCTGGAGAAGGAAAAATGGCATCTTCAACACCCGCCTCTCCCGCACC
TTCGGATATACTGTAAGGGCTAGCACAGTCAGAACGCCCTCCTGGGCGGTGGACATGATGAGATTTAATATTAACGAC
TTTGTTCCCCCGGGAGGGGGGACCAACAAAATCTCTATACCCTTTGAATACTACAGAATAAGAAAGGTTAAGGTTGAA
TTCTGGCCCTGCTCCCCAATCACCCAGGGTGACAGGGGAGTGGGCTCCACTGCTGTTATTCTAGATGATAACTTTGTAA
CTAAGGCCACAGCCCTAACCTATGACCCCTATGTAAACTACTCATCCCGCCATACAATCCCCCAACCCTTCTCCTACCAC
TCCCGGTACTTTACCCCCAAACCTGTCCTTGATTCCACTATTGATTACTTCCAACCAAACAGCAAAAGGAATCAGATTTG
GCTGAGACTACAAACCTCGGCAAATGTGGACCACGTAGGCCTCGGCACTGCGTTCGAAAACAGTAAATACGACCAGG
ACTACAATATCCGTGTAACTATGTATGTACAATTCAGAGAATTTAATCTTAAAGACCCCCCACTTAAACCC--------

>JF683387_pcv2a

ATGACGTATCCAAGGAGGCGTTTCCGCAGACGAAGACACCGCCCCCGCAGCCATCTTGGCCAAATCCTCCGCCGCCGC

CCCTGGCTCGTCCACCCCCGCCACCGTTACCGCTGGAGAAGGAAAAATGGCATCTTCAACACCCGCCTCTCCCGCACCT

TCGGATATACTGTAAAGGCTAGCACAGTCAGAACGCCCTCCTGGGCGGTGGACATGATGAGATTTAATATTAACGACT

TTGTTCCCCCGGGAGGGGGGACCAACAAAATCTCTATACCCTTTGAATACTACAGAATAAGAAAGGTTAAGGTTGAAT

TCTGGCCCTGCTCCCCAATCACCCAGGGTGACAGGGGAGTGGGCTCCACTGCTGTTATTCTAGATGATAACTTTGTACC

TAAGGCCACAGCCCAAACCTATGACCCCTATGTAAACTACTCCTCCCGCCATACAATCCCCCAACCCTTCTCCTACCACT

CCCGGTACTTTACCCCCAAACCTGTCCTTGATTCCACTATTGATTACTTTCAACCAAACAGCAAAAGAAATCAGATTTGG

CTGAGGCTACAAACCTCGGCAAATGTGGACCACGTAGGCCTCGGCACTGCGTTCGAAAACAGTAAATACGACCAGGA

CTACAATATCCGTGTAACTATGTATGTACAATTCAGAGAATTTAACCTTAAAGACCCCCCACTAAAGCCC--------

>AY180396_pcv2a

ATGACGTATCCAAGGAGGCGTTTCCGCAGACGAAGACACCGCCCCCGCAGCCATCTTGGCCAGATCCTCCGCCGCCGC

CCCTGGCTCGTCCACCCCCGCCACCGTTACCGCTGGAGAAGGAAAAATGGCATCTTCAACACCCGCCTCTCCCGCACCT

TCGGATATACTGTAAAGGCTAGCACAGTCAGAACGCCCTCCTGGGCGGTGGACATGATGAGATTTAATATTAACGACT

TTGTTCCCCCGGGAGGGGGGACCAACAAAATCTCTATACCCTTTGAATACTACAGAATAAGAAAGGTTAAGGTTGAAT

TCTGGCCCTGCTCCCCAATCACCCAGGGTGACAGGGGAGTGGGCTCCACTGCTGTTATTCTAGATGATAACTTTGTAA

CAAAGGCCACAGCCCTAACCTATGACCCCTATGTAAACTACTCCTCCCGCCATACAATCCCCCAACCCTTCTCCTACCAC

TCCCGGTACTTTACCCCCAAACCTGTCCTTGATTCCACTATTGATTACTTCCAACCAAACAGCAAAAGGAATCAGATTTG

GCTGAGGCTACAAACCTCGGCAAATGTGGACCACGTAGGCCTCGGCACGGCGTTCGAAAACAGTAAATACGACCAGG

ACTACAATATCCGTGTAACTATGTATGTACAATTCAGAGAATTTAATCTTAAAGACCCCCCACTTAAACCC--------

>AF364094_pcv2a

ATGACGTATCCAAGGAGGCGTTACCGCAGAGGAGGACACCGCCCCCGCAGCCATTTTGGCCAGATCCTCCGCCGCCG

CCCTTGGTTGGTCCACCCCCGCCACTGTTACCGCTGGAGAAGGAAGAATGGCATCTTCAACACCCGCTTCTCCCGCACT

TTCGGATATACTGTGAAGGCTAGCACAGTCCGAACGCCCTCCTGGGCGGTGGACATGATGAGATTTAATATTAACGAC

TTTGTTCCCCCGGGAGGGGGGACCAACAAAATCTCTATACCCTTTGAATACTACAGAATAAGAAAGGTTAAGGTTGAA

TTCTGGCCCTGCTCCCCAATCACCCAGGGTGACAGGGGAGTGGGCTCCACTGCTGTTATTCTAGATGATAACTTTGTAA

CTAAGGCCACAGCCCTAACCTATGACCCCTATGTAAACTACTCCTCCCGCCATACAATCCCCCAACCCTTCTCATACCAC

TCCCGGTACTTTACCCCCAAACCTGTCCTTGATTCCACTATTGATTACTTCCAACCAAACAGCAAAAGGAATCAGCTTTG

GCTGAGGCTACAAACCTCGGCAAATGTGGACCACGTAGGCCTCGGCACTGCGTTCGAAAACAGTAAATACGACCAGG

ACTACAATACCCGTGTAACTATGTATGTACAATTCAGAGAATTTAATCTTAAAGACCCCCCACTTAAACCC--------

>AF154679_pcv2a

ATGACGTATCCAAGGAGGCGTTTCCGCAGACGAAGACACCGCCCCCGCAGCCATCTTGGCCAGATCCTCCGCCGCCGC

CCCTGGCTCGTCCACCCCCGCCAGCGTTACCGCTGGAGAAGGAAAAATGGCATCTTCAACACCCGCCTCTCCCGCACC

TTCGGATATACTGTCAAGGCTAGCACAGTCAGAACGCCCTCCTGGGCGGTGGACATGATGAGATTTGATATTAACGAC

TTTGTTCCCCCGGGAGGGGGGACCAACAAAATCTCTATACCCTTTGAATACTACAGAATAAGAAAGGTTAAGGTTGAA

TTCTGGCCCTGCTCCCCAATCACCCAGGGTGACAGGGGAGTGGGCTCCACTGCTGTTATTCTAGATGATAACTTTGTAA

CTAAGGCCACAGCCCTAACCTATGACCCCTATGTAAACTACTCCTCCCGCCATACAATCCCCCAACCCTTCTCCTACCAC

TCCCGGTACTTTACCCCCAAACCTGTCCTTGATTCCACTATTGATTACTTCCAACCAAACAACAAAAGGAATCAGCTTTG

GCTGAGGCTACAAACCTCGGCAAATGTGGACCACGTAGGCCTCGGCACTGCGTTCGAAAACAGTAAATACAACCAGG

ACTACAATATCCGTGTAACTATGTATGTACAATTCAGAGAATTTAATCTTAAAGACCCCCCACTTAAACCC--------

>AF166528_pcv2a

ATGACGTATCCAAGGAGGCGTTTCCGCAGACGAAGACACCGCCCCCGCAGCCATCTTGGCCAGATCCTCCGCCGCCGC
CCCTGGCTCGTCCACCCCCGCCACCGTTACCGCTGGAGAAGGAAAAATGGCATCTTCAACACCCGCCTCTCCCGCACCT
TCGGATATACTGTCAAGGCTACAACAGTCAGAACGCCCTCCTGGGCGGTGGACATGATGAGATTTAATATTAACGACT
TTGTTCCCCCGGGAGGGGGGACCAACAAAATCTCTATACCCTTTGAATACTACAGAATAAGAAAGGTTAAGGTTGAAT
TCTGGCCCTGTTCCCCAATCACCCAGGGTGACAGGGGAGTGGGCTCCACTGCTGTTATTCTAGATGATAACTTTGTAAC
TAAGGCCACAGCCCTAACCTATGACCCGTATGTAAACTACTCCTCCCGCCATACAATCCCCCAACCCTTCTCCTACCACT
CCCGGTACTTTACCCCCAAACCTGTCCTTGATTCCACTATTGATTACTTCCAACCAAACAACAAAAGGAATCAGCTTTGG
CTGAGGCTACAAACCTCGGCAAATGTGGACCACGTAGGCCTGGGCACTGCGTTCGAAAACAGTAAATACGACCAGGA
CTACAATATCCGTGTAACTATGTATGTACAATTCAGAGAATTTAATCTTAAAGACCCCCCACTTAAACCC--------

>AB426905_pcv2a

ATGACGTATCCAAGGAGGCGTTTCCGCAGGCGAAGACACCGCCCCCGCAGCCATCTTGGCCAGATCCTCCGCCGCCG
CCCCTGGCTCGTCCACCCCCGCCACCGTTACCGCTGGAGACGGAAAAATGGCATCTTCAACACCCGCCTCTCCCGCACC
TTCGGATATACTGTCAAGGCTACCACAGTCAGAACGCCCTCCTGGGCGGTGGACATGATGAGATTTAATATTAACGAC
TTTGTTCCCCCGGGAGGGGGGACCAACAAAATCTCTATACCCTTTGAATACTACAGAATAAGAAAGGTTAAGGTTGAA
TTCTGGCCCTGCTCCCCAATCACCCAGGGTGACAGGGGAGTGGGCTCCACTGCTGTTATTCTAGATGATAACTTTGTAA
CTAAGGCCACAGCTCTAACCTATGACCCCTATGTAAACTACTCCTCCCGCCATACAATCCCCCAACCCTTCTCCTACCAC
TCCCGGTACTTTACCCCCAAACCTGTCCTTGATTCCACTATTGATTACTTCCAACCAAACAACAAAAGGAATCAGCTTTG
GCTGAGGCTACAAACCTCGGCAAATGTGGACCACGTAGGCCTCGGCACTGCGTTCGAAAACAGTAAATACGACCAGG
ACTACAATATCCGTGTAACTATGTATGTACAATTCAGAGAATTTAACCTTAAAGACCCCCCACTTAAACCC--------

>AY146991_pcv2a

ATGACGTATCCAAGGAGGCGTTTCCGCAGACGAAGACACCGCCCCCGCAGCCATCTTGGCCAGATCCTCCGCCGCCGC
CCCTGGCTCGTCCACCCCCGCCACCGTTACCGCTGGAGAAGGAAAAATGGCATCTTCAACACCCGCCTCTCCCGCACCT
TCGGATATACTGTCAAGGCTACCACAGTCAGAACGCCCTCCTGGGCGGTGGACATGATGAGATTTAATATTAACGACT
TTGTTCCCCCGGGAGGGGGGACCAACAAAATCTCTATACCCTTTGAATACTACAGAATAAGAAAGGTTAAGGTTGAAT
TCTGGCCCTGCTCCCCAATCACCCAGGGTGACAGGGGAGTGGGCTCCACTGCTGTTATTCTAGATGATAACTTTGTAA
CTAAGGCCACAGCCCTAACCTATGACCCCTATGTAAACTACTCCTCCCGCCATACAATCCCCCAACCCTTCTCCTACCAC
TCCCGGTACTTTACCCCCAAACCTGTCCTTGATTCCACTATTGATTACTTCCAACCAAACAACAAAAGGAATCAGCTTTG
GCTGAGGCTACAAACCTCGGCAAATGTGGACCACGTAGGCCTCGGCACTGCGTTCGAAAACAGTAAATACGACCAGG
ACTACAATATCCGTGTAACTATGTATGTACAATTCCGAGAATTTAATCTTAAAGACCCCCCACTTAAACCC--------

>AY146993_pcv2a

ATGACGTACCCAAGGAGGCGTTTCCGCCGACGAAGTCGCCGCCCCCGCAGCCATCTTGGCCAGATCCTCCGCCGCCGC
CCCTGGCTCGTCCACCCCCGCCACCGTTACCGCTGGAGAAGGAAAAATGGCATCTTCAACACCCGCCTCTCCCGCACCT
TCGGATATACTGTCAAGGCTACCACAGTCAGTACGCCCTCCTGGGCGGTGGACATGATGAGATTTAATATTAACGACT
TTGTTCCCCCCGGAGGGGGGACCAACAAAATCTCTATACCCTTTGAATACTACAGAATAAGAAAGGTGAAGGTTGAAT
TCTGGCCCTGCTCCCCAATCACCCAGGGTGACAGGGGAGTGGGTTCCACTGCTGTTATTCTAGATGATAACTTTGTAAC
TAAGGCCACAGCCCTAACCTATGACCCCTATGTAAACTACTCCTCCCGCCACACAATCCCCCAACCCTTCTCCTACCACT
CCCGGTACTTTACCCCCAAACCTGTCCTTGATTCCACTATTGATTACTTCCAACCAAACAACAAAAGGAATCAGCTTTGG
CTGAGGCTACAAACCTCGGCAAATGTGGACCACGTAGGCCTCGGCACTGCGTTCGAAAACAGTAAATACGACCAGGA
CTACAATATCCGTGTAACTATGTATGTACAATTCAGAGAATTTAATCTTAAAGACCCCCCACTTAAACCA--------

>FJ483938_pcv2a

ATGACGTATCCAAGGAGGCGTTTCCGCAGACGAAGACACCGCCCCCGCAGCCATCTTGGCCTGATCCTCCGCCGCCGC
CCCTGGCTCGTCCACCCCCGCCACCGTTACCGCTGGAGAAGGAAAAATGGCATCTTCAACACCCGCCTCTCCCGCACCT
TCGGATATACTGTCAAGGCTACCACAGTCAGAACGCCCTCCTGGGCGGTGGACATGATGAGATTTAATATTAACGACT
TTGTTCCCCCGGGAGGGGGGACCAACAAAATCTCTATACCCTTTGAATACTACAGAATAAGAAAGGTTAAGGTTGAAT
TCTGGCCCTGCTCCCCAATCACCCAGGGTGACAGGGGAGTGGGCTCCACTGCTGTTATTCTAGATGATAACTTTGTAA
CTAAGGCCACAGCCCTAACCTATGACCCCTATGTAAACTACTCCTCCCGCCATACAATCCCCCAACCCTTCTCCTACCAC
TCCCGGTACTTTACCCCCAAACCTGTCCTTGATTCCACTATTGATTACTTCCAACCAAACAACAAAAGGAATCAGCTTTG
GCTGAGACTACAAACCTCGGCAAATGTGGACCACGTAGGCCTCGGCACTGCGTTCGAAAACAGTAAATACGACCAGG
ACTACAATATCCGGGTAACTATGTATGTACAATTCAGAGAATTTAATCTTAAAGACCCCCCACTTAAACCC--------

>HQ402903_pcv2a

ATGACGTATCCAAGGAGGCGTTTCCGCAGACGAAGACACCGCCCCCGCAGCCATCTTGGCCTGATCCTCCGCCGCCGC
CCCTGGCTCGTCCACCCCCGCCACCGTTACCGCTGGAGAAGGAAAAATGGCATCTTCAACACCCGCCTCTCCTGCACCT
TCGGATATACTGTCAAGGCTACCACAGTCAGAACGCCCTCCTGGGCGGTGGACATGATGAGATTTAATATTAACGACT
TTGTTCCCCCGGGAGGGGGGACCAACAAAATCTCTATACCCTTTGAATACTACAGAATAAGAAAGGTTAAGGTTGAAT
TCTGGCCCTGCTCCCCAATCACCCAGGGTGACAGGGGAGTGGGCTCCACTGCTGTTATTCTAGATGATAACTTTGTAA
CTAAGGCCACAGCCCTAACCTATGACCCCTATGTAAACTACTCCTCCCGCCATACAATCCCCCAACCCTTCTCCTACCAC
TCCCGGTACTTTACCCCCAAACCTGTCCTTGATTCCACTATTGATTACTTCCAACCAAACAACAAAAGGAATCAGCTTTG
GCTGAGACTACAAACCTCGGCAAATGTGGACCACGTAGGCCTCGGCATTGCGTTCGAAAACAGTACATACGACCAGG
ACTACAATATCCGGGTAACTATGTATGTACAATTCAGAGAATTTAATCTTAAAGACCCCCCACTTAAAACCCTA-----

>HM038034_pcv2a

ATGACGTATCCAAGGAGGCGTTTCCGCAGACGAAGACACCGCCCCCGCAGCCATCTTGGCCTGATCCTCCGCCGCCGC
CCCTGGCTCGTCCACCCCCGCCACCGTTACCGCTGGAGAAGGAAAAATGGCATCTTCAACACCCGCCTCTCCTGCACCT
TCGGATATACTGTCAAGGCTACCACAGTCAGAACGCCCTCCTGGGCGGTGGACATGATGAGATTTAATATTAACGACT
TTGTTCCCCCGGGAGGGGGGACCAACGAAATCTCTATACCCTTTGAATACTACAGAATAAGAAAGGTTAAGGTTGAAT
TCTGGCCCTGCTCCCCAATCACCCAGGGTGACAGGGGAGTGGGCTCCACTGCTGTTATTCTAGATGATAACTTTGTAA
CTAGGGCCACAGCCCTAACCTATGGCCCCTATGTAAACTACTCCTCCCGCCATACAATCCCCCAACCCTTCTCCTACCAC
TCCCGGTACTTTACCCCCAAACCTGTCCTTGATTCCACTATTGATTACTTCCAACCAAACAACAAAAGGAATCAGCTTTG
GCTGAGACTACAAACCTCGGCAAATGTGGACCACGTAGGCCTCGGCATTGCGTTCGAAAACAGTACATACGACCAGG
ACTACAATATCCGGGTAACTATGTATGTACAATTCAGAGAATTTAATCTTAAAGACCCCCCACTTAAACCC--------

>JF682794_pcv2a

ATGACGTATCCAAGGAGGCGTTTCCGCAGACGAAGACACCGCCCCCGCAGCCATCTTGGCCTGATCCTCCGCCGCCGC
CCCTGGCTCGTCCACCCCCGCCACCGTTACCGCTGGAGAAGGAAAAATGGCATCTTCAACACCCGCCTCTCCAGCACCT
TCGGATATACTGTCAAGGCTACCACAGTCAGAACGCCCTCCTGGACGGTGGACATGATTAGATTTAATATTAACGACT
TTGTTCCCCCGGGAGGGGGGACCAACAAAATCTCTATACCCTTTCAATACTACAGAATAAGGAAGGTTAAGGTTGAAT
TCTGGCCCTGCTCCCCAATCATCCAGGGTGACAGGGGAGTGGGCTCCACTGCTGTTATTCTAGATGATAACTTTGTAAC
TAAGGCCACAGCCCTAACCTATGACCCCTATGTAAACTACTCCTCCCGCCATACAATCCCCCAACCCTTCTCCTACCACT
CCCGGTACTTTACCCCCAAACCTGTCCTTGATTCCACTATTGATTACTTCCAACCAAACAACAAAAGGAATCAGCTTTGG
CTGAGACTACAAACCTCGGCAAATGTGGACCACGTAGGCCTCGGCATTGCGTTCGAAAACAGTAAATACGACCAGGA
CTACAATATCCGGGTAACTATGTATGTACAATTCAGAGAATTTAATCTTAAAGACCCCCCACTTAAACCC--------

>HM038033_pcv2a

ATGACGTATCCAAGGAGGCGTTTCCGCAGACGAAGACACCGCCCCCGCAGCCATCTTGGCCTGATCCTCCGCCGCCGC
CCCTGGCTCGTCCACCCCCGCCACCGTTACCGCTGGAGAAGGAAAAATGGCATCTTCAACACCCGCCTCTCCAGCACCT
TCGGATATACTGTCAAGGCTACCACAGTCAGAACGCCCTCCTGGACGGTGGACATGATTAGATTTAATATTAACGACT
TTGTTCCCCCGGGAGGGGGGACCAACAAAATCTCTATACCCTTTCAATACTACAGAATAAGGAAGGTTAAGGTTGAAT
TCTGGCCCTGCTCCCCAATCATCCAGGGTGACAGGGGAGTGGGCTCCACTGCTGTTATTCTAGATGATAACTTTGTAAC
TAAGGCCACAGCCCTAACCTATGACCCCTATGTAAACTACTCCTCCCGCCATACAATCCCCCAACCCTTCTCCTACCACT
CCCGGTACTTTACCCCCAAACCTGTCCTTGATTCCACTATTGATTACTTCCAACCAAACAACAAAAGGAATCAGCTTTGG
CTGAGACTACAAACCTCGGCAAATGTGGACCACGTAGGCCTCGGCATTGCGTTCGAAAACAGTAAATACGACCAGGA
CTACAATATCCGGGTAACTATGTATGTACAATTCAGAGAATTTAATCTTAAAGACCCCCCACTTAAACCC--------

>JF682793_pcv2a

ATGACGTATCCAAGGAGGCGTTTCCGCAGACGAAGACACCGCCCCCGCAGCCATCTTGGCCTGATCCTCCGCCGCCGC
CCCTGGCTCGTCCACCCCCGCCACCGTTACCGCTGGAGAAGGAAAAATGGCATCTTCAACACCCGCCTCTCCAGCACCT
TCGGATATACTGTCAAGGCTACCACAGTCAGAACGCCCTCCTGGACGGTGGACATGATTAGATTTAATATTAACGACT
TTGTTCCCCCGGGAGGGGGGACCAACAAAATCTCTATACCCTTTCAATACTACAGAATAAGGAAGGTTAAGGTTGAAT
TCTGGCCCTGCTCCCCAATCATCCAGGGTGACAGGGGAGTGGGCTCCACTGCTGTTATTCTAGATGATAACTTTGTAAC
TAAGGCCACAGCCCTAACCTATGACCCCTATGTAAACTACTCCTCCCGCCATACAATCCCCCAACCCTTCTCCTACCACT
CCCGGTACTTTACCCCCAAACCTGTCCTTGATTCCACTATTGATTACTTCCAACCAAACAACAAAAGGAATCAGCTTTGG
CTGAGACTACAAACCTCGGCAAATGTGGACCACGTAGGCCTCGGCATTGCGTTCGAAAACAGTAAATACGACCAGGA
CTACAATATCCGGGTAACTATGTATGTACAATTCAGAGAATTTAATCTTAAAGACCCCCCACTTAAACCC--------

>JF682791_pcv2a

ATGACGTATCCAAGGAGGCGTTTCCGCAGACGAAGACACCGCCCCCGCAGCCATCTTGGCCTGATCCTCCGCCGCCGC
CCCTGGCTCGTCCACCCCCGCCACCGTTACCGCTGGAGAAGGAAAAATGGCATCTTCAACACCCGCCTCTCCAGCACCT
TCGGATATACTGTCAAGGCTACCACAGTCAGAACGCCCTCCTGGACGGTGGACATGATTAGATTTAATATTAACGACT
TTGTTCCCCCGGGAGGGGGGACCAACAAAATCTCTATACCCTTTCAATACTACAGAATAAGGAAGGTTAAGGTTGAAT
TCTGGCCCTGCTCCCCAATCATCCAGGGTGACAGGGGAGTGGGCTCCACTGCTGTTATTCTAGATGATAACTTTGTAAC
TAAGGCCACAGCCCTAACCTATGACCCCTATGTAAACTACTCCTCCCGCCATACAATCCCCCAACCCTTCTCCTACCACT
CCCGGTACTTTACCCCCAAACCTGTCCTTGATTCCACTATTGATTACTTCCAACCAAACAACAAAAGGAATCAGCTTTGG
CTGAGACTACAAACCTCGGCAAATGTGGACCACGTAGGCCTCGGCATTGCGTTCGAAAACAGTAAATACGACCAGGA
CTACAATATCCGGGTAACTATGTATGTACAATTCAGAGAATTTAATCTTAAAGACCCCCCACTTAAACCC--------

>AY180397_pcv2a

ATGACGTATCCAAGGAGGCGTTTCCGCAGACGAAGACACCGCCCCCGCAGCCATCTTGGCCAAATCCTCCGCCGCCGC
CCCTGGCTCGTCCACCCCCGCCACCGTTACCGCTGGAGAAGGAAAAATGGCATCTTCAACACCCGCCTCTCCCGCACCT
TCGGATATACTGTCGAGGCTACCACAGTCACAACGCCCTCCTGGACGGTGGACATGATGAGATTTAATATTAACGACT
TTGTTCCCCCGGGAGGGGGGACCAACAAAATCTCTATACCCTTTGAATACTACAGAATAAGAAAGGTTAAGGTTGAAT
TCTGGCCCTGCTCCCCAATCACCCAGGGTGACAGGGGAGTGGGCTCCACTGCTGTTATTCTAGATGATAACTTTGTAA
CAAAGGCCACAGCCCTAACCTATGACCCCTATGTAAACTACTCCTCCCGCCATACAATCCCCCAACCCTTCTCCTACCAC
TCCCGGTACTTTACCCCCAAACCTGTCCTTGATTCCACTATTGATTACTTCCAACCAAACAACAAAAGGAATCAGCTTTG
GCTGAGGCTACAAACCTCGGCAAATGTGGACCACGTAGGCCTCGGCACTGCGTTCGAAAACAGTAAATACGACCAGG
ACTACAATATCCGTGTAACTATGTATGTACAATTCAGAGAATTCAATCTTAAAGACCCCCCACTTAAACCC--------

>AF305533_pcv2a

ATGACGTATCCAAGGAGGCGTTTCCGCAGACGAAGACACCGCCCCCGCAGCCATCTTGGCCAGATCCTCCGCCGCCGC
CCCTGGCTCGTCCACCCCCGCCACCGTTACCGCTGGAGAAGGAAAAATGGCATCTTCAACACCCGCCTCTCCCGCACCT
TCGGATATACTGTCAAGGCTACCACAGTCAGAACGCCCTCCTGGGCGGTGGACATGATGAGATTTAATATTAACGACT
TTGTTCCCCCGGGAGGGGGGACCAACAAAATCTCTATACCCTTTGAATACTACAGAATAAGAAAGGTTAAGGTTGAAT
TCTGGCCCTGCTCCCCAATCACCCAGGGTGACAGGGGAGTGGGCTCCACTGCTGTTATTCTAGATGATAACTTTGTACC
TAAGGCCACAGCCCTAACCTATGACCCCTATGTAAACTACTCCTCCCGCCATACAATCCCCCAACCCTTCTCCTACCACT
CCCGGTACTTTACCCCCAAACCTGTCCTTGATTCCACTATTGATTACTTCCAACCAAACAACAAAAGGAATCAGCTTTGG
CTGAGGCTACAAACCTCGGCAAATGTGGACCACGTAGGCCTCGGCACTGCGTTCGAAAACAGTATATACGACCAGGA
CTACAATATCCGTGTAACCATGTATGTACAATTCAGAGAATTTAATCTTAAAGACCCCCCACTTAAACCC--------

>AF305532_pcv2a

ATGACGTATCCAAGGAGGCGTTTCCGCAGACGAAGACACCGCCCCCGCAGCCATCTTGGCCAGATCCTCCGCCGCCGC
CCCTGGCTCGTCCACCCCCGCCACCGTTACCGCTGGAGAAGGAAAAATGGCATCTTCAACACCCGCCTCTCCCGCACCT
TCGGATATACTGTCAAGGCTACCACAGTCAGAACGCCCTCCTGGGCGGTGGACATGATGAGATTTAATATTAACGACT
TTGTTCCCCCGGGAGGGGGGACCAACAAAATCTCTTTACCCTTTGAATACTACAGAATAAGAAAGGTTAAGGTTGAAT
TCTGGCCCTGCTCCCCAATCACCCAGGGTGACAGGGGAGTGGGCTCCACTGCTGTTATTCTAGATGATAACTTTGTAA
CTAAGGCCACAGCCCTAACCTATGACCCCTATGTAAACTACTCCTCCCGCCATACAATCCCCCAACCCTTCTCCTACCAC
TCCCGGTACTTTACCCCCAAACCTGTCCTTGATTCCACTATTGATTACTTCCAACCAAACAACAAAAGGAATCAGCTTTG
GCTGAGGCTACAAACCTCGGCAAATGTGGACCACGTAGGCCTCGGCACTGCGTTCGAAAACAGTATATACGACCAGG
ACTACAATATCCGTGTAACCATGTATGTACAATTCAGAGAATTTAATCTTAAAGACCCCCCACTTAAACCC--------

>JF927979_pcv2a

ATGACGTATCCAAGGAGGCGTTTCCGCAGACGAAGACACCGCCCCCGCAGCCATCTTGGCCAGATCCTCCGCCGCCGC
CCCTGGCTCGTCCACCCCCGCCACCGTTACCGCTGGAGAAGGAAAAATGGCATCTTCAACACCCGCCTCTCCCGCACCT
TCGGATATACTGTCAAGGCTACCACAGTCAAAACGCCCTCCTGGGCGGTGGACATGATGAGATTTAATATTAACGACT
TTGTTCCCCCGGGAGGGGGGACCAACAAAATCTCTTTACCCTTTGAATACTACAGAATAAGAAAGGTTAAGGTTGAAT
TCTGGCCCTGCTCCCCAATCACCCAGGGTGACAGGGGAGTGGGCTCCACTGCTGTTATTCTAGATGATAACTTTGTAA
CTAAGGCCACAGCCCTAACCTATGACCCCTATGTAAACTACTCCTCCCGCCATACAATCCCCCAACCCTTCTCCTACCAC
TCCCGGTACTTTACCCCCAAACCTGTCCTTGATTCCACTATTGATTACTTCCAACCAAACAACAAAAGGAATCAGCTCTG
GCTGAGGCTACAAACCTCGGCAAATGTGGACCACGTAGGCCTCGGCACTGCGTTCGAAAACAGTATATACGACCAGG
ACTACAATATCCGTGTAACTATGTATGTACAATTCAGAGAATTTAATCTTAAAGACCCCCCACTTAAACCC--------

>AF201309_pcv2a

ATGACGTATCCAAGGAGGCGTTTCCGCAGACGAAGACACCGCCCCCGCAGCCATCTTGGCCAGATCCTCCGCCGCCGC
CCCTGGCTCGTCCACCCCCGCCACCGTTACCGCTGGAGAAGGAAAAATGGCATCTTCAACACCCGCCTCTCCCGCACCT
TCGGATATACTGTCAAGGCTACCACAGTCAGAACGCCCTCCTGGGCGGTGGACATGATGAGATTTAATATTGACGACT
TTGTTCCCCCGGGAGGGGGGACCAACAAAATCTCTATACCCTTTGAATACTACAGAATAAGAAAGGTTAAGGTTGAAT
TCTGGCCCTGCTCCCCAATCACCCAGGGTGATAGGGGAGTGGGCTCCACTGCTGTTATTCTAGATGATAACTTTGTAAC
AAAGGCCACAGCCCTAACCTATGACCCCTATGTAAACTACTCCTCCCGCCATACAATCCCCCAACCCTTCTCCTACCACT
CCCGGTACTTTACCCCCAAACCTGTTCTTGATTCCACTATTGATTACTTCCAACCAAATAACAAAAGGAATCAGCTTTGG
CTGAGGCTACAAACCTCTGCAAATGTGGACCACGTAGGCCTCGGCACTGCCTTCGAAAACAGTAAATACGACCAGGA
CTACAATATCCGTGTAACCATGTATGTACAATTCAGAGAATTTAATCTTAAAGACCCCCCACTTAAACCC--------

>AY256459_pcv2a

ATGACGTATCCAAGGAGGCGTTTCCGCAGACGAAGACACCGCCCCCGCAGCCATCTTGGCCAGATCCTCCGCCGCCGC
CCCTGGCTCCTCCACCCCCGCCACCGTTACCGCTGGAGAAGGAAAAATGGCATCTTCAACACCCGCCTCTCCCGCACCT
TCGGATATACTGTCAAGGCTACCACAGTCAGAACGCCCTCCTGGGCGGTGGACATGATGAGATTTAAAATTGACGACT
TTGTTCCCCCGGGAGGGGGGACCAACAAAATCTCTATACCCTTTGAATACTATAGAATAAGAAAGGTTAAGGTTGAAT
TCTGGCCTTGCTCCCCAATCACCCAGGGTGATAGGGGAGTAGGCTCCACTGCTGTTATTCTGGATGATAACTTTTTTCC
AAAGTCCACAGCCCTAACCTATGACCCCTATGTAAACTACTCCTCCCGCCATACCATAACCCAGCCCTTCTCCTACCACT
CCCGGTACTTTACCCCCAAACCTGTTCTTGATTCCACTATTGATTACTTCCAACCAAATAACAAAAGGAATCAGCTTTGG
CTGAGGCTACAAACCTCTGCAAATGTGGACCACGTAGGCCTCGGCACTGCGTTCGAAAACAGTATATACGACCAGGA
CTACAATATCCGTGTAACCATGTATGTACAATTCAGAGAATTTAATCTTAAAGACCCCCCACTTAAACCC--------

>AF201308_pcv2a

ATGACGTATCCAAGGAGGCGTTTCCGCAGACGAAGACACCGCCCCCGCAGCCATCTTGGCCAGATCCTCCGCCGCCGC
CCCTGGCTCGTCCACCCCCGCCACCGTTACCGCTGGAGAAGGAAAAATGGCATCTTCAACACCCGCCTCTCCCGCACCT
TCGGATATACTGTCAAGGCTACCACAGTCAGAACGCCCTCCTGGGCGGTGGACATGATGAGATTTAATATTGACGACT
TTGTTCCCCCGGGAGGGGGGACCAACAAAATCTCTATACCCTTTGAATACTACAGAATAAGAAAGGTTAAGGTAGAAT
TCTGGCCCTGCTCCCCAATCACCCAGGGTGATAGGGGAGTGGGCTCCACTGCTGTTATTCTAGATGATAACTTTTTTCC
AAAGTCCACAGCCCTAACCTATGACCCCTATGTAAACTACTCCTCCCGCCATACCATACCCCAACCCTTCTCCTACCACT
CCCGGTACTTTACCCCCAAACCTGTTCTTGATTCCACTATTGATTACTTCCAACCAAATAACAAAAGGAATCAGCTTTGG
CTGAGGCTACAAACCTCTGCAAATGTGGACCACGTAGGCCTCGGCACTGCCTTCGAAAACAGTAAATACGACCAGGA
CTACAATATCCGTGTAACCATGTATGTACAATTCAGAGAATTTAATCTTAAAGACCCCCCACTTAAACCC--------

>AF201310_pcv2a

ATGACGTATCCAAGGAGGCGTTTCCGCAGACGAAGACACCGCCCCCGCAGCCATCTTGGCCAGATCCTCCGCCGCCGC
CCCTGGCTCCTCCACCCCCGCCACCGTTACCGCTGGAGAAGGAAAAATGGCATCTTCAACACCCGCCTCTCCCGCACCT
TCGGATATACTGTCAAGGCTACCACAGTCAGAACGCCCTCCTGGGCGGTGGACATGATGAGATTTAAAATTGACGACT
TTGTTCCCCCGGGAGGGGGGACCAACAAAATCTCTATACCCTTTGAATACTACAGAATAAGAAAGGTTAAGGTTGAAT
TCTGGCCCTGCTCCCCAATCACCCAGGGTGATAGGGGAGTGGGCTCCACTGCTGTTATTCTAGATGATAACTTTTTTCC
AAAGTCCACAGCCCTAACCTATGACCCCTATGTAAACTACTCCTCCCGCCATACCATACCCCAGCCCTTCTCCTACCACT
CCCGGTACTTTACCCCCAAACCTGTTCTTGATTCCACTATTGATTACTTCCAACCAAATAACAAAAGGAATCAGCTTTGG
CTGAGGCTACAAACCTCTGCAAATGTGGACCACGTAGGCCTCGGCACTGCCTTCGAAAACAGTAAATACGACCAGGA
CTACAATATCCGTGTAACCATGTATGTACAATTCAGAGAATTTAATCTTAAAGACCCCCCACTTAAACCC--------

>HM623764_pcv2a

ATGACGTATCCAAGGAGGCGTTTCCGCAGACGAAGACACCGCCCCCGCAGCCATCTTGGCCAGATCCTCCGCCGCCGC
CCCTGGCTCCTCCACCCCCGCCACCGTTACCGCTGGAGAAGGAAAAATGGCATCTTCAACACCCGCCTCTCCCGCACCT
TCGGATATACTGTCAAGGCTACCACAGTCAGAACGCCCTCCTGGGCGGTGGACATGATGAGATTTAAAATTGACGACT
TTGTTCCCCCGGGAGGGGGGACCAACAAAATCTCTATACCCTTTGAATACTACAGAATAAGAAAGGTTAAGGTTGAAT
TCTGGCCCTGCTCCCCAATCACCCAGGGTGATAGGGGAGTGGGCTCCACTGCTGTTATTCTAGATGATAACTTTTTTCC
AAAGTCCACAGCCCTAACCTATGACCCCTATGTAAACTACTCCTCCCGCCATACCATACCCCAGCCCTTCTCCTACCACT
CCCGGTACTTTACCCCCAAACCTGTTCTTGATTCCACTATTGATTACTTCCAACCAAATAACAAAAGGAATCAGCTTTGG
CTGAGGCTACAAACCTCTGCAAATGTGGACCACGTAGGCCTCGGCACTGCCTTCGAAAACAGTAAATACGACCAGGA
CTACAATATCCGTGTAACCATGTATGTACAATTCAGAGAATTTAATCTTAAAGACCCCCCACTTAAACCC--------

>AY256455_pcv2a

ATGACGTATCCAAGGAGGCGTTTCCGCCGACGAAGACACCGCCCCCGCAGCCATCTTGGCCAGATCCTCCGCCGCCGC
CCCTGGCTCCTCCACCCCCGCCACCGTTACCGCTGGAGAAGGAAAAATGGCATCTTCAACACCCGCCTCTCCCGCACCT
TCGGATATACTGTCAAGGCTACCACAGTCAGAACGCCCTCCTGGGCGGTGGACATGATGAGATTTAAAATTGACGACT
TTGTTCCCCCGGGAGGGGGGACCAACAAAATCTCTATACCCTTTGAATACTACAGAATAAGAAAGGTTAAGGTAGAAT
TCTGGCCCTGCTCCCCAATCACCCAGGGTGATAGGGGAGTGGGCTCCACTGCTGTTATTCTAGATGATAACTTTTTTCC
AAAGTCCACAGCCCTAACCTATGACCCCTATGTAAACTACTCCTCCCGCCATACCATACCCCAGCCCTTCTCCTACCACT
CCCGGTACTTTACCCCCAAACCTGTTCTTGATTCCACTATTGATTACTTCCAACCAAATAACAAAAGGAATCAGCTTTGG
CTGAGGCTACAAACCTCTGCAAATGTGGACCACGTAGGCCTCGGCACTGCCTTCGAAAACAGTAAATACGACCAGGA
CTACAATATCCGTGTAACCATGTATGTACAATTCAGAGAATTTAATCTTAAAGACCCCCCACTTAAACCC--------

>JN133304_pcv2a

ATGACGTATCCAAGGAGGCGTTTCCGCAGACGAAGACACCGCCCCCGCAGCCATCTTGGCCAGATCCTCCGCCGCCGC
CCCTGGCTCCTCCACCCCCGCCACCGTTACCGCTGGAGAAGGAAAAATGGCATCTTCAACACCCGCCTCTCCCGCACCT
TCGGATATACTGTCAAGGCTACCACAGTCAGAACGCCCTCCTGGGCGGTGGACATGATGAGATTTAAAATTGACGACT
TTGTTCCCCCGGGAGGGGGGACCAACAAAATCTCTATACCCTTTGAATACTACAGAATAAGAAAGGTTAAGGTTGAAT
TCTGGCCCTGCTCCCCAATCACCCAGGGTGATAGGGGAGTGGGCTCCACTGCTGTTATTCTAGATGATAACTTTTTTCC
AAAGTCCACAGCCCTAACCTATGACCCCTATGTAAACTACTCCTCCCGCCATACCATACCCCAGCCCTTCTCCTACCACT
CCCGGTACTTTACCCCCAAACCTGTTCTTGATTCCACTATTGATTACTTCCAACCAAATAACAAAAGGAATCAGCTTTGG
CTGAGGCTACAAACCTCTGCAAATGTGGACCACGTAGGCCTCGGCACTGCCTTCGAAAACAGTAAATACGACCAGGA
CTACAATATCCGTGTAACCATGTATGTACAATTCAGAGAATTTAATCTTAAAGACCCCCCACTTAAACCC--------

>JF683388_pcv2a

ATGACGTATCCAAGGAGGCGTTTCCGCAGACGAAGACACCGCCCCCGCAGCCATCTTGGCCAGATCCTCCGCCGCCGC
CCCTGGCTCGTCCACCCCCGCCACCGTTACCGCTGGAGAAGGAAAAATGGCATCTTCAACGCCCGCCTCTCCCGTACCT
TCGGATATACTGTCAAGGCTACCACAGTCAGCACGCCCTCCTGGGCGGTGGACATGCTGAGATTTAATCTTGACGACT
TTGTTCCCCCGGGAGGGGGGACCAACAAAATCTCCATACCCTTTGAATACTACAGAATAAGAAAGGTTAAGGTTGAAT
TCTGGCCCTGCTCCCCGATCACCCAGGGTGACAGGGGAGTTGGATCCAGTGCTATTATTCTAGATGACAACTTTGTAA
TAAAGGCCACAGCCCAAACCTATGACCCCTATGTAAACTACTCCTCCCGCCATACAATCCCCCAACCCTTCTCCTACCAC
TCCCGTTACTTCACACCCAAACCTGTTCTTGATTCCACTATTGATTACTTCCAACCAAATAACAAAAGGAATCAGCTGTG
GATGAGACTCCAAACCAGTAGAAATGTGGACCACGTAGGCCTCGGCACTGCGTTCGAAAACAGTAAATACGACCAGG
ACTACAATATCCGTGTAACCATGTATGTACAATTCAGGGAATTTAATCTTAAAGACCCCCCACTTAAACCC--------

>HQ831539_pcv2a

ATGACGTATCCAAGGAGGCGTTTCCGCAGACGAAGACACCGCCCCCGGAGCCATCTTGGCCAGATCCTCCGCCGCCG
CCCCTGGCTCCTCCACCCCCGCCACCGTTACCGCTGGAGAAGGAAAAATGGCATCTTCAACGCCCGCCTCTCCCGCACC
TTCGGATATACTGTCAAGGCTACCACAGTCAGCACGCCATCCTGGGCGGTGGACATGCTGAGATTTAATCTTGACGAC
TTTGTTCCCCCGGGAGGGGGGACCAACAAAATCTCTATACCCTTTGAATACTACAGAATAAGAAAGGTTAAGGTTGAA
TTCTGGCCCTGCTCCCCGATCACCCAGGGTGACAGGGGAGTTGGATCCAGTGCTATTATTCTAGATGACAACTTTGTA
ATAAAGGCCACAGCCCAAACCTATGACCCCTATGTAAACTACTCCTCCCGCCATACAATCCCCCAACCCTTCTCCTACCA
CTCCCGTTACTTCACACCCAAACCTGTTCTTGATTCCACTATTGATTACTTCCAACCAAATAACAAAAGGAATCAGCTGT
GGATGAGACTCCAAACCAGTAGAAATGTGGACCACGTAGGCCTCGGCACTGCGTTCGAAAACAGTAAATACGACCAG
GACTACAATATCCGTGTAACCATGTATGTACAATTCAGAGAATTTAATCTTAAAGACCCCCCACTTAAACCC--------

>AF201306_pcv2a

ATGACGTATCCAAGGAGGCGTTTCCGCAGACGCAGACACCGCCCCCGCAGCCATCTTGGCCAGATCCTCCGCCGCCGC
CCCTGGCTCGTCCACCCCCGCCACCGTTACCGCTGGAGAAGGAAAAATGGCATCTTCAACGCCCGCCTCTCCCGCACCT
TCGGATATACTGTCAAGGCTACCACAGTCAGCACGCCCTCCTGGGCGGTGGACATGCTGAGATTTAATCTTGACGACT
TTGTTCCCCCGGGAGGGGGGACCAACAAAATCTCTATACCCTTTGAATACTACAGAATAAGAAAGGTTAAGGTTGAAT
TCTGGCCCTGCTCCCCGATCACCCAGGGTGACAGGGGAGTTGGATCCAGTGCTATTATTCTAGATGACAACTTTGTAA
TAAAGGCCACAGCCCAAACCTATGACCCCTATGTAAACTACTCCTCCCGGCATACAATCCCCCAACCCTTCTCCTACCAC
TCCCGTTACTTCACACCCAAACCTGTTCTTGATTCCACTATTGATTACTTCCAACCAAATAACAAAAGGAATCAGCTGTG
GATGAGACTCCAAACCAGTAGAAATGTGGACCACGTAGGCCTCGGCACTGCGTTCGAAAACAGTAAATACGACCAGG
ACTACAATATCCGTGTAACCATGTATGTACAATTCAGAGAATTTAATCTTAAAGACCCCCCACTTAAACCC--------

>HQ831522_pcv2a

ATGACGTATCCAAGGAGGCGTTTCCGCAGACGAAGACACCGCCCCCGCAGCCATCTTGGCCAGATCCTCCGCCGCCGC
CCCTGGCTCGTCCACCCCCGCCACCGGTACCGCTGGAGAAGGAAAAATGGCATCTTCAACGCCCGCCTCTCCCGCACC
TTCGGATATACTGTCAAGGCTACCACAGTCAGCACGCCCTCCTGGGCGGTGGACATGCTGAGATTTAATCTTGACGAC
TTTGTTCCCCCGGGAGGGGGGACCAACAAAATCTCTATACCCTTTGAATACTACAGAATAAGAAAGATTAAGGTTGAA
TTCTGGCCCTGCTCCCCGATCACCCAGGGTGACAGGGGAGTTGGATCCAGTGCTATTATTCTAGATGACAACTTTGTA
ATAAAGGCCACAGCCCAAACCTATGACCCCTATGTAAACTACTCCTCCCGCCATACAATCCCCCAACCCTTCTCCTACCA
CTCCCGTTACTTCACACCCAAACCTGTTCTTGATTCCACTATTGATTACTTCCAACCAAATAACAAAAGGAATCAGCTGT
GGATGAGACTCCAAACCAGTAGAAATGTGGACCACGTAGGCCTCGGCACTGCGTTCGAAAACAGTAAATACGACCAG
GACTACAATATCCGTGTAACCATGTATGTACAATTCAGAGAATTTAATCTTAAAGACCCCCCACTTAAACCC--------

>AY322004_pcv2a

ATGACGTATCCAAGGAGGCGTTTCCGCAGACGAAGACACCGCCCCCGCAGCCATCTTGGCCAGATACTCCGCCGCCG
CCCCTGGCTCGTCCACCCCCGCCACCGTTACCGCTGGAGAAGGAAAAATGGCATCTTCAACGCCCGCCTCTCCCGCAC
CTTCGGATATACTGTCAAGGCTACCACAGTCAGCACGCCCTCCTGGGCGGTGGACATGCTGAGATTTAATCTTGACGA
CTTTGTTCCCCCGGGAGGGGGGACCAACAAAATCTCTATACCCTTTGAATACTACAGAATAAGAAAGGTTAAGGTTGA
ATTCTGGCCCTGCTCCCCGATCACCCAGGGTGACAGGGGAGTTGGATCCAGTGCTATTATTCTAGATGACAACTTCGT
AATAAAGGCCACAGCCCAAACCTATGACCCCTATGTAAACTACTCCTCCCGCCATACAATCCCCCAACCCTTCTCCTACC
ACTCCCGTTACTTCACACCCAAACCTGTTCTTGATTCCACTATTGATTACTTCCAACCAAATAACAAAAGGAATCAGCTG
TGGATGAGACTCCAAACCAGTAGAAATGTGGACCACGTAGGCCTCGGCACTGCGTTCGAAAACAGTAAATACGACCA
GGACTACAATATCCGTGTAACCATGTATGTACAATTCAGAGAATTTAATCTTAAAGACCCCCCACTTAAACCC--------

>AY874165_pcv2a

ATGACGTATCCAAGGAGGCGTTTCCGCAGACGAAGACACCGCCCCCGCAGCCATCTTGGCCAGATCCTCCGCCGCCGC
CCCTGGCTCGTCCACCCCCGCCACCGTTACCGCTGGAGAAGGAAAAATGGCATCTTCAACGCCCGCCTCTCCCGCACCT
TCGGATATACTGTCAAGGCTACCACAGTCAGCACGCCCTCCTGGGCGGTGGACATGCTGAGATTTAATCTGGACGACT
TTGTTCCCCCGGGAGGGGGGACCAACAAAATCTCTATACCCTTTGAATACTACAGAATAAGAAAGGTTAAGGTTGAAT
TCTGGCCCTGCTCCCCGATCACCCAGGGTGACAGGGGAGTTGGATCCAGTGCTATTATTCTAGATGACAACTTTGTAA
TAAAGGCCACAGCCCAAACCTATGACCCCTATGTAAACTACTCCTCCCGCCATACAATCCCCCAACCCTTCTCCTACCAC
TCCCGTTACTTCACACCCAAACCTGTTCTTGATTCCACTATTGATTACTTCCAACCAAATAACAAAAGGAATCAGCTGTG
GATGAGACTCCAAACCAGTAGAAATGTGGACCACGTAGGCCTCGGCACTGCGTTCGAAAACAGTAAATACGACCAGG
ACTACAATATCCGTGTAACCATGTATGTACAATTCAGAGAATTTAATCTTAAAGACCCCCCACTTAAACCC--------

>HM009337_pcv2a

ATGACGTATCCAAGGAGGCGTTTCCGCAGACGAAGACACCGCCCGCGCAGCCATCTTGGCCAGATCCTCCGCCGCCG

CCCCTGGCTCGTCCACCCCCGCCACCGTTACCGCTGGAGAAGGAAAAATGGCATCTTCAACGCCCGCCTCTCCCGCAC

CTTCGGATATACTGTCAAGGCTACCACAGTCAGCACGCCCTCCTGGGCGGTGGACATGCTGAGATTTAATCTTGACGA

CTTTGTTCCCCCGGGAGGGGGGACCAACAAAATCTCTATACCCTTTGAATACTACAGAATAAGAAAGGTTAAGGTTGA

ATTCTGGCCCTGCTCCCCGATCACCCAGGGTGACAGGGGAGTTGGATCCAGTGCTATTATTCTAGATGACAACTTTGT

AATAAAGGCCACAGCCCAAACCTATGACCCCTATGTAAACTACTCCTCCCGCCATACAATCCCCCAACCCTTCTCCTACC

ACTCCCGTTACTTCACACCCAAACCTGTTCTTGATTCCACTATTGATTACTTCCAACCAAATAACAAAAGGAATCAGCTG

TGGATGAGACTCCAAACCAGTAGAAATGTGGACCACGTAGGCCTCGGCACTGCGTTCGAAAACAGTAAATACGACCA

GGACTACAATATCCGTGTAACCATGTATGTACAATTCAGAGAATTTAATCTTAAAGACCCCCCACTTAAACCC--------

>JX512854_pcv2a

ATGACGTATCCAAGGAGGCGTTTCCGCAGACGAAGACACCGCCCCCGCAGCCATCTTGGCCAGATCCTCCGCCGCCGC

CCCTGGCTCGTCCACCCCCGCCACCGTTACCGCTGGAGAAGGAAAAATGGCATCTTCAACGCCCGCCTCTCCCGCACCT

TCGGATATACTGTCAAGGCTACCACAGTCAGCACGCCCTCCTGGGCGGTGGACATGCTGAGATTTAATCTTGACGACT

TTGTTCCCCCGGGAGGGGGGACCAACAAAATCTCTATACCCTTTGAATACTACAGAATAAGAAAGGTTAAGGTTGAAT

TCTGGCCCTGCTCCCCGATCACCCAGGGTGACAGGGGAGTTGGATCCAGTGCTATTATTCTAGATGACAACTTTGTAA

TAAAGGCCACAGCCCAAACCTATGACCCCTATGTAAACTACTCCTCCCGCCATACAATCCCCCAACCCTTCTCCTACCAC

TCCCGTTACTTCACACCCAAACCTGTTCTTGATTCCACTATTGATTACTTCCAACCAAATAACAAAAGGAATCAGCTGTG

GATGAGACTCCAAACCAGTAGAAATGTGGACCACGTAGGCCTCGGCACTGCGTTCGAAAACAGTAAATACGACCAGG

ACTACAATATCCGTGTAACCATGTACGTACAATTCAGAGAATTTAATCTTAAAGACCCCCCACTTAAACCC--------

>AY874166_pcv2a

ATGACGTATCCAAGGAGGCGTTTCCGCAGACGAAGACACCGCCCCCGCAGCCATCTTGGCCAGATCCTCCGCCGCCGC

CCCTGGCTCGTCCACCCCCGCCACCGTTACCGCTGGAGAAGGAAAAATGGCATCTTCAACGCCCGCCTCTCCCGCACCT

TCGGATATACTGTCAAGGCTACCACAGTCAGCACGCCCTCCTGGGCGGTGGACATGCTGAGATTTAATCTTGACGACT

TTGTTCCCCCGGGAGGGGGGACCAACAAAATCTCTATACCCTTTGAATACTACAGAATAAGAAAGGTTAAGGTTGAAT

TCTGGCCCTGCTCCCCGATCACCCAGGGTGACAGGGGAGTTGGATCCAGTGCTATTATTCTAGATGACAACTTTGTAA

TAAAGGCCACAGCCCAAACCTATGACCCCTATGTAAACTACTCCTCCCGCCATACAATCCCCCAACCCTTCTCCTACCAC

TCCCGTTACTTCACACCCAAACCTGTTCTTGATTCCACCATTGATTACTTCCAACCAAATAACAAAAGGAATCAGCTGTG

GATGAGACTCCAAACCAGTAGAAATGTGGACCACGTAGGCCTCGGCACTGCGTTCGAAAACAGTAAATACGACCAGG

ACTACAATATCCGTGTAACCATGTATGTACAATTCAGAGAATTTAACCTTAAAGACCCCCCACTTAAACCC--------

>EF371520_pcv2a

ATGACGTATCCAAGGAGGCGTTTCCGCAGACGAAGACACCGCCCCCGCAGCCATCTTGGCCAGATCCTCCGCCGCCGC

CCTTGGCTCGTCCACCCCCGCCACCGTTACCGCTGGAGAAGGAAAAATGGCATCTTCAACGCCCGCCTCTCCCGCACCT

TCGGATATACTGTCAAGGCTACCACAGTCCGCACGCCCTCCTGGGCGGTGGACATGCTGAGATTTAATCTTGACGACT

TTGTTCCCCCGGGAGGGGGGACCAACAAAATCTCTATACCCTTTGAATACTACAGAATAAGAAAGGTTAAGGTTGAAT

TCTGGCCCTGCTCCCCGATCACCCAGGGTGACAGGGGAGTTGGATCCAGTGCTATTATTCTAGATGACAACTTTGTAA

TAAAGGCCACAGCCCAAACCTATGACCCCTATGTAAACTACTCCTCCCGCCATACAATCCCCCAACCCTTCTCCTACCAC

TCCCGTTACTTCACACCCAAACCTGTTCTTGATTCCACTGTTGATTACTTCCAACCAAATAACAAAAGGAATCAACTGTG

GATGAGACTCCAAACCAGTAGAAATGTGGACCACGTTGGCCTCGGCACTGCGTTCGAAAACAGTAAATACGACCAGG

ACTACAATATCCGTGTAACCATGTATGTACAATTCAGAGAATTTAATCTTAAAGACCCCCCACTTAAACCC--------

>EF371521_pcv2a

ATGACGTATCCAAGGAGGCGTTTCCGCAGAAGAAGACACCGCCCCCGCAGCCATCTTGGCCAAATCCTCCGCCGCCGC
CCCTGGCTCGTCCACCCCCGCCACCGTTACCGCTGGAGAAGGAAAAATGGCATCTTCAACGCCCGCCTCTCCCGCACCT
TCGGATATACTGTCAAGGCTACCACAGTCAGCACGCCCTCCTGGGCGGTGGACATGCTGAGATTTAATCTTGACGACT
TTGTTCCCCCGGGAGGGGGGACCAACAAAATCTCTATACCCTTTGAATACTACAGAATAAGAAAGGTTAAGGTTGAAT
TCTGGCCCTGCTCCCCGATCACCCAGGGTGACAGGGGAGTTGGATCCAGTGCTATTATTCTAGATGACAACTTTGTAA
TAAAGGCCACAGCCCAAACCTATGACCCCTATGTAAACTACTCCTCCCGCCATACAATCCCCCAACCCTTCTCCTACCAC
TCCCGTTACTTCACACCCAAACCTGTTCTTGATTCCACTATTGATTACTTCCAACCAAATAACAAAAGGAATCAGCTGTG
GATGAGACTCCAAACCAGTAGAAATGTGGACCACGTTGGCCTCGGCACTGCGTTCGAAAACAGTAAATACGACCAGG
ACTACAATATCCGTGTAACCATGTATGTACAATTCAGAGAATTTAATCTTAAAGACCCCCCACTTAAACCC--------

>HQ378164_pcv2a

ATGACGTATCCAAGGAGGCGTTTCCGCAGACGAAGACACCGCCCCCGCAGCCATCTTGGCCAGATCCTCCGCCGCCGC
CCCTGGCTCGTCCACCCCCGCCACCGTTACCGCTGGAGAAGGAAAAATGGCATCTTCAACGCCCGCCTCTCCCGCACCT
TCGGATATACTGTCAAGGCTACCACAGTCAGCACGCCCTCCTGGGCGGTGGACATGCTGAGATTTAATCTTGACGACT
TTGTTCCCCCGGGAGGGGGGACCAACAAAATCTCTATACCCTTTGAATACTACAGAATAAGAAAGGTTAAGGTTGAAT
TCTGGCCCTGCTCCCCGATCACCCAGGGTGACAGGGGAGTTGGATCCAGTGCTATTATTCTAGATGACAACTTTGTAA
TAAAGGCCACAGCCCAAACCTATGACCCCTATGTAAACTACTCCTCCCGCCATACAATCCCCCAACCCTTCTCCTACCAC
TCCCGTTACTTCACACCCAAACCTGTTCTTGATTCCACTATTGATTACTTCCAACCAAATAACAAAAGGAATCAGCTGTG
GATGAGACTCCAAACCAGTAGAAATGTGGACCACGTAGGCCTCGGCACTGCGTTCGAAAACAGTAAATACGACCAGG
ACTACAATATCCGTGTAACCATGTATGTACAATTCAGAGAATTTAATCTTAAAGACCCCCCACTTAAACCC--------

>AF201305_pcv2a

ATGACGTATCCAAGGAGGCGTTTCCGCAGACGAAGACACCGCCCCCGCAGCCATCTTGGCCAGATCCTCCGCCGCCGC
CCCTGGCTCGTCCACCCCCGCCACCGTTACCGCTGGAGAAGGAAAAATGGCATCTTCAACGCCCGCCTCTCCCGCACCT
TCGGATATACTGTCAAGGCTACCACAGTCAGCACGCCCTCCTGGGCGGTGGACATGCTGAGATTTAATCTTGACGACT
TTGTTCCCCCGGGAGGGGGGACCAACAAAATCTCTATACCCTTTGAATACTACAGAATAAGAAAGGTTAAGGTTGAAT
TCTGGCCCTGCTCCCCGATCACCCAGGGTGACAGGGGAGTTGGATCCAGTGCTATTATTCTAGATGACAACTTTGTAA
TAAAGGCCACAGCCCAAACCTATGACCCCTATGTAAACTACTCCTCCCGCCATACAATCCCCCAACCCTTCTCCTACCAC
TCCCGTTACTTCACACCCAAACCTGTTCTTGATTCCACTATTGATTACTTCCAACCAAATAACAAAAGGAATCAGCTGTG
GATGAGACTCCAAACCAGTAGAAATGTGGACCACGTAGGCCTCGGCACTGCGTTCGAAAACAGTAAATACGACCAGG
ACTACAATATCCGTGTAACCATGTATGTACAATTCAGAGAATTTAATCTTAAAGACCCCCCACTTAAACCC--------

>JN006465_pcv2a

ATGACGTATCCAAGGAGGCGTTTCCGCAGACGAAGACACCGCCCCCGCAGCCATCTTGGCCAGATCCTCCGCCGCCGC
CCCTGGCTCGTCCACCCCCGCCACCGTTACCGCTGGAGAAGGAAAAATGGCATCTTCAACGCCCGCCTCTCCCGCACCT
TCGGATATACTGTCAAGGCTACCACAGTCAGCACGCCCTCCTGGGCGGTGGACATGCTGAGATTTAATCTTGACGACT
TTGTTCCCCCGGGAGGGGGGACCAACAAAATCTCTATACCCTTTGAATACTACAGAATAAGAAAGGTTAAGGTTGAAT
TCTGGCCCTGCTCCCCGATCACCCAGGGTGACAGGGGAGTTGGATCCAGTGCTATTATTCTAGATGACAACTTTGTAA
TAAAGGCCACAGCCCAAACCTATGACCCCTATGTAAACTACTCCTCCCGCCATACAATCCCCCAACCCTTCTCCTACCAC
TCCCGTTACTTCACACCCAAACCTGTTCTTGATTCCACTATTGATTACTTCCAACCAAATAACAAAAGGAATCAGCTGTG
GATGAGACTCCAAACCAGTAGAAATGTGGACCACGTAGGCCTCGGCACTGCGTTCGAAAACAGTAAATACGACCAGG
ACTACAATATCCGTGTAACCATGTATGTACAATTCAGAGAATTTAATCTTAAAGACCCCCCACTTAAACCC--------

>AY256458_pcv2a

ATGACGTATCCAAGGAGGCGTTTCCGCAGACGAAGACACCGCCCCCGCAGCCATCTTGGCCAGATCCTCCGCCGCCGC
CCCTGGCTCGTCCACCCCCGCCACCGTTACCGCTGGAGAAGGAAAAATGGTATCTTCAACGCCCGCCTCTCCCGCACCT
TCGGATATACTGTCAAGGCTACCACAGTCAGCACGCCCTCCTGGGCGGTGGACATGCTGAGATTTAATCTTGACGACT
TTGTTCCCCCGGGAGGGGGGACCAACAAAATCTCTATACCCTTTGAATACTACAGAATAAGAAAGGTTAAGGTTGAAT
TCTGGCCCTGCTCCCCGATCACCCAGGGTGACAGGGGAGTTGGATCCAGTGCTATTATTCTAGATGACAACTTTGTAA
TAAAGGCCACAGCCCAAACCTATGACCCCTATGTAAACTACTCCTCCCGCCATACAATCCCCCAACCCTTCTCCTACCAC
TCCCGTTACTTCACACCCAAACCTGTTCTTGATTCCACTATTGATTACTTCCAACCAAATAACAAAAGGAATCAGCTGTG
GATGAGACTCCAAACCAGTAGAAATGTGGACCACGTAGGCCTCGGCACTGCGTTCGAAAACAGTAAATACGACCAGG
ACTACAATATCCGTGTAACCATGTATGTACAATTCAGAGAATTTAATCTTAAAGACCCCCCACTTAAACCC--------

>EF371524_pcv2a

ATGACGTATCCAAGGAGGCGTTTCCGCAGACGAAGACACCGCCCCCGCAGCCATCTTGGCCAGATCCTCCGCCGCCGC
CCCTGGCTCGTCCACCCCCGCCACCGTTACCGCTGGAGAAGGAAAAATGGAATCTTCAACGCCCGCCTCTCCCGCACC
TTCGGATATACTGTCAAGGCAACCACAGTCAGCACGCCCTCCTGGGCGGTGGACATGCTGAGATTTAATCTTGACGAC
TTTGTTCCCCCGGGAGGGGGGACCAACAAAATCTCTATACCCTTTGAATACTACAGAATAAGAAAGGTTAAGGTTGAA
TTCTGGCCCTGCTCCCCGATCACCCAGGGTGACAGGGGAGTTGGATCCAGTGCTATTATTCTAGATGACAACTTTGTA
ATAAAGGCCACAGCCCAAACCTATGACCCCTATGTAAACTACTCCTCCCGCCATACAATCCCCCAACCCTTCTCCTACCA
CTCCCGTTACTTCACACCCAAACCTGTTCTTGATTCCACTATTGATTACTTCCAACCAAATAACAAAAGGAATCAGCTGT
GGATGAGACTCCAAACCAGTAGAAATGTGGACCACGTAGGCCTCGGCACTGCGTTCGAAAACAGTAAATACGACCAG
GACTACAATATCCGTGTAACCATGTATGTACAATTCAGAGAATTTAATCTTAAAGACCCCCCATCCAAACCC--------

>EF371522_pcv2a

ATGACGTATCCAAGGAGGCGTTTCCGCAGACGAAGACACCGCCCCCGCAGCCATCTTGGCCAGATCCTCCGCCGCCGC
CCCTGGCTCGTCCACCCCCGCCACCGTTACCGCTGGAGAAGGAAAAATGGCATCTTCAACGCCCGCCTCTCCCGCACCT
TCGGTTATACTGTCAAGGCTACCACAGTCAGCACGCCCTCCTGGGCGGTGGACATGCTGAGATTTAATCTTGACGACT
TTGTTCCCCCGGGAGGGGGGACCAACAAAATCTCTATACCCTTTGAATACTACAGAATAAGAAAGGTTAAGGTTGAAT
TCTGGCCCTGCTCCCCGATCACCCAGGGTGACAGGGGAGTTGGATCCAGTGCTATTATTCTAGATGACAACTTTGTAA
TAAAGGCCACAGCCCAAACCTATGACCCCTATGTAAACTACTCCTCCCGCCATACAATCCCCCAACCCTTCTCCTACCAC
TCCCGTTACTTCACACCCAAACCTGTTCTTGATTCCACTATTGATTACTTCCAACCAAATAACAAAAGGAATCAGCTGTG
GATGAGACTCCAAACCAGTAGAAATGTGGACCACGTAGGCCTCGGCACTGCGTTCGAAAACAGTAAATACGACCAGG
ACTACAATATCCGTGTAACCATGTATGTACAATTCAGAGAATTTAATCTTAAAGACCCCCCACTTAAACCC--------

>EF371525_pcv2a

ATGACGTATCCAAGGAGGCGTTTCCGCAGAAGAAGACGCCGCCCCCGCAGCCATCTTGGCCAGATCCTCCGCCACCG
CCCCTGGCTCCTCCACCCCCGCCACCGTTACCGCCGGAGAAGGAAAAATGGCATCTTCAACGCCCGCCTCTCCCGCACC
TTCGGATATACTGTCAAGGCAACCACAGTCAGCACGCCCTCCTGGGCGGTGGACATGCTGAGATTTAATCTTGACGAC
TTTGTTCCCCCGGGAGGGGGGACCAACAAAATCTCTATACCCTTTGAATACTACAGAATAAGAAAGGTTAAGGTTGAA
TTCTGGCCCTGCTCCCCGATCACCCAGGGTGACAGGGGAGTTGGATCCAGTGCTATTATTCTACATGACAACTTTGTAA
TAAAGGCCACAGCCCAAACCTATGACCCCTATGTAAACTACTCCTCCCGCCATACAATCCCCCAACCCTTCTCCTACCAC
TCCCGTTACTTCACACCCAAACCTGTTCTTGATTCCACTATTGATTACTTCCAACCAAATAACAAAAGGAATCAGCTGTG
GATGAGACTCCAAACCAGTAGAAATGTGGACCACGTAGGCCTCGGCACTGCCTTCGAAAACAGTAAATACGACCAGG
ACTACAATATCCGTGTAACCATGTATGTACAATTCAGAGAATTTAATCTTAAAGACCCCCCACTTAAACCC--------

>EF067853_pcv2a

ATGACGTATCCAAGGAGGCGTTTCCGCAGACGAAGACACCGCCCCCGCAGCCATCTTGGCCAGATCCTCCGCCGCCGC
CCCTGGCTCGTCCACCCCCGCCACCGTTACCGCTGGAGAAGGAAAAATGGCATCTTCAACGCCCGCCTCTCCCGCACCT
TCGGATATACTGTCAAGGCTACCACAGTCAGCACGCCCTCCTGGGCGGTGGACATGCTGAGATTTAATCTTGACGACT
TTGTTCCCCCGGGAGGGGGGACCAACAAAATCTCTATACCCTTTGAATACTACAGAATAAGAAAGGTTAAGGTTGAAT
TCTGGCCCTGCTCCCCGATCACCCAGGGTGACAGGGGAGTTGGATCCAGTGCTATTATTCTAGATGACAACTTTGTAA
TAAAGGCCACAGCCCAAACCTATGACCCCTATGTAAACTACTCCTCCCGCCATACAATCCCCCAACCCTTCTCCTACCAC
TCCCGTTACTTCACACCCAAACCTGTTCTTGATTCCACTATTGATTACTTCCAACCAAATAACAAAAGGAATCAGCTGTG
GATGAGACTGCAAACCAGTAGAAATGTGGACCACGTAGGCCTCGGCACTGCCTTCGAAAACAGTAAATACGACCAGG
ACTACAATATCCGTGTAACAATGTATGTACAATTCAGAGAATTTAATCTTAAAGACCCCCCACTTAAACCC--------

>JN133305_pcv2a

ATGACGTATCCAAGGAGGCGTTTCCGCAGAAGAAGACACCGCCCCCGCAGCCATCTTGGCCAGATCCTTCGCCGCCGC
CCCTGGCTCGTCCACCCCCGCCACCGTTACCGCTGGAGAAGGAAAAATGGCATCTTCAACGCCCGCCTCTCCCGCACCT
TCGGATATACTGTCAAGGCTACCACAGTCAGCACGCCCTCCTGGGCGGTGGACATGCTGAGATTTAATCTTGACGACT
TTGTTCCCCCGGGAGGGGGGACCAACAAAATCTCTATACCCTTTGAATACTACAGAATAAGAAAGGTTAAGGTTGAAT
TCTGGCCCTGCTCCCCGATCACCCAGGGTGACAGGGGAGTTGGATCCAGTGCTATTATTCTAGATGACAACTTTGTAA
TAAAGGCCAACGCCCAAACCTATGACCCCTATGTAAACTACTCCTCCCGCCATACAATCCCCCAACCCTTCTCCTACCAC
TCCCGTTACTTCACACCCAAACCTGTTCTTGATTCCACTATTGATTACTTCCAACCAAATAACAAAAGGAATCAGCTGTG
GATGAGACTCCAAACCAGTAGAAATGTGGACCACGTAGGCCTCGGCATTGCGTTCGAAAACAGTAAATACGACCAGG
ACTACAATATCCGTGTAACCATGTATGTACAATTCAGAGAATTTAATCTTAAAGACCCCCCACTTAAACCC--------

>HQ831536_pcv2a

ATGACGTATCCAAGGAGGCGTTTCCGCAGACGAAGACACCGCCCCCGCAGCCATCTTGGCCAGATCCTCCGCCGCCGC
CCCTGGCTCGTCCACCCCCGCCACCGTTACCGCTGGAGAAGGAAAAATGGCATCTTCAACGCCCGCCTCTCCCGCACCT
TCGGATATACTGTCAAGGCTACCACAGTCAGCACGCCCTCCTGGGCGGTGGACATGCTGAGATTTAATCTTGACGACT
TTGTTCCCCCGGGAGGGGGGACCAACAAAATCTCTATACCCTTTGAATACTACAGAATAAGAAAGGTTAAGGTTGAAT
TCTGGCCCTGCTCCCCGATCACCCAGGGTGACAGGGGAGTTGGATCCAGTGCTATTATTCTAGATGACAACTTTGTAA
TAAAGGCCAATGCCCAAACCTATGACCCCTATGTAAACTACTCCTCCCGCCATACAATCCCCCAACCCTTCTCCTACCAC
TCCCGTTACTTCACACCCAAACCTGTTCTTGATTCCACTATTGATTACTTCCAACCAAATAACAAAAGGAATCAGCTGTG
GATGAGACTCCAAACCAGTAGAAATGTGGACCACGTAGGCCTCGGCACTGCGTTCGAAAACAGTAAATACGACCAGG
ACTACAATATCCGTGTAACCATGTATGTACAATTCAGAGAATTTAATCTTAAAGACCCCCCACTTAAACCC--------

>HQ831520_pcv2a

ATGACGTATCCAAGGAGGCGTTTCCGCAGACGAAGACACCGCCCCCGAAGCCATCTTGGCCAGATCCTCCGCCGCCG
CCCCTGGCTCGTCCACCCCCGCCACCGTTACCGCTGGAGAAGGAAAAATGGCATCTTCAACGCCCGCCTCTCCCGCAC
CTTCGGATATACTGTCAAGGCTACCACAGTCAGCACGCCCTCCTGGGCGGTGGACATGCTGAGATTTAATCTTGACGA
CTTTGTTCCCCCGGGAGGGGGGACCAACAAAATCTCTATACCCTTTGAATACTACAGAATAAGAAAGGTTAAGGTTGA
ATTCTGGCCCTGCTCCCCGATCACCCAGGGTGACAGGGGAGTTGGATCCAGTGCTATTATTCTAGATGACAACTTTGT
AATAAAGGCCAATGCCCAAACCTATGACCCCTATGTAAACTACTCCTCCCGCCATACAATCCCCCAACCCTTCTCCTACC
ACTCCCGTTACTTCACACCCAAACCTGTTCTTGATTCCACTATTGATTACTTCCAACCAAATAACAAAAGGAATCAGCTG
TGGATGAGACTCCAAACCAGTAGAAATGTGGACCACGTAGGCCTCGGCACTGCGTTCGAAAACAGTAAATACGACCA
GGACTACAATATCCGTGTAACCATGTATGTACAATTCAGAGAATTTAATCTTAAAGACCCCCCACTTAAACCC--------

>HQ831533_pcv2a

ATGACGTATCCAAGGAGGCGTTTCCGCAGACGAAGACACCGCCCCCGGAGCCATCTTGGCCAGATCCTCCGCCGCCG
CCCCTGGCTCGTCCACCCCCGCCACCGTTACCGCTGGAGAAGGAAAAATGGCATCTTCAACGCCCGCCTCTCCCGCAC
CTTCGGATATACTGTCAAGGCTACCACAGTCAGCACGCCCTCCTGGGCGGTGGACATGCTGAGATTTAATCTTGACGA
CTTTGTTCCCCCGGGAGGGGGGACCAACAAAATCTCTATACCCTTTGAATACTACAGAATAAGAAAGGTTAAGGTTGA
ATTCTGGCCCTGCTCCCCGATCACCCAGGGTGACAGGGGAGTTGGATCCAGTGCTATTATTCTAGATGACAACTTTGT
AATAAAGGCCAATGCCCAAACCTATGACCCCTATGTAAACTACTCCTCCCGCCATACAATCCCCCAACCCTTCTCCTACC
ACTCCCGTTACTTCACACCCAAACCTGTTCTTGATTCCACTATTGATTACTTCCAACCAAATAACAAAAGGAATCAGCTG
TGGATGAGACTCCAAACCAGTAGAAATGTGGACCACGTAGGCCTCGGCACTGCGTTCGAAAACAGTAAATACGACCA
GGACTACAATATCCGTGTAACCATGTATGTACAATTCAGAGAATTTAATCTTAAAGACCCCCCACTTAAACCG--------

>AF109399_pcv2a

ATGACGTATCCAAGGAGGCGTTTCCGCAGACGAAGACACCGCCCCCGCAGCCATCTTGGCCAGATCCTCCGCCGCCGC
CCCTGGCTCGTCCACCCCCGCCACCGTTACCGCTGGAAAAGGAAAAATGGCATCTTCAACGCCCGCCTCTCCCGCACCT
TCGGATATACTGTCAAGGCTACCACAGTCAGCACGCCCTCCTGGGCGGTAGACATGCTGAGATTTAATCTTGACGACT
TTGTTCCCCCAGGAGGGGGGACCAACAAAATCTCTATACCCTTTGAATACTACAGAATAAGAAAGGTTAAGGTTGAAT
TCTGGCCCTGCTCCCCGATCACCCAGGGTGACAGGGGAGTTGGATCCAGTGCTATTATTCTAGATGACAACTTTGTAA
TAAAGGCCACAGCCCAAACCTATGACCCCTACGTAAACTACTCCTCCCGCCATACAATCCCCCAACCCTTCTCCTACCAC
TCCAGGTACTTCACACCCAAACCTGTTCTTGATTCCACTATTGATTACTTCCAACCAAATAACAAAAGGAATCAGCTGTG
GATGAGACTCCAAACCAGTAGAAATGTGGACCACGTAGGCCTCGGCACTGCGTTCGAAAACAGTAAATACGACCAGG
ACTACAATATCCGTGTAACCATGTATGTACAATTCAGAGAATTTAATCTTAAAGACCCCCCACTTAAACCC--------

>AB361577_pcv2a

ATGACGTATCCAAGGAGGCGTTTCCGCAGACGAAGACACCGCCCCCGCAGCCATCTTGGCCAGATCCTCCGCCGCCGC
CCCTGGCTCGTCCACCCCCGCCACCGCTACCGCTGGAGAAGGAAAAATGGCATCTTCAACGCCCGCCTCTCCCGCACC
TTCGGTTATACTGTCAAGGCTACCACAGTCAGCACGCCCTCCTGGGCGGTAGACATGCTGAGATTTAATCTTGACGAC
TTTGTTCCCCCAGGAGGGGGGACCAACAAAATCTCTATACCCTTTGAATACTACAGAATAAGAAAGGTTAAGGTTGAA
TTCTGGCCCTGCTCCCCGATCACCCAGGGTGACAGGGGAGTTGGATCCAGTGCTATTATTCTAGATGACAACTTTATAA
TAAAGGCCCCAGCCCAAACCTATGACCCCTATGTAAACTACTCCTCCCGCCATACAATCCCCCAACCCTTCTCCTACCAC
TCCCGTTACTTCACACCCAAACCTGTTCTTGATTCCACTATTGATTACTTCCAACCAAATAACAAAAGGAATCAGCTGTG
GATGAGACTCCAAACCAGTAGAAATGTGGACCACGTAGGCCTCGGCACTGCGTTCGAAAACAGTAAATACGACCAGG
ACTACAATATCCGTGTAACCATGTATGTACAATTCAGAGAATTTAATCTTAAAGACCCCCCACTTAAACCC--------

>AB361576_pcv2a

ATGACGTATCCAAGGAGGCGTTTCCGCAGACGAAGACACCGCCCCCGCAGCCATCTTGCCCAGATCCTCCGCCGCCGC
CCCTGGCTCGTCCACCCCCGCCACCGTTACCGCTGGAGAAGGAAAAATGGCATCTTCAACGCCCGCCTCTCCCGCACCT
TCGGATATACTGTCAAGGCTACCACAGTCAGCACGCCCTCCTGGGCGGTAGACATGCTGAGATTTAATCTTGACGACT
TTGTTCCCCCAGGAGGGGGGACCAACAAAATCTCTATACCCTTTGAATACTACAGAATAAGAAAGGTTAAGGTTGAAT
TCTGGCCCTGCTCCCCGATCACCCAGGGTGACAGGGGAGTTGGATCCAGTGCTATTATTCTAGATGACAACTTTATAAT
AAAGGCCCCAGCCCAAACCTATGACCCCTATGTAAACTACTCCTCCCGCCATACAATCCCCCAACCCTTCTCCTACCACT
CCCGTTACTTCACACCCAAACCTGTTCTTGATTCCACTATTGATTACTTCCAACCAAATAACAAAAGGAATCAGCTGTGG
ATGAGACTCCAAACCAGTAGAAATGTGGACCACGTAGGCCTCGGCACTGCGTTCGAAAACAGTAAATACGACCAGGA
CTACAATATCCGTGTAACCATGTATGTACAATTCAGAGAATTTAATCTTAAAGACCCCCCGCTTAAACCC--------

>AY256456_pcv2a

ATGACGTATCCAAGGAGGCGTTTCCGCAGACGAAGACACCGCCCCCGCAGCCATCTTGGCCAGATCCTCCGCCGCCGC
CCCTGGCTCGTCCACCCCCGCCACCGTTACCGCTGGAGAAGGAAAAATGGCATCTTCAACGCCCGCCTCTCCCGCACCT
TCGGATATACTGTCAAGGCTACCACGGTCAGCACGCCCTCCTGGGCGGTGGACATGCTGAGATTTAATCTTGACGACT
TTGTTCCCCCGGGAGGGGGGACCAACAAAATCTCTATACCCTTTGAATACTACAGAATAAGAAAGGTTAAGGTTGAAT
TCTGGCCCTGCTCCCCGATCACCCAGGGTGACAGGGGAGTGGGATCCAGTGCTATTATTCTAGATGACAACTTTGTAA
TAAAGGCCACAGCCCAAACCTATGACCCCTATGTAAACTACTCCTCCCGCCATACAATCCCCCAACCCTTCTCCTACCAC
TCCCGTTACTTCACACCCAAACCTGTTCTTGATTCCACTATTGATTACTTCCAACCAAATAACAAAAGGAATCAGCTGTG
GATGAGACTCCAAACCAGTAGAAATGTGGACCACGTAGGCCTCGGCACTGCGTTCGAAAACAGTAAATACGACCAGG
ACTACAATATCCGTGTAACCATGTATGTACAATTCAGAGAATTTAATCTTAAAGACCCCCCACTTAAACCC--------

>HQ378166_pcv2a

ATGACGTATCCAAGGAGGCGTTTCCGCAGACGAAGACACCGCCCCCGCAGCCATCTTGGCCAGATCCTCCGCCGCCGC
CCCTGGCTCGTCCACCCCCGCCACCGTTACCGCTGGAGAAGGAAAAATGGCATCTTCAACGCCCGCCTCTCCCGCACCT
TCGGATATACTGTCAAGGCTACCACAGTCAGCACGCCCTCCTGGGCGGTGGACATGCTGAGATTTAATCTTGACGACT
TTGTTCCCCCGGGAGGGGGGACCAACAAAATCTCTATACCCTTTGAATACTACAGAATAAGAAAGGTTAAGGTTGAAT
TCTGGCCCTGCTCCCCGATCACCCAGGGTGACAGGGGAGTTGGATCCAGTGCTATTATTCTAGATGACAACTTTGTAA
TAAAGGCCACAGCCCAAACCTATGACCCCTATGTAAACTACTCCTCCCGCCATACAATCCCCCAACCCTTCTCCTACCAC
TCCCGTTACTTCACACCCAAACCTGTTCTTGATTCCACTATTGATTACTTCCAACCAAATAACAAAAGGAATCAGCTGTG
GATGAGACTACAAACCAGTAGAAATGTGGACCACGTAGGCCTCGGCACTGCGTTCGAAAACAGTAAATACGACCAGG
ACTACAATATCCGTGTAACCATGTATGTACAATTCAGAGAATTTAATCTTAAAGACCCCCCACTTAAACCC--------

>HQ831519_pcv2a

ATGACGTATCCAAGGAGGCGTTTCCGCAGACGAAGACACCGCCCCCGCAGCCATCTTGGCCAGATCCTCCGCCGCCGC
CCCTGGCTCGTCCACCCCCGCCACCGTTACCGCTGGAGAAGGAAAAATGGCATCTTCAACGCCCGCCTCTCCCGTACCT
TCGGATATACTGTCAAGGCTACCACAGTCAGCACGCCCTCCTGGGCGGTGGACATGCTGAGATTTAATCTTGACGACT
TTGTTCCCCCGGGAGGGGGGACCAACAAAATCTCTATACCCTTTGAATACTACAGAATAAGAAAGGTTAAGGTTGAAT
TCTGGCCCTGCTCCCCGATCACCCAGGGTGACAGGGGAGTTGGATCCAGTGCTATTATTCTAGATGACAACTTTGTAA
TAAAGGCCACAGCCCAAACCTATGACCCCTATGTAAACTACTCCTCCCGCCATACAATCCCCCAACCCTTCTCCTACCAC
TCCCGTTACTTCACCCCCAAACCTGTTCTTGATTCCACTATTGATTACTTCCAACCAAATAACAAAAGGAATCAGCTGTG
GATGAGACTCCAAACAAGTAGAAATGTGGACCACGTAGGCCTCGGCACTGCGTTCGAAAACAGTAAATACGACCAGG
ACTACAATATCCGTGTAACCATGTATGTACAATTCAGAGAATTTAATCTTAAAGACCCCCCACTTAAACCC--------

>HQ591374_pcv2a

ATGACGTATCCAAGGAGGCGTTTCCGCAGACGAAGACACCGCCCCCGCAGCCATCTTGGCCAGATCCTCCGCCGCCGC
CCCTGGCTCGTCCACCCCCGCCACCGTTACCGCTGGAGAAGGAAAAATGGCATCTTCAACGCCCGCCTCTCCCGCACCT
TCGGATATACTGTCAAGGCTACCACAGTCAGCACGCCCTCCTGGGCGGTGGACATGCTGAGATTTAATCTTGATGACT
TTGTTCCCCCGGGAGGGGGGACCAACAAAATCTCTATACCCTTTGAATACTACAGAATAAGAAAGGTTAAGGTTGAAT
TCTGGCCCTGCTCCCCGATCACCCAGGGTGACAGGGGAGTTGGATCCAGTGCTATTATTCTAGATGACAACTTTGTAA
TAAAGGCCACAGCCCAAACCTATGACCCCTATGTAAACTACTCCTCCCGCCATACAATCCCCCAACCCTTCTCCTACCAC
TCCCGTTACTTCACACCCAAACCTGTTCTTGATTCCACTATTGATTACTTCCAACCAAATAACAAAAGGAATCAACTGTG
GATGAGACTCCAAACCGGTAGAAATGTGGACCACGTAGGCCTCGGCACTGCGTTCGAAAACAGTAAATACGACCAGG
ACTACAATATCCGTGTAACCATGTATGTACAATTCAGAGAATTTAATCTTAAAGACCCCCCACTTAAACCC--------

>EF371523_pcv2a

ATGACGTATCCAAGGAGGCGTTTCCGCAGACGAAGACACCGCCCCCGCAGCCATCTTGGCCAGATCCTCCGCCGCCGC
CCCTGGCTCGTCCACCCCCGCCACCGTTACCGCTGGAGAAGGAAAAATGGCATCTTCAACGCCCGCCTCTCCCGAACC
TTCGGATATACTGTCAAGGCTACCACAGTCAGCACGCCCTCCTGGGCGGTGGACATGCTGAGATTTAATCTTGATGAC
TTTGTTCCCCCGGGAGGGGGGACCAACAAAATCTCTATACCCTTTGAATACTACAGAATAAGAAAGGTTAAGGTTGAA
TTCTGGCCCTGCTCCCCGATCACCCAGGGTGACAGGGGAGTTGGATCCAGTGCTATTATTCTAGATGACAACTTTGTA
ATAAAGGCCACAGCCCAAACCTATGACCCCTATTTAAACTACTCCTCCCGCCATACAATCCCCCAACCCTTCTCCTACCA
CTCCCGTTACTTCACACCCAAACCTGTTCTTGATTCCACTATTGATTACTTCCAACCAAATAACAAAAGGAATCAGCTGT
GGATGAGACTCCAAACCAGTAGAAATGTGGACCACGTAGGCCTCGGCACTGCGTTCGAAAACAGTAAATACGACCAG
GACTACAATATCCGTGTAACCATGTATGTACAATTCAGAGAATTTAATCTTAAAGACCCCCCACTTAAACCC--------

>HQ591366_pcv2a

ATGACGTATCCAAGGAGGCGTTTCCGCAGACGAAGACACCGCCCCCGCAGCCATCTTGGCCAGATCCTCCGCCGCCGC
CCCTGGCTCGTCCACCCCCGCCACCGTTACCGCTGGAGAAGGAAAAATGGCATCTTCAACGCCCGCCTCTCCCGCACCT
TCGGATATACTGTCAAGGCTACCACAGTCAGCACGCCCTCCTGGGCGGTGGACATGCTGAGATTTAATCTTGATGACT
TTGTTCCCCCGGGAGGGGGGACCAACAAAATCTCTATACCCTTTGAATACTACAGAATAAGAAAGGTTAAGGTTGAAT
TCTGGCCCTGCTCCCCGATCACCCAGGGTGACAGGGGAGTTGGATCCAGTGCTATTATTCTAGATGACAACTTTGTAA
TAAAGGCCACAGCCCAAACCTATGACCCCTATGTAAACTACTCCTCCCGCCATACAATCCCCCAACCCTTCTCCTACCAC
TCCCGTTACTTCACACCCAAACCTGTTCTTGATTCCACTATTGATTACTTCCAACCAAATAACAAAAGGAATCAACTGTG
GATGAGACTCCAAACCAGTAGAAATGTGGACCACGTAGGCCTCGGCACTGCGTTCGAAAACAGTAAATACGACCAGG
ACTACAATATCCGTGTAACCATGTATGTACAATTCAGAGAATTTAATCTTAAAGACCCCCCACTTAAACCC--------

>EF184223_pcv2a

ATGACGTATCCAAGGAGGCGTTTCCGCAGAAGAAGACACCGCCCCCGCAGCCATCTTGGCCAAATCCTCCGCCGCCGC
CCCTGGCTCGTCCACCCCCGCCACCGTTACCGCTGGAGAAGGAAAAATGGCATCTTCAACGCCCGCCTCTCCCGCACCT
TCGGATATACTGTCAAGGCTACCACAGTCAGCACGCCCTCCTGGGCGGTGGACATGCTGAGATTTAATCTTGACGACT
TTGTTCCCCCGGGAGGGGGGACCAACAAAATCTCTATACCCTTTGAATACTACAGAATAAGAAAGGTTAAGGTTGAAT
TCTGGCCCTGCTCCCCGATCACCCAGGGTGACAGGGGAGTTGGATCCAGTGCTATTATTCTAGATGACAACTTTGTAA
TAAAGGCCACAGCCCAAACCTATGACCCCTATGTAAACTACTCCTCCCGCCATACAATCCCCCAACCCTTCTCCTACCAC
TCCCGTTACTTCACACCCAAACCTGTTCTTGATTCCACTATTGATTACTTCCAACCAAATAACAAAAGAAATCAGCTGTG
GATGAGACTCCAAACCAGTAGAAATGTGGACCACGTAGGCCTCGGCACTGCGTTCGAAAACAGTAAATACGACCAGG
ACTACAATATCCGTGTAACCATGTATGTACAATTCAGAGAATTTAATCTTAAAGACCCCCCACTTAAACCC--------

>EF184224_pcv2a

ATGACGTATCCAAGGAGGCGTTTCCGCAGAAGAAGACACCGCCCCCGCAGCCATCTTGGCCAAATCCTCCGCCGCCGC
CCCTGGCTCGTCCACCCCCGCCACCGTTACCGCTGGAGAAGGAAAAATGGCATCTTCAACGCCCGCCTCTCCCGCACCT
TCGGATATACTGTCAAGGCTACCACAGTCAGCACGCCCTCCTGGGCGGTGGACATGCTGAGATTTAATCTTGACGACT
TTGTTCCCCCGGGAGGGGGGACCAACAAAATCTCTATACCCTTTGAATACTACAGAATAAGAAAGGTTAAGGTTGAAT
TCTGGCCCTGCTCCCCGATCACCCAGGGTGACAGGGGAGTTGGATCCAGTGCTATTATTCTAGATGACAACTTTGTAA
TAAAGGCCACAGCCCAAACCTATGACCCCTATGTAAACTACTCCTCCCGCCATACAATCCCCCAACCCTTCTCCTACCAC
TCCCGTTACTTCACACCCAAACCTGTTCTTGATTCCACTATTGATTACTTCCAACCAAATAACAAAAGAAATCAGCTGTG
GATGAGACTCCAAACCAGTAGAAATGTGGACCACGTAGGCCTCGGCACTGCGTTCGAAAACAGTAAATACGACCAGG
ACTACAATATCCGTGTAACCATGTATGTACAATTCAGAGAATTTAATCTTAAAGACCCCCCACTTAAAACC--------

>EF064150_pcv2a

ATGACGTATCCAAGGAGGCGTTACCGTAGACGAAGACACCGCCCCCGCAGCCATCTTGGCCAGATCCTCCGCCGCCG
CCCCTGGCTCCTCCACCCCCGCCACCGTTACCGCTGGAGAAGGAAAAATGGCATCTTCAACGCCCGCCTCTCCCGCACC
TTCGGATATACTGTCAAGGCTACCACAGTCAGCACGCCCTCCTGGGCGGTGGACATGCTGAGATTTAATCTTGACGAC
TTTGTTCCCCCGGGAGGGGGGACCAACAAAATCTCTATACCCTTTGAATACTACAGAATAAGAAAGGTTAAGGTTGAA
TTCTGGCCCTGCTCCCCGATCACCCAGGGTGACAGGGGAGTTGGATCCAGTGCTATAATTCTAGATGACAACTTTGTA
ATAAAGGCCACAGCCCAAACCTACGACCCCTATGTAAACTACTCCTCCCGCCATACAATCCCCCAACCCTTCTCCTACCA
CTCCCGTTACTTCACACCCAAACCTGTTCTTGATTCCACTATTGATTACTTCCAACCAAATAACAAAAGGAATCAGCTGT
GGATGAGACTCCAAACCAGTAGAAATGTGGACCACGTAGGCCTCGGCACTGCCTTCGAAAACAGTAAATACGACCAG
GACTACAATATCCGTGTAACCATGTATGTACAATTCAGAGAATTTAATCTTAAAGACCCCCCACTTAACCCA--------

>AY288135_pcv2a

ATGACGTATCCTAGGAGGCGTTACCGAAGACGAAGACACCGCCCCCGCAGCCATCTTGGCCAAATCCTCCGCCGCCGC
CCCTGGCTCGTCCACCCCCGCCACCGTTACCGCTGGAGAAGGAAAAATGGCATCTTCAACACCCGCCTCTCCCGCACCA
TCGGTTATACTGTCAAGGCTACCACAGTCAGCACGCCCTCCTGGGCGGTGGACATGCTGAGATTTAATCTTGACGACT
TTGTTCCCCCGGGAGGGGGGACCAACAAAATCTCTATACCCTTTGAATACTACAGAATAAGAAAGGTTAAGGTTGAAT
TCTGGCCCTGCTCCCCGATCACCCAGGGTGACAGGGGAGTTGGATCCAGTGCTATTATTCTAGATGACAACTTTGTAA
TAAAGGCCACAGCCCAAACCTATGACCCCTATGTAAACTACTCCTCCCGCCATACAATCCCCCAACCCTTCTCCTACCAC
TCCCGTTACTTCACACCCAAACCTGTTCTTGATTCCACTATTGATTACTTCCAACCAAATAACAAAAGGAATCAGCTGTG
GATGAGACTCCAAACCAGTAGAAATGTGGACCACGTAGGCCTCGGCACTGCGTTCGAAAACAGTAAATACGACCAGG
ACTACAATATCCGTGTAACCATGTATGTACAATTCAGAGAATTTAATCTTAAAGACCCCCCACTTAAACCC--------

>JN382162_pcv2a

ATGACGTATCCAAGGAGGCGTTACCGGAGAAGAAGACACCGCCCCCGCAGCCATCTTGGCCAGATCCTCCGCCGCCG
CCCCTGGCTCGTCCACCCCCGCCACCGTTACCGCTGGAGAAGGAAAAATGGCATCTTCAACGCCCGCCTCTCCCGCAC
CTTCGGATATACTGTCAAGGCTACCACAGTCAGCACGCCCTCCTGGGCGGTGGACATGCTGAGATTTAATCTTGACGA
CTTTGTTCCCCCGGGAGGGGGGACCAACAAAATCTCTATACCCTTTGAATACTACAGAATAAGAAAGGTTAAGGTTGA
ATTCTGGCCCTGCTCCCCGATCACCCAGGGTGACAGGGGAGTTGGATCCAGTGCTATTATTCTAGATGACAACTTTGT
AATAAAGGCCACAGCCCAAACCTATGACCCCTATGTAAACTACTCCTCCCGCCATACAATCCCCCAACCCTTCTCCTACC
ACTCCCGTTACTTCACACCCAAACCTGTTCTTGATTCCACTATTGATTACTTCCAACAAAATAACAAAAGGAATCAGCTG
TGGATGAGACTCCAAACCAGTAGAAATGTGGAACACGTAAGCTTCGGCACTGCGTTCGAAAACAGTAAATACGACCA
GGACTACAATATCCGTGTAACCATGTATGTACAATTCAGAGAATTTAATCTTAAAGACCCCCCACTTAAACCC--------

>JN382161_pcv2a

ATGACGTATCCAAGGAGGCGTTACCGGAGAAGAAGACACCGCCCCCGCAGCCATCTTGGCCAGATCCTCCGCCGCCG
CCCCTGGCTCGTCCACCCCCGCCACCGTTACCGCTGGAGAAGGAAAAATGGCATCTTCAACGCCCGCCTCTCCCGCAC
CTTCGGATATACTGTCAAGGCTACCACAGTCAGCACGCCCTCCTGGGCGGTGGACATGCTGAGATTTAATCTTGACGA
CTTTGTTCCCCCGGGAGGGGGGACCAACAAAATCTCTATACCCTTTGAATACTACAGAATAAGAAAGGTTAAGGTTGA
ATTCTGGCCCTGCTCCCCGATCACCCAGGGTGACAGGGGAGTTGGATCCAGTGCTATTATTCTAGATGACAACTTTGT
AATAAAGGCCACAGCCCAAACCTATGACCCCTATGTAAACTACTCCTCCCGCCATACAATCCCCCAACCCTTCTCCTACC
ACTCCCGTTACTTCACACCCAAACCTGTTCTTGATTCCACTATTGATTACTTCCAACAAAATAACAAAAGGAATCAGCTG
TGGATGAGACTCCAAACCAGTAGAAATGTGGAACACGTAAGCTTCGGCACTGCGTTCGAAAACAGTAAATACGACCA
GGACTACAATATCCGTGTAACCATGTATGTACAATTCAGAGAATTTAATCTTAAAGACCCCCCACTTAAACCC--------

>JN382177_pcv2a

ATGACGTATCCAAGGAGGCGTTACCGGAGAAGAAGACACCGCCCCCGCAGCCATCTTGGCCAGATCCTCCGCCGCCG
CCCCTGGCTCGTCCACCCCCGCCACCGTTACCGCTGGAGAAGGAAAAATGGCATCTTCAACGCCCGCCTCTCCCGCAC
CTTCGGGTATACTGTCAAGGCTACCACAGTCAGCACGCCCTCCTGGGCAGTGGACATGCTGAGATTTAATCTTGACGA
CTTTGTTCCCCCGGGAGGGGGGACCAACAAAATCTCTATACCCTTTGAATACTACAGAATAAGAAAGGTTAAGGTTGA
ATTCTGGCCCTGCTCCCCGATCACCCAGGGTGACAGGGGAGTTGGATCCAGTGCTATTATTCTAGATGACAACTTTGTC
ATAAAAGCAACAGCCCAAACCTATGACCCCTATGTAAACTACTCCTCCCGCCATACAATCCCCCAACCCTTCTCCTACCA
CTCCCGTTACTTCACACCCAAACCTGTTCTTGATTCCACTATTGATTACTTCCAACCAAATAACAAAAGGAATCAGCTGT
GGATGAGACTCCAAACCAGTAGAAATGTGGACCACGTAGGCCTCGGCACTGCGTTCGAAAACAGTAAATACGACCAG
GACTACAATATCCGTGTAACCATGTATGTACAATTCAGAGAATTTAATCTTAAAGACCCCCCACTTAAACCC--------

>AY424401_pcv2a

ATGACGTATCCAAGGAGGCGTTACCGAAGGAGAAGACACCGCCCCCGCAGCCATCTTGGCCAGATCCTCCGCCGCCG
CCCCTGGCTCGTCCACCCCCGCCACCGTTACCGCTGGAGAAGGAAAAATGGCATCTTCAACGCCCGCCTCTCCCGCAC
CTTCGGATATACTGTCAAGGCTACCACAGTCAGCACGCCCTCCTGGGCGGTGGACATGCTGAGATTTAATCTTGACGA
CTTTGTTCCCCCGGGAGGGGGGACCAACAAAATCTCTATACCCTTTGAATACTACAGAATAAGAAAGGTTAAGGTTGA
ATTCTGGCCCTGCTCCCCGATCACCCAGGGTGACAGGGGAGTTGGATCCAGTGCTATTATTCTAGATGACAACTTTGT
AATAAAGGCCACAGCCCAAACCTATGACCCCTATGTAAACTACTCCTCCCGCCATACAATCCCCCAACCCTTCTCCTACC
ACTCCCGTTACTTCACACCCAAACCTGTTCTTGATTCCACTATTGATTACTTCCAACCAAATAACAAAAGGAATCAGCTG
TGGATGAGACTACAAACCAGTAGAAATGTGGACCACGTAGGCCTCGGCACTGCGTTCGAAAACAGTAAATACGACCA
GGACTACAATATCCGTGTAACCATGTATGTACAATTCAGAGAATTTAATCTTAAAGACCCCCCACTTAAACCC--------

>FJ501957_pcv2a

ATGACGTATCCAAGGAGGCGTTACCGAAGGAGAAGACACCGCCCCCGCAGCCATCTTGGCCAGATCCTCCGCCGCCG
CCCCTGGCTCGTCCACCCCCGCCACCGTTACCGCTGGAGAAGGAAAAATGGCATCTTCAACGCCCGCCTCTCCCGCAC
CTTCGGATATACTGTCAAGGCTACCACAGTCAGCACGCCCTCCTGGGCGGTGGACATGCTGAGATTTAATCTTGACGA
CTTTGTTCCCCCGGGAGGGGGGACCAACAAAATCTCTATACCCTTTGAATACTACAGAATAAGAAAGGTTAAGGTTGA
ATTCTGGCCCTGCTCCCCGATCACCCAGGGTGACAGGGGAGTTGGATCCAGTGCTATTATTCTAGATGACAACTTTGT
AATAAAGGCCACAGCCCAAACCTATGACCCCTATGTAAACTACTCCTCCCGCCATACAATCCCCCAACCCTTCTCCTACC
ACTCCCGTTACTTCACACCCAAACCTGTTCTTGATTCCACTATTGATTACTTCCAACCAAATAACAAAAGGAATCAGCTG
TGGATGAGACTACAAACCAGTAGAAATGTGGACCACGTAGGCCTCGGCACTGCGTTCGAAAACAGTAAATACGACCA
GGACTACAATATCCGTGTAACCATGTATGTACAATTCAGAGAATTTAATCTTAAAGACCCCCCACTTAAACCC--------

>NC005148_pcv2a

ATGACGTATCCAAGGAGGCGTTACCGAAGGAGAAGACACCGCCCCCGCAGCCATCTTGGCCAGATCCTCCGCCGCCG
CCCCTGGCTCGTCCACCCCCGCCACCGTTACCGCTGGAGAAGGAAAAATGGCATCTTCAACGCCCGCCTCTCCCGCAC
CTTCGGATATACTGTCAAGGCTACCACAGTCAGCACGCCCTCCTGGGCGGTGGACATGCTGAGATTTAATCTTGACGA
CTTTGTTCCCCCGGGAGGGGGGACCAACAAAATCTCTATACCCTTTGAATACTACAGAATAAGAAAGGTTAAGGTTGA
ATTCTGGCCCTGCTCCCCGATCACCCAGGGTGACAGGGGAGTTGGATCCAGTGCTATTATTCTAGATGACAACTTTGT
AATAAAGGCCACAGCCCAAACCTATGACCCCTATGTAAACTACTCCTCCCGCCATACAATCCCCCAACCCTTCTCCTACC
ACTCCCGTTACTTCACACCCAAACCTGTTCTTGATTCCACTATTGATTACTTCCAACCAAATAACAAAAGGAATCAGCTG
TGGATGAGACTACAAACCAGTAGAAATGTGGACCACGTAGGCCTCGGCACTGCGTTCGAAAACAGTAAATACGACCA
GGACTACAATATCCGTGTAACCATGTATGTACAATTCAGAGAATTTAATCTTAAAGACCCCCCACTTAAACCC--------

>AY424403_pcv2a

ATGACGTATCCAAGGAGGCGTTACCGAAGGAGAAGACACCGCCCCCGCAGCCATCTTGGCCAGATCCTCCGCCGCCG
CCCCTGGCTCGTCCACCCCCGCCACCGTTACCGCTGGAGAAGGAAAAATGGCATCTTCAACGCCCGCCTCTCCCGCAC
CTTCGGATATACTGTCAAGGCTACCACAGTCAGCACGCCCTCCTGGGCGGTGGACATGCTGAGATTTAATCTTGACGA
CTTTGTTCCCCCGGGAGGGGGGACCAACAAAATCTCTATACCCTTTGAATACTACAGAATAAGAAAGGTTAAGGTTGA
ATTCTGGCCCTGCTCCCCGATCACCCAGGGTGACAGGGGAGTTGGATCCAGTGCTATCATTCTAGATGACAACTTTGT
AATAAAGGCCACAGCCCAAACCTATGACCCCTATGTAAACTACTCCTCCCGCCATACAATCCCCCAACCCTTCTCCTACC
ACTCCCGTTACTTCACACCCAAACCTGTTCTTGATTCCACTATTGATTACTTCCAACCAAATAACAAAAGGAATCAGCTG
TGGATGAGACTACAAACCAGTAGAAATGTGGACCACGTAGGCCTCGGCACTGCGTTCGAAAACAGTAAATACGACCA
GGACTACAATATCCGTGTAACCATGTATGTACAATTCAGAGAATTTAATCTTAAAGACCCCCCACTTAAACCC--------

>AY424402_pcv2a

ATGACGTATCCAAGGAGGCGTTACCGAAGGAGAAGACACCGCCCCCGCAGCCATCTTGGCCAGATCCTCCGCCGCCG
CCCCTGGCTCGTCCACCCCCGCCACCGTTACCGCTGGAGAAGGAAAAATGGCATCTTCAACGCCCGCCTCTCCCGCAC
CTTCGGATATACTGTCAAGGCTACCACAGTCAGCACGCCCTCCTGGGCGGTGGACATGCTGAGATTTAATCTTGACGA
CTTTGTTCCCCCGGGAGGGGGGACCAACAAAATCTCTATACCCTTTGAATACTACAGAATAAGAAAGGTTAAGGTTGA
ATTCTGGCCCTGCTCCCCGATCACCCAGGGTGACAGGGGAGTTGGATCCAGTGCTATCATTCTAGATGACAACTTTGT
AATAAAGGCCACAGCCCAAACCTATGACCCCTATGTAAACTACTCCTCCCGCCATACAATCCCCCAACCCTTCTCCTACC
ACTCCCGTTACTTCACACCCAAACCTGTTCTTGATTCCACTATTGATTACTTCCAACCAAATAACAAAAGGAATCAGCTG
TGGATGAGACTACAAACCAGTAGAAATGTGGACCACGTAGGCCTCGGCACTGCGTTCGAAAACAGTAAATACGACCA
GGACTACAATATCCGTGTAACCATGTATGTACAATTCAGAGAATTTAATCTTAAAGACCCCCCACTTAAACCC--------

>DQ870484_pcv2a

ATGACGTATCCAAGGAGGCGTTTCCGCAGACGAAGACACCGCCCCCGCAGCCATCTTGGCCAGATCCTCCGCCGCCGC
CCCTGGCTCGTCCACCCCCGCCACCGTTACCGCTGGAGGAGGAAAAATGGCATTTTCAACGCCCGCCTCTCCCGCACCT
TCGGATATACTGTTAAGGCTACCACAGTCAGCACGCCCTCCTGGGCGGTGGACATGATGAGATTTAATCTTGACGACT
TTGTTCCCCCAGGAGGGGGGACCAACAAAATCTCTATACCCTTTGAATACTACAGAATAAGAAAGGTTAAGGTTGAAT
TCTGGCCCTGCTCCCCGATCACCCAGGGTGACAGGGGAGTTGGATCCAGTGCTATTATTCTAGATGACAACTTTGTAA
TAAAGGCCACAGCCCAAACCTATGACCCCTATGTAAACTACTCCTCCCGCCATACAATCCCCCAACCCTTCTCCTACCAC
TCCCGTTACTTCACACCCAAACCTGTTCTTGATTCCACTATTGATTACTTCCAACCAAATAACAAAAGGAATCAGCTGTG
GATGAGACTCCAAACCAGTAGAAATGTGGACCACGTAGGCCTCGGCACTGCGTTCGAAAACAGTAAATACGACCAGG
ATTACAATATCCGTGTAACCATGTATGTACAATTCAGAGAATTTAATCTTAAAGACCCCCCACTTAAACCC--------

>HQ378165_pcv2a

ATGACGTATCCAAGGAGGCGTTTCCGCAGACGAAGACACCGCCCCCGCAGCCATCTTGGCCAGATCCTCCGCCGCCGC
CCCTGGCTCGTCCACCCCCGCCACCGTTACCGCTGGAGAAGGAAAAATGGCATCTTCAACGCCCGCCTCTCCCGCACCT
TCGGATATACTGTCAAGGCTACCACAGTCAGCACGCCCTCCTGGGCGGTGGACATGCTGAGATTTAATCTTGACGACT
TTGTTCCCCCGGGAGGGGGGACCAACAAAATCTCTATACCCTTTGAATACTACAGAATAAGAAAGGTTAAGGTTGAAT
TCTGGCCCTGCTCCCCGATCACCCAGGGTGACAGGGGAGTTGGATCCAGTGCTATTATTCTAGATGACAACTTTGTAA
TAAAGGCCACAGCCCAAACCTATGACCCCTATGTAAACTACTCCTCCCGCCATACAATCCCCCAACCCTTCTCCTACCAC
TCCCGTTACTTCACACCCAAACCTGTTCTTGATTCCACTATTGATTACTTCCAACCAAATAACAAAAGGAATCAGCTGTG
GCTGAGACTACAAACCAGTAGAAATGTGGACCACGTAGGCCTCGGCACTGCGTTCGAAAACAGTAAATACGACCAGG
ACTACAATATCCGTGTAACCATGTATGTACAATTCAGAGAATTTAATCTTAAAGACCCCCCACTTAAACCC--------

>DQ915588_pcv2a

ATGACGTATCCAAGAAGGCGTTTCCGCAGACGAAGACACCGCCCCCGCAGCCATCTTGGCCAGATCCTCCGCCGCCGC
CCCTGGCTCGTCCACCCCCGCCACCGTTACCGCTGGAGAAGGAAAAATGGCATCTTCAACGCCCGCCTCTCCCGCACCT
TCGGATATACTGTCAAGGGTACCACAGTCAGCACGCCCTCCTGGGCGGTGGACATGCTGAGATTTAATCTTGACGACT
TTGTTCCCCCGGGAGGGGGCTCCAACAAAATCTCTATACCCTTTGAATACTACAGAATAAGAAAGGTTAAGGTTGAAT
TCTGGCCTTGCTCGCCGATCACCCAGGGTGACAGGGGAGTTGGATCCAGTGCTATTATTTTAGATGACAACTTTGTAA
TAAAGGCCACAGCCCAAACCTATGACCCCTATGTAAACTACTCCTCCCGCCATACCATCCCCCAACCCTTCTCCTACCAC
TCCCGTTACTTCACACCCAAACCTGTTCTTGATTCCACTATTGATTACTTCCAACCAAATAACAAAAGGAATCAGCTGTG
GATGAGACTCCAAACTAGTAGAAATGTGGACCACGTAGGCCTCGGCACTGTGTTCGAAAACAGTAAATACGACCAGG
ACTACAATATCCGTGTAACCATGTATGTACAATTCAGAGAATTTAATCTTAAAGACCCCCCACTTAAACCC--------

>JN382157_pcv2a

ATGACGTATCCAAGGAGGCGTTACCGGAGAAGAAGACACCGCCCCCGCAGCCATCTTGGCCAGATCCTCCGCCGCCG
CCCCTGGCTCGTCCACCCCCGCCACCGTTACCGCTGGAGAAGGAAAAATGGCATTTTCAACGCCCGCCTCTCCTGCACC
TTCGGATATACTGTCAAGGCTACCACAGTCAGCACGCCCTCCTGGGCGGTGGACATGCTGAGATTTAATCTAGATGAC
TTTGTTCCCCCGGGAGGGGGGACCAACAAAATCTCTATACCCTTTGAATACTACAGAATAAGAAAGGTTAAGGTTGAA
TTCTGGCCCTGCTCCCCGATCACCCAGGGTGACAGGGGAGTTGGATCCAGTGCTATTATTCTAGATGACAACTTTGTA
ATAAAGGCCACAGCCCAAACCTATGACCCCTATGTAAACTACTCCTCCCGCCATACAATCCCCCAACCCTTCTCCTACCA
CTCCCGTTACTTCACACCCAAACCTGTTCTTGATTCCACCATTGATTACTTCCAACCAAATAACAAAAGGAATCAGCTGT
GGATGAGACTCCAAACCAGTGAAAATGTGGACCACGTAGGCCTCGGCACTGCGTTGGAAAACAGTATATACGACCAG
GAATACAATATCCGTGTAACCATGTATGTACAATTCAGAGAATTTAATCTTAAAGACCCCCCACTTAAACCC--------

>AF264043_pcv2a

ATGACGTATCCAAGGAGGCGTTTCCGCAGACGAAGACACCGCCCCCGCAGCCATCTTGGCCAGATCCTCCGCCGCCGC
CCCTGGCTCGTCCACCCCCGCCACCGTTACCGCTGGAGAAGGAAAAATGGCATCTTCAACGCCCGCCTCTCCCGCACCT
TCGGATATACTGTCAAGGCTACCACAGTCAGCACGCCCTCCTGGGCGGTAGACATGCTGAGATTTAATCTTGACGACT
TTGTTCCCCCAGGAGGGGGGACCAACAAAATCTCTATACCCTTTGAATACTACAGAATAAGAAAGGTTAAGGTTGAAT
TCTGGCCCTGCTCCCCGATCACCCAGGGTGATAGGGGAGTGGGATCCACTGCTGTTATTCTAGATGACAACTTTGTAA
CAAAGGCCACAGCCCTAACCTATGACCCATATGTAAACTACTCCTCCCGCCATACAATCCCCCAACCCTTCTCCTACCAC
TCCCGTTACTTTACACCCAAACCTGTTCTTGACTCCACTATTGATTACTTCCAACCAAATAACAAAAGGAATCAGCTTTG
GATGAGGCTACAAACCTCTAGAAATGTGGACCACGTAGGCCTCGGCACTGCGTTCGAAAACAGTAAATACGACCAGG
ACTACAATATCCGTGTAACCATGTATGTACAATTCAGAGAATTTAATCTTAAAGACCCCCCACTTAAACCC--------

>AF381176_pcv2a

ATGACGTATCCAAGGAGGCGTTTCCGCAGACGAAGACACCGCCCCCGCAGCCATCTTGGCCAGATCCTCCGCCGCCGC
CCCTGGCTCGTCCACCCCCGCCACCGTTACCGCTGGAGAAGGAAAAATGGCATCTTCAACGCCCGCCTCTCCCGCACCT
TCGGATATACTGTCAAGGCTACCACAGTCAGCACGCCCTCCTGGGCGGTGGACATGCTGAGATTTAATCTTGACGACT
TTGTTCCCCCGGGAGGGGGGACCAACAAAATCTCTATACCCTTTGAATACTACAGAATAAGAAAGGTTAAGGTTGAAT
TCTGGCCCTGCTCCCCGATCACCCAGGGTGACAGGGGAGTTGGATCCAGTGCTATTATTCTAGATGACAACTTTGTAA
CAAAGGCCACAGCCCTAACCTATGACCCATATGTAAACTACTCCTCCCGCCATACAATCCCCCAACCCTTCTCCTACCAC
TCCCGTTACTTCACACCCAAACCTGTTCTTGACTCCACTATTGATTACTTCCAACCAAATAACAAAAGGAATCAGCTTTG
GCTGAGGATACAAACCTCTAGAAATGTAGACCACGTAGGCCTCGGCACTGCGTTCGAAAACAGTATATACGACCAGG
ACTACAATATCCGTGTAACCATGTATGTACAATTCAGAGAATTTAATCTTAAAGACCCCCCACTTAAACCC--------

>AF465211_pcv2a

ATGACGTATCCAAGGAGGCGTTACCGCAGAAGAAGACACCGCCCCCGCAGCCATCTTGGCCAGATCCTCCGCCGCCG
CCCCTGGCTCGTCCACCCCCGCCACCGCTACCGTTGGAGAAGGAAAAATGGCATCTTCAACACCCGCCTCTCCCGCACC
TTCGGATATACTGTCAAGCGTACCACAGTCACAACGCCCTCCTGGGCGGTGGACATGATGAGATTTAATATTAACGAC
TTTGTTCCCCCGGGAGGGGGGACCAACAAAATCTCTATACCCTTTGAATACTACAGAATAAGAAAAGTTAAGGTTGAA
TTCTGGCCCTGCTCCCCCATCACCCAGGGTGATAGGGGAGTGGGCTCCACTGCTGTTATTCTAGATGATAACTTTGTAA
CTAAGGCCACAGCCCTAACCTATGACCCCTATGTAAACTACTCCTCCCGCCATACAATCCCCCAACCCTTCTCCTACCAC
TCCCGTTACTTCACACCCAAACCTGTTCTTGACTCCACTATTGATTACTTCCAACCAAATAACAAAAGGAATCAGCTTTG
GCTGAGGCTACAAACCTCTAGAAATGTGGACCACGTAGGCCTCGGCACTGCGTTCGAAAACAGTAAATACGACCAGG
ACTACAATATCCGTGTAACCATGTATGTACAATTCAGAGAATTTAATCTTAAAGACCCCCCACTTAACCCC--------

>AF408635_pcv2a

ATGACGTATCCAAGGAGGCGTTACCGCAGAAGAAGACACCGCCCCCGCAGCCATCTTGGCCAGATCCTCCGCCGCCG
CCCCTGGCTCGTCCACCCCCGCCACCGCTACCGTTGGAGAAGGAAAAATGGCATCTTCAACACCCGCCTCTCCCGCACC
TTCGGATATACTGTCAAGGCTACCACAGTCAGAACGCCCTCCTGGGCAGTGGACATGATGAGATTTACACTTGACGAC
TTTGTTCCCCCGGGAGGGGGGACCAACAAAATCTCTCTACCCTTTGAATACTACAGAATAAGAAAGGTTAAGGTTGAA
TTCTGGCCCTGCTCCCCCATCACCCAGGGCGATAGGGGAGTGGGCTCCACTGCTGTTATTCTAGATGATAACTTTGTAA
CAAAGGCCACAGCCCTAACCTATGACCCATATGTAAACTACTCCTCCCGCCATACAATCCCCCAACCCTTCTCCTACCAC
TCCCGTTACTTCACACCCAAACCTGTTCTTGACTCCACTATTGATTACTTCCAACCAAATAACAAAAGGAATCAGCTTTG
GCTGAGGCTACAAACCTCTAGAAATGTGGACCACGTAGGCCTCGGCACTGCGTTCGAAAACAGTAAATACGACCAGG
ACTACAATATCCGTGTAACCATGTATGTACAATTCAGAGAATTTAATCTTAAAGACCCCCCACTTAAACCC--------

>AY181948_pcv2a

ATGACGTATCCAAGGAGGCGTTACCGCAGAAGAAGACACCGCCCCCGCAGCCATCTTGGCCAGATCCTCCGCCGCCG
CCCCTGGCTCGTCCACCCCCGCCACCGCTACCGTTGGAGAAGGAAAAATGGCATCTTCAACACCCGCCTCTCCCGCACC
TTCGGATATACTGTCAAGGCTACCACAGTCAGAACGCCCTCCTGGGCGGTGGACATGATGAGATTTAAGCTTGACGAC
TTTGTTCCCCCGGGAGGGGGGACCAACAAAATCTCTATACCCTTTGAATACTACAGAATAAGAAAAGTTAAGGTTGAA
TTCTGGCCCTGCTCCCCCATCACCCAGGGTGATAGGGGAGTGGGCTCCACTGCTGTCATTCTAGATGATAACTTTGTAA
CAAAGGCCACAGCCCTAACCTATGACCCATATGTAAACTACTCCTCCCGCCATACAATCCCCCAACCCTTCTCCTACCAC
TCCCGTTACTTCACACCAAAACCTGTTCTTGACTCCACTATTGATTACTTCCAACCAAATAACAAAAGGACTCAGCTTTG
GCTGAGGCTACAAACCTCTAGAAATGTGGACCACGTAGGCCTCGGCACTGCGTTCGAAAACAGTATATACGACCAGG
ACTACAATATCCGTGTAACCATGTATGTACAATTCAGAGAATTTAATCTTAAAGACCCCCCACTTAAACCC--------

>AF027217_pcv2a

ATGACGTATCCAAGGAGGCGTTACCGCAGAAGAAGACACCGCCCCCGCAGCCATCTTGGCCAGATCCTCCGCCGCCG
CCCCTGGCTCGTCCACCCCCGCCACCGCTACCGTTGGAGAAGGAAAAATGGCATCTTCAACACCCGCCTCTCCCGCACC
TTCGGATATACTGTCAAGGCTACCACAGTCAGAACGCCCTCCTGGGCGGTGGACATGATGAGATTTAATATTGACGAC
TTTGTTCCCCCGGGAGGGGGGACCAACAAAATCTCTATACCCTTTGAATACTACAGAATAAGAAAGGTTAAGGTTGAA
TTCTGGCCCTGCTCCCCCATCACCCAGGGTGATAGGGGAGTGGGCTCCACTGCTGTTATTCTAGATGATAACTTTGTAA
CAAAGGCCACAGCCCTAACCTATGACCCATATGTAAACTACTCCTCCCGCCATACAATCCCCCAACCCTTCTCCTACCAC
TCCCGTTACTTCACACCCAAACCTGTTCTTGACTCCACTATTGATTACTTCCAACCAAATAACAAAAGGACTCAGCTTTG
GCTGAGGCTACAAACCTCTAGAAATGTGGACCACGTAGGCCTCGGCACTGCGTTCGAAAACAGTATATACGACCAGG
ACTACAATATCCGTGTAACCATGTATGTACAATTCAGAGAATTTAATCTTAAAGACCCCCCACTTAAACCC--------

>KC618389_pcv2a

ATGACGTATCCAAGGAGGCGTTACCGCAGAAGAAGACACCGCCCCCGCAGCCATCTTGGCCAGATCCTCCGCCGCCG
CCCCTGGCTCGTCCACCCCCGCCACCGCTACCGTTGGAGAAGGAAAAATGGCATCTTCAACACCCGCCTCTCCCGCACC
TTCGGATATACTGTCAAGGCTACCACAGTCAGAACGCCCTCCTGGGCGGTGGACATGATGAGATTTAATATTGACGAC
TTTGTTCCCCCGGGAGGGGGGACCAACAAAATATCCATACCCTTTGAATACTACAGAATAAGAAAGGTTAAGGTTGAA
TTCTGGCCCTGCTCCCCCATCACCCAGGGTGATAGGGGAGTGGGCTCCACTGCTGTTATTCTAGATGATAACTTTGTAA
CAAAGGCCACAGCCCTAACCTATGACCCATATGTAAACTACTCCTCCCGCCATACAATCCCCCAACCCTTCTCCTACCAC
TCCCGTTACTTCACACCCAAACCTGTTCTTGACTCCACTATTGATTACTTCCAACCAAATAACAAAAGGAATCAGCTTTG
GCTGAGGCTACAAACCTCTAGAAATGTGGACCACGTAGGCCTCGGCACGGCGTTCGAAAACAGTATATACGACCAGG
ACTACAATATCCGTGTAACCATGTATGTACAATTCAGAGAATTTAATCTTAAAGACCCCCCACTTAAACCC--------

>AF147751_pcv2a

ATGACGTATCCAAGGAGGCGTTACCGCAGAAGAAGACACCGCCCCCGCAGCCATCTCGGCCAGATCCTCCGCCGCCG
CCCCTGGCTCGTCCACCCCCGCCACCGCTACCGTTGGAGAAGGAAAAATGGCATCTTCAACACCCGCCTCTCCCGCACC
TTCGGATATACTGTCAAGGCTACCACAGTCAGAACGCCCTCATGGGCGGTGGACATGATGAGATTTAATATTGACGAC
TTTGTTCCCCCGGGAGGGGGGACCAACAAAATATCCATACCCTTTGAATACTACAGAATAAGAAAGGTTAAGGTTGAA
TTCTGGCCCTGCTCCCCCATCACCCAGGGTGATAGGGGAGTGGGCTCCACTGCTGTTATTCTAGATGATAACTTTGTAA
CAAAGGCCACAGCCCTAACCTATGACCCATATGTAAACTACTCCTCCCGCCATACAATCCCCCAACCCTTCTCCTGCCAC
TCCCGTTACTTCACACCCAAACCTGTTCTTGACTCCACTATTGATTACTTCCAACCAAATAACAAAAGGAATCAGCTTTG
GCTGAGGCTACAAACCTCTAGAAATGTGGACCACGTAGGCCTCGGCACGGCGTTCGAAAACAGTATATACGACCAGG
ACTACAATATCCGTGTAACCATGTATGTACAATTCAGAGAATTTAATCTTAAAGACCCCCCACTTAAACCC--------

>AF264042_pcv2a

ATGACGTATCCAAGGAGGCGTTACCGCAGAAGAAGACACCGCCCCCGCAGCCATCTTGGCCAGATCCTCCGCCGCCG
CCCCTGGCTCGTCCACCCCCGCCACCGCTACCGTTGGAGAAGGAAAAATGGCATCTTCAACACCCGCCTCTCCCGCACC
TTCGGATATACTGTCAAGGCTACCACAGTCAGAACGCCCTCCTGGGCGGTGGACATGATGAGATTTAATATTGACGAC
TTTGTTCCCCCGGGAGGGGGGACCAACAAAATCTCTATACCCTTTGAATACTACAGAATAAGAAAGGTTAAGGTTGAA
TTCTGGCCCTGCTCCCCCATCACCCAGGGTGATAGGGGAGTGGGCTCCACTGCTGTTATTCTAGATGATAACTTTGTAA
CAAAGGCCACAGCCCTAACCTATGACCCATATGTAAACTACTCCTCCCGCCATACAATCCCCCAACCCTTCTCCTACCAC
TCCCGTTACTTCACACCCAAACCTGTTCTTGACTCCACCATTGATTACTTCCAACCAAATAACAAAAGGAATCAGCTTTG
GATGAGGCTACAAACCTCTAGAAATGTGGACCACGTAGGCCTCGGCACTGCGTTCGAAAACAGTATATACGACCAGG
ACTACAATATCCGTGTAACCATGTATGTACAATTCAGAGAATTTAATCTTAAAGACCCCCCACTTAAACCC--------

>AF118095_pcv2a

ATGACGTATCCAAGGAGGCGTTACCGCAGAAGAAGACACCGCCCCCGCAGCCATCTTGGCCAGATCCTCCGCCGCCG
CCCCTGGCTCGTCCACCCCCGACACCGCTACCGTTGGAGAAGGAAAAATGGCATCTTCAACACCCGCCTCTCCCGCACC
TTCGGATATACTGTCAAGGCTACCACAGTCAGAACGCCCTCCTGGGCGGTGGACATGATGAGATTTAATATTGACGAC
TTTGTTCCCCCGGGAGGGGGGACCAACAAAATCTCTATACCCTTTGAATACTACAGAATAAGAAAGGTTAAGGTTGAA
TTCTGGCCCTGCTCCCCCATCACCCAGGGTGACAGGGGAGTGGGCTCCACTGCTGTTATTCTAGATGATAACTTTGTAA
CAAAGGCCACAGCCCTAACCTATGACCCATATGTAAACTACTCCTCCCGCCATACAATCCCCCAACCCTTCTCCTACCAC
TCCCGTTACTTCACACCCAAACCTGTTCTTGACTCCACTATTGATTACTTCCAACCAAATAACAAAAGGAATCAGCTTTG
GATGAGGCTACAAACCTCTAGAAATGTGGACCACGTAGGCCTCGGCACTGCGTTCGAAAACAGTAAATACGACCAGG
ACTACAATATCCGTGTAACCATGTATGTACAATTCAGAGAATTTAATCTTAAAGACCCCCCACTTAAACCC--------

>EU747125_pcv2a

ATGACGTATCCAAGGAGGCGTTACCGCAGAAGAAGACACCGCCCCCGCAGCCATCTTGGCCAGATCCTCCGCCGCCG
CCCCTGGCTCGTCCACCCCCGCCACCGCTACCGTTGGAGAAGGAAAAATGGCATCTTCAACACCCGCCTCTCCCGCACC
TTCGGATATACTGTCAAGGCTACCACAGTCAGAACGCCCTCCTGGGCGGTGGACATGATGAGATTTAATATTGACGAC
TTTGTTCCCCCGGGAGGGGGGACCAACAAAATCTCTATACCCTTTGAATACTACAGAATAAGAAAGGTCAAGGTTGAA
TTCTGGCCCTGCTCCCCCATCACCCAGGGTGATAGGGGAGTGGGCTCCACTGCTGTTATTCTAGATGATAACTTTGTAA
CAAAGGCCACAGCCCTAACCTATGACCCATATGTAAACTACTCCTCCCGCCATACAATCCCCCAACCCTTCTCCTACCAC
TCCCGTTACTTCACACCCAAACCTGTTCTTGACTCCACTATTGATTACTTCCAACCAAATAACAAAAGGAATCAGCTTTG
GCTGAGGCTACAAACCTCTAGAAATGTAGATCACGTAGGCCTCGGCACTGCGTTCGAAAACAGTAAATACGACCAGG
ACTACAATATCCGTGTAACCATGTATGTACAATTCAGAGAATTTAATCTTAAAGACCCCCCACTTAAACCC--------

>AB072301_pcv2a

ATGACGTATCCAAGGAGGCGTTACCGCAGAAGAAGACACCGCCCCCGCAGCCATCTTGGCCAGATCCTCCGCCGCCG
CCCCTGGCTCGTCCACCCCCGCCACCGCTACCGTTGGAGAAGGAAAAACGGCATCTTCAACACCCGCCTCTCCCGCAC
CTTCGGATATACTGTCAAGGCTACCACAGTCACAACGCCCTCCTGGGCGGTGGACATGATGAGATTTACTATTGACGA
CTTTGTTCCCCCGGGAGGGGGGACCAACAAAATCTCTGTACCCTTTGAATACTACAGAATAAGAAAGGTTAAGGTTGA
ATTCTGGCCCTGCTCCCCCATCACCCAGGGTGATAGGGGAGTGGGCTCCACTGCTGTTATTCTAGATGATAACTTTGTA
ACAAAGGCCCCAGCCCTAACCTATGACCCATATGTAAACTACTCCTCCCGCCATACAATCCCCCAACCCTTCTCCTACCA
CTCCCGTTACTTCACACCCAAACCTGTTCTTGACTCCACTATTGATTACTTCCAACCAAATAACAAAAGGAATCAGCTTT
GGCTGAGGCTACAAACCTCTAGAAATGTGGACCACGTAGGCCTCGGCACTGCGTTCGAAAACAGTAAATACGACCAG
GACTACAATATCCGTGTAACCATGTATGTACAATTCAGAGAATTTAATCTTAAAGACCCCCCACTTAAACCC--------

>AY099498_pcv2a

ATGACGTATCCAAGGAGGCGTTACCGCAGAAGAAGACACCGCCCCCGCAGCCATCTTGGCCAGATCCTCCGCCGCCG
CCCCTGGCTCGTCCACCCCCGCCACCGCTACCGTTGGAGAAGGAAAAATGGCATCTTCAACACCCGCCTCTCCCGCACC
TTCGGATATACTGTCAAGGCTACCACAGTCACAACGCCCTCCTGGGCGGTGGACATGATGAGATTTAATATTGACGAC
TTTGTTCCCCCGGGAGGGGGGACCAACAAAATCTCTATACCCTTTGAATACTACAGAATAAGAAAAGTTAAGGTTGAA
TTCTGGCCCTGCTCCCCCATCACCCAGGGTGATAGGGGAGTGGGCTCCACTGCTGTTATTCTAGATGATAACTTTGTAA
CAAAGGCCTCAGCCCTAACCTATGACCCATATGTAAACTACTCCTCCCGCCATACAATCCCCCAACCCTTCTCCTACCAC
TCCCGTTACTTCACACCCAAACCTGTTCTTGACTCCACTATTGATTACTTCCAACCAAATAACAAAAGGAATCAGCTTTG
GATGAGGATACAAACCTCTAGAAATGTAGACCACGTAGGCCTCGGCACTGCGTTCGAAAACAGTAAATACGACCAGG
ACTACAATATCCGTGTAACCATGTATGTACAATTCAGAGAATTTAATCTTAAAGACCCCCCACTTAAACCC--------

>AJ223185_pcv2a

ATGACGTATCCAAGGAGGCGTTACCGCAGAAGAAGACACCGCCCCCGCAGCCATCTTGGCCAGATCCTCCGCCGCCG
CCCCTGGCTCGTCCACCCCCGCCACCGCTACCGTTGGAGAAGGAAAAATGGCATCTTCAACACCCGCCTCTCCCGCACC
TTCGGATATACTGTCAAGGCTACCACAGTCAGAACGCCGTCCTGGGCGGTGGACATGATGAGATTTAATATTGACGAC
TTTGTTCCCCCGGGAGGGGGGACCAACAAAATCTCTATACCCTTTGAATACTACAGAATAAGAAAGGTTAAGGTTGAA
TTCTGGCCCTGCTCCCCCATCACCCAGGGTGATAGGGGAGTGGGCTCCACTGCTGTTATTCTAGATGATAACTTTGTAA
CAAAGGCCACAGCCCTAACCTATGACCCATATGTAAACTACTCCTCCCGCCATACAATCCCCCAACCCTTCTCCTACCAC
TCCCGTTACTTCACACCCAAACCTGTTCTTGACTCCACTATTGATTACTTCCAACCAAATAACAAAAGGAATCAGCTTTG
GCTAAGGCTACAAACCTCTAGAAATGTGGACCACGTAGGCCTCGGCACTGCGTTCGAAAACAGTAAATACGACCAGG
ACTACAATATCCGTGTAACCATGTATGTACAATTCAGAGAATTTAATCTTAAAGACCCCCCACTTAAACCC--------

>AF086834_pcv2a

ATGACGTATCCAAGGAGGCGTTACCGCAGAAGAAGACACCGCCCCCGCAGCCATCTTGGCCAGATCCTCCGCCGCCG
CCCCTGGCTCGTCCACCCCCGCCACCGCTACCGTTGGAGAAGGAAAAATGGCATCTTCAACACCCGCCTCTCCCGCACC
TTCGGATATACTGTCAAGGCTACCACAGTCACAACGCCCTCCTGGGCGGTGGACATGATGAGATTTAATATTGACGAC
TTTGTTCCCCCGGGAGGGGGGACCAACAAAATCTCTATACCCTTTGAATACTACAGAATAAGAAAGGTTAAGGTTGAA
TTCTGGCCCTGCTCCCCCATCACCCAGGGTGATAGGGGAGTGGGCTCCACTGCTGTTATTCTAGATGATAACTTTGTAA
CAAAGGCCACAGCCCTAACCTATGACCCATATGTAAACTACTCCTCCCGCCATACAATCCCCCAACCCTTCTCCTACCAC
TCCCGTTACTTCACACCCAAACCTGTTCTTGACTCCACTATTGATTACTTCCAACCAAATAACAAAAGGAATCAGCTTTG
GCTGAGGCTACAAACCTCTAGAAATGTGGACCACGTAGGCCTCGGCACTGCGTTCGAAAACAGTAAATACGACCAGG
ACTACAATATCCGTGTAACCATGTATGTACAATTCAGAGAATTTAATCTTAAAGACCCCCCACTTAAACCC--------

>AF264040_pcv2a

ATGACGTATCCAAGGAGGCGTTACCGCAGAAGAAGACACCGCCCCCGCAGCCATCTTGGCCAGATCCTCCGCCGCCG
CCCCTGGCTCGTCCACCCCCGCCACCGCTACCGTTGGAGAAGGAAAAATGGCATCTTCAACACCCGCCTCTCCCGCACC
TTCGGATATACTGTAAAGGCTACCACAGTCACAACGCCCTCCTGGGCGGTAGACATGATGAGATTTAATATTGACGAC
TTTGTTCCCCCGGGAGGGGGGACCAACAAAATCTCTATACCCTTTGAATACTACAGAATAAGAAAGGTTAAGGTTGAA
TTCTGGCCCTGCTCCCCCATCACCCAGGGTGATAGGGGAGTGGGCTCCACTGCTGTTATTCTAGATGATAACTTTGTAA
CAAAGGCCACAGCCCTAACCTATGACCCATATGTAAACTACTCCTCCCGCCATACAATCCCCCAACCCTTCTCCTACCAC
TCCCGTTACTTCACACCCAAACCTGTTCTTGACTCCACTATTGATTACTTCCAACCAAATAACAAAAGGAATCAGCTTTG
GCTGAGGCTACAAACCTCTAGAAATGTGGACCACGTAGGCCTCGGCACTGCGTTCGAAAACAGTAAATACGACCAGG
ACTACAATATCCGTGTAACCATGTATGTACAATTCAGAGAATTTAATCTTAAAGACCCCCCACTTAAACCC--------

>AF381177_pcv2a

ATGACGTATCCAAGGAGGCGTTACCGCAGAAGAAGACACCGCCCCCGCAGCCATCTTGGCCAGATCCTCCGCCGCCG
CCCCTGGCTCGTCCACCCCCGCCACCGCTACCGTTGGAGAAGGAAAAATGGCATCTTCAACACCCGCCTCTCCCGCACC
TTCGGATATACTGTCAAGGCTACCACAGTCAGAACGCCCTCCTGGGCGGTGGACATGATGAGATTTAAAATTGACGAC
TTTGTTCCCCCGGGAGGGGGGACCAACAAAATCTCTATACCCTTTGAATACTACAGAATAAGAAAGGTTAAGGTTGAA
TTCTGGCCCTGCTCCCCCATCACCCAGGGTGATAGGGGAGTGGGCTCCACTGCTGTTATTCTAGATGATAACTTTGTAA
CAAAGGCCACAGCCCTAACCTATGACCCATATGTAAACTACTCCCCCCGCCATACAATCCCCCAACCCTTCTCCTACCAC
TCCCGTTACTTCACACCCAAACCTGTTCTTGACTCCACTATTGATTACTTCCAACCAAATAACAAAAGGAATCAGCTTTG
GCTGAGGCTACAAACCTCTGGAAATGTGGACCACGTAGGCCTCGGCACTGCGTTCGAAAACAGTAAATACGACCAGG
ACTACAATATCCGTGTAACCATGTATGTACAATTCAGAGAATTTAATCTTAAAGACCCCCCACTTAATCCC--------

>AF381175_pcv2a

ATGACGTATCCAAGGAGGCGTTACCGCAGAAGAAGACACCGCCCCCGCAGCCATCTTGGCCAGATCCTCCGCCGCCG
CCCCTGGCTCGTCCACCCCCGCCACCGCTACCGTTGGAGAAGGAAAAATGGCATCTTCAACACCCGCCTCTCCCGCACC
TTCGGATATACTGTCAAGGCTACCACAGTCAGAACGCCCTCCTGGGCGGTGGACATGATGAGATTTAATATTGACGAC
TTTGTTCCCCCGGGAGGGGGGACCAACAAAATCTCTATACCCTTTGAATACTACAGAATAAGAAAGGTTAAGGTTGAA
TTCTGGCCCTGCTCCCCCATCACCCAGGGTGATAGGGGAGTGGGCTCCACTGCTGTTATTCTAGATGATAACTTTGTAA
CAAAGGCCACAGCCCTAACCTATGACCCATATGTAAACTACTCCTCCCGCCATACAATCCCCCAACCCTTCTCCTACCAC
TCCCGTTACTTCACACCCAAACCTGTTCTTGACTCCACTATTGATTACTTCCAACCAAATAACAAAGGGAATCAGCTTTG
GCCGAGGATACAAACCTCTAGAAATGTAGACCACGTAGGCCTCGGCACTGCGTTCGAAAACAGTATATACGACCAGG
ACTACAATATCCGTGTAACCATGTATGTACAATTCAGAGAATTTAATCTTAAAGACCCCCCACTTAAACCC--------

>AB462388_pcv2a

ATGACGTATCCAAGGAGGCGTTACCGCAGAAGAAGACACCGCCCCCGCAGCCATCTTGGCCAGATCCTCCGCCGCCG
CCCCTGGCTCGTCCACCCCCGCCACCGCTACCGTTGGAGAAGGAAAAATGGCATCTTCAACACCCGCCTCTCCCGCACC
TTCGGATATACTGTCAAGGCTACCACAGTCAGAACGCCCTCCTGGGCGGTGGACATGATGAGATTTAATATTGACGAC
TTTGTTCCCCCGGGAGGGGGGACCAACAAAATCTCTATACCCTTTGAATACTACAGAATAAGAAAGGTTAAGGTTGAA
TTCTGGCCCTGCTCCCCCATCACCCAGGGTGATAGGGGAGTGGGCTCCACTGCTGTTATTCTAGATGATAACTTTGTAC
CAAAGGCCAACGCCCAAACCTATGACCCATATGTAAACTACTCCTCCCGCCATACAATCCCCCAACCCTTCTCCTACCAC
TCCCGTTACTTCACACCCAAACCTGTTCTTGACTCCACTATTGATTACTTCCAACCAAATAACAAAAGGAATCAGCTTTG
GCTGAGGATACAAACCTCTAGAAATGTAGACCACGTAGGCCTCGGCACTGCGTTCGAAAACAGTATATACGACCAGG
ACTACAATATCCGTGTAACCATGTATGTACAATTCAGAGAATTTAATCTTAAAGACCCCCCACTTAAACCC--------

>EF514717_pcv2a

ATGACGTATCCAAGGAGGCGTTACCGCAGAAGAAGACACCGCCCCCGCAGCCATCTTGGCCAGATCCTCCGCCGCCG
CCCCTGGCTCGTCCACCCCCGCCACCGCTACCGTTGGAGAAGGAAAAATGGCATCTTCAACACCCGCCTCTCCCGCACC
TTCGGATATACTGTCAAGGCTACCACAGTCAGAACGCCCTCCTGGGCGGTGGACATGATGAGATTTAATATTGACGAC
TTTGTTCCCCCGGGAGGGGGGACCAACAAAATCTCTATACCCTTTGAATACTACAGAATAAGAAAGGTTAAGGTTGAA
TTCTGGCCCTGCTCCCCCATCACCCAGGGTGATAGGGGAGTGGGCTCCACTGCTGTTATTCTAGATGATAACTTTGTAC
CAAAGGCCACAGCCCAAACCTATGACCCATATGTAAACTACTCCACCCGCCATACAATCCCCCAACCCTTCTCCTACCAC
TCCCGTTACTTCACACCCAAACCTGTTCTTGACTCCACTATTGATTACTTCCAACCAAATAACAAAAGGAATCAGCTTTG
GCTGAGGATACAAACCTCTGGAAATGTAGACCACGTAGGCCTCGGCACTGCGTTCGAAAACAGTATATACGACCAGG
ACTACAATATCCGTGTAACCATGTATGTACAATTCAGAGAATTTAATCTTAAAGACCCCCCACTTAAACCC--------

>AF085695_pcv2a

ATGACGTATCCAAGGAGGCGTTACCGCAGAAGAAGACACCGCCCCCGCAGCCATCTTGGCCAGATCCTCCGCCGCCG
CCCCTGGCTCGTCCACCCCCGCCACCGCTACCGTTGGAGAAGGAAAAATGGCATCTTCAACACCCGCCTCTCCCGCACC
TTCGGATATACTGTCAAGCGTACCACAGTCACAACGCCCTCCTGGGCGGTGGACATGATGAGATTTAAAATTGACGAC
TTTGTTCCCCCGGGAGGGGGGACCAACAAAATCTCTATACCCTTTGAATACTACAGAATAAGAAAGGTTAAGGTTGAA
TTCTGGCCTTGCTCCCCCATCACCCAGGGTGATAGGGGAGTGGGCTCCACTGCTGTTATTTTAGATGATAACTTTGTAA
CAAAGGCCACAGCCCTAACCTATGACCCATATGTAAACTACTCCTCCCGCCATACAATCCCCCAACCCTTCTCCTACCAC
TCCCGTTACTTCACACCCAAACCTGTTCTTGACTCCACTATTGATTACTTCCAACCAAATAACAAAAGGAATCAGCTTTG
GCTGAGGCTACAAACCTCTGGAAATGTGGACCACGTAGGCCTCGGCACTGCGTTCGAAAACAGTAAATACGACCAGG
ACTACAATATCCGTGTAACCATGTATGTACAATTCAGAGAATTTAATCTTAAAGACCCCCCACTTGAACCC--------

>AF086836_pcv2a

ATGACGTATCCAAGGAGGCGTTACCGCAGAAGAAGACACCGCCCCCGCAGCCATCTTGGCCAGATCCTCCGCCGCCG
CCCCTGGCTCGTCCACCCCCGCCACCGCTACCGTTGGAGAAGGAAAAATGGCATCTTCAACACCCGCCTCTCCCGCACC
TTCGGATATACTGTCAAGCGTACCACAGTCACAACGCCCTCCTGGGCGGTGGACATGATGAGATTTAAAATTGACGAC
TTTGTTCCCCCGGGAGGGGGGACCAACAAAATCTCTATACCCTTTGAATACTACAGAATAAGAAAGGTTAAGGTTGAA
TTCTGGCCTTGCTCCCCCATCACCCAGGGTGATAGGGGAGTGGGCTCCACTGCTGTTATTTTAGATGATAACTTTGTAA
CAAAGGCCACAGCCCTAACCTATGACCCATATGTAAACTACTCCTCCCGCCATACAATCCCCCAACCCTTCTCCTACCAC
TCCCGTTACTTCACACCCAAACCTGTTCTTGACTCCACTATTGATTACTTCCAACCAAATAACAAAAGGAATCAGCTTTG
GCTGAGGCTACAAACCTCTGGAAATGTGGACCACGTAGGCCTCGGCACTGCGTTCGAAAACAGTAAATACGACCAGG
ACTACAATATCCGTGTAACCATGTATGTACAATTCAGAGAATTTAATCTTAAAGACCCCCCACTTGAACCC--------

>AF086835_pcv2a

ATGACGTATCCAAGGAGGCGTTACCGCAGAAGAAGACACCGCCCCCGCAGCCATCTTGGCCAGATCCTCCGCCGCCG
CCCCTGGCTCGTCCACCCCCGCCACCGCTACCGTTGGAGAAGGAAAAATGGCATCTTCAACACCCGCCTCTCCCGCACC
TTCGGATATACTGTCAAGCGTACCACAGTCACAACGCCCTCCTGGGCGGTGGACATGATGAGATTTAAAATTGACGAC
TTTGTTCCCCCGGGAGGGGGGACCAACAAAATCTCTATACCCTTTGAATACTACAGAATAAGAAAGGTTAAGGTTGAA
TTCTGGCCCTGCTCCCCCATCACCCAGGGTGATAGGGGAGTGGGCTCCACTGCTGTTATTTTAGATGATAACTTTGTAA
CAAAGGCCACAGCCCTAACCTATGACCCATATGTAAACTACTCCTCCCGCCATACAATCCCCCAACCCTTCTCCTACCAC
TCCCGTTACTTCACACCCAAACCTGTTCTTGACTCCACTATTGATTACTTCCAACCAAATAACAAAAGGAATCAGCTTTG
GCTGAGGCTACAAACCTCTGGAAATGTGGACCACGTAGGCCTCGGCACTGCGTTCGAAAACAGTAAATACGACCAGG
ACTACAATATCCGTGTAACCATGTATGTACAATTCAGAGAATTTAATCTTAAAGACCCCCCACTTAAACCC--------

>GU049340_pcv2a

ATGACGTATCCAAGGAGGCGTTACCGCAGAAGAAGACACCGCCCCCGCAGCCATCTTGGCCAGATCCTCCGCCGCCG
CCCCTGGCTCCTCCACCCCCGCCACCGCTACCGTTGGAGAAGGAAAAATGGCATCTTCAACACCCGCCTCTCCCGCACC
TTCGGATATACTGTCAAGCGTACCACAGTCACAACGCCCTCCTGGGCGGTGGACATGATGAGATTTAAAATTGACGAC
TTTGTTCCCCCGGGAGGGGGGACCAACAAAATCTCTATACCCTTTGAATACTACAGAATAAGAAAGGTTAAGGTTGAA
TTCTGGCCCTGCTCCCCCATCACCCAGGGTGATAGGGGAGTGGGCTCCACTGCTGTTATTCTAGATGATAACTTTGTAA
CAAAGGCCACAGCCCTAACCTATGACCCATATGTAAACTACTCCTCCCGCCATACAATCCCCCAACCCTTCTCCTACCAC
TCCCGTTACTTCACACCCAAACCTGTTCTTGACTCCACTATTGATTACTTCCAACCAAATAACAAAAGGAATCAGCTTTG
GCTGAGGCTACAAACCTCTGGAAATGTGGACCACGTAGGCCTCGGCATTGCGTTCGAAAACAGTAAATACGACCAGG
ACTACAATATCCGTGTAACCATGTATGTACAATTCAGAGAATTTAATCTTAAAGACCCCCCACTT--------------

>AF055392_pcv2a

ATGACGTATCCAAGGAGGCGTTACCGCAGAAGAAGACACCGCCCCCGCAGCCATCTTGGCCAGATCCTCCGCCGCCG
CCCCTGGCTCGTCCACCCCCGCCACCGCTACCGTTGGAGAAGGAAAAATGGCATCTTCAACACCCGCCTCTCCCGCACC
TTCGGATATACTGTCAAGCGTACCACAGTCACAACGCCCTCCTGGGCGGTGGACATGATGAGATTTAAAATTGACGAC
TTTGTTCCCCCGGGAGGGGGGACCAACAAAATCTCTATACCCTTTGAATACTACAGAATAAGAAAGGTTAAGGTTGAA
TTCTGGCCCTGCTCCCCCATCACCCAGGGTGATAGGGGAGTGGGCTCCACTGCTGTTATTCTAGATGATAACTTTGTAA
CAAAGGCCACAGCCCTAACCTATGACCCATATGTAAACTACTCCTCCCGCCATACAATCCCCCAACCCTTCTCCTACCAC
TCCCGTTACTTCACACCCAAACCTGTTCTTGACTCCACTATTGATTACTTCCAACCAAATAACAAAAGGAATCAGCTTTG
GCTGAGACTACAAACCTCTGGAAATGTGGACCACGTAGGCCTCGGCGCTGCGTTCGAAAACAGTAAATACGACCAGG
ACTACAATATCCGTGTAACCATGTATGTACAATTCAGAGAATTTAATCTTAAAGACCCCCCACTTAAACCC--------

>AB361573_pcv2a

ATGACGTATCCAAGGAGGCGTTACCGCAGAAGAAGACACCGCCCCCGCAGCCATCTTGGCCAGATCCTCCGCCGCCG
CCCCTGGCTCGTCCACCCCCGCCACCGCTACCGCTGGAGAAGGAAGAATGGCATCTTCAACACCCGCCTCTCCCGCAC
CTTCGGATATACTGTCAAGCGAACCACAGTCACAACGCCCTCCTGGGCGGTGGACATGATGAGATTTAATCTTGACGA
CTTTGTTCCCCCGGGAGGGGGGACCAACAAAATCTCTATACCCTTTGAATACTACAGAATAAGAAAGGTTAAGGTTGA
ATTCTGGCCCTGCTCCCCCATCACCCAGGGTGATAGGGGAGTGGGCTCCACTGCTGTTATTCTAGATGATAACTTTGTA
CCAAAGGCCACAGCCCAAACCTATGACCCATATGTAAACTACTCCTCCCGCCATACAATCCCCCAACCCTTCTCCTACCA
CTCCCGTTACTTCACACCCAAACCTGTCCTTGACTCCACTATTGATTACTTCCAACCAAATAACAAAAGGAATCAGCTTT
GGCTGAGGCTACAAACCTCTAGAAATGTGGACCACGTAGGCCTCGGCACTGCATTCGAAAACAGTAAATACGACCAG
GACTACAATATCCGTGTAACCATGTATGTACAATTCAGAGAATTTAATCTTAAAGACCCCCCACTTAAACCC--------

>AB361572_pcv2a

ATGACGTATCCAAGGAGGCGTTACCGCAGAAGAAGACACCGCCCCCGCAGCCATCTTGGCCAGATCCTCCGCCGCCG
CCCCTGGCTCGTCCACCCCCGCCACCGCTACCGCTGGAGAAGGAAAAATGGCATCTTCAACACCCGCCTCTCCCGCAC
CTTCGGATATACTGTCAAGCGAACCACAGTCACAACGCCCTCCTGGGCGGTGGACATGATGAGATTTAATCTTGACGA
CTTTGTTCCCCCGGGAGGGGGGACCAACAAAATCTCTATACCCTTTGAATACTACAGAATAAGAAAGGTTAAGGTTGA
ATTCTGGCCCTGCTCCCCCATCACCCAGGGTGATAGGGGAGTGGGCTCCACTGCTGTTATTCTAGATGATAACTTTGTA
CCAAAGGTCACAGCCCAAACCTATGACCCATATGTAAACTACTCCTCCCGCCATACAATCCCCCAACCCTTCTCCTACCA
CTCCCGTTACTTCACACCCAAACCTGTTCTTGACTCCACTATTGATTACTTCCAACCAAATAACAAAAGGAATCAGCTTT
GGCTGAGGCTACAAACCTCTAGAAATGTGGACCACGTAGGCCTCGGCACTGCATTCGAAAACAGTAAATACGACCAG
GACTACAATATCCGTGTAACCATGTATGTACAATTCAGAGAATTTAATCTTAAAGACCCCCCACTTAAACCC--------

>HV508994_pcv2a

ATGACGTATCCAAGGAGGCGTTACCGAAGAAGAAGACACCGCCCCCGCAGCCATCTTGGCCAGATCCTCCGCCGCCG
CCCCTGGCTCGTCCACCCCCGCCACCGCTACCGTTGGAGAAGGAAAAATGGCATCTTCAACACCCGCCTCTCCCGCACC
TTCGGATATACTGTCAAGGCTACCACAGTCAGAACGCCCTCCTGGGCGGTGGACATGATGAGATTTAATCTTGACGAC
TTTGTTCCCCCGGGAGGGGGGACCAACAAAATCTCTATACCCTCTGAATACTACAGAATAAGAAAGGTTAAGGTTGAA
TCCTGGCCCTGCTCCCCCATCACCCAGGGTGATAGGGGAGTGGGCTCCACTGCTGTTATTCTAGATGATAACTTTGTAC
CAAAGGTCAATGCCCAAACCTATGACCCATATGTAAACTACTCCTCCCGCCATACAATCCCCCAACCCTTCTCCTACCAC
TCCCGTTACTTCACACCCAAACCTGTTCTTGACTCCACTATTGATTACTTCCAACCAAATAACAAAAGGAATCAGCTTTG
GCTGAGGCTACAAACCTCTAGAAATGTGGACCACGTAGGCCTCGGCACTGCGTTCGAAAACAGTATATACGACCAGG
ACTACAATATCCGTGTAACCATGTATGTACAATTCAGAGAATTTAATCTTAAAGACCCCCCACTTAAACCC--------

>FJ905471_pcv2a

ATGACGTATCCAAGGAGGCGTTACCGAAGAAGAAGACACCGCCCCCGCAGCCATCTTGGCCAGATCCTCCGCCGCCG
CCCCTGGCTCGTCCACCCCCGCCACCGCTACCGTTGGAGAAGGAAAAATGGCATCTTCAACACCCGCCTCTCCCGCACC
TTCGGATATACTGTCAAGGCTACCACAGTCAGAACGCCCTCCTGGGCGGTGGACATGATGAGATTTAATCTTGACGAC
TTTGTTCCCCCGGGAGGGGGGACCAACAAAATCTCTATACCCTCTGAATACTACAGAATAAGAAAGGTTAAGGTTGAA
TCCTGGCCCTGCTCCCCCATCACCCAGGGTGATAGGGGAGTGGGCTCCACTGCTGTTATTCTAGATGATAACTTTGTAC
CAAAGGTCAATGCCCAAACCTATGACCCATATGTAAACTACTCCTCCCGCCATACAATCCCCCAACCCTTCTCCTACCAC
TCCCGTTACTTCACACCCAAACCTGTTCTTGACTCCACTATTGATTACTTCCAACCAAATAACAAAAGGAATCAGCTTTG
GCTGAGGCTACAAACCTCTAGAAATGTGGACCACGTAGGCCTCGGCACTGCGTTCGAAAACAGTATATACGACCAGG
ACTACAATATCCGTGTAACCATGTATGTACAATTCAGAGAATTTAATCTTAAAGACCCCCCACTTAAACCC--------

>HQ395054_pcv2a

ATGACGTATCCAAGGAGGCGTTACCGAAGAAGAAGACACCGCCCCCGCAGCCATCTTGGCCAGATCCTCCGCCGCCG
CCCCTGGCTCGTCCACCCCCGCCACCGCTACCGTTGGAGAAGGAAAAATGGCATCTTCAACACCCGCCTCTCCCGCACC
TTCGGATATACTGTCAAGGCTACCACAGTCAGAACGCCCTCCTGGGCGGTGGACATGATGAGATTTAATCTTGACGAC
TTTGTTCCCCCGGGAGGGGGGACCAACAAAATCTCTATACCCTTTGAATACTACAGAATAAGAAAAGTTAAGGTTGAA
TTCTGGCCCTGCTCCCCCATCACCCAGGGTGATAGGGGAGTGGGCTCCACTGCTGTTATTCTAGATGATAACTTTGTAC
CAAAGGTCAATGCCCAAACCTATGACCCATATGTAAACTACTCCTCCCGCCATACAATCCCCCAACCCTTCTCCTACCAC
TCCCGTTACTTCACACCCAAACCTGTTCTTGACTCCACTATTGATTACTTCCAACCAAATAACAAAAGGAATCAGCTTTG
GCTGAGGCTACAAACCTCTAGAAATGTGGACCACGTAGGCCTCGGCACTGCGTTCGAAAACAGTATATACGACCAGG
ACTACAATATCCGTGTAACCATGTATGTACAATTCAGAGAATTTAATCTTAAAGACCCCCCACTTAAACCC--------

>FJ870967_pcv2a

ATGACGTATCCAAGGAGGCGTTACCGAAGAAGAAGACACCGCCCCCGCAGCCATCTTGGCCAGATCCTCCGCCGCCG
CCCCTGGCTCGTCCACCCCCGCCACCGCTACCGTTGGAGAAGGAAAAATGGCATCTTCAACACCCGCCTCTCCCGCACC
TTCGGATATACTGTCAAGGCTACCACAGTCGGAACGCCCTCCTGGGCGGTGGACATGATGAGATTTAATCTTGACGAC
TTTGTTCCCCCGGGAGGGGGGACCAACAAAATCTCTATACCCTTTGAATACTACAGAATAAGAAAAGTTAAGGTTGAA
TTCTGGCCCTGCTCCCCCATCACCCAGGGTGATAGGGGAGTGGGCTCCACTGCTGTTATTCTAGATGATAACTTTGTAC
CAAAGGTCAATGCCCAAACCTATGACCCATATGTAAACTACTCCTCCCGCCATACAATCCCCCAACCCTTCTCCTACCAC
TCCCGTTACTTCACACCCAAACCCGTTCTTGACTCCACTATTGATTACTTCCAACCAAATAACAAAAGGGATCAGCTTTG
GCTGAGGCTACAAACCTCTAGAAATGTGGACCACGTAGGCCTCGGCACTGCGTTCGAAAACAGTATATACGACCAGG
ACTACAATATCCGTGTAACCATGTATGTACAATTCAGAGAATTTAATCTTAAAGACCCCCCACTTAAACCC--------

>FJ870968_pcv2a

ATGACGTATCCAAGGAGGCGTTACCGAAGAAGAAGACACCGCCCCCGCAGCCATCTTGGCCAGATCCTCCGCCGCCG
CCCCTGGCTCGTCCACCCCCGCCACCGCTACCGTTGGAGAAGGAAAAATGGCATCTTCAACACCCGCCTCTCCCGCACC
TTCGGATATACTGTCAAGGCTACCACAGTCAGAACGCCCTCCTGGGCGGTGGACATGATGAGATTTAATCTTGACGAC
TTTGTTCCCCCGGGAGGGGGGACCAACAAAATCTCTATACCCTTTGAATACTACAGAATAAGAAAAGTTAAGGTTGAA
TTCTGGCCCTGCTCCCCCATCACCCAGGGTGATAGGGGAGTGGGCTCCACTGCTGTTATTCTAGATGATAACTTTGTAC
CAAAGGTCACAGCCCAAACCTATGACCCATATGTAAACTACTCCTCCCGCCATACAATCCCCCAACCCTTCTCCTACCAC
TCCCGTTACTTCACACCCAAACCTGTTCTTGACTCCACTATTGATTACTTCCAACCAAATAACAAAAGGAATCAGCTTTG
GCTGAGGCTACAAACCTCTAGAAATGTGGACCACGTAGGCCTCGGCACTGCGTTCGAAAACAGTATATACGACCAGG
ACTACAATATCCGTGTAACCATGTATGTACAATTCAGAGAATTTAATCTTAAAGACCCCCCACTTAAACCC--------

>JF683403_pcv2a

ATGACGTATCCAAGGAGGCGTTACCGCAGAAGAAGACACCGCCCCCGCAGCCATCTTGGCCAGATCCTCCGGCGCCG
CCCCTGGCTCGTCCACCCCCGCCACCGCTACCGTTGGAGAAGGAAAAATGGCATCTTCAACACCCGCCTCTCCCGCACC
TTCGGATATACTGTCAAGGCTACCACAGTCACAACGCCCTCCTGGGCGGTGGACATGATGAGATTTAGCCTTGACGAC
TTTGTTCCCCCGGGAGGGGGGACCAACAAAATCTCTATACCCTTTGAATACTACAGAATAAGAAAGGTTAAGGTTGAA
TTCTGGCCCTGCTCCCCCATCACCCAGGGTGATAGGGGAGTGGGCTCCACTGCTGTTATTCTAGATGATAACTTTGTAC
CAAAGGTCGCAGCCCAGACCTATGACCCATATGTAAACTACTCCTCCCGCCATACAATCCCCCAACCCTTCTCCTACCAC
TCCCGTTACTTCACACCCAAACCTGTTCTTGACTCCACTATTGATTACTTCCAACCAAATAACAAAAGGAATCAGCTTTG
GCTGAGGCTACAAACCTCTAGAAATGTGGACCACGTAGGCCTCGGAACTGCGTTCGAAAACAGTAAATACGACCAGG
ACTACAATATCCGTGTAACCATGTATGTACAATTCAGAGAATTTAATCTTAAGGACCCCCCACTTAAACCC--------

>JF683398_pcv2a

ATGACGTATCCAAGGAGGCGTTACCGCAGAAGAAGACACCGCCCCCGCAGCCATCTTGGCCAGATCCTCCGGCGCCG
CCCCTGGCTCGTCCACCCCCGCCACCGCTACCGTTGGAGAAGGAAAAATGGCATCTTCAACACCCGCCTCTCCCGCACC
TTCGGATATACTGTCAAGGCTACCACAGTCACAACGCCCTCCTGGGCGGTGGACATGATGAGATTTAGCCTTGACGAC
TTTGTTCCCCCGGGAGGGGGGACCAACAAAATCTCTATACCCTTTGAATACTACAGAATAAGAAAGGTTAAGGTTGAA
TTCTGGCCCTGCTCCCCCATCACCCAGGGTGATAGGGGAGTGGGCTCCACTGCTGTTATTCTAGATGATAACTTTGTAC
CAAAGGTCGCAGCCCACACCTATGACCCATATGTAAACTACTCCTCCCGCCATACAATCCCCCAACCCTTCTCCTACCAC
TCCCGTTACTTCACACCCAAACCTGTTCTTGACTCCACTATTGATTACTTCCAACCAAATAACAAAAGGAATCAGCTTTG
GCTGAGGCTACAAACCTCTAGAAATGTGGACCACGTAGGCCTCGGAACTGCGTTCGAAAACAGTAAATACGACCAGG
ACTACAATATCCGTGTAACCATGTATGTACAATTCAGAGAATTTAATCTTAAGGACCCCCCACTTAAACCC--------

>AY754022_pcv2a

ATGACGTATCCAAGGAGGCGTTACCGCAGAAGAAGACACCGCCCCCGCAGCCATCTTGGCCAGATCCTCCGCCGCCG
CCCCTGGCTCGTCCACCCCCGCCACCGCTACCGTTGGAGAAGGAAAAATGGCATCTTCAACACCCGCCTCTCCCGCACC
TTCGGATATACTGTCAAGGCTACCACAGTCACAACGCCCTCCTGGGCGGTGGACATGATGAGATTTAATATTGACGAC
TTTGTTCCCCCGGGAGGAGGGACAAACAAAATCTCTATACCCTTTGAATACTACAGAATAAGAAAGGTTAAGGTTGAA
TTCTGGCCCTGCTCCCCCATCACCCAGGGTGATAGAGGAGTGGGATCCACTGCTGTTATTCTAGATGATAACTTTGTAC
CAAAGGCCACAGCCCAAACCTATGACCCATATGTAAACTACTCCTCCCGCCATACAATCCCCCAACCCTTCTCCTACCAC
TCCCGTTACTTCACACCCAAACCTGTTCTTGACTCCACTATTGATTACTTCCAACCAAATAACAAAAGGAATCAGCTTTG
GCTGAGGATACAAACCTCTAGAAATGTGGACCACGTAGGCCTCGGCACTGCGTTCGAAAACAGTAAATACGACCAGG
ACTACAATATCCGTGTAACCATGTATGTACAATTCAGGGAATTTAATCTTAAAGACCCCCCACTTAAACCC--------

>AY699793_pcv2a

ATGACGTATCCAAGGAGGCGTTACCGCAGAAGAAGACACCGCCCCCGCAGCCATCTTGGCCAGATCCTCCGCCGCCG
CCCCTGGCTCGTCCACCCCCGCCACCGCTACCGTTGGAGAAGGAAAAATGGCATCTTCAACACCCGCCTCTCCCGCACC
TTCGGATATACTGTCAAGGCTACCACAGTCACAACGCCCTCCTGGGCGGTGGACATGATGAGATTTAATATTGACGAC
TTTGTTCCCCCGGGAGGGGGGACCAACAAAATCTCTATACCCTTTGAATACTACAGAATAAGAAAGGTTAAGGTTGAA
TTCTGGCCCTGCTCCCCCATCACCCAGGGTGATAGGGGAGTGGGCTCCACTGCTGTTATTCTAGATGATAACTTTGTAC
CAAAGGCCACAGCCCTAACCTATGACCCATATGTAAACTACTCCTCCCGCCATACAATCCCCCAACCCTTCTCCTACCAC
TCCCGTTACTTCACACCCAAACCTGTTCTTGACTCCACTATTGATTACTTCCAACCAAATAACAAAAGGAATCAGCTTTG
GCTGAGGCTACAAACCTCTAGAAATGTGGACCACGTAGGCCTCGGCACTGCGTTCGAAAACAGTAAATACGACCAGG
ACTACAATATCCGTGTAACCATGTATGTACAATTCAGAGAATTTAATCTTAAAGACCCCCCACTTAAACCC--------

>AY099500_pcv2a

ATGACGTATCCAAGGAGGCGTTACCGCAGAAGAAGACACCGCCCCCGCAGCCATCTTGGCCAGATCCTCCGCCGCCG
CCCCTGGCTCGTCCACCCCCGCCACCGCTACCGTTGGAGAAGGAAAAATGGCATCTTCAACACCCGGCTTTCCCGCAC
CTTCGGATATACTGTCAAGCGTACCACAGTCACAACGCCCTCCTGGGCGGTGGACATGATGAGATTTAAGCTTGACGA
CTTTGTTCCCCCGGGAGGGGGGACCAACAAAATCTCTATACCCTTTGAATACTACAGAATAAGAAAGGTTAAGGTTGA
ATTCTGGCCCTGCTCCCCCATCACCCAGGGTGATAGGGGAGTGGGCTCCACTGCTGTTATTCTAGATGATAACTTTGTA
CCAAAGGCCACAGCCCTAACCTATGACCCATATGTAAACTACTCCTCCCGCCACACAATCCCCCAACCCTTCTCCTACCA
CTCCCGTTACTTCACACCCAAACCTGTTCTTGACTCCACTATTGATTACTTCCAACCAAATAACAAAAGGAATCAGCTTT
GGCTGAGGCTACAAACCTCTAGAAATGTGGACCACGTAGGCCTCGGCACTGCGTTCGAAAACAGTAAATACGACCAG
GACTACAATATCCGTGTAACCATGTATGTACAATTCAGAGAATTTAATCTTAAAGACCCCCCACTTAAACCC--------

>AF520783_pcv2a

ATGACGTATCCAAGGAGGCGTTACCGCAGAAGAAGACACCGCCCCCGCAGCCATCTTGGCCAGATCCTCCGCCGCCG
CCCCTGGCTCGTCCACCCCCGCCACCGCTACCGTTGGAGAAGGAAAAATGGCATCTTCAACACCCGCCTCTCCCGCACC
TTCGGATATACTGTCAAACGTACCACAGTCACAACGCCCTCCTGGGCGGTGGACATGATGAGATTTACGATTGGCGAC
TTTGTTCCCCCGGGAGGGGGGACCAACAAAATCTCTATACCCTTTGAATACTACAGAATAAGAAAGGTTAAGGTTGAA
TTCTGGCCCTGCTCCCCCATCACCCAGGGTGATAGGGGAGTGGGCTCCACTGCTGTTATTCTAGATGATAACTTTGTAC
CAAAGGCCACAGCCCTAACCTATGACCCATATGTAAACTACTCCTCCCGCCATACAATCCCCCAACCCTTCTCCTACCAC
TCCCGTTACTTCACACCCAAACCTGTTCTTGACTCCACTATTGATTACTTCCAACCAAATAACAAAAGGAATCAGCTTTG
GCTGAGGCTACAAACCTCTAGAAATGTGGACCACGTAGGCCTCGGCACTGCGTTCGAAAACAGTAAATACGACCAGG
ACTACAATATCCGTGTAACCATGTATGTACAATTCAGAGAATTTAATCTTAAAGACCCCCCACTTAAACCC--------

>AF544024_pcv2a

ATGACGTATCCAAGGAGGCGTTACCGCAGAAGGAGACACCGCCCCCGCAGCCATCTTGGCCAGATCCTCCGCCGCCG
CCCCTGGCTCGTCCACCCCCGCCACCGCTACCGTTGGAGAAGGAAAAATGGCATCTTCAACACCCGCCTCTCCCGCACC
TTCGGATATACTGTCAAGCGTACCACAGTCACAACGCCCTCCTGGGCGGTGGACATGATGAGATTTACTATTGACGAC
TTTGTTCCCCCGGGAGGGGGGACCAACAAAATCTCTATACCCTTTGAATACTACAGAATAAGAAAGGTTAAGGTTGAA
TTCTGGCCCTGCTCCCCCATCACCCAGGGTGATAGGGGAGTGGGCTCCACTGCTGTTATTCTAGATGATAACTTTTTTC
CAAAGACCACAGCCCTAACCTATGACCCATATGTAAACTACTCCTCCCGCCATACAATCCCCCAACCCTTCTCCTACCAC
TCCCGTTACTTCACACCCAAACCTGTTCTTGACTCCACTATTGATTACTTCCAACCAAATAACAAAAGGAATCAGCTTTG
GCTGAGGCTACAAACCTCTAGAAATGTGGACCACGTAGGCCTCGGCACTGCGTTCGAAAACAGTAAATACGACCAGG
ACTACAATATCCGTGTAACCATGTATGTACAATTCAGAGAATTTAATCTTAAAGACCCCCCACTTAAACCC--------

>AY099496_pcv2a

ATGACGTATCCAAGGAGGCGTTACCGCAGAAGAAGACACCGCCCCCGCAGCCATCTTGGCCAGATCCTCCGCCGCCG
CCCCTGGCTCGTCCACCCCCGCCACCGCTACCGTTGGAGAAGGAAAAATGGCATCTTCAACACCCGCCTCTCCCGCACC
TTCGGATATACTGTCAAGGCTACCACAGTCACAACGCCCTCCTGGGCGGTGGACATGATGAGATTTACGATTGACGAC
TTTGTTCCCCCGGGAGGGGGGACCAACAAAATCTCTATACCCTTTGAATACTACAGAATAAGAAAGGTTAAGGTTGAA
TTCTGGCCCTGCTCCCCCATCACCCAGGGTGATAGGGGAGTGGGCTCCACTGCTGTTATTCTAGATGATAACTTTTTTC
CAAAGAGCACAGCCCTAACCTATGACCCATATGTAAACTACTCCTCCCGCCATACAATCCCCCAACCCTTCTCCTACCAC
TCCCGTTACTTCACACCCAAACCTGTTCTTGACTCCACTATTGATTACTTCCAACCAAATAACAAAAGGAATCAGCTTTG
GCTGAGGCTACAAACCTCTAGAAATGTGGACCACGTAGGCCTCGGCACTGCGTTCGAAAACAGTAAATACGACCAGG
ACTACAATATCCGTGTAACCATGTATGTACAATTCAGAGAATTTAATCTTAAAGACCCCCCACTTAAACCC--------

>HM000100_pcv2a

ATGACGTATCCAAGGAGGCGTTACCGCAGAAGAAGACACCGCCCCCGCAGCCATCTTGGCCAGATCCTCCGCCGCCG
CCCCTGGCTCGTCCACCCCCGCCACCGCTACCGTTGGAGAAGGAAAAATGGCATCTTCAACACCCGCCTCTCCCGCACC
TTCGGATATACTGTCAAGGGTACCACAGTCACAACGCCCTCCTGGGCGGTGGACATGATGAGATTTAAAATTGACGAC
TTTGTTCCCCCGGGAGGGGGGACCAACAAAATCTCTATACCCTTTGAATACTACAGAATAAGAAAGGTCAAGGTTGAA
TTCTGGCCCTGCTCCCCCATCACCCAGGGTGATAGGGGAGTGGGCTCCACTGCTGTTATTCTAGATGATAACTTTGTAC
CAAAGGCCACAGCCCTAACCTATGACCCATATGTAAACTACTCCTCCCGCCATACAATCCCCCAACCCTTCTCCTACCAC
TCCCGTTACTTCACACCCAAACCTGTTCTTGACTCCACTATTGATTACTTCCAACCAAATAACAAAAGGAATCAGCTTTG
GCTGAGGCTACAAACCTCTAGAAATGTGGACCACGTAGGCCTTGGCACTGCGTTCGAAAACAGTAAATACGACCAGG
ACTACAATATCCGTGTAACCATGTATGTACAATTCAGAGAATTTAATCTTAAAGACCCCCCACTTAACCCC--------

>DQ104423_pcv2a

ATGACGTATCCAAGGAGGCGTTACCGCAGAAGAAGACACCGCCCCCGCAGCCATCTTGGCCAGATCCTCCGCCGCCG
CCCCTGGCTCCTCCACCCCCGCCACCGCTACCGCTGGAGAAGGAAAAATGGCATCTTCAACACCCGCCTCTCCCGCACC
TTCGGATATACTGTCAAGCGTACCACGGTCACAACGCCCTCCTGGGCGGTGGACATGATGAGATTTAAACTTGACGAC
TTTGTTCCCCCGGGAGGGGGGACCAACAAAATCTCTATACCCTTTGAATACTACAGAATAAGAAAGGTTAAGGTTGAA
TTCTGGCCCTGCTCCCCCATCACCCAGGGTGATAGGGGAGTGGGCTCCACTGCTGTTATTCTAGATGATAACTTTGTAC
CAAAGGCCACAGCCCTAACCTATGACCCATATGTAAACTACTCCTCCCGCCATACAATCCCCCAACCCTTCTCCTACCAC
TCCCGTTACTTCACACCCAAACCTGTTCTTGACTCCACTATTGATTACTTCCAACCAAATAACAAAAGGAATCAGCTTTG
GCTGAGGCTACAAACCTCTAGAAATGTGGACCACGTAGGCCTCGGCACTGCGTTCGAAAACAGTAAATACGACCAGG
ACTACAATATCCGTGTAACCATGTATGTACAATTCAGAGAATTTAATCTTAAAGACCCCCCACTTAACCCC--------

>DQ104421_pcv2a

ATGACGTATCCAAGGAGGCGTTACCGCAGAAGAAGACACCGCCCCCGCAGCCATCTTGGCCAGATCCTCCGCCGCCG
CCCCTGGCTCCTCCACCCCCGCCACCGCTACCGCTGGAGAAGGAAAAATGGCATCTTCAACACCCGCCTCTCCCGCACC
TTCGGATATACTGTCAAGCGTACCACGGTCACAACGCCCTCCTGGGCGGTGGACATGATGAGATTTAAACTTGACGAC
TTTGTTCCCCCGGGAGGGGGGACCAACAAAATCTCTATACCCTTTGAATACTACAGAATAAGAAAGGTTAAGGTTGAA
TTCTGGCCCTGCTCCCCCATCACCCAGGGTGATAGGGGAGTGGGCTCCACTGCTGTTATTCTAGATGATAACTTTGTAC
CAAAGGCCACAGCCCTAACCTATGACCCATATGTAAACTACTCCTCCCGCCATACAATCCCCCAACCCTTCTCCTACCAC
TCCCGTTACTTCACACCCAAACCTGTTCTTGACTCCACTATTGATTACTTCCAACCAAATAACAAAAGGAATCAGCTTTG
GCTGAGGCTACAAACCTCTAGAAATGTGGACCACGTAGGCCTCGGCACTGCGTTCGAAAACAGTAAATACGACCAGG
ACTACAATATCCGTGTAACCATGTATGTACAATTCAGAGAATTTAATCTTAAAGACCCCCCACTTAACCCC--------

>AY325495_pcv2a

ATGACGTATCCAAGGAGGCGTTACCGCAGAAGAAGACACCGCCCCCGCAGCCATCTTGGCCAGATCCTCCGCCGCCG
CCCCTGGCTCGTCCACCCCCGCCACCGCTACCGTTGGAGAAGGAAAAATGGCATCTTCAACACCCGCCTCTCCCGCACC
TTCGGATATACTGTCAAGCGTACCACGGTCACAACGCCCTCCTGGGCGGTGGACATGATGAGATTTAAACTTGACGAC
TTTGTTCCCCCGGGAGGGGGGACCAACAAAATCTCTATACCCTTTGAATACTACAGAATAAGAAAGGTTAAGGTTGAA
TTCTGGCCCTGCTCCCCCATCACCCAGGGTGATAGGGGAGTGGGCTCCACTGCTGTTATTCTAGATGATAACTTTGTAC
CAAAGGCCACAGCCCTAACCTATGACCCATATGTAAACTACTCCTCCCGCCATACAATCCCCCAACCCTTCTCCTACCAC
TCCCGTTACTTCACACCCAAACCTGTTCTTGACTCCACTATTGATTACTTCCAACCAAATAACAAAAGGAATCAGCTTTG
GCTGAGGCTACAAACCTCTAGAAATGTGGACCACGTAGGCCTCGGCACTGCGTTCGAAAACAGTAAATACGACCAGG
ACTACAATATCCGTGTAACCATGTATGTACAATTCAGAGAATTTAATCTTAAAGACCCCCCACTTAACCCC--------

>DQ397521_pcv2a

ATGACGTATCCAAGGAGGCGTTACCGCAGAAGAAGACACCGCCCCCGCAGCCATCTTGGCCAGATCCTCCGCCGCCG
CCCCTGGCTCGTCCACCCCCGCCACCGCTACCGTTGGAGAAGGAAAAATGGCATCTTCAACACCCGCCTCTCCCGCACC
TTCGGATATACTGTCAAGCGTACCACGGTCACAACGCCCTCCTGGGCGGTGGACATGATGAGATTTAAACTTGACGAC
TTTGTTCCCCCGGGAGGGGGGACCAACAAAATCTCTATACCCTTTGAATACTACAGAATAAGAAAGGTTAAGGTTGAA
TTCTGGCCCTGCTCCCCCATCACCCAGGGTGATAGGGGAGTGGGCTCCACTGCTGTTATTCTAGATGATAACTTTGTAC
CAAAGGCACCAGCCCTAACCTATGACCCATATGTAAACTACTCCTCCCGCCATACAATCCCCCAACCCTTCTCCTACCAC
TCCCGTTACTTCACACCCAAACCTGTTCTTGACTCCACTATTGATTACTTCCAACCAAATAACAAAAGGAATCAGCTTTG
GCTGAGGCTACAAACCTCTAGAAATGTGGACCACGTAGGCCTCGGCACTGCGTTCGAAAACAGTAAATACGACCAAG
ACTACAATATCCGTGTAACCATGTATGTACAATTCAGAGAATTTAATCTTAAAGACCCCCCACTTAACCCC--------

>FJ233908_pcv2a

ATGACGTATCCAAGGAGGCGTTACCGCAGAAGAAGACACCGCCCCCGCAGCCATCTTGGCCAGATCCTCCGCCGCCG
CCCCTGGCTCGTCCACCCCCGCCACCGCTACCGTTGGAGAAGGAAAAATGGCATCTTCAACACCCGCCTCTCCCGCACC
TTCGGATATACTGTCAAGCGTACCACGGTCACAACGCCCTCCTGGGCGGTGGACATGATGAGATTTAAACTTGACGAC
TTTGTTCCCCCGGGAGGGGGGACCAACAAAATCTCTATACCCTTTGAATACTACAGAATAAGAAAGGTTAAGGTTGAA
TTCTGGCCCTGCTCCCCCATCACCCAGGGTGATAGGGGAGTGGGCTCCACTGCTATTATTCTAGATGACAACTTTGTAC
CAAAGGCCACAGCCCTAACCTATGACCCATATGTAAACTACTCCTCCCGCCATACAATCCCCCAACCCTTCTCCTACCAC
TCCCGTTACTTCACACCCAAACCTGTTCTTGACTCCACTATTGATTACTTCCAACCAAATAACAAAAGGAATCAGCTTTG
GCTGAGGCTACAAACCTCTAGAAATGTGGACCACGTAGGCCTCGGCACTGCGTTCGAAAACAGTAAATACGACCAGG
ACTACAATATCCGTGTAACCATGTATGTACAATTCAGAGAATTTAATCTTAAAGACCCCCCACTTAACCCC--------

>AF264038_pcv2a

ATGACGTATCCCAGGAGGCGTTACCGCAGAAGAAGACACCGCCCCCGCAGCCATCTTGGCCAGATCCTCCGCCGCCG
CCCCTGGCTCCTCCACCCCCGCCACCGCTACCGTTGGAGAAGGAAAAATGGCATCTTCAACACCCGCCTCTCCCGCACC
TTCGGATATACTGTCAAGCGTACCACGGTCACAACGCCCTCCTGGGCGGTGGACATGATGAGATTTAAACTTGACGAC
TTTGTTCCCCCGGGAGGGGGGACCAACAAAATCTCTATACCCTTTGAATACTACAGAATAAGAAAGGTTAAGGTTGAA
TTCTGGCCCTGCTCCCCCATCACCCAGGGTGATAGGGGAGTGGGCTCCACTGCTGTTATTCTAGATGATAACTTTGTAC
CAAAGGCCAAAGCCCTAACCTATGACCCATATGTAAACTACTCCTCCCGCCATACAATCCCCCAACCCTTCTCCTACCAC
TCCCGTTACTTCACACCCAAACCTGTTCTTGACTCCACTATTGATTACTTCCAACCAAATAACAAAAGGAATCAGCTTTG
GCTGAGGCTACAAACCTCTAGAAATGTGGACCACGTAGGCCTCGGCACTGCGTTCGAAAACAGTAAATACGACCAGG
ACTACAATATCCGTGTAACCATGTATGTACAATTCAGAGAATTTAATCTTAAAGACCCCCCACTTAACCCC--------

>DQ104419_pcv2a

ATGACGTATCCAAGGAGGCGTTACCGCAGAAGAAGACACCGCCCCCGCAGCCATCTTGGCCAGATCCTCCGCCGCCG
CCCCTGGCTCCTCCACCCCCGCCACCGCTACCGTTGGAGAAGGAAAAATGGCATCTTCAACACCCGCCTCTCCCGCACC
TTCGGATATACTGTCAAGCGTACCACGGTCACAACGCCCTCCTGGGCGGTGGACATGATGAGATTTAAACTTGACGAC
TTTGTTCCCCCGGGAGGGGGGACCAACAAAATCTCTATACCCTTTGAATACTACAGAATAAGAAAGGTTAAGGTTGAA
TTCTGGCCCTGCTCCCCCATCACCCAGGGTGATAGGGGAGTGGGCTCCACTGCTGTTATTCTAGATGATAACTTTGTAC
CAAAGGCCACAGCCCTAACCTATGACCCATATGTAAACTACTCCTCCCGCCATACAATCCCCCAACCCTTCTCCTACCAC
TCCCGTTACTTCACACCCAAACCTGTTCTTGACTCCACTATTGATTACTTCCAACCAAATAACAAAAGGAATCAGCTTTG
GCTGAGGCTACAAACCTCTAGAAATGTGGACCACGTAGGCCTCGGCACTGCGTTCGAAAACAGTAAATACGACCAGG
ACTACAATATCCGTGTAACCATGTATGTACAATTCAGAGAATTTAATCTTAAAGACCCCCCACTTAACCCC--------

>AF264039_pcv2a

ATGACGTATCCAAGGAGGCGTTACCGCAGAAGAAGACACCGCCCCCGCAGCCATCTTGGCCAGATCCTCCGCCGCCG
CCCCTGGCTCCTCCACCCCCGCCACCGCTACCGTTGGAGAAGGAAAAATGGCATCTTCAACACCCGCCTCTCCCGCACC
TTCGGATATACTGTCAAGCGTACCACGGTCACAACGCCCTCCTGGGCGGTGGACATGATGAGATTTAAACTTGACGAC
TTTGTTCCCCCGGGAGGGGGGACCAACAAAATCTCTATACCCTTTGAATACTACAGAATAAGAAAGGTTAAGGTTGAA
TTCTGGCCCTGCTCCCCCATCACCCAGGGTGATAGGGGAGTGGGCTCCACTGCTGTTATTCTAGATGATAACTTTGTAC
CAAAGGCCACAGCCCTAACCTATGACCCATATGTAAACTACTCCTCCCGCCATACAATCCCCCAACCCTTCTCCTACCAC
TCCCGTTACTTCACACCCAAACCTGTTCTTGACTCCACTATTGATTACTTCCAACCAAATAACAAAAGGAATCAGCTTTG
GCTGAGGCTACAAACCTCTAGAAATGTGGACCACGTAGGCCTCGGCACTGCGTTCGAAAACAGTAAATACGACCAGG
ACTACAATATCCGTGTAACCATGTATGTACAATTCAGAGAATTTAATCTTAAAGACCCCCCACTTAACCCC--------

>AB361574_pcv2a

ATGACGTATCCAAGGAGGCGTTACCGCAGAAGAAGACACCGCCCCCGCAGCCATCTTGGCCAGATCCTCCGCCGCCG
CCCCTGGCTCGTCCACCCCCGCCACCGCTACCGTTGGAGAAGGAAAAATGGCATCTTCAACACCCGCCTCTCCCGCACC
TTCGGATATACTGTCAAGCGTACCACAGTCACAACGCCCTCCTGGGCGGTGGACATGATGAGATTTAAGCTTGACGAC
TTTGTTCCCCCGGGAGGGGGGACCAACAAAATTTCTATACCCTTTGAATACTACAGAATAAGAAAGGTTAAGGTTGAA
TTCTGGCCCTGCTCCCCCATCACCCAGGGTGATAGGGGAGTGGGCTCCACTGCTGTTATTCTAGATGATAACTTTGTAA
CAAAGGCCAATGCCCTAACCTATGACCCATATGTAAACTACTCCTCCCGCCATACAATCCCCCAACCCTTCTCCTACCAC
TCCCGTTACTTCACACCCAAACCTGTTCTTGACTCCACTATTGATTACTTCCAACCAAATAACAAAAGGAATCAGATTTG
GCTGAGGCTACAAACCTCTAGAAATGTGGACCACGTAGGCCTCGGCACTGCGTTCGAAAACAGTAAATACGACCAGG
ACTACAATATCCGTGTAACCATGTATGTACAATTCAGAGAATTTAATCTTAAAGACCCCCCACTTAACCCC--------

>AB462385_pcv2a

ATGACGTATCCAAGGAGGCGTTACCGCAGAAGAAGACTCCGCCCCCGCAGCCATCTTGGCCAGATCCTCCGCCGCCG
CCCCTGGCTCGTCCACCCCCGCCACCGCTACCGTTGGAAAAGGAAAAATGGCATCTTCAACACCCGCCTCTCCCGCACC
TTCGGATATACTGTCAAGCGTACCACAGTCACAACGCCCTCCTGGGCGGTGGACATGATGAGATTTAAGCTTGACGAC
TTTGTTCCCCCGGGAGGGGGGACAAACAAACTCTCTATACCCTTTGAATACTACAGAATAAGAAAAGTTAAGGTTGAA
TTCTGGCCCTGCTCTCCCATCACCCAGGGTGATAGGGGAGTGGGCTCCACTGCTGTTATTCTAGATGATAACTTTGTAC
CAAAGGCCAATGCCCTAACCTATGACCCATATGTAAACTACTCCTCCCGCCATACAATCCCCCAACCCTTCTCCTACCAC
TCCCGTTACTTCACACCCAAACCTGTTCTTGACTCCACTATTGATTACTTCCAACCAAATAACAAAAGGAATCAGCTTTG
GCTGAGGCTACAAACCTCTAGAAATGTGGACCACGTAGGCCTCGGCACTGCGTTCGAAAACAGTAAATACGACCAGG
ACTACAATATCCGTGTAACCATGTATGTACAATTCAGAGAATTTAATCTTAAAGACCCCCCACTTAACCCC--------

>GU370064_pcv2a

ATGACGTACCCAAGGAGGCGTTTCCGCAGACGAAGACACCGCCCCCGCAGCCATCTTGGCCAGATCCTCCGCCGCCG
CCCCTGGCTCGTCCACCCCCGCCACCGTTACCGCTGGAGAAGGAAAAATGGCATCTTCAACGCCCGCCTCTCCCGCAC
CTTCGGATATACTGTCAAGCGTACCACAGTCACAACGCCCTCCTGGGCGGTGGACATGATGAGATTTAAGCTTGACGA
CTTTGTTCCCCCGGGAGGGGGGACCAACAAAATCTCTATACCCTTTGAATACTACAGAATAAGAAAAGTTAAGGTTGA
ATTCTGGCCCTGCTCCCCCATCACCCAGGGTGATAGGGGAGTGGGCTCCACTGCTGTTATTCTAGATGATAACTTTGTA
CCAAAGGCCAATGCCCTAACCTATGACCCATATGTAAACTACTCCTTCCGCCATACAATCCCCCAACCCTTCTCCTACCA
CTCCCGTTACTTCACACCCAAACCTGTTCTTGACTCCACTATTGATTACTTCCAACCAAATAACAAAAGGAATCAGCTTT
GGATGAGGATACAAACCTCTAGAAATGTGGACCACGTAGGCCTCGGCACTGCGTTCGAAAACAGTAAATACGACCAG
GACTACAATATCCGTGTAACCATGTATGTACAATTCAGAGAATTTAATCTTAAAGACCCCCCACTTAAACCC--------

>JQ994269_pcv2a

ATGACGTATCCAAGGAGGCGTTACCGCAGAAGAAGACACCGCCCCCGCAGCCATCTTGGCCAGATCCTCCGCCGCCG
CCCCTGGCTCGTCCACCCCCGCCACCGCTACCGTTGGAGAAGGAAAAATGGCATCTTCAACACCCGCCTCTCCCGCACC
TTCGGATATACTGTCAAGCGTACCACAGTCACAACGCCCTCCTGGGCGGTGGACATGATGAGATTTAAGCTTGACGAC
TTTGTTCCCCCGGGAGGGGGGACCAACAAAATCTCTATACCCTTTGAATACTACAGAATAAGAAAAGTAAAGGTTGAA
TTCTGGCCCTGCTCCCCCATCACCCAGGGTGATAGGGGAGTGGGCTCCACTGCTGTTATTCTAGATGATAACTTTGTAC
CAAAGGCCAATGCCCTAACCTATGACCCATATGTAAACTACTCCTCCCGCCATACAATCCCCCAACCCTTCTCCTACCAC
TCCCGTTACTTCACACCCAAACCTGTTCTTGACTCCACTATTGATTACTTCCAACCCAATAACAAAAGGAATCAGCTTTG
GCTGAGGCTACAAACCTCTAGAAATGTGGACCACGTAGGCCTCGGCACTGCGTTCGAAAACAGTATATACGACCAGG
ACTACAATATCCGTGTAACCATGTATGTACAATTCAGAGAATTTAATCTTAAAGACCCCCCACTTAAACCC--------

>DQ629114_pcv2a

ATGACGTATCCAAGGAGGCGTTACCGCAGAAGAAGACACCGCCCCCGCAGCCATCTTGGCCAAATCCTCCGCCGCCG
CCCCTGGCTCGTCCACCCCGGCCACCGCTACCGTTGGAGAAGGAAAACTGGCATCTTCAACACCCGCCTCTCCCGCAC
CTTCGGATATACTGTCAAGCGTACCACAGTCACAACGCCCTCCTGGGCGGTGGACATGATGAGATTTAAGCTTGACGA
CTTTGTTCCCCCGGGAGGGGGGACCAACAAAATCTCTATACCCTTTGAATACTACAGAATAAAAAAAGTTAAGGTTGA
ATTCTGGCCCTGCTCCCCCATCACCCAGGGTGATAGGGGAGTGGGCTCCACTGCTGTTATTCTAGATGATAACTTTGTA
ACAAAGGCCACTGCCCTAACCTATGACCCATATGTAAACTACTCCTCCCGCCATACAATCCCCCAACCCTTCTCCTACCA
CTCCCGTTACTTCACACCCAAACCTGTTCTTGACTCCACTATTGATTACTTCCAACCAAATAACAAAAGGAATCAGCTTT
GGCTGAGGCTACAAACCTCTAGAAATGTGGACCACGTAGGCCTCGGCACTGCGTTCGAAAACAGTAAATACGACCAG
GACTACAATATCCGTGTAACCATGTATGTACAATTCAGAGAATTTAATCTTAAAGACCCCCCACTTAACCCC--------

>DQ629113_pcv2a

ATGACGTATCCAAGGAGGCGTTACCGCAGAAGAAGACACCGCCCCCGCAGCCATCTTGGCCAAATCCTCCGCCGCCG
CCCCTGGCTCGTCCACCCCGGCCACCGCTACCGTTGGAGAAGGAAAACTGGCATCTTCAACACCCGCCTCTCCCGCAC
CTTCGGATATACTGTCAAGCGTACCACAGTCACAACGCCCTCCTGGGCGGTGGACATGATGAGATTTAAGCTTGACGA
CTTTGTTCCCCCGGGAGGGGGGACCAACAAAATCTCTATACCCTTTGAATACTACAGAATAAGAAAAGTTAAGGTTGA
ATTCTGGCCCTGCTCCCCCATCACCCAGGGTGATAGGGGAGTGGGCTCCACTGCTGTTATTCTAGATGATAACTTTGTA
ACAAAGGCCACTGCCCTAACCTATGACCCATATGTAAACTACTCCTCCCGCCATACAATCCCCCAACCCTTCTCCTACCA
CTCCCGTTACTTCACACCCAAACCTGTTCTTGACTCCACTATTGATTACTTCCAACCAAATAACAAAAGGAATCAGCTTT
GGCTGAGGCTACAAACCTCTAGAAATGTGGACCACGTAGGCCTCGGCACTGCGTTCGAAAACAGTAAATACGACCAG
GACTACAATATCCGTGTAACCATGTATGTACAATTCAGAGAATTTAATCTTAAAGACCCCCCACTTAACCCC--------

>FJ218001_pcv2a

ATGACGTATCCAAGGAGGCGTTACCGCAGAAGAAGACACCGCCCCCGCAGCCATCTTGGCCAAATCCTCCGCCGCCG
CCCCTGGCTCGTCCACCCCGGCCACCGCTACCGTTGGAGAAGGAAAACTGGCATCTTCAACACCCGCCTCTCCCGCAC
CTTCGGATATACTGTCAAGCGTACCACAGTCACAACGCCCTCCTGGGCGGTGGACATGATGAGATTTAAGCTTGACGA
CTTTGTTCCCCCGGGAGGGGGGACCAACAAAATCTCTATACCCTTTGAATACTACAGAATAAGAAAAGTTAAGGTTGA
ATTCTGGCCCTGCTCCCCCATCACCCAGGGTGATAGGGGAGTGGGCTCCACTGCTGTTATTCTAGATGATAACTTTGTA
ACAAAGGCCACTGCCCTAACCTATGACCCATATGTAAACTACTCCTCCCGCCATACAATCCCCCAACCCTTCTCCTACCA
CTCCCGTTACTTCACACCCAAACCTGTTCTTGACTCCACTATTGATTACTTCCAACCAAATAACAAAAGGAATCAGCTTT
GGCTGAGGCTACAAACCTCTAGAAATGTGGACCACGTAGGCCTCGGCACTGCGTTCGAAAACAGTAAATACGACCAG
GACTACAATATCCGTGTAACCATGTATGTACAATTCAGAGAATTTAATCTTAAAGACCCCCCACTTAACCCC--------

>AY094619_pcv2a

ATGACGTATCCAAGGAGGCGTTACCGCAGAAGAAGACACCGCCCCCGCAGCCATCTTGGCCAGATCCTCCGCCGCCG
CCCCTGGCTCGTCCACCCCCGCCACCGCTACCGTTGGAGAAGGAAAAATGGCATCTTCAACACCCGCCTCTCCCGCACC
TTCGGATATACTGTCAAGCGTACCACAGTCACAACGCCCTCCTGGGCGGTGGACATGATGAGATTTAAGTTTGACGAC
TTTGTTCCCCCGGGAGGGGGGACCAACAAAATCTCTATACCCTTTGAATACTACAGAATAAGAAAAGTTAAGGTTGAA
TTCTGGCCCTGCTCCCCCATCACCCAGGGTGATAGGGGAGTGGGCTCCACTGCTGTTATTCTAGATGATAACTTTGTAC
CAAAGGCCAATGCCCTAACCTATGACCCATATGTAAACTACTCCTCCCGCCATACAATCCCCCAACCCTTCTCCTACCAC
TCCCGTTACTTCACACCCAAACCTGTTCTTGACTCCACTATTGATTACTTCCAACCAAATAACAAAAGGAATCAGCTTTG
GCTGAGGCTACAGACCTCTAGAAATGTGGACCACGTAGGCCTCGGCACTGCGTTCGAAAACAGTAAATACGACCAGG
ACTACAATATCCGTATAACCATGTATGTACAATTCAGAGAATTTAATCTTAAAGACCCCCCACTTAACCCC--------

>AB361568_pcv2a

ATGACGTATCCAAGGAGGCGTTACCGCAGAAGAAGACAACGCCCCCGCAGCCATCTTGGCCAGATCCTCCGCCGCCG
CCCCTGGCTCGTCCACCCCCGCCACCGCTACCGTTGGAGAAGGAAAAATGGCATCTTCAACACCCGCCTCTCCCGCACC
TTCGGATATACTGTCAAGCGTACCACAGTCACAACGCCCTCCTGGGCGGTGGACATGATGAGATTTAAGCTTGACGAC
TTTGTTCCCCCGGGAGGGGGGACCAACAAAATCTCTATACCCTTTGAATACTACAGAATAAGAAAAGTTAAGGTTGAA
TTCTGGCCCTGCTCCCCCATCACCCAGGGTGATAGGGGAGTGGGCTCCACTGCTGTCATTCTAGATGATAACTTTGTAC
CAAAGGCCACTGCCCTAACCTATGACCCATATGTAAACTACTCCTCCCGCCATACAATCCCCCAACCCTTCTCCTACCAC
TCCCGTTACTTCACACCCAAACCTGTTCTTGACTCCACTATTGATTACTTCCAACCAAATAACAAAAGGAATCAGCTTTG
GCTGAGGCTACAAACCTCTAGAAATGTGGACCACGTAGGCCTCGGCACTGCGTTCGAAAACAGTAAATACGACCAGG
ACTACAATATCCGTGTAACCATGTATGTACAATTCAGAGAATTTAATCTTAAAGACCCCCCACTTAACCCC--------

>FR823451_pcv2a

ATGACGTATCCAAGGAGGCGTTACCGCAGAAGAAGACAACGCCCCCGCAGCCATCTTGGCCAGATCCTCCGCCGCCG
CCCCTGGCTCGTCCACCCCCGCCACCGCTACCGTTGGAGAAGGAAAAATGGCATCTTCAACACCCGCCTCTCCCGCACC
TTCGGATATACTGTCAAGCGTACCACAGTCACAACGCCCTCCTGGGCGGTGGACATGATGAGATTTAAGCTGGACGAC
TTTGTTCCCCCGGGAGGGGGGACCAACAAAATCTCTATACCCTTTGAATACTACAGAATAAGAAAAGTTAAGGTTGAA
TTCTGGCCCTGCTCCCCCATCACCCAAGGTGATAGGGGAGTGGGCTCCACTGCTGTTATTCTAGATGATAACTTTGTAC
CAAAGGCCACTGCCCTAACCTATGACCCATATGTAAACTACTCCTCCCGCCATACAATCCCCCAACCCTTCTCCTACCAC
TCCCGTTACTTCACACCCAAACCTGTTCTTGACTCCACTATTGATTACTTCCAACCAAATAACAAAAGGAATCAGCTTTG
GCTGAGGCTACAAACCTCTAGAAATGTGGACCACGTAGGCCTCGGCACTGCGTTCGAAAACAGTAAATACGACCAGG
ACTACAATATCCGTGTAACCATGTATGTACAATTCAGAGAATTTAATCTTAAAGACCCCCCACTTAACCCC--------

>EF394776_pcv2a

ATGACGTATCCAAGGAGGCGTTACCGCAGAAGAAGAGACCGCCCCCGCAGCCATCTTGGCCAGATCCTCCGCCGCCG
CCCCTGGCTCGTCCACCCCCGCCACCGCTACCGTTGGAGAAGGAAAAATGGCATCTTCAACACCCGCCTCTCCCGCACC
TTCGGATATACTGTCAAGCGTACCACAGTCACAACGCCCTCCTGGGCGGTGGACATGATGAGATTTAAGCTTGACGAC
TTTGTTCCCCCGGGAGGGGGGACCAACAAAATCTCTATACCCTTTGAATACTACAGAATAAGAAAAGTTAAGGTTGAA
TTCTGGCCCTGCTCCCCCATCACCCAGGGTGATAGGGGAGTGGGCTCCACTGCTGTTATTCTAGATGATAACTTTGTAC
CAAAGGCCACTGCCCAAACCTATGACCCATATGTAAACTACTCCTCCCGCCATACAATCCCCCAACCCTTCTCCTACCAC
TCCCGTTACTTCACACCCAAACCTGTTCTTGACTCCACTATTGATTACTTCCAACCAAATAACAAAAGGAATCAGCTTTG
GCTGAGGCTACAAACCTCTAGAAATGTGGACCACGTAGGCCTCGGCACTGCGTTCGAAAACAGTAAATACGACCAGG
ACTACAATATCCGTGTAACCCTGTATGTACAATTCAGAGAATTTAATCTTAAAGACCCCCCACTTAACCCC--------

>AY099497_pcv2a

ATGACGTATCCAAGGAGGCGTTACCGCAGAAGAAGACACCGCCCCCGCAGCCATCTTGGCCAGATCCTCCGCCGCCG
CCCCTGGCTCGTCCACCCCCGCCAC------
CGTTGGAGAAGGAAAAATGGCATCTTCAACACCCGCCTCTCCCGCACCTTCGGATATACTGTCAAGCGTACCACAGTC
ACAACGCCCTCGTGGGCGGTGGACATGATGAGATTTAAGCTTGACGACTTTGTTCCCCCGGGAGGGGGGACCAACAA
AATCTCTATACCCTTTGAATACTACAGAATAAGAAAAGTTAAGGTTGAATTCTGGCCCTGCTCCCCCATCACCCAGGGT
GATAGGGGAGTGGGCTCCACTGCTGTTATTCTAGATGATAACTTTGTACCAAAGGCCAATGCCCTAACCTATGACCCA
TATGTAAACTACTCCTCCCGCCATACAATCCCCCAACCCTTCTCCTACCACTCCCGTTACTTCACACCCAAACCTGTTCTT
GACTCCACTATTGATTACTTCCAACCAAATAACAAAAGGAATCAGCTTTGGCTGAGGCTACAAACCTCTAGAAATGTG
GACCACGTAGGCCTCGGCACTGCGTTCGAAAACAGTAAATACGACCAGGACTACAATATCCGTGTAACCATGTATGTA
CAATTCAGAGAATTTAATCTTAAAGACCCCCCACTTAACCCC--------

>AB361583_pcv2a

ATGACGTATCCAAGGAGGCGTTACCGCAGAAGAAGACACCGCCCCCGCAGCCATCTTGGCCAGATCCTCCGCCGCCG
CCCCTGGCTCGTCCACCCCCGCCACCGCTACCGTTGGAGAAGGAAAAATGGCATCTTCAACACCCGCCTCTCCCGCACC
TTCGGATATACTGTCAAGCGTACCACAGTCACAACGCCCTCCTGGGCGGTGGACATGATGAGATTTAAGCTTGACGAC
TTTGTTCCCCCGGGAGGGGGGACCAACAAAATCTCTATACCCTTTGAATACTACAGAATAAGAAAAGTTAAGGTTGAA
TTCTGGCCCTGCTCCCCCATCACCCAGGGTGATAGGGGAGTGGGCTCCACTGCTGTTATTCTAGATGATAACTTTGTAC
CAAAGGCCAATGCCCTGACCTATGACCCATATGTAAACTACTCCTCCCGCCATACAATCCCCCAACCCTTCTCCTACCAC
TCCCGTTACTTCACACCCAAACCTGTTCTTGACTCCACTATTGATTACTTCCAACCAAATAACAAAAGGAATCAGCTTTG
GCTGAGGCTACAAACCTCTAGAAATGTGGACCACGTAGGCCTCGGCACTGCATTCGAAAACAGTAAATACGACCAGG
ACTACAATATCCGTGTAACCATGTATGTACAATTCAGAGAATTTAATCTTAAAGACCCCCCACTTAACCCC--------

>AB361582_pcv2a

ATGACGTATCCAAGGAGGCGTTACCGCAGAAGAAGACACCGCCCCCGCAGCCATCTTGGCCAGATCCTCCGCCGCCG
CCCCTGGCTCGTCCACCCCCGCCACCGCTACCGTTGGAGAAGGAAAAATGGCATCTTCAACACCCGCCTCTCCCGCACC
TTCGGATATACTGTCAAGCGTACCACAGTCACAACGCCCTCCTGGGCGGTGGACATGATGAGATTTAAGCTTGACGAC
TTTGTTCCCCCGGGAGGGGGGACCAACAAAATCTCTATACCCTTTGAATACTACAGAATAAGAAAAGTTAAGGTTGAA
TTCTGGCCCTGCTCCCCCATCACCCAGGGTGATAGGGGAGTGGGCTCCACTGCTGTTATTCTAGATGATAACTTTGTAC
AAAAGGCCAATGCCCTGACCTATGACCCATATGTAAACTACTCCTCCCGCCATACAATCCCCCAACCCTTCTCCTACCAC
TCCCGTTACTTCACACCCAAACCTGTTCTTGACTCCACTATTGATTACTTCCAACCAAATAACAAAAGGAATCAGCTTTG
GCTGAGGCTACAAACCTCTAGAAATGTGGACCACGTAGGCCTCGGCACTGCATTCGAAAACAGTAAATACGACCAGG
ACTACAATATCCGTGTAACCATGTATGTACAATTCAGAGAATTTAATCTTAAAGACCCCCCACTTAACCCC--------

>AY099499_pcv2a

ATGACGTATCCAAGGAGGCGTTACCGCAGAAGAAGACACCGCCCCCGCAGCCATCTTGGCCAGATCCTCCGCCGCCG
CCCCTGGCTCGTCCACCCCCGCCACCGCTACCGTTGGAGAAGGAAAAATGGCATCTTCAACACCCGCCTCTCCCGCACC
TTCGGATATACTGTCAAGCGTACCACAGTCACAACGCCCTCCTGGGCGGTGGACATGATGAGATTTAAGCTTGACGAC
TTTGTTCCCCCGGGAGGGGGGACCAACAAAATCTCTATACCCTTTGAATACTACAGAATAAGAAAAGTTAAGGTTGAA
TTCTGGCCCTGCTCCCCCATCACCCAGGGTGATAGGGGAGTGGGCTCCACTGCTGTTATTCTAGATGATAACTTTGTAC
CAAAGGCCAATGCCCTAACCTATGACCCATATGTAAACTACTCCTCCCGCCATACAATCCCCCAACCCTTCTCCTACCAC
TCCCGTTACTTCACACCCAAACCTGTTCTTGACTCCACTATTGATTACTTCCAACCAAATAACAAAAGGAATCAGCTTTG
GCTGAGGCTACAAACCTCTAGAAATGTGGACCACGTAGGCCTCGGCACTGCGTTCGAAAACAGTAAATACGACCAGG
ACTACAATATCCGTGTAACCATGTATGTACAATTCAGAGAATTTAATCTTAAAGACCCCCCACTTAACCCC--------

>AB361571_pcv2a

ATGACGTATCCAAGGAGGCGTTACCGCAGAAGAAGACACCGCCCCCGCAGCCATCTTGGCCAGATCCTCCGCCGCCG
CCCCTGGCTCGTCCACCCCCGCCACCGCTACCGTTGGAGAAGGAAAAATGGCATCTTCAACACCCGCCTCTCCCGCACC
TTCGGATATACTGTCAAGCGTACCACAGTCACAACGCCCTCCTGGGCGGTGGACATGATGAGATTTAAGCTTGACGAC
TTTGTTCCCCCGGGAGGGGGGACCAACAAAATCTCTATACCCTTTGAATACTACAGAATAAGAAAAGTTAAGGTTGAA
TTCTGGCCCTGCTCCCCCATCACCCAGGGTGATAGGGGAGTGGGCTCCACTGCTGTTATTCTAGATGATAACTTTGTAC
CAAAGGCCAATGCCCTAACCTATGACCCATATGTAAACTACTCCTCCCGCCATACAATCCCCCAACCCTTCTCCTACCAC
TCCCGTTACTTCACACCCAAACCTGTTCTTGACTCCACTATTGATTACTTCCAACCAAATAACAAAAGGAATCAGCTTTG
GCTGAGGCTACAAACCTCTAGAAATGTGGACCACGTAGGCCTCGGCACTGCGTTCGAAAACAGTAAATACGACCAGG
ACTACAATATCCGTGTAACCATGTATGTACAATTCAGAGAATTTAATCTTAAAGACCCCCCACTTAACCCC--------

>AB462383_pcv2a

ATGACGTATCCAAGGAGGCGTTACCGCAGAAGAAGACACCGCCCCCGCAGCCATCTTGGCCAGATCCTCCGCCGCCG
CCCCTGGCTCGTCCACCCCCGCCACCGCTACCGTTGGAGAAGGAAAAATGGCATCTTCAACACCCGCCTCTCCCGCACC
TTCGGATATACTGTCAAGCGTACCACAGTCACAACGCCCTCCTGGGCGGTGGACATGATGAGATTTAAGCTTGACGAC
TTTGTTCCCCCGGGAGGGGGGACCAACAAAATCTCTATACCCTTTGAATACTACAGAATAAGAAAAGTTAAGGTTGAA
TTCTGGCCCTGCTCCCCCATCACCCAGGGTGATAGGGGAGTGGGCTCCACTGCTGTTATTCTAGATGATAACTTTGTAC
CAAAGGCCAATGCCCTAACCTATGACCCATATGTAAACTACTCCTCCCGCCATACAATCCCCCAACCCTTCTCCTACCAC
TCCCGTTACTTCACACCCAAACCTGTTCTTGACTCCACTATTGATTACTTCCAACCAAATAACAAAAGGAATCAGCTTTG
GCTGAGGCTACAAACCTCTAGAAATGTGGACCACGTAGGCCTCGGCACTGCGTTCGAAAACAGTAAATACGACCAGG
ACTACAATATCCGTGTAACCATGTATGTACAATTCAGAGAATTTAATCTTAAAGACCCCCCACTTAACCCC--------

>AF118097_pcv2a

ATGACGTATCCAAGGAGGCGTTACCGCAGAAGAAGACACCGCCCCCGCAGCCATCTTGGCCAGATCCTCCGCCGCCG

CCCCTGGCTCGTCCACCCCCGCCACCGCTACCGTTGGAGAAGGAAAAATGGCATCTTCAACACCCGCCTCTCCCGCACC

TTCGGATATACTGTCAAGCGTACCACAGTCACAACGCCCTCCTGGGCGGTGGACATGATGAGATTTAAGCTTGACGAC

TTTGTTCCCCCGGGAGGGGGGACCAACAAAATCTCTATACCCTTTGAATACTACAGAATAAGAAAAGTTAAGGTTGAA

TTCTGGCCCTGCTCCCCCATCACCCAGGGTGATAGGGGAGTGGGCTCCACTGCTGTTATTCTAGATGATAACTTTGTAC

CAAAGGCCAATGCCCTAACCTATGACCCATATGTAAACTACTCCTCCCGCCATACAATCCCCCAACCCTTCTCCTACCAC

TCCCGTTACTTCACACCCAAACCTGTTCTTGACTCCACTATTGATTACTTCCAACCAAATAACAAAAGGAATCAGCTTTG

GCTGAGGCTACAAACCTCTAGAAATGTGGACCACGTAGGCCTCGGCACTGCGTTCGAAAACAGTAAATACGACCAGG

ACTACAATATCCGTGTAACCATGTATGTACAATTCAGAGAATTTAATCTTAAAGACCCCCCACTTAACCCC--------

>AB361570_pcv2a

ATGACGTATCCAAGGAGGCGTTACCGCAGAAGAAGACACCGCCCCCGCAGCCATCTTGGCCAGATCCTCCGCCGCCG

CCCCTGGCTCGTCCACCCCCGCCACCGCTACCGTTGGAGAAGGAAAAATGGCATCTTCAACACCCGCCTCTCCCGCACC

TTCGGATATACTGTCAAGCGTACCACAGTCACAACGCCCTCCTGGGCGGTGGACATGATGAGATTTAAGCTTGACGAC

TTTGTTCCCCCGGGAGGGGGGACCAACAAAATCTCTATACCCTTTGAATACTACAGAATAAGAAAAGTTAAGGTTGAA

TTCTGGCCCTGCTCCCCCATCACCCAGGGTGATAGGGGAGTGGGCTCCACTGCTGTTATTCTAGATGATAACTTTGTAC

CAAAGGCCAATGCCCTAACCTATGACCCATATGTAAACTACTCCTCCCGCCATACAATCCCCCAACCCTTCTCCTACCAC

TCCCGTTACTTCACACCCAAACCTGTTCTTGACTCCACTATTGATTACTTCCAACCAAATAACAAAAGGAATCAGCTTTG

GCTGAGGCTACAAACCTCTAGAAATGTGGACCACGTAGGCCTCGGCACTGCGTTCGAAAACAGTAAATACGACCAGG

ACTACAATATCCGTGTAACCATGTATGTACAATTCAGAGAATTTAATCTTAAAGACCCCCCACTTAACCCC--------

>AY099495_pcv2a

ATGACGTATCCAAGGAGGCGTTACCGCAGAAGAAGACACCGCCCCCGCAGCCATCTTGGCCAGATCCTCCGCCGCCG

CCCCTGGCTCGTCCACCCCCGCCACCGCTACCGTTGGAGAAGGAAAAATGGCATCTTCAACACCCGCCTCTCCCGCACC

TTCGGATATACTGTCAAGCGTACCACAGTCACAACGCCCTCCTGGGCGGTGGACATGATGAGATTTAAGCTTGACGAC

TTTGTTCCCCCGGGAGGGGGGACCAACAAAATCTCTATACCCTTTGAATACTACAGAATAAGAAAAGTTAAGGTTGAA

TTCTGGCCCTGCTCCCCCATCACCCAGGGTGATAGGGGAGTGGGCTCCACTGCTGTTATTCTAGATGATAACTTTGTAC

CAAAGGCCAATGCCCTAACCTATGACCCATATGTAAACTACTCCTCCCGCCATACAATCCCCCAACCCTTCTCCTACCAC

TCCCGTTACTTCACACCCAAACCTGTTCTTGACTCCACTATTGATTACTTCCAACCAAATAACAAAAGGAATCAGCTTTG

GCTGAGGCTACAAACCTCTAGAAATGTGGACCACGTAGGCCTCGGCACTGCGTTCGAAAACAGTAAATACGACCAGG

ACTACAATATCCGTGTAACCATGTATGTACAATTCAGAGAATTTAATCTTAAAGACCCCCCACTTAACCCC--------

>EF394775_pcv2a

ATGACGTATCCAAGGAGGCGTTACCGCAGAAGAAGACACCGCCCCCGCAGCCATCTTGGCCAGATCCTCCGCCGCCG

CCCCTGGCTCGTCCACCCCCGCCACCGCTACCGTTGGAGAAGGAAAAATGGCATCTTCAACACCCGCCTCTCCCGCACC

TTCGGATATACTGTCAAGCGTACCACAGTCACAACGCCCTCCTGGGCGGTGGACATGATGAGATTTAAGCTTGACGAC

TTTGTTCCCCCGGGAGGGGGGACCAACAAAATCTCTATACCCTTTGAATACTACAGAATAAGAAAAGTTAAGGTTGAA

TTCTGGCCCTGCTCCCCCATCACCCAGGGTGATAGGGGAGTGGGTTCCACTGCTGTTATTCTAGATGATAACTTTGTAC

CAAAGGCCAATGCCCTAACCTATGACCCATATGTAAACTACTCCTCCCGCCATACAATCCCCCAACCCTTCTCCTACCAC

TCCCGTTACTTCACACCCAAACCTGTTCTTGACTCCACTATTGATTACTTCCAACCAAATAACAAAAGGAATCAGCTTTG

GCTGAGGCTACAAACCTCTAGAAATGTGGACCACGTAGGCCTCGGCACTGCGTTCGAAAACAGTAAATACGACCAGG

ACTACAATATCCGTGTAACCATGTATGTACAATTCAGAGAATTTAATCTTAAAGACCCCCCACTTAACCCC--------

>AF264041_pcv2a

ATGACGTATCCAAGGAGGCGTTACCGCAGAAGAAGACACCGCCCCCGCAGCCATCTTGGCCAGATCCTCCGCCGCCG
CCCCTGGCTCGTCCACCCCCGCCACCGCTACCGTTGGAGAAGGAAAAATGGCATCTTCAACACCCGCCTCTCCCGCACC
TTCGGATATACTGTCAAGCGTACCACAGTCACAACGCCCTCCTGGGCGGTGGACATGATGAGATTTAAGCTTGACGAC
TTTGTTCCCCCGGGAGGGGGGACCAACAAAATCTCTATACCCTTTGAATACTACAGAATAAGAAAAGTTAAGGTTGAA
TTCTGGCCCTGCTCCCCCATCACCCAGGGTGATAGGGGAGTGGGCTCCACTGCTGTTATTCTAGATGATAACTTTATAC
CAAAGGCCAATGCCCTAACCTATGACCCATATGTAAACTACTCCTCCCGCCATACAATCCCCCAACCCTTCTCCTACCAC
TCCCGTTACTTCACACCCAAACCTGTTCTTGACTCCACTATTGATTACTTCCAACCAAATAACAAAAGGAATCAGCTTTG
GCTGAGGCTACAAACCTCTAGAAATGTGGACCACGTAGGCCTCGGCACTGCGTTCGAAAACAGTAAATACGACCAGG
ACTACAATATCCGTGTAACCATGTATGTACAATTCAGAGAATTTAATCTTAAAGACCCCCCACTTAACCCC--------

>AF454546_pcv2a

ATGACGTATCCAAGGAGGCGTTACCGCAGAAGAAGACACCGCCCCCGCAGCCATCTTGGCCAGATCCTCCGCCGCCG
CCCCTGGCTCGTCCACCCCCGCCACCGCTACCGTTGGAGAAGGAAAAATGGCATCTTCAACACCCGCCTCTCCCGCACC
TTCGGATATACTGTCAGGCGTACCACAGTCACAACGCCCTCCTGGGCGGTGGACATGATGAGATTTAAGCTTGACGAC
TTTGTTCCCCCGGGAGGGGGGACCAACAAAATCTCTATACCCTTTGAATACTACAGAATAAGAAAAGTTAAGGTTGAA
TTCTGGCCCTGCTCCCCCATCACCCAGGGTGATAGGGGAGTGGGCTCCACTGCTGTTATTCTAGATGATAACTTTGTAC
CAAAGGCCAATGCCCTAACCTATGACCCATATGTAAACTACTCCTCCCGCCATACAATCCCCCAACCCTTCTCCTACCAC
TCCCGTTACTTCACACCCAAACCTGTTCTTGACTCCACTATTGATTACTTCCAACCAAATAACAAAAGGAATCAGCTTTG
GCTGAGGCTACAAACCTCTAGAAATGTGGACCACGTAGGCCTCGGCACTGCGTTCGAAAACAGTAAATACGACCAGG
ACTACAATATCCGTGTAACCATGTATGTACAATTCAGAGAATTTAATCTTAAAGACCCCCCACTTAACCCC--------

>AY672601_pcv2a

ATGACGTATCCAAGGAGGCGTTACCGCAGAAGAAGACACCGCCCCCGCAGCCATCTTGGCCAGATCCTCCGCCGCCG
CCCCTGGCTCGTCCACCCCCGCCACCGCTACCGTTGGAGAAGGAAAAATGGCATCTTCAACACCCGCCTCTCCCGCACC
TTCGGATATACTGTCAAGCGTACCACAGTCACAACGCCCTCCTGGGCGGTGGACATGATGAGATTTAAGCTTGACGAC
TTTGTTCCCCCGGGTGGGGGGACCAACAAAATCTCTATACCCTTTGAATACTACAGAATAAGAAAAGTTAAGGTTGAA
TTCTGGCCCTGCTCCCCCATCACCCAGGGTGATAGGGGAGTGGGCTCCACTGCTGTTATTCTAGATGATAACTTTGTAC
CAAAGGCCAATGCCCTAACCTATGACCCATATGTAAACTACTCCTCCCGCCATACAATCCCCCAACCCTTCTCCTACCAC
TCCCGTTACTTCACACCCAAACCTGTTCTTGACTCCACTATTGATTACTTCCAACCAAATAACAAAAGGAATCAGCTTTG
GCTGAGGCTACAAACCTCTAGAAATGTGGACCACGTAGGCCTCGGCACTGCGTTCGAAAACAGTAAATACGACCAGG
ACTACAATATCCGTGTAACCATGTATGTACAATTCAGAAAATTTAATCTTAAAGACCCCCCACTTAAACCC--------

>AY672600_pcv2a

ATGACGTATCCAAGGAGGCGTTACCGCAGAAGAAGACACCGCCCCCGCAGCCATCTTGGCCAGATCCTCCGCCGCCG
CCCCTGGCTCGTCCACCCCCGCCACCGCTACCGTTGGAGAAGGAAAAATGGCATCTTCAACACCCGCCTCTCCCGCACC
TTCGGATATACTGTCAAGCGTACCACAGTCACAACGCCCTCCTGGGCGGTGGACATGATGAGATTTAAGCTTGACGAC
TTTGTTCCCCCGGGAGGGGGGACCAACAAAATCTCTATACCCTTTGAATACTACAGAATAAGAAAAGTTAAGGTTGAA
TTCTGGCCCTGCTCCCCCATCACCCAGGGTGATAGGGGAGTGGGCTCCACTGCTGTTATTCTAGATGATAACTTTGTAC
CAAAGGCCAATGCCCTAACCTATGACCCATATGTAAACTACTCCTCACGCCATACAATCCCCCAACCCTTCTCCTACCAC
TCCCGTTACTTCACACCCAAACCTGTTCTTGACTCCACTATTGATTACTTCCAACCAAATAACAAAAGGAATCAGCTTTG
GCTGAGGCTACAAACCTCTAGAAATGTGGACCACGTAGGCCTCGGCACTGCGTTCGAAAACAATAAATACGACCAGG
ACTACAATATCCGTGTAACCATGTATGTACAATTCAGAGAATTTAATCTTAAAGACCCCCCACTTAAACCC--------

>AY129155_pcv2a

ATGACGTATCCAAGGAGGCGTTACCGCAGAAGAAGACACCGCCCCCGCAGCCATCTTGGCCAGATCCTCCGCCGCCG
CCCCTGGCTCGTCCACCCCCGCCACCGCTACCGTTGGAGAAGGAAAAATGGCATCTTCAACACCCGCCTCTCCCGCACC
TTCGGATATACTGTCAAGCGTACCACAGTCACAACGCCCTCCTGGGCGGTGGACATGATGAGATTTAAGCTTGACGAC
TTTGTTCCCCCGGGAGGGGGGACCAACAAAATCTCTATACCCTTTGAATACTACAGAATAAGAAAAGTTAAGGTTGAA
TTCTGGCCCTGCTCCCCCATCACCCAGGGTGATAGGGGAGTGGGCTCCACTGCTGTTATTCTAGATGATAACTTTGTAC
CAAAGGCCAATGCTCTAACCTATGACCCATATGTAAACTACTCCTCCCGCCATACAATCCCCCAACCCTTCTCCTACCAC
CCCCGTTACTTCACACCCAAACCTGTTCTTGACTCCACTATTGATTACTTCCAACCAAATAACAAAAGGAATCAGCTTTG
GCTGAGGATACAAACCTCTAGAAATGTGGACCACGTAGGCCTCGGCACTGCGTTCGAAAACAGTAAATACGACCAGG
ACTACAATATCCGTGTAACCATGTATGTACAATTCAGAGAATTTAATCTTAAAGACCCCCCACTTAACCCC--------

>AY129154_pcv2a

ATGACGTATCCAAGGAGGCGTTACCGCAGAAGAAGACACCGCCCCCGCAGCCATCTTGGCCAGATCCTCCGCCGCCG
CCCCTGGCTCGTCCACCCCCGCCACCGCTACCGTTGGAGAAGGAAAAATGGCATCTTCAACACCCGCCTCTCCCGCACC
TTCGGATATACTGTCAAGCGTACCACAGTCACAACGCCCTCCTGGGCGGTGGACATGATGAGATTTAAGCTTGACGAC
TTTGTTCCCCCGGGAGGGGGGACCAACAAAATCTCTATACCCTTTGAATACTACAGAATAAGAAAAGTTAAGGTTGAA
TTCTGGCCCTGCTCCCCCATCACCCAGGGTGATAGGGGAGTGGGCTCCACTGCTGTTATTCTAGATGATAACTTTGTAC
CAAAGTCCGATGCCCTAACCTATGACCCATATGTAAACTACTCCTCCCGCCATACAATCCCCCAACCCTTCTCCTACCAC
TCCCGTTACTTCACACCCAAACCTGTTCTTGACTCCACTATTGATTACTTCCAACCAAATAACAAAAGGAATCAGCTTTG
GCTGAGGCTACAAACCTCTAGAAATGTGGACCACGTAGGCCTCGGCACTGCGTTCGAAAACAGTAAATACGACCAGG
ACTACAATATCCGTGTAACCATGTATGTACAATTCAGAGAATTTAATCTTAAAGACCCCCCACTTAACCCC--------

>EF394774_pcv2a

ATGACGTATCCAAGGAGGCGTTACCGCAGAAGAAGAGACCGCCCCCGCAGCCATCTTGGCCAGATCCTCCGCCGCCG
CCCCTGGCTCGTCCACCCCCGCCACCGCTACCGTTGGAGAAGGAAAAATGGCATCTTCAACACCCGCCTCTCCCGCACC
TTCGGATATACTGTCAAGCGTACCACAGTCACAACGCCCTCCTGGGCGGTGGACATGATGAGATTTAAGCTTGACGAC
TTTGTTCCCCCGGGAGGGGGGACCAACAAAATCTCTATACCCTTTGAATACTACAGAATAAGAAAAGTTAAGGTTGAA
TTCTGGCCCTGTTCCCCCATCACCCAGGGTGATAGGGGAGTGGGCTCCACTGCTGTCATTCTAGATGATAACTTTGTAC
CAAAGGCCAATGCCCTAACCTATGACCCATATGTAAACTACTCCTCCCGCCATACAATCCCCCAACCCTTCTCCTACCAC
TCCCGTTACTTCACACCCAAACCTGTTCTTGACTCCACTATTGATTACTTCCAACCAAATAACAAAAGGAATCAGCTTTG
GCTGAGGCTACAAACCTCTAGAAATGTGGACCACGTAGGCCTCGGCACTGCGTTCGAAAACAGTAAATACGACCAGG
ACTACAATATCCGTGTAACCATGTATGTACAATTCAGAGAATTTAATCTTAAAGACCCCCCACTTAACCCC--------

>KC188796_pcv2a

ATGACGTATCCAAGGAGGCGTTACCGCAGAAGAAGACACCGCCCCCGCAGCCATCTTGGCCAGATCCTCCGCCGCCG
CCCCTGGCTCGTCCACCCTCGCCACCGCTACCGTTGGAGAAGGAAAAATGGCATCTTCAACACCCGCCTCTCCCGCACC
TTCGGATATACTGTCAAGCGTACCACAGTCACAACGCCCTCCTGGGCGGTGGACATGATGAGATTTAAGCTTGACGAC
TTTGTTCCCCCGGGAGGGGGGACCAACAAAATCTCTATACCCTTTGAATACTACAGAATAAGAAAAGTTAAGGTTGAA
TTCTGGCCCTGCTCCCCCATCACCCAGGGTGATAGGGGAGTGGGCTCCACTGCTGTCATTCTAGATGATAACTTTGTAC
CAAAGGCCAATGCCCTAACCTATGACCCATATGTAAACTACTCCTCCCGCCATACAATCCCCCAACCCTTCTCCTACCAC
TCCCGTTACTTCACACCCAAACCTGTTCTGGACTCCACTATTGATTACTTCCAACCAAATAACAAAAGGAATCAGCTTTG
GCTGAGGCTACAAACCTCTAGAAATGTGGACCACGTAGGCCTCGGCACTGCGTTCGAAAACAGTAAATACGACCAGG
ACTACAATATCCGTGTAACCATGTATGTACAATTCAGAGAATTTAATCTTAAAGACCCCCCACTTAACCCC--------

>JF317584_pcv2a

ATGACGTATCCAAGGAGGCGTTACCGCAGAAGAAGACACCGCCCCCGCAGCCATCTTGGCCAGATCCTCCGCCGCCG
CCCCTGGCTCGTCCACCCCCGCCACCGCTACCGTTGGAGAAGGAAAAATGGCATCTTCAACACCCGCCTCTCCCGCACC
TTCGGATATACTGTCAAGCGTACCACAGTCACAACGCCCTCCTGGTCGGTGGACATGATTAGATTTAAGCTTGACGAC
TTTGCTCCCCCGGGAGGGGGGACCAACAAAATCTCTATACCCTTTGAATACTACAGAATAAGAAAAGTTAAGGTTGAA
TTCTGGCCCTGCTCCCCCATCACCCAGGGTGATAGGGGAGTGGGCTCCACTGCTGTCATTCTAGATGATAACTTTGTAC
CAAAGGCCAATGCCCTAACCTATGACCCATATGTAAACTACTCCTCCCGCCATACAATCCCCCAACCCTTCTCCTACCAC
TCCCGTTACTTCACACCCAAACCTGTTCTGGACTCCACTATTGATTACTTCCAACCAAATAACAAAAGGAATCAGCTTTG
GCTGAGGCTACAAACCTCTAGAAATGTGGACCACGTAGGCCTCGGCACTGCGTTCGAAAACAGTAAATACGACCAGG
ACTACAATATCCGTGTAACCATGTATGTACAATTCAGAGAATTTAATCTTAAAGACCCCCCACTTAACCCC--------

>EU503040_pcv2b

ATGACGTATCCAAGGAGGCGTTACCGGAGCAGAAGACACCGCCCCCGCAGCCATCTTGGCCAGATCCTCCGCCGCCG
CCCCTGGCTCGTCCACCCCCGCCACCGTTACCGCTGGAGAAGGAAAAATGGCATCTTCAACACCCGCCTCTCCCGCACC
TTCGGATATACTGTCAAGAGAACCACAGTCAGAACGCCATCCTGGGCGGTGGACATGATGAGATTCAATATTAATGAC
TTTCTTCCCCCAGGAGGGGGCTCAAACCCCCGCTCTGTGCCCTTTGAATACTACAGAATAAGAAAGGTTAAGGTTGAA
TTCTGGCCCTGCTCCCCGATCACCCAGGGTGACAGGGGAGTGGGCTCCAGTGCTGTTATTCTAGATGATAACTTTGTA
ACAAAGGCCACAGCCCTCACCTATGACCCCTATGTAAACTACTCCTCCCGCCATACCATAACCCAGCCCTTCTCCTACCA
CTCCCGTTACTTTACCCCCAAACCTGTTCTTGATTCCACTATTGATTACTTCCAACCAAATAACAAAAGAAATCAGCTGT
GGCTGAGACTACAAACTTCTGGAAATGTAGACCACGTAGGCCTCGGCACTGCGTTCGAAAACAGTAAATACGACCAG
GCATACAATATCCGTGTAACCATGTATGTGCAATTCAGAGAATTTAATCTTAAAGACCCCCCACTTAACCCT--------

>JF690919_pcv2b

ATGACGTATCCAAGGAGGCGTTACCGGAGAAGAAGACACCGCCCCCGCAGCCATCTTGGCCAGATCCTCCGCCGCCG
CCCCTGGCTCGTCCACCCCCGCCACCGTTACCGGTGGAGAAGGAAAAATGGCATCTTCAACACCCGCCTCTCCCGCAC
CTTCGGATATACTATCAAGCGAACCACAGTCAGAACGCCCTCCTGGGCGGTGGACATGATGAGATTCAATATTAATGA
CTTTCTTCCCCCAGGAGGGGGCTCAAACCCCCCCTCTGTGCCCTTTGAATACTACAGAATAAGAAAGGTTAAGGTTGA
ATTCTGGCCCTGCTCCCCGATCACCCAGGGTGACAGGGGAGTGGGCTCCACTGCTGTTATTCTAGATGATAACTTTGT
AACAAAGGCCACAGCCCTGACCTATGACCCCTATGTAAACTACTCCTCCCGCCATACCATACCCCAGCCCTTCTCCTACC
ACTCCCGCTACTTTACCCCCAAACCTGTCCTAGATTCCACTATTGATTACTTCCAACCAAACAACAAAAGAAATCAGCTG
TGGCTGAGACTACAAACTTCTGGAAATGTAGACCACGTAGGCCTCGGCACTGCGTTCGAAAACAGTATATACGACCAG
GACTACAATATCCGTGTAACCATGTATGTACAATTCAGAGAATTTAATCTTAAAGACCCCCCACTTAACCCT--------

>JF690920_pcv2b

ATGACGTATCCAAGGAGGCGTTACCGGAGAAGAAGACACCGCCCCCGCAGCCATCTTGGCCAGATCCTCCGCCGCCG
CCCCTGGCTCGTCCACCCCCGCCACCGTTACCGTTGGAGAAGGAAAAATGGCATCTTCAACACCCGCCTCTCCCGCACC
TTCGGATATACTATCAAGCGGACCACAGTCAGAACGCCCTCCTGGGCGGTGGACATGATGAGATTCAATATTAATGAC
TTTCTTCCCCCAGGAGGGGGCTCAAACCCCCTCTCTGTGCCCTTTGAATACTACAGAATAAGAAAGGTTAAGGTTGAAT
TCTGGCCCTGCTCCCCGATCACCCAGGGTGACAGGGGAGTGGGCTCCACTGCTGTTATTCTAGATGATAACTTTGTAA
CAAAGGCCACAGCCCTGACCTATGACCCCTATGTAAACTACTCCTCCCGCCATACCATACCCCAGCCCTTCTCCTACCAC
TCCCGCTACTTTACCCCCAAACCTGTCCTAGATTCCACTATTGATTACTTCCAACCAAACAACAAAAGAAATCAGCTGTG
GCTGAGACTACAAACTTCTGGAAATGTAGACCACGTAGGCCTCGGCACTGCGTTCGAAAACAGTATATACGACCAGG
ACTACAATATCCGTGTAACCATGTATGTACAATTCAGAGAATTTAATCTTAAAGACCCCCCACTTAACCCT--------

>JN382175_pcv2b

ATGACGTATCCAAGGAGGCGTTACCGGAGAAGAAGACACCGCCCCCGCAGCCATCTTGGCCAGATCCTCCGCCGCCC
CCCGTTGTTCGTCCACCCCCGCCACCGTTACCGCTGGAGAAGGAAAAATGGCATCTTCAACACCCGCCTCTCCCGCACC
TTCGGATATACTATCAAGCGAACCACAGTCAAAACGCCCTCGTGGGCGGTGGACATGATGAGATTTAATATTAATGAT
TTTCTTCCCCCAGGAGGGGGCTCAAACCCCCTCACTGTGCCCTTTGAATACTACAGAATAAGAAAGGTTAAGGTTGAA
TTCTGGCCCTGCTCCCCAATCACCCAGGGTGACAGGGGAGTGGGCTCCACTGCTGTTATTCTAGATGATAACTTTGTAA
CAAAGGCCAATGCCCTCACCTATGACCCCTATGTAAACTACTCCTCCCGCCATACCATACCCCAGCCCTTCTCCTACCAC
TCCCGCTACTTTACCCCCAAACCTCTCCTAGACTCCACTATTGATTACCTCCAACCAAACAACAAAAGAAATCAGCTGTG
GCTGAGACTACAAACTGCTGGAAATGTAGACCACGTAGGCCTCGGCACTGCGTTCGAAAACAGTATATACGACCAGG
AATACAATATCCGTGTAACCATGTATGTACAATTCAGAGAATTTAATCTTAAAGACCCCCCACTTAACCCT--------

>JN382185_pcv2b

ATGACGTATCCAAGGAGGCGTTACCGGAGAAGAAGACACCGCCCCCGCAGCCATCTTGGCCAGATCCTCCGCCGCCG
CCCCTGGCTCGTCCACCCCCGCCACCGCTACCGTTGGAGAAGGAAAAATGGAATCTTCAACACCCGCCTCTCCCGCACC
TTCGGATATACTATCAAGCGAACCACAGTCAAAACCCCCTCCTGGGCGGTGGACATGATGAGATTTAATATTAATGAT
TTTCTTCCCCCAGGAGGGGGCTCAAACCCCCGCTCTGTGCCCTTTGAATACTACAGAATAAGGAAGGTTAAGGTTGAA
TTCTGGCCCTGCTCCCCAATCACCCAGGGTGACAGGGGAGTGGGCTCCACTGCTGTTATTCTAGATGATAACTTTGTAA
CAAAGGCCAATGCCCTCACCTATGACCCCTATGTAAACTACTCCTCCCGCCATACCATAACCCAGCCCTTCTCCTACCAC
TCCCGCTACTTTACCCCCAAACCTGTCCTAGATTCCACTATTGATTACTTCCAACCCAACAACAAAAGAAATCAGCTGTG
GCTGAGACTACAAACTGCTGGAAATGTAGACCACGTAGGCCTCGGCACTGCGTTCGAAAACAGTATATACGACCAGG
ACTACAATATCCGTGTAACCATGTATGTACAATTCAGAGAATTTAATCTTAAAGACCCCCCACTTAACCCT--------

>HM030908_pcv2b

ATGACGTATCCAAGGAGGCGTTACCGGAGAAGAAGACACCGCCCCCGCAGCCATCTTGGCCAGATCCTCCGCCGCCG
CCCCTGGCTCGTCCACCCCCGCCACCGTTACCGCTGGAGAAGGAAAAATGGCATCTTCAACACCCGCCTCTCCCGCACC
TTCGGATATACTATCAAGCGAACCACAGTCAGAACGCCCTCCTGGGCGGTGGACATGATGAGATTCAATATTAATGAC
TTTCTTCCCCCAGGAGGGGGCTCAAACCCCCGCTCTGTGCCCTTTGAATACTACAGAATAAGAAAGGTTAAGGTTGAA
TTCTGGCCCTGCTCCCCGATCACCCAGGGTGACAGGGGAGTGGGCTCCACTGCTGTTATTCTAGATGATAACTTTGTA
ACAAAGGCCACAGCCCTCACCTATGACCCCTATGTAAACTACTCCTCCCGCCATACCATAACCCAGCCCTTCTCCTACCA
CTCCCGCTACTTTACCCCCAAACCTGTCCTAGATTCCACTATTGATTACTTCCAACCAAACAACAAAAGAAATCAGCTGT
GGCTGAGACTGCAAACTGCTGGAAATGTAGACCACGTAGGCCTCGGCACTGCGTTCGAAAACAGTATATACGACCAG
GAATACAATATCCGTGTAACCATGTATGTACAATTCAGAGAATTTAATCTTAAAGACCCCCCACTTAACCCT--------

>JF272497_pcv2b

ATGACGTATCCAAGGAGGCGTTACCGGAGAAGAAGACACCGCCCCCGCAGCCATCTTGGCCAGATCCTCCGCCGCCG
CCCCTGGCTCGTCCACCCCCGCCACCGTTACCGCTGGAGAAGGAAAAATGGCATCTTCAACACCCGCCTCTCCCGCACC
TTCGGATATACTATCAAGCGAACCACAGTCAGAACGCCCTCCTGGGCGGTGGACATGATGAGATTCAATATTAATGAC
TTTCTTCCCCCAGGAGGGGGCTCAAACCCCCGCTCTGTGCCCTTTGAATACTACAGAATAAGAAAGGTTAAGGTTGAA
TTCTGGCCCTGCTCCCCGATCACCCAGGGTGACAGGGGAGTGGGCTCCACTGCTGTTATTCTAGATGATAACTTTGTA
ACAAAGGCCACAGCCCTCACCTATGACCCCTATGTAAACTACTCCTCCCGCCATACCATAACCCAGCCCTTCTCCTACCA
CTCCCGCTACTTTACCCCCAAACCTGTCCTAGATTCCACTATTGATTACTTCCAACCAAACAACAAAAGAAATCAGCTGT
GGCTGAGACTGCAAACTGCTGGAAATGTAGACCACGTAGGCCTCGGCACTGCGTTCGAAAACAGTATATACGACCAG
GAATACAATATCCGTGTAACCATGTATGTACAATTCAGAGAATTTAATCTTAAAGACCCCCCACTTAACCCT--------

>EU980089_pcv2b

ATGACGTATCCAAGGAGGCGTTACCGGAGAAGAAGACACCGCCCCCGCAGCCATCTTGGCCAGATCCTCCGCCGCCG
CCCCTGGCTCGTCCACCCCCGCCACCGTTACCGCTGGAGAAGGAAAAATGGCATCTTCAACACCCGCCTCTCCCGCACC
TTCGGATATACTATCAAGCGAACCACAGTCAGAACGCCCTCCTGGGCGGTGGACATGATGAGATTCAATATTAATGAC
TTTCTTCCCCCAGGAGGGGGCTCAAACCCCCGCTCTGTGCCCTTTGAATACTACAGAATAAGAAAGGTTAAGGTTGAA
TTCTGGCCCTGCTCCCCGATCACCCAGGGTGACAGGGGAGTGGGCTCCAGTGCTGTTATTCTAGATGATAACTTTGTA
ACAAAGGCCACAGCCCTCACCTATGACCCCTATGTAAACTACTCCTCCCGCCATACCATAACCCAGCCCTTCTCCTACCA
CTCCCGCTACTTTACCCCCAAACCTGTCCTAGATTCCACTATTGATTACTTCCAACCAAACAACAAAAGAAACCAGCTGT
GGCTGAGGCTACAAACTGCTGGAAATGTAGACCACGTAGGCCTCGGCACTGCGTTCGAAAACAGTATATACGACCAG
GAATACAATATCCGTGTAACCATGTATGTACAATTCAGAGAATTTAATCTTAAAGACCCCCCACTTAACCCT--------

>EU980090_pcv2b

ATGACGTATCCAAGGAGGCGTTACCGGAGAAGAAGACACCGCCCCCGCAGCCATCTTGGCCAGATCCTCCGCCGCCG
CCCCTGGCTCGTCCACCCCCGCCACCGTTACCGCTGGAGAAGGAAAAATGGCATCTTCAACACCCGCCTCTCCCGCACC
TTCGGATATACTATCAAGCGAACCACAGTCAGAACGCCCTCCTGGGCGGTGGACATGATGAGATTCAATATTAATGAC
TTTCTTCCCCCAGGAGGGGGCTCAAACCCCCGCTCTGTGCCCTTTGAATACTACAGAATAAGAAAGGTTAAGGTTGAA
TTCTGGCCCTGCTCCCCGATCACCCAGGGTGACAGGGGAGTGGGCTCCAGTGCTGTTATTCTAGATGATAACTTTGTA
ACAAAGGCCACAGCCCTCACCTATGACCCCTATGTAAACTACTCCTCCCGCCATACCATAACCCAGCCCTTCTCCTACCA
CTCCCGCTACTTTACCCCCAAACCTGTCCTAGATTCCACTATTGATTACTTCCAACCAAACAACAAAAGAAACCAGCTGT
GGCTGAGGCTACAAACTGCTGGAAATGTAGACCACGTAGGCCTCGGCACTGCGTTCGAAAACAGTATATACGACCAG
GAATACAATATCCGTGTAACCATGTATGTACAATTCAGAGAATTTAATCTTAAAGACCCCCCACTTAACCCT--------

>EU545551_pcv2b

ATGACGTATCCAAGGAGGCGTTACCGGAGAAGAAGACACCGCCCCCGCAGCCATCTTGGCCAGATCCTCCGCCGCCG
CCCCTGGCTCGTCCACCCCCGCCACCGTTACCGCTGGAGAAGGAAAAATGGCATCTTCAACACCCGCCTCTCCCGCACC
TTCGGATATACTATCAAGAGAACCACAGTCAGAACGCCCTCCTGGGCGGTGGACATGATGAGATTCAATATTAATGAC
TTTCTTCCCCCAGGAGGGGGCTCAAACCCCCGCTCTGTGCCCTTTGAATACTACAGAATAAGAAAGGTTAAGGTTGAA
TTCTGGCCCTGCTCCCCGATCACCCAGGGTGACAGGGGAGTGGGCTCCAGTGCTGTTATTCTAGATGATAACTTTGTA
ACAAAGGCCACAGCCCTCACCTATGACCCCTATGTAAACTACTCCTCCCGCCATACCATAACCCAGCCCTTCTCCTACCA
CTCCCGCTACTTTACCCCCAAACCTGTCCTAGATTCCACTATTGATTACTTCCAACCAAACAACAAAAGAAATCAGCTGT
GGCTGAGGCTACAAACTGCTGGAAATGTAGACCACGTAGGCCTCGGCACTGCGTTCGAAAACAGTATATACGACCAG
GAATACAATATCCGTGTAACCATGTATGTACAATTCAGAGAATTTAATCTTAAAGACCCCCCACTTAACCCT--------

>KC835193_pcv2b

ATGACGTATCCAAGGAGGCGTTACCGGAGAAGAAGACACCGCCCCCGCAGCCATCTTGGCCAGATCCTCCGCCGCCG
CCCCTGGCTCGTCCACCCCCGCCACCGTTACCGCTGGAGAAGGAAAAATGGCATCTTCAACACCCGCCTCTCCCGCACC
TTCGGATATACTATCAAGCGAACCACAGTCAGAACGCCCTCCTGGGCGGTGGACATGATGAGATTCAATATTAATGAC
TTTCTTCCCCCAGGAGGGGGCTCAAACCCCCGCTCTGTGCCCTTTGAATACTACAGAATAAGAAAGGTTAAGGTTGAA
TTCTGGCCCTGCTTCCCGATCACCCAGGGTGACAGGGGAGTGGGCTCCAGTGCTGTTATTCTAGATGATAACTTTGTA
ACAAAGGCCACAGCCCTCACCTATGACCCCTATGTAAACTACTCCTCCCGCCATACCATAACCCAGCCCTTCTCCTACCA
CTCCCGCTACTTTACCCCCAAACCTGTCCTAGATTCCACTATTGATTATTTCCAACCAAACAACAAAAGAAATCAGCTGT
GGCTGAGACTACAAACTGCTGGAAATGTAGACCACGTAGGCCTCGGCACTGCGTTCGAAAACAGTATATACGACCAG
GACTACAATATCCGTGTAACCATGTATGTACAATTCAGAGAATTTAATCTTAAAGACCCCCCACTTAACCCT--------

>KC835190_pcv2b

ATGACGTATCCAAGGAGGCGTTACCGGAGAAGAAGACACCGCCCCCGCAGCCATCTTAGCCAGATCCTCCGCCGCCG
CCCCTGGCTCGTCCACCCCCGCCACCGTTACCGCTGGAGAAGGAAAAATGGCATCTTCAACACCCGCCTCTCCCGCACC
TTCGGATATACTATCAAGCGAACCACAGTCAGAACGCCCTCCTGGGCGGTGGACATGATGAGATTCAATATTAATGAC
TTTCTTCCCCCAGGAGGGGGCTCAAACCCCCGCTCTGTGCCCTTTGAATACTACAGAATAAGAAAGGTTAAGGTTGAA
TTCTGGCCCTGCTCCCCGATCACCCAGGGTGACAGGGGAGTGGGCTCCAGTGCTGTTATTCTAGATGATAACTTTGTA
ACAAAGGCCACAGCCCTCACCTATGACCCCTATGTAAACTACTCCTCCCGCCATACCATAACCCAGCCCTTCTCCTACCA
CTCCCGCTACTTTACCCCCAAACCTGTCCTAGATTCCACTATTGATTACTTCCAACCAAACAACAAAAGAAACCAGCTGT
GGCTGAGACTACAAACTGCTGGAAATGTAGACCACGTAGGCCTCGGCACTGCGTTCGAAAACAGTATATACGACCAG
GACTACAATATCCGTGTAACCATGTATGTACAATTCAGAGAATTTAATCTTAAAGACCCCCCACTTAACCCT--------

>HQ831525_pcv2b

ATGACGTATCCAAGGAGGCGTTACCGGAGAAGAAGACACCGCCCCCGCAGCCATCTTGGCCAGATCCTCCGCCGCCG
CCCCTGGCTCGTCCACCCCCGCCACCGTTACCGCTGGAGAAGGAAAAATGGCATCTTCAACACCCGCCTCTCCCGCACC
TTCGGATATACTATCAAGCGAACCACAGTCAGAACGCCCTCCTGGGCGGTGGACATGATGAGATTCAATATTAATGAC
TTTCTTCCCCCAGGAGGGGGCTCAAACCCCCGCTCTGTGCCCTTTGAATACTACAGAATAAGAAAGGTTAAGGTTGAA
TTCTGGCCCTGCTCCCCGATCACCCAGGGTGACAGGGGAGTGGGCTCCAGTGCTGTTATTCTAGATGATAACTTTGTA
ACAAAGGCCACAGCCCTCACCTATGACCCCTATGTAAACTACTCCTCCCGCCATACCATAACCCAGCCCTTCTCCTACCA
CTCCCGCTACTTTACCCCCAAACCTGTCCTAGATTCCACTATTGATTACTTCCAACCAAACAACAAAAGAAATCAGCTGT
GGCTGAGACTACAAACTTCTGGAAATGTAGACCACGTAGGCCTCGGCACTGCGTTCGAAAACAGTATATACGACCAG
GAATACAATATCCGTGTAACCATGTATGTACAATTCAGAGAATTTAATCTTAAAGACCCCCCACTTAACCCT--------

>GQ915289_pcv2b

ATGACGTATCCAAGGAGGCGTTACCGGAGAAGAAGACACCGCCCCCGCAGCCATCTTGGCCAGATCCTCCGCCGCCG
CCCCTGGCTCGTCCACCCCCGCCACCGTTACCGCTGGAGAAGGAAAAATGGCATCTTCAACACCCGCCTCTCCCGCACC
TTCGGATATACTATCAAGCGAACCACAGTCAGAACGCCCTCCTGGGCGGTGGACATAATGAGATTCAATATTAATGAC
TTTCTTCCCCCAGGAGGGGGCTCAAACCCCCGCTCTGTGCCCTTTGAATACTACAGAATAAGAAAGGTTAAGGTTGAA
TTCTGGCCCTGCTCCCCGATCACCCAGGGTGACAGGGGAGTGGGCTCCAGTGCTGTTATTCTAGATGATAACTTTGTA
ACAAAGGCCACTGCCCTCACCTATGACCCCTATGTAAACTACTCCTCCCGCCATACCATAACCCAGCCCTTCTCCTACCA
CTCCCGCTACTTTACCCCCAAACCTGTCCTAGATTCCACTATTGATTACTTCCAACCAAACAACAAAAGAAATCAGCTGT
GGCTGAGACTACAAACTGCTGGAAATGTAGACCACGTAGGCCTCGGCACTGCGTTCGAAAACAGTATATACGACCAG
GAATACAATATCCGTGTAACCATGTATGTACAATTCAGAGAATTTAATCTTAAAGACCCCCCACTTAACCCT--------

>HM038029_pcv2b

ATGACGTATCCAAGGAGGCGTTACCGGAGAAGAAGACACCGCCCCCGCAGCCATCTTGGCCAGATCCTCCGCCGCCG
CCCCTGGCTCGTCCACCCCCGCCACCGTTACCGCTGGAGAAGGAAAAATGGCATCTTCAACACCCGCCTCTCCCGCACC
TTCGGATATACTATCAAGCGAACCACAGTCAGAACGCCCTCCTGGGCGGTGGACATGATGAGATTCAATATTAATGAC
TTTCTTCCCCCAGGAGGGGGCTCAAACCCCCGCTCTGTGCCCTTTGAATACTACAGAATAAGAAAGGTTAAGGTTGAA
TTCTGGCCCTGCTCCCCGATCACCCAGGGTGACAGGGGAGTGGGCTCCAGTGCTGTTATTCTAGATGATAACTTTGTA
ACAAAGGCCACAGCCCTCACCTATGACCCCTATGTAAACTACTCCTCCCGCCATACCATAACCCAACCCTTCTCCTACCA
CTCCCGCTACTTTACCCCCAAACCTGTCCTAGATTCCACTATTGATTACTTCCAACCAAACAACAAAAGAAATCAGCTGT
GGCTGAGACTACAAACTGCTGGAAATGTAGACCACGTAGGCCTCGGCACTGCGTTCGAAAACAGTATATACGACCAG
GAATACAATATCCGTGTAACCATGTATGTACAATTCAGAGAATTTAATCTTAAAGACCCCCCACTTAACCCT--------

>HM535639_pcv2b

ATGACGTATCCAAGGAGGCGTTACCGGAGAAGACGACACCGCCCCCGCAGCCATCTTGGCCAGATCCTCCGCCGCCG
CCCCTGGCTCGTCCACCCCCGCCACCGTTACCGCTGGAGAAGGAAAAATGGCATCTTCAACACCCGCCTCTCCCGCACC
TTCGGATATACTATCAAGCGAACCACAGTCAGAACGCCCTCCTGGGCGGTGGACATGATGAGATTCAATATTAATGAC
TTTCTTCCCCCAGGAGGGGGCTCAAACCCCCGCTCTGTACCCTTTGAATACTACAGAATAAGAAAGGTTAAGGTTGAA
TTCTGGCCCTGCTCCCCGATCACCCAGGGTGACAGGGGAGTGGGCTCCAGTGCTGTTATTCTAGATGATAACTTTGTA
ACAAAGGCCACAGCCCTCACCTATGACCCCTATGTAAACTACTCCTCCCGCCATACCATAACCCAGCCCTTCTCCTACCA
CTCCCGCTACTTTACCCCCAAACCTGTCCTAGATTCCACTATTGATTACTTCCAACCAAACAACAAAAGAAATCAGCTGT
GGCTGAGACTACAAACTGCTGGAAATGTAGACCACGTAGGCCTCGGCACTGCGTTCGAAAACAGTATATACGACCAG
GAATACAATATCCGTGTAACCATGTATGTACAATTCAGAGAATTTAATCTTAAAGACCCCCCACTTAACCCT--------

>JF272499_pcv2b

ATGACGTATCCAAGGAGGCGTTACCGGAGAAGACGACACCGCCCCCGCAGCCATCTTGGCCAGATCCTCCGCCGCCG
CCCCTGGCTCGTCCACCCCCGCCACCGTTACCGCTGGAGAAGGAAAAATGGCATCTTCAACACCCGCCTCTCCCGCACC
TTCGGATATACTATCAAGCGAACCACAGTCAGAACGCCCTCCTGGGCGGTGGACATGATGAGATTCAATATTAATGAC
TTTCTTCCCCCAGGAGGGGGCTCAAACCCCCGCTCTGTACCCTTTGAATACTACAGAATAAGAAAGGTTAAGGTTGAA
TTCTGGCCCTGCTCCCCGATCACCCAGGGTGACAGGGGAGTGGGCTCCAGTGCTGTTATTCTAGATGATAACTTTGTA
ACAAAGGCCACAGCCCTCACCTATGACCCCTATGTAAACTACTCCTCCCGCCATACCATAACCCAGCCCTTCTCCTACCA
CTCCCGCTACTTTACCCCCAAACCTGTCCTAGATTCCACTATTGATTACTTCCAACCAAACAACAAAAGAAATCAGCTGT
GGCTGAGACTACAAACTGCTGGAAATGTAGACCACGTAGGCCTCGGCACTGCGTTCGAAAACAGTATATACGACCAG
GAATACAATATCCGTGTAACCATGTATGTACAATTCAGAGAATTTAATCTTAAAGACCCCCCACTTAACCCT--------

>HQ395057_pcv2b

ATGACGTATCCAAGGAGGCGTTACCGGAGAAGAAGACACCGCCCCCGCAGCCATCTTGGCCAGATCCTCCGCCGCCG
CCCCTGGCTCGTCCACCCCCGCCACCGTTACCGCTGGAGAAGGAAAAATGGCATCTTCAACACCCGCCTCTCCCGCACC
TTCGGATATACTATCAAGCGAACCACAGTCAGAACGCCCTCCTGGGCGGTGGACATGATGAGATTCAATATTAATGAC
TTTCTTCCCCCAGGAGGGGGCTCAAACCCCCGCTCTGTACCCTTTGAATACTACAGAATAAGAAAGGTTAAGGTTGAA
TTCTGGCCCTGCTCCCCGATCACCCAGGGTGACAGGGGAGTGGGCTCCAGTGCTGTTATTCTAGATGATAACTTTGTA
ACAAAGGCCACAGCCCTCACCTATGACCCCTATGTAAACTACTCCTCCCGCCATACCATAACCCAGCCCTTCTCCTACCA
CTCCCGCTACTTTACCCCAAAACCTGTCCTAGATTCCACTATTGATTACTTCCAACCAAACAACAAAAGAAATCAGCTGT
GGCTGAGACTACAAACTGCTGGAAATGTAGACCACGTAGGCCTCGGCACTGCGTTCGAAAACAGTATATACGACCAG
GAATACAATATCCGTGTAACCATGTATGTACAATTCAGAGAATTTAATCTTAAAGACCCCCCACTTAACCCT--------

>HM565923_pcv2b

ATGACGTATCCAAGGAGGCGTTACCGGAGAAGAAGACACCGCCCCCGCAGCCATCTTGGCCAGATCCTCCGCCGCCG
CCCCTGGCTCGTCCACCCCCGCCACCGTTACCGCTGGAGAAGGAAAAATGGCATCTTCAACACCCGCCTCTCCCGCACC
TTCGGATATACTATCAAGCGAACCACAGTCAGAACGCCCTCCTGGGCGGTGGACATGATGAGATTCAATATTAATGAC
TTTCTTCCCCCAGGAGGGGGCTCAAACCCCCGCTCTGTACCCTTTGAATACTACAGAATAAGAAAGGTTAAGGTTGAA
TTCTGGCCCTGCTCCCCGATCACCCAGGGTGACAGGGGAGTGGGCTCCAGTGCTGTTATTCTAGATGATAACTTTGTA
ACAAAGGCCACAGCCCTCACCTATGACCCCTATGTAAACTACTCCTCCCGCCATACCATAACCCAGCCCTTCTCCTACCA
CTCCCGCTACTTTACCCCCAAACCTGTCCTAGATTCCACTATTGATTACTTCCAACCAAACAACAAAAGAAACCAGCTGT
GGCTGAGACTACAAACTACTGGAAATGTAGACCACGTAGGCCTCGGCACTGCGTTCGAAAACAGTATATACGACCAG
GAATACAATATCCGTGTAACCATGTATGTACAATTCAGAGAATTTAATCTTAAAGACCCCCCACTTAACCCT--------

>JX512859_pcv2b

ATGACGTATCCAAGGAGGCGTTACCGGAGAAGAAGACACCGCCCCCGCAGCCATCTTGGCCAGATCCTCCGCCGCCG
CCCCTGGCTCGTCCACCCCCGCCACCGTTACCGCTGGAGAAGGAAAAATGGCATCTTCAACACCCGCCTCTCCCGCACC
TTCGGATATACTATCAAGCGAACCACAGTCAAAACGCCCTCCTGGGCGGTGGACATGATGCGATTCAATATTAATGAC
TTTCTTCCCCCAGGAGGGGGCTCAAACCCCCGCTCTGTACCCTTTGAATACTACAGAATAAGAAAGGTTAAGGTTGAA
TTCTGGCCCTGCTCCCCGATCACCCAGGGTGACAGGGGAGTGGGCTCCAGTGCTGTTATTCTAGATGATAACTTTGTA
ACAAAGGCCACAGCCCTCACCTATGACCCCTATGTAAACTACTCCTCCCGCCATACCATAACCCAGCCCTTCTCCTACCA
CTCCCGCTACTTTACCCCCAAACCTGTCCTAGATTCCACTATTGATTACTTCCAACCAAACAACAAAAGAAATCAGCTGT
GGCTGAGACTACAAACTACTGGAAATGTAGACCACGTAGGCCTCGGCACTGCGTTCGAAAACAGTATATACGACCAG
GAATACAATATCCGTGTAACCATGTATGTACAATTCAGAGAATTTAATCTTAAAGACCCCCCACTTAACCCT--------

>FN398026_pcv2b

ATGACGTATCCAAGGAGGCGTTACCGGAGAAGAAGACACCGCCCCCGCAGCCATCTTGGCCAGATCCTCCGCCGCCG
CCCCTGGCTCGTCCACCCCCGCCACCGTTACCGCTGGAGAAGGAAAAATGGCATCTTCAACACCCGCCTCTCCCGCACC
TTCGGATATACTATCAAGCGAACCACAGTCAAAACGCCCTCCTGGGCGGTGGACATGATGAGATTCAATATTAATGAC
TTTCTTCCCCCAGGAGGGGGCTCAAACCCCCGCTCTGTACCCTTTGAATACTACAGAATAAGAAAGGTTAAGGTTGAA
TTCTGGCCCTGCTCCCCGATCACCCAGGGTGACAGGGGAGTGGGCTCCAGTGCTGTTATTCTAGATGATAACTTTGTA
ACAAAGGCCACAGCCCTCACCTATGACCCCTATGTAAACTACTCCTCCCGCCATACCATAACCCAGCCCTTCTCCTACCA
CTCCCGCTACTTTACCCCCAAACCTGTCCTAGATTCCACTATTGATTACTTCCAACCAAACAACAAAAGAAATCAGCTGT
GGCTGAGACTACAAACTACTGGAAATGTAGACCACGTAGGCCTCGGCACTGCGTTCGAAAACAGCATATACGACCAG
GAATACAATATCCGTGTAACCATGTATGTACAATTCAGAGAATTTAATCTTAAAGACCCCCCACTTAACCCT--------

>HM009332_pcv2b

ATGACGTATCCAAGGAGGCGTTACCGGAGAAGAAGACACCGCCCCCGCAGCCATCTTGGCCAGATCCTCCGCCGCCG
CCCCTGGCTCGTCCACCCCCGCCACCGTTACCGCTGGAGAAGGAAAAATGGCATCTTCAACACCCGCCTCTCCCGCACC
TTCGGATATACTATCAAGCGAACCACAGTCAAAACGCCCTCCTGGGCGGTGGACATGATGAGATTCAATATTAATGAC
TTTCTTCCCCCAGGAGGGGGCTCAAACCCCCGCTCTGTACCCTTTGAATACTACAGAATAAGAAAGGTTAAGGTTGAA
TTCTGGCCCTGCTCCCCGATCACCCAGGGTGACAGGGGAGTGGGCTCCAGTGCTGTTATTCTAGATGATAACTTTGTA
ACAAAGGCCACAGCCCTCACCTATGACCCCTATGTAAACTACTCCTCCCGCCATACCATAACCCAGCCCTTCTCCTACCA
CTCCCGCTACTTTACCCCCAAACCTGTCCTAGATTCCACTATTGATTACTTCCAACCAAACAACAAAAGAAATCAGCTGT
GGCTGAGACTACAAACTGCTGGAAATGTAGACCACGTAGGCCTCGGCACTGCGTTCGAAAACAGTATATACGACCAG
GAATACAATATCCGTGTAACCATGTATGTACAATTCAGAGAATTTAATCTTAAAGACCCCCCACTTAACCCA--------

>FJ870969_pcv2b

ATGACGTATCCAAGGAGGCGTTACCGGAGAAGAAGACACCGCCCCCGCAGCCATCTTGGCCAGATCCTCCGCCGCCG
CCCCTGGCTCGTCCACCCCCGCCACCGTTACCGCTGGAGAAGGAAAAATGGCATCTTCAACACCCGCCTCTCCCGCACC
TTCGGATATACTATCAAGCGAACCACAGTCAGAACGCCCTCCTGGGCGGTGGACATGATGAGATTCAATATTAATGAC
TTTCTTCCCCCAGGAGGGGGCTCAAACCCCCGCTCTGTGCCCTTTGAATACTACAGAATAAGAAAGGTTAAGGTTGAA
TTCTGGCCCTGCTCCCCGATCACCCAGGGTGACAGGGGAGTGGGCTCCAGTGCCGTTATTCTAGATGATAACTTTGTA
ACAAAGGCCACAGCCCTCACCTATGACCCCTATGTAAACTACTCCTCCCGCCATACCATAACCCAGCCCTTCTCCTACCA
CTCCCGCTACTTTACCCCCAAACCTGTCCTAGATTCCACTATTGATTACTTCCAACCAAACAACAAAAGAAATCAGCTGT
GGCTGAGACTACAAACTGCTGGAAATGTAGACCACGTAGGCCTCGGCACTGCGTTCGAAAACAGTATATACGACCAG
GAATACAATATCCGTGTAACCATGTATGTACAATTCAGAGAATTTAATCTTAAAGACCCCCCACTTAACCCC--------

>FJ905468_pcv2b

ATGACGTATCCAAGGAGGCGTTACCGGAGAAGAAGACACCGCCCCCGCAGCCATCTTGGCCAGATCCTCCGCCGCCG
CCCCTGGCTCGTCCACCCCCGCCACCGTTACCGCTGGAGAAGGAAAAATGGCATCTTCAACACCCGCCTCTCCCGCACC
TTCGGATATACTATCAAGCGAACCACAGTCAAAACGCCCTCCTGGGCGGTGGACATGATGAGATTCAATATTAATGAC
TTTCTTCCCCCAGGAGGGGGCTCAAACCCCCGCTCTGTGCCCTTTGAATACTACAGAATAAGAAAGGTTAAGGTTGAA
TTCTGGCCCTGCTCCCCGATCACCCAGGGTGACAGGGGAGTGGGCTCCAGTGCTGTTATTCTAGATGATAACTTTGTA
ACAAAGGCCACAGCCCTCACCTATGACCCCTATGTAAACTACTCCTCCCGCCATACCATAACCCAGCCCTTCTCCTACCA
CTCCCGCTACTTTACCCCCAAACCTGTCCTAGATTCCACTATTGATTACTTCCAACCAAACAACAAAAGAAATCAGCTGT
GGCTGAGACTACAAACTGCTGGAAATGTAGACCACGTAGGCCTCGGCACTGCGTTCGAAAACAGTATATACGACCAG
GAATACAATATCCGTGTAACCATGTATGTACAATTCAGAGAATTTAATCTTAAAGACCCCCCACTTAACCCC--------

>JF317568_pcv2b

ATGACGTATCCAAGGAGGCGTTACCGGAGAAGAAGACACCGCCCCCGCAGCCATCTTGGCCAGATCCTCCGCCGCCG
CCCCTGGCTCGTCCACCCCCGCCACCGTTACCGCTGGAGAAGGAAAAATGGCATCTTCAACACCCGCCTCTCCCGCACC
TTCGGATATACTATAAAGCGAACCACAGTCAAAACGCCCTCCTGGGCGGTGGACATGATGAGATTCAATATTAATGAC
TTTCTTCCCCCAGGAGGGGGCTCAAACCCCCGCTCTGTGCCCTTTGAATACTACAGAATAAGAAAGGTTAAGGTTGAA
TTCTGGCCCTGCTCCCCGATCACCCAGGGTGACAGGGGAGTGGGCTCCAGTGCTGTTATTCTAGATGATAACTTTGTA
ACAAAGGCCACAGCCCTCACCTATGACCCCTATGTAAACTACTCCTCCCGCCATACCATAACCCAGCCCTTCTCCTACCA
CTCCCGCTACTTTACCCCCAAACCTGTCCTAGATTCCACTATTGATTACTTCCAACCAAACAACAAAAGAAATCAGCTGT
GGCTGAGACTACAAACTGCTGGAAATGTAGACCACGTAGGCCTCGGCACTGCGTTCGAAAACAGTATATACGACCAG
GAATACAATATCCGTGTAACCATGTATGTACAATTCAGAGAATTTAATCTTAAAGACCCCCCACTTAACCCC--------

>JF317583_pcv2b

ATGACGTATCCAAGGAGGCGTTACCGGAGAAGAAGACACCGCCCCCGCAGCCATCTTGGCCAGATCCTCCGCCGCCG
CCCCTGGCTCGTCCACCCCCGCCACCGTTACCGCTGGAGAAGGAAAAATGGCATCTTCAACACCCGCCTCTCCCGCACC
TTCGGATATACTATAAAGCGAACCACAGTCAAAACGCCCTCCTGGGCGGTGGACATGATGAGATTCAATATTAATGAC
TTTCTTCCCCCAGGAGGGGGCTCAAACCCCCGCTCTGTGCCCTTTGAATACTACAGAATAAGAAAGGTTAAGGTTGAA
TTCTGGCCCTGCTCCCCGATCACCCAGGGTGACAGGGGAGTGGGCTCCAGTGCTGTTATTCTAGATGATAACTTTGTA
ACAAAGGCCACAGCCCTCACCTATGACCCCTATGTAAACTACTCCTCCCGCCATACCATAACCCAGCCCTTCTCCTACCA
CTCCCGCTACTTTACCCCCAAACCTGTCCTAGATTCCACTATTGATTACTTCCAACCAAACAACAAAAGAAATCAGCTGT
GGCTGAGACTACAAACTGCTGGAAATGTAGACCACGTAGGCCTCGGCACTGCGTTCGAAAACAGTATATACGACCAG
GAATACAATATCCGTGTAACCATGTATGTACAATTCAGAGAATTTAATCTTAAAGACCCCCCACTTAACCCC--------

>JF317577_pcv2b

ATGACGTATCCAAGGAGGCGTTACCGGAGAAGAAGACACCGCCCCCGCAGCCATCTTGGCCAGATCCTCCGCCGCCG
CCCCTGGCTCGTCCACCCCCGCCACCGTTACCGCTGGAGAAGGAAAAATGGCATCTTCAACACCCGCCTCTCCCGCACC
TTCGGATATACTATAAAGCGAACCACAGTCAAAACGCCCTCGTGGGCGGTGGACATGATGAGATTCAATATTAATGAC
TTTCTTCCCCCAGGAGGGGGCTCAAACCCCCGCTCTGTGCCCTTTGAATACTACAGAATAAGAAAGGTTAAGGTTGAA
TTCTGGCCCTGCTCCCCGATCACCCAGGGTGACAGGGGAGTGGGCTCCAGTGCTGTTATTCTAGATGATAACTTTGTA
ACAAAGGCCACAGCCCTCACCTATGACCCCTATGTAAACTACTCCTCCCGCCATACCATAACCCAGCCCTTCTCCTACCA
CTCCCGCTACTTTACCCCCAAACCTGTCCTAGATTCCACTATTGATTACTTCCAACCAAACAACAAAAGAAATCAGCTGT
GGCTGAGACTACAAACTGCTGGAAATGTAGACCACGTAGGCCTCGGCACTGCGTTCGAAAACAGTATATACGACCAG
GAATACAATATCCGTGTAACCATGTATGTACAATTCAGAGAATTTAATCTTAAAGACCCCCCACTTAACCCC--------

>JF317578_pcv2b

ATGACGTATCCAAGGAGGCGTTACCGGAGAAGAAGACACCGCCCCCGCAGCCATCTTGGCCAGATCCTCCGCCGCCG
CCCCTGGCTCGTCCACCCCCGCCACCGTTACCGCTGGAGAAGGAAAAATGGCATCTTCAACACCCGCCTCTCCCGCACC
TTCGGATATACTATAAAGCGAACCACAGTCAAAACGCCCTCGTGGGCGGTGGACATGATGAGATTCAATATTAATGAC
TTTCTTCCCCCAGGAGGGGGCTCAAACCCCCGCTCTGTGCCCTTTGAATACTACAGAATAAGAAAGGTTAAGGTTGAA
TTCTGGCCCTGCTCCCCGATCACCCAGGGTGACAGGGGAGTGGGCTCCAGTGCTGTTATTCTAGATGATAACTTTGTA
ACAAAGGCCACAGCCCTCACCTATGACCCCTATGTAAACTACTCCTCCCGCCATACCATAACCCAGCCCTTCTCCTACCA
CTCCCGCTACTTTACCCCCAAACCTGTCCTAGATTCCACTATTGATTACTTCCAACCAAACAACAAAAGAAATCAGCTGT
GGCTGAGACTACAAACTGCTGGAAATGTAGACCACGTAGGCCTCGGCACTGCGTTCGAAAACAGTATATACGACCAG
GAATACAATATCCGTGTAACCATGTATGTACAATTCAGAGAATTTAATCTTAAAGACCCCCCACTTAACCCC--------

>FJ667595_pcv2b

ATGACGTATCCAAGGAGGCGTTACCGGAGAAGAAGACACCGCCCCCGCAGCCATCTTGGCCAGATCCTCCGCCGCCG
CCCCTGGCTCGTCCACCCCCGCCACCGTTACCGCTGGAGAAGGAAAAATGGCATCTTCAACACCCGCCTCTCCCGCACC
TTCGGATATACTATCAAGCGAACCACAGTCAGAACGCCCTCCTGGGCGGTGGACATGATGAGATTCAATATTAATGAC
TTTCTTCCCCCAGGAGGGGGCTCAAACCCCCGCTCTGTGCCCTTTGAATACTACAGAATAAGAAAGGTTAAGGTTGAA
TTCTGGCCCTGCTCCCCGATCACCCAGGGTGACAGGGGAGTGGGCTCCAGTGCTGTTATTCTAGATGATAACTTTGTA
ACAAAGGCCACAGCCCTCACCTATGACCCCTATGTAAACTACTCCTCCCGCCATACCATAACCCAGCCCTTCTCCTACCA
CTCCCGCTACTTTACCCCCAAACCTGTCCTAGATTCCACTATTGATTACTTCCAACCAAACAACAAAAGAAATCAGCTGT
GGCTGAGACTACAAACTGCTGGAAATGTAGACCACGTAGGCCTCGGAACTGCCTTCGAAAACAGTATATACGACCAG
GAATACAATATCCGTGTAACCATGTATGTACAATTCAGAGAATTTAATCTTAAAGACCCCCCACTTAAACCTCAA-----

>FJ667596_pcv2b

ATGACGTATCCAAGGAGGCGTTACCGGAGAAGAAGACACCGCCCCCGCAGCCATCTTGGCCAGATCCTCCGCCGCCG
CCCCTGGCTCGTCCACCCCCGCCACCGTTACCGCTGGAGAAGGAAAAATGGCATCTTCAACACCCGCCTCTCCCGCACC
TTCGGATATACTATCAAGCGAACCACAGTCAGAACGCCCTCCTGGGCGGTGGACATGATGAGATTCAATATTAATGAC
TTTCTTCCCCCAGGAGGGGGCTCAAACCCCCGCTCTGTGTCCTTTGAATACTACAGAATAAGAAAGGTTAAGGTTGAA
TTCTGGCCCTGCTCCCCGATCACCCAGGGTGACAGGGGAGTGGGCTCCAGTGCTGTTATTCTAGATGATAACTTTGTA
ACAAAGGCCACAGCCCTCACCTATGACCCCTATGTAAACTACTCCTCCCGCCATACCATAACCCAGCCCTTCTCCTACCA
CTCCCGCTACTTTACCCCCAAACCTGTCCTAGATTCCACTATTGATTACTTCCAACCAAACAACAAAAGAAATCGGCTGT
GGCTGAGACTACAAACTGCTGGAAATGTAGACCACGTAGGCCTCGGAACTGCCTTCGAAAACAGTATATACGACCAG
GAATACAATATCCGTGTAACCATGTATGTACAATTCAGAGAATTTAATCTTAAAGACCCCCCACTTAAACCTCAA-----

>FJ667594_pcv2b

ATGACGTATCCAAGGAGGCGTTACCGGAGAAGAAGACACCGCCCCCGCAGCCATCTTGGCCAGATCCTCCGCCGCCG
CCCCTGGCTCGTCCACCCCCGCCACCGTTACCGCTGGAGAAGGAAAAATGGCATCTTCAACACCCGCCTCTCCCGCACC
TTCGGATATACTATCAAGCGAACCACAGTCAGAACGCCCTCCTGGGCGGTGGACATGATGAGATTCAATATTAATGAC
TTTCTTCCCCCAGGAGGGGGCTCAAACCCCCGCTCTGTGCCCTTTGAATACTACAGAATAAGAAAGGTTAAGGTTGAA
TTCTGGCCCTGCTCCCCGATCACCCAGGGTGACAGGGGAGTGGGCTCCAGTGCTGTTATTCTAGATGATAACTTTGTA
ACAAAGGCCACAGCCCTCACCTATGACCCCTATGTAAACTACTCCTCCCGCCATACCATAACCCAGCCCTTCTCCTACCA
CTCCCGCTACTTTACCCCCAAACCTGTCCTAGATTCCACTATTGATTACTTCCAACCAAACAACAAAAGAAATCAGCTGT
GGCTGAGACTACAAACTGCTGGAAATGTAGACCACGTAGGCCTCGGAACTGCCTTCGAAAACAGTATATACGACCAG
GAATACAATATCCGTGTAACCATGTATGTACAATTCAGAGAATTTAATCTTAAAGACCCCCCACTTAAACCTCAA-----

>FJ716703_pcv2b

ATGACGTATCCAAGGAGGCGTTACCGGAGAAGAAGACACCGCCCCCGCAGCCATCTTGGCCAGATCCTCCGCCGCCG
CCCCTGGCTCGTCCACCCCCGCCACCGTTACCGCTGGAGAAGGAAAAATGGCATCTTCAACACCCGCCTCTCCCGCACC
TTCGGATATACTATCAAGCGAACCACAGTCAGAACGCCCTCCTGGGCGGTGGACATGATGAGATTCAATATTAATGAC
TTTCTTCCCCCAGGAGGGGGCTCAAACCCCCGCTCTGTGCCCTTTGAATACTACAGAATAAGAAAGGTTAAGGTTGAA
TTCTGGCCCTGCTCCCCGATCACCCAGGGTGACAGGGGAGTGGGCTCCAGTGCTGTTATTCTAGATGATAACTTTGTA
ACAAAGGCCACAGCCCTCACCTATGACCCCTATGTAAACTACTCCTCCCGCCATACCATAACCCAGCCCTTCTCCTACCA
CTCCCGCTACTTTACCCCCAAACCTGTCCTAGATTCCACTATTGATTACTTCCAACCAAACAACAAAAGAAATCAGCTGT
GGCTGAGACTACAAACTGCTGGAAATGTAGACCACGTAGGCCTCGGAACTGCCTTCGAAAACAGTATATACGACCAG
GAATACAATATCCGTGTAACCATGTATGTACAATTCAGAGAATTTAATCTTAAAGACCCCCCACTTAAACCTCAA-----

>FJ716704_pcv2b

ATGACGTATCCAAGGAGGCGTTACCGGAGAAGAAGACACCGCCCCCGCAGCCATCTTGGCCAGATCCTCCGCCGCCG
CCCCTGGCTCGTCCACCCCCGCCACCGTTACCGCTGGAGAAGGAAAAATGGCATCTTCAACACCCGCCTCTCCCGCACC
TTCGGATATACTATCAAGCGAACCACAGTCAGAACGCCCTCCTGGGCGGTGGACATGATGAGATTCAATATTAATGAC
TTTCTTCCCCCAGGAGGGGGCTCAAACCCCCGCTCTGTGCCCTTTGAATACTACAGAATAAGAAAGGTTAAGGTTGAA
TTCTGGCCCTGCTCCCCGATCACCCAGGGTGACAGGGGAGTGGGCTCCAGTGCTGTTATTCTAGATGATAACTTTGTA
ACAAAGGCCACAGCCCTCACCTATGACCCCTATGTAAACTACTCCTCCCGCCATACCATAACCCAGCCCTTCTCCTACCA
CTCCCGCTACTTTACCCCCAAACCTGTCCTAGATTCCACTATTGATTACTTCCAACCAAACAACAAAAGAAATCAGCTGT
GGCTGAGACTACAAACTGCTGGAAATGTAGACCACGTAGGCCTCGGAACTGCCTTCGAAAACAGTATATACGACCAG
GAATACAATATCCGTGTAACCATGTATGTACAATTCAGAGAATTTAATCTTAAAGACCCCCCACTTAAACCTCAA-----

>HM038018_pcv2b

ATGACGTATCCAAGGAGGCGTCACCGGAGAAGAAGACACCGCCCCCGCAGCCATCTTGGCCAGATCCTCCGCCGCCG
CCCCTGGCTCGTCCACCCCCGCCACCGTTACCGCTGGAGAAGGAAAAATGGCATCTTCAACACCCGCCTCTCCCGCACC
TTCGGATATACTATCAAGCGAACCACAGTCAAAACGCCCTCCTGGGCGGTGGACATGATGAGATTCAATATTAATGAC
TTTCTTCCCCCAGGAGGGGGCTCAAACCCCCGCTCTGTGCCCTTTGAATACTACAGAATAAGAAAGGTTAAGGTTGAA
TTCTGGCCCTGCTCCCCGATCACCCAGGGTGACAGGGGAGTGGGCTCCAGTGCTGTTATTCTAGATGATAACTTTGTA
ACAAAGGCCACAGCCCTCACCTATGACCCCTATGTAAACTACTCCTCCCGCCATACCATAACCCAGCCCTTCTCCTACCA
CTCCCGCTACTTTACCCCCAAACCTGTCCTAGATTCCACTATTGATTACTTCCAACCAAACAACAAAAGAAATCAGCTGT
GGCTGAGACTACAAACTGCTGGAAATGTAGACCACGTAGGCCTCGGCACTGCCTTCGAAAACAGTATATACGACCAG
GAATACAATATCCGTGTAACCATGTATGTACAATTCAGAGAATTTAATCTTAAAGACCCCCCACTTAACCCT--------

>HQ591370_pcv2b

ATGACGTATCCAAGGAGGCGTCACCCGAGAAGAAGACACCGCCCCCGCAGCCATCTTGGCCAGATCCTCCGCCGCCG
CCCCAGGCTCGTCCACCCCCGCCACCGTTACCGCTGGAGAAGGAAAAATAGCATCTTCAACACCCGCCTCTCCCGCACC
TTCGGATATACCATCAAGCGAACCACAGTCAAAACGCCCTCCTGGGCGGTGGACATGATGAGATTCAATATTAATGAC
TTTCTTCCCCCAGGAGGGGGCTCAAACCCCCGCTCTGTGCCCTTTGAATACTACAGAATAAGAAAGGTTAAGGTTGAA
TTCTGGCCCTGCTCCCCGATCACCCAGGGTGACAGGGGAGTGGGCTCCAGTGCTGTTATTCTAGATGATAACTTTGTA
ACAAAGGCCACAGCCCTCACCTATGACCCCTATGTAAACTACTCCTCCCGCCATACCATAACCCAGCCCTTCTCCTACCA
CTCCCGCTACTTTACCCCCAAACCTGTCCTAGATTCCACTATTGATTACTTCCAACCAAACAACAAAAGAAATCAGCTGT
GGCTGAGACTACAAACTGCTGGAAATGTAGACCACGTAGGCCTCGGCACTGCGTTCGAAAACAGTATATACGACCAG
GAATACAATATCCGTGTAACCATGTATGTACAATTCAGAGAATTTAATCTTAAAGACCCCCCACTTAACCCT--------

>JQ866920_pcv2b

ATGACGTATCCAAGAGGCGTTACCGCAAGAAGAAGACACCGCCCCCGCAGCCATCTTGGCCAGATCCTCCGCCGCCG
CCCCTGGCTCGTCCACCCACGCCACCGTTACCGCTGGAGAAGGAAAAATGGCATCTTCAACACCCGCCTCTCCCGCACC
TTCGGATATACTATCAAGCGAACCACAGTCAGAACGCCCTCCTGGGCGGTGGACATGATGAGATTCAATATTAATGAC
TTTCTTCCCCCAGGAGGGGGCTCAAACCCCCGCTCTGTGCCCTTTGAATACTACAGAATAAGAAAGGTTAAGGTTGAA
TTCTGGCCCTGCTCCCCGATCACCCAGGGTGACAGGGGAGTGGGCTCCAGTGCTGTTATTCTAGATGATAACTTTGTA
ACAAAGGCCACAGCCCTCACCTATGACCCCTATGTAAACTACTCCTCCCGCCATACCATAACCCAGCCCTTCTCCTACCA
CTCCCGCTACTTTACCCCCAAACCTGTCCTAGATTCCACTATTGATTACTTCCAACCAAACAACAAAAGAAATCAGCTGT
GGCTGAGACTACAAACTGCTGGAAATGTATACCACGTAGGCCTCGGCACTGCGTTCGAAAACAGTATATACGACCAG
GAATACAATATCCGTGCAACCATGTATGTACAATTCAGAGAATTTAATCTTAAAGACCCCCCACTTAACCCT--------

>GQ227412_pcv2b

ATGACGTATCCAAGAAGGCGTTACCGGAGAAGAAGACACCGCCCCCGCAGCCATCTTGGCCAGATCCTCCGCCGCCG
CCCCTGGCTCGTCCACCCCCGCCACCGTTACCGCTGGAGAAGGAAAAATGGCATCTTCAACACCCGCCTCTCCCGCACC
TTCGGATATACTATCAAGCGAACCACAGTCAAAACGCCCTCCTGGGCGGTGGACATGATGAGATTCAATATTAATGAC
TTTCTTCCCCCAGGAGGGGGCTCAAACCCCCGCTCTGTGCCCTTTGAATACTACAGAATAAGAAAGGTTAAGGTTGAA
TTCTGGCCCTGCTCCCCGATCACCCAGGGTGACAGGGGAGTGGGCTCCAGTGCTGTTATTCTAGATGATAACTTTGTA
ACAAAGGCCACAGCCCTCACCTATGACCCCTATGTAAACTACTCCTCCCGCCATACCATAACCCAGCCCTTCTCCTACCA
CTCCCGCTACTTTACCCCCAAACCTGTCCTAGATTCCACTATTGATTACTTCCAACCAAACAACAAAAGAAATCAGCTGT
GGCTGAGACTACAAACTGCTGGAAATGTAGACCACGTAGGCCTCGGCACTGCGTTCGAAAACAGTATATACGACCAG
GAATACAATATCCGTGTAACCATGTATGTACAATTCAGAGAATTTAATCTTAAAGACCCCCCACTTAACCCT--------

>HM038024_pcv2b

ATGACGTATCCAAGGAGGCGTTACCGGAGAAGAAGACACCGCCCCCGCAGCCATCTTGGCCAGATCCTCCGCCGCCG
CCCCCGGCTCGTCCACCCCCGCCACCGTTACCGCTGGAGAAGGAAAAATGGCATCTTCAACACCCGCCTCTCCCGCAC
CTTCGGATATACTATCAAGCGAACCACAGTCAAAACGCCCTCCTGGGCGGTGGACATGATGAGATTCAATATTAATGA
CTTTCTTCCCCCAGGAGGGGGCTCAAACCCCCGCTCTGTGCCCTTTGAATACTACAGAATAAGAAAGGTTAAGGTTGA
ATTCTGGCCCTGCTCCCCGATCACCCAGGGTGACAGGGGAGTGGGCTCCAGTGCTGTTATTCTAGATGATAACTTTGT
AACAAAGGCCACAGCCCTCACCTATGACCCCTATGTAAACTACTCCTCCCGCCATACCATAACCCAGCCCTTCTCCTACC
ACTCCCGCTACTTTACCCCCAAACCTGTCCTAGATTCCACTATTGATTACTTCCAACCAAACAACAAAAGAAATCAGCTG
TGGCTGAGACTACAAACTGCTGGAAATGTAGACCACGTAGGCCTCGGCACTGCGTTCGAAAACAGTATATACGACCA
GGAATACAATATCCGTGTAACCATGTATGTACAATTCAGAGAATTTAATCTTAAAGACCCCCCACTTAACCCT--------

>HM038026_pcv2b

ATGACGTATCCAAGGAGGCGTTACCGGAGAAGAAGACACCGCCCCCGCAGCCATCTTGGCCAGATCCTCCGCCGCCG
CCCCCGGCTCGTCCACCCCCGCCACCGTTACCGCTGGAGAAGGAAAAATGGCATCTTCAACACCCGCCTCTCCCGCAC
CTTCGGATATACTATCAAGCGAACCACAGTCAAAACGCCCTCCTGGGCGGTGGACATGATGAGATTCAATATTAATGA
CTTTCTTCCCCCAGGAGGGGGCTCAAACCCCCGCTCTGTGCCCTTTGAATACTACAGAATAAGAAAGGTTAAGGTTGA
ATTCTGGCCCTGCTCCCCGATCACCCAGGGTGACAGGGGAGTGGGCTCCAGTGCTGTTATTCTAGATGATAACTTTGT
AACAAAGGCCACAGCCCTCACCTATGACCCCTATGTAAACTACTCCTCCCGCCATACCATAACCCAGCCCTTCTCCTACC
ACTCCCGCTACTTTACCCCCAAACCTGTCCTAGATTCCACTATTGATTACTTCCAACCAAACAACAAAAGAAATCAGCTG
TGGCTGAGACTACAAACTGCTGGAAATGTAGACCACGTAGGCCTCGGCACTGCGTTCGAAAACAGTATATACGACCA
GGAATACAATATCCGTGTAACCATGTATGTACAATTCAGAGAATTTAATCTTAAAGACCCCCCACTTAACCCT--------

>EF592576_pcv2b

ATGACGTATCCAAGGAGGCGTTACCGGAGAAGAAGACACCGCCCCCGCAGCCATCTTGGCCAGATCCTCCGCCGCCG
GCCCTGGCTCGTCCAGCCCCGCCACCGTTACCGCTGGAGATGGAAAATTGGCATCTTCAACACCCGCCTCTCCCGCACC
TTCGGATATACTATCAAGAGAACCACAGTCAAAACGCCCTCCTGGGCGGTGGACATGATGAGATTCAATATTAATGAC
TTTCTTCCCCCAGGAGGGGGCTCAAACCCCCGCTCTGTGCCCTTTGAATACTACAGAATAAGTAAGGTTAAGGTTGAA
TTCTGGCCCTGCTCCCCGATCACCCAGGGTGACAGGGGAGTGGGCTCCAGTGCTGTTATTCTAGATGATAACTTTGTA
ACAAAGGCCACAGCCCTCACCTATGACCCCTATGTAAACTACTCCTCCCGCCATACCATAACCCAGCCCTTCTCCTACCA
CTCCCGCTACTTTACCCCCAAACCTGTCCTAGATTCCACTATTGATTACTTCCAACCAAACAACAAAAGAAATCAGCTGT
GGCTGAGACTACAAACTGCTGGAAATGTAGACCACGTAGGCCTCGGCACTGCGTTCGAAAACAGTATATACGACCAG
GAATACAATATCCGTGTAACCATGTATGTGCAATTCAGAGAATTTAATCTTAAAGACCCCCCACTTAACCCT--------

>EF619037_pcv2b

ATGACGTATCCAAGGAGGCGTTACCGGAGTAGAAGACACCGCCCCCGCAGCCATCTTGGCCAGATCCTCCGCCGCCG
CGCGTGGCTCGTCCAGGGCCGCCACCGTTACCGCTGGAGAAGGTACATTGGCATCTTCAACACGCGGCTCTCCCGCAC
CTTCGGATATACTATCAAGCGAACCACAGTCAGAACGCCCTCCTGGGCGGTGGACATGATGAGATTCAATAATAATGA
CTTTCTTGGGGCAGGAGCCGGCTCTATCGCCCGCTCTGTGCGCTTTGTATACTACAGAATAAGTAAGGTTAAGGTTGA
ATTCTGGCCCTGCTGCCCGATCACCCAGGGTGACAGCGGAGTGGGCTCCAGTGCTGTTATTCTGGATGACAACTTTGT
AACAAAGGCCACAGCCCTCACCTATGAGCCCTATGTAAACTACTCCTCCCGCCATACCATAACCCAGCCCTTCTCCTACC
ACTCCCGCTACTTTAGCCCCAAACCTGTCCTAGATTCCACTATTGATTACTTCCAACCAAACAACAAAAGAAATCAGCTG
TGGCTGAGACTACAAATTGCTGGAAATGTAGACCACGTAGGCCTCGGCACTGCGTTCGAAAACAGTATATACGACTTG
GAATACAATATCCGTGTAACCATGTATGTGCAATTCAGAGAATTTAATCTTAAAGACCCCCCACTTAACCCT--------

>EF592575_pcv2b

ATGACGTATCCAAGGAGGCGTTACCGGAGAAGAAGACACCGCCCCCGCAGCCATCTTGGCCAGATCCTCCGCCGCCG
GCCCTGGCTCGTCCAGGCCCGCCACCGTTACCGCTGGAGAAGGAAAAATGGCATCTTCAACACCCGCCTCTCCCGCAC
CTTCGGATATACTATCAAGCGAACCACAGTCAAAACGCCCTCCTGGGCGGTGGACATGATGAGATTCAATATTAATGA
CATTCTTCCCCCAGGACGGGGCTCAAAGCCCCGCTCTGTGCCCTTTGAATACTACAGAATAAGAAAGGTTAAGGTTGA
ATTCTGGCCCTGCTCCCCGATCACCCAGGGTGACAGAGGAGTGGGCTCCAGTGCTGTTATTCTAGATGATAACTTTGT
AACAAAGGCCACAGCCCTCACCTATGACCCCTATGTAAACTACTCCTCCCGCCATACCATAACCCAGCCCTTCTCCTACC
ACTCCCGCTACTTTACCCCCAAACCTGTCCTAGATTCCACTATTGATTACTTCCAACCAAACAACAAAAGAAATCAGCTG
TGGCTGAGACTACAAACTGCTGGAAATGTAGACCACGTAGGCCTCGGCACTGCGTTCGAAAACAGTATATACGACCA
AGAATACAATATCCGTGTAACCATGTATGTACAATTCAGAGAATTTAATCTTAAAGACCCCCCACTTAACCCT--------

>EF565359_pcv2b

ATGACGTATCCAAGGAGGCGTTACCGGAGAAGAAGACACCGCCCCCGCAGCCATCTTGGCCAGATCCTCCGCCGCCG
CCCCTGGCTCGTCCACCCCCGCCACCGTTACCGCTGGAGAAGGAAAAATGGCATCTTCAACACCCGCCTCTCCCGCACC
TTCGGATATACTATCAAGCGAACCACAGTCAAAACGCCCTCCTGGGCGGTGGACATGATGAGATTCAATATTAATGAC
TTTCTTCCCCCAGGAGGGGGCTCAAACCCCCGCTCTGTGCCCTTTGAATACTACAGAATAAGAAAGGTTAAGGTTGAA
TTCTGGCCCTGCTCCCCGATCACCCAGGGTGACAGGGGAGTGGGCTCCAGTGCTGTTATTCTAGATGATAACTTTGTA
ACAAAGGCCACAGCCCTCACCTATGACCCCTATGTAAACTACTCCTCCCGCCATACCATAACCCAGCCCTTCTCCTACCA
CTCCCGCTACTTTACCCCCAAACCTGTCCTAGATTCCACTATTGATTACTTCCAACCAAACAACAAAAGAAATCAGCTGT
GGCTGAGACTACAAACTGCTGGAAATGTAGACCACGTAGGCCTCGGCACTGCATTCGAAAACAGTATATACGACCAA
GAATACAATATCCGTGTAACCATGTATGTACAATTCAGAGAATTTAATCTTAAAGACCCCCCACTTAACCCT--------

>EF565352_pcv2b

ATGACGTATCCAAGGAGGCGTTACCGGAGAAGAAGACACCGCCCCCGCAGCCATCTTGGCCAGATCCTCCGCCGCCG
CCCCTGGCTCGTCCACCCCCGCCACCGTTACCGCTGGAGAAGGAAAAATGGCATCTTCAACACCCGCCTCTCCCGCACC
TTCGGATATACTATCAAGCGAACCACAGTCAAAACGCCCTCCTGGGCGGTGGACATGATGAGATTCAATATTAATGAC
TTTCTTCCCCCAGGAGGGGGCTCAAACCCCCGCTCTGTGCCCTTTGAATACTACAGAATAAGAAAGGTTAAGGTTGAA
TTCTGGCCCTGCTCCCCGATCACCCAGGGTGACAGGGGAGTGGGCTCCAGTGCTGTTATTCTAGATGATAACTTTGTA
ACAAAGGCCACAGCCCTCACCTATGACCCCTATGTAAACTACTCCTCCCGCCATACCATAACCCAGCCCTTCTCCTACCA
CTCCCGCTACTTTACCCCCAAACCTGTCCTAGATTCCACTATTGATTACTTCCAACCAAACAACAAAAGAAATCAGCTGT
GGCTGAGACTACAAACTGCTGGAAATGTAGACCACGTAGGCCTCGGCACTGCGTTCGAAAACAGTATATACGACCAA
GAATACAATATCCGTGTAACCATGTATGTACAATTCAGAGAATTTAATCTTAAAGACCCCCCACTTAACCCT--------

>HQ831526_pcv2b

ATGACGTATCCAAGGAGGCGTTACCGGAGAAGAAGACACCGCCCCCGCAGCCATCTTAGCCAGATCCTCCGCCGCCG
CCCCTGGCTCGTCCACCCCCGCCACCGTTACCGCTGGAGAAGGAAAAATGGCATCTTCAACACCCGCCTCTCCCGCACC
TTCGGATATACTATCAAGCGAACCACAGTCAAAACGCCCTCCTGGGCGGTGGACATGATGAGATTCAATATTAATGAC
TTTCTTCCCCCAGGAGGGGGCTCAAACCCCCGCTCTGTGCCCTTTGAATACTACAGAATAAGAAAGGTTAAGGTTGAA
TTCTGGCCCTGCTCCCCGATCACCCAGGGTGACAGGGGAGTGGGCTCCAGTGCTGTTATTCTAGATGATAACTTTGTA
ACAAAGGCCACAGCCCTCACCTATGACCCCTATGTAAACTACTCCTCCCGCCATACCATAACCCAGCCCTTCTCCTACCA
CTCCCGCTACTTTACCCCCAAACCTGTCCTAGATTCCACTATTGATTACTTCCAACCAAACAACAAAAGAAATCAGCTGT
GGCTGAGACTACAAACTGCTGGAAATGTAGACCACGTAGGCCTCGGCACTGCATTCGAAAACAGTATATACGACCAG
GAATACAATATCCGTGTAACCATGTATGTACAATTCAGAGAATTTAATCTTAAAGACCCCCCACTTAACCCT--------

>AY321992_pcv2b

ATGACGTATCCAAGGAGGCGTTACCGGAGAAGAAGACACCGCCCCCGCAGCCATCTTGGCCAGATCCTCCGCCGCCG
CCCCTGGCTCGTCCACCCCCGCCACCGTTACCGCTGGAGAAGGAAAAATGGCATCTTCAACACCCGCCTCTCCCGCACC
TTCGGATATACTATCAAGCGAACCACAGTCAAAACGCCCTCCTGGGCGGTGGACATGATGAGATTCAATATTAATGAC
TTTCTTCCCCCAGGAGGGGGCTCAAACCCCCGCTCTGTGCCCTTTGAATACTACAGAATAAGAAAGGTTAAGGTTGAA
TTCTGGCCCTGCTCCCCGATCACCCAGGGTGACAGGGGAGTGGGCTCCAGTGCTGTTATTCTAGATGATAACTTTGTA
ACAAAGGCCACAGCCCTCACCTATGACCCCTATGTAAACTACTCCTCCCGCCATACCATAACCCAGCCCTTCTCCTACCA
CTCCCGCTACTTTACCCCCAAACCTGTCCTAGATTCCACTATTGATTACTTCCAACCAAACAACAAAAGAAATCAGCTGT
GGCTGAGACTACAAACTGCTGGAAATGTAGACCACGTAGGCCTCGGCACTGCGTTCGAAAACAGTATATACGACCAG
GAATACAATATCCGTGTAACCATGTATGTTCAATTCAGAGAATTTAATCTTAAAGACCCCCCACTTAACCCT--------

>JX512860_pcv2b

ATGACGTATCCAAGGAGGCGTTACCGGAGAAGAAGACACCGCCCCCGCAGCCATCTTGGCCAGATCCTCCGCCGCCG
CCCCTGGCTCGTCCACCCCCGCCACCGTTACCGCTGGAGAAGGAAAAATGGCATCTTCAACACCCGCCTCTCCCGCACC
TTCGGATATACTATCAAGCGAACCACAGTCAAAACGCCCTCCTGGGCGGTGGACATGATGAGATTCAATATTAATGAC
TTTCTTCCCCCAGGAGGGGGCTCAAACCCCCGCTCTGTGCCCTTTGAATACTACAGAATAAGAAAGGTTAAGGTTGAA
TTCTGGCCCTGCTCCCCGATCACCCAGGGTGACAGGGGAGTGGGCTCCAGTGCTGTTATTCTAGATGATAACTTTGTA
ACAAAGGCCACAGCCCTCACCTATGACCCCTATGTAAACTACTCCTCCCGCCATACCATAACCCAGCCCTTCTCCTACCA
CTCCCGCTACTTTACCCCCAAACCTGTCCTAGATTCCACTATTGATTACTTCCAACCAAACAACAAAAGAAATCAGCTGT
GGCTGAGACTACAAACTGCTGGAAATGTAGACCACGTAGGCCTCGGCACTGCGTTCGAAAACAGTATATACGACCAG
GAATACAATATCCGTGTAACCATGTATGTACAATTCAGAGAATTTAATCTTAAAGACCCCCCACTTAACCCT--------

>AB361584_pcv2b

ATGACGTATCCAAGGAGGCGTTACCGGAGAAGAAGACACCGCCCCCGCAGCCATCTTGGCCAGATCCTCCGCCGCCG
CCCCTGGCTCGTCCACCCCCGCCACCGTTACCGCTGGAGAAGGAAAAATGGCATCTTCAACACCCGCCTCTCCCGCACC
TTCGGATATACTATCAAGCGAACCACAGTCAAAACGCCCTCCTGGGCGGTGGACATGATGAGATTCAATATTAATGAC
TTTCTTCCCCCAGGAGGGGGCTCAAACCCCCGCTCTGTGCCCTTTGAATACTACAGAATAAGAAAGGTTAAGGTTGAA
TTCTGGCCCTGCTCCCCGATCACCCAGGGTGACAGGGGAGTGGGCTCCAGTGCTGTTATTCTAGATGATAACTTTGTA
ACAAAGGCCACAGCCCTCACCTATGACCCCTATGTAAACTACTCCTCCCGCCATACCATAACCCAGCCCTTCTCCTACCA
CTCCCGCTACTTTACCCCCAAACCTGTCCTAGATTCCACTATTGATTACTTCCAACCAAACAACAAAAGAAATCAGCTGT
GGCTGAGACTACAAACTGCTGGAAATGTAGACCACGTAGGCCTCGGCACTGCGTTCGAAAACAGTATATACGACCAG
GAATACAATATCCGTGTAACCATGTATGTACAATTCAGAGAATTTAATCTTAAAGACCCCCCACTTAACCCT--------

>AY291316_pcv2b

ATGACGTATCCAAGGAGGCGTTACCGGAGAAGAAGACACCGCCCCCGCAGCCATCTTGGCCAGATCCTCCGCCGCCG
CCCCTGGCTCGTCCACCCCCGCCACCGTTACCGCTGGAGAAGGAAAAATGGCATCTTCAACACCCGCCTCTCCCGCACC
TTCGGATATACTATCAAGCGAACCACAGTCAAAACGCCCTCCTGGGCGGTGGACATGATGAGATTCAATATTAATGAC
TTTCTTCCCCCAGGAGGGGGCTCAAACCCCCGCTCTGTGCCCTTTGAATACTACAGAATAAGAAAGGTTAAGGTTGAA
TTCTGGCCCTGCTCCCCGATCACCCAGGGTGACAGGGGAGTGGGCTCCAGTGCTGTTATTCTAGATGATAACTTTGTA
ACAAAGGCCACAGCCCTCACCTATGACCCCTATGTAAACTACTCCTCCCGCCATACCATAACCCAGCCCTTCTCCTACCA
CTCCCGCTACTTTACCCCCAAACCTGTCCTAGATTCCACTATTGATTACTTCCAACCAAACAACAAAAGAAATCAGCTGT
GGCTGAGACTACAAACTGCGGGAAATGTAGACCACGTAGGCCTCGGCACTGCGTTCGAAAACAGTATATACGACCAG
GAATACAATATCCGTGTAACCATGTATGTACAATTCAGAGAATTTAATCTTAAAGACCCCCCACTTAACCCT--------

>JN382176_pcv2b

ATGACGTATCCAAGGAGGCGTTACCGGAGAAGAAGACACCGCCCCCGCAGCCATCTTGGCCAGATCCTCCGCCGCCG
CCCCTGGCTCGTCCACCCCCGCCACCGTTACCGCTGGAGAAGGAAAAATGGCATCTTCAACACCCGCCTCTCCCGCACC
TTCGGATATACTATCAAGCGAACCACAGTCAAAACGCCCTCCTGGGCGGTGGACATGATGAGATTCAATATTAATGAC
TTTCTTCCCCCAGGAGGGGGCTCAAACCCCCGCTCTGTGCCCTTTGAATACTACAGAATAAGAAAGGTTAAGGTTGAA
TTCTGGCCCTGCTCCCCGATCACCCAGGGTGACAGAGGAGTGGGCTCCAGTGCTGTTATTCTAGATGATAACTTTGTA
ACAAAGGCCACAGCCCTCACCTATGACCCCTATGTAAACTACTCCTCCCGCCATACCATAACCCAGCCCTTCTCCTACCA
CTCCCGCTACTTTACCCCCAAACCTGTCCTAGATTCCACTATTGATTACTTCCAACCAAACAACAAAAGAAATCAGCTGT
GGCTGAGACTACAAACTGCGGGAAATGTAGACCACGTAGGCCTCGGCACTGCGTTCGAAAACAGTATATACGACCAG
GAATACAATATCCGTGTAACCATGTATGTACAATTCAGAGAATTTAATCTTAAAGACCCCCCACTTAACCCT--------

>EF565356_pcv2b

ATGACGTATCCAAGGAGGCGTTACCGGAGAAGAAGACACCGCCCCCGCAGCCATCTTGGCCAGATCCTCCGCCGCCG
CCCCTGGCTCGTCCACCCCCGCCACCGTTACCGCTGGAGAAGGAAAAATGGCATCTTCAACACCCGCCTCTCCCGCACC
TTCGGATATACTATCAAGCGAACCACAGTCAAAACGCCCTCCTGGGCGGTGGACATGATGAGATTCAATATTAATGAC
TTTCTTCCCCCAGGAGGGGGCTCAAACCCCCGCTCTGTGCCCTTTGAATACTACAGAATAAGAAAGGTTAAGGTTGAA
TTCTGGCCCTGCTCCCCGATCACCCAGGGTGACAGGGGAGTGGGCTCCAGTGCTGTTATTCTAGATGATAACTTTGTA
ACAAAGGCCACAGCCCTCACCTATGACCCCTATGTAAACTACTCCTCCCGCCATACCATAACCCAGCCCTTCTCCTACCA
CTCCCGCTACTTTACCCCCAAACCTGTCCTAGATTCCACTATTGATTACTTCCAACCAAACAACAAAAGAAATCAGCTGT
GGCTGAGACTACAAACTGCGGGAAATGTAGACCACGTAGGCCTCGGCACTGCGTTCGAAAACAGTATATACGACCAG
GAATACAATATCCGTGTAACCATGTATGTACAATTCAGAGAATTTAATCTTAAAGACCCCCCACTTAACCCT--------

>EF565367_pcv2b

ATGACGTATCCAAGGAGGCGTTACCGGAGAAGAAGACACCGCCCCCGCAGCCATCTTGGCCAGATCCTCCGCCGCCG
CCCCTGGCTCGTCCACCCCCGCCACCGTTACCGCTGGAGAAGGAAAAATGGCATCTTCAACACCCGCCTCTCCCGCACC
TTCGGATATACTATCAAGCGAACCACAGTCAAAACGCCCTCCTGGGCGGTGGACATGATGAGATTCAATATTAATGAC
TTTCTTCCCCCAGGAGGGGGCTCAAACCCCCGCTCTGTGCCCTTTGAATACTACAGAATAAGAAAGGTTAAGGTTGAA
TTCTGGCCCTGCTCCCCGATCACCCAGGGTGACAGGGGAGTGGGCTCCAGTGCTGTTATTCTAGATGATAACTTTGTA
ACAAAGGCCACAGCCCTCACCTATGACCCCTATGTAAACTACTCCTCCCGCCATACCATAACCCAGCCCTTCTCCTACCA
CTCCCGCTACTTTACCCCCAAACCTGTCCTAGATTCCACTATTGATTACTTCCAACCAAACAACAAAAGAAATCAGCTGT
GGCTGAGACTACAAACTGCGGGAAATGTAGACCACGTAGGCCTCGGCACTGCGTTCGAAAACAGTATATACGACCAG
GAATACAATATCCGTGTAACCATGTATGTACAATTCAGAGAATTTAATCTTAAAGACCCCCCACTTAACCCT--------

>AY691169_pcv2b

ATGACGTATCCAAGGAGGCGTTACCGGAGAAGAAGACACCGCCCCCGCAGCCATCTTGGCCAGATCCTCCGCCGCCG
CCCCTGGCTCGTCCACCCCCGCCACCGTTACCGCTGGAGAAGGAAAAATGGCATCTTCAACACCCGCCTCTCCCGCACC
TTCGGATATACTATCAAGCGAACCACAGTCAAAACGCCCTCCTGGGCGGTGGACATGATGAGATTCAATATTAATGAC
TTTCTTCCCCCAGGAGGGGGCTCAAACCCCCGCTCTGTGCCCTTTGAATACTACAGAATAAGAAAGGTTAAGGTTGAA
TTCTGGCCCTGCTCCCCGATCACCCAGGGTGACAGGGGAGTGGGCTCCAGTGCTGTTATTCTAGATGATAACTTTGTA
ACAAAGGCCACAGCCCTCACCTATGACCCCTATGTAAACTACTCCTCCCGCCATACCATAACCCAGCCCTTCTCCTACCA
CTCCCGCTACTTTACCCCCAAACCTGTCCTAGATTCCACTATTGATTACTTCCAACCAAACAACAAAAGAAATCAGCTGT
GGCTGAGACTACAAACTGCTGGAAATGTAGACCACGTAGGCCTCGGCACTGCGTTCGAAAACAGTATATACGACCAG
GAATACAATATCCGTGTAACCATGTATGTACAATTCAGAGAATTTAATCTTAAAGACCCCCCACTTAACCCT--------

>JN382180_pcv2b

ATGACGTATCCAAGGAGGCGTTACCGGAGAAGAAGACACCGCCCCCGCAGCCATCTTGGCCAGATCCTCCGCCGCCG
CCCCTGGCTCGTCCACCCCCGCCACCGTTACCGCTGGAGAAGGAAAAATGGCATCTTCAACACCCGCCTCTCCCGCACC
TTCGGATATACTATCAAGCGAACCACAGTCAAAACGCCCTCCTGGGCGGTGGACATGATGAGATTCAATATTAATGAC
TTTCTTCCCCCAGGAGGGGGCTCAAACCCCCGCTCTGTGCCCTTTGAATACTACAGAATAAGAAAGGTTAAGGTTGAA
TTCTGGCCCTGCTCCCCGATCACCCAGGGTGACAGGGGAGTGGGCTCCAGTGCTGTTATTCTAGATGATAACTTTGTA
ACAAAGGCCACAGCCCTCACCTATGACCCCTATGTAAACTACTCCTCCCGCCATACCATAACCCAGCCCTTCTCCTACCA
CTCCCGCTACTTTACCCCCAAACCTGTCCTAGATTCCACTATTGATTACTTCCAACCAAACAACAAAAGAAATCAGCTGT
GGCTGAGACTACAAACTGCTGGAAATGTAGACCACGTAGGCCTCGGCACTGCGTTCGAAAACAGTATATACGACCAG
GAATACAATATCCGTGTAACCATGTATGTACAATTCAGAGAATTTAATCTTAAAGACCCCCCACTTAACCCT--------

>FN687850_pcv2b

ATGACGTATCCAAGGAGGCGTTACCGGAGAAGAAGACACCGCCCCCGCAGCCATCTTGGCCAGATCCTCCGCCGCCG
CCCCTGGCTCGTCCACCCCCGCCACCGTTACCGCTGGAGAAGGAAAAATGGCATCTTCAACACCCGCCTATCCCGCACC
TTCGGATATACTATCAAGCGAACCACAGTCAAAACGCCCTCCTGGGCAGTGGACATGATGAGATTCAATATTAATGAC
TTTCTTCCCCCAGGAGGGGGCTCAAACCCCCGCTCTGTGCCCTTTGAATACTACAGAATAAGAAAGGTTAAGGTTGAA
TTCTGGCCCTGCTCCCCGATCACCCAGGGTGACAGGGGAGTGGGCTCCAGTGCTGTTATTCTAGATGATAACTTTGTA
ACAAAGGCCACAGCCCTCACCTATGACCCCTATGTAAACTACTCCTCCCGCCATACCATAACCCAGCCCTTCTCCTACCA
CTCCCGCTACTTTACCCCCAAACCTGTCCTAGATTCCACTATTGATTACTTCCAACCAAACAACAAAAGAAACCAACTGT
GGCTGAGACTACAAACTGCTGGAAATGTAGACCACGTAGGCCTCGGCACTGCATTCGAAAACAGTATATACGACCAG
GAATACAATATCCGCGTAACCATGTATGTACAATTCAGAGAATTTAATCTTAAAGACCCCCCACTTAACCCT--------

>FN687851_pcv2b

ATGACGTATCCAAGGAGGCGTTACCGGAGAAGAAGACACCGCCCCCGCAGCCATCTTGGCCAGATCCTCCGCCGCCG
CCCCTGGCTCGTCCACCCCCGCCACCGTTACCGCTGGAGAAGGAAAAATGGCATCTTCAACACCCGCCTATCCCGCACC
TTCGGATATACTATCAAGCGAACCACAGTCAAAACGCCCTCCTGGGCAGTGGACATGATGAGATTCAATATTAATGAC
TTTCTTCCCCCAGGAGGGGGCTCAAACCCCCGCTCTGTGCCCTTTGAATACTACAGAATAAGAAAGGTTAAGGTTGAA
TTCTGGCCCTGCTCCCCGATCACCCAGGGTGACAGGGGAGTGGGCTCCAGTGCTGTTATTCTAGATGATAACTTTGTA
ACAAAGGCCACAGCCCTCACCTATGACCCCTATGTAAACTACTCCTCCCGCCATACCATAACCCAGCCCTTCTCCTACCA
CTCCCGCTACTTTACCCCCAAACCTGTCCTAGATTCCACTATTGATTACTTCCAACCAAACAACAAAAGAAACCAACTGT
GGCTGAGACTACAAACTGCTGGAAATGTAGACCACGTAGGCCTCGGCACTGCATTCGAAAACAGTATATACGACCAG
GAATACAATATCCGCGTAACCATGTATGTACAATTCAGAGAATTTAATCTTAAAGACCCCCCACTTAACCCT--------

>FN398025_pcv2b

ATGACGTATCCAAGGAGGCGTTACCGGAGAAGAAGACACCGCCCCCGCAGCCATCTTGGCCAGATCCTCCGCCGCCG
CCCCTGGCTCGTCCACCCCCGCCACCGTTACCGCTGGAGAAGGAAAAATGGCATCTTCAACACCCGCCTATCCCGCACC
TTCGGATATACTATCAAGCGAACCACAGTCAAAACGCCCTCCTGGGCAGTGGACATGATGAGATTCAATATTAATGAC
TTTCTTCCCCCAGGAGGGGGCTCAAACCCCCGCTCTGTGCCCTTTGAATACTACAGAATAAGAAAGGTTAAGGTTGAA
TTCTGGCCCTGCTCCCCGATCACCCAGGGTGACAGGGGAGTGGGCTCCAGTGCTGTTATTCTAGATGATAACTTTGTA
ACAAAGGCCACAGCCCTCACCTATGACCCCTATGTAAACTACTCCTCCCGCCATACCATAACCCAGCCCTTCTCCTACCA
CTCCCGCTACTTTACCCCCAAACCTGTCCTAGATTCCACTATTGATTACTTCCAACCAAACAACAAAAGAAACCAACTGT
GGCTGAGACTACAAACTGCTGGAAATGTAGACCACGTAGGCCTCGGCACTGCATTCGAAAACAGTATATACGACCAG
GAATACAATATCCGCGTAACCATGTATGTACAATTCAGAGAATTTAATCTTAAAGACCCCCCACTTAACCCT--------

>FN398023_pcv2b

ATGACGTATCCAAGGAGGCGTTACCGGAGAAGAAGACACCGCCCCCGCAGCCATCTTGGCCAGATCCTCCGCCGCCG
CCCCTGGCTCGTCCACCCCCGCCACCGTTACCGCTGGAGAAGGAAAAATGGCATCTTCAACACCCGCCTATCCCGCACC
TTCGGATATACTATCAAGCGAACCACAGTCAAAACGCCCTCCTGGGCAGTGGACATGATGAGATTCAATATTAATGAC
TTTCTTCCCCCAGGAGGGGGCTCAAACCCCCGCTCTGTGCCCTTTGAATACTACAGAATAAGAAAGGTTAAGGTTGAA
TTCTGGCCCTGCTCCCCGATCACCCAGGGTGACAGGGGAGTGGGCTCCAGTGCTGTTATTCTAGATGATAACTTTGTA
ACAAAGGCCACAGCCCTCACCTATGACCCCTATGTAAACTACTCCTCCCGCCATACCATAACCCAGCCCTTCTCCTACCA
CTCCCGCTACTTTACCCCCAAACCTGTCCTAGATTCCACTATTGATTACTTCCAACCAAACAACAAAAGAAACCAACTGT
GGCTGAGACTACAAACTGCTGGAAATGTAGACCACGTAGGCCTCGGCACTGCATTCGAAAACAGTATATACGACCAG
GAATACAATATCCGCGTAACCATGTATGTACAATTCAGAGAATTTAATCTTAAAGACCCCCCACTTAACCCT--------

>HQ738641_pcv2b

ATGACGTATCCAAGGAGGCGTTACCGGAGAAGAAGACACCGCCCCCGCAGCCATCTTGGCCAGATCCTCCGCCGCCG
CCCCTGGCTCGTCCACCCCCGCCACCGTTACCGCTGGAGAAGGAAAAATGGCATCTTCAACACCCGCCTATCCCGCACC
TTCGGATATACTATCAAGCGAACCACAGTCAAAACGCCCTCCTGGGCGGTGGACATGATGAGATTCAATATTAATGAC
TTTCTTCCCCCAGGAGGGGGCTCAAACCCCCGCTCTGTGCCCTTTGAATACTACAGAATAAGAAAGGTTAAGGTTGAA
TTCTGGCCCTGCTCCCCGATCACCCAGGGTGACAGGGGAGTGGGCTCCAGTGCTGTTATTCTAGATGATAACTTTGTA
ACAAAGGCCACAGCCCTCACCTATGACCCCTATGTAAACTACTCCTCCCGCCATACCATAACCCAGCCCTTCTCCTACCA
CTCCCGCTACTTTACCCCCAAACCTGTCCTAGATTCCACTATTGATTACTTCCAACCAAACAACAAAAGAAACCAGCTGT
GGCTGAGACTACAAACTGCTGGAAATGTAGACCACGTAGGCCTCGGCACTGCGTTCGAAAACAGTATATACGACCAG
GAATACAATATCCGTGTAACCATGTATGTACAATTCAGAGAATTTAATCTTAAAGACCCCCCACTTAACCCT--------

>HQ738640_pcv2b

ATGACGTATCCAAGGAGGCGTTACCGGAGAAGAAGACACCGCCCCCGCAGCCATCTTGGCCAGATCCTCCGCCGCCG
CCCCTGGCTAGTCCACCCCCGCCACCGTTACCGCTGGAGAAGGAAAAATGGCATCTTCAACACCCGCCTATCCCGCAC
CTTCGGATATACTATCAAGCGAACCACAGTCAAAACGCCCTCCTGGGCGGTGGACATGATGAGATTCAATATTAATGA
CTTTCTTCCCCCAGGAGGGGGCTCAAACCCCCGCTCTGTGCCCTTTGAATACTACAGAATAAGAAAGGTTAAGGTTGA
ATTCTGGCCCTGCTCCCCGATCACCCAGGGTAACAGGGGAGTGGGCTCCAGTGCTGTTATTCTAGATGATAACTTTGT
AACAAAGGCCACAGCCCTCACCTATGACCCCTATGTAAACTACTCCTCCCGCCATACCATAACCCAGCCCTTCTCCTACC
ACTCCCGCTACTTTACCCCCAAACCTGTCCTAGATTCCACTATTGATTACTTCCAACCAAACAACAAAAGAAACCAGCTG
TGGCTGAGACTACAAACTGCTGGAAATGTAGACCACGTAGGCCTCGGCACTGCGTTCGAAAACAGTATATACGACCA
GGAATACAATATCCGTGTAACCATGTATGTACAATTCAGAGAATTTAATCTTAAAGACCCCCCACTTAACCCT--------

>HQ738639_pcv2b

ATGACGTATCCAAGGAGGCGTTACCGGAGAAGAAGACACCGCCCCCGCAGCCATCTTGGCCAGATCCTCCGCCGCCG
CCCCTGGCTAGTCCACCCCCGCCACCGTTACCGCTGGAGAAGGAAAAATGGCATCTTCAACACCCGCCTATCCCGCAC
CTTCGGATATACTATCAAGCGAACCACAGTCAAAACGCCCTCCTGGGCGGTGGACATGATGAGATTCAATATTAATGA
CTTTCTTCCCCCAGGAGGGGGCTCAAACCCCCGCTCTGTGCCCTTTGAATACTACAGAATAAGAAAGGTTAAGGTTGA
ATTCTGGCCCTGCTCCCCGATCACCCAGGGTGACAGGGGAGTGGGCTCCAGTGCTGTTATTCTAGATGATAACTTTGT
AACAAAGGCCACAGCCCTCACCTATGACCCCTATGTAAACTACTCCTCCCGCCATACCATAACCCAGCCCTTCTCCTACC
ACTCCCGCTACTTTACCCCCAAACCTGTCCTAGATTCCACTATTGATTACTTCCAACCAAACAACAAAAGAAACCAGCTG
TGGCTGAGACTACAAACTGCTGGAAATGTAGACCACGTAGGCCTCGGCACTGCGTTCGAAAACAGTATATACGACCA
GGAATACAATATCCGTGTAACCATGTATGTACAATTCAGAGAATTTAATCTTAAAGACCCCCCACTTAACCCT--------

>JF317565_pcv2b

ATGACGTATCCAAGGAGGCGTTACCGGAGAAGAAGACACCGCCCCCGCAGCCATCTTGGCCAGATCCTCCGCCGCCG
CCCCTGGCTCCTCCACCCCCGCCACCGTTACCGCTGGAGAAGGAAAAATGGCATCTTCAACACCCGCCTATCCCGCACC
TTCGGATATACTATCAAGCGAACCACAGTCAGAACGCCCTCCTGGGCGGTGGACATGATGAGATTCAATATTAATGAC
TTTCTTCCCCCAGGAGGGGGCTCAAACCCCCGCTCTGTGCCCTTTGAATACTACAGAATAAGAAAGGTTAAGGTTGAA
TTCTGGCCCTGCTCCCCGATCACCCAGGGTGACAGGGGAGTGGGATCCACTGCTGTTATTCTAGATGATAACTTTGTA
ACAAAGGCCACAGCCCTCACCTATGACCCCTATGTAAACTACTCCTCCCGCCATACCATAACCCAGCCCTTCTCCTACCA
CTCCCGCTACTTTACCCCCAAACCTGTCCTAGATTCCACTATTGATTACTTCCAACCAAACAACAAAAGAAACCAGCTGT
GGCTGAGACTACAAACTGCTGGAAATGTAAACCACGTAGGCCTCGGCACTGCGTTCGAAAACAGTATATACGACCAG
GAATACAATATCCGTGTAACCATGTATGTACAATTCAGAGAATTTAATCTTAAAGACCCCCCACTTAACCCT--------

>EU518247_pcv2b

ATGACGTATCCAAGGAGGCGTTACCGGAGAAGAAGACACCGCCCCCGCAGCCATCTAGGCCAGATCCTCCGCCGCCG
CCCCTGGCTCGTCCACCCCCGCCACCGTTACCGCTGGAGAAGGAAAAATGGCATCTTCAACACCCGCCTATCCCGCACC
TTCGGATATACTATCAAGCGAACCACAGTCAGAACGCCCTCCTGGGCGGTGGACATGATGAGATTCAATATTAATGAC
TTTCTTCCCCCAGGAGGGGGCTCAAACCCCCGCTCTGTGCCCTTTGAATACTACAGAATAAGAAAGGTTAAGGTTGAA
TTCTGGCCCTGCTCCCCGATCACCCAGGGTGACAGGGGAGTGGGCTCCAGTGCTGTTATTCTAGATGATAACTTTGTA
ACAAAGGCCACAGCCCTCACCTATGACCCCTATGTAAACTACTCCTCCCGCCATACCATAACCCAGCCCTTCTCCTACCA
CTCCCGCTACTTTACCCCCAAACCTGTCCTAGATTCCACTATTGATTACTTCCAACCAAACAACAAAAGAAACCAGCTGT
GGCTGAGACTACAAACTGCTGGAAATGTAGACCACGTAGGCCTCGGCACTGCGTTCGAAAACAGTATATACGACCAG
GAATACAATATCCGTGTAACCATGTATGTACAATTCAGAGAATTTAATCTTAAAGACCCCCCACTTAACCCT--------

>JN382165_pcv2b

ATGACGTATCCAAGGAGGCGTTACCGGAGAAGAAGACACCGCCCCCGCAGCCATCTTGGCCAGATCCTCCGCCGCCG
CCCCTGGCTCGTCCACCCCCGCCACCGTTACCGCTGGAGAAGGAAAAATGGCATCTTCAACACCCGCCTCTCCCGCACC
TTCGGATATACTATCAAGCGAACCACAGTCAAAACGCCCTCCTGGGCGGTGGACATGATGAGATTCAATATTAATGAC
TTTCTTCCCCCAGGAGGGGGCTCAAACCCCCGCTCTGTGCCCTTTGAATACTACAGAATAAGAAAGGTTAAGGTTGAA
TTCTGGCCCTGCTCCCCGATCACCCAGGGTGACAGGGGAGTGGGCTCCAGTGCTGTTATTCTAGATGATAACTTTGTA
ACAAAGGCCACAGCCCTCACCTATGACCCCTATGTAAACTACTCCTCCCGCCATACCATAACCCAGCCCTTCTCCTACCA
CTCCCGCTACTTTACCCCCAAACCTGTCCTAGATTCCACTATCGATTACTTCCAACCAAACAACAAAAGAAACCAGCTGT
GGCTGAGACTACAAACTGCTGGAAATGTAGACCACGTAGGCCTCGGCACTGCGTTCGAAAACAGTATATACGACCAG
GAATACAATATCCGTGTAACCATGTATGTACAATTCAGAGAATTTAATCTTAAAGACCCCCCACTTAACCCT--------

>DQ910866_pcv2b

ATGACGTATCCAAGGAGGCGTTACCGGAGAAGAAGACACCGCCCCCGCAGCCATCTTGGCCAGATCCTCCGCCGCCG
CCCCTGGCTCGTCCACCCCCGCCACCGTTACCGCTGGAGAAGGAAAAATGGAATCTTCAACACCCGCCTCTCCCGCACC
TTCGGATATACTATCAAGCGAACCACAGTCAAAACGCCCTCCTGGGCGGTGGACATGATGAGATTCAATATTAATGAC
TTTCTTCCCCCAGGAGGGGGCTCAAACCCCCGCTCTGTGCCCTTTGAATACTACAGAATAAGAAAGGTTAAGGTTGAA
TTCTGGCCCTGCTCCCCGATCACCCAGGGTGACAGGGGAGTGGGCTCCAGTGCTGTTATTCTGGATGATAACTTTGTA
ACAAAGGCCACAGCCCTCACCTATGACCCCTATGTAAACTACTCCTCCCGCCATACCATAACCCAGCCCTTCTCCTACCA
CTCCCGCTACTTTACCCCCAAACCTGTCCTAGATTCCACTATTGATTACTTCCAACCAAACAACAAAAGAAACCAGCTGT
GGCTGAGACTACAAACTGCTGGAAATGTAGACCACGTAGGCCCCGGCACTGCGTTCGAAAACAGTATATACGACCAG
GAATACAATATCCGTGTAACCATGTATGTACAATTCAGAGAATTTAATCTTAAAGACCCCCCACTTAACCCT--------

>EU980094_pcv2b

ATGACGTATCCAAGGAGGCGTTACCGGAGAAGAAGACACCGCCCCCGCAGCCATCTTGGCCAGATCCTCCGCCGCCG
CCCCTGGCTCGTCCACCCCCGCCACCGTTACCGCTGGAGAAGGAAAAATGGCATCTTCAACACCCGCCTCTCCCGCACC
TTCGGATATACTATCAAGCGAACCACAGTCAAAACGCCCTCCTGGGCGGTGGACATGATGAGATTCAATATTAATGAC
TTTCTTCCCCCAGGAGGGGGCTCAAACCCCCGCTCTGTGCCCTTTGAATACTACAGAATAAGAAAGGTTAAGGTTGAA
TTCTGGCCCTGCTCCCCGATCACCCAGGGTGACAGGGGAGTGGGCTCCAGTGCTGTTATTCTAGATGATAACTTTGTA
ACAAAGGCCACAGCCCTCACCTATGACCCCTATGTAAACTACTCCTCCCGCCATACCATAACCCAGCCCTTCTCCTACCA
CTCCCGCTACTTTACCCCCAAACCTGTCCTAGATTCCACTATTGATTACTTCCAACCAAACAACAAAAGAAACCAGCTGT
GGCTGAGACTACAAACTGCTGGAAATGTAGACCACGTAGGCCTCGGCACTGCGTTCGAAAACAGTATATACGACCAG
GAATACAATATCCGTGTAACCATGTATGTACAATTCAGAGAATTTAATCTTAAAGACCCCCCACTTAACCCT--------

>JN382184_pcv2b

ATGACGTATCCAAGGAGGCGTTACCGGAGAAGAAGACACCGCCCCCGCAGCCATCTTGGCCAGATCCTCCGCCGCCG
CCCCTGGCTCGTCCACCCCCGCCACCGTTACCGCTGGAGAAGGAAAAATGGCATCTTCAACACCCGCCTCTCCCGCACC
TTCGGATATACTATCAAGCGAACCACAGTCAAAACGCCCTCCTGGGCGGTTGACATGATGAGATTCAATATTAATGAC
TTTCTTCCCCCAGGAGGGGGCTCAAACCCCCGCTCTGTGCCCTTTGAATACTACAGAATAAGAAAGGTTAAGGTTGAA
TTCTGGCCCTGCTCCCCGATCACCCAGGGTGACAGGGGAGTGGGCTCCAGTGCTGTTATTCTAGATGATAACTTTGTA
ACAAAGGCCACAGCCCTCACCTATGACCCCTATGTAAACTACTCCTCCCGCCATACCATAACCCAGCCCCTCTCCTACCA
CTCCCGCTACTTTACCCCCAAACCTCTCCTAGACTCCACTATTGATTACCTCCAACCAAACAACAAAAGAAATCAGCTGT
GGCTGAGACTACAAACTGCTGGAAATGTAGACCACGTAGGCCTCGGCACTGCGTTCGAAAACAGTATATACGACCAG
GAATACAATATCCGTGTAACCATGTATGTACAATTCAGAGAATTTAATCTTAAAGACCCCCCACTTAACCCT--------

>HQ831535_pcv2b

ATGACGTATCCAAGGAGGCGTTACCGGAGAAGAAGACACCGCCCCCGCAGCCATCTTGGCCAGATCCTCCGCCGCCG
CCCCTGGCTCGTCCACCCCCGCCACCGTTACCGCTGGAGAAGAAAAATGGCATCTTCAACACCCGCCTCTCCCGCACC
TTCGGATATACTATCAAGCGAACCACAGTCAAAACGCCCTCCTGGGCGGTAGACATGATGAGATTCAATATTAATGAC
TTTCTTCCCCCAGGAGGGGGCTCAAACCCCCGCTCTGTGCCCTTTGAATACTACAGAATAAGAAAGGTTAAGGTTGAA
TTCTGGCCCTGCTCCCCGATCACCCAGGGTGACAGGGGAGTGGGCTCCAGTGCTGTTATTCTAGATGATAACTTTGTA
ACAAAGGCCACAGCCCTCACCTATGACCCCTATGTAAACTACTCCTCCCGCCATACCATAACCCAGCCCTTCTCCTACCA
CTCCCGCTACTTTACCCCCAAACCTGTCCTAGACTCCACTATTGATTACTTCCAACCAAACAACAAAAGAAACCAGCTAT
GGCTGAGACTACAAACTGCTGGAAATGTAGACCACGTAGGCCTCGGCACTGCGTTCGAAAACAGTATATACGACCAG
GAATACAATATCCGTGTAACCATGTATGTACAATTCAGAGAATTTAATCTTAAAGACCCCCCACTTAACCCT--------

>HQ831531_pcv2b

ATGACGTATCCAAGGAGGCGTTACCGGAGAAGAAGACACCGCCCCCGCAGCCATCTTGGCCAGATCCTCCGCCGCCG
CCCCTGGCTCGTCCACCCCCGCCACCGTTACCGCTGGAGAAGGAAAAATGGCATCTTCAACACCCGCCTCTCCCGCACC
TTCGGATATACTATCAAGCGAACCACAGTCAGAACGCCCTCCTGGGCGGTAGACATGATGAGATTCAACATTAATGAC
TTTCTTCCCCCAGGAGGGGGCTCAAACCCCCGCTCTGTGCCCTTTGAATACTACAGAATAAGAAAGGTTAAGGTTGAA
TTCTGGCCCTGCTCCCCGATCACCCAGGGTGACAGGGGAGTGGGCTCCAGTGCTGTTATTCTAGATGATAACTTTGTA
ACAAAGGCCACAGCCCTCACCTATGACCCCTATGTAAACTACTCCTCCCGCCATACCATAACCCAGCCCTTCTCCTACCA
CTCCCGCTACTTTACCCCCAAACCTGTCCTAGACTCCACTATTGATTACTTCCAACCAAACAACAAAAGAAACCAGCTGT
GGCTGAGACTACAAACTGCTGGAAATGTAGACCACGTAGGCCTCGGCACTGCGTTCGAAAACAGTATATACGACCAG
GAATACAATATCCGTGTAACCATGTATGTACAATTCAGAGAATTTAATCTTAAAGACCCCCCACTTAACCCT--------

>HQ831532_pcv2b

ATGACGTATCCAAGGAGGCGTTACCGGAGAAGAAGACACCGCCCCCGCAGCCATCTTGGCCAGATCCTCCGCCGCCG
CCCCTGGCTCGTCCACCCCCGCCACCGTTACCGCTGGAGAAGGAAAAATGGCATCTTCAACACCCGCCTCTCCCGCACC
TTCGGATATACTATCAAGCGAACCACAGTCAGAACGCCCTCCTGGGCGGTAGACATGATGAGATTCAATATTAATGAC
TTTCTTCCCCCAGGAGGGGGCTCAAACCCCCGCTCTGTGCCCTTTGAATACTACAGAATAAGAAAGGTTAAGGTTGAA
TTCTGGCCCTGCTCCCCGATCACCCAGGGTGACAGGGGAGTGGGCTCCAGTGCTGTTATTCTAGATGATAACTTTGTA
ACAAAGGCCACAGCCCTCACCTATGACCCCTATGTAAACTACTCCTCCCGCCATACCATAACCCAGCCCTTCTCCTACCA
CTCCCGCTACTTTACCCCCAAACCTGTCCTAGACTCCACTATTGATTACTTCCAACCAAACAACAAAAGAAACCAGCTGT
GGCTGAGACTACAAACTGCTGGAAATGTAGACCACGTAGGCCTCGGCACTGCGTTCGAAAACAGTATATACGACCAG
GAATACAATATCCGTGTAACCATGTATGTACAATTCAGAGAATTTAATCTTAAAGACCCCCCACTTAACCCT--------

>EF493839_pcv2b

ATGACGTATCCAAGGAGGCGTTACCGGAGAAGAAGACGCCGCCCCCGCAGCCATCTTGGCCAGATCCTCCGCCGCCG
CCCCTGGCTCGTCCACCCCCGCCACCGTTACCGCTGGAGAAGGAAAAATGGCATCTTCAACACCCGCCTCTCCCGCACC
TTCGGATATACTATCAAGCGAACCACAGTCAAAACGCCCTCCTGGGCGGTGGACATGATGAGATTCAATATTAATGAC
TTTCTTCCCCCAGGAGGGGGCTCAAACCCCCGCTCTGTGCCCTTTGAATACTACAGAATAAGAAAGGTTAAGGTTGAA
TTCTGGCCCTGCTCCCCGATCACCCAGGGTGACAGGGGAGTGGGCTCCAGTGCTGTTATTCTAGATGATAACTTTGTA
ACAAAGGCCACAGCCCTCACCTATGACCCCTATGTAAATTACTCCTCCCGCCATACCATAACCCAGCCCCTCTCCTACCA
CTCCCGCTACTTTACCCCCAAACCTGTCCTAGATTCCACTATTGATTACTTCCAACCAAACAACAAAAGAAATCAGCTGT
GGCTGAGACTACAAACTGCTGGAAATGTAGACCACGTAGGCCTCGGCACTGCGTTCGAAAACAGTATATACGACCAG
GAATACAATATCCGTGTAACCATGTATGTACAATTCAGAGAATTTAATCTTAAAGACTCCCCACTTAACCCT--------

>JF683400_pcv2b

ATGACGTATCCAAGGAGGCGTTACCGGAGAAGAAGACACCGCCCCCGCAGCCATCTTGGCCAGATCCTCCGCCGCCG
CCCCTGGCTCGTCCACCCCCGCCACCGTTACCGCTGGAGAAGGAAAAATGGCATCTTCAACACCCGCCTGTCCCGCAC
CTTCGGATATACTATCAAGCGAACCACAGTCAAAACGCCCTCCTGGGCGGTGGACATGATGAGATTCAATATTAATGA
CTTTCTTCCCCCAGGAGGGGGCTCAAACCCCCGCTCTGTGCCCTTTGAATACTACAGAATAAGAAAGGTTAAGGTTGA
ATTCTGGCCCTGCTCCCCGATCACCCAGGGTGACAGGGGAGTGGGCTCCAGTGCTGTTATTCTAGATGATAACTTTGT
AACAAAGGCCACAGCCCTCACCTATGACCCCTATGTAAACTACTCCTCCCGCCATACCATAACCCAGCCCTTCTCCTACC
ACTCCCGCTACTTTACCCCCAAACCTGTCCTAGATTCCACTATTGATTACTTCCAACCAAACAACAAAAGAAATCAGCTG
TGGCTGAGACTACAAACTGCTGGAAATGTAGACCACGTAGGCCTCGGCACTGCGTTCGAAAACAGTATATACGACCA
GGAATACAATATCCGTGTAACCATGTATGTACAATTCAGAGAATTTAATCTTAAAGACCCCCCACTTAACCCT--------

>EF524527_pcv2b

ATGACGTATCCAAGGAGGCGTTACCGGAGAAGAAGACACCGCCCCCGCAGCCATCTTGGCCAGATCCTCCGCCGCCG
CCCCTGGCTCGTCCACCCCCGCCACCGTTACCGCTGGAGAAGGAAAAATGGCATCTTCAACACCCGCCTCTCCCGCACC
TTCGGATATACTATCAAGCGAACCACAGTCAAAACGCCCTCCTGGGCGGTAGACATGATGAGATTCAATATTAATGAT
TTTCTTCCCCCAGGAGGGGGCTCAAACCCCCGCTCTGTGCCCTTTGAATACTACAGAATAAGAAAGGTTAAGGTTGAA
TTCTGGCCCTGCTCCCCGATCACCCAGGGTGACAGGAGAGTGGGCTCCAGTGCTGTTATTCTAGATGATAACTTTGTA
ACAAAGGCCACAGCCCTCACCTATGACCCCTATGTAAACTACTCCTCCCGCCATACCATAACCCAGCCCTTCTCCTACCA
CTCCCGCTACTTTACCCCCAAACCTGTCCTAGATTCCACTATTGATTACTTCCAACCAAACAACAAAAGAAATCAGCTGT
GGCTGAGACTACAAACTGCTGGAAATGTAGACCACGTAGGCCTCGGCACTGCGTTCGAAAACAGTATATACGACCAG
GAATACAATATCCGTGTAACCATGTATGTACAATTCAGAGAATTTAATCTGAAAGACCCCCCACTTAACCCT--------

>EF524525_pcv2b

ATGACGTATCCAAGGAGGCGTTACCGGAGAAGAAGACACCGCCCCCGCAGCCATCTTGGCCAGATCCTCCGCCGCCG
CCCCTGGCTCGTCCACCCCCGCCACCGTTACCGCTGGAGAAGGAAAAATGGCATCTTCAACACCCGCCTCTCCCGCACC
TTCGGATATACTATCAAGCGAACCACAGTCAAAACGCCCTCCTGGGCGGTAGACATGATGAGATTCAATATTAATGAT
TTTCTTCCCCCAGGAGGGGGCTCAAACCCCCGCTCTGTGCCCTTTGAATACTACAGAATAAGAAAGGTTAAGGTTGAA
TTCTGGCCCTGCTCCCCGATCACCCAGGGTGACAGGGGAGTGGGCTCCAGTGCTGTTATTCTAGATGATAACTTTGTA
ACAAAGGCCACAGCCCTCACCTATGACCCCTATGTAAACTACTCCTCCCGCCATACCATAACCCAGCCCTTCTCCTACCA
CTCCCGCTACTTTACCCCCAAACCTGTCCTAGATTCCACTATTGATTACTTCCAACCAAACAACAAAAGAAATCAGCTGT
GGCTGAGACTACAAACTGCTGGAAATGTAGACCACGTAGGCCTCGGCACTGCGTTCGAAAACAGTATATACGACCAG
GAATACAATATCCGTGTAACCATGTATGTACAATTCAGAGAATTTAATCTGAAAGACCCCCCACTTAACCCT--------

>AY321991_pcv2b

ATGACGTATCCAAGGAGGCGTTACCGGAGAAGAAGACACCGCCCCCGCAGCCATCTTGGCCAGATCCTCCGCCGCCG
CCCCTGGCTCGTCCACCCCCGCCACCGTTACCGCTGGAGAAGGAAAAATGGCATCTTCAACACCCGCCTCTCCCGCACC
TTCGGATATACTATCAAGCGAACCACAGTCAAAACGCCCTCCTGGGCGGTGGACATGATGAGATTCAATATTAATGAC
TTTCTTCCCCCAGGAGGGGGCTCAAACCCCCGCTCTGTGCCCTTTGAATACTACAGAATAAGAAAGGTTAAGGTTGAA
TTCTGGCCCTGCTCCCCGATCACCCAGGGTGACAGGGGAGTGGGCTCCAGTGCTGTTATTCTAGATGATAACTTTGTA
ACAAAGGCCACAGCCCTCACCTATGACCCCTATGTAAACTACTCCTCCCGCCATACCATAACCCAGCCCTTCTCCTACCA
CTCCCGCTACTTTACCCCCAAACCTGTCCTAGATTCCACTATTGATTACTTCCAACCAAACAACAAAAGAAATCAGCTGT
GGCTGAGACTACAAACTGCTGGAAATGTAGACCACGTAGGCCTCGGCACTGCGTTCGAAAACAGTATATACGACCAG
GAATACAATATCCGTGTAACCATGTATGTACAATTCAGAGAATTTAATCTGAAAGACCCCCCACTTAACCCT--------

>EF421973_pcv2b

ATGACGTATCCAAGGAGGCGTTACCGGAGAAGAAGACACCGCCCCCGCAGCCATCTTGGCCAGATCCTCCGCCGCCG
CCCCTGGCTCGTCCACCCCCGCCACCGTTACCGCTGGAGAAGGAAAAATGGCATCTTCAACACCCGCCTCTCCCGCACC
TTCGGATATACTATCAAGCGAACCACAGTCAAAACGCCCTCCTGGGCGGTGGACATGATGAGATTCAATATTAATGAC
TTTCTTCCCCCAGGAGGGGGCTCAAACCCCCGCTCTGTGCCCTTTGAATACTACAGAATAAGAAAGGTTAAGGTTGAA
TTCTGGCCCTACTCCCCGATCACCCAGGGTGACAGGGGAGTGGGCTCCAGTGCTGTTATTCTAGATGATAACTTTGTA
ACAAAGGCCACAGCCCTCACCTATGACCCCTATGTAAACTACTCCTCCCGCCATACCATAACCCAGCCCTTCTCCTACCA
CTCCCGCTACTTTACCCCCAAACCTGTCCTAGATTCCACTATTGATTACTTCCAACCAAACAACAAAAGAAATCAGCTGT
GGCTGAGACTACAAACTGCTGGAAATGTAGACCACGTAGGCCTCGGCACTGCGTTCGAAAACAGTATATACGACCAG
GAATACAATATCCGTGTAACCATGTATGTACAATTCAGAGAATTTAATCTTAAAGACCCCCCACTTAACCCT--------

>FJ623185_pcv2b

ATGACGTATCCAAGGAGGCGTTACCGGAGAAGAAGACACCGCCCCCGCAGCCATCTTGGCCAGATCCTCCGCCGCCG
CCCCTGGCTCGTCCACCCCCGCCACCGTTACCGCTGGAGAAGGAAAAATGGCATCTTCAACACCCGCCTCTCCCGCACC
TTCGGATATACTATCAAGCGAACCACAGTCAAAACGCCCTCCTGGGCGGTGGACATGATGAGATTCAATATTAATGAT
TTTCTTCCCCCAGGAGGGGGCTCAAACCCCCGCTCTGTGCCCTTTGAATACTACAGAATAAGAAAGGTTAAGGTTGAA
TTCTGGCCCTACTCCCCGATCACCCAGGGTGACAGGGGAGTGGGCTCCAGTGCTGTTATTCTAGATGATAACTTTGTA
ACAAAGGCCACAGCCCTCACCTATGACCCCTATGTAAACTACTCCTCCCGCCATACCATAACCCAGCCCTTCTCCTACCA
CTCCCGCTACTTTACCCCCAAACCTGTCCTAGATTCCACTATTGATTACTTCCAACCAAACAACAAAAGAAATCAGCTGT
GGCTGAGACTACAAACTGCTGGAAATGTAGACCACGTAGGCCTCGGCACTGCGTTCGAAAACAGTATATACGACCAG
GAATACAATATCCGTGTAACCATGTATGTACAATTCAGAGAATTTAATCTTAAAGACCCCCCACTTAACCCT--------

>DQ910865_pcv2b

ATGACGTATCCAAGGAGGCGTTACCGGAGAAGAAGACACCGCCCCCGCAGCCATCTTGGCCAGATCCTCCGCCGCCG
CCCCTGGCTCGTCCACCCCCGCCACCGTTACCGCTGGAGAAGGAAAAATGGCATCTTCAACACCCGCCTCTCCCGCACC
TTCGGATATACTATCAAGCGAACCACAGTCAAAACGCCCTCCTGGGCGGTGGACATGATGAGATTCAATATTAATGAT
TTTCTTCCCCCAGGAGGGGGCTCAAACCCCCGCTCTGTGCCCTTTGAATACTACAGAATAAGAAAGGTTAAGGTTGAA
TTCTGGCCCTGCTCCCCGATCACCCAGGGTGACAGGGGAGTGGGCTCCAGTGCTGTTATTCTAGATGATAACTTTGTA
ACAAAGGCCACAGCCCTCACCTATGACCCCTATGTAAACTACTCCTCCCGCCATACCATAACCCAGCCCTTCTCCTACCA
CTCCCGCTACTTTACCCCCAAACCTGTCCTAGATTCCACTATTGATTACTTCCAACCAAACAACAAAAGAAATCAGCTGT
GGCTGAGACTACAAACTGCTGGAAATGTAGACCACGTAGGCCTCGGCACTGCGTTCGAAAACAGTATATACGACCAG
GAATACAATATCCGTGTAACCATGTATGTACAATTCAGAGAATTTAATCTTAAAGACCCCCCACTTAACCCT--------

>HM038025_pcv2b

ATGACGTATCCAAGGAGGCGTTACCGGAGAAGAAGACACCGCCCCCGCAGCCATCTTGGCCAGATCCTCCGCCGCCG
CCCCTGGCTCGTCCACCCCCGCCACCGTTACCGCAGGAGAAGGAAAAATGGCATCTTCAACACCCGCCTCTCCCGCAC
CTTCGGATATACTATCAAGCGAACCACAGTCAAAACGCCCTCCTGGGCGGTGGACATGATGAGATTCAATATTAATGA
TTTTCTTCCCCCAGGAGGGGGCTCAAACCCCCGCTCTGTGCCCTTTGAATACTACAGAATGAGAAAGGTTAAGGTTGA
ATTCTGGCCCTGCTCCCCGATCACCCAGGGTGACAGGGGAGTGGGCTCCAGTGCTGTTATTCTAGATGATAACTTTGT
AACAAAGGCCACAGCCCTCACCTATGACCCCTATGTAAACTACTCCTCCCGCCATACCATAACCCAGCCCTTCTCCTACC
ACTCCCGCTACTTTACCCCCAAACCCGTCCTAGATTCCACTATTGATTACTTCCAACCAAACAACAAAAGAAATCAGCTG
TGGCTGAGACTACAAACTGCTGGAAATGTAGACCACGTAGGCCTCGGCACTGCGTTCGAAAACAGTATATACGACCA
GGAATACAATATCCGTGTAACCATGTATGTACAATTCAGAGAATTTAATCTTAAAGACCCCCCACTTAACCCT--------

>GU247988_pcv2b

ATGACGTATCCAAGGAGGCGTTACCGGAGAAGAAGACACCGCCCCCGCAGCCATCTTGGCCAGATCCTCCGCCGCCG
CCCCTGGCTCGTCCACCCCCGCCACCGTTACCGCTGGAGAAGGAAAAATGGCATCTTCAACACTCGCCTCTCCCGCACC
TTCGGATATACTATCAAGCGAACCACAGTCAAAACGCCCTCCTGGGCGGTGGACATGATGAGATTCAATATTAATGAT
TTTCTTCCCCCAGGAGGGGGCTCAAACCCCCGCTCTGTGCCCTTTGAATACTACAGAATAAGAAAGGTTAAGGTTGAA
TTCTGGCCCTGCTCCCCGATCACCCAGGGTGACAGGGGAGTGGGCTCCAGTGCTGTTATTCTAGATGATAACTTTGTA
ACAAAGGCCACAGCCCTCACCTATGACCCCTATGTAAACTACTCCTCCCGCCATACCATAACCCAGCCCTTCTCCTACCA
CTCCCGCTACTTTACCCCCAAACCTGTCCTAGATTCCACTATTGATTACTTCCAACCAAACAACAAAAGAAATCAGCTGT
GGCTGAGACTACAAACTGCTGGAAATGTAGACCACGTAGGCCTCGGCACTGCGTTCGAAAACAGTATATACGACCAG
GAATACAATATCCGTGTAACCATGTATGTACAATTCAGAGAATTTAATCTTAAAGACCCCCCACTTAACCCT--------

>AY678532_pcv2b

ATGACGTATCCAAGGAGGCGGTACCGGAGAAGAAGACACCGCCCCCGCAGCCATCTTGGCCAGATCCTCCGCCGCCG
CCCCTGGCTCGTCCACCCCCGCCACCGTTACCGCTGGAGAAGGAAAAATGGCATCTTCAACACCCGCCTCTCCCGCACC
TTCGGATATACTATCAAGCGAACCACAGTCAAAACGCCCTCCTGGGCGGTGGACATGATGAGATTCAATATTAATGAT
TTTCTTCCCCCAGGAGGGGGCTCAAACCCCCGCTCTGTGCCCTTTGAATACTACAGAATAAGAAAGGTTAAGGTTGAA
TTCTGGCCCTGCTCCCCGATCACCCAGGGTGACAGGGGAGTGGGCTCCAGTGCTGTTATTCTAGATGATAACTTTGTA
ACAAAGGCCACAGCCCTCACCTATGACCCCTATGTAAACTACCCCTCCCGCCATACCATAACCCAGCCCTTCTCCTACCA
CTCCCGCTACTTTACCCCCAAACCTGTCCTAGATTCCACTATTGATTACTTCCAACCAAACAACAAAAGAAATCAGCTGT
GGCTGAGACTACAAACTGCTGGAAATGTAGACCACGTAGGCCTCGGCACTGCGTTCGAAAACAGTATATACGACCAG
GAATACAATATCCGTGTAACCATGTATGTACAATTCAGAGAATTTAATCTTAAAGACCCCCCACTTAACCCT--------

>EF675244_pcv2b

ATGACGTATCCAAGGAGGCGTTACCGGAGAAGAAGACACCGCCCCCGCAGCCATCTTGGCCAGATCCTCCGCCGCCG
CCCCTGGCTCGTCCACCCCCGCCACCGTTACCGCTGGAGAAGGAAAAATGGCATCTTCAACACCCGCCTCTCCCGCACC
TTCGGATATACTATCAAGCGAACCACAGTCAAAACGCCCTCCTGGGCGGTGGACATGATGAGATTCAATATTAATGAT
TTTCTTCCCCCAGGAGGGGGCTCAAACCCCCGCTCTGTGCCCTTTGAATACTACAGAATAAGAAAGGTTAAGGTTGAA
TTCTGGCCCTGCTCCCCGATCACCCAGGGTGACAGGGGAGTGGGCTCCAGTGCTGTTATTCTAGATGATAACTTTGTA
ACAAAGGCCACAGCCCTCACCTATGACCCCTATGTAAACTACTCCTCCCGCCATACCATAACACAGCCCTTCTCCTACCA
CTCCCGCTACTTTACCCCCAAACCTGTCCTAGATTCCACTATTGATTACTTCCAACCAAACAACAAAAGAAATCAGCTGT
GGCTGAGACTACAAACTGCTGGAAATGTAGACCACGTAGGCCTCGGCACTGCGTTCGAAAACAGTATATACGACCAG
GAATACAATATCCGTGTAACCATGTATGTACAATTCAGAGAATTTAATCTTAAAGACCCCCCACTTAACCCT--------

>EU521709_pcv2b

ATGACGTATCCAAGGAGGCGTTACCGGAGAAGAAGACACCGCCCCCGCAGCCATCTTGGCCAGATCCTCCGCCGCCG
CCCCTGGCTCGTCCACCCCCGCCACCGTTACCGCTGGAGAAGGAAAAATGGCATCCTCAACACCCGCCTCTCCCGCAC
CTTCGGATATACTATCAAGCGAACCACAGTCAAAACGCCCTCCTGGGCGGTGGACATGATGAGATTCAATATTAATGA
TTTTCTTCCCCCAGGAGGGGGCTCAAACCCCCGCTCTGTGCCCTTTGAATACTACAGAATAAGAAAGGTTAAGGTTGA
ATTCTGGCCCTGCTCCCCGATCACCCAGGGTGACAGGGGAGTGGGCTCCAGTGCTGTTATTCTAGATGATAACTTTGT
AACAAAGGCCACAGCCCTCACCTATGACCCCTATGTAAACTACTCCTCCCGCCATACCATAACCCAGCCCTTCTCCTACC
ACTCCCGCTACTTTACCCCCAAACCTGTCCTAGATTCCACTATTGATTACTTCCAACCAAACAACAAAAGAAATCAGCTG
TGGCTGAGACTACAAACTGCTGGAAATGTAGACCACGTAGGCCTCGGCACTGCGTTCGAAAATAGTATATACGACCA
GGAATACAATATCCGTGTAACCATGTATGTACAATTCAGAGAATTTAATCTTAAAGACCCCCCACTTAACCCT--------

>FJ644557_pcv2b

ATGACGTATCCAAGGAGGCGTTACCGGAGAAGAAGACACCGCCCCCGCAGCCATCTTGGCCAGATCCTCCGCCGCCG
CCCCTGGCTCGTCCACCCCCGCCACCGTTACCGCTGGAGAAGAAAAAATGGCATCTTCAACACCCGCCTCTCCCGCACC
TTCGGATATACTATCAAGCGAACCACAGTCAAAACGCCCTCCTGGGCGGTGGACATGATGAGATTCAATATTAATGAT
TTTCTTCCCCCAGGAGGGGGCTCAAACCCCCGCTCTGTGCCCTTTGAATACTACAGAATAAGAAAGGTTAAGGTTGAA
TTCTGGCCCTGCTCCCCGATCACCCAGGGTGACAGGGGAGTGGGCTCCAGTGCTGTTATTCTAGATGATAACTTTGTA
ACAAAGGCCACAGCCCTCACCTATGACCCCTATGTAAACTACTCCTCCCGCCATACCATAACCCAGCCCTTCTCCTACCA
CTCCCGCTACTTTACCCCCAAACCTGTCCTAGATTCCACTATTGATTACTTCCAACCAAACAACAAAAGAAATCAGCTGT
GGCTGAGACTACAAACTGCTGGAAATGTAGACCACGTAGGCCTAGGCACTGCGTTCGAAAACAGTATATACGACCAG
GAATATAATATCCGTGTAACCATGTATGTACAATTCAGAGAATTTAATCTTAAAGACCCCCCACTTAACCCT--------

>HQ591380_pcv2b

ATGACGTATCCAAGGAGGCGTTACCGGAGAAGAAGACACCGCCCCCGCAGCCATCTTGGCCAGATCCTCCGCCGCCG
CCCCTGGCTCGTCCACCCCCGCCACCGTTACCGCTGGAGAAGGAAAAATGGCATCTTCAACACCCGCCTCTCCCGCACC
TTCGGATATACTATCAAGCGAACCACAGTCAAAACGCCCTCCTGGGCGGTGGACATGATGAGATTCAATATTAATGAC
TTTCTTCCCCCAGGAGGGGGCTCAAACCCCCGCTCTGTGCCCTTTGAATACTACAGAATAAGAAAGGTTAAGGTTGAA
TTCTGGCCCTGCTCCCCGATCACCCAGGGTGACAGGGGAGTGGGCTCCAGTGCTGTTATTCTAGATGATAACTTTGTA
ACAAAGGCCACAGCCCTCACCTATGACCCCTATGTAAACTACTCCTCCCGCCATACCATAACCCAGCCCTTCTCCTACCA
CTCCCGCTACTTTACCCCCAAACCTGTCCTAGATTCCACTATTGATTACTTCCAACCAAACAACAAAAGAAATCAGCTGT
GGCTGAGACTACAAACTGCTGGAAATGTAGACCACGTAGGCCTCGGCACTGCGTTCGAAAACAGTATATACGACCAG
GAATACAATATCCGTGTAACCATGTATGTACAATTCAGAGAATTTAATCTTAAAGACCCCCCACTTAACCCT--------

>EF565347_pcv2b

ATGACGTATCCAAGGAGGCGTTACCGGAGAAGAAGACACCGCCCCCGCAGCCATCTTGGCCAGATCCTCCGCCGCCG
CCCCTGGCTCGTCCACCCCCGCCACCGTTACCGCTGGAGAAGGAAAAATGGCATCTTCAACACCCGCCTCTCCCGCACC
TTCGGATATACTATCAAGCGAACCACAGTCAAAACGCCCTCCTGGGCGGTGGACATGATGAGATTCAATATTAATGAC
TTTCTTCCCCCAGGAGGGGGCTCAAACCCCCGCTCTGTGCCCTTTGAATACTACAGAATAAGAAAGGTTAAGGTTGAA
TTCTGGCCCTGCTCCCCGATCACCCAGGGTGACAGGGGAGTGGGCTCCAGTGCTGTTATTCTAGATGATAACTTTGTA
ACAAAGGCCACAGCCCTCACCTATGACCCCTATGTAAACTACTCCTCCCGCCATACCATAACCCAGCCCTTCTCCTACCA
CTCCCGCTACTTTACCCCCAAACCTGTCCTAGATTCCACTATTGATTACTTCCAACCAAACAACAAAAGAAATCAGCTGT
GGCTGAGACTACAAACTGCTGGAAATGTAGACCACGTAGGCCTCGGCACTGCGTTCGAAAACAGTATATACGACCAG
GAATACAATATCCGTGTAACCATGTATGTACAATTCAGAGAATTTAATCTTAAAGACCCCCCACTTAACCCT--------

>AY874163_pcv2b

ATGACGTATCCAAGGAGGCGTTACCGGAGAAGAAGACACCGCCCCCGCAGCCATCTTGGCCAGATCCTCCGCCGCCG
CCCCTGGCTCGTCCACCCCCGCCACCGTTACCGCTGGAGAAGGAAAAATGGCATCTTCAACACCCGCCTCTCCCGCACC
TTCGGATATACTATCAAGCGAACCACAGTCAAAACGCCCTCCTGGGCGGTGGACATGATGAGATTCAATATTAATGAC
TTTCTTCCCCCAGGAGGGGGCTCAAACCCCCGCTCTGTGCCCTTTGAATACTACAGAATAAGAAAGGTTAAGGTTGAA
TTCTGGCCCTGCTCCCCGATCACCCAGGGTGACAGGGGAGTGGGCTCCAGTGCTGTTATTCTAGATGATAACTTTGTA
ACAAAGGCCACAGCCCTCACCTATGACCCCTATGTAAACTACTCCTCCCGCCATACCATAACCCAGCCCTTCTCCTACCA
CTCCCGCTACTTTACCCCCAAACCTGTCCTAGATTCCACTATTGATTACTTCCAACCAAACAACAAAAGAAATCAGCTGT
GGCTGAGACTACAAACTGCTGGAAATGTAGACCACGTAGGCCTCGGCACTGCGTTCGAAAACAGTATATACGACCAG
GAATACAATATCCGTGTAACCATGTATGTACAATTCAGAGAATTTAATCTTAAAGACCCCCCACTTAACCCT--------

>EF371544_pcv2b

ATGACGTATCCAAGGAGGCGTTACCGGAGAAGAAGACACCGCCCCCGCAGCCATCTTGGCCAGATCCTCCGCCGCCG
CCCCTGGCTCGTCCACCCCCGCCACCGTTACCGCTGGAGAAGGAAAAATGGCATCTTCAACACCCGCCTCTCCCGCACC
TTCGGATATACTATCAAGCGAACCACAGTCAAAACGCCCTCCTGGGCGGTGGACATGATGAGATTCAATATTAATGAC
TTTCTTCCCCCAGGAGGGGGCTCAAACCCCCGCTCTGTGCCCTTTGAATACTACAGAATAAGAAAGGTTAAGGTTGAA
TTCTGGCCCTGCTCCCCGATCACCCAGGGTGACAGGGGAGTGGGCTCCAGTGCTGTTATTCTAGATGATAACTTTGTA
ACAAAGGCCACAGCCCTCACCTATGACCCCTATGTAAACTACTCCTCCCGCCATACCATAACCCAGCCCTTCTCCTACCA
CTCCCGCTACTTTACCCCCAAACCTGTCCTAGATTCCACTATTGATTACTTCCAACCAAACAACAAAAGAAATCAGCTGT
GGCTGAGACTACAAACTGCTGGAAATGTAGACCACGTAGGCCTCGGCACTGCGTTCAAAAACAGTATATACGACCAG
GAATACAATATCCGTGTAACCATGTATGTACAATTCAGAGAATTTAATCTTAAAGACCCCCCACTTAACCCT--------

>EF565358_pcv2b

ATGACGTATCCAAGGAGGCGTTACCGGAGAAGAAGATACCGCCCCCGCAGCCATCTTGGCCAGATCCTCCGCCGCCG
CCCCTGGCTCGTCCACCCCCGCCACCGTTACCGCTGGAGAAGGAAAAATGGCATCTTCAACACCCGCCTCTCCCGCACC
TTCGGATATACTATCAAGCGAACCACAGTCAAAACGCCCTCCTGGGCGGTGGACATGATGAGATTCAATATTAATGAC
TTTCTTCCCCCAGGAGGGGGCTCAAACCCCCGCTCTGTGCCCTTTGAATACTACAGAATAAGAAAGGTTAAGGTTGAA
TTCTGGCCCTGCTCCCCGATCACCCAGGGTGACAGGGGAGTGGGCTCCAGTGCTGTTATTCTAGATGATAACTTTGTA
ACAAAGGCCACAGCCCTCACCTATGACCCCTATGTAAACTACTCCTCCCGCCATACCATAACCCAGCCCTTCTCCTACCA
CTCCCGCTACTTTACCCCCAAACCTGTCCTAGATTCCACTATTGATTACTTCCAACCAAACAACAAAAGAAATCAGCTGT
GGCTGAGACTACAAACTGCTGGAAATGTAGACCACGTAGGCCTCGGCACTGCGTTCGAAAACAGTATATACGACCAG
GAATACAATATCCGTGTAACCATGTATGTACAATTCAGAGAATTTAATCTTAAAGACCCCCCACTTAACCCT--------

>FJ158606_pcv2b

ATGACGTATCCAAGGAGGCGTTACCGGAGAAGAAGACACCGCCCCCGCAGCCATCTTGGCCAGATCCTCCGCCGCCG
CCCCTGGCTCGTCCACCCCCGCCACCGTTACCGCTGGAGAAGGAAAAATGGCATCTTCAACACCCGCCTCTCCCGCACC
TTCGGATATACTATCAAGCGAACCACAGTCAAAACGCCCTCCTGGGCGGTGGACATGATGAGATTCAATATTAATGAC
TTTCTTCCCCCAGGAGGGGGCTCAAACCCCCGCTCTGTGCCCTTTGAATACTACAGAATAAGAAAGGTTAAGGTTGAA
TTCTGGCCCTGCTCCCCGATCACCCAGGGTGACAGGGGAGTGGGCTCCAGTGCTGTTATTCTAGATGATAACTTTGTA
ACAAAGGCCACAGCCCTCACCTATGACCCCTATGTAAACTACTCCTCCCGCCATACCATAACCCAGCCCTTCTCCTACCA
CTCCCGCTACTTTACCCCCAAACCTGTCCTAGATTCCACTATTGATTACTTCCAACCAAACAACAAAAGAAATCAGCTGT
GGCTGAGACTACAAACTGCTGGAAATGTAGACCACGTAGGCCTCGGCACTGCGTTCGAAAACAGTATATACGACCAG
GAATACAATATCCGTGTAACCATGTATGTACAATTCAGAGAATTTAATCTTAAAGACCCCCCACTTAACCCT--------

>JN382173_pcv2b

ATGACGTATCCAAGGAGGCGTTACCGGAGAAGAAGACACCGCCCCCGCAGCCATCTTGGCCAGATCCTCCGCCGCCG
CCCCTGGCTCGTCCACCCCCGCCACCGTTACCGCTGGAGAAGGAAAAATGGCATCTTCAACACCCGCCTCTCCCGCACC
TTCGGATATACTATCAAGCGAACCACAGTCAAAACGCCCTCCTGGGCGGTGGACATGATGAGATTCAATATTAATGAC
TTTCTTCCCCCAGGAGGGGGCTCAAACCCCCGCTCTGTGCCCTTTGAATACTACAGAATAAGAAAGGTTAAGGTTGAA
TTCTGGCCCTGCTCCCCGATCACCCAGGGTGACAGGGGAGTGGGCTCCAGTGCTGTTATTCTAGATGATAACTTTGTA
ACAAAGGCCACAGCCCTCACCTATGACCCCTATGTAAACTACTCCTCCCGCCATACCATAACCCAGCCCTTCTCCTACCA
CTCCCGCTACTTTACCCCCAAACCTGTCCTAGATTCCACTATTGATTACTTCCAACCAAACAACAAAAGAAATCAGCTGT
GGCTGAGACTACAAACTGCTGGAAATGTAGACCACGTAGGCCTCGGCACTGCGTTCGAAAACAGTATATACGACCAG
GAATACAATATCCGTGTAACCATGTATGTACAATTCAGAGAATTTAATCTTAAAGACCCCCCACTTAACCCT--------

>AY682990_pcv2b

ATGACGTATCCAAGGAGGCGTTACCGGAGAAGAAGACACCGCCCCCGCAGCCATCTTGGCCAGATCCTCCGCCGCCG
CCCCTGGCTCGTCCACCCCCGCCACCGTTACCGCTGGAGAAGGAAAAATGGCATCTTCAACACCCGCCTCTCCCGCACC
TTCGGATATACTATCAAGCGAACCACAGTCAAAACGCCCTCCTGGGCGGTGGACATGATGAGATTCAATATTAATGAC
TTTCTTCCCCCAGGAGGGGGCTCAAACCCCCGCTCTGTGCCCTTTGAATACTACAGAATAAGAAAGGTTAAGGTTGAA
TTCTGGCCCTGCTCCCCGATCACCCAGGGTGACAGGGGAGTGGGCTCCAGTGCTGTTATTCTAGATGATAACTTTGTA
ACAAAGGCCACAGCCCTCACCTATGACCCCTATGTAAACTACTCCTCCCGCCATACCATAACCCAGCCCTTCTCCTACCA
CTCCCGCTACTTTACCCCCAAACCTGTCCTAGATTCCACTATTGATTACTTCCAACCAAACAACAAAAGAAATCAGCTGT
GGCTGAGACTACAAACTGCTGGAAATGTAGACCACGTAGGCCTCGGCACTGCGTTCGAAAACAGTATATACGACCAG
GAATACAATATCCGTGTAACCATGTATGTACAATTCAGAGAATTTAATCTTAAAGACCCCCCACTTAACCCT--------

>EF565349_pcv2b

ATGACGTATCCAAGGAGGCGTTACCGGAGAAGAAGACACCGCCCCCGCAGCCATCTTGGCCAGATCCTCCGCCGCCG
CCCCTGGCTCGTCCACCCCCGCCACCGTTACCGCTGGAGAAGGAAAAATGGCATCTTCAACACCCGCCTCTCCCGCACC
TTCGGATATACTATCAAGCGAACCACAGTCAAAACGCCCTCCTGGGCGGTGGACATGATGAGATTCAATATTAATGAC
TTTCTTCCCCCAGGAGGGGGCTCAAACCCCCGCTCTGTGCCCTTTGAATACTACAGAATAAGAAAGGTTAAGGTTGAA
TTCTGGCCCTGCTCCCCGATCACCCAGGGTGACAGGGGAGTGGGCTCCAGTGCTGTTATTCTAGATGATAACTTTGTA
ACAAAGGCCACAGCCCTCACCTATGACCCCTATGTAAACTACTCCTCCCGCCATACCATAACCCAGCCCTTCTCCTACCA
CTCCCGCTACTTTACCCCCAAACCTGTCCTAGATTCCACTATTGATTACTTCCAACCAAACAACAAAAGAAATCAGCTGT
GGCTGAGACTACAAACTGCTGGAAATGTAGACCACGTAGGCCTCGGCACTGCGTTCGAAAACAGTATATACGACCAG
GAATACAATATCCGTGTAACCATGTATGTACAATTCAGAGAATTTAATCTTAAAGACCCCCCACTTAACCCT--------

>FJ644558_pcv2b

ATGACGTATCCAAGGAGGCGTTACCGGAGAAGAAGACACCGCCCCCGCAGCCATCTTGGCCAGATCCTCCGCCGCCG
CCCCTGGCTCGTCCACCCCCGCCACCGTTACCGCTGGAGAAGGAAAAATGGCATCTTCAACACCCGCCTCTCCCGCACC
TTCGGATATACTATCAAGCGAACCACAGTCAAAACGCCCTCCTGGGCGGTGGACATGATGAGATTCAATATTAATGAC
TTTCTTCCCCCAGGAGGGGGCTCAAACCCCCGCTCTGTGCCCTTTGAATACTACAGAATAAGAAAGGTTAAGGTTGAA
TTCTGGCCCTGCTCCCCGATCACCCAGGGTGACAGGGGAGTGGGCTCCAGTGCTGTTATTCTAGATGATAACTTTGTA
ACAAAGGCCACAGCCCTCACCTATGACCCCTATGTAAACTACTCCTCCCGCCATACCATAACCCAGCCCTTCTCCTACCA
CTCCCGCTACTTTACCCCCAAACCTGTCCTAGATTCCACTATTGATTTCTTCCAACCAAACAACAAAAGAAATCAGCTGT
GGCTGAGACTACAAACTGCTGGAAATGTAGACCACGTAGGCCTCGGCACTGCGTTCGAAAACAGTATATACGACCAG
GAATACAATATCCGTGTAACCATGTATGTACAATTCAGAGAATTTAATCTTAAAGACCCCCCACTTAACCCT--------

>AY579893_pcv2b

ATGACGTATCCAAGGAGGCGTTACCGGAGAAGAAGACACCGCCCCCGCAGCCATCTTGGCCAGATCCTCCGCCGCCG
CCCCTGGCTCGTCCACCCCCGCCACCGTTACCGCTGGAGAAGGAAAAATGGCATCTTCAACACCCGCCTCTCCCGCACC
TTCGGATATACTATCAAGCGAACCACAGTCAAAACGCCCTCCTGGGCGGTGGACATGATGAGATTCAATATTAATGAC
TTTCTTCCCCCAGGAGGGGGCTCAAACCCCCGCTCTGTGCCCTTTGAATACTACAGAATAAGAAAGGTTAAGGTTGAA
TTCTGGCCCTGCTCCCCGATCACCCAGGGTGACAGGGGAGTGGGCTCCAGTGCTGTTATTCTAGATGATAACTTTGTA
ACAAAGGCCACAGCCCTCACCTATGACCCCTATGTAAACTACTCCTCCCGCCATACCATAACCCAGCCCTTCTCCTACCA
CTCCCGCTACTTTACCCCCAAACCTGTCCTAGATTCCACTATTGATTACTTCCAACCAAACAACAAAAGAAATCAGCTGT
GGCTGAGACTACAAACTGCTGGAAATGTAGACCACGTAGGCCTCGGCACTGCGTTCGAAAACAGTATATACGACCAG
GAATACAATATCCGTGTAACCATGTATGTACAATTCAGAGAATTTAATCTTAAAGACCCCCCACTTAACCCT--------

>JN006458_pcv2b

ATGACGTATCCAAGGAGGCGTTACCGGAGAAGAAGACACCGCCCCCGCAGCCATCTTGGCCAGATCCTCCGCCGCCG
CCCCTGGCTCGTCCACCCCCGCCACCGTTACCGCTGGAGAAGGAAAAATGGCATCTTCAACACCCGCCTCTCCCGCACC
TTCGGATATACTATCAAGCGAACCACAGTCAAAACGCCCTCCTGGGCGGTGGACATGATGAGATTCAATATTAATGAC
TTTCTTCCCCCAGGAGGGGGCTCAAACCCCCGCTCTGTGCCCTTTGAATACTACAGAATAAGAAAGGTTAAGGTTGAA
TTCTGGCCCTGCTCCCCGATCACCCAGGGTGACAGGGGAGTGGGCTCCAGTGCTGTTATTCTAGATGATAACTTTGTA
ACGAAGGCCACAGCCCTCACCTATGACCCCTATGTAAACTACTCCTCCCGCCATACCATAACCCAGCCCTTCTCCTACCA
CTCCCGCTACTTTACCCCCAAACCTGTCCTAGATTCCACTATTGATTACTTCCAACCAAACAACAAAAGAAATCAGCTGT
GGCTGAGACTACAAACTGCTGGAAATGTAGACCACGTAGGCCTCGGCACTGCGTTCGAAAACAGTATATACGACCAG
GAATACAATATCCGTGTAACCATGTATGTACAATTCAGAGAATTTAATCTTAAAGACCCCCCACTTAACCCT--------

>HQ591372_pcv2b

ATGACGTATCCAAGGAGGCGTTACCGGAGAAGAAGACACCGCCCCCGCAGCCATCTTGGCCAGATCCTCCGCCGCCG
CCCCTGGCTCGTCCACCCCCGCCACCGTTACCGCTGGAGAAGGAAAAATGGCATCTTCAACACCCGCCTCTCCCGCACC
TTCGGATATACTATCAAGCGAACCACAGTCAAAACGCCCTCCTGGGCGGTGGACATGATGAGATTCAATATTAATGAC
TTTCTTCCCCCAGGAGGGGGCTCAAACCCCCGCTCTGTGCCCTTTGAATACTACAGAATAAGAAAGGTTAAGGTTGAA
TTCTGGCCCTGCTCCCCGATCACCCAGGGTGACAGGGGAGTGGGCTCCAGTGCTGTTATTCTAGATGATAACTTTGTA
ACAAAGGCCACAGCCCTCACCTATGACCCCTATGTAAACTACTCCTCCCGCCATACCATAACCCAGCCCTTCTCCTACCA
CTCCCGCTACTTTACCCCCAAACCTGTCCTAGATTCCACTATTGATTACTTCCAACCAAACAACAAAAGAAATCAGCTGT
GGCTGAGACTACAAACTGCTGGAAATGTAGACCACGTAGGCCTCGGCACTGCGTTCGAAAACAGTATATACGACCAG
GAATACAATATCCGTGTAACCATGTATGTACAATTCAGAGAATTTAATCTTAAAGACCCCCCACTTAACCCT--------

>JX512858_pcv2b

ATGACGTATCCAAGGAGGCGTTACCGGAGAAGAAGACACCGCCCCCGCAGCCATCTTGGCCAGATCCTCCGCCGCCG
CCCCTGGCTCGTCCACCCCCGCCACCGTTACCGCTGGAGAAGGAAAAATGGCATCTTCAACACCCGCCTCTCCCGCACC
TTCGGATATACTATCAAGCGAACCACAGTCAAAACGCCCTCCTGGGCGGTGGACATGATGAGATTCAATATTAATGAC
TTTCTTCCCCCAGGAGGGGGCTCAAACCCCCGCTCTGTGCCCTTTGAATACTACAGAATAAGAAAGGTTAAGGTTGAA
TTCTGGCCCTGCTCCCCGATCACCCAGGGTGACAGGGGAGTGGGCTCCAGTGCTGTTATTCTAGATGATAACTTTGTA
ACAAAGGCCACAGCCCTCACCTATGACCCCTATGTAAACTACTCCTCCCGCCATACCATAACCCAGCCCTTCTCCTACCA
CTCCCGCTACTTTACCCCCAAACCTGTCCTAGATTCCACTATTGATTACTTCCAACCAAACAACAAAAGAAATCAGCTGT
GGCTGAGACTACAAACTGCTGGAAATGTAGACCACGTAGGCCTCGGCACTGCGTTCGAAAACAGTATATACGACCAG
GAATACAATATCCGTGTAACCATGTATGTACAATTCAGAGAATTTAATCTTAAAGACCCCCCACTTAACCCT--------

>EU418627_pcv2b

ATGACGTATCCAAGGAGGCGTTACCGGAGAAGAAGACACCGCCCCCGCAGCCATCTTGGCCAGATCCTCCGCCGCCG
CCCCTGGCTCGTCCACCCCCGCCACCGTTACCGCTGGAGAAGGAAAAATGGCATCTTCAACACCCGCCTCTCCCGCACC
TTCGGATATACTATCAAGCGAACCACAGTCAAAACGCCCTCCTGGGCGGTGGACATGATGAGATTCAATATTAATGAC
TTTCTTCCCCCAGGAGGGGGCTCAAACCCCCGCTCTGTGCCCTTTGAATACTACAGAATAAGAAAGGTTAAGGTTGAA
TTCTGGCCCTGCTCCCCGATCACCCAGGGTGACAGGGGAGTGGGCTCCAGTGCTGTTATTCTAGATGATAACTTTGTA
ACAAAGGCCACAGCCCTCACCTATGACCCCTATGTAAACTACTCCTCCCGCCATACCATAACCCAGCCCTTCTCCTACCA
CTCCCGCTACTTTACCCCCAAACCTGTCCTAGATTCCACTATTGATTACTTCCAACCAAACAACAAAAGAAATCAGCTGT
GGCTGAGACTACAAACTGCTGGAAATGTAGACCACGTAGGCCTCGGCACTGCGTTCGAAAACAGTATATACGACCAG
GAATACAATATCCGTGTAACCATGTATGTACAATTCAGAGAATTTAATCTTAAAGACCCCCCACTTAACCCT--------

>JN382166_pcv2b

ATGACGTATCCAAGGAGGCGTTACCGGAGAAGAAGACACCGCCCCCGCAGCCATCTTGGCCAGATCCTCCGCCGCCG
CCCCTGGCTCGTCCACCCCCGCCACCGTTACCGCTGGAGAAGGAAAAATGGCATCTTCAACACCCGCCTCTCCCGCACC
TTCGGATATACTATCAAGCGAACCACAGTCAAAACGCCCTCCTGGGCGGTGGACATGATGAGATTCAATATTAATGAC
TTTCTTCCCCCAGGAGGGGGCTCAAACCCCCGCTCTGTGCCCTTTGAATACTACAGAATAAGAAAGGTTAAGGTTGAA
TTCTGGCCCTGCTCCCCGATCACCCAGGGTGACAGGGGAGTGGGCTCCAGTGCTGTTATTCTAGATGATAACTTTGTA
ACAAAGGCCACAGCCCTCACCTATGACCCCTATGTAAACTACTCCTCCCGCCATACCATAACCCAGCCCTTCTCCTACCA
CTCCCGCTACTTTACCCCCAAACCTGTCCTAGATTCCACTATTGATTACTTCCAACCAAACAACAAAAGAAATCAGCTGT
GGCTGAGACTACAAACTGCTGGAAATGTAGACCACGTAGGCCTCGGCACTGCGTTCGAAAACAGTATATACGACCAG
GAATACAATATCCGTGTAACCATGTATGTACAATTCAGAGAATTTAATCTTAAAGACCCCCCACTTAACCCT--------

>HM038027_pcv2b

ATGACGTATCCAAGGAGGCGTTACCGGAGAAGAAGACACCGCCCCCGCAGCCATCTTGGCCAGATCCTCCGCCGCCG
CCCCTGGCTCGTCCACCCCCGCCACCGTTACCGCTGGAGAAGGAAAAATGGCATCTTCAACACCCGCCTCTCCCGCACC
TTCGGATATACTATCAAGCGAACCACAGTCAAAACGCCCTCCTGGGCGGTGGACATGATGAGATTCAATATTAATGAC
TTTCTTCCCCCAGGAGGGGGCTCAAACCCCCGCTCTGTGCCCTTTGAATACTACAGAATAAGAAAGGTTAAGGTTGAA
TTCTGGCCCTGCTCCCCGATCACCCAGGGTGACAGGGGAGTGGGCTCCAGTGCTGTTATTCTAGATGATAACTTTGTA
ACAAAGGCCACAGCCCTCACCTATGACCCCTATGTAAACTACTCCTCCCGCCATACCATAACCCAGCCCTTCTCCTACCA
CTCCCGCTACTTTACCCCCAAACCTGTCCTAGATTCCACTATTGATTACTTACAACCAAACAACAAAAGAAATCAGCTGT
GGCTGAGACTACAAACTGCTGGAAATGTAGACCACGTAGGCCTCGGCACTGCGTTCGAAAACAGTATATACGACCAG
GAATACAATATCCGTGTAACCATGTATGTACAATTCAGAGAATTTAATCTTAAAGCCCCCACACTTAACCCT--------

>FJ948167_pcv2b

ATGACGTATCCAAGGAGGCGTTACCGGAGAAGAAGACACCGCCCCCGCAGCCATCTTGGCCAGATCCTCCGCCGCCG
CCCCTGGCTCGTCCACCCCCGCCACCGTTACCGCTGGAGAAGGAAAAATGGCATCTTCAACACCCGCCTCTCCCGCACC
TTCGGATATACTATCAAGCGAACCACAGTCAAAACGCCCTCCTGGGCGGTGGACATGATGAGATTCAATATTAATGAC
TTTCTTCCCCCAGGAGGGGGCTCAAACCCCCGCTCTGTGCCCTTTGAATACTACAGAATAAGAAAGGTTAAGGTTGAA
TTCTGGCCCTGCTCCCCGATCACCCAGGGTGACAGGGGAGTGGGCTCCAGTGCTGTTATTCTAGATGATAACTTTGTA
ACAAAGGCCACAGCCCTCACCTATGACCCCTATGTAAACTACTCCTCCCGCCATACCATAACCCAGCCCTTCTCCTACCA
CTCCCGCTACTTTACCCCCAAACCTGTCCTAGATTCCACTATTGATTACTTCCAACCAAACAACAAAAGAAATCAGCTGT
GGCTGAGACTACAAACTGCTGGAAATGTAGACCACGTAGGCCTCGGCACTGCGTTCGAAAACAGTATATACGACCAG
GAATACAATATCCGTGTAACCATGTATGTACAATTCAGAGAATTTAATCTTAAAGACCCCCCACTTAACCCT--------

>HQ591378_pcv2b

ATGACGTATCCAAGGAGGCGTTACCGGAGAAGAAGACACCGCCCCCGCAGCCATCTTGGCCAGATCCTCCGCCGCCG
CCCCTGGCTCGTCCACCCCCGCCACCGTTACCGCTGGAGAAGGAAAAATGGCATCTTCAACACCCGCCTCTCCCGCACC
TTCGGATATACTATCAAGCGAACCACAGTCAAAACGCCCTCCTGGGCGGTGGACATGATGAGATTCAATATTAATGAC
TTTCTTCCCCCAGGAGGGGGCTCAAACCCCCGCTCTGTGCCCTTTGAATACTACAGAATAAGAAAGGTTAAGGTTGAA
TTCTGGCCCTGCTCCCCGATCACCCAGGGTGACAGGGGAGTGGGCTCCAGTGCTGTTATTCTAGATGATAACTTTGTA
ACAAAGGCCACAGCCCTCACCTATGACCCCTATGTAAACTACTCCTCCCGCCATACCATAACCCAGCCCTTCTCCTACCA
CTCCCGCTACTTTACCCCCAAACCTGTCCTAGATTCCACTATTGATTACTTCCAACCAATCAACAAAAGAAATCAGCTGT
GGCTGAGACTACAAACTGCTGGAAATGTAGACCACGTAGGCCTCGGCACTGCGTTCGAAAACAGTATATACGACCAG
GAATACAATATCCGTGTAACCATGTATGTACAATTCAGAGAATTTAATCTTAAAGACCCCCCACTTAACCCT--------

>EU136720_pcv2b

ATGACGTATCCAAGGAGGCGTTACCGGAGAAGAAGACACCGCCCCCGCAGCCATCTTGGCCAGATCCTCCGCCGCCG
CCCCTGGCTCGTCCACCCCCGCCACCGTTACCGCTGGAGAAGGAAAAATGGCATCTTCAACACCCGCCTCTCCCGCACC
TTCGGATATACTATCAAGCGAACCACAGTCAAAACGCCCTCCTGGGCGGTGGACATGATGAGATTCAATATTAATGAC
TTTCTTCCCCCAGGAGGGGGCTCAAACCCCCGCTCTGTGCCCTTTGAATACTACAGAATAAGAAAGGTTAAGGTTGAA
TTCTGGCCCTGCTCCCCGATCACCCAGGGTGACAGGGGAGTGGGCTCCAGTGCTGTTATTCTAGATGATAACTTTGTA
ACAAAGGCCACAGCCCTCACCTATGACCCCTATGTAAACTACTCCTCCCGCCATACCATAACCCAGCCCTTCTCCTACCA
CTCCCGCTACTTTACCCCCAAACCTGTCCTAGATTCCACTATTGATTACTTCCAACCAAACAACAAAAGAAATCAGCTGT
GGCTGAGACTACAAACTGCTGGAAATGTAGACCACGTAGGCCTCGGCACTGCGTTCGAAAACAGTATATACGACCAG
GAATACAATATCCGTGTAACCATGTATGTACAATTCAGAGAATTTAATCTTAAAGACCCCCCACTTAACCCT--------

>AY849938_pcv2b

ATGACGTATCCAAGGAGGCGTTACCGGAGAAGAAGACACCGCCCCCGCAGCCATCTTGGCCAGATCCTCCGCCGCCG
CCCCTGGCTCGTCCACCCCCGCCACCGTTACCGCTGGAGAAGGAAAAATGGCATCTTCAACACCCGCCTCTCCCGCACC
TTCGGATATACTATCAAGCGAACCACAGTCAAAACGCCCTCCTGGGCGGTGGACATGATGAGATTCAATATTAATGAC
TTTCTTCCCCCAGGAGGGGGCTCAAACCCCCGCTCTGTGCCCTTTGAATACTACAGAATAAGAAAGGTTAAGGTTGAA
TTCTGGCCCTGCTCCCCGATCACCCAGGGTGACAGGGGAGTGGGCTCCAGTGCTGTTATTCTAGATGATAACTTTGTA
ACAAAGGCCACAGCCCTCACCTATGACCCCTATGTAAACTACTCCTCCCGCCATACCATAACCCAGCCCTTCTCCTACCA
CTCCCGCTACTTTACCCCCAAACCTGTCCTAGATTCCACTATTGATTACTTCCAACCAAACAACAAAAGAAATCAGCTGT
GGCTGAGACTACAAACTGCTGGAAATGTAGACCACGTAGGCCTCGGCACTGCGTTCGAAAACAGTATATACGACCAG
GAATACAATATCCGTGTAACCATGTATGTACAATTCAGAGAATTTAATCTTAAAGACCCCCCACTTAACCCT--------

>AY321997_pcv2b

ATGACGTATCCAAGGAGGCGTTACCGGAGAAGAAGACACCGCCCCCGCAGCCATCTTGGCCAGATCCTCCGCCGCCG
CCCCTGGCTCGTCCACCCCCGCCACCGTTACCGCTGGAGAAGGAAAAATGGCATCTTCAACACCCGCCTCTCCCGCACC
TTCGGATATACTATCAAGCGAACCACAGTCAAAACGCCCTCCTGGGCGGTGGACATGATGAGATTCAATATTAATGAC
TTTCTTCCCCCAGGAGGGGGCTCAAACCCCCGCTCTGTGCCCTTTGAATACTACAGAATAAGAAAGGTTAAGGTTGAA
TTCTGGCCCTGCTCCCCGATCACCCAGGGTGACAGGGGAGTGGGCTCCAGTGCTGTTATTCTAGATGATAACTTTGTA
ACAAAGGCCACAGCCCTCACCTATGACCCCTATGTAAACTACTCCTCCCGCCATACCATAACCCAGCCCTTCTCCTACCA
CTCCCGCTACTTTACCCCCAAACCTGTCCTAGATTCCACTATTGATTACTTCCAACCAAACAACAAAAGAAATCAGCTGT
GGCTGAGACTACAAACTGCTGGAAATGTAGACCACGTAGGCCTCGGCACTGCGTTCGAAAACAGTATATACGACCAG
GAATACAATATCCGTGTAACCATGTATGTACAATTCAGAGAATTTAATCTTAAAGACCCCCCACTTAACCCT--------

>HM565924_pcv2b

ATGACGTATCCAAGGAGGCGTTACCGGAGAAGAAGACACCGCCCCCGCAGCCATCTTGGCCAGATCCTCCGCCGCCG
CCCCTGGCTCGTCCACCCCCGCCACCGTTACCGCTGGAGAAGGAAAAATGGCATCTTCAACACCCGCCTCTCCCGCACC
TTCGGATATACTATCAAGCGAACCACAGTCAAAACGCCCTCCTGGGCGGTGGACATGATGAGATTCAATATTAATGAC
TTTCTTCCCCCAGGAGGGGGCTCAAACCCCCGCTCTGTGCCCTTTGAATACTACAGAATAAGAAAGGTTAAGGTTGAA
TTCTGGCCCTGCTCCCCGATCACCCAGGGTGACAGGGGAGTGGGCTCCAGTGCTGTTATTCTAGATGATAACTTTGTA
ACAAAGGCCACAGCCCTCACCTATGACCCCTATGTAAACTACTCCTCCCGCCATACCATAACCCAGCCCTTCTCCTACCA
CTCCCGCTACTTTACCCCCAAACCTGTCCTAGATTCCACTATTGATTACTTCCAACCAAACAACAAAAGAAATCAGCTGT
GGCTGAGACTACAAACTGCTGGAAATGTAGACCACGTAGGCCTCGGCACTGCGTTCGAAAACAGTATATACGACCAG
GAATACAATATCCGTGTAACCATGTATGTACAATTCAGAGAATTTAATCTTAAAGACCCCCCACTTAACCCT--------

>GU247992_pcv2b

ATGACGTATCCAAGGAGGCGTTACCGGAGAAGAAGACACCGCCCCCGCAACCATCTTGGCCAGATCCTCCGCCGCCG
CCCCTGGCTCGTCCACCCCCGCCACCGTTACCGCTGGAGAAGGAAAAATGGCATCTTCAACACCCGCCTCTCCCGCACC
TTCGGATATACTATCAAGCGAACCACAGTCAAAACGCCCTCCTGGGCGGTGGACATGATGAGATTCAATATTAATGAC
TTTCTTCCCCCAGGAGGGGGCTCAAACCCCCGCTCTGTGCCCTTTGAATACTACAGAATAAGAAAGGTTAAGGTTGAA
TTCTGGCCCTGCTCCCCGATCACCCAGGGTGACAGGGGAGTGGGCTCCAGTGCTGTTATTCTAGATGATAACTTTGTA
ACAAAGGCCACAGCCCTCACCTATGACCCCTATGTAAACTACTCTTCCCGCCATACCATAACCCAGCCCTTCTCCTACCA
CTCCCGCTACTTTACCCCCAAACCTGTCCTAGATTCCACTATTGATTACTTCCAACCAAACAACAAAAGAAATCAGCTGT
GGCTGAGACTACAAACTGCTGGAAATGTAGACCACGTAGGCCTCGGCACTGCGTTCGAAAACAGTATATACGACCAG
GAATACAATATCCGTGTAACCATGTATGTACAATTCAGAGAATTTAATCTTAAAGACCCCCCACTTAACCCT--------

>EU545548_pcv2b

ATGACGTATCCAAGGAGGCGTTACCGGAGAAGAAGACACCGCCCCCGCAGCCATCTTGGCCAGATCCTCCGCCGCCG
CCCCTGGCTCGTCCACCCCCGCCACCGTTACCGCTGGAGAAGGAAAAATGGCATCTTCAACACCCGCCTCTCCCGCACC
TTCGGATATACTATCAAGCGAACCACAGTCAAAACGCCCTCCTGGGCGGTGGACATGATGAGATTCAATATTAATGAC
TTTCTTCCCCCAGGAGGGGGCTCAAACCCCCGCTCTGTGCCCTTTGAATACTACAGAATAAGAAAGGTTAAGGTTGAA
TTCTGGCCCTGCTCCCCGATCACCCAGGGTGACAGGGGAGTGGGCTCCAGTGCTGTTATTCTAGATGATAACTTTGTA
ACAAAGGCCACAGCCCTCACCTATGACCCCTATGTAAACTACTCCTCCCGCCATACCATAACCCAGCCCTTCTCCTACCA
CTCCCGCTACTTTACCCCCAAACCTGTCCTAGATTCCACTATTGATTACTTCCAACCAAACAACAAAAGAAATCAGCTGT
GGCTGAGACTACAAACTGCTGGAAATGTAGACCACGTAGGCCTCGGCACTGCGTTCGAAAACAGTATATACGACCAG
GAATACAATATCCGTGTAACCATGTATGTACAATTCAGAGAATTTAATCTTAAAGACCCCCCACTTAACCCT--------

>FJ905464_pcv2b

ATGACGTATCCAAGGAGGCGTTACCGGAGAAGAAGACACCGCCCCCGCAGCCATCTTGGCCAGATCCTCCGCCGCCG
CCCCTGGCTCGTCCACCCCCGCCACCGTTACCGCTGGAGAAGGAAAAATGGCATCTTCAACACCCGCCTCTCCCGCACC
TTCGGATATACTATCAAGCGAACCACAGTCAAAACGCCCTCCTGGGCGGTGGACATGATGAGATTCAATATTAATGAC
TTTCTTCCCCCAGGAGGGGGCTCAAACCCCCGCTCTGTGCCCTTTGAATACTACAGAATAAGAAAGGTTAAGGTTGAA
TTCTGGCCCTGCTCCCCGATCACCCAGGGTGACAGGGGAGTGGGCTCCAGTGCTGTTATTCTAGATGATAACTTTGTA
ACAAAGGCCACAGCCCTCACCTATGACCCCTATGTAAACTACTCCTCCCGCCATACCATAACCCAGCCCTTCTCCTACCA
CTCCCGCTACTTTACCCCCAAACCTGTCCTAGATTCCACTATTGATTACTTCCAACCAAACAACAAAAGAAATCAGCTGT
GGCTGAGACTACAAACTGCTGGAAATGTAGACCACGTAGGACTCGGCACTGCGTTCGAAAACAGTATATACGACCAG
GAATACAATATCCGTGTAACCATGTATGTACAATTCAGAGAATTTAATCTTAAAGACCCCCCACTTAACCCT--------

>EF565368_pcv2b

ATGACGTATCCAAGGAGGCGTTACCGGAGAAGAAGACACCGCCCCCGCAGCCATCTTGGCCAGATCCTCCGCCGCCG
CCCCTGGCTCGTCCACCCCCGCCACCGTTACCGCTGGAGAAGGAAAAATGGCATCTTCAACACCCGCCTCTCCCGCACC
TTCGGATATACTATCAAGCGAACCACAGTCAAAACGCCCTCCTGGGCGGTGGACATGATGAGATTCAATATTAATGAC
TTTCTTCCCCCAGGAGGGGGCTCAAACCCCCGCTCTGTGCCCTTTGAATACTACAGAATAAGAAAGGTTAAGGTTGAA
TTCTGGCCCTGCTCCCCGATCACCCAGGGTGACAGGGGAGTGGGCTCCAGTGCTGTTATTCTAGATGATAACTTTGTA
ACAAAGGCCACAGCCCTCACCTATGACCCCTATGTAAACTACTCCTCCCGCCATACCATAACCCAGCCCTTCTCCTACCA
CTCCCGCTACTTTACCCCCAAACCTGTCCTAGATTCCACTATTGATTACTTCCAACCAAACAACAAAAGAAATCAGCTGT
GGCTGAGACTACAAACTGCTGGAAATGTAGACCACGTAGGCCTCGGCACTGCGTTCGAAAACAGTATATACGACCAG
GAATACAATATCCGTGTAACCATGTATGTACAATTCAGAGAATTTAATCTTAAAGACCCCCCACTTAACCCT--------

>HM009333_pcv2b

ATGACGTATCCAAGGAGGCGTTACCGGAGAAGAAGACACCGCCCCCGCAGCCATCTTGGCCAGATCCTCCGCCGCCG
CCCCTGGCTCGTCCACCCCCGCCACCGTTACCGCTGGAGAAGGAAAAATGGCATCTTCAACACCCGCCTCTCCCGCACC
TTCGGATATACTATCAAGCGAACCACAGTCAAAACGCCCTCCTGGGCGGTGGACATGATGAGATTCAATATTAATGAC
TTTCTTCCCCCAGGAGGGGGCTCAAACCCCCGCTCTGTGCCCTTTGAATACTACAGAATAAGAAAGGTTAAGGTTGAA
TTCTGGCCCTGCTCCCCGATCACCCAGGGTGACAGGGGAGTGGGCTCCAGTGCTGTTATTCTAGATGATAACTTTGTA
ACAAAGGCCACAGCCCTCACCTATGACCCCTATGTAAACTACTCCTCCCGCCATACCATAACCCAGCCCTTCTCCTACCA
CTCCCGCTACTTTACCCCCAAACCTGTCCTAGATTCCACTATTGATTACTTCCAACCAAACAACAAAAGAAATCAGCTGT
GGCTGAGACTACAAACTGCTGGAAATGTAGACCACGTAGGCCTCGGCACTGCGTTCGAAAACAGTATATACGACCAG
GAATACAATATCCGTGTAACCATGTATGTACAATTCAGAGAATTTAATCTTAAAGACCCCCCACTTAACCCT--------

>EF565346_pcv2b

ATGACGTATCCAAGGAGGCGTTACCGGAGAAGAAGACACCGCCCCCGCAGCCATCTTGGCCAGATCCTCCGCCGCCG
CCCCTGGCTCGTCCACCCCCGCCACCGTTACCGCTGGAGAAGGAAAAATGGCATCTTCAACACCCGCCTCTCCCGCACC
TTCGGATATACTATCAAGCGAACCACAGTCAAAACGCCCTCCTGGGCGGTGGACATGATGAGATTCAATATTAATGAC
TTTCTTCCCCCAGGAGGGGGCTCAAACCCCCGCTCTGTGCCCTTTGAATACTACAGAATAAGAAAGGTTAAGGTTGAA
TTCTGGCCCTGCTCCCCGATCACCCAGGGTGACAGGGGAGTGGGCTCCAGTGCTGTTATTCTAGATGATAACTTTGTA
ACAAAGGCCACAGCCCTCACCTATGACCCCTATGTAAACTACTCCTCCCGCCATACCATAACCCAGCCCTTCTCCTACCA
CTCGCGCTACTTTACCCCCAAACCTGTCCTAGATTCCACTATTGATTACTTCCAACCAAACAACAAAAGAAATCAGCTGT
GGCTGAGACTACAAACTGCTGGAAATGTAGACCACGTAGGCCTCGGCACTGCGTTCGAAAACAGTATATACGACCAG
GAATACAATATCCGTGTAACCATGTATGTACAATTCAGAGAATTTAATCTTAAAGACCCCCCACTTAACCCT--------

>AY484414_pcv2b

ATGACGTATCCAAGGAGGCGTTACCGGAGAAGAAGACACCGCCCCCGCAGCCATCTTGGCCAGATCCTCCGCCGCCG
CCCCTGGCTCGTCCACCCCCGCCACCGTTACCGCTGGAGAAGGAAAAATGGCATCTTCAACACCCGCCTCTCCCGCACC
TTCGGATATACTATCAAGCGAACCACAGTCAAAACGCCCTCCTGGGCGGTGGACATGATGAGATTCAATATTAATGAC
TTTCTTCCCCCAGGAGGGGGCTCAAACCCCCGCTCTGTGCCCTTTGAATACTACAGAATAAGAAAGGTTAAGGTTGAA
TTCTGGCCCTGCTCCCCGATCACCCAGGGTGACAGGGGAGTGGGCTCCAGTGCTGTTATTCTAGATGATAACTTTGTA
ACAAAGGCCACAGCCCTCACCTATGACCCCTATGTAAACTACTCCTCCCGCCATACCATAACCCAGCCCTTCTCCTACCA
CTCCCGCTACTTTACCCCCAAACCTGTCCTAGATTCCACTATTGATTACTTCCAACCAAACAACAAAAGAAATCAGCTGT
GGCTGAGACTACAAACTGCTGGAAATGTAGACCACGTAGGCCTCGGCACTGCGTTCGAAAACAGTATATACGACCAG
GAATACAATATCCGTGTAACCATGTATGTACAATTCAGAGAATTTAATCTTAAAGACCCCCCACTTAACCCT--------

>AY322002_pcv2b

ATGACGTATCCAAGGAGGCGTTACCGGAGAAGAAGACACCGCCCCCGCAGCCATCTTGGCCAGATCCTCCGCCGCCG
CCCCTGGCTCGTCCACCCCCGCCACCGTTACCGCTGGAGAAGGAAAAATGGCATCTTCAACACCCGCCTCTCCCGCACC
TTCGGATATACTATCAAGCGAACCACAGTCAAAACGCCCTCCTGGGCGGTGGACATGATGAGATTCAATATTAATGAC
TTTCTTCCCCCAGGAGGGGGCTCAAACCCCCGCTCTGTGCCCTTTGAATACTACAGAATAAGAAAGGTTAAGGTTGAA
TTCTGGCCCTGCTCCCCGATCACCCAGGGTGACAGGGGAGTGGGCTCCAGTGCTGTTATTCTAGATGATAACTTTGTA
ACAAAGGCCACAGCCCTCACCTATGACCCCTATGTAAACTACTCCTCCCGCCATACCATAACCCAGCCCTTCTCCTACCA
CTCCCGCTACTTTACCCCCAAACCTGTCCTAGATTCCACTATTGATTACTTCCAACCAAACAACAAAAGAAATCAGCTGT
GGCTGAGACTACAAACTGCTGGAAATGTAGACCACGTAGGCCTCGGCACTGCGTTCGAAAACAGTATATACGACCAG
GAATACAATATCCGTGTAACCATGTATGTACAATTCAGAGAATTTAATCTTAAAGACCCCCCACTTAACCCT--------

>EU136717_pcv2b

ATGACGTATCCAAGGAGGCGTTACCGGAGAAGAAGACACCGCCCCCGCAGCCATCTTGGCCAGATCCTCCGCCGCCG
CCCCTGGCTCGTCCACCCCCGCCACCGTTACCGCTGGAGAAGGAAAAATGGCATCTTCAACACCCGCCTCTCCCGCACC
TTCGGATATACTATCAAGCGAACCACAGTCAAAACGCCCTCCTGGGCGGTGGACATGATGAGATTCAATATTAATGAC
TTTCTTCCCCCAGGAGGGGGCTCAAACCCCCGCTCTGTGCCCTTTGAATACTACAGAATAAGAAAGGTTAAGGTTGAA
TTCTGGCCCTGCTCCCCGATCACCCAGGGTGACAGGGGAGTGGGCTCCAGTGCTGTTATTCTAGATGATAACTTTGTA
ACAAAGGCCACAGCCCTCACCTATGACCCCTATGTAAACTACTCCTCCCGCCATACCATAACCCAGCCCTTCTCCTACCA
CTCCCGCTACTTTACCCCCAAACCTGTCCTAGATTCCACTATTGATTACTTCCAACCAAACAACAAAAGAAATCAGCTGT
GGCTGAGACTACAAACTGCTGGAAATGTAGACCACGTAGGCCTCGGCACTGCGTTCGAAAACAGTATATACGACCAG
GAATACAATATCCGTGTAACCATGTATGTACAATTCAGAGAATTTAATCTTAAAGACCCCCCACTTAACCCT--------

>EF675235_pcv2b

ATGACGTATCCAAGGAGGCGTTACCGGAGAAGAAGACACCGCCCCCGCAGCCATCTTGGCCAGATCCTCCGCCGCCG
CCCCTGGCTCGTCCACCCCCGCCACCGTTACCGCTGGAGAAGGAAAAATGGCATCTTCAACACCCGCCTCTCCCGCACC
TTCGGATATACTATCAAGCGAACCACAGTCAAAACGCCCTCCTGGGCGGTGGACATGATGAGATTCAATATTAATGAC
TTTCTTCCCCCAGGAGGGGGCTCAAACCCCCGCTCTGTGCCCTTTGAATACTACAGGATAAGAAAGGTTAAGGTTGAA
TTCTGGCCCTGCTCCCCGATCACCCAGGGTGACAGGGGAGTGGGCTCCAGTGCTGTTATTCTAGATGATAACTTTGTA
ACAAAGGCCACAGCCCTCACCTATGACCCCTATGTAAACTACTCCTCCCGCCATACCATAACCCAGCCCTTCTCCTACCA
CTCCCGCTACTTTACCCCCAAACCTGTCCTAGATTCCACTATTGATTACTTCCAACCAAACAACAAAAGAAATCAGCTGT
GGCTGAGACTACAAACTGCTGGAAATGTAGACCACGTAGGCCTCGGCACTGCGTTCGAAAACAGTATATACGACCAG
GAATACAATATCCGTGTAACCATGTATGTACAATTCAGAGAATTTAATCTTAAAGACCCCCCACTTAACCCT--------

>EF675234_pcv2b

ATGACGTATCCAAGGAGGCGTTACCGGAGAAGAAGACACCGCCCCCGCAGCCATCTTGGCCAGATCCTCCGCCGCCG
CCCCTGGCTCGTCCACCCCCGCCACCGTTACCGCTGGAGAAGGAAAAATGGCATCTTCAACACCCGCCTCTCCCGCACC
TTCGGATATACTATCAAGCGAACCACAGTCAAAACGCCCTCCTGGGCGGTGGACATGATGAGATTCAATATTAATGAC
TTTCTTCCCCCAGGAGGGGGCTCAAACCCCCGCTCTGTGCCCTTTGAATACTACAGGATAAGAAAGGTTAAGGTTGAA
TTCTGGCCCTGCTCCCCGATCACCCAGGGTGACAGGGGAGTGGGCTCCAGTGCTGTTATTCTAGATGATAACTTTGTA
ACAAAGGCCACAGCCCTCACCTATGACCCCTATGTAAACTACTCCTCCCGCCATACCATAACCCAGCCCTTCTCCTACCA
CTCCCGCTACTTTACCCCCAAACCTGTCCTAGATTCCACTATTGATTACTTCCAACCAAACAACAAAAGAAATCAGCTGT
GGCTGAGACTACAAACTGCTGGAAATGTAGACCACGTAGGCCTCGGCACTGCGTTCGAAAACAGTATATACGACCAG
GAATACAATATCCGTGTAACCATGTATGTACAATTCAGAGAATTTAATCTTAAAGACCCCCCACTTAACCCT--------

>EF565360_pcv2b

ATGACGTATCTAAGGAGGCGTTACCGGAGAAGAAGACACCGCCCCCGCAGCCATCTTGGCCAGATCCTCCGCCGCCG
CCCCTGGCTCGTCCACCCCCGCCACCGTTACCGCTGGAGAAGGAAAAATGGCATCTTCAACACCCGCCTCTCCCGCACC
TTCGGATATACTATCAAGCGAACCACAGTCAAAACGCCCTCCTGGGCGGTGGACATGATGAGATTCAATATTAATGAC
TTTCTTCCCCCAGGAGGGGGCTCAAACCCCCGCTCTGTGCCCTTTGAATACTACAGGATAAGAAAGGTTAAGGTTGAA
TTCTGGCCCTGCTCCCCGATCACCCAGGGTGACAGGGGAGTGGGCTCCAGTGCTGTTATTCTAGATGATAACTTTGTA
ACAAAGGCCACAGCCCTCACCTATGACCCCTATGTAAACTACTCCTCCCGCCATACCATAACCCAGCCCTTCTCCTACCA
CTCCCGCTACTTTACCCCCAAACCTGTCCTAGATTCCACTATTGATTACTTCCAACCAAACAACAAAAGAAATCAGCTGT
GGCTGAGACTACAAACTGCTGGAAATGTAGACCACGTAGGCCTCGGCACTGCGTTCGAAAACAGTATATACGACCAG
GAATACAATATCCGTGTAACCATGTATGTACAATTCAGAGAATTTAATCTTAAAGACCCCCCACTTAACCCT--------

>JN006451_pcv2b

ATGACGTATCCAAGGAGGCGTTATCGGAGAAGAAGACACCGCCCCCGCAGCCATCTTGGCCAGATCCTCCGCCGCCG
CCCCTGGCTCGTCCACCCCCGCCACCGTTACCGCTGGAGAAGGAAAAATGGCATCTTCAACACCCGCCTCTCCCGCACC
TTCGGATATACTATCAAGCGAACCACAGTCAAAACGCCCTCCTGGGCGGTGGACATGATGAGATTCAATATTAATGAC
TTTCTTCCCCCAGGAGGGGGCTCAAACCCCCGCTCTGTGCCCTTTGAATACTACAGAATAAGAAAGGTTAAGGTTGAA
TTCTGGCCCTGCTCCCCGATCACCCAGGGTGACAGGGGAGTGGGCTCCAGTGCTGTTATTCTAGATGATAACTTTGTA
ACAAAGGCCACAGCCCTCACCTATGACCCCTATGTAAACTACTCCTCCCGCCATACCATAACCCAGCCCTTCTCCTACCA
CTCCCGCTACTTTACCCCAAACCTGTCCTAGATTCCACTATTGATTACTTCCAACCAAACAGCAAAAGAAATCAGCTGT
GGCTGAGACTACAAACTGCTGGAAATGTAGACCACGTAGGCCTCGGCACTGCGTTCGAAAACAGTATATACGACCAG
GAATACAATATCCGTGTAACCATGTATGTACAATTCAGAGAATTTAATCTTAAAGACCCCCCACTTAACCCT--------

>JN006452_pcv2b

ATGACGTATCCAAGGAGGCGTTACCGGAGAAGAAGACACCGCCCCCGCAGCCATCTTGGCCAGATCCTCCGCCGCCG
CCCCTGGCTCGTCCACCCCCGCCACCGTTACCGCTGGAGAAGGAAAAATGGCATCTTCAACACCCGCCTCTCCCGCACC
TTCGGATATACTATCAAGCGAACCACAGTCAAAACGCCCTCCTGGGCGGTGGACATGATGAGATTCAATATTAATGAC
TTTCTTCCCCCAGGAGGGGGCTCAAACCCCCGCTCTGTGCCCTTTGAATACTACAGAATAAGAAAGGTTAAGGTTGAA
TTCTGGCCCTGCTCCCCGATCACCCAGGGTGACAGGGGAGTGGGCTCCAGTGCTGTTATTCTAGATGATAACTTTGTA
ACAAAGGCCACAGCCCTCACCTATGACCCCTATGTAAACTACTCCTCCCGCCATACCATAACCCAGCCCTTCTCCTACCA
CTCCCGCTACTTTACCCCAAACCTGTCCTAGATTCCACTATTGATTACTTCCAACCAAACAGCAAAAGAAATCAGCTGT
GGCTGAGACTACAAACTGCTGGAAATGTAGACCACGTAGGCCTCGGCACTGCGTTCGAAAACAGTATATACGACCAG
GAATACAATATCCGTGTAACCATGTATGTACAATTCAGAGAATTTAATCTTAAAGACCCCCCACTTAACCCT--------

>JQ866915_pcv2b

ATGACGTATCCAAGGAGGCGTTACCGGAGAAGAAGACACCGCCCCCGCAGCCATCTTGGCCAGATCCTCCGCCGCCG
CCCCTGGCTCGTCCACCCCCGCCACCGTTACCGCTGGAGAAGGAAAAATGGCATCTTCAACACCCGCCTCTCCCGCACC
TTCGGATATACTATCAAGCGAACCACAGTCAAAACGCCCTCCTGGGCGGTGGACATGATGAGATTCAATATTAATGAC
TTTCTTCCCCCAGGAGGGGGCTCAAACCCCCGCTCTGTGCCCTTTGAATACTACAGAATAAGAAAGGTTAAGGTTGAA
TTCTGGCCCTGCTCCCCGATCACCCAGGGTGACAGGGGAGTGGGCTCCAGTGCTGTTATTCTAGATGATAACTTTGTA
ACAAAGGCCACAGCCCTCACCTATGACCCCTATGTAAACTACTCCTCCCGCCATACCATAACCCAGCCCTTCTCCTACCA
CTCCCGCTACTTTACCCCAAACCTGTCCTAGATTCCACTATTGATTACTTCCAACCAAACAACAAAAGAAATCAGCTGT
GGCTGAGACTACAAACTGCTGGAAATGTAGACCACGTAGGCCTCGGCACTGCGTTCGAAAACAGTATATACGACCAG
GAATACAATATCCGTGTAACCATGTATGTACAATTCAGAGAATTTAATCTTAAAGACCCCCCACTTAACCCT--------

>EU136712_pcv2b

ATGACGTATCCAAGGAGGCGTTACCGGAGAAGAAGACACCGCCCCCGCAGCCATCTTGGCCAGATCCTCCGCCGCCG
CCCCTGGCTCGTCCACCCCCGCCACCGTTACCGCTGGAGAAGGAAAAATGGCATCTTCAACACCCGCCTCTCCCGCACC
TTCGGATATACTATCAAGCGAACCACAGTCAAAACGCCCTCCTGGGCGGTGGACATGATGAGATTCAATATTAATGAC
TTTCTTCCCCCAGGAGGGGGCTCAAACCCCCGCTCTGTGCCCTTTGAATACTACAGAATAAGAAAGGTTAAGGTTGAA
TTCTGGCCCTGCTCCCCGATCACCCAGGGTGACAGGGGAGTGGGCTCCAGTGCTGTTATTCTAGATGATAACTTTGTA
ACAAAGGCCACAGCCCTCACCTATGACCCCTATGTAAACTACTCCTCCCGCCATACCATAACCCAGCCCTTCTCCTACCA
CTCCCGCTACTTTACCCCAAACCTGTCCTAGATTCCACTATTGATTACTTCCAACCAAACAACAAAAGAAATCAGCTGT
GGCTGAGACTACAAACTGCTGGAAATGTAGACCACGTAGGCCTCGGCACTGCGTTCGAAAACAGTATATACGACCAG
GAATACAATATCCGTGTAACCATGTATGTACAATTCAGAGAATTTAATCTTAAAGACCCCCCACTTAACCCT--------

>EU136714_pcv2b

ATGACGTATCCAAGGAGGCGTTACCGGAGAAGAAGACACCGCCCCCGCAGCCATCTTGGCCAGATCCTCCGCCGCCG
CCCCTGGCTCGTCCACCCCCGCCACCGTTACCGCTGGAGAAGGAAAAATGGCATCTTCAACACCCGCCTCTCCCGCACC
TTCGGATATACTATCAAGCGAACCACAGTCAAAACGCCCTCCTGGGCGGTGGACATGATGAGATTCAATATTAATGAC
TTTCTTCCCCCAGGAGGGGGCTCAAACCCCCGCTCTGTGCCCTTTGAATACTACAGAATAAGAAAGGTTAAGGTTGAA
TTCTGGCCCTGCTCCCCGATCACCCAGGGTGACAGGGGAGTGGGCTCCAGTGCTGTTATTCTAGATGATAACTTTGTA
ACAAAGGCCACAGCCCTCACCTATGACCCCTATGTAAACTACTCCTCCCGCCATACCATAACCCAGCCCTTCTCCTACCA
CTCCCGCTACTTTACCCCCAAACCTGTCCTAGATTCCACTATTGATTACTTCCAACCAAACAACAAAAGAAATCAGCTGT
GGCTGAGACTACAAACTGCTGGAAATGTAGACCACGTAGGCCTCGGCACTGCGTTCGAAAACAGTATATACGACCAG
GAATACAATATCCGTGTAACCATGTATGTACAATTCAGAGAATTTAATCTTAAAGACCCCCCACTTAACCCT--------

>HQ831530_pcv2b

ATGACGTATCCAAGGAGGCGTTACCGGAGAAGAAGACACCGCCCCCGCAGCCATCTTGGCCAGATCCTCCGCCGCCG
CCCCTGGCTCGTCCACCCCCGCCACCGTTACCGCTGGAGAAGGAAAAATGGCATCTTCAACACCCGCCTCTCCCGCACC
TTCGGATATACTATCAAGCGAACCACAGTCAAAACGCCCTCCTGGGCGGTGGACATGATGAGATTCAATATTAATGAC
TTTCTTCCCCCAGGAGGGGGCTCAAACCCCCGCTCTGTGCCCTTTGAATACTACAGAATAAGAAAGGTTAAGGTTGAA
TTCTGGCCCTGCTCCCCGATCACCCAGGGTGACAGGGGAGTGGGCTCCAGTGCTGTTATTCTAGATGATAACTTTGTA
ACAAAGGCCACAGCCCTCACCTATGACCCCTATGTAAACTACTCCTCCCGCCATACCATAACCCAGCCCTTCTCCTACCA
CTCCCGCTACTTTACCCCCAAACCTGTCCTAGATTCCACTATTGATTACTTCCAACCAAACAACAAAAGAAATCAGCTGT
GGCTGAGACTACAAACTGCTGGAAATGTAGACCACGTAGGCCTCGGCACTGCGTTCGAAAACAGTATATACGACCAG
GAATACAATATCCGTGTAACCATGTATGTACAATTCAGAGAATTTAATCTTAAAGACCCCCCACTTAACCCT--------

>EF565354_pcv2b

ATGACGTATCCAAGGAGGCGTTACCGGAGAAGAAGACACCGCCCCCGCAGCCATCTTGGCCAGATCCTCCGCCGCCG
CCCCTGGCTCGTCCACCCCCGCCACCGTTACCGCTGGAGAAGGAAAAATGGCATCTTCAACACCCGCCTCTCCCGCACC
TTCGGATATACTATCAAGCGAACCACAGTCAAAACGCCCTCCTGGGCGGTGGACATGATGAGATTCAATATTAATGAC
TTTCTTCCCCCAGGAGGGGGCTCAAACCCCCGCTCTGTGCCCTTTGAATACTACAGAATAAGAAAGGTTAAGGTTGAA
TTCTGGCCCTGCTCCCCGATCACCCAGGGTGACAGGGGAGTGGGCTCCAGTGCTGTTATTCTAGATGATAACTTTGTA
ACAAAGGCCACAGCCCTCACCTATGACCCCTATGTAAACTACTCCTCCCGCCATACCATAACCCAGCCCTTCTCCTACCA
CTCCCGCTACTTTACCCCCAAACCTGTCCTAGATTCCACTATTGATTACTTCCAACCAAACAACAAAAGAAATCAGCTGT
GGCTGAGACTACAAACTGCTGGAAATGTAGACCACGTAGGCCTCGGCACTGCGTTCGAAAACAGTATATACGACCAG
GAATACAATATCCGTGTAACCATGTATGTACAATTCAGAGAATTTAATCTTAAAGACCCCCCACTTAACCCT--------

>EU780074_pcv2b

ATGACGTATCCAAGGAGGCGTTACCGGAGAAGAAGACACCGCCCCCGCAGCCATCTTGGCCAGATCCTCCGCCGCCG
CCCCTGGCTCGTCCACCCCCGCCACCGTTACCGCTGGAGAAGGAAAAATGGCATCTTCAACACCCGCCTCTCCCGCACC
TTCGGATATACTATCAAGCGAACCACAGTCAAAACGCCATCCTGGGCGGTGGACATGATGAGATTCAATATTAATGAC
TTTCTTCCCCCAGGAGGGGGCTCAAACCCCCGCTCGGTGCCCTTTGAATACTACAGAATAAGAAAGGTTAAGGTTGAA
TTCTGGCCCTGCTCCCCGATCACCCAGGGTGACAGGGGAGTGGGCTCCAGTGCTGTTATTCTAGATGATAACTTTGTA
ACAAAGGCCACAGCCCTCACCTATGACCCCTATGTAAACTACTCCTCCCGCCATACCATAACCCAGCCCTTCTCCTACCA
CTCCCGCTACTTTACCCCCAAACCTGTCCTAGATTCCACTATTGATTACTTCCAACCAAACAACAAAAGAAATCAGCTGT
GGCTGAGACTACAAACTGCTGGAAATGTAGACCACGTAGGCCTCGGCACTGCGTTCGAAAACAGTATATACGACCAG
GAATACAATATCCGTGTAACCATGTATGTACAATTCAGAGAATTTAATCTTAAAGACCCCCCACTTAACCCT--------

>AY322000_pcv2b

ATGACGTATCCAAGGAGGCGTTACCGGAGAAGAAGACACCGCCCCCGCAGCCATCTTGGCCAGATCCTCCGCCGCCG
CCCCTGGCTCGTCCACCCCCGCCACCGTTACCGCTGGAGAAGGAAAAATGGCATCTTCAACACCCGCCTCTCCCGCACC
TTCGGATATACTATCAAGCGAACCACAGTCAAAACGCCCTCCTGGGCGGTGGACATGATGAGATTCAATATTAATGAC
TTTCTTCCCCCAGGAGGGGGCTCAAACCCCCGCTCTGTGCCCTTTGAATACTACAGAATAAGAAAGGTTAAGGTTGAA
TTCTGGCCCTGCTCCCCGATCACCCAGGGTGACAGGGGAGTGGGCTCCAGTGCTGTTATTCTAGATGATAACTTTGTA
ACAAAGGCCACAGCCCTCACCTATGACCCCTATGTAAACTACTCCTCCCGCCATACCATAACCCAGCCCTTCTCCTACCA
CTCCCGCTACTTTACCCCCAAACCTGTCCTAGATTCCACTATTGATTACTTCCAACCAAACAACAAAAGAAATCAGCTGT
GGCTGAGACTACAAACTGCTGGAAATGTAGACCACGTAGGCCTCGGCACTGCGTTCGAAAACAGTATATACGACCAG
GAATACAATATCCGTGTAACCATGTATGTACAATTCAGAGAATTTAATCTTAAAGACCCCCCACTTAACCCT--------

>EF565342_pcv2b

ATGACGTATCCAAGGAGGCGTTACCGGAGAAGAAGACACCGCCCCCGCAGCCATCTTGGCCAGATCCTCCGCCGCCG
CCCCTGGCTCGTCCACCCCCGCCACCGTTACCGCTGGAGAAGGAAAAATGGCATCTTCAACACCCGCCTCTCCCGCACC
TTCGGATATACTATCAAGCGAACCACAGTCAAAACGCCCTCCTGGGCGGTGGACATGATGAGATTCAATATTAATGAC
TTTCTTCCCCCAGGAGGGGGCTCAAACCCCCGCTCTGTGCCCTTTGAATACTACAGAATAAGAAAGGTTAAGGTTGAA
TTCTGGCCCTGCTCCCCGATCACCCAGGGTGACAGGGGAGTGGGCTCCAGTGCTGTTATTCTAGATGATAACTTTGTA
ACAAAGGCCACAGCCCTCACCTATGACCCCTATGTAAACTACTCCTCCCGACATACCATAACCCAGCCCTTCTCCTACCA
CTCCCGCTACTTTACCCCCAAACCTGTCCTAGATTCCACTATTGATTACTTCCAACCGAACAACAAAAGAAATCAGCTGT
GGCTGAGACTACAAACTGCTGGAAATGTAGACCACGTAGGCCTCGGCACTGCGTTCGAAAACAGTATATACGACCAG
GAATACAATATCCGTGTAACCATGTATGTACAATTCAGAGAATTTAATCTTAAAGACCCCCCACTTAACCCT--------

>AB462391_pcv2b

ATGACGTATCCAAGGAGGCGTTACCGGAGAAGAAGACACCGCCCCCGCAGCCATCTTGGCCAGATCCTCCGCCGCCG
CCCCTGGCTCGTCCACCCCCGCCACCGTTACCGCTGGAGAAGGAAAAATGGCATCTTCAACACCCGCCTCTCCCGCACC
TTCGGATATACTATCAAGCGAACCACAGTCAAAACGCCCTCCTGGGCGGTGGACATGATGAGATTCAATATTAATGAC
TTTCTTCCCCCAGGAGGGGGCTCAAACCCCCGCTCTGTGCCCTTTGAATACTACAGAATAAGAAAGGTTAAGGTTGAA
TTCTGGCCCTGCTCCCCGATCACCCAGGGTGACAGGGGAGTGGGCTCCAGTGCTGTTATTCTAGATGATAACTTTGTA
ACAAAGGCCACAGCCCTCACCTATGACCCCTATGTAAACTACTCCTCCCGCCATACCATAACCCAGCCCTTCTCCTACCA
CTCCCGCTACTTTACCCCCAAACCTGTCCTAGATTCCACTATTGATTACTTCCAACCTAACAACAAAAGAAATCAGCTGT
GGCTGAGACTACAAACTGCTGGAAATGTAGACCACGTAGGCCTCGGCACTGCGTTCGAAAACAGTATATACGACCAG
GAATACAATATCCGTGTAACCATGTATGTACAATTCAGAGAATTTAATCTTAAAGACCCCCCACTTAACCCT--------

>EF371542_pcv2b

ATGACGTATCCAAGGAGGCGTTACCGGAGAAGAAGACACCGCCCCCGCAGCCATCTTGGCCAGATCCTCCGCCGCCG
CCCCTGGCTCGTCCACCCCCGCCACCGTTACCGCTGGAGAAGGAAAAATGGCATCTTCAACACCCGCCTCTCCCGCACC
TTCGGATATACTATCAAGCGAACCACAGTCAAAACGCCCTCCTGGGCGGTGGACATGATGAGATTCAATATTAATGAC
TTTCTTCCCCCAGGAGGGGGCTCAAACCCCCGCTCTGTGCCCTTTGAATACTACAGAATAAGAAAGGTTAAGGTTGAA
TTCTGGCCCTGCTCCCCGATCACCCAGGGTGACAGGGGAGTGGGCTCCAGTGCTGTTATTCTAGATGATAACTTTGTA
ACAAAGGCCACAGCCCTCACCTATGACCCCTATGTAAACTACTCCTCCCGCCATACCATAACCCAGCCCTTCTCCTACCA
CTCCCGCTACTTTACCCCCAAACCTGTCCTAGATTCCACTATTGATTACTTCCAACCAAACAACAAAAGAAATCAGCTGT
GGCTGAGACTACAAACTGCTGGAAATGTAGACCACGTAGGCCTCGGCACTGCGTTCGAAAACAGTATATACGACCAG
GAATACAATATCCGTGTAACCATGTATGTACAATTCAGAGAATTTAATCTTAAAGACCCCCCACTTAACCCT--------

>DQ233257_pcv2b

ATGACGTATCCAAGGAGGCGTTACCGGAGAAGAAGACACCGCCCCCGCAGCCATCTTGGCCAGATCCTCCGCCGCCG
CCCCTGGCTCGTCCACCCCCGCCACCGTTACCGCTGGAGAAGGAAAAATGGCATCTTCAACACCCGCCTCTCCCGCACC
TTCGGATATACTATCAAGCGAACCACAGTCAAAACGCCCTCCTGGGCGGTGGACATGATGAGATTCAATATTAATGAC
TTTCTTCCCCCAGGAGGGGGCTCAAACCCCCGCTCTGTGCCCTTTGAATACTACAGAATAAGAAAGGTTAAGGTTGAA
TTCTGGCCCTGCTCCCCGATCACCCAGGGTGACAGGGGAGTGGGCTCCAGTGCTGTTATTCTAGATGATAACTTTGTA
ACAAAGGCCACAGCCCTCACCTATGACCCCTATGTAAACTACTCCTCCCGCCATACCATAACCCAGCCCTTCTCCTACCA
CTCCCGCTACTTTACCCCCAAACCTGTCCTAGATTCCACTATTGATTACTTCCAACCAAACAACAAAAGAAATCAGCTGT
GGCTGAGACTACAAACTGCTGGAAATGTAGACCACGTAGGCCTCGGCACTGCGTTCGAAAACAGTATATACGACCAG
GAATACAATATCCGTGTAACCATGTATGTACAATTCAGAGAATTTAATCTTAAAGACCCCCCACTTAACCCT--------

>AY181945_pcv2b

ATGACGTATCCAAGGAGGCGTTACCGGAGAAGAAGACACCGCCCCCGCAGCCATCTTGGCCAGATCCTCCGCCGCCG
CCCCTGGCTCGTCCACCCCCGCCACCGTTACCGCTGGAGAAGGAAAAATGGCATCTTCAACACCCGCCTCTCCCGCACC
TTCGGATATACTATCAAGCGAACCACAGTCAAAACGCCCTCCTGGGCGGTGGACATGATGAGATTCAATATCAATGAC
TTTCTTCCCCCAGGAGGGGGCTCAAACCCCCGCTCTGTGCCCTTTGAATACTACGGAATAAGAAAGGTTAAGGTTGAA
TTCTGGCCCTGCTCCCCGATCACCCAGGGTGACAGGGGAGTGGGCTCCAGTGCTGTTATTCTAGATGATAACTTTGTA
ACAAAGGCCACAGCCCTCACCTATGACCCCTATGTAAACTACTCCTCCCGCCATACCATAACCCAGCCCTTCTCCTACCA
CTCCCGCTACTTTACCCCCAAACCTGTCCTAGATTCCACTATTGATTACTTCCAACCAAACAACAAAAGAAATCAGCTGT
GGCTGAGACTACAAACTGCTGGAAATGTAGACCACGTAGGCCTCGGCACTGCGTTCGAAAACAGTATATACGACCAG
GAATACAATATCCGTGTAACCATGTATGTACAATTCAGAGAATTTAATCTTAAAGACCCCCCACTTAACCCT--------

>EU545546_pcv2b

ATGACGTATCCAAGGAGGCGTTACCGGAGAAGAAGACACCGCCCCCGCAGCCATCTTGGCCAGATCCTCCGCCGCCG
CCCCTGGCTCGTCCACCCCCGCCACCGTTACCGCTGGAGAAGGAAAAATGGCATCTTCAACACCCGCCTCTCCCGCACC
TTCGGATATACTATCAAGCGAACCACAGTCAAAACGCCCTCCTGGGCGGTGGACATGATGAGATTCAATATCAATGAC
TTTCTTCCCCCAGGAGGGGGCTCAAACCCCCGCTCTGTGCCCTTTGAATACTACAGAATAAGAAAGGTTAAGGTTGAA
TTCTGGCCCTGCTCCCCGATCACCCAGGGTGACAGGGGAGTGGGCTCCAGTGCTGTTATTCTAGATGATAACTTTGTA
ACAAAGGCCACAGCCCTCACCTATGACCCCTATGTAAACTACTCCTCCCGCCATACCATAACCCAGCCCTTCTCCTACCA
CTCCCGCTACTTTACCCCCAAACCTGTCCTAGATTCCACTATTGATTACTTCCAACCAAACAACAAAAGAAATCAGCTGT
GGCTGAGACTACAAACTGCTGGAAATGTAGACCACGTAGGCCTCGGCACTGCGTTCGAAAACAGTATATACGACCAG
GAATACAATATCCGTGTAACCATGTATGTACAATTCAGAGAATTTAATCTTAAAGACCCCCCACTTAACCCT--------

>EU136719_pcv2b

ATGACGTATCCAAGGAGGCGTTACCGGAGAAGAAGACACCGCCCCCGCAGCCATCTTGGCCAGATCCTCCGCCGCCG
CCCCTGGCTCGTCCACCCCCGCCACCGTTACCGCTGGAGAAGGAAAAATGGCATCTTCAACACCCGCCTCTCCCGCACC
TTCGGATATACTATCAAGCGAACCACAGTCAAAACGCCCTCCTGGGCGGTGGACATGATGAGATTCAATATTAATGAC
TTTCTTCCCCCAGGAGGGGGCTCAAACCCCCGCTCTGTGCCCTTTGAATACTACAGAATAAGAAAGGTTAAGGTTGAA
TTCTGGCCCTGCTCCCCGATCACCCAGGGTGACAGGGGAGTGGGCTCCAGTGCTGTTATTCTAGATGATAACTTTGTA
ACAAAGGCCACAGCCCTCACCTATGACCCCTATGTAAACTACTCCTCCCGCCATACCATAACCCAGCCCTTCTCCTACCA
CTCCCGCTACTTTACCCCCAAACCTGTCCTAGATTCCACTATTGATTACTTCCAACCAAACAACAAAAGAAATCAGCTGT
GGCTGAGACTACAAACTGCTGGAAATGTAGACCACGTAGGCCTCGGCACTGCGTTCGAAAACAGTATATACGACCAG
GAATACAATATCCGTGTAACCATGTATGTACAATTCAGAGAATTTAATCTTAAAGACCCCCCACTTAACCCT--------

>FJ644563_pcv2b

ATGACGTATCCAAGGAGGCGTTACCGGAGAAGAAGACACCGCCCCCGCAGCCATCTTGGCCAGATCCTCCGCCGCCG
CCCCTGGCTCGTCCACCCCCGCCACCGTTACCGCTGGAGAAGGAAAAATGGCATCTTCAACACCCGCCTCTCCCGCACC
TTCGGATATACTATCAAGCGAACCACAGTCAAAACGCCCTCCTGGGCGGTGGACATGATGAGATTCAATATTAATGAC
TTTCTTCCCCCAGGAGGGGGCTCAAACCCCCGCTCTGTGCCCTTTGAATACTACAGAATAAGAAAGGTTAAGGTTGAA
TTCTGGCCCTGCTCCCCGATCACCCAGGGTGACAGGGGAGTGGGCTCCAGTGCTGTTATTCTAGATGATAACTTTGTA
ACAAAGGCCACAGCCCTCACCTATGACCCCTATGTAAACTACTCCTCCCGCCATACCATAACCCAGCCCTTCTCCTACCA
CTCCCGCTACTTTACCCCCAAACCTGTCCTAGATTCCACTATTGATTACTTCCAACCAAACAACAAAAGAAATCAGCTGT
GGCTGAGACTACAAACTGCTGGAAATGTAGACCACGTAGGCCTCGGCACTGCGTTCGAAAACAGTATATACGACCAG
GAATACAATATCCGTGTAACCATGTATGTACAATTCAGAGAATTTAATCTTAAAGACCCCCCACTTAACCCT--------

>EU136713_pcv2b

ATGACGTATCCAAGGAGGCGTTACCGGAGAAGAAGACACCGCCCCCGCAGCCATCTTGGCCAGATCCTCCGCCGCCG
CCCCTGGCTCGTCCACCCCCGCCACCGTTACCGCTGGAGAAGGAAAAATGGCATCTTCAACACCCGCCTCTCCCGCACC
TTCGGATATACTATCAAGCGAACCACAGTCAAAACGCCCTCCTGGGCGGTGGACATGATGAGATTCAATATTAATGAC
TTTCTTCCCCCAGGAGGGGGCTCAAACCCCCGCTCTGTGCCCTTTGAATACTACAGAATAAGAAAGGTTAAGGTTGAA
TTCTGGCCCTGCTCCCCGATCACCCAGGGTGACAGGGGAGTGGGCTCCAGTGCTGTTATTCTAGATGATAACTTTGTA
ACAAAGGCCACAGCCCTCACCTATGACCCCTATGTAAACTACTCCTCCCGCCATACCATAACCCAGCCCTTCTCCTACCA
CTCCCGCTACTTTACCCCCAAACCTGTCCTAGATTCCACTATTGATTACTTCCAACCAAACAACAAAAGAAATCAGCTGT
GGCTGAGACTACAAACTGCTGGAAATGTAGACCACGTAGGCCTCGGCACTGCGTTCGAAAACAGTATATACGACCAG
GAATACAATATCCGTGTAACCATGTATGTACAATTCAGAGAATTTAATCTTAAAGACCCCCCACTTAACCCT--------

>JQ866914_pcv2b

ATGACGTATCCAAGGAGGCGTTACCGGAGAAGAAGACACCGCCCCCGCAGCCATCTTGGCCAGATCCTCCGCCGCCG
CCCCTGGCTCGTCCACCCCCGCCACCGTTACCGCTGGAGAAGGAAAAATGGCATCTTCAACACCCGCCTCTCCCGCACC
TTCGGATATACTATCAAGCGAACCACAGTCAAAACGCCCTCCTGGGCGGTGGACATGATGAGATTCAATATTAATGAC
TTTCTTCCCCCAGGAGGGGGCTCAAACCCCCGCTCTGTGCCCTTTGAATACTACAGAATAAGAAAGGTTAAGGTTGAA
TTCTGGCCCTGCTCCCCGATCACCCAGGGTGACAGGGGAGTGGGCTCCAGTGCTGTTATTCTAGATGATAACTTTGTA
ACAAAGGCCACAGCCCTCACCTATGACCCCTATGTAAACTACTCCTCCCGCCATACCATAACCCAGCCCTTCTCCTACCA
CTCCCGCTACTTTACCCCCAAACCTGTCCTAGATTCCACTATTGATTACTTCCAACCAAACAACAAAAGAAATCAGCTGT
GGCTGAGACTACAAACTGCTGGAAATGTAGACCACGTAGGCCTCGGCACTGCGTTCGAAAACAGTATATACGACCAG
GAATACAATATCCGTGTAACCATGTATGTACAATTCAGAGAATTTAATCTTAAAGACCCCCCACTTAACCCT--------

>EF190934_pcv2b

ATGACGTATCCAAGGAGGCGTTACCGGAGAAGAAGACACCGCCCCCGCAGCCATCTTGGCCAGATCCTCCGCCGCCG
CCCCTGGCTCGTCCACCCCCGCCACCGTTACCGCTGGAGAAGGAAAAATGGCATCTTCAACACCCGCCTCTCCCGCACC
TTCGGATATACTATCAAGCGAACCACAGTCAAAACGCCCTCCTGGGCGGTGGACATGATGAGATTCAATATTAATGAC
TTTCTTCCCCCAGGAGGGGGCTCAAACCCCCGCTCTGTGCCCTTTGAATACTACAGAATAAGAAAGGTTAAGGTTGAA
TTCTGGCCCTGCTCCCCGATCACCCAGGGTGACAGGGGAGTGGGCTCCAGTGCTGTTATTCTAGATGATAACTTTGTA
ACAAAGGCCACAGCCCTCACCTATGACCCCTATGTAAACTACTCCTCCCGCCATACCATAACCCAGCCCTTCTCCTACCA
CTCCCGCTACTTTACCCCCAAACCTGTCCTAGATTCCACTATTGATTACTTCCAACCAAACAACAAAAGAAATCAGCTGT
GGCTGAGACTACAAACTGCTGGAAATGTAGACCACGTAGGCCTCGGCACTGCGTTCGAAAACAGTATATACGACCAG
GAATACAATATCCGTGTAACCATGTATGTACAATTCAGAGAATTTAATCTTAAAGACCCCCCACTTAACCCT--------

>FJ660968_pcv2b

ATGACGTATCCAAGGAGGCGTTACCGGAGAAGAAGACACCGCCCCCGCAGCCATCTTGGCCAGATCCTCCGCCGCCG
CCCCTGGCTCGTCCACCCCCGCCACCGTTACCGCTGGAGAAGGAAAAATGGCATCTTCAACACCCGCCTCTCCCGCACC
TTCGGATATACTATCAAGCGAACCACAGTCAAAACGCCCTCCTGGGCGGTGGACATGATGAGATTCAATATTAATGAC
TTTCTTCCCCCAGGAGGGGGCTCAAACCCCCGCTCTGTGCCCTTTGAATACTACAGAATAAGAAAGGTTAAGGTTGAA
TTCTGGCCCTGCTCCCCGATCACCCAGGGTGACAGGGGAGTGGGCTCCAGTGCTGTTATTCTAGATGATAACTTTGTA
ACAAAGGCCACAGCCCTCACCTATGACCCCTATGTAAACTACTCCTCCCGCCATACCATAACCCAGCCCTTCTCCTACCA
CTCCCGCTACTTTACCCCCAAACCTGTCCTAGATTCCACTATTGATTACTTCCAACCAAACAACAAAAGAAATCAGCTGT
GGCTGAGACTACAAACTGCTGGAAATGTAGACCACGTAGGCCTCGGCACTGCGTTCGAAAACAGTATATACGACCAG
GAATACAATATCCGTGTAACCATGTATGTACAATTCAGAGAATTTAATCTTAAAGACCCCCCACTTAACCCTAAG-----

>EF371532_pcv2b

ATGACGTATCCAAGGAGGCGTTACCGGAGAAGAAGACACCGCCCCCGCAGCCATCTTGGCCAGATCCTCCGCCGCCG
CCCCTGGCTCGTCCACCCCCGCCACCGTTACCGCTGGAGAAGGAAAAATGGCATCTTCAACACCCGCCTCTCCCGCACC
TTCGGATATACTATCAAGCGAACCACAGTCAAAACGCCCTCCTGGGCGGTGGACATGATGAGATTCAATATTAATGAC
TTTCTTCCCCCAGGAGGGGGCTCAAACCCCCGCTCTGTGCCCTTTGAATACTACAGAATAAGAAAGGTTAAGGTTGAA
TTCTGGCCCTGCTCCCCGATCACCCAGGGTGACAGGGGAGTGGGCTCCAGTGCTGTTATTCTAGATGATAACTTTGTA
ACAAAGGCCACAGCCCTCACCTATTACCCCTATGTAAACTACTCCTCCCGCCATACCATAACCCAGCCCTTCTCCTACCA
CTCCCGCTACTTTACCCCCAAACCTGTCCTAGATTCCACTATTGATTACTTCCAACCAAACAACAAAAGAAATCAGCTGT
GGCTGAGACTACAAACTGCTGGAAATGTAGACCACGTAGGCCTCGGCACTGCGTTCGAAAACAGTATATACGACCAG
GAATACAATATCCGTGTAACCATGTATGTACAATTCAGAGAATTTAATCTTAAAAACCCCCCACTTTACCCT--------

>EF371531_pcv2b

ATGACGTATCCAAGGAGGCGTTACCGGAGAAGAAGACACCGCCCCCGCAGCCATCTTGGCCAGATCCTCCGCCGCCG
CCCCTGGCTCGTCCACCCCCGCCACCGTTACCGCTGGAGAAGGAAAAATGGCATCTTCAACACCCGCCTCTCCCGCACC
TTCGGATATACTATCAAGCGAACCACAGTCAAAACGCCCTCCTGGGCGGTGGACATGATGAGATTCAATATTAATGAC
TTTCTTCCCCCAGGAGGGGGCTCAAACCCCCGCTCTGTGCCCTTTGAATACTACAGAATAAGAAAGGTTAAGGTTGAA
TTCTGGCCCTGCTCCCCGATCACCCAGGGTGACAGGGGAGTGGGCTCCAGTGCTGTTATTCTAGATGATAACTTTGTA
ACAAAGGCCACAGCCCTCACCTATTACCCCTATGTAAACTACTCCTCCCGCCATACCATAACCCAGCCCTTCTCCTACCA
CTCCCGCTACTTTACCCCCAAACCTGTCCTAGATTCCACTATTGATTACTTCCAACCAAACAACAAAAGAAATCAGCTGT
GGCTGAGACTACAAACTGCTGGAAATGTAGACCACGTAGGCCTCGGCACTGCGTTCGAAAACAGTATATACGACCAG
GAATACAATATCCGTGTAACCATGTATGTACAATTCAGAGAATTTAATCTTAAAAACCCCCCACTTTACCCT--------

>EF371530_pcv2b

ATGACGTATCCAAGGAGGCGTTACCGGAGAAGAAGACACCGCCCCCGCAGCCATCTTGGCCAGATCCTCCGCCGCCG
CCCCTGGCTCGTCCACCCCCGCCACCGTTACCGCTGGAGAAGGAAAAATGGCATCTTCAACACCCGCCTCTCCCGCACC
TTCGGATATACTATCAAGCGAACCACAGTCAAAACGCCCTCCTGGGCGGTGGACATGATGAGATTCAATATTAATGAC
TTTCTTCCCCCAGGAGGGGGCTCAAACCCCCGCTCTGTGCCCTTTGAATACTACAGAATAAGAAAGGTTAAGGTTGAA
TTCTGGCCCTGCTCCCCGATCACCCAGGGTGACAGGGGAGTGGGCTCCAGTGCTGTTATTCTAGATGATAACTTTGTA
ACAAAGGCCACAGCCCTCACCTATTACCCCTATGTAAACTACTCCTCCCGCCATACCATAACCCAGCCCTTCTCCTACCA
CTCCCGCTACTTTACCCCCAAACCTGTCCTAGATTCCACTATTGATTACTTCCAACCAAACAACAAAAGAAATCAGCTGT
GGCTGAGACTACAAACTGCTGGAAATGTAGACCACGTAGGCCTCGGCACTGCGTTCGAAAACAGTATATACGACCAG
GAATACAATATCCGTGTAACCATGTATGTACAATTCAGAGAATTTAATCTTAAAAACCCCCCACTTTACCCT--------

>AB462390_pcv2b

ATGACGTATCCAAGGAGGCGTTACCGGAGAAGAAGACACCGCCCCCGCAGCCATCTTGGCCAGATCCTCCGCCGCCG
CCCCTGGCTCGTCCACCCCCGCCACCGTTACCGCTGGAGAAGGAAAAATGGCATCTTCAACACCCGCCTCTCCCGCACC
TTCGGATATACTATCAAGCGAACCACAGTCAAAACGCCCTCCTGGGCGGTGGACATGATGAGATTCAATATTAATGAC
TTTCTTCCCCCAGGAGGGGGCTCAAACCCCCGCTCTGTGCCCTTTGAATACTACAGAATAAGAAAGGTTAAGGTTGAA
TTCTGGCCCTGCTCCCCGATCACCCAGGGTGACAGGGGAGTGGGCTCCAGTGCTGTTATTCTAGATGATAACTTTGTA
ACAAAGGCCACAGCCCTCACCTATGACCCCTATGTAAACTACTCCTCCCGCCATACCATAACCCAGCCCTTCTCCTACCA
CTCCCGCTACTTTACCCCCAAACCTGTCCTAGATTCCACTATTGATTACTTCCAACCAAACAACAAAAGAAATCAGCTGT
GGCTGAGACTACAAACTGCTGGAAATGTAGACCACGTAGGCCTCGGCACTGCGTTCGAAAACAGTATATACGACCAG
GAATACAATATCCGTGTAACCATGTATGTACAATTCAGAGAATTTAATCTTAAAGACCCCCCACTTAACCCT--------

>AY321996_pcv2b

ATGACGTATCCAAGGAGGCGTTACCGGAGAAGAAGACACCGCCCCCGCAGCCATCTTGGCCAGATCCTCCGCCGCCG
CCCCTGGCTCGTCCACCCCCGCCACCGTTACCGCTGGAGAAGGAAAAATGGCATCTTCAACACCCGCCTCTCCCGCACC
TTCGGATATACTATCAAGCGAACCACAGTCAAAACGCCCTCCTGGGCGGTGGACATGATGAGATTCAATATTAATGAC
TTTCTTCCCCCAGGAGGGGGCTCAAACCCCCGCTCTGTGCCCTTTGAATACTACAGAATACGAAAGGTTAAGGTTGAA
TTCTGGCCCTGCTCCCCGATCACCCAGGGTGACAGGGGAGTGGGCTCCAGTGCTGTTATTCTAGATGATAACTTTGTA
ACAAAGGCCACAGCCCTCACCTATGACCCCTATGTAAACTACTCCTCCCGCCATACCATAACCCAGCCCTTCTCCTACCA
CTCCCGCTACTTTACCCCCAAACCTGTCCTAGATTCCACTATTGATTACTTCCAACCAAACAACAAAAGAAATCAGCTGT
GGCTGAGACTACAAACTGCTGGAAATGTAGACCACGTAGGCCTCGGCACTGCGTTCGAAAACAGTATATACGACCAG
GAATACAATATCCGTGTAACCATGTATGTACAATTCAGAGAATTTAATCTTAAAGACCCCCCACTTAACCCT--------

>HQ591368_pcv2b

ATGACGTATCCAAGGAGGCGTTACCGGAGAAGAAGACACCGCCCCCGCAGCCATCTTGGCCAGATCCTCCGCCGCCG
CCCCTGGCTCGTCCACCCCCGCCACCGTTACCGCTGGAGAAGGAAAAATGGCATCTTCAACACCCGCCTCTCCCGCACC
TTCGGATATACTATCAAGCGAACCACAGTCAAAACGCCCTCCTGGGCGGTGGACATGATGAGATTCAATATTAATGAC
TTTCTTCCCCCAGGAGGGGGCTCAAACCCCCGCTCTGTGCCCTTTGAATACTACAGAATAAGAAAGGTTAAGGTTGAA
TTCTGGCCCTGCTCCCCGATCACCCAGGGTGACAGGGGAGTGGGCTCCAGTGCTGTTATTCTAGATGATAACTTTGTA
ACAAAGGCCACAGCCCTCACCTATGACCCCTATGTAAACTACTCCTCCCGCCATACCATAACCCAGCCCTTCTCCTACCA
CTCCCGCTACTTTACCCCCAAACCTGTCCTAGATTCCACTATTGATTACTTCCAACCAAACAACAAAAGAAATCAGCTGT
GGCTGAGACTACAAACTGCTGGAAATGTAGACCACGTAGGCCTCGGCACTGCGTTCGAAAACAGTATATACGACCAG
GAATACAATATCCGTGTAACCATGTATGTACAATTCAGAGAATTTAATCTTAAAGACCCCCCACTTAACCCT--------

>HQ591371_pcv2b

ATGACGTATCCAAGGAGGCGTTACCGGAGAAGAAGACACCGCCCCCGCAGCCATCTTGGCCAGATCCTCCGCCGCCG
CCCCTGGCTCGTCCACCCCCGCCACCGTTACCGCTGGAGAAGGAAAAATGGCATCTTCAACACCCGCCTCTCCCGCACC
TTCGGATATACTATCAAGCGAACCACAGTCAAAACGCCCTCCTGGGCGGTGGACATGATGAGATTCAATATTAATGAC
TTTCTTCCCCCAGGAGGGGGCTCAAACCCCCGCTCTGTGCCCTTTGAATACTACAGAATAAGAAAGGTTAAGGTTGAA
TTCTGGCCCTGCTCCCCGATCACCCAGGGTGACAGGGGAGTGGGCTCCAGTGCTGTTATTCTAGATGATAACTTTGTA
ACAAAGGCCACAGCCCTCACCTATGACCCCTATGTAAACTACTCCTCCCGCCATACCATAACCCAGCCCTTCTCCTACCA
CTCCCGCTACTTTACCCCCAAACCTGTCCTAGATTCCACTATTGATTACTTCCAACCAAACAACAAAAGAAATCAGCTGT
GGCTGAGACTACAAACTGCTGGAAATGTAGACCACGTAGGCCTCGGCACTGCGTTCGAAAACAGTATATACGACCAG
GAATACAATATCCGTGTAACCATGTATGTACAATTCAGAGAATTTAATCTTAAAGACCCCCCACTTAACCCT--------

>AY874168_pcv2b

ATGACGTATCCAAGGAGGCGTTACCGGAGAAGAAGACACCGCCCCCGCAGCCATCTTGGCCAGATCCTCCGCCGCCG
CCCCTGGCTCGTCCACCCCCGCCACCGTTACCGCTGGAGAAGGAAAAATGGCATCTTCAACACCCGCCTCTCCCGCACC
TTCGGATATACTATCAAGCGAACCACAGTCAAAACGCCCTCCTGGGCGGTGGACATGATGAGATTCAATATTAATGAC
TTTCTTCCCCCAGGAGGGGGCTCAAACCCCCGCTCTGTGCCCTTTGAATACTACAGAATAAGAAAGGTTAAGGTTGAA
TTCTGGCCCTGCTCCCCGATCACCCAGGGTGACAGGGGAGTGGGCTCCAGTGCTGTTATTCTAGATGATAACTTTGTA
ACAAAGGCCACAGCCCTCACCTATGACCCCTATGTAAACTACTCCTCCCGCCATACCATAACCCAGCCCTTCTCCTACCA
CTCCCGCTACTTTACCCCCAAACCTGTCCTAGATTCCACTATTGATTACTTCCAACCAAACAACAAAAGAAATCAGCTGT
GGCTGAGACTACAAACTGCTGGAAATGTAGACCACGTAGGCCTCGGCACTGCGTTCGAAAACAGTATATACGACCAG
GAATACAATATCCGTGTAACCATGTATGTACAATTCAGAGAATTTAATCTTAAAGACCCCCCACTTAACCCT--------

>AY288134_pcv2b

ATGACGTATCCAAGGAGGCGTTACCGGAGAAGAAGACACCGCCCCCGCAGCCATCTTGGCCAGATCCTCCGCCGCCG
CCCCTGGCTCGTCCACCCCCGCCACCGTTACCGCTGGAGAAGGAAAAATGGCATCTTCAACACCCGCCTCTCCCGCACC
TTCGGATATACTATCAAGCGAACCACAGTCAAAACGCCCTCCTGGGCGGTGGACATGATGAGATTCAATATTAATGAC
TTTCTTCCCCCAGGAGGGGGCTCAAACCCCCGCTCTGTGCCCTTTGAATACTACAGAATAAGAAAGGTTAAAGTTGAA
TTCTGGCCCTGCTCCCCGATCACCCAGGGTGACAGGGGAGTGGGCTCCAGTGCTGTTATTCTAGATGATAACTTTGTA
ACAAAGGCCACAGCCCTCACCTATGACCCCTATGTAAACTACTCCTCCCGCCATACCATAACCCAGCCCTTCTCCTACCA
CTCCCGCTACTTTACCCCCAAACCTGTCCTAGATTCCACTATTGATTACTTCCAACCAAACAACAAAAGAAATCAGCTGT
GGCTGAGACTACAAACTGCTGGAAATGTAGACCACGTAGGCCTCGGCACTGCGTTCGAAAACAGTATATACGACCAG
GAATACAATATCCGTGTAACCATGTATGTACAATTCAGAGAATTTAATCTTAAAGACCCCCCACTTAACCCT--------

>AY682992_pcv2b

ATGACGTATCCAAGGAGGCGTTACCGGAGAAGAAGACACCGCCCCCGCAGCCATCTTGGCCAGATCCTCCGCCGCCG
CCCCTGGCTCGTCCACCCCCGCCACCGTTACCGCTGGAGAAGGAAAAATGGCATCTTCAACACCCGCCTCTCCCGCACC
TTCGGATATACTATCAAGCGAACCACAGTCAAAACGCCCTCCTGGGCGGTGGACATGATGAGATTCAATATTAATGAC
TTTCTTCCCCCAGGAGGGGGCTCAAACCCCCGCTCTGTGCCCTTTGAATACTACAGAATAAGAAAGGTTAAGGTTGAA
TTCTGGCCCTGCTCCCCGATCACCCAGGGTGACAGGGGAGTGGGCTCCAGTGCTGTTATTCTAGATGATAACTTTGTA
ACAAAGGCCACAGCCCTCACCTACGACCCCTATGTAAACTACTCCTCCCGCCATACCATAACCCAGCCCTTCTCCTACCA
CTCCCGCTACTTTACCCCCAAACCTGTCCTAGATTCCACTATTGATTACTTCCAACCAAACAACAAAAGAAATCAGCTGT
GGCTGAGACTACAAACTGCTGGAAATGTAGACCACGTAGGCCTCGGCACTGCGTTCGAAAACAGTATATACGACCAG
GAATACAATATCCGTGTAACCATGTATGTACAATTCAGAGAATTTAATCTTAAAGACCCCCCACTTAACCCT--------

>JN989555_pcv2b

ATGACGTATCCAAGGAGGCGTTACCGGAGAAGAAGACACCGCCCCCGCAGCCATCTTGGCCAGATCCTCCGCCGCCG
CCCCTGGCTCGTCCACCCCCGCCACCGTTACCGCTGGAGAAGGAAAAATGGCATCTTCAACACCCGCCTCTCCCGCACC
TTCGGATATACTATCAAGCGAACCACAGTCAAAACGCCCTCCTGGGCGGTGGACATGATGAGGTTCAATATTAATGAC
TTTCTTCCCCCAGGAGGGGGCTCAAACCCCCGCTCTGTGCCCTTTGAATACTACAGAATAAGAAAGGTTAAGGTTGAA
TTCTGGCCCTGCTCCCCGATCACCCAGGGTGACAGGGGAGTGGGCTCCAGTGCTGTTATTCTAGATGATAACTTTGTA
ACAAAGGCCACAGCCCTCACCTATGACCCCTATGTAAACTACTCCTCCCGCCATACCATAACCCAGCCCTTCTCCTACCA
CTCCCGCTACTTTACCCCCAAACCTGTCCTAGATTCCACTATTGATTACTTCCAACCAAACAACAAAAGAAATCAGCTGT
GGCTGAGACTACAAACTGCTGGAAATGTAGACCACGTAGGCCTCGGCACTGCGTTCGAAAACAGTATATACGACCAG
GAATACAATATCCGTGTAACCATGTATGTACAATTCAGAGAATTTAATCTTAGAGACCCCCCACTTAACCCT--------

>AY484415_pcv2b

ATGACGTATCCAAGGAGGCGTTACCGGAGAAGAAGACACCGCCCCCGCAGCCATCTTGGCCAGATCCTCCGCCGCCG
CCCCTGGCTCGTCCACCCCCGCCACCGTTACCGCTGGAGAAGGAAAAATGGCATCTTCAACACCCGCCTCTCCCGCACC
TTCGGATATACTATCAAGCGAACCACAGTCAAAACGCCCTCCTGGGCGGTGGACATGATGAGATTCAATATTAATGAC
TTTCTTCCCCCAGGAGGGGGCTCAAACCCCCGCTCTGTGCCCTTTGAATACTACAGAATAAGAAAGGTTAAGGTTGAA
TTCTGGCCCTGCTCCCCGATCACCCAGGGTGACAGGGGAGTGGGCTCCAGTGCTGTTATTCTAGATGATAACTTTGTA
ACAAAGGCCACAGCCCTCACCTATGACCCCTATGTAAACTACTCCTCCCGCCATACCATAACCCAGCCCTTTTCCTACCA
CTCCCGCTACTTTACCCCCAAACCTGTCCTAGATTCCACTATTGATTACTTCCAACCAAACAACAAAAGAAATCAGCTGT
GGCTGAGACTACAAACTGCTGGAAATGTAGACCACGTAGGCCTCGGCACTGCGTTCGAAAACAGTATATACGACCAG
GAATACAATATCCGTGTAACCATGTATGTACAATTCAGAGAATTTAATCTTAAAGACCCCCCACTTAACCCT--------

>EF565344_pcv2b

ATGACGTATCCAAGGAGGCGTTACCGGAGAAGAAGACACCGCCCCCGCAGCCATCTTGGCCAGATCCTCCGCCGCCG
CCCCTGGCTCGTCCACCCCCGCCACCGTTACCGCTGGAGAAGGAAAAATGGCATCTTCAACACCCGCCTCTCCCGCACC
TTCGGATATACTATCAAGCGAACCACAGTCAAAACGCCCTCCTGGGCGGTGGACATGATGAGATTCAATATTAATGAC
TTTCTTCCCCCAGGAGGGGGCTCAAACCCCCGCTCTGTGCCCTTTGAATACTACAGAATAAGAAAGGTTAAGGTTGAA
TTCTGGCCCTGCTCCCCGATCACCCAGGGTGACAGGGGAGTGGGCTCCAGTGCTGTTATTCTAGATGATAACTTTGTA
ACAAAGGCCACAGCCCTCACCTATGACCCCTATGTAAACTACTCCTCCCGCCATACCATAACCCAGCCCTTCTCCTACCA
CTCCCGCTACTTTACCCCCAAACCTGTCCTAGATTCCACTATTGATTACTTCCAACCAAACAACAAAAGAAATCAGCTGT
GGCTGAGACTACAAACTGCTGGAAATGTAGACCACGTAGGCCTCGGCACTGCGTTCGAAAACAGTATATACGACCAG
GAATACAATATCCGTGTAACCATGTATGTACAATTCAGAGAATTTAATCTTAAAGACCCCCCACTTAACCCT--------

>GU938303_pcv2b

ATGACGTATCCAAGGAGGCGTTACCGGAGAAGAAGACACCGCCCCCGCAGCCATCTTGGCCAGATCCTCCGCCGCCG
CCCCTGGCTCGTCCACCCCCGCCACCGTTACCGCTGGAGAAGGAAAAATGGCATCTTCAACACCCGCCTCTCCCGCACC
TTCGGATATACTATCAAGCGAACCACAGTCAAAACGCCCTCCTGGGCGGTGGACATGATGAGATTCAATATTAATGAC
TTTCTTCCCCCAGGAGGGGGCTCAAACCCCCGCTCTGTGCCCTTTGAATACTACAGAATAAGAAAGGTTAAGGTTGAA
TTCTGGCCCTGCTCCCCGATCACCCAGGGTGACAGGGGAGTGGGCTCCAGTGCTGTTATTCTAGATGATAACTTTGTA
ACAAAGGCCACAGCCCTCACCTATGACCCCTATGTAAACTACTCCTCCCGCCATACCATAACCCAGCCCTTCTCCTACCA
CTCCCGCTACTTTACCCCCAAACCTGTCCTAGATTCCACTATTGATTACTTCCAACCAAACAACAAAAGAAATCAGCTGT
GGCTGAGACTACAAACTGCTGGAAATGTAGACCACGTAGGCCTCGGCACTGCGTTCGAAAACAGTATATACGACCAG
GAATACAATATCCGTGTAACCATGTATGTACAATTCAGAGAATTTAATCTTAAAGACCCCCCACTTAACCCT--------

>HM038021_pcv2b

ATGACGTATCCAAGGAGGCGTTACCGGAGAAGAAGACACCGCCCCCGCAGCCATCTTGGCCAGATCCTCCGCCGCCG
CCCCTGGCTCGTCCACCCCCGCCACCGTTACCGCTGGAGAAGGAAAAATGGCATCTTCAACACCCGCCTCTCCCGCACC
TTCGGATATACTATCAAGCGAACCACAGTCAAAACGCCCTCCTGGGCGGTGGACATGATGAGATTCAATATTAATGAC
TTTCTTCCCCCAGGAGGGGGCTCAAACCCCCGCTCTGTGCCCTTTGAATACTACAGAATAAGAAAGGTTAAGGTTGAA
TTCTGGCCCTGCTCCCCGATCACCCAGGGTGACAGGGGAGTGGGCTCCAGTGCTGTTATTCTAGATGATAACTTTGTA
ACAAAGGCCACAGCCCTCACCTATGACCCCTATGTAAACTACTCCTCCCGCCATACCATAACCCAGCCCTTCTCCTACCA
CTCCCGCTACTTTACCCCCAAACCTGTCCTAGATTCCACTATTGATTACTTCCAACCAAACAACAAAAGAAATCAGCTGT
GGCTGAGACTACAAACTGCTGGAAATGTAGACCACGTAGGCCTCGGCACTGCGTTCGAAAACAGTATATACGACCAG
GAATACAATATCCGTGTAACCATGTATGTACAATTCAGAGAATTTAATCTTAAAGACCCCCCACTTAACCCT--------

>EF565361_pcv2b

ATGACGTATCCAAGGAGGCGTTACCGGAGAAGAAGACACCGCCCCCGCAGCCATCTTGGCCAGATCCTCCGCCGCCG
CCCCTGGCTCGTCCACCCCCGCCACCGTTACCGCTGGAGAAGGAAAAATGGCATCTTCAACACCCGCCTCTCCCGCACC
TTCGGATATACTATCAAGCGAACCACAGTCAAAACGCCCTCCTGGGCGGTGGACATGATGAGATTCAATATTAATGAC
TTTCTTCCCCCAGGAGGGGGCTCAAACCCCCGCTCTGTGCCCTTTGAATACTACAGAATAAGAAAGGTTAAGGTTGAA
TTCTGGCCCTGCTCCCCGATCACCCAGGGTGACAGGGGAGTGGGCTCCAGTGCTGTTATTCTAGATGATAACTTTGTA
ACAAAGGCCACAGCCCTCACCTATGACCCCTATGTAAACTACTCCTCCCGCCATACCATAACCCAGCCCTTCTCCTACCA
CTCCCGCTACTTTACCCCCAAACCTGTCCTAGATTCCACTATTGATTACTTCCAACCAAACAACAAAAGAAATCAGCTGT
GGCTGAGACTACAAACTGCTGGAAATGTAGACCACGTAGGCCTCGGCACTGCGTTCGAAAACAGTATATACGACCAG
GAATACAATATCCGTGTAACCATGTATGTACAATTCAGAGAATTTAATCTTAAAGACCCCCCACTTAACCCT--------

>GU247987_pcv2b

ATGACGTATCCAAGGAGGCGTTACCGGAGAAGAAGACACCGCCCCCGCAGCCATCTTGGCCAGATCCTCCGCCGCCG
CCCCTGGCTCGTCCACCCCCGCCACCGTTACCGCTGGAGAAGGAAAAATGGCATCTTCAACACCCGCCTCTCCCGCACC
TTCGGATATACTATCAAGCGAACCACAGTCAAAACGCCCTCCTGGGCGGTGGACATGATGAGATTCAATATTAATGAC
TTTCTTCCCCCAGGAGGGGGCTCAAACCCCCGCTCTGTGCCCTTTGAATACTACAGAATAAGAAAGGTTAAGGTTGAA
TTCTGGCCCTGCTCCCCGATCACGCAGGGTGACAGGGGAGTGGGCTCCAGTGCTGTTATTCTAGATGATAACTTTGTA
ACAAAGGCCACAGCCCTCACCTATGACCCCTATGTAAACTACTCCTCCCGCCATACCATAACCCAGCCCTTCTCCTACCA
CTCCCGCTACTTTACCCCCAAACCTGTCCTAGATTCCACTATTGATTACTTCCAACCAAACAACAAAAGAAATCAGCTGT
GGCTGAGACTACAAACTGCTGGAAATGTAGACCACGTAGGCCTCGGCACTGCGTTCGAAAACAGTATATACGACCAG
GAATACAATATCCGTGTAACCATGTATGTACAATTCAGAGAATTTAATCTTAAAGACCCCCCACTTAACCCT--------

>HQ378162_pcv2b

ATGACGTATCCAAGGAGGCGTTACCGGAGAAGAAGACACCGCCCCCGCAGCCATCTTGGCCAGATCCTCCGCCGCCG
CCCCTGGCTCGTCCACCCCCGCCACCGTTACCGCTGGAGAAGGAAAAATGGCATCTTCAACACCCGCCTCTCCCGCACC
TTCGGATATACTATCAAGCGAACCACAGTCAAAACGCCCTCCTGGGCGGTGGACATGATGAGATTCAATATTAATGAC
TTTCTTCCCCCAGGAGGGGGCTCAAACCCCCGCTCTGTGCCCTTTGAATACTACAGAATAAGAAAGGTTAAGGTTGAA
TTCTGGCCCTGCTCCCCGATCACCCAGGGTGACAGGGGAGTGGGCTCCAGTGCTGTTATTCTAGATGATAACTTTGTA
ACAAAGGCCACAGCCCTCACCTATGACCCCTATGTAAACTACTCCTCCCGCCATACCATAACCCAGCCCTTCTCCTACCA
CTCCCGCTACTTTACCCCCAAACCTGTCCTAGATTCCACTATTGATTACTTCCAACCAAACAACAAAAGAAATCAGCTGT
GGCTGAGACTACAAACTGCTGGAAATGTAGACCACGTAGGCCTCGGCACTGCGTTCGAAAACAGTATATACGACCAG
GAATACAATATCCGTGTAACCATGTATGTACAATTCAGAGAATTTAATCTTAAAGACCCCCCACTTAACCCT--------

>HM776451_pcv2b

ATGACGTATCCAAGGAGGCGTTACCGGAGAAGAAGACACCGCCCCCGCAGCCATCTTGGCCAGATCCTCCGCCGCCG
CCCCTGGCTCGTCCACCCCCGCCACCGTTACCGCTGGAGAAGGAAAAATGGCATCTTCAACACCCGCCTCTCCCGCACC
TTCGGATATACTATCAAGCGAACCACAGTCAAAACGCCCTCCTGGGCGGTGGACATGATGAGATTCAATATTAATGAC
TTTCTTCCCCCAGGAGGGGGCTCAAACCCCCGCTCTGTGCCCTTTGAATACTACAGAATAAGAAAGGTTAAGGTTGAA
TTCTGGCCCTGCTCCCCGATCACCCAGGGTGACAGGGGAGTGGGCTCCAGTGCTGTTATTCTAGATGATAACTTTGTA
ACAAAGGCCACAGCCCTCACCTATGACCCCTATGTAAACTACTCCTCCCGCCATACCATAACCCAGCCCTTCTCCTACCA
CTCCCGCTACTTTACCCCCAAACCTGTCCTAGATTCCACTATTGATTACTTCCAACCAAACAACAAAAGAAATCAGCTGT
GGCTGAGACTACAAACTGCTGGAAATGTAGACCACGTAGGCCTCGGCACTGCGTTCGAAAACAGTATATACGACCAG
GAATACAATATCCGTGTAACCATGTATGTACAATTCAGAGAATTTAATCTTAAAGACCCCCCACTTAACCCT--------

>EU136718_pcv2b

ATGACGTATCCAAGGAGGCGTTACCGGAGAAGAAGACACCGCCCCCGCAGCCATCTTGGCCAGATCCTCCGCCGCCG
CCCCTGGCTCGTCCACCCCCGCCACCGTTACCGCTGGAGAAGGAAAAATGGCATCTTCAACACCCGCCTCTCCCGCACC
TTCGGATATACTATCAAGCGAACCACAGTCAAAACGCCCTCCTGGGCGGTGGACATGATGAGATTCAATATTAATGAC
TTTCTTCCCCCAGGAGGGGGCTCAAACCCCCGCTCTGTGCCCTTTGAATACTACAGAATAAGAAAGGTTAAGGTTGAA
TTCTGGCCCTGCTCCCCGATCACCCAGGGTGACAGGGGAGTGGGCTCCAGTGCTGTTATTCTAGATGATAACTTTGTA
ACAAAGGCCACAGCCCTCACCTATGACCCCTATGTAAACTACTCCTCCCGCCATACCATAACCCAGCCCTTCTCCTACCA
CTCCCGCTACTTTACCCCCAAACCTGTCCTAGATTCCACTATTGATTACTTCCAACCAAACAACAAAAGAAATCAGCTGT
GGCTGAGACTACAAACTGCTGGAAATGTAGACCACGTAGGCCTCGGCACTGCGTTCGAAAACAGTATATACGACCAG
GAATACAATATCCGTGTAACCATGTATGTACAATTCAGAGAATTTAATCTTAAAGACCCCCCACTTAACCCT--------

>HM038020_pcv2b

ATGACGTATCCAAGGAGGCGTTACCGGAGAAGAAGACACCGCCCCCGCAGCCATCTTGGCCAGATCCTCCGCCGCCG
CCCCTGGCTCGTCCACCCCCGCCACCGTTACCGCTGGAGAAGGAAAAATGGCATCTTCAACACCCGCCTCTCCCGCACC
TTCGGATATACTATCAAGCGAACCACAGTCAAAACGCCCTCCTGGGCGGTGGACATGATGAGATTCAATATTAATGAC
TTTCTTCCCCCAGGAGGGGGCTCAAACCCCCGCTCTGTGCCCTTTGAATACTACAGAATAAGAAAGGTTAAGGTTGAA
TTCTGGCCCTGCTCCCCGATCACCCAGGGTGACAGGGGAGTGGGCTCCAGTGCTGTTATTCTAGATGATAACTTTGTA
ACAAAGGCCACAGCCCTCACCTATGACCCCTATGTAAACTACTCCTCCCGCCATACCATAACCCAGCCCTTCTCCTACCA
CTCCCGCTACTTTACCCCCAAACCTGTCCTAGATTCCACTATTGATTACTTCCAACCAAACAACAAAAGGAATCAGCTGT
GGCTGAGACTACAAACTGCTGGAAATGTAGACCACGTAGGCCTCGGCACTGCGTTCGAAAACAGTATATACGACCAG
GAATACAATATCCGTGTAACCATGTATGTACAATTCAGAGAATTTAATCTTAAAGACCCCCCACTTAACCCT--------

>HM009336_pcv2b

ATGACGTATCCAAGGAGGCGTTACCGGAGAAGAAGACACCGCCCCCGCAGCCATCTTGGCCAGATCCTCCGCCGCCG
CCCCTGGCTCGTCCACCCCCGCCACCGTTACCGCTGGAGAAGGAAAAATGGCATCTTCAACACCCGCCTCTCCCGCACC
TTCGGATATACTATCAAGCGAACCACAGTCAAAACGCCCTCCTGGGCGGTGGACATGATGAGATTCAATATTAATGAC
TTTCTTCCCCCAGGAGGGGGCTCAAACCCCCGCTCTGTGCCCTTTGAATACTACAGAATAAGAAAGGTTAAGGTTGAA
TTCTGGCCCTGCTCCCCGATCACCCAGGGTGACAGGGGAGTGGGCTCCAGTGCTGTAATTCTAGATGATAACTTTGTA
ACAAAGGCCACAGCCCTCACCTATGACCCCTATGTAAACTACTCCTCCCGCCATACCATAACCCAGCCCTTCTCCTACCA
CTCCCGCTACTTTACCCCCAAACCTGTCCTAGATTCCACTATTGATTACTTCCAACCAAACAACAAAAGAAATCAGCTGT
GGCTGAGACTACAAACTGCTGGAAATGTAGACCACGTAGGCCTCGGCACTGCGTTCGAAAACAGTATATACGACCAG
GAATACAATATCCGTGTAACCATGTATGTACAATTCAGAGAATTTAATCTTAAAGACCCCCCACTTAACCCT--------

>EU136716_pcv2b

ATGACGTATCCAAGGAGGCGTTACCGGAGAAGAAGACACCGCCCCCGCAGCCATCTTGGCCAGATCCTCCGCCGCCG
CCCCTGGCTCGTCCACCCCCGCCACCGTTACCGCTGGAGAAGGAAAAATGGCATCTTCAACACCCGCCTCTCCCGCACC
TTCGGATATACTATCAAGCGAACCACAGTCAAAACGCCCTCCTGGGCGGTGGACATGATGAGATTCAATATTAATGAC
TTTCTTCCCCCAGGAGGGGGCTCAAACCCCCGCTCTGTGCCCTTTGAATACTACAGAATAAGAAAGGTTAAGGTTGAA
TTCTGGCCCTGCTCCCCGATCACCCAGGGTGACAGGGGAGTGGGCTCCAGTGCTGTGATTCTAGATGATAACTTTGTA
ACAAAGGCCACAGCCCTCACCTATGACCCCTATGTAAACTACTCCTCCCGCCATACCATAACCCAGCCCTTCTCCTACCA
CTCCCGCTACTTTACCCCCAAACCTGTCCTAGATTCCACTATTGATTACTTCCAACCAAACAACAAAAGAAATCAGCTGT
GGCTGAGACTACAAACTGCTGGAAATGTAGACCACGTAGGCCTCGGCACTGCGTTCGAAAACAGTATATACGACCAG
GAATACAATATCCGTGTAACCATGTATGTACAATTCAGAGAATTTAATCTTAAAGACCCCCCACTTAACCCT--------

>HQ591376_pcv2b

ATGACGTATCCAAGGAGGCGTTACCGGAGAAGAAGACACCGCCCCCGCAGCCATCTTGGCCAGATCCTCCGCCGCCG
CCCCTGGCTCGTCCACCCCCGCCACCGTTACCGCTGGAGAAGGAAAAATGGCATCTTCAACACCCGCCTCTCCCGCACC
TTCGGATATACTATCAAGCGAACCACAGTCAAAACGCCCTCCTGGGCGGTGGACATGATGAGATTCAATATTAATGAC
TTTCTTCCCCCAGGAGGGGGCTCAAACCCCCGCTCTGTGCCCTTTGAATACTACAGAATAAGAAAGGTTAAGGTTGAA
TTCTGGCCCTGCTCCCCGATCACCCAGGGTGACAGGGGAGTGGGCTCCAGTGCTGTTATTCTAGATGATAACTTTGTA
ACAAAGGCCACAGCCCTCACTTATGACCCCTATGTAAACTACTCCTCCCGCCATACCATAACCCAGCCCTTCTCCTACCA
CTCCCGCTACTTTACCCCCAAACCTGTCCTAGATTCCACTATTGATTATTTCCAACCAAACAACAAAAGAAATCAGCTGT
GGATGAGACTACAAACTGCTGGAAATGTAGACCACGTAGGCCTCGGCACTGCGTTCGAAAACAGTATATACGACCAG
GAATACAATATCCGTGTAACCATGTATGTACAATTCAGAGAATTTAATCTTAAAGACCCCCCACTTAACCCT--------

>HQ591377_pcv2b

ATGACGTATCCAAGGAGGCGTTACCGGAGAAGAAGACACCGCCCCCGCAGCCATCTTGGCCAGATCCTCCGCCGCCG
CCCCTGGCTCGTCCACCCCCGCCACCGTTACCGCTGGAGAAGGAAAAATGGCATCTTCAACACCCGCCTCTCCCGCACC
TTCGGATATACTATCAAGCGAACCACAGTCAAAACGCCCTCATGGGCGGTGGACATGATGAGATTCAATATTAATGAC
TTTCTTCCCCCAGGAGGGGGCTCAAACCCCCGCTCTGTGCCCTTTGAATACTACAGAATAAGAAAGGTTAAGGTTGAA
TTCTGGCCCTGCTCCCCGATCACCCAGGGTGACAGGGGAGTGGGCTCCAGTGCTGTTATTCTAGATGATAACTTTGTA
ACAAAGGCCACAGCCCTCACTTATGACCCCTATGTAAACTACTCCTCCCGCCATACCATAACCCAGCCCTTCTCCTACCA
CTCCCGCTACTTTACCCCCAAACCTGTCCTAGATTCCACTATTGATTATTTCCAACCAAACAACAAAAGAAATCAGCTGT
GGCTGAGACTACAAACTGCTGGAAATGTAGACCACGTAGGCCTCGGCACTGCGTTCGAAAACAGTATATACGACCAG
GAATACAATATCCGTGTAACCATGTATGTACAATTCAGAGAATTTAATCTTAAAGACCCCCCACTTAACCCT--------

>EF565366_pcv2b

ATGACGTATCCAAGGAGGCGTTACCGGAGAAGAAGACACCGCCCCCGCAGCCATCTTGGCCAGATCCTCCGCCGCCG
CCCCTGGCTCGTCCACCCCCGCCACCGTTACCGCTGGAGAAGGAAAAATGGCATCTTCAACACCCGCCTCTCCCGCACC
TTCGGATATACTATCAAGCGAACCACAGTCAAAACGCCCTCCTGGGCGGTGGACATGATGAGATTCAATATTAATGAC
TTTCTTCCCCCAGGAGGGGGCTCAAACCCCCGCTCTGTGCCCTTTGAATACTACAGAATAAGAAAGGTTAAGGTTGAA
TTCTGGCCCTGCTCCCCGATCACCCAGGGTGACAGGGGAGTGGGCTCCAGTGCTGTTATTCTAGATGATAACTTTGTA
ACAAAGGCCACAGCCCTCACCTATGACCCCTATGTAAACTACTCCTCCCGCCATACCATAACCCAGCCCTTCTCCTACCA
CTCCCGCTACTTTACCCCCAAACCTGTCCTAGATTCCACTATTGATTATTTCCAACCAAACAACAAAAGAAATCAGCTGT
GGCTGAGACTACAAACTGCTGGAAATGTAGACCACGTAGGCCTCGGCACTGCGTTCGAAAACAGTATATACGACCAG
GAATACAATATCCGTGTAACCATGTATGTACAATTCAGAGAATTTAATCTTAAAGACCCCCCACTTAACCCT--------

>EF565350_pcv2b

ATGACGTATCCAAGGAGGCGTTACCGGAGAAGAAGACACCGCCCCCGCAGCCATCTTGGCCAGATCCTCCGCCGCCG
CCCCTGGCTCGTCCACCCCCGCCACCGTTACCGCTGGAGAAGGAAAAATGGCATCTTCAACACCCGCCTCTCCCGCACC
TTCGGATATACTATCAAGCGAACCACAGTCAAAACGCCCTCCTGGGCGGTGGACATGATGAGATTCAATATTAATGAC
TTTCTTCCCCCAGGAGGGGGCTCAAACCCCCGCTCTGTGCCCTTTGAATACTACAGAATAAGAAAGGTTAAGGTTGAA
TTCTGGCCCTGCTCCCCGATCACCCAGGGTGACAGGGGAGTGGGCTCCAGTGCTGTTATTCTAGATGATAACTTTGTA
ACAAAGGCCACAGCCCTCACCTATGACCCCTATGTAAACTACTCCTCCCGCCATACCATAACCCAGCCCTTCTCCTACCA
CTCCCGCTACTTTACCCCCAAACCTGTCCTAGATTCCACTATTGATTATTTCCAACCAAACAACAAAAGAAATCAGCTGT
GGCTGAGACTACAAACTGCTGGAAATGTAGACCACGTAGGCCTCGGCACTGCGTTCGAAAACAGTATATACGACCAG
GAATACAATATCCGTGTAACCATGTATGTACAATTCAGAGAATTTAATCTTAAAGACCCCCCACTTAACCCT--------

>EF565364_pcv2b

ATGACGTATCCAAGGAGGCGTTACCGGAGAAGAAGACACCGCCCCCGCAGCCATCTTGGCCAGATCCTCCGCCGCCG
CCCCTGGCTCGTCCACCCCCGCCACCGTTACCGCTGGAGAAGGAAAAATGGCATCTTCAACACCCGCCTCTCCCGCACC
TTCGGATATACTATCAAGCGAACCACAGTCAAAACGCCCTCCTGGGCGGTGGACATGATGAGATTCAATATTAATGAC
TTTCTTCCCCCAGGAGGGGGCTCAAACCCCCGCTCTGTGCCCTTTGAATACTACAGAATAAGAAAGGTTAAGGTTGAA
TTCTGGCCCTGCTCCCCGATCACCCAGGGTGACAGGGGAGTGGGCTCCAGTGCTGTTATTCTAGATGATAACTTTGTA
ACAAAGGCCACAGCCCTCACCTATGACCCCTATGTAAACTACTCCTCCCGCCATACCATAACCCAGCCCTTCTCCTACCA
CTCCCGCTACTTTACCCCCAAACCTGTCCTAGATTCCACTATTGATTATTTCCAACCAAACAACAAAAGAAATCAGCTGT
GGCTGAGACTACAAACTGCTGGAAATGTAGACCACGTAGGCCTCGGCACTGCGTTCGAAAACAGTATATACGACCAG
GAATACAATATCCGTGTAACCATGTATGTACAATTCAGAGAATTTAATCTTAAAGACCCCCCACTTAACCCT--------

>EF565355_pcv2b

ATGACGTATCCAAGGAGGCGTTACCGGAGAAGAAGACACCGCCCCCGCAGCCATCTTGGCCAGATCCTCCGCCGCCG
CCCCTGGCTCGTCCACCCCCGCCACCGTTACCGCTGGAGAAGGAAAAATGGCATCTTCAACACCCGCCTCTCCCGCACC
TTCGGATATACTATCAAGCGAACCACAGTCAAAACGCCCTCCTGGGCGGTGGACATGATGAGATTCAATATTAATGAC
TTTCTTCCCCCAGGAGGGGGCTCAAACCCCCGCTCTGTGCCCTTTGAATACTACAGAATAAGAAAGGTTAAGGTTGAA
TTCTGGCCCTGCTCCCCGATCACCCAGGGTGACAGGGGAGTGGGCTCCAGTGCTGTAATTCTAGATGATAACTTTGTA
ACAAAGGCCACAGCCCTCACCTATGACCCCTATGTAAACTACTCCTCCCGCCATACCATAACCCAGCCCTTCTCCTACCA
CTCCCGCTACTTTACCCCCAAACCTGTCCTAGATTCCACTATTGATTATTTCCAACCAAACAACAAAAGAAATCAGCTGT
GGCTGAGACTACAAACTGCTGGAAATGTAGACCACGTAGGCCTCGGCACTGCGTTCGAAAACAGTATATACGACCAG
GAATACAATATCCGTGTAACCATGTATGTACAATTCAGAGAATTTAATCTTAAAGACCCCCCACTTAACCCT--------

>KC835192_pcv2b

ATGACGTATCCAAGGAGGCGTTACCGGAGAAGAAGACACCGCCCCCGCAGCCATCTTGGCCAGATCCTCCGCCGCCG
CCCCTGGCTCGTCCACCCCCGCCACCGTTACCGCTGGAGAAGGAAAAATGGCATCTTCAACACCCGCCTCTCCCGCACC
TTCGGATATACTATCAAACGAACCACAGTCAAAACGCCCTCCTGGGCGGTGGACATGATGAGATTCAATATTAATGAC
TTTCTTCCCCCAGGAGGGGGCTCAAACCCCCGCTCTGTGCCCTTTGAATACTACAGAATAAGAAAGGTTAAGGTTGAA
TTCTGGCCCTGCTCCCCGATCACCCAGGGTGACAGGGGAGTGGGCTCCAGTGCTGTTATTCTAGATGATAACTTTGTA
ACAAAGGCCACAGCCCTCACCTATGACCCCTATGTAAACTACTCCTCCCGCCATACCATAACCCAGCCCTTCTCCTACCA
CTCCCGCTACTTTACCCCCAAACCTGTCCTAGATTCCACTATTGATTATTTCCAACCAAACAACAAAAGAAATCAGCTGT
GGCTGAGACTACAAACTGCTGGAAATGTAGACCACGTAGGCCTCGGCACTGCGTTCGAAAACAGTATATACGACCAG
GAATACAATATCCGTGTAACCATGTATGTACAATTCAGAGAATTTAATCTTAAAGACCCCCCACTTAACCCT--------

>EU755374_pcv2b

ATGACGTATCCAAGGAGGCGTTACCGGAGAAGAAGACACCGCCCACGCAGCCATCTTGGCCAGATCCTCCGCCGCCG
CCCCTGGCTCGTCCACCCCCGCCACCGTTACCGCTGGAGAAGGAAAAATGGCATCTTCAACACCCGCCTCTCCCGCACC
TTCGGATATACTATCAAACGAACCACAGTCAAAACGCCCTCCTGGGCGGTGGACATGATGAGATTCAATATTAATGAC
TTTCTTCCCCCAGGAGGGGGCTCAAACCCCCGCTCTGTGCCCTTTGAATACTACAGAATAAGAAAGGTTAAGGTTGAA
TTCTGGCCCTGCTCCCCGATCACCCAGGGTGACAGGGGAGTGGGCTCCAGTGCTGTTATTCTAGATGATAACTTTGTA
ACAAAGGCCACAGCCCTCACCTATGACCCCTATGTAAACTACTCCTCCCGCCATACCATAACCCAGCCCTTCTCCTACCA
CTCCCGCTACTTTACCCCCAAACCTGTCCTAGATTCCACTATTGATTACTTCCAACCAAACAACAAAAGAAATCAGCTGT
GGCTGAGACTACAAACTGCTGGAAATGTAGACCACGTAGGCCTCGGCACTGCGTTCGAAAACAGTATATACGACCAG
GAATACAATATCCGTGTAACCATGTATGTACAATTCAGAGAATTTAATCTTAAAGACCCCCCATTAAACCCT--------

>EU755371_pcv2b

ATGACGTATCCAAGGAGGCGTTACCGGAGAAGAAGACACCGCCCACGCAGCCATCTTGGCCAGATCCTCCGCCGCCG

CCCCTGGCTCGTCCACCCCCGCCACCGTTACCGCTGGAGAAGGAAAAGTGGCATCTTCAACACCCGCCTCTCCCGCAC

CTTCGGATATACTATCAAACGAACCACAGTCAAAACGCCCTCCTGGGCGGTGGACATGATGAGATTCAATATTAATGA

CTTTCTTCCCCCAGGAGGGGGCTCAAACCCCCGCTCTGTGCCCTTTGAATACTACAGAATAAGAAAGGTTAAGGTTGA

ATTCTGGCCCTGCTCCCCGATCACCCAGGGTGACAGGGGAGTGGGCTCCAGTGCTGTTATTCTAGATGATAACTTTGT

AACAAAGGCCACAGCCCTCACCTATGACCCCTATGTAAACTACTCCTCCCGCCATACCATAACCCAGCCCTTCTCCTACC

ACTCCCGCTACTTTACCCCCAAACCTGTCCTAGATTCCACTATTGATTACTTCCAACCAAACAACAAAAAGAATCAGCTG

TGGCTGAGACTACAAACTGCTGGAAATGTAGACCACGTAGGCCTCGGCACTGCCGTCGAAAACAGTATATACGACCA

GGAATACAATATCCGTGTAACCATGTATGTACAATTCAGAGAATTTAATCTTACAGACCCCCCATTTAACCGT--------

>EU755372_pcv2b

ATGACGTATCCAAGGAGGCGTTACCGGAGAAGAAGACACCGCCCACGCAGCCATCTTGGCCAGATCCTCCGCCGCCG

CCCCTGGCTCGTCCACCCCCGCCACCGTTACCGCTGGAGAAGGAAAAATGGCATCTTCAACACCCGCCTCTCCCGCACC

TTCGGATATACTATCAAACGAACCACAGTCAAAACGCCCTCCTGGGCGGTGGACATGATGAGATTCAATATTAATGAC

TTTCTTCCCCCAGGAGGGGGCTCAAACCCCCGCTCTGTGCCCTTTGAATACTACAGAATAAGAAAGGTTAAGGTTGAA

TTCTGGCCCTGCTCCCCGATCACCCAGGGTGACAGGGGAGTGGGCTCCAGTGCTGTTATTCTAGATGATAACTTTGTA

ACAAAGGCCACAGCCCTCACCTATGACCCCTATGTAAACTACTCCTCCCGCCATACCATAACCCAGCCCTTCTCCTACCA

CTCCCGCTACTTTACCCCCAAACCTGTCCTAGATTCCACTATTGATTACTTCCAACCAAACAAAAAAAGAAATCAGCTGT

GGCTGAGACTACAAACTGCTGGAAATGTAGACCACGTAGGCCTCGGCACTGCGTTCGAAAACAGTATATACGAACAG

GAATACAATATCCGTGTAACCATGTATGTACAATTCAGAGAATTTAATCTTAAAGAACCCCCATTTAACCCT--------

>DQ364650_pcv2b

ATGACGTATCCAAGGAGGCGTTACCGGAGAAGAAGACACCGCCCACGCAGCCATCTTGGCCAGATCCTCCGCCGCCG

CCCCTGGCTCATCCACCCCCGCCACCGTTACCGCTGGAGAAGGAAAAATGGCATCTTCAACACCCGCCTCTCCCGCACC

TTCGGATATACTATCAAACGAACCACAGTCAAAACGCCCTCCTGGGCGGTGGACATGATGAGATTCAATATTAATGAC

TTTCTTCCCCCAGGAGGGGGCTCAAACCCCCGCTCTGTGCCCTTTGAATACTACAGAATAAGAAAGGTTAAGGTTGAA

TTCTGGCCCTGCTCCCCGATCACCCAGGGTGACAGGGGAGTGGGCTCCAGTGCTGTTATTCTAGATGATAACTTTGTA

ACAAAGGCCACAGCCCTCACCTATGACCCCTATGTAAACTACTCCTCCCGCCATACCATAACCCAGCCCTTCTCCTACCA

CTCCCGCTACTTTACCCCCAAACCTGTCCTAGATTCCACTATTGATTACTTCCAACCAAACAACAAAAGAAATCAGCTGT

GGCTGAGACTACAAACTGCTGGAAATGTAGACCACGTAGGCCTCGGCACTGCGTTCGAAAACAGTATATACGACCAG

GAATACAATATCCGTGTAACCATGTATGTACAATTCAGAGAATTTAATCTTAAAGACCCCCCACTTAACACT--------

>JQ387582_pcv2b

ATGACGTATCCAAGGAGGCGTTACCGGAGAAGAAGACACCGCCCCCGCAGCCATCTTGGCCAGATCCTCCGCCGCCG

CCCCTGGCTCGTCCACCCCCGCCACCGTTACCGCTGGAGAAGGAAAAATGGCATCTTCAACACCCGCCTCTCCCGCACC

TTCGGATATACTATCAAACGAACCACAGTCAAAACGCCCTCCTGGGCGGTGGACATGATGAGATTCAATATTAATGAC

TTTCTTCCCCCAGGAGGGGGCTCAAACCCCCGCTCTGTGCCCTTTGAATACTACAGAATAAGAAAGGTTAAGGTTGAA

TTCTGGCCCTGCTCCCCGATCACCCAGGGTGACAGGGGAGTGGGCTCCAGTGCTGTTATTCTAGATGATAACTTTGTA

ACAAAGGCCACAGCCCTCACCTATGACCCCTATGTAAACTACTCCTCCCGCCATACCATAACCCAGCCCTTCTCCTACCA

CTCCCGCTACTTTACCCCCAAACCTGTCCTAGATTCCACTATTGATTACTTCCAACCAAACAACAAAAGAAATCAGCTGT

GGCTGAGACTACAAACTGCTGGAAATGTAGACCACGTAGGCCTCGGCACTGCGTTCGAAAACAGTATATACGACCAG

GAATACAATATCCGTGTAACCATGTATGTACAATTCAGAGAATTTAATCTTAAAGACCCCCCACTTAACCCT--------

>DQ861900_pcv2b

ATGACGTATCCAAGGAGGCGTTACCGGAGAAGAAGACACCGCCCCCGCAGCCATCTTGGCCAGATCCTCCGCCGCCG
CCCCTGGCTCGTCCACCCCCGCCACCGTTACCGCTGGAGAAGGAAAAATGGCATCTTCAACACCCGCCTCTCCCGCACC
TTCGGATATACTATCAAACGAACCACAGTCAAAACGCCCTCCTGGGCGGTGGACATGATGAGATTCAATATTAATGAC
TTTCTTCCCCCAGGAGGGGGCTCAAACCCCCGCTCTGTGCCCTTTGAATACTACAGAATAAGAAAGGTTAAGGTTGAA
TTCTGGCCCTGCTCCCCGATCACCCAGGGTGACAGGGGAGTGGGCTCCAGTGCTGTTATTCTAGATGATAACTTTGTA
ACAAAGGCCACAGCCCTCACCTATGACCCCTATGTAAACTACTCCTCCCGCCATACCATAACCCAGCCCTTCTCCTACCA
CTCCCGCTACTTTACCCCCAAACCTGTCCTAGATTCCACTATTGATTACTTCCAACCAAACAACAAAAGAAATCAGCTGT
GGCTGAGACTACAAACTGCTGGAAATGTAGACCACGTAGGCCTCGGCACTGCGTTCGAAAACAGTATATACGACCAG
GAATACAATATCCGTGTAACCATGTATGTACAATTCAGAGAATTTAATCTTAAAGACCCCCCACTTAACCCT--------

>DQ861901_pcv2b

ATGACGTATCCAAGGAGGCGTTACCGGAGAAGAAGACACCGCCCCCGCAGCCATCTTGGCCAGATCCTCCGCCGCCG
CCCCTGGCTCGTCCACCCCCGCCACCGTTACCGCTGGAGAAGGAAAAATGGCATCTTCAACACCCGCCTCTCCCGCACC
TTCGGATATACTATCAAACGAACCACAGTCAAAACGCCCTCCTGGGCGGTGGACATGATGAGATTCAATATTAATGAC
TTTCTTCCCCCAGGAGGGGGCTCAAACCCCCGCTCTGTGCCCTTTGAATACTACAGAATAAGAAAGGTTAAGGTTGAA
TTCTGGCCCTGCTCCCCGATCACCCAGGGTGACAGGGGAGTGGGCTCCAGTGCTGTTATTCTAGATGATAACTTTGTA
ACAAAGGCCACAGCCCTCACCTATGACCCCTATGTAAACTACTCCTCCCGCCATACCATAACCCAGCCCTTCTCCTACCA
CTCCCGCTACTTTACCCCCAAACCTGTCCTAGATTCCACTATTGATTACTTCCAACCAAACAACAAAAGAAATCAGCTGT
GGCTGAGACTACAAACTGCTGGAAATGTAGACCACGTAGGCCTCGGCACTGCGTTCGAAAACAGTATATACGACCAG
GAATACAATATCCGTGTAACCATGTATGTACAATTCAGAGAATTTAATCTTAAAGACCCCCCACTTAACCCT--------

>DQ861898_pcv2b

ATGACGTATCCAAGGAGGCGTTACCGGAGAAGAAGACACCGCCCCCGCAGCCATCTTGGCCAGATCCTCCGCCGCCG
CCCCTGGCTCGTCCACCCCCGCCACCGTTACCGCTGGAGAAGGAAAAATGGCATCTTCAACACCCGCCTCTCCCGCACC
TTCGGATATACTATCAAACGAACCACAGTCAAAACGCCCTCCTGGGCGGTGGACATGATGAGATTCAATATTAATGAC
TTTCTTCCCCCAGGAGGGGGCTCAAACCCCCGCTCTGTGCCCTTTGAATACTACAGAATAAGAAAGGTTAAGGTTGAA
TTCTGGCCCTGCTCCCCGATCACCCAGGGTGACAGGGGAGTGGGCTCCAGTGCTGTTATTCTAGATGATAACTTTGTA
ACAAAGGCCACAGCCCTCACCTATGACCCCTATGTAAACTACTCCTCCCGCCATACCATAACCCAGCCCTTCTCCTACCA
CTCCCGCTACTTTACCCCCAAACCTGTCCTAGATTCCACTATTGATTACTTCCAACCAAACAACAAAAGAAATCAGCTGT
GGCTGAGACTACAAACTGCTGGAAATGTAGACCACGTAGGCCTCGGCACTGCGTTCGAAAACAGTATATACGACCAG
GAATACAATATCCGTGTAACCATGTATGTACAATTCAGAGAATTTAATCTTAAAGACCCCCCACTTAACCCT--------

>HM641752_pcv2b

ATGACGTATCCAAGGAGGCGTTACCGGAGAAGAAGACACCGCCCCCGCAGCCATCTTGGCCAGATCCTCCGCCGCCG
CCCCTGGCTCGTCCACCCCCGCCACCGTTACCGCTGGAGAAGGAAAAATGGCATCTTCAACACCCGCCTCTCCCGCACC
TTCGGATATACTATCAAGCGAACCACAGTCAAAACGCCCTCCTGGGCGGTGGACATGATGAGATTCAATATTAATGAC
TTTCTTCCCCCAGGAGGGGGCTCAAACCCCCGCTCTGTGCCCTTTGAATACTACAGAATAAGAAAGGTTAAGGTTGAA
TTCTGGCCCTGCTCCCCGATCACCCAGGGTGACAGGGGAGTGGGCTCCAGTGCTGTTATTCTAGATGATAACTTTGTA
ACAAAGGCCACAGCCCTCACCTATGACCCCTATGTAAACTACTCCTCCCGCCATACCATAACCCAGCCCTTCTCCTACCA
CTCCCGCTACTTTACCCCCAAACCTGTCCTAGATTCCACTATTGATTACTTCCAACCAAACAACAAAAGAAATCAGCTGT
GGCTGAGACTACAAACTGCTGGAAATGTAGACCACGTAGGCCTCGGCACTGCGTTCGAAAACAGTATATACGACCAG
GAATACAATATCCGTGTAACCATGTATGTACAATTCAGAGAATTTAATCTTAAAGACCCCCCACTTAACCCT--------

>HQ591365_pcv2b

ATGACGTATCCAAGGAGGCGTTACCGGAGAAGAAGACACCGCCCCCGCAGCCATCTTGGCCAGATCCTCCGCCGCCG
CCCCTGGCTCGTCCACCCCCGCCACCGTTACCGCTGGAGAAGGAAAAATGGCATCTTCAACACCCGCCTCTCCCGCACC
TTCGGATATACTATCAAGCGAACCACAGTCAAAACGCCCTCCTGGGCGGTGGACATGATGAGATTCAATATTAATGAC
TTTCTTCCCCCAGGAGGGGGCTCAAACCCCCGCTCTGTGCCCTTTGAATACTACAGAATAAGAAAGGTTAAGGTTGAA
TTCTGGCCCTGCTCCCCGATCACCCAGGGTGACAGGGGAGTGGGCTCCAGTGCTGTTATTCTAGATGATAACTTTGTA
ACAAAGGCCACAGCCCTCACCTATGACCCCTATGTAAACTACTCCTCCCGCCATACCATAACCCAGCCCTTCTCCTACCA
CTCCCGCTACTTTACCCCAAAACCTGTCCTAGATTCCACTATTGATTACTTCCAACCAAACAACAAAAGAAATCAGCTGT
GGCTGAGACTACAAACTGCTGGAAATGTAGACCACGTAGGCCTCGGCACTGCGTTCGAAAACAGTATATACGACCAG
GAATACAATATCCGTGTAACCATGTATGTACAATTCAGAGAATTTAATCTTAAAGACCCCCCACTTAACCCT--------

>HM009335_pcv2b

ATGACGTATCCAAGGAGGCGTTACCGGAGAAGAAGACACCGCCCCCGCAGCCATCTTGGCCAGATCCTCCGCCGCCG
CCCCTGGCTCGTCCACCCCCGCCACCGTTACCGCTGGAGAAGGAAAAATGGCATCTTCAACACCCGCCTCTCCCGCACC
TTCGGATATACTATCAAGCGAACCACAGTCAAAACGCCCTCCTGGGCGGTGGACATGATGAGATTCAATATTAATGAC
TTTCTTCCCCCAGGAGGGGGCTCAAACCCCCGCTCTGTGCCCTTTGAATACTACAGAATAAGAAAGGTTAAGGTTGAA
TTCTGGCCCTGCTCCCCGATCACCCAGGGTGACAGGGGAGTGGGCTCCAGTGCTGTTATTCTAGATGATAACTTTGTA
ACAAAGGCCACAGCCCTCACCTATGACCCCTATGTAAACTACTCCTCCCGCCATACCATAACCCAGCCCTTCTCCTACCA
CTCCCGCTACTTTACCCCCAAACCTGTCCTAGATTCCACTATTGATTACTTCCAACCAAACAACAAAAGAAATCAGCTGT
GGCTGAGACTACAAACTGCTGGAAATGTAGACCACGTAGGCCTCGGCACTGCGTTCGAAAACAGTATATACGACCAG
GAATACAATATCCGTGTAACCATGTATGTACAATTCAGAGAATTTAATCTTAAAGACCCCCCACTTAACCCT--------

>JN382174_pcv2b

ATGACGTATCCAAGGAGGCGTTACCGGAGAAGAAGACACCGCCCCCGCAGCCATCTTGGCCAGATCCTCCGCCGCCG
CCCCTGGCTCGTCCACCCCCGCCACCGTTACCGCTGGAGAAGGAAAAATGGCATCTTCAACACCCGCCTCTCCCGCACC
TTCGGATATACTATCAAGCGAACCACAGTCAAAACGCCCTCCTGGGCGGTGGACATGATGAGATTCAATATTAATGAC
TTTCTTCCCCCAGGAGGGGGCTCAAACCCCCGCTCTGTGCCCTTTGAATACTACAGAATAAGAAAGGTTAAGGTTGAA
TTCTGGCCCTGCTCCCCGATCACCCAGGGTGACAGGGGAGTGGGCTCCAGTGCTGTTATTCTAGATGATAACTTTGTA
ACAAAGGCCACAGCCCTCACCTATGACCCCTATGTAAACTACTCCTCCCGCCATACCATAACCCAGCCCTTCTCCTACCA
CTCCCGCTACTTTACCCCCAAACCTGTCCTAGATTCCACTATTGATTACTTCCAACCAAACAACAAAAGAAATCAGCTGT
GGCTGAGACTACAAACTGCTGGAAATGTAGACCACGTAGGCCTCGGCACTGCGTTCGAAAACAGTATATACGACCAG
GAATACAATATCCGTGTAACCATGTATGTACAATTCAGAGAATTTAATCTTAAAGACCCCCCACTTAACCCT--------

>AY484407_pcv2b

ATGACGTATCCAAGGAGGCGTTACCGGAGAAGAAGACACCGCCCCCGCAGCCATCTTGGCCAGATCCTCCGCCGCCG
CCCCTGGCTCGTCCACCCCCGCCACCGTTACCGCTGGAGAAGGAAAAATGGCATCTTCAACACCCGCCTCTCCCGCACC
TTCGGATATACTATCAAGCGAACCACAGTCAAAACGCCCTCCTGGGCGGTGGACATGATGAGATTCAATATTAATGAC
TTTCTTCCCCCAGGAGGGGGCTCAAACCCCCGCTCTGTGCCCTTTGAATACTACAGAATAAGAAAGGTTAAGGTTGAA
TTCTGGCCCTGCTCCCCGATCACCCAGGGTGACAGGGGAGTGGGCTCCAGTGCTGTTATTCTAGATGATAACTTTGTA
ACAAAGGCCACAGCCCTCACCTATGACCCCTATGTAAACTACTCCTCCCGCCATACCATAACCCAGCCCTTCTCCTACCA
CTCCCGCTACTTTACCCCCAAACCTGTCCTAGATTCCACTATTGATTACTTCCAACCAAACAACAAAAGAAATCAGCTGT
GGCTGAGACTACAAACTGCTGGAAATGTAGACCACGTAGGCCTCGGCACTGCGTTCGAAAACAGTATATACGACCAG
GAATACAATATCCGTGTAACCATGTATGTACAATTCAGAGAATTTAATCTTAAAGACCCCCCACTTAACCCT--------

>AY321990_pcv2b

ATGACGTATCCAAGGAGGCGTTACCGGAGAAGAAGACACCGCCCCCGCAGCCATCTTGGCCAGATCCTCCGCCGCCG
CCCCTGGCTCGTCCACCCCCGCCACCGTTACCGCTGGAGAAGGAAAAATGGCATCTTCAACACCCGCCTCTCCCGCACC
TTCGGATATACTATCAAGCGAACCACAGTCAAAACGCCCTCCTGGGCGGTGGACATGATGAGATTCAATATTAATGAC
TTTCTTCCCCCAGGAGGGGGCTCAAACCCCCGCTCTGTGCCCTTTGAATACTACAGAATAAGAAAGGTTAAGGTTGAA
TTCTGGCCCTGCTCCCCGATCACCCAGGGTGACAGGGGAGTGGGCTCCAGTGCTGTTATTCTAGATGATAACTTTGTA
ACAAAGGCCACAGCCCTCACCTATGACCCCTATGTAAACTACTCCTCCCGCCATACCATAACCCAGCCCTTCTCCTACCA
CTCCCGCTACTTTACCCCCAAACCTGTCCTAGATTCCACTATTGATTACTTCCAACCAAACAACAAAAGAAATCAGCTGT
GGCTGAGACTACAAACTGCTGGAAATGTAGACCACGTAGGCCTCGGCACTGCGTTCGAAAACAGTATATACGACCAG
GAATACAATATCCGTGTAACCATGTATGTACAATTCAGAGAATTTAATCTTAAAGACCCCCCACTTAACCCT--------

>AY321999_pcv2b

ATGACGTATCCAAGGAGGCGTTACCGGAGAAGAAGACACCGCCCCCGCAGCCATCTTGGCCAGATCCTCCGCCGCCG
CCCCTGGCTCGTCCACCCCCGCCACCATTACCGCTGGAGAAGGAAAAATGGCATCTTCAACACCCGCCTCTCCCGCACC
TTCGGATATACTATCAAGCGAACCACAGTCAAAACGCCCTCCTGGGCGGTGGACATGATGAGATTCAATATTAATGAC
TTTCTTCCCCCAGGAGGGGGCTCAAACCCCCGCTCTGTGCCCTTTGAATACTACAGAATAAGAAAGGTTAAGGTTGAA
TTCTGGCCCTGCTCCCCGATCACCCAGGGTGACAGGGGAGTGGGCTCCAGTGCTGTTATTCTAGATGATAACTTTGTA
ACAAAGGCCACAGCCCTCACCTATGACCCCTATGTAAACTACTCCTCCCGCCATACCATAACCCAGCCCTTCTCCTACCA
CTCCCGCTACTTTACCCCCAAACCTGTCCTAGATTCCACTATTGATTACTTCCAACCAAACAACAAAAGAAATCAGCTGT
GGCTGAGACTCCAAACTGCTGGAAATGTAGACCACGTAGGCCTCGGCACTGCGTTCGAAAACAGTATATACGACCAG
GAATACAATATCCGTGTAACCATGTATGTACAATTCAGAGAATTTAATCTTAAAGACCCCCCACTTAACCCT--------

>EU755381_pcv2b

ATGACGTATCCAAGGAGGCGTTACCGGAGAAGAAGACACCGCCCACGCAGCCATCTTGGCCAGATCCTCCGCCGCCG
CCCCTGGCTCGTCCACCCCCGCCACCGTTACCGCTGGAGAAGGAAAAATGGCATCTTCAACACCCGCCTCTCCCGCACC
TTCGGATATACTATCAAACGAACCACAGTCAAAACGCCCTCCTGGGCGGTGGACATGATGAGATTCAATATTAATGAC
TTTCTTCCCCCAGGAGGGGGCTCAAACCCCCGCTCTGTGCCCTTTGAATACTACAGAATAAGAAAGGTTAAGGTTGAA
TTCTGCCCCTGCTCCCCGATCACCCAGGGTGACAGGGGAGTGGGCTCCAGTGCTGTTATTCTAGATGATAACTTTGTA
ACAAAGGCCACAGCCCTCACCTATGACCCCTATGTAGACTACTCCTCCCGCCATACCATAACCCAGCCCTTCTCCTACCA
CTCCCGCTACTTTACCCCCAAACCTGTCCTAGATTCCACTATTGATTACTTCCAACCAAACAACAAAAGAAATCAGCTGT
GGCTGAGACTACAAACTGCTGGAAATGTAGACCACGTAGGCCTCGGCACTGCGTTCGAAAACAGTATATACGACCAG
GAATACAATATCCGTGTAACCATGTATGTACAATTCAGAGAATTTAATCTTAAAGACCCCCCACTTAAACCC--------

>EU755380_pcv2b

ATGACGTATCCAAGGAGGCGTTACCGGAGAAGAAGACACCGCCCACGCAGCCATCTTGGCCAGATCCTCCGCCGCCG
CCCCTGGCTCGTCCACCCCCGCCACCGTTACCGCTGGAGAAGGAAAAATGGCATCTTCAACACCCGCCTCTCCCGCACC
TTCGGATATACTATCAAACGAACCACAGTCAAAACGCCCTCCTGGGCGGTGGACATGATGAGATTCAATATTAATGAC
TTTCTTCCCCCAGGAGGGGGCTCAAACCCCCGCTCTGTGCCCTTTGAATACTACAGAATAAGAAAGGTTAAGGTTGAA
TTCTGGCCCTGCTCCCCGATCACCCAGGGTGACAGGGGAGTGGGCTCCAGTGCTGTTATTCTAGATGATAACTTTGTA
ACAAAGGCCACAGCCCTCACCTATGACCCCTATGTAAACTACTCCTCCCGCCATACCATAACCCAGCCCTTCTCCTACCA
CTCCCGCTACTTTACCCCCAAACCTGTCCTAGATTCCACTATTGATTACTTCCAACCAAACAACAAAAGAAATCAGCCGT
GGCTGAGACTACAAACTGCTGGAAATGTAGACCACGTAGGCCTCGGCACTGCGTTCGAAAACAGTATATACGACCAG
GAATACAATATCCGTGTAACCATGTATGTACAATTCAGAGAATTTAATCTTAAAGACCCCCCACTTAAACCC--------

>EU755378_pcv2b

ATGACGTATCCAAGGAGGCGTTACCGGAGAAGAAGACACCGCCCACGCAGCCATCTTGGCCAGATCCTCCGCCGCCG
CCCCTGGCTCGTCCACCCCCGCCACCGTTACCGCTGGAGAAGGAAAAATGGCATCTTCAACACCCGCCTCTCCCGCACC
TTCGGATATACTATCAAACGAACCACAGTCAAGACGCCCTCCTGGGCGGTGGACATGATGAGATTCAATATTAATGAC
TTTCTTCCCCCAGGAGGGGGCTCAAACCCCCGCTCTGTGCCCTTTGAATACTACAGAATAAGAAAGGTTAAGGTTGAA
TTCTGGCCCTGCTCCCCGATCACCCAGGGTGACAGGGGAGTGGGCTCCAGTGCTGTTATTCTAGATGATAACTTTGTA
ACAAAGGCCACAGCCCTCACCTATGACCCCTATGTAAACTACTCCTCCCGCCATACCATAACCCAGCCCTTCTCCTACCA
CTCCCGCTACTTTACCCCCAAACCTGTCCTAGATTCCACTATTGATTACTTCCAACCAAACAACAAAAGAAATCAGCTGT
GGCTGAGACTACAAACTGCTGGAAATGTAGACCACGTAGGCCTCGGCACTGCGTTCGAAAACAGTATATACGACCAG
GAATACAATATCCGTGTAACCATGTATGTACAATTCAGAGAATTTAATCTTAAAGACCCCCCACTTAAACCC--------

>EU755379_pcv2b

ATGACGTATCCAAGGAGGCGTTACCGGAGAAGAAGACACCGCCCACGCAGCCATCCTGGCCAGATCCTCCGCCGCCG
CCCCTGGCTCGTCCACCCCCGCCACCGTTACCGCTGGAGAAGGAAAAATGGCATCTTCAACACCCGCCTCTCCCGCACC
TTCGGATATACTATCAAACGAACCACAGTCAAAACGCCCTCCTGGGCGGTGGACATGATGAGATTCAATATTAATGAC
TTTCTTCCCCCAGGAGGGGGCTCAAACCCCCGCTCTGTGCCCTTTGAATACTACAGAATAAGAAAGGTTAAGGTTGAA
TTCTGGCCCTGCTCCCCGATCACCCAGGGTGACAGGGGAGTGGGCTCCAGTGCTGTTATTCTAGATGATAACTTTGTA
ACAAAGGCCACAGCCCTCACCTATGACCCCTATGTAAACTACTCCTCCCGCCATACCATAACCCAGCCCTTCTCCTACCA
CTCCCGCTACTTTACCCCCAAACCTGTCCTAGATTCCACTATTGATTACTTCCAACCAAACAACAAAAGAAATCAGCTGT
GGCTGAGACTACAAACTGCTGGAAATGTAGACCACGTAGGCCTCGGCACTGCGTTCGAAAACAGTATATACGACCAG
GAATACAATATCCGTGTAACCATGTATGTACAATTCAGAGAATTTAATCTTAAAGACCCCCCACTTAAACCC--------

>EU755375_pcv2b

ATGACGTATCCAAGGAGGCGTTACCGGAGAAGAAGACACCGCCCACGCAGCCATCTTGGCCAGATCCTCCGCCGCCG
CCCCTGGCTCGTCCACCCCCGCCACCGTTACCGCTGGAGAAGGAAAAATGGCATCTTCAACACCCGCCTCTCCCGCACC
TTCGGATATACTATCAAACGAACCACAGTCAAAACGCCCTCCTGGGCGGTGGACATGATGAGATTCAATATTAATGAC
TTTCTTCCCCCAGGAGGGGGCTCAAACCCCCGCTCTGTGCCCTTTGAATACTACAGAATAAGAAAGGTTAAGGTTGAA
TTCTGGCCCTGCTCCCCGATCACCCAGGGTGACAGGGGAGTGGGCTCCAGTGCTGTTATTCTAGATGATAACTTTGTA
ACAAAGGCCACAGCCCTCACCTATGACCCCTATGTAAACTACTCCTCCCGCCATACCATAACCCAGCCCTTCTCCTACCA
CTCCCGCTACTTTACCCCCAAACCTGTCCTAGATTCCACTATTGATTACTTCCAACCAAACAACAAAAGAAATCAGCTGT
GGCTGAGACTACAAACTGCTGGAAATGTAGACCACGTAGGCCTCGGCACTGCGTTCGAAAACAGTATATACGACCAG
GAATACAATATCCGTGTAACCATGTATGTACAATTCAGAGAATTTAATCTTAAAGACCCCCCACTTAAACCC--------

>HQ735207_pcv2b

ATGACGTATCCAAGGAGGCGTTACCGGAGAAGAAGACACCGCCCCCGCAGCCATCTTGGCCAGATCCTCCGCCGCCG
CCCCTGGCTCGTCCACCCCCGCCACCGTTACCGCTGGAGAAGGAAAAATGGCATCTTCAACACCCGCCTCTCCCGCACC
TTCGGATATACTATCAAACGAACCACAGTCAAAACGCCCTCCTGGGCGGTGGACATGATGAGATTCAATATTAATGAC
TTTCTTCCCCCAGGAGGGGGCTCAAACCCCCGCTCTGTGCCCTTTGAATACTACAGAATAAGAAAGGTTAAGGTTGAA
TTCTGGCCCTGCTCCCCGATCACCCAGGGTGACAGGGGAGTGGGCTCCAGTGCTGTTATTCTAGATGATAACTTTGTA
ACAAAGGCCACAGCCCTCACCTATGACCCCTATGTAAACTACTCCTCCCGCCATACCATAACCCAGCCCTTCTCCTACCA
CTCCCGCTACTTTACCCCCAAACCTGTCCTAGATTCCACTATTGATTACTTCCAACCAAACAACAAAAGAAATCAGCTGT
GGCTGAGACTACAAACTGCTGGAAATGTAGACCACGTAGGCCTCGGCACTGCGTTCGAAAACAGTATATACGACCAG
GAATACAATATCCGTGTAACCATGTATGTACAATTCAGAGAATTTAATCTTAAAGACCCCCCACTTAAACCC--------

>EU755373_pcv2b

ATGACGTATCCAAGGAGGCGTTACCGGAGAAGAAGACACCGCCCCCGCAGCCATCTTGGGCAGATCCTCCGCCGCCG
CCCCTGGCTCGTCCACCCCCGCCACCGTTACCGCTGGAGAAGGAAAAATGGCATCTTCAACACCCGCCTCTCCCGCACC
TTCGGATATACTATCAAACGAACCACAGTCAAAACGCCCTCCTGGGCGGTGGACATGATGAGATTCAATATTAATGAC
TTTCTTCCCCCAGGAGGGGGCTCAAACCCCCGCTCTGTGCCCTTTGAATACTACAGAATAAGAAAGGTTAAGGTTGAA
TTCTGGCCCTGCTCCCCGATCACCCAGGGTGACAGGGGAGTTGGCTCCAGTGCTGTTATTCTAGATGATAACTTTGTAA
CAAAGGCCACAGCCCTCAGCTATGACCCCTATGTAAACTACTCCTCCCGCCATACCATAACCCAGCCCTTCTCCTACCAA
TCCCGCTACTTTACACCTAAAGCTGTCCTAGATTCCACTATTGATTACTTCCAAACAAACAAAAAAAGAAATCAGCTGT
GGCTGACACTACAAACTGCTGAAAATGTAGACCACGTAGGCCTCGGCACTGCGTTCGAAAACAGTATATACGACCAG
GAATACAATATCCGTGTAACCATGTATGTACAATCTAGAGAATTTAATCTTAAAGACCCCCCACTTAAACCC--------

>EU755377_pcv2b

ATGACGTATCCAAGGAGGCGTTACCGGAGAAGAAGACACCGCCCCCGCAGCCATCTTGGCCAGATCCTCCGCCGCCG
CCCCTGGCTCGTCCACCCCCGCCACCGTTACCGCTGGAGAAGGAAAAATGGCATCTTCAACACCCGCCTCTCCCGCACC
TTCGGATATACTATCAAACGAACCACAGTCAAAACGCCCTCCTGGGCGGTGGACATGATGAGATTCAATATTAATGAC
TTTCTTCCCCCAGGAGGGGGCTCAAACCCCCGCTCTGTGCCCTTTGAATACTACAGAATAAGAAAGGTTAAGGTTGAA
TTCTGGCCCTGCTCCCCGATCACCCAGGGTGACAGGGGAGTGGGCTCGAGTGCTGTTATTCTAGATGATAACTTTGTA
ACAAAGGCCACAGCCCTCACCTATGACCCCTATGTAAACTACTCCTCCCGCCATACCATAACCCAGCCCTTCTCCTACCA
CTCCCGCTACTTTACCCCCAAAGCTGTCCTAGATTCCACTATAGATTACTTCCAACCAAACAACAAAAGAAATCAGCTGT
GGCTGAGACTACAAACTGCTGGAAATGTAGACCACGTAGGCCTCGGCACTGCGTTCGAAAACAGTATATACGACCAA
GAATACAATATCCGTGTAACCATGTATGTACAATTCAGAGAATTTAATCCTAAAGACCCCCCACTTAAACCC--------

>DQ534442_pcv2b

ATGACGTATCCAAGGAGGCGTTACCGGAGAAGAAGACACCGCCCCCGCAGCCATCTTGGCCAGATCCTCCGCCGCCG
CCCCTGGCTCGTCCACCCCCGCCACCGTTACCGCTGGAGAAGGAAAAATGGCATCTTCAACACCCGCCTCTCCCGCACC
TTCGGATATACTATCAAGCGAACCACAGTCAAAACGCCCTCCTGGGCGGTGGACATGATGAGATTCAATATTAATGAC
TTTCTTCCCCCAGGAGGGGGCTCAAACTCCCGCTCTGTGCCCTTTGAATACTACAGAATAAGAAAGGTTAAGGTTGAA
TTCTGGCCCTGCTCCCCGATCACCCAGGGTGACAGGGGAGTGGGCTCCAGTGCTGTTATTCTAGATGATAACTTTGTA
ACAAAGGCCACAGCCCTCACCTATGACCCCTATGTAAACTACTCCTCCCGCCATACCATAACCCAGCCCTTCTCCTACCA
CTCCCGCTACTTTACCCCCAAACCTGTCCTAGATTCCACTATTGATTACTTCCAACCAAACAACAAAAGAAATCAGCTGT
GGCTGAGACTACAAACTGCTGGAAATGTAGACCACGTAGGCCTCGGCACTGCGTTCGAAAACAGTATATACGACCAG
GAATACAATATCCGTGTAACCATGTATGTACAATTCAGAGAATTTAATCTTAAAGACCCCCCACTTAAACCC--------

>EF184227_pcv2b

ATGACGGATCCAAGGAGGCGTTACCGGAGAAGAAGACACCGCCCCCGCAGCCATCTTGGCCAGATCCTCCGCCGCCG
CCCCTGGCTCGTCCACCCCCGCCACCGTTACCGCTGGAGAAGGAAAAATGGCATCTTCAACACCCGCCTCTCCCGCACC
TTCGGATATACTATCAAGCGAACCACAGTCAAAACGCCCTCCTGGGCGGTGGACATGATGAGATTCAATATTAATGAC
TTTCTTCCCCCAGGAGGGGGCTCAAACCCCCGCTCTGTGCCCTTTGAATACTACAGAATAAGAAAGGTTAAGGTTGAA
TTCTGGCCCTGCTCCCCGATCACCCAGGGTGACAGGGGAGTGGGCTCCAGTGCTGTTATTCTAGATGATAACTTTGTA
ACAAAGGCCACAGCCCTCACCTATGACCCCTATGTAAACTACTCCTCCCGCCATACCATAACCCAGCCCTTCTCCTACCA
CTCCCGCTACTTTACCCCCAAACCTGTCCTAGATTCCACTATTGATTACTTCCAACCAAACAACAAAAGAAATCAGCTGT
GGCTGAGACTACAAACTGCTGGAAATGTAGACCACGTAGGCCTCGGCACTGCGTTCGAAAACAGTATATACGACCAG
GAATACAATATCCGTGTAACCATGTATGTACAATTCAGAGAATTTAATCTTAAAGACCCCCCACTTAAACCC--------

>EF067852_pcv2b

ATGACGTATCCAAGGAGGCGTTACCGGAGAAGAAGACACCGCCCCCGCAGCCATCTTGGCCAGATCCTCCGCCGCCG
CCCCTGGCTCGTCCACCCCCGCCACCGTTACCGCTGGAGAAGGAAAAATGGCATTTTCAACACCCGCCTCTCCCGCACC
TTCGGATATACTATCAAGCGAACCACAGTCAAAACGCCCTCCTGGGCGGTGGACATGATGAGATTCAATATTAATGAC
TTTTTTTCCCCAGGAGGGGGCTCAAACCCCCGCTCTGTGCCCTTTGAATACTACAGAATAAGAAAGGTTAAGGTTGAA
TTCTGGCCCTGCTCCCCGATCACCCAGGGTGACAGGGGAGTGGGCTCCAGTGCTGTTATTTTAGATGATAACTTTGTA
ACAAAGGCCACAGCCCTCACCTATGACCCCTATGTAAACTACTCCTCCCGCCATACCATAACCCAGCCCTTCTCCTACCA
CTCCCGCTACTTTACCCCCAAACCTGTCCTAGATTCCACTATTGATTACTTCCAACCAAACAACAAAAGAAATCAGCTGT
GGCTGAGACTACAAACTGCTGGAAATGTAGACCACGTAGGCCTCGGCACTGCGTTCGAAAACAGTATATACGACCAG
GAATACAATATCCGTGTAACCATGTATGTACAATTCAGAGAATTTAATTTTAAAGACCCCCCACTTAAACCC--------

>GQ449672_pcv2b

ATGACGTATCCAAGGAGGCGTTACCGGAGGAGAAGACACCGCCCCCGCAGCCATCTTGGCCAGATCCTCCGCCGCCG
CCCCTGGCTCGTCCACCCCCGCCACCGTTACCGCTGGAGAAGGAAAAATGGCATCTTCAACACCCGCCTCTCCCGCACC
TTCGGATATACTATCAAGCGAACCACAGTCAAAACGCCCTCCTGGGCGGTGGACATGATGAGATTCAATATTAATGAC
TTTCTTCCCCCAGGAGGGGGCTCAAACCCCCGCTCTGTGCCCTTTGAATACTACAGAATAAGAAAGGTTAAGGTTGAA
TTCTGGCCCTGCTCCCCGATCACCCAGGGTGACAGGGGAGTGGGCTCCAGTGCTGTTATTCTAGATGATAACTTTGTA
ACAAAGGCCACAGCCCTCACCTATGACCCCTATGTAAACTACTCCTCCCGCCATACCATAACCCAGCCCTTCTCCTACCA
CTCCCGCTACTTTACCCCCAAACCTGTCCTAGATTCCACTATTGATTACTTCCAACCAAACAACAAAAGAAATCAGCTGT
GGCTGAGACTACAAACTGCTGGAAATGTAGACCACGTAGGCCTCGGCACTGCGTTCGAAAACAGTATATACGACCAG
GAATACAATATCCGTGTAACCATGTATGTACAATTCAGAGAATTTAATCTTAAAGACCCCCCACTTAAACCC--------

>JF317571_pcv2b

ATGACGTATCCAAGGAGGCGTTACCGGAGAAGAAGACACCGCCCCCGCAGCCATCTTGGCCAGATCCTCCGCCGCCG
CCCCTGGCTCGTCCACCCCCGCCACCGTTACCGCTGGAGAAGGAAAAATGGCATCTTCAACACCCGCCTCTCCCGCACC
TTCGGATATACTATCAAGCGAACCACAGTCAAAACGCCCTCCTGGGCGGTGGACATGATGAGATTCAATATTAATGAC
TTTCTTCCCCCAGGAGGGGGCTCAAACCCCCGCTCTGTGCCCTTTGAATACTACAGAATAAGAAAGGTTAAGGTAGAA
TTCTGGCCCTGCTCCCCGATCACCCAAGGTGACAGGGGAGTGGGATCCAGTGCTGTTATTCTAGATGATAACTTTGTA
ACAAAGGCCACAGCCCTCACCTATGACCCCTATGTAAACTACTCCTCCCGCCATACCATAACCCAGCCCTTCTCCTACCA
CTCCCGCTACTTTACCCCCAAACCTGTCCTAGATTCCACTATTGATTACTTCCAACCAAACAACAAAAGAAATCAGCTGT
GGCTGAGACTACAAACTGCTGGAAATGTAGACCACGTAGGCCTCGGCACTGCGTTCGAAAACAGTATATACGACCAG
GAATACAATATCCGTGTAACCATGTATGTACAATTCAGAGAATTTAATCTTAAAGACCCCCCACTTAAACCC--------

>AY539828_pcv2b

ATGACGTATCCAAGGAGGCGTTACCGGAGAAGAAGACACCGCCCCCGCAGCCATCTTGGCCAGATCCTCCGCCGCCG
CCCCTGGCTCGTCCACCCCCGCCATCGTTACCGCTGGAGAAGGAAAAATGGCACCTTCAACACCCGCCTCTCCCGCACC
TTCGGATATACTATCAAGCGAACCACAGTCAAAACGCCCTCCTGGGCGGTGGACATGATGAGATTCAATATTAATGAC
TTTCTTCCCCCAGGAGGGGGCTCAAACCCCCGCTCTGTGCCCTTTGAATACTACAGAATAAGAAAGGTTAAGGTTGAA
TTCTGGCCCTGCTCCCCGATCACCCAGGGTGACAGGGGAGTGGGCTCCAGTGCTGTTATTCTAGATGATAACTTTGTA
ACAAAGGCCACAGCCCTCACCTATGACCCCTATGTAAACTACTCCTCCCGCCATACCATAACCCAGCCCTTCTCCTACCA
CTCCCGCTACTTTACCCCCAAACCTGTCCTAGATTCCACTATTGATTACTTCCAACCAAACAACAAAAGAAATCAGCTGT
GGCTGAGACTACAAACTGCTGGAAATGTAGACCACGTAGGCCTCGGCACTGCGTTCGAAAACAGTATATACGACCAG
GAATACAATATCCGTGTAACCATGTATGTACAATTCAGAGAATTTAATCTTAAAGACCCCCCACTTAAACCC--------

>EF371529_pcv2b

ATGACGTATCCAAGGAGGCGTTACCGGAGAAGAAGACACCGCCCCCGCAGCCATCTTGGCCAGATCCTCCGCCGCCG
CCCCTGGCTCGTCCACCCCCGCCACCGTTACCGCTGGAGAAGGAAAAATGGCATCTTCAACACCCGCCTCTCCCGCACC
TTCGGATATACTATCAAGCGAACCACAGTCAAAACGCCCTCCTGGGCGGTGGACATGATGAGATTCAATATTAATGAC
TTTCTTCCCCCAGGAGGGGGCTCAAACCCCCGCTCTGTGCCCTTTGAATACTACAGAATAAGAAAGGTTAAGGTTGAA
TTCTGGCCCTGCTCCCCGATCACCCAGGGTGACAGGGGAGTGGGCTCCAGTGCTGTTATTCTAGATGATAACTTTGTA
ACAAAGGCCACAGCCCTCACCTATGACCCCTATGTAAACTACTCCTCCCGCCATACCATAACCCAGCCCTTCTCCTACCA
CTCCCGCTACTTTACCCCCAAACCTGTCCTAGATTCCACTATTGATTACTTCCAACCAAACAACAAAAGAAATCAGCTGT
GGCTGAGACTACAAACTGCTGGAAATGTAGACCACGTAGGCCTCGGCACTGCGTTCGAAAACAGTATATACGACCAG
GAATACAATATCCGTGTAACCATGTATGTACAATTCAGAGAATTTAATCTTAAAGACCCCCCACTTAAACCC--------

>AY885225_pcv2b

ATGACGTATCCAAGGAGGCGTTACCGGAGAAGACGACACCGCCCCCGCAGCCATCTTGGCCAGATCCTCCGCCGCCG
CCCCTGGCTCGTCCACCCCCGCCACCGTTACCGCTGGAGAAGGAAAAATGGCATCTTCAACACCCGCCTCTCCCGCACC
TTCGGATATACTATCAAGCGAACCACAGTCAAAACGCCCTCCTGGGCGGTGGACATGATGAGATTCAATATTAATGAC
TTTCTTCCCCCAGGAGGGGGCTCAAACCCCCGCTCTGTGCCCTTTGAATACTACAGAATAAGAAAGGTTAAGGTTGAA
TTCTGGCCCTGCTCCCCGATCACCCAGGGTGACAGGGGAGTGGGCTCCAGTGCTGTTATTCTAGATGATAACTTTGTA
ACAAAGGCCACAGCCCTCACCTATGACCCCTATGTAAACTACTCCTCCCGCCATACCATAACCCAGCCCTTCTCCTACCA
CTCCCGCTACTTTACCCCCAAACCTGTCCTAGATTCCACTATTGATTACTTCCAACCAAACAACAAAAGAAATCAGCTGT
GGCTGAGACTACAAACTGCTGGAAATGTAGACCACGTAGGCCTCGGCACTGCGTTCGAAAACAGTATATACGACCAG
GAATACAATATCCGTGTAACCATGTATGTACAATTCAGAGAATTTAATCTTAAAGACCCCCCACTTAAACCC--------

>EF371533_pcv2b

ATGACGTATCCAAGGAGGCGTTACCGGAGAAGAAGACACCGCCCCCGCAGCCATCTTGGCCAGATCCTCCGCCGCCG
CCCCTGGCTCGTCCACCCCCGCCACCGTTACCGCTGGAGAAGGAAAAATGGCATCTTCAACACCCGCCTCTCCCGCACC
TTCGGATATACTATCAAGCGAACCACAGTCAAAACGCCCTCCTGGGCGGTGGACATGATGAGATTCAATATTAATGAC
TTTCTTCCCCCAGGAGGGGGCTCAAACCCCCGCTCTGTGCCCTTTGAATACTACAGAATAAGAAAGGTTAAGGTTGAA
TTCTGGCCCTGCTCCCCGATCACCCAGGGTGACAGGGGAGTGGGCTCCAGTGCTGTTATTCTAGATGATAACTTTGTA
ACAAAGGCCACAGCCCTCACCTATGACCCCTATGTAAACTACTCCTCCCGCCATACCATAACCCAGCCCTTCTCCTACCA
CTCCCGCTACTTTACCCCCAAACCTGTCCTAGATTCCACTATTGATTACTTCCAACCAAACAACAAAAGAAATCAGCTGT
GGCTGAGACTACAAACTGCTGGAAATGTAGACCACGTAGGCCTCGGCACTGCGTTCGAAAACAGTATATACGACCAG
GAATACAATATCCGTGTAACCATGTATGTACAATTCAGAGAATTTAATCTTAAAGACCCCCCACTTAAACCC--------

>EF371528_pcv2b

ATGACGTATCCAAGGAGGCGTTACCGGAGAAGAAGACACCGCCCCCGCAGCCATCTTGGCCAGATCCTCCGCCGCCG
CCCCTGGCTCGTCCACCCCCGCCACCGTTACCGCTGGAGAAGGAAAAATGGCATCTTCAACACCCGCCTCTCCCGCACC
TTCGGATATACTATCAAGCGAACCACAGTCAAAACGCCCTCCTGGGCGGTGGACATGATGAGATTCAATATTAATGAC
TTTCTTCCCCCAGGAGGGGGCTCAAACCCCCGCTCTGTGCCCTTTGAATACTACAGAATAAGAAAGGTTAAGGTTGAA
TTCTGGCCCTGCTCCCCGATCACCCAGGGTGACAGGGGAGTGGGCTCCAGTGCTGTTATTCTAGATGATAACTTTGTA
ACAAAGGCCACAGCCCTCACCTATGACCCCTATGTAAACTACTCCTCCCGCCATACCATAACCCAGCCCTTCTCCTACCA
CTCCCGCTACTTTACCCCCAAACCTGTCCTAGATTCCACTATTGATTACTTCCAACCAAACAACAAAAGAAATCAGCTGT
GGCTGAGACTACAAACTGCTGGAAATGTAGACCACGTAGGCCTCGGCACTGCGTTCGAAAACAGTATATACGACCAG
GAATACAATATCCGTGTAACCATGTATGTACAATTCAGAGAATTTAATCTTAAAGACCCCCCACTTAAACCC--------

>EU755376_pcv2b

ATGACGTATCCAAGGAGGCGTTACCGGAGAAGAAGACACCGCCCCCGCAGCCATCTTGGCCAGATCCTCCGCCGCCG
ACCCTGGCTCGTCCACCCCCGCCACCGTTACCGCTGGAGAAGGAAAAATGGCATCTTCAACACCCGCCTCTCCCGCACC
TTCGGATATACTATCAAGCGAACCACAGTCAAAACGCCCTCCTGGGCGGTGGACATGATGAGATTCAATATTAATGAC
TTTCTTCCCCCAGGAGGGGGCTCAAACCCCCGCTCTGTGCCCTTTGAATACTACAGAATAAGAAAGGTTAAGGTTGAA
TTCTGGCCCTGCTCCCCGATCACCCAGGGTGACAGGGGAGTGGGCTCCAGTGCTGTTATTCTAGATGATAACTTTGTA
ACAAAGGCCACAGCCCTCACCTATGACCCCTATGTAAACTACTCCTCCCGCCATACCATAACCCAGCCCTTCTCCTACCA
CTCCCGCTACTTTACCCCCAAACCTGTCCTAGATTCCACTATTGATTACTTCCAACCAAACAACAAAAGAAATCAGCTGT
GGCTGAGACTACAAACTACTGGAAATGTAGACCACGTAGGCCTCGGCACTGCGTTCGAAAACAGTATATACGACCAG
GAATACAATATCCGTGTAACCATGTATGTACAATTCAGAGAATTTAATCTTAAAGACCCCCCACTTAAACCC--------

>FJ755686_pcv2b

ATGACGTATCCAAGGAGGCGTTACCGGAGAAGAAGACACCGCCCCCGCAGCCATCTTGGCCAGATCCTCCGCCGCCG
CCCCTGGCTCGTCCACCCCCGCCACCGTCACCGCTGGAGAAGGAAAAATGGCATCTTCAACACCCGCCTCTCCCGCAC
CTTCGGATATACTATCAAGCGAACCACAGTCAAAACGCCCTCCTGGGCGGTGGACATGATGAGATTCAATATTAATGA
CTTTCTTCCCCCAGGAGGGGGCTCAAACCCCCGCTCTGTGCCCTTTGAATACTACAGAATAAGAAAGGTTAAGGTTGA
ATTCTGGCCCTGCTCCCCGATCACCCAGGGTGACAGGGGAGTGGGCTCCAGTGCTGTTATTCTAGATGATAACTTTGT
AACAAAGGCCACAGCCCTCACCTATGACCCCTATGTAAACTACTCCTCCCGCCATACCATAACCCAGCCCTTCTCCTACC
ACTCCCGCTACTTTACCCCCAAACCTGTCCTAGATTCCACTATTGATTACTTCCAACCAAACAACAAAAGAAATCAGCTG
TGGTTGAGACTACAAACTGCTGGAAATGTAGACCACGTAGGCCTCGGCACTGCGTTCGAAAACAGTATATACGACCA
GGAATACAATATCCGTGTAACCATGTATGTACAGTTCAGAGAATTTAATCTTAAAGACCCCCCACTTAAACCC--------

>EF371535_pcv2b

ATGACGTATCCAAGGAGGCGTTACCGGAGAAGAAGACACCGCCCCCGCAGCCATCTTGGCCAGATCCTCCGCCGCCG
CCCCTGGCTCGTCCACCCCCGCCACCGTTACCGCTGGAGAAGGAAAAATGGCATCTTCAACACCCGCCTCTCCCGCACC
TTCGGATATACTATCAAGCGAACCACAGTCAAAACGCCCTCCTGGGCGGTGGACATGATGAGATTCAATATTAATGAC
TTTCTTCCCCCAGGAGGGGGCTCAAACCCCCGCTCTGTGCCCTTTGAATACTACAGAATAAGAAAGGTTAAGGTTGAA
TTCTGGCCCTGCTCCCCGATCACCCAGGGTGACAGGGGACTGGGCTCCAGTGCTGTTATTCTAGATGATTACTTTGTAA
CAAAGGCCACAGCCCTCACCTATTATCCCTATGTAAACTACTCCTCCCGCCATACCATAACCCAACCCTTCTCCTACCAC
TCCCGCTACTTTACCCCCAAACCTGTCCTAGATTCCACTATTGATTACTTCCAACCAAACAACAAAAGAAATCAGCTGTG
GCTGAGACTACAAACTGCTGGAAATGTAGACCACGTAGGCCTCGGCACTGCGTTCGAAAACAGTATATACGACCAGG
AATACAATATCCGTGTAACCATGTATGTACAATTCAGAGAATTTAATCTTAAAGACCCCCCACTTAAACCC--------

>EF371534_pcv2b

ATGACGTATCCAAGGAGGCGTTACCGGAGAAGAAGACACCGCCCCCGCAGCCATCTTGGCCAGATCCTCCGCCGCCG
CCCCTGGCTCGTCCACCCCCGCCACCGTTACCGCTGGAGAAGGAAAAATGGCATCTTCAACACCCGCCTCTCCCGCACC
TTCGGATATACTATCAAGCGAACCACAGTCAAAACGCCCTCCTGGGCGGTGGACATGATGAGATTCAATATTAATGAC
TTTCTTCCCCCAGGAGGGGGCTCAAACCCCCGCTCTGTGCCCTTTGAATACTACAGAATAAGAAAGGTTAAGGTTGAA
TTCTGGCCCTGCTCCCCGATCACCCAGGGTGACAGGGGAGTGGGCTCCAGTGCTGTTATTCTAGATTATGACTTTGTA
ACAAAGGCCACAGCCCTCACCTATGACCCCTATGTAAACTACTCCTCCCGCCATACCATAACCCAGCCCTTCTCCTACCA
CTCCCGCTACTTTACCCCCAAACCTGTCCTAGATTCCACTATTGATTACTTCCAACCAAACAACAAAAGAAATCAGCTGT
GGCTGAGACTACAAACTGCTGGAAATGTAGACCACGTAGGCCTCGGCACTGCGTTCGAAAACAGTATATACGACCAG
GAATACAATATCCGTGTAACCATGTATGTACAATTCAGAGAATTTAATCTTAAAGACCCCCCACTTAAACCC--------

>EF184228_pcv2b

ATGACGTATCCAAGGAGGCGTTACCGGAGAAGAAGACACCGCCCCCGCAGCCATCTTGGCCAGATCCTCCGCCGCCG
CCCCTGGCTCGTCCACCCCCGCCACCGTTACCGCTGGAGAAGGAAAAATGGCATCTTCAACACCCGCCTCTCCCGCACC
TTCGGATATACTATCAAGCGAACCACAGTCAAAACGCCCTCCTGGGCGGTGGACATGATGAGATTCAATATTAATGAC
TTTCTTCCCCCAGGAGGGGGCTCAAACCCCCGCTCTGTGCCCTTTGAATACTACAGAATAAGAAAGGTTAAGGTTGAA
TTCTGGCCCTGCTCCCCGATCACCCAGGGTGACAGGGGAGTGGGCTCCAGTGCTGTTATTCTAGATTATGACTTTGTA
ACAAAGGCCACAGCCCTCACCTATGACCCCTATGTAAACTACTCCTCCCGCCATACCATAACCCAGCCCTTCTCCTACCA
CTCCCGCTACTTTACCCCCAAACCTGTCCTAGATTCCACTATTGATTACTTCCAACCAAACAACAAAAGAAATCAGCTGT
GGCTGAGACTACAAACTGCTGGAAATGTAGACCACGTAGGCCTCGGCACTGCGTTCGAAAACAGTATATACGACCAG
GAATACAATATCCGTGTAACCATGTATGTACAATTCAGAGAATTTAATCTTAAAGACCCCCCACTTAAACCC--------

>EF371540_pcv2b

ATGACGTATCCAAGGAGGCGTTACCGGAGAAGAAGACACCGCCCCCGCAGCCATCTTGGCCAGATCCTCCGCCGCCG
GCCCTGGCTCGTCCACCCCCGCCACCGTTACCGCTGGAGAAGGAAAAATGGCATCTTCAACACCCGCCTCTCCCGCAC
CTTCGGATATACTATCAAGCGAACCACAGTCAAAACGCCCTCCTGGGCGGTGGACATGATGAGATTCAATATTAATGA
CTTGCTTCCCCCAGGAGGGGGCTCAAACCCCCGCTCTGTGCCCTTTGAATACTACAGAATAAGAAAGGTTAAGGTTGA
ATTCTGGCCCTGCTCCCCGATCACCCAGGGTGACAGGGGAGAGGGCTCCAGTGCTGTTATTCTAGAATATGACTTTGT
AACACAGGCCACAGCCCTCACCTATGACCCCTATGTAAACTACTCCTCCCGCCATACCATAACCCAGCCCTTCTCCTACC
ACTCCCGCTACTTTACCCCCAAACCTGTCCTAGATTCCACTATTGATTACTTCCAACCAAACAACAAAAGAAATCAGCTG
TGGCTGAGACTACAAACTGCTGGAAATGTAGACCACGTTGGCCTCGGCACTGCGTTCGAAAACAGTATATACGACCA
GGAATACAATATCCGTGTAACCTTGTTTGTTCAATTCAGAGAATTTAATCTTAAAGACCCCCCACTTAAACCT--------

>EF371536_pcv2b

ATGACGTATCCAAGGAGGCGTTACCGGAGAAGAAGACACCTGCCCCGCAGCCATCTTGTGCAGATCCTCCGCCGCCG
CCCCTGGCTCGTCCACCCCCGCCACCGTTACCGCTGGAGAAGGAAAAATGGCATCTTCAACACCCGCCTCTCCCGCACC
TTCGGATATACTATCAAGCGAACCACAGTCAAAACGCCCTCCTGGGCGGTGGACATGATGAGATTCAATATTAATGAC
TTTCTTCCCCCAGGAGGGGGCTCAAACCCCCGCTCTGTGCCCTTTGAATACTACAGAATAAGAAAGGTTAAGGTTGAA
TTCTGGCCCTGCTCCCCGATCACCCAGGGTGACAGGGGAGTGGGCTCCAGTGCTGTTATTCTAGATGATAACTTTGTA
ACAAAGGCCACAGCCCTCACCTATGACCCCTATGTAAACTACTCCTCCCGCCATACCATAACCCAGCCCTTCTCCTACCA
CTCCCGCTACTTTACCCCCAAACCTGTCCTAGATTCCACTATTGATTACTTCCAACCAAACAACAAAAGAAATCAGTTGT
GGTTGAGACTACAAACTGCTGGAAATGTTGACCACGTTGGCCTCGGGACTGCGTTCGAAAACAGTATATACGACCAG
GAATACAATTTCCGTGTAACCTTGTTTGTACAATTCAGAGAATTTAATCTTAAAGACCCCCCACTTAAACCC--------

>EU186062_pcv2b

ATGACGTACCCAAGGAGGCGTTACCGGAGAAGAAGACACCGCCCCCGCAGCCATCTTGGCCAGATCCTCCGCCGCCG
CCCCTGGCTCGTCCACCCCCGCCACCGTTACCGCTGGAGAAGGAAAAATGGCATCTTCAACACCCGCCTCTCCCGCACC
TTCGGATATACTATCAAGCGAACCACAGTCAAAACGCCCTCCTGGGCGGTGGACATGATGAGATTCAATATTAATGCT
TTTCTTCCCCCAGGAGGGGGCTCAAACCCCCGCTCTGTGCCCTTTGAATACTACAGAATAAGAAAGGTTAAGGTTGAA
TTCTGGCCCTGCTCCCCGATCACCCAGGGTGACAGGGGAGTGGGCTCCAGTGCTGTTATACTAGATGATAACTTTGTA
ACAAAGGCCACAGCCCTCACCTATGACCCCTATGTAAACTACTCCTCCCGCCATACCATAACCCAGCCCTTCTCCTACCA
CTCCCGCTACTTTACCCCCAAACCTGTCCTAGATTCCACTATTGATTACTTCCAACCAAACAACAAAAGAAATCAGCTGT
GGCTGAGACTACAAACTGCTGGAAATGTAGACCACGTAGGCCTTGGCACTGCGTTCGAAAACAGTATATACGAGAAG
GAATACAATATCCGTGTAACCATGGAAGTCAATCAGAGAATTAATCTAGAAAAAGACCCCCCACTTAAACCATTA-----

>GQ449670_pcv2b

ATGACGTATCCAAGGAGGCGTTACCGGAGAAGAAGACACCGCCCCCGCAGCCATCTTGGCCAGATCCTCCGCCGCCG
CCCCTGGCTCGTCCACCCCCGCCACCGTTACCGCTGGAGAAGGAAAAATGGCATCTTCAACACCCGCCTCTCCCGCACC
TTCGGATATACTATCAAGCGAACCACAGTCAAAACGCCCTCTTGGGCGGTGGACATGATGAGATTCAATATTAATGAT
TTTCTTCCCCCAGGAGGGGGCTCAAACCCCCGCTCTGTGCCCTTTGAATACTACAGAATAAGAAAGGTTAAGGTTGAA
TTCTGGCCCTGCTCCCCGATCACCCAGGGTGACAGGGGAGTGGGCTCCAGTGCTGTTATTCTAGATGATAACTTTGTA
ACAAAGGCCACAGCCCTCACCTATGACCCCTATGTAAACTACTCCTCCCGCCATACCATAACCCAGCCCTTCTCCTACCA
CTCCCGCTACTTTACCCCCAAACCTGTCCTAGATTCCACTATTGATTACTTCCAACCAAACAACAAAAGAAATCAGCTGT
GGCTGAGACTACAAACTGCTGGAAATGTAGACCACGTAGGCCTCGGCACTGCGTTAGAAAACAGTATATACGACCAG
GAATACAATATCCGTGTAACCATGTATGTACAATTCAGAGAATTTAATCTTAAAGACCCCCCACTTAAACCC--------

>EU350548_pcv2b

ATGACGTATCCAAGGAGGCGTTACCGGAGAAGAAGACACCGCCCCCGCAGCCATCTTGGCCAGATCCTCCGCCGCCG
CCCCTGGCTCGTCCACCCCCGCCACCGTTACCGCTGGAGAAGGAAAAATGGCATCTTCAACACCCGCCTCTCCCGCACC
TTCGGATATACTATCAAGCGAACCACAGTCAAAACGCCCTCCTGGGCGGTGGACATGATGAGATTCAATGTTAATGAT
TTTCTTCCCCCAGGAGGGGGCTCAAACCCCCGCTCTGTGCCCTTTGAATACTACAGAATAAGAAAGGTTAAGGTTGAA
TTCTGGCCCTGCTCCCCGATCACCCAGGGTGACAGGGGAGTGGGCTCCAGTGCTGTTATTCTAGATGATAACTTTGTA
ACAAAGGCCACAGCCCTCACCTATGACCCCTATGTAAACTACTCCTCCCGCCATACCATAACCCAGCCCTTCTCCTACCA
CTCCCGCTACTTTACCCCCAAACCTGTGCTAGATTCCACTATTGATTACTTCCAACCAAACAACAAAAGAAATCAGCTGT
GGCTGAGACTACAAACTGCTGGAAATGTAGACCACGTAGGCCTCGGCACTGCGTTCGAAAACAGTATATACGACCAG
GAATACAATATCCGTGTAACCATGTATGTACAATTCAGAGAATTTAATCTTAAAGACCCCCCACTTAAACCC--------

>EF371538_pcv2b

ATGACGTATCCAAGGAGGCGTTACCGGAGAAGAAGACACCTGCCCCGCAGCCATCTTGGCCAGATCCTCCGCCGCCG
CCCCTGGCTCGTCCACCCCCGCCACCGTTACCGCTGGAGAAGGAAAAATGGCATCTTCAACACCCGCCTCTCCCGCACC
TTCGGATATACTATCAAGCGAACCACAGTCAAAACGCCCTCCTGGGCGGTGGACATGATGAGATTCAATATTAATGAC
TTTCTTCCCCCAGGAGGGGGCTCAAACCCCCGCTCTGTGCCCTTTGAATACTACAGAATAAGAAAGGTTAAGGTTGAA
TTCTGGCCCTGCTCCCCGATCACCCAGGGTGACAGGGGAGTGGGCTCCAGTGCTGTTATTCTAGATGATAACTTTGTA
ACAAAGGCCACAGCCCTCACCTATGACCCCTATGTAAACTACTCCTCCCGCCATACCATAACCCAACCCTTCTCCTACCA
CTCCCGCTACTTTACCCCCAAACCTGTCCTAGATTCCACTATTGATTACTTCCAACCAAACAACAAAAGAAATCAGCTGT
GGCTGAGACTACAAACTGCTGGAAATGTAGACCACGTAGGCCTCGGCACTGCCTTCGAAAACAGTATATACGACCAG
GAATACAATATCCGTGTAACCATGTATGTACAATTCAGAGAATTTAATCTTAAAGACCCCCCACTTAAACCT--------

>EF371539_pcv2b

ATGACGTATCCAAGGAGGCGTTACCGGAGAAGAAGACACCTGCCCCGCAGCCATCTTGGCCAGATCCTCCGCCGCCG
CCCCTGGCTCGTCCACCCCCGCCACCGTTACCGCTGGAGAAGGAAAAATGGCATCTTCAACACCCGCCTCTCCCGCACC
TTCGGATATACTATCAAGCGAACCACAGTCAAAACGCCCTCCTGGGCGGTGGACATGATGAGATTCAATATTAATGAC
TTTCTTCCCCCAGGAGGGGGCTCAAACCCCCGCTCTGTGCCCTTTGAATACTACAGAATAAGAAAGGTTAAGGTTGAA
TTCTGGCCCTGCTCCCCGATCACCCAGGGTGACAGGGGAGTGGGCTCCAGTGCTGTTATTCTAGATGATAACTTTGTA
ACAAAGGCCACAGCCCTCACCTATGACCCCTATGTAAACTACTCCTCCCGCCATACCATAACCCAGCCCTTCTCCTACCA
CTCCCGCTACTTTACCCCCAAACCTGTCCTAGATTCCACTATTGATTACTTCCAACCAAACAACAAAAGAAATCAGCTGT
GGCTGAGACTACAAACTGCTGGAAATGTAGACCACGTAGGCCTCGGCACTGCGTTCGAAAACAGTATATACGACCAG
GAATACAATATCCGTGTAACCATGTATGTACAATTCAGAGAATTTAATCTTAAAGACCCCCCACTTAAACCT--------

>EF371537_pcv2b

ATGACGTATCCAAGGAGGCGTTACCGGAGAAGAAGACACCTGCCCCGCAGCCATCTTGGCCAGATCCTCCGCCGCCG
CCCCTGGCTCGTCCACCCCCGCCACCGTTACCGCTGGAGAAGGAAAAATGGCATCTTCAACACCCGCCTCTCCCGCACC
TTCGGATATACTATCAAGCGAACCACAGTCAAAACGCCCTCCTGGGCGGTGGACATGATGAGATTCAATATTAATGAC
TTTCTTCCCCCAGGAGGGGGCTCAAACCCCCGCTCTGTGCCCTTTGAATACTACAGAATAAGAAAGGTTAAGGTTGAA
TTCTGGCCCTGCTCCCCGATCACCCAGGGTGACAGGGGAGTGGGCTCCAGTGCTGTTATTCTAGATGATAACTTTGTA
ACAAAGGCCACAGCCCTCACCTATGACCCCTATGTAAACTACTCCTCCCGCCATACCATAACCCAGCCCTTCTCCTACCA
CTCCCGCTACTTTACCCCCAAACCTGTCCTAGATTCCACTATTGATTACTTCCAACCAAACAACAAAAGAAATCAGCTGT
GGCTGAGACTACAAACTGCTGGAAATGTAGACCACGTAGGCCTCGGCACTGCGTTCGAAAACAGTATATACGACCAG
GAATACAATATCCGTGTAACCATGTATGTACAATTCAGAGAATTTAATCTTAAAGACCCCCCACTTAAACCC--------

>EF184226_pcv2b

ATGACGTATCCAAGGAGGCGTTACCGGAGAAGAAGACACCTGCCCCGCAGCCATCTTGGCCAGATCCTCCGCCGCCG
CCCCTGGCTCGTCCACCCCCGCCACCGTTACCGCTGGAGAAGGAAAAACGGCATCTTCAACACCCGCCTCTCCCGCAC
CTTCGGATATACTATCAAGCGAACCACAGTCAAAACGCCCTCCTGGGCGGTGGACATGATGAGATTCAATATTAATGA
CTTTCTTCCCCCAGGAGGGGGCTCAAACCCCCGCTCTGTGCCCTTTGAATACTACAGAATAAGAAAGGTTAAGGTTGA
ATTCTGGCCCTGCTCCCCGATCACCCAGGGTGACAGGGGAGTGGGCTCCAGTGCTGTTATTCTAGATGATAACTTTGT
AACAAAGGCCACAGCCCTCACCTATGACCCCTATGTAAACTACTCCTCCCGCCATACCATAACCCAGCCCTTCTCCTACC
ACTCCCGCTACTTTACCCCCAAACCTGTCCTAGATTCCACTATTGATTACTTCCAACCAAACAACAAAAGAAATCAGCTG
TGGCTGAGACTACAAACTGCTGGAAATGTAGACCACGTAGGCCTCGGCACTGCGTTCGAAAACAGTATATACGACCA
GGAATACAATATCCGTGTAACCATGTATGTACAATTCAGAGAATTTAATCTTAAAGACCCCCCACTTAAACCC--------

>EU408780_pcv2b

ATGACGTATCCAAGGAGGCGTTACCGGAGAAGAAGACACCTGCCCCGCAGCCATCTTGGCCAGATCCTCCGCCGCCG
CCCCTGGCTCGTCCACCCCCGCCACCGTTACCGCTGGAGAAGGAAAAACGGCATCTTCAACACCCGCCTCTCCCGCAC
CTTCGGATATACTATCAAGCGAACCACAGTCAAAACGCCCTCCTGGGCGGTGGACATGATGAGATTCAATATTAATGA
CTTTCTTCCCCCAGGAGGGGGCTCAAACCCCCGCTCTGTGCCCTTTGAATACTACAGAATAAGAAAGGTTAAGGTTGA
ATTCTGGCCCTGCTCCCCGATCACCCAGGGTGACAGGGGAGTGGGCTCCAGTGCTGTTATTCTAGATGATAACTTTGT
AACAAAGGCCACAGCCCTCACCTATGACCCCTATGTAAACTACTCCTCCCGCCATACCATAACCCAGCCCTTCTCCTACC
ACTCCCGCTACTTTACCCCCAAACCTGTCCTAGATTCCACTATTGATTACTTCCAACCAAACAACAAAAGAAATCAGCTG
TGGCTGAGACTACAAACTGCTGGAAATGTAGACCACGTAGGCCTCGGCACTGCGTTCGAAAACAGTATATACGACCA
GGAATACAATATCCGTGTAACCATGTATGTACAATTCAGAGAATTTAATCTTAAAGACCCCCCACTTAAACCC--------

>HF542107_pcv2b

ATGACGTATCCAAGGAGGCGTTACCGGAGAAGAAGACACCGCCCCCGCAGCCATCTTGGCCAGATCCTCCGCCGCCG
CCCCTGGCTCGTCCACCCCCGCCACCGTTACCGCTGGAGAAGGAAAAATGGCATCTTCAACACCCGCCTCTCCCGCACC
TTCGGATATACTATCAAGCGAACCACAGTCAAAACGCCCTCCTGGGCGGTGGACATGATGAGATTCAATATTAATGAC
TTTCTTCCCCCAGGAGGGGGCTCAAACCCCCGCTCTGTGCCCTTTGAATACTACAGAATAAGAAAGGTTAAGGTTGAA
TTCTGGCCCTGCTCCCCGATCACCCAGGGTGACAGGGGAGTGGGCTCCAGTGCTGTTATTCTAGATGATAACTTTGTA
ACAAAGGCCACAGCCCTCACCTATGACCCCTATGTAAACTACTCCTCCCGCCATACCATAACCCAGCCCTTCTCATACCA
CTCCCGCTACTTTACCCCCAAACCTGTCCTAGATTCCACTATTGATTACTTCCAACCAAACAACAAAAGAAATCAGCTGT
GGCTGAGACTACAAACTACTGGAAATGTAGACCACGTAGGCCTCGGCACTGCGTTCGAAAACAGTATATACGACCAG
GTATACAATATCCGTGTAACCATGTATGTACAATTCAGAGAATTTAATCTTAAAGACCCCCCACTTAAACCT--------

>HQ395027_pcv2b

ATGACGTATACAAGGAGGCGTTACCGGAGAAGAAGACACCGCCCCCGCAGCCATCTTGGCCAGATCCTCCGCCGCCG
CCCCTGGCTCGTCCACCCCCGCCACCGTTACCGCTGGAGAAGGAAAAATGGCATCTTCAACACCCGCCTCTCCCGCACC
TTCGGATATACTATCAAGCGAACCACTGTCAAAACGCCCTCCTGGGCGGTGGACATGATGAGATTCAATATTAATGAC
TTTCTTCCCCCAGGAGGGGGCTCAAACCCCCGCTCTGTGCCCTTTGAATACTACAGAATAAGAAAGGTTAAGGTTGAA
TTCTGGCCCTGCTCCCCGATCACCCAGGGTGACAGGGGAGTGGGCTCCAGTGCTGTTATTCTAGATGATAACTTTGTA
ACAAAGGCCACAGCCCTCACCTATGACCCCTATGTAAACTACTCCTCCCGCCATACCATAACCCAGCCCTTCTCCTACCA
CTCCCGCTACTTTACCCCCAAACCTGTCCTAGATTCCACTATTGATTACTTCCAACCAAACAACAAAAGAAATCAGCTGT
GGCTGAGACTACAAACTACTGGAAATGTAGACCACGTAGGCCTCGGCACTGCGTTCGAAAACAGTATATACGACCAG
GAATACAATATCCGTGTAACCATGTATGTACAATTCAGAGAATTTAATCTTAAAGACCCCCCACTTAAACCT--------

>FN687849_pcv2b

ATGACGTATCCAAGGAGGCGTTACCGGAGAAGAAGACACCGCCCCCGCAGCCATCTTGGCCAGATCCTCCGCCGCCG
CCCCTGGCTCGTCCACCCCCGCCACCGTTACCGCTGGAGAAGGAAAAATGGCATCTTCAACACCCGCCTCTCCCGCACC
TTCGGATATACTATCAAGCGAACCACAGTCAAAACGCCCTCCTGGGCGGTGGACATGATGAGATTCAATATTAATGAC
TTTCTTCCCCCAGGAGGGGGCTCAAACCCCCGCTCTGTGCCCTTTGAATACTACAGAATAAGAAAGGTTAAGGTTGAA
TTCTGGCCCTGCTCCCCGATCACCCAAGGTGACAGGGGAGTGGGCTCCAGTGCTGTTATTCTAGATGATAACTTTGTA
ACAAAGGCCACAGCCCTCACCTATGACCCCTATGTAAACTACTCCTCCCGCCATACCATAACCCAGCCCTTCTCCTACCA
CTCCCGCTACTTTACCCCCAAACCCGTCCTAGATTCCACTATTGATTACTTCCAACCAAACAACAAAAGAAATCAGCTGT
GGCTGAGACTACAAACTGCTGGAAATGTAGACCACGTAGGCCTCGGCACTGCGTTCGAAAACAGCATATACGACCAG
GAATACAATATCCGTGTAACCATGTATGTACAATTCAGAGAATTTAATCTTAAAGACCCCCCACTTAACCCT--------

>FN687848_pcv2b

ATGACGTATCCAAGGAGGCGTTACCGGAGAAGAAGACACCGCCCCCGCAGCCATCTTGGCCAGATCCTCCGCCGCCG
CCCCTGGCTCGTCCACCCCCGCCACCGTTACCGCTGGAGAAGGAAAAATGGCATCTTCAACACCCGCCTCTCCCGCACC
TTCGGATATACTATCAAGCGAACCACAGTCAAAACGCCCTCCTGGGCGGTGGACATGATGAGATTCAATATTAATGAC
TTTCTTCCCCCAGGAGGGGGCTCAAACCCCCGCTCTGTGCCCTTTGAATACTACAGAATAAGAAAGGTTAAGGTTGAA
TTCTGGCCCTGCTCCCCGATCACCCAGGGTGACAGGGGAGTGGGCTCCAGTGCTGTTATTCTAGATGATAACTTTGTA
ACAAAGGCCACAGCCCTCACCTATGACCCCTATGTAAACTACTCCTCCCGCCATACCATAACCCAGCCCTTCTCCTACCA
CTCCCGCTACTTTACCCCCAAACCCGTCCTAGATTCCACTATTGATTACTTCCAACCAAACAACAAAAGAAATCAGCTGT
GGCTGAGACTACAAACTACTGGAAATGTAGACCACGTAGGCCTCGGCACTGCGTTCGAAAACAGCATATACGACCAG
GATTACAATATCCGTGTAACCATGTATGTACAATTCAGAGAATTTAATCTTAAAGACCCCCCACTTAAACCT--------

>AY596822_pcv2b

ATGACGTATCCAAGGAGGCGTTACCGGAGAAGAAGACACCGCCCCCGCAGCCATCTTGGCCAGATCCTCCGCCGCCG
CCCCTGGCTCGTCCACCCCCGCCACCGTTACCGCTGGAGAAGGAAAAATGGCATCTTCAACACCCGCCTCTCCCGCACC
TTCGGATATACTATCAAGCGAACCACAGTCAAAACGCCCTCCTGGGCGGTGGACATGATGAGATTCAATATTAATGAC
TTTCTTCCCCCAGGAGGGGGCTCAAACCCCCGCTCTGTGCCCTTTGAATACTACAGAATAAGAAAGGTTAAGGTTGAA
TTCTGGCCCTGCTCCCCGATCACCCAGGGTGACAGGGGAGTGGGCTCCAGTGCTGTTATTCTAGATGATAACTTTGTC
ACAAAGGCCACAGCCCTCACCTATGACCCCTATGTAAACTACTCCTCCCGCCATACCATAACCCAGCCCTTCTCCTACCA
CTCCCGCTACTTTACCCCCAAACCTGTCCTAGATTCCACTATTGATTACTTCCAACCAAACAACAAAAGAAATCAGCTGT
GGCTGAGACTACAAACTGCTGGAAATGTAGACCACGTAGGCCTCGGCACTGCGTTCGAAAACAGTATATACGACCAG
GAATACAATATCCGTGTAACCATGTATGTACAATTCAGAGAATTTAATCTTAAAGAGCCCCCAATTAAACCT--------

>DQ478947_pcv2b

ATGACGTATCCAAGGAGGCGTTACCGGAGAAGAAGACACCGCCCCCGCAGCCATCTTGGCCAGATCCTCCGCCGCCG
CCCCTGGCTCGTTCACCCCCGCCACCGTTACCGCTGGAGAAGGAAAAATGGCATCTTCAACACCCGCCTCTCCCGCACC
TTCGGATATACTATCAAGCGAACCACAGTCAAAACGCCCTCCTGGGCGGTGGACATGATGAGATTCAATATTAATGAC
TTTCTTCCCCCAGGAGGGGGCTCAAACCCCCGCTCTGTGCCCTTTGAATACTACAGAATAAGAAAGGTTAAGGTTGAA
TTCTGGCCCTGCTCCCCGATCACCCAGGGTGACAGGGGAGTGGGCTCCAGTGCTGTTATTCTAGATGATAACTTTGTA
ACAAAGGCCACAGCCCTCACCTATGACCCCTATGTAAACTACTCCTCCCGCCATACCATAACCCAGCCCTTCTCCTACCA
CTCCCGCTACTTTACCCCCAAACCTGTCCTAGATTCCACTATTGATTACTTCCAACCAAACAACAAAAGAAATCAGCTGT
GGCTGAGACTACAAACTGCTGGAAATGTAGACCACGTAGGCCTCGGCACTGCGTTCGAAAACAGTATATACGACCAG
GAATACAATATCCGTGTAACCATGTATGTACAACTCAGAGAATTTAATCTTAAAGACCCCCCACT--------------

>AY682997_pcv2b

ATGACGTATCCAAGGAGGCGTTACCGGAGAAGAAGACACCGCCCCCGCAGCCATCTTGGCCAGATCCTCCGCCGCCG
CCCCTGGCTCGTCCACCCCCGCCACCGTTACCGCTGGAGAAGGAAAAATGGCATTTTCAACACCCGCCTCTCCCGCACC
TTCGGATATACTATCAAGCGAACCACAGTCAAAACGCCCTCCTGGGCGGTGGACATGATGAGATTCAATATTAATGAC
TTTCTTCCCCCAGGAGGGGGCTCAAACCCCCGCTCTGTGCCCTTTGAATACTACAGAATAAGAAAGGTTAAGGTTGAA
TTCTGGCCCTGCTCCCCGATCACCCAGGGTGACAGGGGAGTGGGCTCCAGTGCTGTTATTCTAGATGATAACTTTGTA
ACAAAGGCCACAGCCCTCACCTATGACCCGTATGTAAACTACTCCTCCCGCCATACCATAACCCAGCCCTTCTCCTACCA
CTCCCGCTACTTTACCCCCAAACCTGTCCTAGATTCCACTATTGATTACTTCCAACCAAACAACAAAAGAAATCAGCTGT
GGCTGAGACTACAAACTGCTGGAAATGTAGACCACGTAGGCCTCGGCACTGCGTTCGAAAACAGTATATACGACCAG
GAATACAATATCCGTGTAACCATGTATGTACAATTCAGAGAATTTAACCTTAAAGACCCCCACTTAACCCTTAAG-----

>JQ955679_pcv2b

ATGACGTATCCAAGGAGGCGTTACCGGAGAAGAAGACACCGCCCCCGCAGCCATCTTGGCCAGATCCTCCGCCGCCG
CCCCTGGCTCGTCCACCCCCGCCACCGTTACCGCTGGAGAAGGAAAAATGGCATCTTCAACACCCGCCTCTCCCGCACC
TTCGGATATACTATCAAGAGAACCACAGTCAAAACGCCCTCCTGGGCGGTGGACATGATGAGATTCAATATTAATGAC
TTTCTTCCCCCAGGAGGGGGCTCAAACCCCCGCTCTGTGCCCTTTGAATACTACAGAATAAGAAAGGTTAAGGTTGAA
TTCTGGCCCTGCTCCCCGATCACCCAGGGTGACAGGGGAGTGGGCTCCAGTGCTGTTATTCTAGATGATAACTTTGTA
ACAAAGGCCACAGCCCTCACCTACGACCCCTATGTAAACTACTCCTCCCGCCATACCATAACCCAGCCCTTCTCCTACCA
CTCCCGCTACTTTACCCCCAAACCTGTCCTAGATTCCACTATTGATTACTTCCAACCAAACAACAAAAGAAATCAGCTGT
GGCTGAGACTACAAACTGCTGGAAATGTAGACCACGTAGGCCTCGGCACTGCGTTCGAAAACAGTATATACGACCAG
GAATACAATATCCGTGTAACCATGTATGTGCAATTCAGAGAATTTAATCTTAAAACCCCCCACTTAACCCTT--------

>EF371545_pcv2b

ATGACGTATCCAAGGAGGCGTTACCGGAGAAGAAGACACCGCCCCCGCAGCCATCTTGGCCAGATCCTCCGCCGCCG
CCCCTGGCTCGTCCACCCCCGCCACCGTTACCGCTGGAGAAGGAAAAATGGCATCTTCAACACCCGCCTCTCCCGCACC
TTCGGATATACTATCAAGCGAACCACAGTCAAAACGCCCTCCTGGGCGGTGGACATGATGAGATTCAATATTAATGAC
TTTCTTCCCCCAGGAGGGGGCTCAAACCCCCGCTCTGTGCCCTTTGAATACTACAGAATAAGAAAGGTTAAGGTTGAA
TTCTGGCCCTGCTCCCCGATCACCCAGGGTGACAGGGGAGTGGGCTCCAGTGCTGTTATTCTAGATGATAACTTTGTA
ACAAAGGCCACAGCCCTCACCTATGACCCCTATGTAAACTACTCCTCCCGCCATACCATAACCCAGCCCTTCTCCTACCA
CTCCCGCTACTTTACCCCCAAACCTGTCCTAGATTCCACTATTGATTACTTCCAACCAAACAACAAAAGAAATCAGCTGT
GGCTGAGACTACAAACTGCTGGAAATGTAGACCACGTAGGCCTCGGCACTGCGTTCGAAAACAGTATATACGACCAG
GAATACAATATCCGTGTAACCATGTATGTACAATTCAGAGAATTTAATCTTAAGACCCCCCACTTAACCCTT--------

>EF371543_pcv2b

ATGACGTATCCAAGGAGGCGTTACCGGAGAAGAAGACACCGCCCCCGCAGCCATCTTGGCCAGATCCTCCGCCGCCG
CCCCTGGCTCGTCCACCCCCGCCACCGTTACCGCTGGAGAAGGAAAAATGGCATCTTCAACACCCGCCTCTCCCGCACC
TTCGGATATACTATCAAGCGAACCACAGTCAAAACGCCCTCCTGGGCGGTGGACATGATGAGATTCAATATTAATGAC
TTTCTTCCCCCAGGAGGGGGCTCAAACCCCCGCTCTGTGCCCTTTGAATACTACAGAATAAGAAAGGTTAAGGTTGAA
TTCTGGCCCTGCTCCCCGATCACCCAGGGTGACAGGGGAGTGGGCTCCAGTGCTGTTATTCTAGATGATAACTTTGTA
ACAAAGGCCACAGCCCTCACCTATGACCCCTATGTAAACTACTCCTCCCGCCATACCATAACCCAGCCCTTCTCCTACCA
CTCCCGCTACTTTACCCCCAAACCTGTCCTAGATTCCACTATTGATTACTTCCAACCAAACAACAAAAGAAATCAGCTGT
GGCTGAGACTACAAACTGCTGGAAATGTAGACCACGTAGGCCTCGGCACTGCGTTCGAAAACAGTATATACGACCAG
GAATACAATATCCGTGTAACCATGTATGTACAATTCAGAGAATTAATCCTCAAGACCCCCCACTTAACCCTTAAA-----

>EU420015_pcv2b

ATGACGTATCCAAGGAGGCGTTACCGGAGAAGAAGACACCGCCCCCGCAGCCATCTTGGCCAGATCCTCCGCCGCCG
CCCCTGGCTCGTCCACCCCCGCCACCGTTACCGCTGGAGAAGGAAAAATGGCATCTTCAACACCCGCCTCTCCCGCACC
TTCGGATATACTATCAAGCGAACCACAGTCAAAACGCCCTCCTGGGCGGTGGACATGATGAGATTCAATATTAATGAC
TTTCTTCCCCCAGGAGGGGGCTCAAACCCCCGCTCTGTGCCCTTTGAATACTACAGAATAAGAAAGGTTAAGGTTGAA
TTCTGGCCCTGCTCCCCGATCACCCAGGGTGACAGAGGAGTGGGCTCCAGTGCTGTTATTCTAGATGATAACTTTGTA
ACAAAGGCCACAGCCCTCACCTATGACCCCTATGTAAACTACTCCTCCCGCCATACCATAACCCAGCCCTTCTCCTACCA
CTCCCGCTACTTTACCCCCAAACCTGTCCTAGATTCCACTATTGATTACTTCCAACCAAACAACAAAAGAAATCAGCTGT
GGCTGAGACTACAAACTGCTGGAAATGTAGACCACGTAGGCCTCGGCACTGCGTTCGAAAACAGTATATACGACCAA
GAATACAATATCCGTGTAACCATGTATGTACAATTCAGAGAATTTAATCTAAAGACCCCCCACTTAACCCTTAATGAA--

>EU257511_pcv2b

ATGACGTATCCAAGGAGGCGTTACCGGAGAAGAAGACACCGCCCCCGCAGCCATCTTGGCCAGATCCTCCGCCGCCG
CCCCTGGCTCGTCCACCCCCGCCACCGTTACCGCTGGAGAAGGAAAAATGGCATCTTCAACACCCGCCTCTCCCGCACC
TTCGGATATACTATCAAGCGAACCACAGTCAAAACGCCCTCCTGGGCGGTGGACATGATGAGATTCAATATTAATGAC
TTTCTTCCCCCAGGAGGGGGCTCAAACCCCCGCTCTGTGCCCTTTGAATACTACAGAATAAGAAAGGTTAAGGTTGAA
TTCTGGCCCTGCTCCCCGATCACCCAGGGTGACAGGGGAGTGGGCTCCAGTGCTGTTATTCTAGATGATAACTTTGTA
ACAAAGGCCACAGCCCTCACCTATGACCCCTATGTAAACTACTCCTCCCGCCATACCATAACCCAGCCCTTCTCCTACCA
CTCCCGCTACTTTACCCCCAAACCTGTCCTAGATTCCACTATTGATTACTTCCAACCAAACAACAAAAGAAATCAGCTGT
GGCTGAGACTACAAACTGCTGGAAATGTGGACCACGTAGGCCTCGGCATTGCGTTCGAAAACAGTATATACGACCAG
GAATACAATATCCGTGTAACAATGTATGTACAATTCAGAGAATTTAATTTAAAGACCCCCCACTTAACCCTT--------

>EU257516_pcv2b

ATGACGTATCCAAGGAGGCGTTACCGGAGAAGAAGACACCGCCCCCGCAGCCATCTTGGCCAGATCCTCCGCCGCCG
CCCCTGGCTCGTCCACCCCCGCCACCGTTACCGCTGGAGAAGGAAAAATGGCATCTTCAACACCCGCCTCTCCCGCACC
TTCGGATATACTATCAAGCGAACCACAGTCAAAACACCCTCCTGGGCGGTGGACATGATGAGATTCAATATTAATGAC
TTTCTTCCCCCAGGAGGGGGCTCAAACCCCCGCTCTGTGCCCTTTGAATACTACAGAATAAGAAAGGTTAAGGTTGAA
TTCTGGCCCTGCTCCCCGATCACCCAGGGTGACAGGGGAGTGGGCTCCAGTGCTGTTATTCTAGATGATAACTTTGTA
ACAAAGGCCACAGCCCTCACCTATGACCCCTATGTAAACTACTCCTCCCGCCATACCATAACCCAGCCCTTCTCCTACCA
CTCCCGCTACTTTACCCCCAAACCTGTCCTAGATTCCACTATTGATTACTTCCAACCAAACAACAAAAGAAATCAGCTGT
GGCTGAGACTACAAACTGCTGGAAATGTGGACCACGTAGGCCTCGGCATTGCGTTCGAAAACAGTATATACGACCAG
GAATACAATATCCGTGTAACCATGTATGTACAATTCAGAGAATTTAATTTAAAGACCCCCCACTTAACCCTT--------

>HM038032_pcv2b

ATGACGTATCCAAGGAGGCGTTACCGGAGAAGAAGACACCGCCCCCGCAGCCATCTTGGCCAGATCCTCCGCCGCCG
CCCCTGGCTCGTCCACCCCCGCCACCGTTACCGCTGGAGAAGGAAAAATGGCATCTTCAACGCCCGCCTCTCCCGCAC
CTTCGGATATACTATCAAGCGAGCCACAGTCAAAACGCCCTCCTGGGCGGTGGACATGATGAGATTCAATATTAATGA
CTTTCTTCCCCCAGGAGGGGGCTCAAACCCCCGCTCTGTGCCCTTTGAATACTACAGAATAAGAAAGGTTAAGGTTGA
ATTCTGGCCCTGCTCCCCGATCACCCAGGGTGACAGGGGAGTGGGCTCCAGTGCTGCTATTCTAGATGATAACTTTGT
AACAAAGGCCACAGCCCTCACCTATGACCCCTATGTAAACTACTCCTCCCGCCATACCATAACCCAGCCCTTCTCCTACC
ACTCCCGCTACTTTACCCCCAAACCTGTCCTAGATTCCACTATTGATTACTTCCAACCAAACAACAAAAGAAATCAGCTG
TGGCTGAGGCTACAAACTGCTGGAAATGTAGACCACGTAGGCCTCGGCACTGCGTTCGAAAACAGTATATACGACCA
GGAATACAATATCCGTGTAACCATGTATGTACAATTCAGAGAATTTAATCTTAAAGACCCCCCACTTAACCTT--------

>AY596823_pcv2b

ATGACGTATCCAAGGAGGCGTTACCGGAGAAGAAGACACCGCCCCCGCAGCCATCTTGGCCAGATCCTCCGCCGCCG
CCCCTGGCTCGTCCACCCCCGCCACCGTTACCGCTGGAGAAGGAAAAATGGCATCTTCAACGCCCGCCTCTCCCGCAC
CTTCGGATATACTATCAAGCGAACCACTGTCAAAACGCCCTCCTGGGCGGTGGACATGATGAGATTCAATATTAATGA
CTTTCTTCCCCCAGGAGGGGGCTCAAACCCCCGCTCTGTGCCCTTTGAATACTACAGAATAAGAAAGGTTAAGGTTGA
ATTCTGGCCCTGCTCCCCGATCACCCAGGGTGACAGGGGAGTGGGCTCCAGTGCTGTTATTCTAGATGATAACTTTGT
AACAAAGGCCACAGCCCTCACCTATGACCCCTATGTAAACTACTCCTCCCGCCATACCATAACCCAGCCCTTCTCCTACC
ACTCCCGCTACTTTACCCCCAAACCTGTCCTAGATTCCACTATTGATTACTTCCAACCAAACAACAAAAGAAATCAGCTG
TGGCTGAGACTACAAACTGCTGGAAATGTAGACCACGTAGGCCTCGGCACTGCGTTCGAAAACAGTATATACGACCA
GGAATACAATATCCGTGTAACCATGTATGTACAATTCAGAGAATTTAATCTTAAAGACCCCCCACTTAAGCCT--------

>JN006457_pcv2b

ATGACGTATCCAAGGAGGCGTTACCGGAGAAGAAGACACCGCCCCCGCAGCCATCTTGGCCAGATCCTCCGCCGCCG
CCCCTGGCTCGTCCACCCCCGCCACCGTTACCGCTGGAGAAGGAAAAATGGAATCTTCAACACCCGCCTCTCCCGCACC
TTCGGATATACTATCAAGCGAACCACTGTCAAAACGCCCTCCTGGGCGGTGGACATGATGAGATTCAATATTAATGAC
TTTCTTCCCCCAGGAGGGGGCTCAAACCCCCGCTCTGTGCCCTTTGAATACTACAGAATAAGAAAGGTTAAGGTTGAA
TTCTGGCCCTGCTCCCCGATCACCCAGGGTGACAGGGGAGTGGGCTCCAGTGCTGTTATTCTAGATGATAACTTTGTC
ACAAAGGCCACAGCCCTCACCTATGACCCCTATGTAAACTACTCCTCCCGCCATACCATAACCCAGCCCTTCTCCTACCA
CTCCCGCTACTTTACCCCCAAACCTGTCCTAGATTCCACTATTGATTACTTCCAACCAAACAACAAAAGAAATCAGCTGT
GGCTGAGACTACAAACTGCTGGAAATGTAGACCACGTAGGCCTCGGCACTGCGTTCGAAAACAGTATATACGACCAG
GATTACAATATCCGTGTAACCATGTATGTACAATTCAGAGAATTTAATCTTAAAGACCCCCCACTTAACCCT--------

>JN382159_pcv2b

ATGACGTATCCAAGGAGGCGTTACCGGAGAAGAAGACACCGCCCCCGCAGCCATCTTGGCCAGATCCTCCGCCTCCG
CCCCTGGCTCGTCCACCCGGGGCGCCGTTACGGCTGGAGAAGGAAAAAAGGCATGTTCAACACCCGCCTCTCCCGCA
CCTTCGGATATACTATCAAGCGAACCACTGTCAAAACGCCCTCCTGGGCGGTGGACATGATGAGATTCAATATTAATG
ACTTTCTTCCCCCAGGAGGGGGCTCAAACCCCCGCTCTGTGCCCTTTGAATACTACAGAATAAGAAAGGTTAAGGTTG
AATTCTGGCCCTGCTCCCCGATCACCCAGGGTGACAGGGGAGTGGGCTCCAGTGCTGTTATTCTAGATGATAACTTTG
TAACAAAGGCCACAGCCCTCACCTATGACCCCTATGTAAACTACTCCTCCCGCCATACCATAACCCAGCCCTTCTCCTAC
CACTCCCGCTACTTTACCCCCAAACCTGTCCTAGATTCCACTATTGATTACTTCCAACCAAACAACAAAAGAAATCAGCT
GTGGCTGAGACTACAAACTGCTGGAAATGTAGACCACGTAGGCCTCGGCATTGCGTTCGAAAACAGTATATACGACC
AGGAATACAATATCCGTGTAACCATGTATGTACAATTCAGAGAATTTAATCTTAAAGACCCCCCACTTAACCCT--------

>JN382164_pcv2b

ATGACGTATCCAAGGAGGCGTTACCGGAGAAGAAGACACCGCCCCCGCAGCCATCTCGGCCAGATCCTCCGCCGCCG
CCCGTTGCTCGTCCACCCCCGCCGTCGTTTCCGCTGGAGAAGGAAAAATGGCATCTTCAACACCCGCCTCTCCCGCACC
TTAGGATATACTATCAAGGGAACCACTGTCAAAACGCCCTCCTGGGCGGTGGACATGATGAGATTCAATATTAATGAC
TTTCTTCCCCCAGGAGGGGGCTCAAACCCCCGCTTTGTGCCCTTTGAATACTACAGAATAAGAAAGGTTAAGGTTGAA
TTCTGGCCCTGCTCCGCGATCACCCAGGGTGACAGGGGAGTGGGCTCCAGTGCTGTTATTCTAGATGATAACTTTGTA
ACAAAGGCCACAGCCCTCACCTATGACCCCTATGTAAACTACTCCTCCCGCCATACCATAACCCAGCCCTTCTCCTACCA
CTCCCGCTACTTTACCCCCAAACCTGTCCTAGATTCCACTATTGATTACTTCCAACCAAACAACAAAAGAAATCAGCTGT
GGCTGAGACTACAAACTGCTGGAAATGTAGACCACGTAGGCCTCGGCACTGCGTTCGAAAACAGTATATACGACCAG
GAATACAATATCCGTGTAACCATGTATGTACAATTCAGAGAATTTAATCTTAAAGACCCCCCACTTAACCCT--------

>JF927981_pcv2b

ATGACGTATCCAAGGAGGCGTTACCGGAGAAGAAGACACCGCCCCCGCAGCCATCTTGGCCAGATCCTCCGCCGCCG
CCCCTGGCTCGTCCACCCCCGCCACCGTTACCGCTGGAGAAGGAAAAATGGCATCTTCAACACCCGCCTCTCCCGCACA
TTCGGATATACTATCAAGCGAACCACAGTCAAAACGCCCTCCTGGGCGGTGGACATGATGAGATTCAATATTAATGAC
TTTCTTCCCCCAGGAGGGGGCTCAAACCCCCGCTCTGTGCCCTTTGAATACTACAGAATAAGAAAGGTTAAGGTTGAA
TTCTGGCCCTGCTCCCCGATCACCCAGGGTGACAGGGGAGTGGGCTCCAGTGCTGTTATTCTAGATGATAACTTTGTA
ACAAAGGCCACAGCCCTCACCTATGACCCCTATGTAAACTACTCCTCCCGCCATACCATAACCCAGCCCTTCTCCTACCA
CTCCCGCTATTTTACCCCCAAACCTGTCCTAGATTCCACTATTGATTACTTCCAACCAAACAACAAAAGAAATCAGCTGT
GGCTGAGACTACAAACTGCTGGAAATGTAGACCACGTAGGCCTCGGCACTGCGTTCGAAAACAGTATATACGACCAG
GAATACAATATCCGTGTAACCATGTATGTACAATTCAGAGAATTTAATCTTAAAGACCCCCCACTTAACCCT--------

>GU244506_pcv2b

ATGACGTATCCAAGGAGGCGTTACCGGAGAAGAAGACACCGCCCCCGCAGCCATCTTGGCCAGATCCTCCGCCGCCG
CCCCTGGCTCGTCCACCCCCGCCACCGTTACCGCTGGAGAAGGAAAAATGGCTTCTTCCACACCCGCCTCTCCCGCACA
TTCGGATATACTATCAAGCGAACCACAGTCAAAACGCCCTCCTGGGCGGTAGACATGATGAGATTCAATATTAATGAC
TTTCTTCCCCCAGGAGGGGGCTCAAACCCCCGCTCTGTGTCCTTTGAATACTACAGAATAAGAAAGGTTAAGGTTGAA
TTCTGGCCCTGCTCCCCGATCACCCAGGGTGACAGGGGAGTGGGCTCCAGTGCTGTTATTCTAGATGATAACTTTGTA
ACAAAGGCCACAGCCCTCACCTATGACCCCTATGTAAACTACTCCTCCCGCCATACCATAACCCAGCCCTTCTCCTACCA
CTCCCGCTATTTTACCCCCAAACCTGTCCTAGATTCCACTATTGATTACTTCCAACCAAACAACAAAAGAAATCAGCTGT
GGCTGAGACTACAAACTGCTGGAAATGTAGACCACGTAGGCCTCGGCACTGCGTTCGAAAACAGTATATACGACCAG
GAATACAATATCCGTGTAACCATGTATGTACAATTCAGAGAATTTAATCTTAAAGACCCCCCACTTAACCCT--------

>JF927980_pcv2b

ATGACGTATCCAAGGAGGCGTTACCGGAGAAGAAGACACCGCCCCCGCAGCCATCTTGGCCAGATCCTCCGCCGCCG
CCCCTGGCTCGTCCACCCCCGCCACCGTTACCGCTGGAGAAGGAAAAATGGCATCTTCAACACCCGCCTCTCCCGCACA
TTCGGATATACTATCAAGCGAACCACAGTCAAAACGCCCTCCTGGGCGGTGGACATGATGAGATTCAATATTAATGAC
TTTCTTCCCCCAGGAGGGGGCTCAAACCCCCGCTCTGTGCCCTTTGAATACTACAGAATAAGAAAGGTTAAGGTTGAA
TTCTGGCCCTGCTCCCCGATCACCCAGGGTGACAGGGGAGTGGGCTCCAGTGCTGTTATTCTTGATGATAACTTTGTA
ACAGAGGCCACAGCCCTCACCTATGACCCCTATGTAAACTACTCCTCCCGCCATACCATAACCCAGCCCTTCTCCTACCA
CTCCCGCTATTTTACCCCCAAACCTGTCCTAGATTCCACTATTGATTACTTCCAACCAAACAACAAAAGAAATCAGCTGT
GGCTGAGACTACAAACTGCTGGAAATGTAGACCACGTAGGCCTCGGCACTGCGTTCGAAAACAGTATATACGACCAG
GAATACAATATCCGTGTAACCATGTATGTACAATTCAGAGAATTTAATCTTAAAGACCCCCCACTTAACCCT--------

>JF927982_pcv2b

ATGACGTATCCAAGGAGGCGTTACCGCAGAAGAAGACACCGCCCCCGCAGCCATCTTGGCCAGATCCTCCGCCGCCG
CCCCTGGCTCGTCCACCCCCGCCACCGTTACCGCTGGAGAAGGAAAAATGGCATCTTCAACACCCGCCTCTCCCGCACA
TTCGGATATACTATCAAGCGAACCACAGTCAAAACGCCCTCCTGGGCGGTGGACATGATGAGATTCAATATTAATGAC
TTTCTTCCCCCAGGAGGGGGCTCAAACCCCCGCTCTGTGCCCTTTGAATACTACAGAATAAGAAAGGTTAAGGTTGAA
TTCTGGCCCTGCTCCCCGATCACCCAGGGTGACAGGGGAGTGGGCTCCAGTGCTGTTATTCTAGATGATAACTTTGTA
ACAAAGGCGACAGCCCTCACCTACGACCCCTATGTAAACTACTCCTCCCGCCATACCATAACCCAGCCCTTCTCCTACCA
CTCCCGCTATTTTACCCCCAAACCTGTCCTAGATTCCACTATTGATTACTTCCAACCAAACAACAAAAGAAATCAGCTGT
GGCTGAGACTACAAACTGCTGGAAATGTAGACCACGTAGGCCTCGGCACTGCGTTCGAAAACAGTATATACGACCAG
GAATACAATATCCGTGTAACCATGTATGTACAATTCAGAGAATTTAATCTTAAAGACCCCCCACTTAACCCT--------

>JF927976_pcv2b

ATGACGTATCCAAGGAGGCGTTACCGGAGAAGAAGACACCGCCCCCGCAGCCATCTTGGCCAGATCCTCCGCCGCCG
CCCCTGGCTCGTCCACCCCCGCCACCGTTACCGCTGGAGAAGGAAAAATGGCATCTTCAACACCCGCCTCTCCCGCACA
TTCGGATATACTATCAAGCGAACCACAGTCAAAACCCCCTCCTGGGCGGTGGACATGATGAGATTCAATATTAATGAC
TTTCTTCCCCCAGGAGGGGGCTCAAACCCCCGCTCTGTGCCCTTTGAATACTACAGAATAAGAAAGGTTAAGGTTGAA
TTCTGGCCCTGCTCCCCGATCACCCAGGGTGACAGGGGAGTGGGCTCCAGTGCTGTTATTCTAGATGATAACTTTGTA
ACAAAGGCCACAGCCCTCACCTATGACCCCTATGTAAACTACTCCTCCCGCCATACCATAACCCAGCCCTTCTCCTACCA
CTCCCGCTATTTTACCCCCAAACCTGTCCTAGATTCCACTATTGATTACTTCCAACCAAACAGCAAAAGAAATCAGCTGT
GGCTGAGACTACAAACTGCTGGAAATGTAGACCACGTAGGCCTCGGCACTGCGTTCGAAAACAGTATATACGACCAG
GAATACAATATCCGTGTAACCATGTATGTACAATTCAGAGAATTTAATCTTAAAGACCCCCCACTTAACCCT--------

>JF683406_pcv2b

ATGACGTATCCAAGGAGGCGTTACCGGAGAAGAAGACACCGCCCCCGCAGCCATCTTGGCCAGATCCTCCGCCGCCG
CCCCTGGCTCGTCCACCCCCGCCACCGTTACCGCTGGAGAAGGAAAAATGGCATCTTCAACACCCGCCTCTCCCGCACA
TTCGGATATACTATCAAACGAACCACAGTCAAAACGCCCTCCTGGGCGGTGGACATGATGAGATTCAATATTAATGAC
TTTCTTCCCCCAGGAGGGGGCTCAAACCCCCGCTCTGTGCCCTTTGAATACTACAGAATAAGAAAGGTTAAGGTTGAA
TTCTGGCCCTGCTCCCCGATCACCCAGGGTGACAGGGGAGTGGGCTCCAGTGCTGTTATTCTAGATGATAACTTTGTA
ACAAAGGCCACAGCCCTCACCTATGACCCCTATGTAAACTACTCCTCCCGCCATACCATAACCCAGCCCTTCTCCTACCA
CTCCCGCTATTTTACCCCCAAACCTGTCCTAGATTCCACTATTGATTACTTCCAACCAAACAACAAAAGAAATCAGCTAT
GGCTGAGACTACAAACTACTGGAAATGTAGACCACGTAGGCCTCGGCACTGCGTTCGAAAACAGTATATACGACCAG
GAATACAATATCCGTGTAACCATGTATGTACAATTCAGAGAATTTAATCTTAAAGACCCCCCACTTAACCCT--------

>JF927990_pcv2b

ATGACGTATCCAAGGAGGCGTTACCGGAGAAGAAGACACCGCCCCCGCAGCCATCTTGGCCAGATCCTCCGCCGCCG
CCCCTGGCTCGTCCACCCCCGCCACCGTTACCGCTGGAGAAGGAAAAATGGCATCTTCAACACCCGCCTCTCCCGCACA
TTCGGATATACTATCAAACGAACCACAGTCAAAACGCCCTCCTGGGCGGTGGACATGATGAGATTCAATATTAATGAC
TTTCTTCCCCCAGGAGGGGGCTCAAACCCCCGCTCTGTGCCCTTTGAATACTACAGAATAAGAAAGGTTAAGGTTGAA
TTCTGGCCCTGCTCCCCGATCACCCAGGGTGACAGGGGAGTGGGCTCCAGTGCTGTTATTCTAGATGATAACTTTGTA
ACAAAGGCCACAGCCCTCACCTATGACCCCTATGTAAACTACTCCTCCCGCCATACCATAACCCAGCCCTTCTCCTACCA
CTCCCGCTATTTTACCCCCAAACCTGTCCTAGATTCCACTATTGATTACTTCCAACCAAACAACAAAAGAAATCAGCTAT
GGCTGAGACTACAAACTACTGGAAATGTAGACCACGTAGGCCTCGGCACTGCGTTCGAAAACAGTATATACGACCAG
GAATACAATATCCGTGTAACCATGTATGTACAATTCAGAGAATTTAATCTTAAAGACCCCCCACTTAACCCT--------

>JN644771_pcv2b

ATGACGTATCCAAGGAGGCGTTACCGGAGAAGAAGACACCGCCCCCGCAGCCATCTTGGCCAGATCCTCCGCCGCCG
CCCCTGGCTCGTCCACCCCCGCCACCGTTACCGCTGGAGAAGGAAAAATGGCATCTTCAACACCCGCCTCTCCCGCACC
TTCGGATATACTATCAAGCGAACCACAGTCAAAACGCCCTCCTGGGCGGTGGACATGATGAGATTCAATATTAATGAC
TTTCTTCCCCCAGGAGGGGGCTCAAACCCCCGCTCTGTGCCCTTTGAATACTACAGAATAAGAAAGGTTAAGGTTGAA
TTCTGGCCCTGCTCCCCGATCACCCAGGGTGACAGGGGAGTGGGCTCCAGTGCTGTTATTCTAGATGATAACTTTGTA
ACAAAGGCCACAGCCCTCACCTATGACCCCTATGTAAACTACTCCTCCCGCCATACCATAACCCAGCCCTTCTCCTACCA
CTCCCGGTATTTTACCCCCAAACCTGTCCTAGATTCCACTATTGATTACTTCCAACCAAACAACAAAAGAAATCAGCTGT
GGCTGAGACTACAAACTACTGGAAATGTAGACCACGTAGGCCTCGGCACTGCGTTCGAAAACAGTATATACGACCAG
GAATACAATATCCGTGTAACCATGTATGTACAATTCAGAGAATTTAATCTTAAAGACCCCCCACTTAACCCT--------

>JN644770_pcv2b

ATGACGTATCCAAGGAGGCGTTACCGGAGAAGAAGACACCGCCCCCGCAGCCATCTTGGCCAGATCCTCCGCCGCCG
CCCCTGGCTCGTCCACCCCCGCCACCGTTACCGCTGGAGAAGGAAAAATGGCATCTTCAACACCCGCCTCTCCCGCACC
TTCGGATATACTATCAAGCGAACCACAGTCAAAACGCCCTCCTGGGCGGTGGACATGATGAGATTCAATATTAATGAC
TTTCTTCCCCCAGGAGGGGGCTCAAACCCCCGCTCTGTGCCCTTTGAATACTACAGAATAAGAAAGGTTAAGGTTGAA
TTCTGGCCCTGCTCCCCGATCACCCAGGGTGACAGGGGAGTGGGCTCCAGTGCTGTTATTCTAGATGATAACTTTGTA
ACAAAGGCCACAGCCCTCACCTATGACCCCTATGTAAACTACTCCTCCCGCCATACCATAACCCAGCCCTTCTCCTACCA
CTCCCGGTATTTTACCCCCAAACCTGTCCTAGATTCCACTATTGATTACTTCCAACCAAACAACAAAAGAAATCAGCTGT
GGCTGAGACTACAAACTACTGGAAATGTAGACCACGTAGGCCTCGGCACTGCGTTCGAAAACAGTATATACGACCAG
GAATACAATATCCGTGTAACCATGTATGTACAATTCAGAGAATTTAATCTTAAAGACCCCCCACTTAACCCT--------

>JN644769_pcv2b

ATGACGTATCCAAGGAGGCGTTACCGGAGAAGAAGACACCGCCCCCGCAGCCATCTTGGCCAGATCCTCCGCCGCCG
CCCCTGGCTCGTCCACCCCCGCCACCGTTACCGCTGGAGAAGGAAAAATGGCATCTTCAACACCCGCCTCTCCCGCACC
TTCGGATATACTATCAAGCGAACCACAGTCAAAACGCCCTCCTGGGCGGTGGACATGATGAGATTCAATATTAATGAC
TTTCTTCCCCCAGGAGGGGGCTCAAACCCCCGCTCTGTGCCCTTTGAATACTACAGAATAAGAAAGGTTAAGGTTGAA
TTCTGGCCCTGCTCCCCGATCACCCAGGGTGACAGGGGAGTGGGCTCCAGTGCTGTTATTCTAGATGATAACTTTGTA
ACAAAGGCCACAGCCCTCACCTATGACCCCTATGTAAACTACTCCTCCCGCCATACCATAACCCAGCCCTTCTCCTACCA
CTCCCGGTATTTTACCCCCAAACCTGTCCTAGATTCCACTATTGATTACTTCCAACCAAACAACAAAAGAAATCAGCTGT
GGCTGAGACTACAAACTACTGGAAATGTAGACCACGTAGGCCTCGGCACTGCGTTCGAAAACAGTATATACGACCAG
GAATACAATATCCGTGTAACCATGTATGTACAATTCAGAGAATTTAATCTTAAAGACCCCCCACTTAACCCT--------

>EF371546_pcv2b

ATGACGTATCCAAGGAGGCGTTACCGGAGAAGAAGACACCGCCCCCGCAGCCATCTTGGCCAGATCCTCCGCCGCCG
CCCCTGGCTCGTCCACCCCCGCCACCGTTACCGCTGGAGAAGGAAAAATGGCATCTTCAACACCCGCCTCTCCCGCACC
TTCGGATATACTATCAAGCGAACCACAGTCAAAACGCCCTCCTGGGCGGTGGACATGATGAGATTCAATATTAATGAC
TTTCTTCCCCCAGGAGGGGGCTCAAACCCCCGCTCTGTGCCCTTTGAATACTACAGAATAAGAAAGGTTAAGGTTGAA
TTCTGGCCCTGCTCCCCGATCACCCAGGGTGACAGGGGAGTGGGCTCCAGTGCTGTTATTCTAGATGATAACTTTGTA
ACAAAGGCCACAGCCCTCACCTATGACCCCTATGTAAACTACTCCTCCCGCCATACCATAACCCAGCCCTTCTCCTACCA
CTCCCGCTATTTTACCCCCAAACCTGTCCTAGATTCCACTATTGATTACTTCCAACCAAACAACAAAAGAAATCAGCTGT
GGCTGAGACTACAAACTGCTGGAAATGTAGACCACGTAGGCCTCGGCACTGCGTTCGAAAACAGTATATACGACCAG
GAATACAATATCCGTGTAACTATGTATGTACAATTCAGAGAATTTAATCTTAAAGACCCCCCACTTAACCCT--------

>HQ831537_pcv2b

ATGACGTATCCAAGGAGGCGTTACCGGAGAAGAAGACACCGCCCCCGCAGCCATCTTGGCCAGATCCTCCGCCGCCG
CCCCTGGCTCGTCCACCCCCGCCACCGTTACCGCTGGAGAAGGAAAAATGGCATCTTCAACACCCGCCTCTCCCGCACC
TTCGGATATACTATCAAGCGAACCACAGTCAAAACGCCCTCCTGGGCGGTGGACATGATGAGATTCAATATTAATGAC
TTTCTTCCCCCAGGAGGGGGCTCAAACCCCCGCTCTGTGCCCTTTGAATACTACAGAATAAGAAAGGTTAAGGTTGAA
TTCTGGCCCTGCTCCCCGATCACCCAGGGTGACAGGGGAGTGGGCTCCAGTGCTGTTATTCTAGATGATAACTTTGTA
ACAAAGGCCACAGCCCTCACCTATGACCCCTATGTAAACTACTCCTCCCGCCATACCATAACCCAGCCCTTCTCCTACCA
CTCCCGCTACTTTACCCCCAAACCTGTCCTAGATTCCACTATTGATTACTTCCAACCAAACAACAAAAGAAATCAGCTGT
GGCTGAGACTACAAACTACTGGAAATGTAGACCACGTAGGCCTCGGCACTGCGTTCGAAAACAGTATATACGACCAG
GACTACAATATCCGTGTAACCATGTATGTACAATTCAGAGAATTTAATCTTAAAGACCCCCCACTTAACCCT--------

>JN006464_pcv2b

ATGACGTATCCAAGGAGGCGTTACCGGAGAAGAAGACACCGCCCCCGCAGCCATCTTGGCCAGATCCTCCGCCGCCG
CCCCTGGCTCGTCCACCCCCGCCACCGTTACCGCTGGAGAAGGAAAAATGGCATCTTCAACACCCGCCTCTCCCGCACC
TTCGGATATACTATCAAGCGAACCACAGTCAAAACGCCCTCCTGGGCGGTGGACATGATGAGATTCAATATTAATGAC
TTTCTTCCCCCAGGAGGGGGCTCAAACCCCCGCTCTGTGCCCTTTGAATACTACAGAATAAGAAAGGTTAAGGTTGAA
TTCTGGCCCTGCTCCCCGATCACCCAGGGTGACAGGGGAGTGGGCTCCAGTGCTGTTATTCTAGATGATAACTTTGTA
ACAAAGGCCACAGCCCTCACCTATGACCCCTATGTAAACTACTCCTCCCGCCATACCATAACCCAGCCCTTCTCCTACCA
CTCCCGCTACTTTACCCCCAAACCTGTCCTAGATTCCACTATTGATTACTTCCAACCAAACAACAAAAGAAATCAACTGT
GGCTGAGACTACAAACTACTGGAAATGTAGACCACGTAGGCCTCGGCACTGCGTTCGAAAACAGTATATACGACCAG
GACTACAATATCCGTGTAACCATGTATGTACAATTCAGAGAATTTAATCTTAAAGACCCCCCACTTAACCCT--------

>EU521708_pcv2b

ATGACGTATCCAAGGAGGCGTTACCGGAGAAGAAGACACCGCCCCCGCAGCCATCTTGGCCAGATCCTCCGCCGCCG
CCCCTGGCTCGTCCACCCCCGCCACCGTTACCGCTGGAGAAGGAAAAATGGCATCTTCAACACCCGCCTCTCCCGCACC
TTCGGATATACTATCAAGCGAACCACAGTCAAAACGCCCTCCTGGGCGGTGGACATGATGAGATTCAATATTAACGAC
TTTCTTCCCTCAGGAGGGGGCTCAAACCCCCGCTCTGTGCCCTTTGAATACTACAGAATAAGAAAGGTTAAGGTTGAA
TTCTGGCCCTGCTCCCCGATCACCCAGGGTGACAGGGGAGTGGGCTCCAGTGCTGTTATTCTAGATGATAACTTTGTA
ACAAAGGCCACAGCCCTCACCTATGACCCCTATGTAAACTACTCCTCCCGCCATACCATAACCCAGCCCTTCTCCTACCA
CTCCCGCTACTTTACCCCCAAACCTGTCCTAGATTCCACTATTGATTACTTCCAACCAAACAACAAAAGAAATCAGCTGT
GGCTGAGGCTACAAACTACTGGAAATGTAGACCACGTAGGCCTCGGCACTGCGTTCGAAAACAGTATATACGACCAG
GAATACAATATCCGTGTAACCATGTATGTACAATTCAGAGAATTTAATCTTAAAGACCCCCCACTTAACCCT--------

>HQ395019_pcv2b

ATGACGTACCCAAGGAGGCGTTACCGGAGAAGAAGACACCGCCCCCGCAGCCATCTTGGCCAGATCCTCCGCCGCCG
CCCCTGGCTCCTCCACCCCCGCCACCGTTACCGCTGGAGAAGGAAAAATGGCATCTTCAACACCCGCCTCTCCCGCACC
TTCGGATATACTATCAAGCGAACCACAGTCAAAACGCCCTCCTGGGCGGTGGACATGATGAGATTCAATATTAATGAC
TTTCTTCCCCCAGGAGGGGGCTCAAACCCCCGCTCTGTGCCCTTTGAATACTACAGAATAAGAAAGGTTAAGGTTGAA
TTCTGGCCCTGCTCCCCGATCACCCAGGGTGACAGGGGAGTGGGCTCCAGTGCTGTTATTCTAGATGATAACTTTGTA
GCAAAGGCCACAGCCCTCACCTATGACCCCTATGTAAACTACTCCTCCCGCCATACCATAACCCAGCCCTTCTCCTACCA
CTCCCGCTACTTTACCCCCAAACCTGTCCTAGATTCCACTATTGATTACTTCCAACCAAACAACAAAAGAAATCAGCTGT
GGCTGAGACTACAAACTACTGGAAATGTAGACCACGTAGGCCTCGGCACTGCGTTCGAAAACAGTATATACGACCAG
GAATACAATATCCGTGTAACCATGTATGTACAATTCAGAGAATTTAATCTTAAAGACCCCCCACTTAACCCT--------

>HQ395020_pcv2b

ATGACGTACCCAAGGAGGCGTTACCGGAGAAGAAGACACCGCCCCCGCAGCCATCTTGGCCAGATCCTCCGCCGCCG
CCCCTGGCTCCTCCACCCCCGCCACCGTTACCGCTGGAGAAGGAAAAATGGCATCTTCAACACCCGCCTCTCCCGCACC
TTCGGATATACTATCAAGCGAACCACAGTCAAAACGCCCTCCTGGGCGGTGGACATGATGAGATTCAATATTAATGAC
TTTCTTCCCCCAGGAGGGGGCTCAAACCCCCGCTCTGTGCCCTTTGAATACTACAGAATAAGAAAGGTTAAGGTTGAA
TTCTGGCCCTGCTCCCCGATCACCCAGGGTGACAGGGGAGTGGGCTCCAGTGCTGTTATTCTGGATGATAACTTTGTA
ACAAAGGCCACAGCCCTCACCTATGACCCCTATGTAAACTACTCCTCCCGCCATACCATAACCCAGCCCTTCTCCTACCA
CTCCCGCTACTTTACCCCCAAACCTGTCCTAGATTCCACTATTGATTACTTCCAACCAAACAACAAAAGAAATCAGCTGT
GGCTGAGACTACAAACTACTGGAAATGTAGACCACGTAGGCCTCGGCACTGCGTTCGAAAACAGTATATACGACCAG
GAATACAATATCCGTGTAACCATGTATGTACAATTCAGAGAATTTAATCTTAAAGACCCCCCACTTAACCCT--------

>EU296794_pcv2b

ATGACGTATCCAAGGAGGCGTTACCGGAGAAGAAGACACCGCCCCCGCAGCCATCTTGGCCAGATCCTCCGCCGCCG
CCCCTGGCTCCTCCACCCCCGCCACCGTTACCGCTGGAGAAGGAAAAATGGCATCTTCAACACCCGCCTCTCCCGCACC
TTCGGATATACTATCAAGCGAACCACAGTCAAAACGCCCTCCTGGGCGGTGGACATGATGAGATTCAATATTAATGAC
TTTCTTCCCCCAGGAGGGGGCTCAAACCCCCGCTCTGTGCCCTTTGAATACTACAGAATAAGAAAGGTTAAGGTTGAA
TTCTGGCCCTGCTCCCCGATCACCCAGGGTGACAGGGGAGTGGGCTCCAGTGCTGTTATTCTAGATGATAACTTTGTA
ACAAAGGCCACAGCCCTCACCTATGACCCCTATGTAAACTACTCCTCCCGCCATACCATAACCCAGCCCTTCTCCTATCA
CTCCCGCTACTTTACCCCCAAACCTGTCCTAGATTCCACTATTGATTACTTCCAACCAAACAACAAAAGAAGTCAGCTGT
GGCTGAGACTACAAACTACTGGAAATGTAGACCACGTAGGCCTCGGCACTGCGTTCGAAAACAGTATATACGACCAG
GAATACAATATCCGTGTAACCATGTATGTACAATTCAGAGAATTTAATCTTAAAGACCCCCCACTTAACCCA--------

>JX678978_pcv2b

ATGACGTATCCAAGGAGGCGTTACCGGAGAAGAAGACACCGCCCCCGCAGCCATCTTGGCCAGATCCTCCGCCGCCG
CCCCTGGCTCCTCCACCCCCGCCACCGCTACCGCTGGAGAAGGAAAAATGGCATCTTCAACACCCGCCTCTCCCGCACC
TTCGGATATACTATCAAGCGAACCACAGTCAAAACGCCCTCCTGGGCGGTGGACATGATGAGATTCAATATTAATGAC
TTTCTTCCCCCAGGAGGGGGCTCAAACCCCCGCTCTGTGCCCTTTGAATACTACAGAATAAGAAAGGTTAAGGTTGAA
TTCTGGCCCTGCTCCCCCATCACCCAGGGTGACAGGGGAGTGGGCTCCAGTGCTGTTATTCTAGATGATAACTTTGTA
ACAAAGGCCACAGCCCTCACCTATGACCCCTATGTAAACTACTCCTCCCGCCATACCATAACCCAGCCCTTCTCCTACCA
CTCCCGCTACTTTACCCCCAAACCTGTCCTAGATTCCACTATTGATTACTTCCAACCAAACAACAAAAGAAATCAGCTGT
GGCTGAGACTACAAACTACTGGAAATGTAGACCACGTAGGCCTCGGCACTGCGTTCGAAAACAGTATATACGACCAG
GAATACAATATCCGTGTAACCATGTATGTACAATTCAGAGAATTTAATCTTAAAGACCCCCCACTTAACCCT--------

>FJ870976_pcv2b

ATGACGTATCCAAGGAGGCGTTACCGGAGAAGAAGACACCGCCCCCGCAGCCATCTTGGCCAGATCCTCCGCCGCCG
CCCCTGGCTCGTCCACCCCCGCCACCGCTACCGCTGGAGAAGGAAAAATGGCATCTTCAACACCCGCCTCTCCCGCAC
CTTCGGATATACTATCAAGCGAACCACAGTCAAAACGCCCTCCTGGGCGGTTGACATGATGAGATTCAATATTAATGA
CTTTCTTCCCCCAGGAGGGGGCTCAAACCCCCGCTCTGTGCCCTTTGAATACTACAGAATAAGAAAGGTTAAGGTTGA
ATTCTGGCCCTGCTCCCCGATCACCCAGGGTGACAGGGGAGTGGGCTCCAGTGCTGTTATTTTGGATGATAACTTTGT
AACAAAGGCCACAGCCCTCACCTATGACCCCTATGTAAACTACTCCTCCCGCCATACCATAACCCAGCCCTTCTCCTACC
ACTCCCGCTACTTTACCCCCAAACCTGTCCTAGATTCCACTATTGATTACTTCCAACCAAACAACAAAAGAAATCAGCTG
TGGCTGAGACTACAAACTGCTGGAAATGTAGACCACGTAGGCCTCGGCACTGCGTTCGAAAACAGTATATACGACCA
GGAATACAATATCCGTGTAACCATGTATGTACAATTCAGAGAATTTAATCTTAAAGACCCCCCACTTAACCCT--------

>JX945577_pcv2b

ATGACGTATCCAAGGAGGCGTTACCGGAGAAGAAGACAACGCCCCCGCAGCCATCTTGGCCAGATCCTCCGCCGCCG
CCCCTGGCTCGTCCACCCCCGCCACCGCTACCGCTGGAGAAGGAAAAATGGCATCTTCAACACCCGCCTCTCCCGCAC
CTTCGGATATACTATCAAGCGAACCACAGTCAAGACGCCCTCCTGGGCGGTGAACATGATGAGATTCAATATTAATGA
CTTTCTTCCCCCAGGAGGGGGCTCAAACCCCCGCTCTGTGCCCTTTGAATACTACAGAATAAGAAAGGTTAAGGTTGA
ATTCTGGCCCTGCTCCCCGATCACCCAGGGTGACAGGGGAGTGGGCTCCAGTGCTGTTATTCTGGATGATAACTTTGT
AACAAAGGCCACAGCCCTCACCTATGACCCCTATGTAAACTACTCCTCCCGCCATACCATAACCCAGCCCTTCTCCTACC
ACTCCCGCTACTTTACCCCCAAACCTGTCCTAGATTCCACTATTGATTACTTCCAACCAAACAACAAAAGAAATCAGCTG
TGGCTGAGACTACAAACTACTGGAAATGTGGACCACGTAGGCCTCGGAACTGCGTTCGAAAACAGTATATACGACCA
GGAATACAATATCCGTGTAACCATGTATGTACAATTCAGAGAATTTAATCTTAAAGACCCCCCACTTAACCCT--------

>JX945576_pcv2b

ATGACGTATCAAAGGAGGCGTTACCGGAGAAGAAGACACCGCCCCCGCAGCCATCTTGGCCAAATCCTCCGCCGCCG
CCCCTGGCTCGTCCACCCCCGCCACCGCTACCGCTGGAGAAGGAAAAATGGCATCTTCAACGCCCGCCTCTCCCGCAC
CTTCGGATATACTATCAAGCGAACCACAGTCAAAACGCCCTCCTGGGCGGTGGACATGATGAGATTCAATATTAATGA
CTTTCTTCCCCCAGGAGGGGGCTCAAACCCCCGCTCTGTGCCCTTTGAATACTACAGAATAAGAAAGGTTAAGGTTGA
ATTCTGGCCCTGCTCCCCAATCACCCAGGGTGACAGGGGAGTGGGCTCCAGTGCTGTTATTCTGGATGATAACTTTGT
AACAAAGGCCACAGCCCTCACCTATGACCCCTATGTAAACTACTCCTCCCGCCATACCATAACCCAGCCCTTCTCCTACC
ACTCCCGCTACTTTACGCCCAAACCTGTCCTAGATTCCACTATTGATTACTTCCAACCAAACAACAAAAGAAATCAGCTG
TGGCTGAGACTACAAACTACTGGAAATGTAGACCACGTAGGCCTCGGCACTGCGCTCGAAAACAGTATATACGACCA
GGAATACAATATCCGTGTAACCATGTATGTACAATTCAGAGAATTTAATCTTAAAGACCCCCCACTTAACCCT--------

>JQ181585_pcv2b

ATGACGTATCCAAGGAGGCGTTACCGGAGAAGAAGACACCGCCCCCGCAGCCATCTTGGCCAGATCCTCCGCCGCCG
CCCCTGGCTCGTCCACCCCCGCCACCGCTACCGCTGGAGAAGGAAAAATGGCATCTTCAACACCCGCCTCTCCCGCAC
CTTCGGATATACTATCAAGCGAACCACAGTCAAAACGCCCTCCTGGGCGGTGGACATGATGAGATTCAATATTAATGA
CTTTCTTCCCCCAGGAGGGGGCTCAAACCCCCGCTCTGTACCCTTTGAATACTACAGAATAAGAAAGGTTAAGGTTGA
ATTCTGGCCCTGCTCCCCGATCACCCAGGGTGACAGGGGAGTGGGCTCCAGTGCTGTTATTCTGGATGATAACTTTGT
AACAAAGGCCACAGCCCTCACCTATGACCCCTATGTAAACTACTCCTCCCGCCATACCATAACCCAGCCCTTCTCCTACC
ACTCCCGCTACTTTACCCCCAAACCTGTCCTAGATTCCACTATTGATTACTTCCAACCAAACAACAAAAGAAATCAGCTG
TGGCTGAGACTACAAACTACTGGAAATGTAGACCACGTAGGCCTCGGCACTGCGTTCGAAAACAGTATATACGACCA
GGAATACAATATCCGTGTAACCATGTATGTACAATTCAGAGAATTTAATCTTAAAGACCCCCCACTTAACCCT--------

>JQ181589_pcv2b

ATGACGTATCCAAGGAGGCGTTACCGGAGAAGAAGACACCGCCCCCGCAGCCATCTTGGCCAGATCCTCCGCCGCCG
CCCCTGGCTCGTCCACCCCCGCCACCGCTACCGCTGGAGAAGGAAAAATGGCATCTTCAACACCCGCCTCTCCCGCAC
CTTCGGATATACTATCAAGCGAACCACAGTCAAAACGCCCTCCTGGGCGGTGGACATGATGAGATTCAATATTAATGA
CTTTCTTCCCCCAGGAGGGGGCTCAAACCCCCGCTCTGTACCCTTTGAATACTACAGAATAAGAAAGGTTAAGGTTGA
ATTCTGGCCCTGCTCCCCGATCACCCAGGGTGACAGGGGAGTGGGCTCCAGTGCTGTTATTCTGGATGATAACTTTGT
AACAAAGGCCACAGCCCTCACCTATGACCCCTATGTAAACTACTCCTCCCGCCATACCATAACCCAGCCCTTCTCCTACC
ACTCCCGCTACTTTACCCCCAAACCTGTCCTAGATTCCACTATTGATTACTTCCAACCAAACAACAAAAGAAATCAGCTG
TGGCTGAGACTACAAACTACTGGAAATGTAGACCACGTAGGCCTCGGCACTGCGTTCGAAAACAGTATATACGACCA
GGAATACAATATCCGTGTAACCATGTATGTACAATTCAGAGAATTTAATCTTAAAGACCCCCCACTTAACCCT--------

>DQ648031_pcv2b

ATGACGTATCCAAGGAGGCGTTACCGGAGAAGAAGACACCGCCCCCGCAGCCATCTTGGCCAGATCCTCCGCCGCCG
CCCCTGGCTCGTTCACCCCCGCCACCGTTACCGCTGGAGAAGGAAAAATGGCATCTTCAACACCCGCCTCTCCCGCACC
TTCGGATATACTATCAAGCGAACCACAGTCAAAACGCCCTCCTGGGCGGTGGACATGATGAGATTCAATATTAATGAC
TTTCTTCCCCCAGGAGGGGGCTCAAACCCCCGCTCTGTGCCCTTTGAATACTACAGAATAAGAAAGGTTAAGGTTGAA
TTCTGGCCCTGCTCCCCGATCACCCAGGGTGACAGGGGAGTGGGCTCCAGTGCTGTTATTCTAGATGATAACTTTGTA
ACAAAGGCCACAGCCCTCACCTATGACCCCTATGTAAACTACTCCTCCCGCCATACCATAACCCAGCCCTTCTCCTACCA
CTCCCGCTACTTTACCCCCAAACCTGTCCTAGATTCCACTATTGATTACTTCCAACCAAACAACAAAAGAAATCAGCTGT
GGCTGAGACTACAAACTGCTGGAAATGTAGACCACGTAGGCCTCGGCACTGCGTTCGAAAACAGTATATACGACCAG
GAATACAATATCCGTGTAACCATGTATGTACAATTCAGAGAATTTAATCTTAAAGACCCCCCACTTAACCCT--------

>JF690916_pcv2b

ATGACGTATCCAAGGAGGCGTTACCGGAGAAGAAGACACCGCCCCCGCAGCCATCTTGGCCAGATCCTCCGCCGCCG
CCCCTGGCTCGTCCACCCCCGCCACCCTTACCGCTGGAGAAGGAAAAATGGCATCTTCAACACCCGCCTCTCCCGCACC
TTCGGATATACTATCAAGCGAACCACAGTCAAAACGCCCTCCTGGGCGGTGGACATGATGAGATTCAATATTAATGAC
TTTCTTCCCCCAGGAGGGGGCTCAAACCCCCGCTCTGTGCCCTTTGAATACTACAGAATAAGAAAGGTTAAGGTTGAA
TTCTGGCCCTGCTCCCCGATCACCCAGGGTGACAGGGGAGTGGGCTCCAGTGCTGTTATTCTAGATGATAACTTTGTA
ACAAAGGCCACAGCCCTCACCTATGCCCCCTATGTAAACTACTCCTCCCGCCATACCATACCCCAGCCCTTCTCCTACCA
CTCCCGCTACTTTACCCCCAAACCTGTCCTAGATTCCACTATAGATTACTTCCAACCAAACAACAAAAGAAATCAGCTGT
GGCTGAGACTACAAACTGCTGGAAATGTAGACCACGTAGGCCTCGGCACTGCGTTCGAAAACAGTATATACGACCAG
GAATACAATATCCGTGTAACCATGTATGTACAATTCAGAGAATTTAATCTTAAAGACCCCCCACTTAACCCT--------

>JF690917_pcv2b

ATGACGTATCCAAGGAGGCGTTACCGGAGAAGAAGACACCGCCCCCGCAGCCATCTTGGCCAGATCCTCCGCCGCCG
CCCCTGGCTCGTCCACCCGCCCCACCGTTACCGCTGGAGAAGGAAAAATGGCATCTTCAACACCCGCCTCTCCCGCACC
TTCGGATATACTATCAAGCGAACCACAGTCAAAACGCCCTCCTGGGCGGTGGACATGATGAGATTCAATATTAATGAC
TTTCTTCCCCCAGGAGGGGGCTCAAACCCCCGCTCTGTGCCCTTTGAATACTACAGAATAAGAAAGGTTAAGGTTGAA
TTCTGGCCCTGCTCCCCGATCACCCAGGGTGACAGGGGAGTGGGCTCCAGTGCTGTTATTCTAGATGATAACTTTGTA
ACAAAGGCCACAGCCCTCACCTATGGCCCCTATGTAAACTACTCCTCCCGCCATACCATACCCCAGCCCTTCTCCTACCA
CTCCCGCTACTTTACCCCCAAACCTGTCCTAGATTCCACTATTGATTACTTCCAACCAAACAACAAAAGAAATCAGCTGT
GGCTGAGACTACAAACTGCTGGAAATGTAGACCACGTAGGCCTCGGCACTGCGTTCGAAAACAGTATATACGACCAG
GAATACAATATCCGTGTAACCATGTATGTACAATTCAGAGAATTTAATCTTAAAGACCCCCCACTTAACCCT--------

>HQ395039_pcv2b

ATGACGTATCCAAGGAGGCGTTACCGGAGAAGAAGACACCGCCCCCGCAGCCATCTTGGCCAGATCCTCCGCCGCCG
CCCCTGGCTCGTCCACCCCCGCCACCGTTACCGCTGGAGAAGGAAAAATGGCATCTTCAACACCCGCCTCTCCCGCACC
TTCGGATATACTATCAAGCGAACCACAGTCAAAACGCCCTCCTGGGCGGTGGACATGATGAGATTCAATATTAATGAC
TTTCTTCCCCCAGGAGGGGGCTCAAACCCCCGCTCTGTGCCCTTTGAATACTACAGAATAAGAAAGGTTAAGGTTGAA
TTCTGGCCCTGCTCCCCGATCACCCAGGGTGACAGGGGAGTGGGCTCCAGTGCTGTTATTCTAGATGATAACTTTGTA
ACAAAGGCCACAGCCCTCACCTATGACCCCTATGTAAACTACTCCTCCCGCCATACCATACCCCAGCCCTTCTCCTACCA
CTCCCGCTACTTTACCCCCAAACCTGTCCTAGATTCCACTATTGATTACTTCCAACCAAACAACAAAAGAAATCAGCTGT
GGCTGAGACTACAAACTGCTGGAAATGTAGACCACGTAGGCCTCGGCACTGCGTTCGAAAACAGTATATACGACCAG
GAATACAATATCCGTGTAACCATGTATGTACAATTCAGAGAATTTAATCTTAAAGACCCCCCACTTAAACCT--------

>FJ644556_pcv2b

ATGACGTATCCAAGGAGGCGTTACCGGAGAAGAAGACACCGCCCCCGCAGCCATCTTGGCCAGATCCTCCGCCGCCG
CCCCTGGCTCGTCCACCCCCGCCACCGTTACCGCTGGAGAAGGAAAAATGGCATCTTCAACACCCGCCTCTCCCGCACC
TTCGGATATACTATCAAGCGAACCACAGTCAAAACGCCCTCCTGGGCGGTGGACATGATGAGATTCAATATTAATGAC
TTTCTTCCCCCAGGAGGGGGGACAAACAAAAGCTCTGTGCCCTTTGAATACTACAGAATAAGAAAGGTTAAGGTTGAA
TTCTGGCCCTGCTCCCCGATCACCCAAGGTGACAGGGGAGTGGGCTCCAGTGCTGTTATTCTAGATGATAACTTTGTA
ACAAAGGCCACAGCCCTCACCTATGACCCCTATGTAAACTACTCCTCCCGCCATACCATCCCCCAGCCCTTCTCCTACCA
CTCCCGCTACTTTACCCCCAAACCTGTCCTAGATTCCACTATTGATTACTTCCAACCAAACAACAAAAGAAATCAGCTGT
GGCTGAGACTACAAACTGCTGGAAATGTAGACCACGTAGGCCTCGGCACTGCGTTCGAAAACAGTATATACGACCAG
GAATACAATATCCGTGTAACCATGTATGTACAATTCAGAGAATTTAATCTTAAAGACCCCCCACTTAACCCT--------

>DQ915584_pcv2b

ATGACGTATCCAAGGAGGCGTTACCGGAGAAGAAGACACCGCCCCCGCAGCCATCTTGGCCAGATCCTCCGCCGCCG
CCCCTGGCTCGTCCACCCCCGCCACCGTTACCGCTGGAGAAGGAAAAATGGCATCTTCAACACCCGCCTCTCCCGCACC
TTCGGATATACTATCAAGCGAACCACAGTCAAAACGCCCTCCTGGGCGGTGGACATGATGAGATTTAATCTTGACGAC
TTTGTTCCCCCGGGAGGGGGCTCAAACCCCCGCTCTGTGCCCTTTGAATACTACAGAATAAGAAAGGTTAAGGTGGAA
TTCTGGCCCTGCTCCCCGATTACCCAGGGTGACAGGGGAGTGGGCTCCAGTGCTGTTATTCTAGATGATAACTTTGTA
ACAAAGGCCACAGCCCTCACCTATGACCCCTATGTAAACTACTCCTCCCGCCATACCATCCCCCAGCCCTTCTCCTACCA
CTCCCGCTACTTCACCCCCAAACCTGTCCTAGATTCCACTATTGATTACTTCCAACCAAATAACAAAAGAAATCAGCTGT
GGCTGAGACTACAAACTGCTGGAAATGTAGACCACGTAGGCCTCGGCACTGCGTTCGAAAACAGTAAATACGACCAG
GACTACAATATCCGTGTAACCATGTATGTACAATTCAGAGAATTTAATCTTAAAGACCCCCCACTTAACCCT--------

>AY256457_pcv2b

ATGACGTATCCAAGGAGGCGTTACCGGAGAAGAAGACACCGCCCCCGCAGCCATCTTGGCCAGATCCTCCGCCGCCG
CCCCTGGCTCGTCCACCCCCGCCACCGTTACCGCTGGAGAAGGAAAAATGGCATCTTCAACACCCGCCTCTCCCGCACC
TTCGGATATACTATCAAGCGAACCACAGTCAAAACGCCCTCCTGGGCGGTGGACATGATGAGATTCAATATTAATGAC
TTTCTTCCCCCAGGAGGGGGCTCAAACCCCCGCTCTGTGCCCTTTGAATACTACAGAATAAGAAAGGTTAAGGTTGAA
TTCTGGCCCTGCTCCCCGATCACCCAAGGTGACAGGGGAGTGGGCTCCAGTGCTGTTATTCTAGATGATAACTTTGTA
ACAAAGGCCACAGCCCTCACCTATGACCCCTATGTAAACTACTCCTCCCGCCATACCATAACCCAGCCCTTCTCCTACCA
CTCCCGCTACTTTACCCCCAAACCTGTCCTAGATTCCACTATTGATTACTTCCAACCAAACAACAAAAGAAATCAGCTGT
GGCTGAGACTACAAACTGCTGGAAATGTAGACCACGTAGGCCTCGGCACTGCGTTCGAAAACAGTATATACGACCAG
GAATACAATATCCGTGTAACCATGTATGTACAATTCAGAGAATTTAATCTTAAAGACCCCCCACTTAACCCT--------

>FJ158603_pcv2b

ATGACGTATCCAAGGAGGCGTTACCGGAGAAGAAGACACCGCCCCCGCAGCCATCTTGGCCAGATCCTCCGCCGCCG
CCCCTGGCTCGTCCACCCCCGCCACCGTTACCGCTGGAGAAGGAAAAATGGCATCTTCAACACCCGCCTCTCCCGCACC
TTCGGATATACTATCGAGCGAACCACAGTCAAAACGCCCTCCTGGGCGGTGGACATGATGAGATTCAATATTAATGAC
TTTCTTCCCCCAGGAGGGGGCTCAAACCCCCGCTCTGTGCCCTTTGAATACTACAGAATAAGAAAGGTTAAGGTTGAA
TTCTGGCCCTGCTCCCCGATCACCCAAGGTGACAGGGGAGTGGGCTCCAGTGCTGTTATTCTAGATGATAACTTTGTA
ACAAAGGCCGCAGCCCTCACCTATGACCCCTATGTAAACTACTCCTCCCGCCATACCATAACCCAGCCCTTCTCCTACCA
CTCCCGCTACTTTACCCCCAAACCTGTCCTAGATTCCACTATTGATTACTTCCAACCAAACAACAAAAGAAATCAGCTGT
GGCTGAGACTACAAACTGCTGGAAATGTAGACCACGTAGGCCTCGGCACTGCGTTCGAAAACAGTATATACGACCAG
GAATACAATATCCGTGTAACCATGTATGTACAATTCAGAGAATTTAATCTTAAAGACCCCCCACTTAACCCT--------

>FJ644930_pcv2b

ATGACGTATCCAGGGAGGCGTTACCGGAGAAGAAGACACCGCCCCCGCAGCCATCTTGGCCAGATCCTCCGCCGCCG
CCCCTGGCTCGTCCACCCCCGCCACCGCTACCGCTGGAGAAGGAAAAATGGCATCTTCAACACCCGCCTCTCCCGCAC
CTTCGGATATACTATCAAGCGAACGACAGTCAAAACGCCCTCCTGGGCGGTGGACATGATGAGATTCAATATTAATGA
CTTTCTTCCCCCAGGAGGGGGCTCAAACCCCCGCTCTGTGCCCTTTGAATACTACAGAATAAGAAAGGTTAAGGTTGA
ATTCTGGCCCTGCTCCCCGATCACCCAGGGTGACAGGGGAGTGGGCTCCAGTGCTGTTATTCTAGATGATAACTTTGT
AACAAAGGCCACAGCCCTCACCTATGACCCCTATGTAAACTACTCCTCCCGCCATACCATCACCCAGCCCTTCTCCTACC
ACTCCCGCTACTTTACCCCCAAACCTGTCCTAGATTCCACTATTGATTACTTCCAACCAAACAACAAAAGAAATCAGCTG
TGGCTGAGACTACAAACTGCTGGAAATGTAGACCACGTAGGCCTCGGCACTGCGTTCGAAAACAGTATATACGACCA
GGAATACAATATCCGTGTAACCATGTATGTACAATTCAGAGAATTTAATCTTAAAGACCCCCCACTTAACCCT--------

>EU257513_pcv2b

ATGACGTATCCAAGGAGGCGTTACCGGAGAAGAAGACACCGCCCCCGCAGCCATCTTGGCCAGATCCTCCGCCGCCG
CCCCTGGCTCGTCCACCCCCGCCACCGTTACCGCTGGAGAAGGAAAAATGGCATCTTCAACACCCGCCTCTCCCGCACC
TTCGGATATACTATCAAGCGAACGACAGTCAAAACGCCCTCCTGGGCGGTGGACATGATGAGATTCAATATTAATGAC
TTTCTTCCCCCAGGAGGGGGCTCAAACCCCCGCTCTGTGCCCTTTGAATACTACAGAATAAGAAAGGTTAAGGTTGAA
TTCTGGCCCTGCTCCCCGATCACCCAGGGTGACAGGGGAGTGGGCTCCAGTGCTGTTATTCTAGATGATAACTTTGTA
ACAAAGGCCACAGCCCTCACCTATGACCCCTATGTAAACTACTCCTCCCGCCATACCATAACCCAGCCCTTCTCCTACCA
CTCCCGCTACTTTACCCCCAAACCTGTCCTAGATTCCACTATTGATTACTTCCAACCAAACAACAAAAGAAATCAGCTGT
GGCTGAGACTACAAACTGCTGGAAATGTAGACCACGTAGGCCTCGGCACTGCGTTCGAAAACAGTATATACGACCAG
GAATACAATATCCGTGTAACCATGTATGTACAATTCAGAGAATTTAATCTTAAAGACCCCCCACTTAACCCT--------

>HM038019_pcv2b

ATGACGTATCCAAGGAGGCGTTACCGGAGAAGAAGACACCGCCCCCGCAGCCATCTTGGCCAGATCCTCCGCCGCCG
CCCCTGGCTCGTCCACCCCCGCCACCGTTGCCGCTGGAGAAGGAAAAATGGCATCTTCAACACCCGCCTCTCCCGCAC
CTTCGGATATACTATCAAGCGAACCACAGTAAAAACGCCCTCCTGGGCGGTGGACATGATGAGATTCAATATTAATGA
CTTTCTTCCCCCAGGAGGGGGCTCAAACCCCCGCTCTGTGCCCTTTGAATACTACAGAATAAGAAAGGTTAAGGTTGA
ATTCTGGCCCTGCTCCCCGATCACCCAGGGTGACAGGGGAGTGGGCTCCAGTGCTGTTATTCTAGATGATAACTTTGT
AACAAAGGCCACAGCCCTCACCTATGACCCCTATGTAAACTACTCCTCCCGCCATACCATAACCCAGCCCTTCTCCTACC
ACTCCCGCTACTTTACCCCCAAACCTGTCCTAGATTCCACTATTGATTACTTCCAACCAAACAACAAAAGAAATCAGCTG
TGGCTGAGACTACAAACTGCTGGAAATGTAGACCACGTAGGCCTCGGCACTGCGTTCGAAAACAGTATATACGACCA
GGAATACAATATCCGTGTAACCATGTATGTACAATTCAGAGAATTTAATCTTAAAGACCCCCCACTTAACCCT--------

>HQ231328_pcv2b

ATGACGTATCCAAGGAGGCGTTACCGGAGAAGAAGACACCGCCCCCGCAGCCATCTTGGCCAGATCCTCCGCCGCCG
CCCCTGGCTCGTCCACCCCCGCCACCGTTGCCGCTGGAGAAGGAAAAATGGCATCTTCAACACCCGCCTCTCCCGCAC
CTTCGGATATACTATCAAGCGAACCACAGTCAAAACGCCCTCCTGGGCGGTGGACATGATGAGATTCAATATTAATGA
CTTTCTTCCCCCAGGAGGGGGCTCAAACCCCCGCTCTGTGCCCTTTGAATACTACAGAATAAGAAAGGTTAAGGTTGA
ATTCTGGCCCTGCTCCCCGATCACCCAGGGTGACAGGGGAGTGGGCTCCAGTGCTGTTATTCTAGATGATAACTTTGT
AACAAAGGCCACAGCCCTCACCTATGACCCCTATGTAAACTACTCCTCCCGCCATACCATAACCCAGCCCTTCTCCTACC
ACTCCCGCTACTTTACCCCCAAACCTGTCCTAGATTCCACTATTGATTACTTCCAACCAAACAACAAAAGAAATCAGCTG
TGGCTGAGACTACAAACTGCTGGAAATGTAGACCACGTAGGCCTCGGCACTGCGTTCGAAAACAGTATATACGACCA
GGAATACAATATCCGTGTAACCATGTATGTACAATTCAGAGAATTTAATCTTAAAGACCCCCCACTTAACCCT--------

>FJ935780_pcv2b

ATGACGTATCCAAGGAGGCGTTACCGGAGAAGAAGACACCGCCCCCGCAGCCATCTTGGCCAGATCCTCCGCCGCCG
CCCCTGGCTCGTCCACCCCCGCCACCGTTACCGCTGGAGAAGGAAAAATGGCATCTTCAACACCCGCCTCTCCCGCACC
TTCGGATATACTATCAAGCGAACCACAGTAAAAACGCCCTCCTGGGCGGTAGACATGATGAGATTCAATATTAATGAC
TTTCTTCCCCCAGGAGGGGGCTCAAACCCCCGCTCTGTGCCCTTTGAATACTACAGAATAAGAAAGGTTAAGGTTGAA
TTCTGGCCCTGCTCCCCGATCACCCAGGGTGACAGGGGAGTGGGCTCCAGTGCTGTTATTCTAGATGATAACTTTGTA
ACAAAGGCCACAGCCCTCACCTATGACCCCTATGTAAACTACTCCTCCCGCCATACCATAACCCAGCCCTTCTCCTACCA
CTCCCGCTACTTTACCCCCAAACCTGTCCTAGATTCCACTATTGATTACTTCCAACCAAACAACAAAAGAAATCAGCTGT
GGCTGAGACTACAAACTGCTGGAAATGTAGACCACGTAGGCCTCGGCACTGCGTTCGAAAACAGTATATACGACCAG
GAATACAATATCCGTGTAACCATGTATGTACAATTCAGAGAATTTAATCTTAAAGACCCCCCACTTAACCCT--------

>HM776442_pcv2b

ATGACGTATCCAAGGAGGCGTTACCGGAGAAGAAGACACCGCCCCCGCAGCCATCTTGGCCAGATCCTCCGCCGCCG
CCCCTGGCTCGTCCACCCCCGCCACCGCTACCGCTGGAGAAGGAAGAATGGCATCTTCAACACCCGCCTCTCCCGCAC
CTTCGGATATACTATCAAGCGAACCACAGTAAAAACGCCCTCCTGGGCGGTGGACATGATGAGATTCAATATTAATGA
CTTTCTTCCCCCAGGAGGGGGCTCAAACCCCCGCTCTGTGCCCTTTGAATACTACAGAATAAGAAAGGTTAAGGTTGA
ATTCTGGCCCTGCTCCCCGATCACCCAGGGTGACAGGGGAGTGGGCTCCAGTGCTGTTATTCTAGATGATAACTTTGT
AACAAAGGCCACAGCCCTCACCTATGACCCCTATGTAAACTACTCCTCCCGCCATACCATAACCCAGCCCTTCTCCTACC
ACTCCCGCTACTTTACCCCCAAACCTGTCCTAGATTCCACTATTGATTACTTCCAACCAAACAACAAAAGAAATCAGCTG
TGGCTGAGACTACAAACTGCTGGAAATGTAGACCACGTAGGCCTCGGCACTGCGTTCGAAAACAGTATATACGACCA
GGAATACAATATCCGTGTAACCATGTATGTACAATTCAGAGAATTTAATCTTAAAGACCCCCCACTTAACCCT--------

>EU366324_pcv2b

ATGACGTATCCAAGGAGGCGTTACCGGAGAAGAAGACACCGCCCCCGCAGCCATCTTGGCCAGATCCTCCGCCGCCG
CCCCTGGCTGGTCCACCCCCGCCACCGCTACCGCTGGAGAAGGAAAAATGGCATCTTCAACACCCGCCTCTCCCGCAC
CTTCGGATATACTATCAAGCGAACCACAGTAAAAACGCCCTCCTGGGCGGTGGACATGATGAGATTCAATATTAATGA
CTTTCTTCCCCCAGGAGGGGGCTCAAACCCCCGCTCTGTGCCCTTTGAATACTACAGAATAAGAAAGGTTAAGGTTGA
ATTCTGGCCCTGCTCCCCGATCACCCAGGGTGACAGGGGAGTGGGCTCCAGTGCTGTTATTCTAGATGATAACTTTGT
AACAAAGGCCACAGCCCTCACCTATGACCCCTATGTAAACTACTCCTCCCGCCATACCATAACCCAGCCCTTCTCCTACC
ACTCCCGCTACTTTACCCCCAAACCTGTCCTAGATTCCACTATTGATTACTTCCAACCAAACAACAAAAGAAATCAGCTG
TGGCTGAGACTACAAACTGCTGGAAATGTAGACCACGTAGGCCTCGGCACTGCGTTCGAAAACAGTATATACGACCA
GGAATACAATATCCGTGTAACCATGTATGTACAATTCAGAGAATTTAATCTTAAAGACCCCCCACTTAACCCT--------

>EU366326_pcv2b

ATGACGTATCCAAGGAGGCGTTACCGGAGAAGAAGACACCGCCCCCGCAGCCATCTTGGCCAGATCCTCCGCCGCCG
CCCCTGGCTGGTCCACCCCCGCCACCGCTACCGCTGGAGAAGGAAAAATGGCATCTTCAACACCCGCCTCTCCCGCAC
CTTCGGATATACTATCAAGCGAACCACAGTAAAAACGCCCTCCTGGGCGGTGGACATGATGAGATTCAATATTAATGA
CTTTCTTCCCCCAGGAGGGGGCTCAAACCCCCGCTCTGTGCCCTTTGAATACTACAGAATAAGAAAGGTTAAGGTTGA
ATTCTGGCCCTGCTCCCCGATCACCCAGGGTGACAGGGGAGTGGGCTCCAGTGCTGTTATTCTAGATGATAACTTTGT
AACAAAGGCCACAGCCCTCACCTATGACCCCTATGTAAACTACTCCTCCCGCCATACCATAACCCAGCCCTTCTCCTACC
ACTCCCGCTACTTTACCCCCAAACCTGTCCTAGATTCCACTATTGATTACTTCCAACCAAACAACAAAAGAAATCAGCTG
TGGCTGAGACTACAAACTGCTGGAAATGTAGACCACGTAGGCCTCGGCACTGCGTTCGAAAACAGTATATACGACCA
GGAATACAATATCCGTGTAACCATGTATGTACAATTCAGAGAATTTAATCTTAAAGACCCCCCACTTAACCCT--------

>FJ660971_pcv2b

ATGACGTATCCAAGGAGGCGTTACCGGAGAAGAAGACACCGCCCCCGCAGCCATCTTGGCCAGATCCTCCGCCGCCG
CCCCTGGCTCGTCCACCCCCGCCACCGTTACCGCTGGAGAAGGAAAAATGGCATCTTCAACACCCGCCTCTCCCGCACC
TTCGGATATACTATCAAGCGAACCACAGTCAAAACGCCCTCCTGGGCGGTAGACATGATGAGATTCAATATTAATGAC
CTTCTTCCCCCAGGAGGGGGCTCAAACCCCCGCTCTGTGCCCTTTGAATACTACAGAATAAGAAAGGTTAAGGTTGAA
TTCTGGCCCTGCTCCCCGATCACCCAGGGTGACAGGGGAGTGGGCTCCAGTGCTGTTATTCTAGATGATAACTTTGTA
ACAAAGGCCACAGCCCTCACCTATGACCCCTATGTAAACTACTCCTCCCGCCATACCATAACCCAGCCTTTCTCCTACCA
CTCCCGCTACTTTACCCCCAAACCTGTCCTAGATTCCACTATTGATTACTTCCAACCAAACTACAAAAGAAATCAGCTGT
GGCTGAGACTACAAACTGCTGGGAATGTAGACCACGTAGGCCTCGGCACTGCGTTCGAAAACAGTATATACGACCAG
GAATACAATATCCGTGTAACCATGTATGTACAATTCAGAGAATTTAATCTTAAAGACCCCCCACTTAACCCTAAG-----

>FJ660970_pcv2b

ATGACGTATCCAAGGAGGCGTTACCGGAGAAGAAGACACCGCCCCCGCAGCCATCTTGGCCAGATCCTCCGCCGCCG
CCCCTGGCTCGTCCACCCCCGCCACCGTTACCGCTGGAGAAGGAAAAATGGCATCTTCAACACCCGCCTCTCCCGCACC
TTCGGATATACTATCAAGCGAACCACAGTCAAAACGCCCTCCTGGGCGGTAGACATGATGAGATTCAATATTAATGAC
TTTCTTCCCCCAGGAGGGGGCTCAAACCCCCGCTCTGTGCCCTTTGAATACTACAGAATAAGAAAGGTTAAGGTTGAA
TTCTGGCCCTGCTCCCCGATCACCCAGGGTGACAGGGGAGTGGGCTCCAGTGCTGTTATTCTAGATGATAACTTTGTA
ACAAAGGCCACAGCCCTCACCTATGACCCCTATGTAAACTACTCCTCCCGCCATACCATAACCCAGCCTTTCTCCTACCA
CTCCCGCTACTTTACCCCCAAACCTGTCCTAGATTCCACTATTGATTACTTCCAACCAAACTACAAAAGAAATCAGCTGT
GGCTGAGACTACAAACTGCTGGGAATGTAGACCACGTAGGCCTCGGCACTGCGTTCGAAAACAGTATATACGACCAG
GAATACAATATCCGTGTAACCATGTATGTACAATTCAGAGAATTTAATCTTAAAGACCCCCCACTTAACCCT--------

>FJ660967_pcv2b

ATGACGTATCCAAGGAGGCGTTACCGGAGAAGAAGACACCGCCCCCGCAGCCATCTTGGCCAGATCCTCCGCCGCCG
CCCCTGGCTCGTCCACCCCCGCCACCGTTACCGCTGGAGAAGGAAAAATGGCATCTTCAACACCCGCCTCTCCCGCACC
TTCGGATATACTATCAAGCGAACCACAGTCAAAACGCCCTCCTGGGCGGTAGACATGATGAGATTCAATATTAATGAC
TTTCTTCCCCCAGGAGGGGGCTCAAACCCCCGCTCTGTGCCCTTTGAATACTACAGAATAAGAAAGGTTAAGGTTGAA
TTCTGGCCCTGCTCCCCGATCACCCAGGGTGACAGGGGAGTGGGCTCCAGTGCTGTTATTCTAGATGATAACTTTGTA
ACAAAGGCCACAGCCCTCACCTATGACCCCTATGTAAACTACTCCTCCCGCCATACCATAACCCAGCCTTTCTCCTACCA
CTCCCGCTACTTTACCCCCAAACCTGTCCTAGATTCCACTATTGATTACTTCCAACCAAACAACAAAAGAAATCAGCTGT
GGCTGAGACTACAAACTGCTGGGAATGTAGACCACGTAGGCCTCGGCACTGCGTTCGAAAACAGTATATACGACCAG
GAATACAATATCCGTGTAACCATGTATGTACAATTCAGAGAATTTAATCTTAAAGACCCCCCACTTAACCCT--------

>JN119256_pcv2b

ATGACGTATCCAAGGAGGCGTTACCGGAGAAGAAGACACCGCCCCCGCAGCCATCTTGGCCAGATCCTCCGCCGCCG
CCCCTGGCTCGTCCACCCCCGCCACCGTTACCGCTGGAGAAGGAAAAATGGCATCTTCAACACCCGCCTCTCCCGCACC
TTCGGATATACTATCAAGCGAACCACAGTCAAAACGCCCTCCTGGGCGGTGGACATGATGAGATTCAATATTAATGAC
TTTCTTCCCCCAGGAGGGGGCTCAAACCCCCGCTCTGTGCCCTTTGAATACTACAGAATAAGAAAGGTTAAGGTTGAA
TTCTGGCCCTGCTCCCCGATCACCCAGGGTGACAGGGGAGTGGGCTCCAGTGCTGTTATTCTAGATGATAACTTTGTA
ACAAAGGCCACAGCCCTCACCTATGACCCCTATGTAAACTACTCCTCCCGCCATACCATAACCCAGCCTTTCTCCTACCA
CTCCCGCTACTTTACCCCCAAACCTGTCCTAGATTCCACTATTGATTACTTCCAACCAAACAACAAAAGAAATCAGCTGT
GGCTGAGACTACAAACTGCTGGGAATGTAGACCACGTAGGCCTCGGCACTGCGTTCGAAAACAGTATATACGACCAG
GAATACAATATCCGTGTAACCATGTATGTACAATTCAGAGAATTTAATCTTAAAGACCCCCCACTTAACCCT--------

>FJ870974_pcv2b

ATGACGTATCCAAGGAGGCGTTACCGGAGAAGAAGACACCGCCCCCGCAGCCATCTTGGCCAGATCCTCCGCCGCCG
CCCCTGGCTCGTCCACCCCCGCCACCGTTACCGCTGGAGAAGGAAAAATGGCATCTTCAACACCCGCCTCTCCCGCACC
TTCGGATATACTATCAAGCGAACCACAGTCAAAACGCCCTCCTGGGCGGTGGACATGATGAGATTCAATATTAATGAC
TTTCTTCCCCCAGGAGGGGGCTCAAACCCCCGCTCTGTGCCCTTTGAATACTACAGAATAAGAAAGGTTAAGGTTGAA
TTCTGGCCCTGCTCCCCGATCACCCAGGGTGACAGGGGAGTGGGCTCCAGTGCTGTTATTCTAGATGATAACTTTGTA
ACAAAGGCCACAGCCCTCACCTATGACCCCTATGTAAACTACTCCTCCCGCCATACCATAACCCAGCCCTTCTCCTACCA
CTCCCGCTACTTTACCCCCAAACCTGTCCTAGATTCCACTATTGATTACTTCCAACCAAACAACAAAAGAAATCAGCTGT
GGCTGAGACTACAAACTGCTGGGAATGTAGACCACGTAGGCCTCGGCACTGCGTTCGAAAACAGTATATACGACCAG
GAATACAATATCCGTGTAACCATGTATGTACAATTCAGAGAATTTAATCTTAAAGACCCCCCACTTAAACCT--------

>FJ644562_pcv2b

ATGACGTATCCAAGGAGGCGTTACCGGAGAAGAAGACACCGCCCCCGCAGCCATCTTGGCCAGATCCTCCGCCGCCG
CCCCTGGCTCGTCCACCCCCGCCACCGTTACCGCTGGAGAAGGAAAAATGGCATCTTCAACACCCGCCTCTCCCGCACC
TTCGGATATACTATCAAGCGAACCACAGTCAAAACGCCCTCCTGGGCGGTGGACATGATGAGATTCAATATTAATGAC
TTTCTTCCCCCAGGAGGGGGCTCAAACCCCCGCTCTGTGCCCTTTGAATACTACAGAATAAGAAAGGTTAAGGTTGAA
TTCTGGCCCTGCTCCCCGATCACCCAGGGTGACAGGGGAGTGGGCTCCAGTGCTGTTATTCTAGATGATAACTTTGTA
ACAAAGGCCACAGCCTTCACCTATGACCCCTATGTAAACTACTCCTCCCGCCATACCATAACCCAGCCCTTCTCCTACCA
CTCCCGCTACTTTACCCCCAAACCTGTCCTAGATTCCACTATTGATTACTTCCAACCAAACAACAAAAGAAATCAGCTGT
GGCTGAGACTACAAACTGCTGGTAATGTAGACCAGGTAGGCCTCGGCACTGCGTTAGAAAACAGTATATACGGCCAG
GAGTACAATATCTGTGTAACCATGTATGTACAATTCAGAGAGTTTAATCTTAAAGACCCCCCACTTAACCCT--------

>GQ359008_pcv2b

ATGACGTATCCAAGGAGGCGTTACCGGAGAAGAAGACACCGCCCCCGCAGCCATCTTGGCCAGATCCTCCGCCGCCG
CCCCTGGCTCGTCCACCCCCGCCACCGTTACCGCTGGAGAAGGAAAAATGGCATCTTCAACACCCGCCTCTCCCGCACC
TTCGGATATACTATCAAGCGAACCACAGTCAAAACGCCCTCCTGGGCGGTGGACATGATGAGATTCAATATTAATGAC
TTTCTACCCCCAGGAGGGGGCTCAAACCCCCGCTCTGTGCCCTTTGAATACTACAGAATAAGAAAGGTTAAGGTTGAA
TTCTGGCCCTGCTCCCCGATCACCCAGGGTGACAGGGGAGTGGGCTCCAGTGCTGTTATTCTAGATGATAACTTTGTA
ACAAAGGCCACAGCCCTAACCTATGACCCCTATGTAAACTACTCCTCCCGCCATACCATAACCCAGCCCTTCTCCTACCA
CTCCCGCTACTTTACCCCCAAACCTGTCCTAGATTCCACTATTGATTACTTCCAACCAAACAACAAAAGAAATCAGCTGT
GGCTGAGACTACAAACTGCTGGGAATGTAGACCACGTAGGCCTCGGCACTGCGTTCGAAAACAGTATATACGACCAG
GATTACAATATCCGTGTAACCATGTATGTACAATTCAGAGAATTTAATCTTAAAGACCCCCCACTTAACCCT--------

>GU247989_pcv2b

ATGACGTATCCAAGGAGGCGTTACCGGAGAAGAAGACACCGCCCCCGCAGCCATCTTGGCCAGATCCTCCGCCGCCG
CCCCTGGCTCGTCCACCCCCGCCACCGTTACCGCTGGAGAAGGAAAAATGGCACCTTCAACACCCGCCTCTCCCGCAC
CTTCGGATATACTATCAAGCGAACCACAGTCAAAACGCCCTCCTGGGCGGTGGACATGATGAGATTCAATATTAATGA
CTTTCTTCCCCCAGGAGGGGGCTCAAACCCCCGCTCTGTGCCCTTTGAATACTACAGAATAAGAAAGGTTAAGGTTGA
ATTCTGGCCCTGCTCCCCGATCACCCAGGGTGACAGGGGAGTGGGCTCCAGTGCTGTTATTCTAGATGATAACTTTGT
AACAAAGGCCACAGCCCTCACCTATGACCCCTATGTAAACTACTCCTCCCGCCATACCATAACCCAGCCCTTCTCCTACC
ACTCCCGCTACTTTACCCCCAAACCTGTCCTAGATTCCACTATTGATTACTTCCAACCAAACAACAAAAGAAATCAGCTG
TGGCTGAGACTACAAACTGCTGGAAATGTAGACCACGTAGGCCTCGGCACTGCGTTCGAAAACAGTATATACGACCA
GGAATACAATATCCGTGTAACCATGTATGTACAATTCAGAGAATTTAATCTTAAAGACCCCCCACTTAACCCT--------

>HM038022_pcv2b

ATGACGTATCCAAGGAGGCGTTACCGGAGAAGAAGACACCGCCCCCGCGGCCATCTTGGCCAGATCCTCCGCCGCCG
CCCCTGGCTCGTCCACCCCCGCCACCGTTACCGCTGGAGAAGGAAAAATGGCACCTTCAACACCCGCCTCTCCCGCAC
CTTCGGATATACTATCAAGCGAACCACAGTCAAAACGCCCTCCTGGGCGGTGGACATGATGAGATTCAATATTAATGA
CTTTCTTCCCCCAGGAGGGGGCTCAAACCCCCGCTCTGTGCCCTTTGAATACTACAGAATAAGAAAGGTTAAGGTTGA
ATTCTGGCCCTGCTCCCCGATCACCCAGGGTGACAGGGGAGTGGGCTCCAGTGCTGTTATTCTAGATGATAACTTTGT
AACAAAGGCCACAGCCCTCACCTATGACCCCTATGTAAACTACTCCTCCCGCCATACCATAACCCAGCCCTTCTCCTACC
ACTCCCGCTACTTTACCCCCAAACCTGTCCTAGATTCCACTATTGATTACTTCCAACCAAACAACAAAAGAAATCAGCTG
TGGCTGAGACTACAAACTGCTGGGAATGTAGACCACGTAGGCCTCGGCACTGCGTTCGAAAACAGTATATACGACCA
GGAATACAATATCCGTGTAACCATGTATGTACAATTCAGAGAATTTAATCTTAAAGACCCCCCACTTAACCCT--------

>JF682792_pcv2b

ATGACGTATCCAAGGAGGCGTTACCGGAGAAGAAGACACCGCCCCCGCGGCCATCTTGGCCAGATCCTCCGCCGCCG
CCCCTGGCTCGTCCACCCCCGCCACCGTTACCGCTGGAGAAGGAAAAATGGCACCTTCAACACCCGCCTCTCCCGCAC
CTTCGGATATACTATCAAGCGAACCACAGTCAAAACGCCCTCCTGGGCGGTGGACATGATGAGATTCAATATTAATGA
CTTTCTTCCCCCAGGAGGGGGCTCAAACCCCCGCTCTGTGCCCTTTGAATACTACAGAATAAGAAAGGTTAAGGTTGA
ATTCTGGCCCTGCTCCCCGATCACCCAGGGTGACAGGGGAGTGGGCTCCAGTGCTGTTATTCTAGATGATAACTTTGT
AACAAAGGCCACAGCCCTCACCTATGACCCCTATGTAAACTACTCCTCCCGCCATACCATAACCCAGCCCTTCTCCTACC
ACTCCCGCTACTTTACCCCCAAACCTGTCCTAGATTCCACTATTGATTACTTCCAACCAAACAACAAAAGAAATCAGCTG
TGGCTGAGACTACAAACTGCTGGGAATGTAGACCACGTAGGCCTCGGCACTGCGTTCGAAAACAGTATATACGACCA
GGAATACAATATCCGTGTAACCATGTATGTACAATTCAGAGAATTTAATCTTAAAGACCCCCCACTTAACCCT--------

>JN382192_pcv2b

ATGACGTATCCAAGGAGGCGTTACCGGAGAAGAAGACACCGCCCCCGCAGCCATCTTGGCCAGATCCTCCGCCGCCG
CCCCTGGCTCGTCCACCCCCGCCACCGTTACCGCTGGAGAAGGAAAAATGGCATCTTCAACACCCGCCTCTCCCGCACC
TTCGGATATACTATCAAGCGAACCACAGTCAAAACCCCCTCCTGGGCGGTGGACATGATGAGATTCAATATTAATGAC
TTTCTTCCCCCAGGAGGGGGCTCAAACCCCCGCTCTGTGCCCTTTGAATACTACAGAATAAGAAAGGTTAAGGTTGAA
TTCTGGCCCTGCTCCCCGATCACCCAGGGTGACAGGGGAGTGGGCTCCAGTGCTGTTATTCTAGATGATAACTTTGTA
ACAAAGGCCACAGCCCTCACCTATGACCCCTATGTAAACTACTCCTCCCGCCATACCATAACCCAGCCCTTCTCCTACCA
CTCCCGCTACTTTACCCCCAAACCTGTCCTAGATTCCACTATTGATTACTTCCAACCAAACAACAAAAGAAATCAGCTGT
GGCTGAGACTACAAACTGCTGGAAATGTAGACCACGTAGGCCTCGGCACTGCGTTCGAAAACAGTATATACGACCAG
GAATACAATATCCGTGTAACCATGTATGTACAATTCAGAGAATTTAATCTTAAAGACCCCCCACTTAACCCT--------

>JN382191_pcv2b

ATGACGTATCCAAGGAGGCGTTACCGGAGAAGAAGACACCGCCCCCGCAGCCATCTCGGCCAGATCCTCCGCCGCCG
CCCCTGGCTCGTCCACCCCCGCCACCGTTACCGCTGGAGAAGGAAAAATGGCATCTTCAACACCCGCCTCTCCCGCACC
TTCGGATATACTATCAAGCGAACCACAGTCAAAACCCCCTCCTGGGCGGTGGACATGATGAGATTCAATATTAATGAC
TTTCTTCCCCCAGGAGGGGGCTCAAACCCCCGCTCTGTGCCCTTTGAATACTACAGAATAAGAAAGGTTAAGGTTGAA
TTCTGGCCCTGCTCCCCGATCACCCAGGGTGACAGGGGAGTGGGCTCCAGTGCTGTTATTCTAGATGATAACTTTGTA
ACAAAGGCCACAGCCCTCACCTATGACCCCTATGTAAACTACTCCTCCCGCCATACCATAACCCAGCCCTTCTCCTACCA
CTCCCGCTACTTTACCCCCAAACCTGTCCTAGATTCCACTATTGATTACTTCCAACCAAACAACAAAAGAAATCAGCTGT
GGCTGAGACTACAAACTGCTGGAAATGTAGACCACGTAGGCCTCGGCACTGCGTTCGAAAACAGTATATACGACCAG
GAATACAATATCCGTGTAACCATGTATGTACAATTCAGAGAATTTAATCTTAAAGACCCCCCACTTAACCCT--------

>JN382190_pcv2b

ATGACGTATCCAAGGAGGCGTTACCGGAGAAGAAGACACCGCCCCCGCAGCCATCTTGGCCAGATCCTCCGCCGCCG

CCCCTGGCTCGTCCACCCCCGCCACCGTTACCGCTGGAGAAGGAAAAATGGCATCTTCAACACCCGCCTCTCCCGCACC

TTCGGATATACTATCAAGCGAACCACAGTCAAAACCCCCTCCTGGGCGGTGGACATGATGAGATTCAATATTAATGAC

TTTCTTCCCCCAGGAGGGGGCTCAAACCCCCGCTCTGTGCCCTTTGAATACTACAGAATAAGAAAGGTTAAGGTTGAA

TTCTGGCCCTGCTCCCCGATCACCCAGGGTGACAGGGGAGTGGGCTCCAGTGCTGTTATTCTAGATGATAACTTTGTA

ACAAAGGCCACAGCCCTCACCTATGACCCCTATGTAAACTACTCCTCCCGCCATACCATAACCCAGCCCTTCTCCTACCA

CTCCCGCTACTTTACCCCCAAACCTGTCCTAGATTCCACTATTGATTACTTCCAACCAAACAACAAAAGAAATCAGCTGT

GGCTGAGACTACAAACTGCTGGAAATGTAGACCACGTAGGCCTCGGCACTGCGTTCGAAAACAGTATATACGACCAG

GAATACAATATCCGTGTAACCATGTATGTACAATTCAGAGAATTTAATCTTAAAGACCCCCCACTTAACCCT--------

>JN382186_pcv2b

ATGACGTATCCAAGGAGGCGTTACCGGAGAAGAAGACACCGCCCCCGCAGCCATCTTGGCCAGATCCTCCGCCGCCG

CCCCTGGCTCGTCCACCCCCGCCACCGTTACCGCTGGAGAAGGAAAAATGGCATCTTCAACACCCGCCTCTCCCGCACC

TTCGGATATACTATCAAGCGAACCACAGTCAAAACCCCCTCCTGGGCGGTGGACATGATGAGATTCAATATTAATGAC

TTTCTTCCCCCAGGAGGGGGCTCAAACCCCCGCTCTGTGCCCTTTGAATACTACAGAATAAGAAAGGTTAAGGTTGAA

TTCTGGCCCTGCTCCCCGATCACCCAGGGTGACAGGGGAGTGGGCTCCAGTGCTGTTATTCTAGATGATAACTTTGTA

ACAAAGGCCACAGCCCTCACCTATGACCCCTATGTAAACTACTCCTCCCGCCATACCATAACCCAGCCCTTCTCCTACCA

CTCCCGCTACTTTACCCCCAAACCTGTCCTAGATTCCACTATTGATTACTTCCAACCAAACAACAAAAGAAATCAGCTGT

GGCTGAGACTACAAACTGCTGGAAATGTGGACCACGTAGGCCTCGGCACTGCGTTCGAAAACAGTATATACGACCAG

GAATACAATATCCGTGTAACCATGTATGTACAATTCAGAGAATTTAATCTTAAAGACCCCCCACTTAACCCT--------

>JQ002672_pcv2b

ATGACGTATCCAAGGAGGCGTTACCGGAGAAGAAGACACCGCCCCCGCAGCCATCTTGGCCAGATCCTCCGCCGCCG

CCCCTGGCTCGTCCACCCCCGCCACCGTTACCGCTGGAGAAGGAAAAATGGCATCTTCAACACCCGCCTCTCCCGCACC

TTCGGATATACTATCAAGCGAACCACAGTCAAAACACCCTCCTGGGCGGTGGACATGATGAGATTCAATATTAATGAC

TTTCTTCCCCCAGGAGGGGGCTCAAACCCCCGCTCTGTGCCCTTTGAATACTACAGAATAAGAAAGGTTAAGGTTGAA

TTCTGGCCCTGCTCCCCGATTACCCAGGGTGACAGGGGAGTGGGCTCCAGTGCTGTTATTCTAGATGATAACTTTGTA

ACAAAGGCCACAGCCCTCACCTATGACCCCTATGTAAACTACTCCTCCCGCCATACCATAACCCAGCCCTTCTCCTACCA

CTCCCGCTACTTTACCCCCAAACCTGTCCTAGATTCCACTATTGATTACTTCCAACCAAACAACAAAAGAAATCAGCTGT

GGCTGAGACTACAAACTGCTGGAAATGTAGACCACGTAGGCCTCGGCACTGCGTTCGAAAACAGTATATACGACCAG

GAATACAATATCCGTGTAACCATGTATGTACAATTCAGAGAATTTAATCTTAAAGACCCCCCACTTAACCCT--------

>HQ831521_pcv2b

ATGACGTATCCAAGGAGGCGTTACCGGAGAAGAAGACACCGCCCCCGCAGCCATCTTGGCCAGATCCTCCGCCGCCG

CCCCTGGCTCGTCCACCCCCGCCACCGTTACCGCTGGAGAAGGAAAAATGGCATCTTCAACACCCGCCTCTCCCGCACC

TTCGGATATACTATCAAGCGAACCACAGTCAAAACACCCTCCTGGGCGGTGGACATGATGAGATTCAATATTAATGAC

TTTCTTCCCCCAGGAGGGGGCTCAAACCCCCGCTCTGTGCCCTTTGAATACTACAGAATAAGAAAGGTTAAGGTTGAA

TTCTGGCCCTGCTCCCCGATCACCCAGGGTGACAGGGGAGTGGGCTCCAGTGCTGTTATTCTAGATGATAACTTTGTA

ACAAAGGCCACAGCCCTCACCTATGACCCCTATGTAAACTACTCCTCCCGCCATACCATAACCCAGCCCTTCTCCTACCA

CTCCCGCTACTTTACCCCCAAACCTGTCCTAGATTCCACTATTGATTACTTCCAACCAAACAACAAAAGAAATCAGCTGT

GGCTGAGACTACAAACTGCTGGAAATGTAGACCACGTAGGCCTCGGGACTGCGTTCGAAAACAGTATATACGACCAG

GAATACAATATCCGTGTAACCATGTATGTACAATTCAGAGAATTTAATCTTAAAGACCCCCCACTTAACCCT--------

>HQ831534_pcv2b

ATGACGTATCCAAGGAGGCGTTACCGGAGAAGAAGACACCGCCCCCGCAGCCATCTTGGCCAGATACTCCGCCGCCG
CCCCTGGCTCGTCCACCCCCGCCACCGTTACCGCTGGAGAAGGAAAAATGGCATCTTCAACACCCGCCTCTCCCGCACC
TTCGGATATACTATCAAGCGAACCACAGTCAAAACACCCTCCTGGGCGGTGGACATGATGAGATTCAATATTAATGAC
TTTCTTCCCCCAGGAGGGGGCTCAAACCCCCGCTCTGTGCCCTTTGAATACTACAGAATAAGAAAGGTTAAGGTTGAA
TTCTGGCCCTGCTCCCCGATCACCCAGGGTGACAGGGGAGTGGGCTCCAGTGCTGTTATTCTAGATGATAACTTTGTA
ACAAAGGCCACAGCCCTCACCTATGACCCCTATGTAAACTACTCCTCCCGCCATACCATAACCCAGCCCTTCTCCTACCA
CTCCCGCTACTTTACCCCCAAACCTGTCCTAGATTCCACTATTGATTACTTCCAACCAAACAACAAAAGAAATCAGCTGT
GGCTGAGACTACAAACTGCTGGAAATGTAGACCACGTAGGCCTCGGGACTGCGTTCGAAAACAGTATATACGACCAG
GAATACAATATCCGTGTAACAATGTATGTACAATTCAGAGAATTTAATCTTAAAGACCCCCCACTTAACCCT--------

>HM776445_pcv2b

ATGACGTATCCAAGGAGGCGTTACCGGAGAAGAAGACACCGCCCCCGCAGCCATCTTGGCCAGATCCTCCGCCGCCG
CCCCTGGCTCGTCCACCCCCGCCACCGTTACCGCTGGAGAAGGAAAAATGGCATCTTCAACACCCGCCTCTCCCGCACC
TTCGGATATACTATCAAGCGAACCACAGTCAAAACGCCCTCCTGGGCGGTGGACATGATGAGATTCAATATTAATGAC
TTTCTTCCCCCAGGAGGGGGCTCAAACCCCCGCTCTGTGCCCTTTGAATACTACAGAATAAGAAAGGTTAAGGTTGAA
TTCTGGCCCTGCTCCCCGATCACCCAGGGTGACAGGGGAGTGGGCTCCAGTGCTGTTATTCTAGATGATAACTTTGTA
ACAAAGACCACAGCCCTCACCTATGACCCCTATGTAAACTACTCCTCCCGCCGTACCATAACCCAGCCCTTCTCCTACCA
CTCCCGCTACTTTACCCCCAAACCTGTCCTAGATTCCACTATTGATTACTTCCAACCAAACAACAAAAGAAATCAACTGT
GGCTGAGACTACAAACTGCTGGAAATGTAGACCACGTAGGCCTCGGCACTGCGTTCGAAAACAGTATATACGACCAG
GAATACAATATCCGTGTAACCATGTATGTACAATTCAGAGAATTTAATCTTAAAGACCCCCCACTTAACCCT--------

>AY641542_pcv2b

ATGACGTATCCAAGGAGGCGTTACCGGAGAAGAAGACACCGCCCCCGCAGCCATCTTGGCCAGATCCTCCGCCGCCG
CCCCTGGCTCGTCCACCCCCGCCACCGTTACCGCTGGAGAAGGAAAAATGGCATCTTCAACACCCGCCTCTCCCGCACC
TTCGGATATACTATCAAGCGAACCACAGTCAAAACGCCCTCCTGGGCGGTGGACATGATGAGATTCAATATTAATGAC
TTTCTTCCCCCAGGAGGGGGCTCAAACCCCCGCTCTGTGCCCTTTGAATACTACAGAATAAGAAAGGTTAAGGTTGAA
TTCTGGCCCTGCTCCCCGATCACCCAGGGTGACAGGGGAGTGGGCTCCAGTGCTGTTATTCTAGATGATAACTTTGTA
ACAAAGACCACAGCCCTCACCTATGACCCCTATGTAAACTACTCCTCCCGCCGTACCATAACCCAGCCCTTCTCCTACCA
CTCCCGCTACTTTACCCCCAAACCTGTCCTAGATTCCACTATTGATTACTTCCAACCAAACAACAAAAGAAATCAACTGT
GGCTGAGACTACAAACTGCTGGAAATGTAGACCACGTAGGCCTCGGCACTGCGTTCGAAAACAGTATATACGACCAG
GAATACAATATCCGTGTAACCATGTATGTACAATTCAGAGAATTTAATCTTAAAGACCCCCCACTTAACCCT--------

>EF524521_pcv2b

ATGACGTATCCAAGGAGGCGTTACCGGAGAAGAAGACACCGCCCCCGCAGCCATCTTGGCCAGATCCTCCGCCGCCG
CCCCTGGCTCGTCCACCCCCGCCACCGTTACCGCTGGAGAAGGAAAAATGGCATCTTCAACACCCGGCTCTCCCGCAC
CTTCGGATATACTATCAAGCGAACCACAGTCAAAACGCCCTCCTGGGCGGTGGACATGATGAGATTCAATATTAATGA
CTTTCTTCCCCCAGGAGGGGGCTCAAACCCCCGCTCTGTGCCCTTTGAATACTACAGAATAAGAAAGGTTAAGGTTGA
ATTCTGGCCCTGCTCCCCGATCACCCAGGGTGACAGGGGAGTGGGCTCCAGTGCTGTTATTCTAGATGATAACTTTGT
AACAAAGGCCACAGCCCTCACCTATGACCCCTATGTAAACTACTCCTCCCGCCATACCATAACCCAGCCCTTCTCCTACC
ACTCCCGCTACTTTACCCCCAAACCTGTCCTAGATTCCACTATTGATTACTTCCAACCAAACAACAAAAGAAATCAACTG
TGGCTGAGACTACAAACTGCTGGAAATGTAGACCACGTAGGCCTCGGCACTGCGTTCGAAAACAGTATATACGACCA
GGAATACAATATCCGTGTAACCATGTATGTACAATTCAGAGAATTTAATCTTAAAGACCCCCCACTTAACCCT--------

>EF524519_pcv2b

ATGACGTATCCAAGGAGGCGTTACCGGAGAAGAAGACACCGCCCCCGCAGCCATCTTGGCCAGATCCTCCGCCGCCG
CCCCTGGCTCGTCCACCCCCGCCACCGTTACCGCTGGAGAAGGAAAAATGGCACCTTCAACACCCGCCTCTCCCGCAC
CTTCGGATATACTATCAAGCGAACCACAGTCAAAACGCCCTCCTGGGCGGTGGACATGATGAGATTCAATATTAATGA
CTTTCTTCCCCCAGGAGGGGGCTCAAACCCCCGCTCTGTGCCCTTTGAATACTACAGAATAAGAAAGGTTAAGGTTGA
ATTCTGGCCCTGCTCCCCGATCACCCAGGGTGACAGGGGAGTGGGCTCCAGTGCTGTTATTCTAGATGATAACTTTGT
AACAAAGGCCACAGCCCTCACCTATGACCCCTATGTAAACTACTCCTCCCGCCATACCATAACCCAGCCCTTCTCCTACC
ACTCCCGCTACTTTACCCCCAAACCTGTCCTAGATTCCACTATTGATTACTTCCAACCAAACAACAAAAGAAATCAACTG
TGGCTGAGACTACAAACTGCTGGAAATGTAGACCACGTAGGCCTCGGCACTGCGTTCGAAAACAGTATATACGACCA
GGAATACAATATCCGTGTAACCATGTATGTACAATTCAGAGAATTTAATCTTAAAGACCCCCCACTTAACCCT--------

>EU545549_pcv2b

ATGACGTATCCAAGGAGGCGTTACCGGAGAAGAAGACACCGCCCCCGCAGCCATCTTGGCCAGATCCTCCGCCGCCG
CCCCTGGCTCGTCCACCCCCGCCACCGTTACCGCTGGAGAAGGAAAAATGGCATCTTCAACACCCGCCTCTCCCGCACC
TTCGGATATACTATCAAGCGAACCACAGTCAAAACGCCCTCCTGGGCGGTGGACATGATGAGATTCAATATTAATGAC
TTTCTTCCCCCAGGAGGGGGCTCAAACCCCCGCTCTGTGCCCTTTGAATACTACAGAATAAGAAAGGTTAAGGTTGAA
TTCTGGCCCTGCTCCCCGATCACCCAGGGTGACAGGGGAGTGGGCTCCAGTGCTGTTATTCTAGATGATAACTTTGTA
ACAAAGGCCACAGCCCTCACCTATGACCCCTATGTAAACTACTCCTCCCGCCATACCATAACCCAGCCCTTCTCCTACCA
CTCCCGCTACTTTACCCCCAAACCTGTCCTAGATTCCACTATTGATTACTTCCAACCAAACAACAAAAGAAATCAACTGT
GGCTGAGACTACAAACTGCTGGAAATGTAGACCACGTAGGCCTCGGCACTGCGTTCGAAAACAGTATATACGACCAG
GAATACAATATCCGTGTAACCATGTATGTACAATTCAGAGAATTTAATCTTAAAGACCCCCCACTTAACCCT--------

>GU325766_pcv2b

ATGACGTATCCAAGGAGGCGTTACCGGAGAAGAAGACACCGCCCCCGCAGCCATCTTGGCCAGATCCTCCGCCGCCG
CCCCTGGCTCGTCCACCCCCGCCACCGTTACCGCTGGAGAAGGAAAGATGGCATCTTCAACACCCGCCTCTCCCGCAC
CTTCGGATATACTATCAAGCGAACCACAGTCAAAACGCCCTCCTGGGCCGTGGACATGATGAGATTCAATATTAATGA
CTTTCTTCCCCCAGGAGGGGGCTCAAACCCCCGCTCTGTGCCCTTTGAATACTACAGAATAAGAAAGGTTAAGGTTGA
ATTCTGGCCCTGCTCCCCGATCACCCAGGGTGACAGGGGAGTGGGCTCCAGTGCTGTTATTCTAGATGATAACTTTGT
AACAAAGGCCACAGCCCTCACCTATGACCCCTATGTAAACTACTCCTCCCGCCATACCATAACCCAGCCCTTCTCCTACC
ACTCCCGCTACTTTACCCCCAAACCTGTCCTAGATTCCACTATTGATTACTTCCAACCAAACAACAAAAGAAATCAACTG
TGGCTGAGACTACAAACTGCTGGAAATGTGGACCACGTAGGCCTCGGCACTGCGTTCGAAAACAGTATATACGACCA
GGAATACAATATCCATGTAACCATGTATGTACAATTCAGAGAATTTAATCTTAAAGACCCCCCACTTAACCCT--------

>JF317575_pcv2b

ATGACGTATCCAAGGAGGCGTTACCGGAGAAGAAGACACCGCCCCCGCAGCCATCTTGGCCAGATCCTCCGCCGCCG
CCCCTGGCTCGTCCACCCCCGCCACCGTTACCGCTGGAGAAGGAAAAATGGCATCTTCAACACCCGCCTCTCCCGCACC
TTCGGATATACTATCAAGCGAACCACAGTCAAAACGCCCTCCTGGGCGGTGGACATGATGAGATTCAATATTAATGAC
TTTCTTCCCCCAGGAGGGGGCTCAAACCCCCGCTCTGTGCCCTTTGAATACTACAGAATAAGAAAGGTTAAGGTTGAA
TTCTGGCCCTGCTCCCCGATCACCCAGGGTGACAGGGGAGTGGGCTCCAGTGCTGTTATTCTAGATGATAACTTTGTA
ACAAAGGCCACAGCCCTCACCTATGACCCCTATGTAAACTACTCCTCCCGCCATACCATAACCCAGCCCTTCTCCTACCA
CTCCCGCTACTTTACCCCCAAACCTGTCCTAGATTCCACTATTGATTACTTCCAACCAAACAACAAAAGAAATCAGCTGT
GGCTGAGACTACAAACTGCTGGAAATGTGGACCACGTAGGCCTCGGCACTGCGTTCGAAAACAGTATATACGACCAG
GAATACAATATCCGTGTAACCATGTATGTACAATTCAGAGAATTTAATCTTAAAGACCCCCCACTTAACCCT--------

>AY536755_pcv2b

ATGACGTATCCAAGGAGGCGTTACCGGAGAAGAAGACACCGCCCCCGCAGCCATCTTGGCCAGATCCTCCGCCGCCG
CCCCTGGCTCGTCCACCCCCGCCACCGTTACCGCTGGAGAAGGAAAAATGGCATCTTCAACACCCGCCTCTCCCGCACC
TTCGGATATACTATCAAGCGAACCACAGTCAAAACGCCCTCCTGGGCGGTGGACATGATGAGATTCAATATTAATGAC
TTTCTTCCCCCAGGAGGGGGCTCAAACCCCCGCTCTGTGCCCTTTGAATACTACAGAATAAGAAAGGTTAAGGTTGAA
TTCTGGCCCTGCTCCCCGATCACCCAGGGTGACAGGGGAGTGGGCTCCAGTGCTGTTATTCTAGATGATAACTTTGTA
ACAAAGGCCACAGCCCTCACCTATGACCCCTATGTAAACTACTCCTCCCGCCATACCATAACCCAGCCCTTCTCCTACCA
CTCCCGCTACTTTACCCCCAAACCTGTCCTAGATTCCACTATTGATTACTTCCAACCAAACAACAAAAGAAATCAGCTGT
GGCTGAGACTACAAACTGCTGGAAATGTGGACCACGTAGGCCTCGGCACTGCGTTCGAAAACAGTATATACGACCAG
GAATACAATATCCGTGTAACCATGTATGTACAATTCAGAGAATTTAATCTTAAAGACCCCCCACTTAACCCT--------

>AY217743_pcv2b

ATGACGTATCCAAGGAGGCGTTACCGGAGAAGAAGACACCGCCCCCGCAGCCATCTTGGCCAGATCCTCCGCCGCCG
CCCCTGGCTCGTCCACCCCCGCCACCGTTACCGCTGGAGAAGGAAAAATGGCATCTTCAACACCCGCCTCTCCCGCACC
TTCGGATATACTATCAAGCGAACCACAGTCAAAACGCCCTCCTGGGCGGTGGACATGATGAGATTCAATATTAATGAC
TTTCTTCCCCCAGGAGGGGGCTCAAACCCCCGCTCTGTGCCCTTTGAATACTACAGAATAAGAAAGGTTAAGGTTGAA
TTCTGGCCCTGCTCCCCGATCACCCAGGGTGACAGGGGAGTGGGCTCCAGTGCTGTTATTCTAGATGATAACTTTGTA
ACAAAGGCCACAGCCCTCACCTATGACCCCTATGTAAACTACTCCTCCCGCCATACCATAACCCAGCCCTTCTCCTACCA
CTCCCGCTACTTTACCCCCAAACCTGTCCTAGATTCCACTATTGATTACTTCCAACCAAACAACAAAAGAAATCAGCTGT
GGCTGAGACTACAAACTGCTGGAAATGTGGACCACGTAGGCCTCGGCACTGCGTTCGAAAACAGTATATACGACCAG
GAATACAATATCCGTGTAACCATGTATGTACAATTCAGAGAATTTAATCTTAAAGACCCCCCACTTAACCCT--------

>AY188355_pcv2b

ATGACGTATCCAAGGAGGCGTTACCGGAGAAGAAGACACCGCCCCCGCAGCCATCTTGGCCAGATCCTCCGCCGCCG
CCCCTGGCTCGTCCACCCCCGCCACCGTTACCGCTGGAGAAGGAAAAATGGCATCTTCAACACCCGCCTCTCCCGCACC
TTCGGATATACTATCAAGCGAACCACAGTCAAAACGCCCTCCTGGGCGGTGGACATGATGAGATTCAATATTAATGAC
TTTCTTCCCCCAGGAGGGGGCTCAAACCCCCGCTCTGTGCCCTTTGAATACTACAGAATAAGAAAGGTTAAGGTTGAA
TTCTGGCCCTGCTCCCCGATCACCCAGGGTGACAGGGGAGTGGGCTCCAGTGCTGTTATTCTAGATGATAACTTTGTA
ACAAAGGCCACAGCCCTCACCTATGACCCCTATGTAAACTACTCCTCCCGCCATACCATAACCCAGCCCTTCTCCTACCA
CTCCCGCTACTTTACCCCCAAACCTGTCCTAGATTCCACTATTGATTACTTCCAACCAAACAACAAAAGAAATCAGCTGT
GGCTGAGACTACAAACTGCTGGAAATGTGGACCACGTAGGCCTCGGCACTGCGTTCGAAAACAGTATATACGACCAG
GAATACAATATCCGTGTAACCATGTATGTACAATTCAGAGAATTTAATCTTAAAGACCCCCCACTTAACCCT--------

>EF190929_pcv2b

ATGACGTATCCAAGGAGGCGTTACCGGAGAAGAAGACACCGCCCCCGCAGCCATCTTGGCCAGATCCTCCGCCGCCG
CCCCTGGCTCGTCCACCCCCGCCACCGTTACCGCTGGAGAAGGAAAAATGGCATCTTCAACACCCGCCTCTCCCGCACC
TTCGGATATACTATCAAGCGAACCACAGTCAAAACGCCCTCCTGGGCGGTGGACATGATGAGATTCAATATTAATGAC
TTTCTTCCCCCAGGAGGGGGCTCAAACCCCCGCTCTGTGCCCTTTGAATACTACAGAATAAGAAAGGTTAAGGTTGAA
TTCTGGCCCTGCTCCCCGATCACCCAGGGTGACAGGGGAGTGGGCTCCAGTGCTGTTATTCTAGATGATAACTTTGTA
ACAAAGGCCACAGCCCTCACCTATGACCCCTATGTAAACTACTCCTCCCGCCATACCATAACCCAGCCCTTCTCCTACCA
CTCCCGCTACTTTACCCCCAAACCTGTCCTAGATTCCACTATTGATTACTTCCAACCAAACAACAAAAGAAATCAGCTGT
GGCTGAGACTACAAACTGCTGGAAATGTGGACCACGTAGGCCTCGGCACTGCGTTCGAAAACAGTATATACGACCAG
GAATACAATATCCGTGTAACCATGTATGTACAATTCAGAGAATTTAATCTTAAAGACCCCCCACTTAACCCT--------

>EF190922_pcv2b

ATGACGTATCCAAGGAGGCGTTACCGGAGAAGAAGACACCGCCCCCGCAGCCATCTTGGCCAGATCCTCCGCCGCCG
CCCCTGGCTCGTCCACCCCCGCCACCGTTACCGCTGGAGAAGGAAAAATGGCATCTTCAACACCCGCCTCTCCCGCACC
TTCGGATATACTATCAAGCGAACCACAGTCAAAACGCCCTCCTGGGCGGTGGACATGATGAGATTCAATATTAATGAC
TTTCTTCCCCCAGGAGGGGGCTCAAACCCCCGCTCTGTGCCCTTTGAATACTACAGAATAAGAAAGGTTAAGGTTGAA
TTCTGGCCCTGCTCCCCGATCACCCAGGGTGACAGGGGAGTGGGCTCCAGTGCTGTTATTCTAGATGATAACTTTGTA
ACAAAGACCACAGCCCTCACCTATGACCCCTATGTAAACTACTCCTCCCGCCATACCATAACCCAGCCCTTCTCCTACCA
CTCCCGCTACTTCACCCCCAAACCTGTCCTAGATTCCACTATTGATTACTTCCAACCAAACAACAAAAGAAATCAGCTGT
GGCTGAGACTACAAACTGCTGGAAATGTGGACCACGTAGGCCTCGGCACTGCGTTCGAAAACAGTATATACGACCAG
GAATACAATATCCGTGTAACCATGTATGTACAATTCAGAGAATTTAATCTTAAAGACCCCCCACTTAAGCCT--------

>AB361569_pcv2b

ATGACGTATCCAAGGAGGCGTTACCGGAGAAGAAGACACCGCCCCCGCAGCCATCTTGGCCAGATCCTCCGCCGCCG
CCCCTGGCTCCTCCACCCCCGCCACCGTTACCGCTGGAGAAGGAAAAATGGCATCTTCAACACCCGCCTCTCCCGCACC
TTCGGATATACTATCAAGCGAACCACAGTCAAAACGCCCTCCTGGGCGGTGGACATGATGAGATTCAATATTAATGAC
TTTCTTCCCCCAGGAGGGGGCTCAAACCCCCGCTCTGTGCCCTTTGAATACTACAGAATAAGAAAGGTTAAGGTTGAA
TTCTGGCCCTGCTCCCCGATCACCCAGGGTGACAGGGGAGTGGGCTCCAGTGCTGTTATTCTAGATGATAACTTTGTA
ACAAAGGCCACAGCCCTCACCTATGACCCCTATGTAAACTACTCCTCCCGCCATACCATAACCCAGCCCTTCTCCTACCA
CTCCCGCTACTTCACCCCCAAACCTGTCCTAGATTCCACTATTGATTACTTCCAACCAAACAACAAAAGAAATCAGCTGT
GGCTGAGACTACAAACTGCTGGAAATGTAGACCACGTAGGCCTCGGCACTGCGTTCGAAAACAGTATATACGACCAG
GAATACAATATCCGTGTAACCATGTATGTACAATTCAGAGAATTTAATCTTAAAGACCCCCCACTTAACCCT--------

>JN006463_pcv2b

ATGACGTATCCAAGGAGGCGTTACCGGAGACGAAGACACCGCCCCCGCAGCCATCTTGGCCAGATCCTCCGCCGCCG
CCCCTGGCTCGTCCACCCCCGCCACCGTTACCGCTGGAGAAGGAAAAATGGCATCTTCAACACCCGCCTCTCCCGCACC
TTCGGATATACTATCAAGCGAACCACAGTCAAAACGCCCTCCTGGGCGGTGGACATGATGAGATTCAATATTAATGAC
TTTCTTCCCCCAGGAGGGGGCTCAAACCCCCGCTCTGTGCCCTTTGAATACTACAGAATAAGAAAGGTTAAGGTTGAA
TTCTGGCCCTGCTCCCCGATCACCCAGGGTGACAGGGGAGTGGGCTCCAGTGCTGTTATTCTAGATGATAACTTTGTA
ACAAAGGCCACAGCCCTCACCTATGACCCCTATGTAAACTACTCCTCCCGCCATACCATAACCCAGCCCTTCTCCTACCA
CTCCCGCTACTTCACCCCCAAACCTGTCCTAGATTCCACTATTGATTACTTCCAACCAAACAACAAAAGAAATCAGCTGT
GGCTGAGACTACAAACTGCTGGAAATGTAGACCACGTAGGCCTCGGCACTGCGTTCGAAAACAGTATATACGACCAG
GAATACAATATCCGTGTAACCATGTATGTACAATTCAGAGAATTTAATCTTAAAGACCCCCCACTTAACCCT--------

>JN006462_pcv2b

ATGACGTATCCAAGGAGGCGTTACCGGAGAAGAAGACACCGCCCCCGCAGCCATCTTGGCCAGATCCTCCGCCGCCG
CCCCTGGCTCGTCCACCCCCGCCACCGTTACCGCTGGAGAAGGAAAAATGGCATCTTCAACACCCGCCTCTCCCGCACC
TTCGGATATACTATCAAGCGAACCACAGTCAAAACGCCCTCCTGGGCGGTGGACATGATGAGATTCAATATTAATGAC
TTTCTTCCCCCAGGAGGGGGCTCAAACCCCCGCTCTGTGCCCTTTGAATACTACAGAATAAGAAAGGTTAAGGTTGAA
TTCTGGCCCTGCTCCCCGATCACCCAGGGTGACAGGGGAGTGGGCTCCAGTGCTGTTATTCTAGATGATAACTTTGTA
ACAAAGGCCACAGCCCTCACCTATGACCCCTATGTAAACTACTCCTCCCGCCATACCATAACCCAGCCCTTCTCCTACCA
CTCCCGCTACTTCACCCCCAAACCTGTCCTAGATTCCACTATTGATTACTTCCAACCAAACAACAAAAGAAATCAGCTGT
GGCTGAGACTACAAACTGCTGGAAATGTAGACCACGTAGGCCTCGGCACTGCGTTCGAAAACAGTATATACGACCAG
GAATACAATATCCGTGTAACCATGTATGTACAATTCAGAGAATTTAATCTTAAAGACCCCCCACTTAACCCT--------

>AB361566_pcv2b

ATGACGTATCCAAGGAGGCGTTACCGGAGAAGAAGACACCGCCCCCGCAGCCATCTTGGCCAGATCCTCCGCCGCCG
CCCCTGGCTCGTCCACCCCCGCCACCGTTACCGCTGGAGAAGGAAAAATGGCATCTTCAACACCCGCCTCTCCCGCACC
TTCGGATATACTATCAAGCGAACCACAGTCAAAACGCCCTCCTGGGCGGTGGACATGATGAGATTCAATATTAATGAC
TTTCTTCCCCCAGGAGGGGGCTCAAACCCCCGCTCTGTGCCCTTTGAATACTACAGAATAAGAAAGGTTAAGGTTGAA
TTCTGGCCCTGCTCCCCGATCACCCAGGGTGACAGGGGAGTGGGCTCCAGTGCTGTTATTCTAGATGATAACTTTGTA
ACAAAGGCCACAGCCCTCACCTATGACCCCTATGTAAACTACTCCTCCCGCCATACCATAACCCAGCCCTTCTCCTACCA
CTCCCGCTACTTCACCCCCAAACCTGTCCTAGATTCCACTATTGATTACTTCCAACCAAACAACAAAAGAAATCAGCTGT
GGCTGAGACTACAAACTGCTGGAAATGTAGACCACGTAGGCCTCGGCACTGCGTTCGAAAACAGTATATACGACCAG
GAATACAATATCCGTGTAACCATGTATGTACAATTCAGAGAATTTAATCTTAAAGACCCCCCACTTAACCCT--------

>AB361567_pcv2b

ATGACGTATCCAAGGAGGCGTTACCGGAGAAGAAGACACCGCCCCCGCAGCCATCTTGGCCAGATCCTCCGCCGCCG
CCCCTGGCTCGTCCACCCCCGCCACCGTTACCGCTGGAGAAGGAAAAATGGCATCTTCAACACCCGCCTCTCCCGCACC
TTCGGATATACTATCAAGCGAACCACAGTCAAAACGCCCTCCTGGGCGGTGGACATGATGAGATTCAATATTAATGAC
TTTCTTCCCCCAGGAGGGGGCTCAAACCCCCGCTCTGTGCCCTTTGAATACTACAGAATAAGAAAGGTTAAGGTTGAA
TTCTGGCCATGCTCCCCGATCACCCAGGGTGACAGGGGAGTGGGCTCCAGTGCTGTTATTCTAGATGATAACTTTGTA
ACAAAGGCCACAGCCCTCACCTATGACCCCTATGTAAACTACTCCTCCCGCCATACCATAACCCAGCCCTTCTCCTACCA
CTCCCGCTACTTCACCCCCAAACCTGTCCTAGATTCCACTATTGATTACTTCCAACCAAACAACAAAAGAAATCAGCTGT
GGCTGAGACTACAAACTGCTGGAAATGTAGACCACGTAGGCCTCGGCACTGCGTTCGAAAACAGTATATACGACCAG
GAATACAATATCCGTGTAACCATGTATGTACAATTCAGAGAATTTAATCTTAAAGACCCCCCACTTAACCCT--------

>FN687845_pcv2b

ATGACGTATCCAAGGAGGCGTTACCGGAGAAGAAGACACCGCCCCCGCAGCCATCTTGGCCAGATCCTCCGCCGCCG
CCCCTGGCTCGTCCACCCCCGCCACCGTTACCGCTGGAGAAGGAAAAATGGCATCTTCAACACCCGCCTCTCCCGCACC
TTCGGATATACTATCAAGCGAACCACAGTCAAAACGCCCTCCTGGGCGGTGGACATGATGAGATTCAATATTAATGAC
TTTCTTCCCCCAGGAGGGGGCTCAAACCCCCGCTCTGTGCCCTTTGAATACTACAGAATAAGAAAGGTTAAGGTTGAA
TTCTGGCCCTGCTCCCCGATCACCCAGGGTGACAGGGGAGTGGGCTCCAGTGCTGTTATTCTAGATGATAACTTTGTA
ACAAAGGCCACAGCCCTCACCTATGACCCCTATGTAAACTACTCCTCCCGCCATACCATAACCCAGCCCTTCTCCTACCA
CTCCCGCTACTTCACCCCCAAACCTGTCCTAGATTCCACTATTGATTACTTCCAACCAAACAACAAAAGAAATCAGCTGT
GGCTGAGACTACAAACTGCTGGAAATGTAGACCACGTAGGCCTCGGCACTGCGTTCGAAAACAGCATATACGACCAG
GAATACAATATCCGTGTAACCATGTATGTACAATTCAGAGAATTTAATCTTAAAGACCCCCCACTTAACCCT--------

>FN687842_pcv2b

ATGACGTATCCAAGGAGGCGTTACCGGAGAAGAAGACACCGCCCCCGCAGCCATCTTGGCCAGATCCTCCGCCGCCG
CCCCTGGCTCGTCCACCCCCGCCACCGTTACCGCTGGAGAAGGAAAAATGGCATCTTCAACACCCGCCTCTCCCGCACC
TTCGGATATACTATCAAGCGAACCACAGTCAAAACGCCCTCCTGGGCGGTGGACATGATGAGATTCAATATTAATGAC
TTTCTTCCCCCAGGAGGGGGCTCAAACCCCCGCTCTGTGCCCTTTGAATACTACAGAATAAGAAAGGTTAAGGTTGAA
TTCTGGCCCTGCTCCCCGATCACCCAGGGTGACAGGGGAGTGGGCTCCAGTGCTGTTATTCTAGATGATAACTTTGTA
ACAAAGGCCACAGCCCTCACCTATGACCCCTATGTAAACTACTCCTCCCGCCATACCATAACCCAGCCCTTCTCCTACCA
CTCCCGCTACTTCACCCCCAAACCTGTCCTAGATTCCACTATTGATTACTTCCAACCAAACAACAAAAGAAATCAGCTGT
GGCTGAGACTACAAACTGCTGGAAATGTAGACCACGTAGGCCTCGGCACTGCGTTCGAAAACAGCATATACGACCAG
GAATACAATATCCGTGTAACCATGTATGTACAATTCAGAGAATTTAATCTTAAAGACCCCCCACTTAACCCT--------

>FN687843_pcv2b

ATGACGTATCCAAGGAGGCGTTACCGGAGAAGAAGACACCGCCCCCGCAGCCATCTTGGCCAGATCCTCCGCCGCCG
CCCCTGGCTCGTCCACCCCCGCCACCGTTACCGCTGGAGAAGGAAAAATGGCATCTTCAACACCCGCCTCTCCCGCACC
TTCGGATATACTATCAAGCGAACCACAGTCAAAACGCCCTCCTGGGCGGTGGACATGATGAGATTCAATATTAATGAC
TTTCTTCCCCCAGGAGGGGGCTCAAACCCCCGCTCTGTGCCCTTTGAATACTACAGAATAAGAAAGGTTAAGGTTGAA
TTCTGGCCCTGCTCCCCGATCACCCAGGGTGACAGGGGAGTGGGCTCCAGTGCTGTTATTCTAGATGATAACTTTGTA
ACAAAGGCCACAGCCCTCACCTATGACCCCTATGTAAACTACTCCTCCCGCCATACCATAACCCAGCCCTTCTCCTACCA
CTCCCGCTACTTCACCCCCAAACCTGTCCTAGATTCCACTATTGATTACTTCCAACCAAACAACAAAAGAAATCAGCTGT
GGCTGAGACTACAAACTGCTGGAAATGTAGACCACGTAGGCCTCGGCACTGCGTTCGAAAACAGCATATACGACCAG
GAATACAATATCCGTGTAACCATGTATGTACAATTCAGAGAATTTAATCTTAAAGACCCCCCACTTAACCCT--------

>FN687846_pcv2b

ATGACGTATCCAAGGAGGCGTTACCGGAGAAGAAGACACCGCCCCCGCAGCCATCTTGGCCAGATCCTCCGCCGCCG
CCCCTGGCTCGTCCACCCCCGCCACCGTTACCGCTGGAGAAGGAAAAATGGCATCTTCAACACCCGCCTCTCCCGCACC
TTCGGATATACTATCAAGCGAACCACAGTCAAAACGCCCTCCTGGGCGGTGGACATGATGAGATTCAATATTAATGAC
TTTCTTCCCCCAGGAGGGGGCTCAAACCCCCGCTCTGTGCCCTTTGAATACTACAGAATAAGAAAGGTTAAGGTTGAA
TTCTGGCCCTGCTCCCCGATCACCCAGGGTGACAGGGGAGTGGGCTCCAGTGCTGTTATTCTAGATGATAACTTTGTA
ACAAAGGCCACAGCCCTCACCTATGACCCCTATGTAAACTACTCCTCCCGCCATACCATAACCCAGCCCTTCTCCTACCA
CTCCCGCTACTTCACCCCCAAACCTGTCCTAGATTCCACTATTGATTACTTCCAACCAAACAACAAAAGAAATCAGCTGT
GGCTGAGACTACAAACTGCTGGAAATGTAGACCACGTAGGCCTCGGCACTGCGTTCGAAAACAGCATATACGACCAG
GAATACAATATCCGTGTAACCATGTATGTACAATTCAGAGAATTTAATCTTAAAGACCCCCCACTTAACCCT--------

>FN687844_pcv2b

ATGACGTATCCAAGGAGGCGTTACCGGAGAAGAAGACACCGCCCCCGCAGCCATCTTGGCCAGATCCTCCGCCGCCG
CCCCTGGCTCGTCCACCCCCGCCACCGTTACCGCTGGAGAAGGAAAAATGGCATCTTCAACACCCGCCTCTCCCGCACC
TTCGGATATACTATCAAGCGAACCACAGTCAAAACGCCCTCCTGGGCGGTGGACATGATGAGATTCAATATTAATGAC
TTTCTTCCCCCAGGAGGGGGCTCAAACCCCCGCTCTGTGCCCTTTGAATACTACAGAATAAGAAAGGTTAAGGTTGAA
TTCTGGCCCTGCTCCCCGATCACCCAGGGTGACAGGGGAGTGGGCTCCAGTGCTGTTATTCTAGATGATAACTTTGTA
ACAAAGGCCACAGCCCTCACCTATGACCCCTATGTAAACTACTCCTCCCGCCATACCATAACCCAGCCCTTCTCCTACCA
CTCCCGCTACTTCACCCCCAAACCTGTCCTAGATTCCACTATTGATTACTTCCAACCAAACAACAAAAGAAATCAGCTGT
GGCTGAGACTACAAACTGCTGGAAATGTAGACCACGTAGGCCTCGGCACTGCGTTCGAAAACAGCATATACGACCAG
GAATACAATATCCGTGTAACCATGTATGTACAATTCAGAGAATTTAATCTTAAAGACCCCCCACTTAACCCT--------

>JN382172_pcv2b

ATGACGTATCCAAGGAGGCGTTACCGGAGAAGAAGACACCGCCCCCGCAGCCATCTTGGCCAGATCCTCCGCCGCCG
CCCCTGGCTCGTCCACCCCCGCCACCGTTACCGCTGGAGAAGGAAAAATGGCATCTTCAACACCCGCCTCTCCCGCACC
TTCGGATATACTATCAAGCGAACCACAGTCAAAACGCCCTCCTGGGCGGTGGACATGATGAGATTCAATATTAATGAC
TTTCTTCCCCCAGGAGGGGGCTCAAACCCCCGCTCTGTGCCCTTTGAATACTACAGAATAAGAAAGGTTAAGGTTGAA
TTCTGGCCCTGCTCCCCGATCACCCAGGGTGACAGGGGAGTGGGCTCCAGTGCTGTTATTCTAGATGATAACTTTGTA
ACAAAGGCCACAGCCCTCACCTATGACCCCTATGTAAACTACTCCTCCCGCCATACCATAACCCAGCCCTTCTCCTACCA
CTCCCGCTACTTTACCCCCAAACCTGTCCTAGATTCCACTATTGATTACTTCCAACCAAACAACAAAAGAAATCAGCTGT
GGCTGAGACTACAAACTGCTGGAAATGTAGACCACGTAGGCCTCGGCACTGCGTTCGAAAACAGTATATACGACCAG
GAATACAATATCCGTGTAACCATGTATGTACAATTCAGAGAATTTAATCTTAAAGACCCCCCACTTAACCCT--------

>EF371541_pcv2b

ATGACGTATCCAAGGAGGCGTTACCGGAGAAGAAGACACCGCCCCCGCAGCCATCTTGGCCAGATCCTCCGCCGCCG
CCCCTGGCTCGTCCACCCCCGCCACCGTTACCGCTGGAGAAGGAAAAATGGCATCTTCAACACCCGCCTCTCCCGCACC
TTCGGATATACTATCAAGCGAACCACAGTCAAAACGCCCTCCTGGGCGGTGGACATGATGAGATTCAATATTAATGAC
TTTCTTCCCCCAGGAGGGGGCTCAAACCCCCGCTCTGTGCCCTTTGAATACTACAGAATAAGAAAGGTTAAGGTTGAA
TTCTGGCCCTGCTCCCCGATCACCCAGGGTGACAGGGGAGTGGGCTCCAGTGCTGTTATTCTAGATGATAACTTTGTA
ACAAAGGCCACAGCCCTCACCTATGACCCCTATGTAAACTACTCCTCCCGCCATACCATAACCCAGCCCTTCTCCTACCA
CTCCCGCTACTTTACCCCCAAACCTGTCCTAGATTCCACTATTGATTACTTCCAACCAAACAACAAAAGAAATCAGCTGT
GGCTGAGACTACAAACTGCTGGAAATGTAGACCACGTAGGCCTCGGCACTGCGTTCGAAAACAGTATATACGACCAG
GAATACAATATCCGTGTAACCATGTATGTACAATTCAGAGAATTTAATCTTAAAGACCCCCCACTTAACCCT--------

>HQ591373_pcv2b

ATGACGTATCCAAGGAGGCGTTACCGGAGAAGAAGACACCGCCCCCGCAGCCATCTTGGCCAGATCCTCCGCCGCCG
CCCCTGGCTCGTCCACCCCCGCCACCGTTACCGCTGGAGAAGGAAAAATGGCATCTTCAACACCCGCCTCTCCCGCACC
TTCGGATATACTATCAAGCGAACCACAGTCAAAACGCCCTCCTGGGCGGTGGACATGATGAGATTCAATATTAATGAC
TTTCTTCCCCCAGGAGGGGGCTCAAACCCCCGCTCTGTGCCCTTTGAATACTACAGAATAAGAAAGGTTAAGGTTGAA
TTCTGGCCCTGCTCCCCGATCACCCAGGGTGACAGGGGAGTGGGCTCCAGTGCTGTTATTCTAGATGATAACTTTGTA
ACAAAGGCCACAGCCCTCACCTATGACCCCTATGTAAACTACTCCTCCCGCCATACCATAACCCAGCCCTTCTCCTACCA
CTCCCGCTACTTTACCCCCAAACCTGTCCTAGATTCCACTATTGATTACTTCCAACCAAACAACAAAAGAAATCAGCTGT
GGCTGAGACTACAAACTGCTGGAAATGTAGACCACGTAGGCCTCGGCACTGCGTTCGAAAACAGTATATACGACCAG
GAATACAATATCCGTGTAACCATGTATGTACAATTCAGAGAATTTAATCTTAAAGACCCCCCACTTAACCCT--------

>AY321995_pcv2b

ATGACGTATCCAAGGAGGCGTTACCGGAGAAGAAGACACCGCCCCCGCAGCCATCTTGGCCAGATCCTCCGCCGCCG
CCCCTGGCTCGTCCACCCCCGCCACCGTTACCGCTGGAGAAGGAAAAATGGCATCTTCAACACCCGCCTCTCCCGCACC
TTCGGATATACTATCAAGCGAACCACAGTCAAAACGCCCTCCTGGGCGGTGGACATGATGAGATTCAATATTAATGAC
TTTCTTCCCCCAGGAGGGGGCTCAAACCCCCGCTCTGTGCCCTTTGAATACTACAGAATAAGAAAGGTTAAGGTTGAA
TTCTGGCCCTGCTCCCCGATCACCCAGGGTGACAGGGGAGTGGGCTCCAGTGCTGTTATTCTAGATGATAACTTTGTA
ACAAAGGCCACAGCCCTCACCTATGACCCCTATGTAAACTACTCCTCCCGCCATACCATAACCCAGCCCTTCTCCTACCA
CTCCCGCTACTTTACCCCCAAACCTGTCCTAGATTCCACTATTGATTACTTCCAACCAAACAACAAAAGAAATCAGCTGT
GGCTGAGACTACAAACTGCTGGAAATGTAGACCACGTAGGCCTCGGCACTGCGTTCGAAAACAGTATATACGACCAG
GAATACAATATCCGTGTAACCATGTATGTACAATTCAGAGAATTTAATCTTAAAGACCCCCCACTTAACCCT--------

>EF493837_pcv2b

ATGACGTATCCAAGGAGGCGTTACCGGAGAAGAAGACACCGCCCCCGCAGCCATCTTGGCCAGATCCTCCGCCGCCG
CCCCTGGCTCGTCCACCCCCGCCACCGTTACCGCTGGAGAAGGAAAAATGGCATCTTCAACACCCGCCTCTCCCGCACC
TTCGGATATACTATCAAGCGAACCACAGTCAAAACGCCCTCCTGGGCGGTGGACATGATGAGATTCAATATTAATGAC
TTTCTTCCCCCAGGAGGGGGCTCAAACCCCCGCTCTGTGCCCTTTGAATACTACAGAATAAGAAAGGTTAAGGTTGAA
TTCTGGCCCTGCTCCCCGATCACCCAGGGTGACAGGGGAGTGGGCTCCAGTGCTGTTATTCTAGATGATAACTTTGTA
ACAAAGGCCACAGCCCTCACCTATGACCCCTATGTAAACTACTCCTCCCGCCATACCATAACCCAGCCCTTCTCCTACCA
CTCCCGCTACTTTACCCCCAAACCTGTCCTAGATTCCACTATTGATTACTTCCAACCAAACAACAAAAGAAATCAGCTGT
GGCTGAGACTACAAACTGCTGGAAATGTAGACCACGTAGGCCTCGGCACTGCGTTCGAAAACAGTATATACGACCAG
GAATACAATATCCGTGTAACCATGTATGTACAATTCAGAGAATTTAATCTTAAAGACCCCCCACTTAACCCT--------

>EF565363_pcv2b

ATGACGTATCCAAGGAGGCGTTACCGGAGAAGAAGACACCGCCCCCGCAGCCATCTTGGCCAGATCCTCCGCCGCCG
CCCCTGGCTCGTCCACCCCCGCCACCGTTACCGCTGGAGAAGGAAAAATGGCATCTTCAACACCCGCCTCTCCCGCACC
TTCGGATATACTATCAAGCGAACCACAGTCAAAACGCCCTCCTGGGCGGTGGACATGATGAGATTCAATATTAATGAC
TTTCTTCCCCCAGGAGGGGGCTCAAACCCCCGCTCTGTGCCCTTTGAATACTACAGAATAAGAAAGGTTAAGGTTGAA
TTCTGGCCCTGCTCCCCGATCACCCAGGGTGACAGGGGAGTGGGCTCCAGTGCTGTTATTCTAGATGATAACTTTGTA
ACAAAGGCCACAGCCCTCACCTATGACCCCTATGTAAACTACTCCTCCCGCCATACCATAACCCAGCCCTTCTCCTACCA
CTCCCGCTACTTTACCCCCAAACCTGTCCTAGATTCCACTATTGATTACTTCCAACCAAACAACAAAAGAAATCAGCTGT
GGCTGAGACTACAAACTGCTGGAAATGTAGACCACGTCGGCCTCGGCACTGCGTTCGAAAACAGTATATACGACCAG
GAATACAATATCCGTGTAACCATGTATGTACAATTCAGAGAATTTAATCTTAAAGACCCCCCACTTAACCCT--------

>HM038028_pcv2b

ATGACGTATCCAAGGAGGCGTTACCGGAGAAGAAGACACCGCCCCCGCAGCCATCTTGGCCAGATCCTCCGCCGCCG
CCCCTGGCTCGTCCACCCCCGCCACCGTTACCGCTGGAGAAGGAAAAATGGCATCTTCAACACCCGCCTCTCCCGTACC
TTCGGATATACTATCAAGCGAACCACAGTCAAAACGCCCTCCTGGGCGGTGGACATGATGAGATTCAATATTAATGAC
TTTCTTCCCCCAGGAGGGGGCTCAAACCCCCGCTCTGTGCCCTTTGAATACTACAGAATAAGAAAGGTTAAGGTTGAA
TTTTGGCCCTGCTCCCCGATCACCCAGGGTGACAGGGGAGTGGGCTCCAGTGCTGTTATTCTAGATGATAACTTTGTA
ACAAAGGCCACAGCCCTCACCTATGACCCCTATGTAAACTACTCCTCCCGCCATACCATAACCCAGCCCTTCTCCTACCA
CTCCCGCTACTTTACCCCCAAACCTGTCCTAGATTCCACTATTGATTACTTCCAACCAAACAACAAAAGAAATCAGCTGT
GGCTGAGACTACAAACTGCTGGAAATGTAGACCACGTAGGCCTCGGCACTGCGTTCGAAAACAGTATATACGACCAG
GAATACAATATCCGTGTAACCATGTATGTACAATTCAGAGAATTTAATCTTAAAGACCCCCCACTTAACCCT--------

>AY732494_pcv2b

ATGACGTATCCAAGGAGGCGTTACCGGAGAAGAAGACACCGCCCCCGCAGCCATCTTGGCCAGATCCTACGCCGCCG
CCCCTGGCTCGTCCACCCCCGCCACCGTTACCGCTGGAGAAGGAAAAATGGCATCTTCAACACCCGCCTCTCCCGTACC
TTCGGATATACTATCAAGCGAACCACAGTCAAAACGCCCTCCTGGGCGGTGGACATGATGAGATTCAATATTAATGAC
TTTCTTCCCCCAGGAGGGGGCTCAAACCCCCGCTCTGTGCCCTTTGAATACTACAGAATAAGAAAGGTTAAGGTTGAA
TTCTGGCCCTGCTCCCCGATCACCCAGGGTGACAGGGGAGTGGGCTCCAGTGCTGTTATTCTAGATGATAACTTTGTA
ACAAAGGCCACAGCCCTCACCTATGACCCCTATGTAAACTACTCCTCCCGCCATACCATAACCCAGCCCTTCTCCTACCA
CTCCCGCTACTTTACCCCCAAACCTGTCCTAGATTCCACTATTGATTACTTCCAACCAAACAACAAAAGAAATCAGCTGT
GGCTGAGACTACAAACTGCTGGAAATGTAGACCACGTAGGCCTCGGCACTGCGTTCGAAAACAGTATATACGACCAG
GAATACAATATCCGTGTAACCATGTATGTACAATTCAGAGAATTTAATCTTAAAGACCCCCCACTTAACCCT--------

>FJ644919_pcv2b

ATGACGTATCCAAGGAGGCGTTACCGGAGAAGAAGACACCGCCCCCGCAGCCATCTTGGCCAGATCCTCCGCCGCCG
CCCCTGGCTCGTCCACCCCCGCCACCGTTACCGCTGGAGAAGGAAAAATGGCATCTTCAACACCCGCCTCTCCCGTACC
TTCGGATATACTATCAAGCGAACCACAGTCAAAACGCCCTCCTGGGCGGTGGACATGATGAGATTCAATATTAATGAC
TTTCTTCCCCCAGGAGGGGGCTCAAACCCCCGCTCTGTGCCCTTTGAATACTACAGAATAAGAAAGGTTAAGGTTGAA
TTCTGGCCCTGCTCCCCGATCACCCAGGGTGACAGGGGAGTGGGCTCCAGTGCTGTTATTCTAGATGATAACTTTGTA
ACAAAGGCCACAGCCCTCACCTATGACCCCTATGTAAACTACTCCTCCCGCCATACCATAACCCAGCCCTTCTCCTACCA
CTCCCGCTACTTTACCCCCAAACCTGTCCTAGATTCCACTATTGATTACTTCCAACCAAACAACAAAAGAAATCAGCTGT
GGCTGAGACTACAAACTGCTGGAAATGTAGACCACGTAGGCCTCGGCACTGCGTTCGAAAACAGTATATACGACCAG
GAATACAATATCCGTGTAACCATGTATGTACAATTCAGAGAATTTAATCTTAAAGACCCCCCACTTAACCCT--------

>EU555439_pcv2b

ATGACGTATCCAAGGAGGCGTTACCGGAGAAGAAGACACCGCCCCCGCAGCCATCTTGGCCAGATCCTCCGCCGCCG
CCCCTGGCTCGTCCACCCCCGCCACCGTTACCGCTGGAGAAGGAAAAATGGCATCTTCAACACCCGCCTCTCCCGTACC
TTCGGATATACTATCAAGAGAACCACAGTCAAAACGCCCTCCTGGGCTGTGGACATGATGAGATTCAATATTAATGAC
TTTCTTCCCCCAGGAGGGGGCTCAAACCCCCGCTCTGTGCCCTTTGAATACTACAGAATAAGAAAGGTTAAGGTTGAA
TTCTGGCCCTGCTCCCCGATCACCCAGGGTGACAGGGGAGTGGGCTCCAGTGCTGTTATTCTAGATGATAACTTTGTA
ACAAAGGCCACAGCCCTCACCTATGACCCCTATGTAAACTACTCCTCCCGCCATACCATAACCCAGCCCTTCTCCTACCA
CTCCCGCTACTTTACCCCCAAACCTGTCCTAGATTCCACTATTGATTACTTCCAACCAAACAACAAAAGAAATCAGCTGT
GGCTGAGACTACAAACTGCTGGAAATGTAGACCACGTAGGCCTCGGCACTGCGTTCGAAAACAGTATATACGACCAG
GAATACAATATCCGTGTAACCATGTATGTACAATTCAGAGAATTTAATCTTAAAGACCCCTCACTTAACCCT--------

>EU589623_pcv2b

ATGACGTATCCAAGGAGGCGTTACCGGAGAAGAAGACACCGCCCCCGCAGCCATCTTGGCCAGATCCTCCGCCGCCG
CCCCTGGCTCGTCCACCCCCGCCACCGTTACCGCTGGAGAAGGAAAAATGGCATCTTCAACACCCGCCTCTCCCGTACC
TTCGGATATACTATCAAGAGAACCACAGTCAAAACGCCCTCCTGGGCTGTGGACATGATGAGATTCAATATTAATGAC
TTTCTTCCCCCAGGAGGGGGCTCAAACCCCCGCTCTGTGCCCTTTGAATACTACAGAATAAGAAAGGTTAAGGTTGAA
TTCTGGCCCTGCTCCCCGATCACCCAGGGTGACAGGGGAGTGGGCTCCAGTGCTGTTATTCTAGATGATAACTTTGTA
ACAAAGGCCACAGCCCTCACCTATGACCCCTATGTAAACTACTCCTCCCGCCATACCATAACCCAGCCCTTCTCCTACCA
CTCCCGCTACTTTACCCCCAAACCTGTCCTAGATTCCACTATTGATTACTTCCAACCAAACAACAAAAGAAATCAGCTGT
GGCTGAGACTACAAACTGCTGGAAATGTAGACCACGTAGGCCTCGGCACTGCGTTCGAAAACAGTATATACGACCAG
GAATACAATATCCGTGTAACCATGTATGTACAATTCAGAGAATTTAATCTTAAAGACCCCCCACTTAACCCA--------

>EF565362_pcv2b

ATGACGTATCCAAGGAGGCGTTACCGGAGAAGAAGACACCGCCCCCGCAGCCATCTTGGCCAGATCCTCCGCCGCCG
CCCCTGGCTCGTCCACCCCCGCCACCGTTACCGCTGGAGAAGGAAAAATGGCATCTTCAACACCCGCCTCTCCCGAACC
TTCGGATATACTATCAAGCGAACCACAGTCAAAACGCCCTCCTGGGCGGTGGACATGATGAGATTCAATATTAATGAC
TTTCTTCCCCCAGGAGGGGGCTCAAACCCCCGCTCTGTGCCCTTTGAATACTACAGAATAAGAAAGGTTAAGGTTGAA
TTCTGGCCCTGCTCCCCGATCACCCAGGGTGACAGGGGAGTGGGCTCCAGTGCTGTTATTCTAGATGATAACTTTGTA
ACAAAGGCCACAGCCCTCACCTATGACCCCTATGTAAACTACTCCTCCCGCCATACCATAACCCAGCCCTTCTCCTACCA
CTCCCGCTACTTTACCCCCAAACCTGTCCTAGATTCCACTATTGATTACTTCCAACCAAACAACAAAAGAAATCAGCTGT
GGCTGAGACTACAAACTGCTGGAAATGTAGACCACGTAGGCCTCGGCACTGCGTTCGAAAACAGTATATACGACCAG
GAATACAATATCCGTGTAACCATGTATGTACAATTCAGAGAATTTAATCTTAAAGACCCCCCACTTAACCCT--------

>HQ831538_pcv2b

ATGACGTATCCAAGGAGGCGTTACCGGAGAAGAAGACACCGCCCCCGCAGCCATCTTGGCCAGATCCTCCGCCGCCG
CCCCTGGCTCGTCCACCCCCGCCACCGTTACCGCTGGAGAAGGAAAAATGGCATCTTCAACACCCGCCTCTCCCGAACC
TTCGGATATACTATCAAGCGAACCACAGTCAAAACGCCCTCTTGGGCGGTGGACATGATGAGATTCAATATTAATGAC
TTTCTTCCCCCAGGAGGGGGCTCAAACCCCCGCTCTGTGCCCTTTGAATACTACAGAATAAGAAAGGTTAAGGTTGAA
TTCTGGCCCTGCTCCCCGATCACCCAGGGTGACAGGGGAGTGGGCTCCAGTGCTGTTATTCTAGATGATAACTTTGTA
ACAAAGGCCACAGCCCTCACCTATGACCCCTATGTAAACTACTCCTCCCGCCATACCATAACCCAGCCCTTCTCCTACCA
CTCCCGCTACTTTACCCCCAAACCTGTCCTAGATTCCACTATTGATTACTTCCAACCAAACAACAAAAGAAATCAGCTGT
GGCTGAGACTACAAACTGCTGGAAATGTAGACCACGTAGGCCTCGGCACTGCGTTCGAAAACAGTATATATGACCAG
GAATACAATATCCGTGTAACCATGTATGTACAATTCAGAGAATTTAATCTTAAAGACCCCCCACTTAACCCT--------

>JF317588_pcv2b

ATGACGTATCCAAGGAGGCGTTACCGGAGAAGAAGACACCGCCCCCGCAGCCATCTTGGCCAGATCCTCCGCCGCCG
CCCCTGGCTCGTCCACCCCCGCCTCCGTTACCGCTGGAGAAGGAAAAATGGCATCTTCAACACCCGCCTCTCCCGTACC
TTCGGATATACTATCAAGCGAACCACAGTCAAAACGCCCTCTTGGGCGGTGGACATGATGAGATTCAATATTAATGAC
TTTCTTCCCCCAGGAGGGGGCTCAAACCCCCGCTCTGTGCCCTTTGAATACTACAGAATAAGAAAGGTTAAGGTTGAA
TTCTGGCCCTGCTCCCCGATCACCCAGGGTGACAGGGGAGTGGGCTCCAGTGCTGTTATTCTAGATGATAACTTTGTA
ACAAAGGCCACAGCCCTCACCTATGACCCCTATGTAAACTACTCCTCCCGCCATACCATAACCCAGCCCTTCTCCTACCA
CTCCCGCTACTTTACCCCCAAACCTGTCCTAGATTCCACTATTGATTACTTCCAACCAAACAACAAAAGAAATCAGCTGT
GGCTGAGACTACAAACTGCTGGAAATGTAGACCACGTAGGCCTCGGCACTGCGTTCGAAAACAGTATATACGACCAG
GAATACAATATCCGTGTAACCATGTATGTACAATTCAGAGAATTTAATCTTAAAGACCCCCCACTTAACCCT--------

>HM038023_pcv2b

ATGACGTATCCAAGGAGGCGTTACCGGAGAAGAAGACACCGCCCCCGCAGCCATCTTGGCCAGATCCTCCGCCGCCG
CCCCTGGCTCGTCCACCCCCGCCACCGTTACCGCTGGAGAAGGAAAAATGGCATCTTCAACACCCGCCTCTCCCGTACC
TTCGGATATACTATCAAGCGAACCACAGTCAAAACGCCCTCCTGGGCGGTGGACATGATGAGATTCAATATTAATGAC
TTTCTTCCCCCAGGAGGGGGCTCAAACCCCCGCTCTGTGCCCTTTGAATACTACAGAATAAGAAAGGTTAAGGTTGAA
TTCTGGCCCTGCTCCCCGATCACCCAGGGTGACAGGGGAGTGGGCTCCAGTGCTGTTATTCTAGATGATAACTTTGTA
ACAAAGGCCACAGCCCTCACCTATGACCCCTATGTAAACTACTCCTCCCGCCATACCATAACCCAGCCCTTCTCCTACCA
CTCCCGCTACTTTACCCCCAAACCTGTCCTAGATTCCACTATTGATTACTTCCAACCAAACAACAAAAGAAATCAGCTGT
GGCTGAGACTACAAACTGCTGGAAATATAGACCACGTAGGCCTCGGCACTGCGTTCGAAAACAGTATATACGACCAG
GAATACAATATCCGTGTAACCATGTATGTACAATTCAGAGAATTTAATCTTAAAGACCCCCCACTTAACCCT--------

>FN687857_pcv2b

ATGACGTATCCAAGGAGGCGTTACCGGAGAAGAAGACACCGCCCCCGCAGCCATCTTGGCCAGATCCTCCGCCGCCG
CCCCTGGCTCGTCCACCCCCGCCACCGTTACCGCTGGAGAAGGAAAAATGGCATCTTCAACACCCGCCTCTCCCGCACC
TTCGGATATACTATCAAGCGAACCACAGTCAAAACGCCCTCCTGGGCGGTGGACATGATGAGATTCAATCTTAATGAC
TTTCTTCCCCCAGGAGGGGGCTCAAACCCCCGCTCTGTGCCCTTTGAATACTACAGAATAAGAAAAGTTAAGGTTGAA
TTCTGGCCCTGCTCCCCGATCACCCAGGGTGACAGGGGAGTGGGCTCCAGTGCTATTATTCTAGATGATAACTTTGTA
CCAAAGGCCACAGCCCTCACCTATGACCCCTATGTAAACTACTCCTCCCGCCATACCATAACCCAGCCCTTCTCCTACCA
CTCCCGCTACTTTACCCCCAAACCTGTCCTAGATTCCACTATTGATTACTTCCAACCAAACAACAAAAGAAATCAGCTGT
GGCTGAGGCTACAAACTGCTGGAAATATAGACCACGTAGGCCTCGGCACTGCGTTCGAAAACAGTATATACGACCAG
GAATACAATATCCGTGTAACCATGTATGTACAATTCAGAGAATTTAATCTTAAAGACCCCCCACTTAACCCT--------

>EF371550_pcv2b

ATGACGTATCCAAGGAGGCGTTACCGGAGAAGAAGACACCGCCCCCGCAGCCATCTTGGCCAGATCCTCCGCCGCCG
CCCCTGGCTCGTCCACCCCCGCCACCGTTACCGCTGGAGAAGGAAAAATGGCATCTTCAACACCCGCCTCTCCCGCACC
TTCGGATATACTATCAAGCGAACCACAGTCAAAACGCCCTCCTGGGCGGTGGACATGATGAGATTCAATATTAATGAC
TTTCTTCCCCCAGGAGGGGGCTCAAACCCCCGCTCTGTGCCCTTTGAATACTACAGAATAAGAAAGGTTAAGGTTGAA
TTCTGGCCCTGCTCCCCGATCACCCAGGGTGACAGGGGAGTGGGCTCCAGTGCTGTTATTCTAGATGATAACTTTGTA
ACAAAGGCCACAGCCCTCACCTATGACCCCTATGTAAACTACTCCTCCCGCCATACCATAACCCAGCCCTTCTCCTACCA
CTCCCGCTACTTTACCCCCAAACCTGTCCTAGATTCCACTATTGATTACTTCCAACCAAACAACAAAAGAAATCAGCTGT
GGCTGAGACTACAAACTGCTGGAAATATAGACCACGTAGGCCTCGGCACTGCGTTCGAAAACAGTATATACGACCAG
GAATACAATATCCGTGTAACCATGTATGTACAATTCAGAGAATTTAATCTTAAAGACCCCCCACTTAACCCT--------

>HQ591375_pcv2b

ATGACGTATCCAAGGAGGCGTTACCGGAGAAGAAGACACCGCCCCCGCAGCCATCTTGGCCAGATCCTCCGCCGCCG
CCCCTGGCTCGTCCACCCCCGCCACCGTTACCGCTGGAGAAGGAAAAATGGCATCTTCAACACCCGCCTCTCCCGCACC
TTCGGATATACTATCAAGCGAACCACAGTCAAAACGCCCTCATGGGCGGTGGACATGATGAGATTCAATATTAATGAC
TTTCTTCCCCCAGGAGGGGGCTCAAACCCCCGCTCTGTGCCCTTTGAATACTACAGAATAAGAAAGGTTAAGGTTGAA
TTCTGGCCCTGCTCCCCGATCACCCAGGGTGACAGGGGAGTGGGCTCCAGTGCTGTTATTCTAGATGATAACTTTGTA
ACAAAGGCCACAGCCCTCACCTATGACCCCTATGTAAACTACTCCTCCCGCCATACCATAACCCAGCCCTTCTCCTACCA
CTCCCGCTACTTTACCCCCAAACCTGTCCTAGATTCCACTATTGATTACTTCCAACCAAACAACAAAAGAAATCAGCTGT
GGCTGAGACTACAAACTGCTGGAAATGTAGACCACGTAGGCCTCGGCACTGCGTTCGAAAACAGTATATACGACCAG
GAATACAATATCCGTGTAACCATGTATGTACAATTCAGAGAATTTAATCTTAAAGACCCCCCACTTAACCCT--------

>EF560609_pcv2b

ATGACGTATCCAAGGAGGCGTTACCGGAGAAGAAGACACCGCCCCCGCAGCCATCTTGGCCAGATCCTCCGCCGCCG
CCCCTGGCTCGTCCACCCCCGCCACCGTTACCGCTGGAGAAGGAAAAATGGCATCTTCAACACCCGCCTCTCCCGCACC
TTCGGATATACTATCAAGCGAACCACAGTCAAAACGCCCTCCTGGGCAGTGGACATGATGAGATTCAATATTAATGAC
TTTCTTCCCCCAGGAGGTGGCTCAAACCCCCGCTCTGTGCCCTTTGAATACTACAGAATAAGAAAGGTTAAGGTTGAAT
TCTGGCCCTGCTCCCCGATCACCCAGGGTGACAGGGGAGTGGGCTCCAGTGCTGTTATTCTAGATGATAACTTTATAA
CAAAGGCCACAGCCCTCACCTATGACCCCTATGTAAACTACTCCTCCCGCCATACCATAACCCAGCCCTTCTCCTACCAC
TCCCGCTACTTTACCCCCAAACCTGTCCTAGATTCCACTATTGATCACTTCCAACCAAACAACAAAAGAAATCAGCTGTG
GCTGAGACTACAAACTGCTGGAAATGTAGACCACGTAGGCCTGGGCACTGCGTTCGAAAACAGTATATACGACCAGG
AATACAATATCCGTGTAACCATGTATGTACAATTCAGAGAATTTAATCTTAAAGACCCCCCACTTAACCCT--------

>EF560608_pcv2b

ATGACGTATCCAAGGAGGCGTTACCGGAGAAGAAGACACCGCCCCCGCAGCCATCTTGGCCAGATCCTCCGCCGCCG
CCCCTGGCTCGTCCACCCCCGCCACCGTTACCGCTGGAGAAGGAAAAATGGCATCTTCAACACCCGCCTCTCCCGCACC
TTCGGATATACTATCAAGCGAACCACAGTCAAAACGCCCTCCTGGGCAGTGGACATGATGAGATTCAATATTAATGAC
TTTCTTCCCCCAGGAGGTGGCTCAAACCCCCGCTCTGTGCCCTTTGAATACTACAGAATAAGAAAGGTTAAGGTTGAAT
TCTGCCCCTGCTCCCCGATCACCCAGGGTGACAGGGGAGTGGGCTCCAGTGCTGTTATTCTAGATGATAACTTTGTAA
CAAAGGCCACAGCCCTCACCTATGACCCCTATGTAAACTACTCCTCCCGCCATACCATAACCCAGCCCTTCTCCTACCAC
TCCCGCTACTTTACCCCCAAACCTGTCCTAGATTCCACTATTGATTACTTCCAACCAAACAACAAAAGAAATCAGCTGTG
GCTGAGACTACAAACTGCTGGAAATGTAGACCACGTGGGCCTGGGCACTGCGTTCGAAAACAGTATATACGACCAGG
AATACAATATCCGTGTAACCATGTATGTACAATTCAGAGAATTTACTCTTAAAGACCCCCCACTTAACCCT--------

>EF560610_pcv2b

ATGACGTATCCAAGGAGGCGTTACCGGAGAAGAAGACACCGCCCCCGCAGCCATCTTGGCCAGATCCTCCGCCGCCG
CCCCTGGCTCGTCCACCCCCGCCACCGTTACCGCTGGAGAAGGAAAAATGGCATCTTCAACACCCGCCTCTCCCGCACC
TTCGGATATACTATCAAGCGAACCACAGTCAAAACGCCCTCCTGGGCAGTGGACATGATGAGATTCAATATTAATGAC
TTTCTTCCCCCAGGAGGTGGCTCAAACCCCCGCTCTGTGCCCTTTGAATACTACAGAATAAGAAAGGTTAAGGTTGAAT
TCTGGCCCTGCTCCCCGATCACCCAGGGTGACAGGGGAGTGGGCTCCAGTGCTGTTATTCTAGATGATAACTTTGTAA
CAAAGGCCACAGCCCTCACCTATGACCCCTATGTAAACTACTCCTCCCGCCATACCATAACCCAGCCCTTCTCCTACCAC
TCCCGCTACTTTACCCCCAAACCTGTCCTAGATTCTACTATTGATTACTTCCAACCAAACAACAAAAGAAATCAGCTGTG
GCTGAGACTACAAACTGCTGGAAATGTAGACCACCTAGGCCTGGGCACTGCGTTCGAAAACAGTATATACGACCAGG
AATACAATATCCGTGTAACCATGTATGTACAATTCAGAGAATTTAATCTTAAAGACCCCCCACTTAACCCT--------

>EF190928_pcv2b

ATGACGTATCCAAGGAGGCGTTACCGGAGAAGAAGACACCGCCCCCGCAGCCATCTTGGCCAGATCCTCCGCCGCCG
CCCCTGGCTCGTCCACCCCCGCCACCGTTACCGCTGGAGAAGGAAAAATGGCATCTTCAACACCCGCCTCTCCCGCACC
TTCGGATATACTATCAAGCGAACCACAGTCAAAACGCCCTCCTGGGCAGTGGACATGATGAGATTCAATATTAATGAC
TTTCTTCCCCCAGGAGGTGGCTCAAACCCCCGCTCTGTGCCCTTTGAATACTACAGAATAAGAAAGGTTAAGGTTGAAT
TCTGGCCCTGCTCCCCGATCACCCAGGGTGACAGGGGAGTGGGCTCCAGTGCTGTTATTCTAGATGATAACTTTGTAA
CAAAGGCCACAGCCCTCACCTATGACCCCTATGTAAACTACTCCTCCCGCCATACCATAACCCAGCCCTTCTCCTACCAC
TCCCGCTACTTTACCCCCAAACCTGTCCTAGATTCCACTATTGATTACTTCCAACCAAACAACAAAAGAAATCAGCTGTG
GCTGAGACTACAAACTGCTGGAAATGTAGACCACGTAGGCCTGGGCACTGCGTTCGAAAACAGTATATACGACCAGG
AATACAATATCCGTGTAACCATGTATGTACAATTCAGAGAATTTAATCTTAAAGACCCCCCACTTAACCCT--------

>DQ201642_pcv2b

ATGACGTATCCAAGGAGGCGTTACCGGAGAAGAAGACACCGCCCCCGCAGCCATCTTGGCCAGATCCTCCGCCGCCG
CCCCTGGCTCGTCCACCCCCGCCACCGTTACCGCTGGAGAAGGAAAAATGGCATCTTCAACACCCGCCTCTCCCGCACC
TTCGGATATACTATCAAGCGAACCACAGTCAAAACGCCCTCCTGGGCAGTGGACATGATGAGATTCAATATTAATGAC
TTTCTTCCCCCAGGAGGGGGCTCAAACCCCCGCTCTGTGCCCTTTGAATACTACAGAATAAGAAAGGTTAAGGTTGAA
TTCTGGCCCTGCTCCCCGATCACCCAGGGTGACAGGGGAGTGGGCTCCAGTGCTGTTATTCTAGATGATAACTTTGTA
ACAAAGGCCACAGCCCTCACCTATGACCCCTATGTAAACTACTCCTCCCGCCATACCATAACCCAGCCCTTCTCCTACCA
CTCCCGCTACTTTACCCCCAAACCTGTCCTAGATTCCACTATTGATTACTTCCAACCAAACAACAAAAGAAATCAGCTGT
GGCTGAGACTACAAACTGCTGGAAATGTAGACTACGTAGGCCTCGGCACTGCGTTCGAAAACAGTATATACGACCAG
GAATACAATATCCGTGTAACCATGTATGTACAATTCAGAGAATTTAATCTTAAAGACCCCCCACTTAACCCT--------

>HQ831528_pcv2b

ATGACGTATCCAAGGAGGCGTTACCGGAGAAGAAGACACCGCCCCCGCAGCCATCTTGGCCAGATCCTCCGCCGCCG
CCCCTGGCTCGTCAACCCCCGCCACCGTTACCGCTGGAGAAGGAAAAATGGCATCTTCAACACCCGCCTCTCCCGCACC
TTCGGATATACTATCAAGCGAACCACAGTCAAAACGCCCTCCTGGGCGGTGGACATGATGAGATTCAATATTAATGAC
TTTCTTCCCCCAGGAGGGGGCTCAAACCCCCGCTCTGTGCCCTTTGAATACTACAGAATAAGAAAGGTTAAGGTTGAA
TTCTGGCCCTGCTCCCCGATCACCCAGGGTGACAGGGGAGTGGGCTCCAGTGCTGTTATTCTAGATGATAACTTTGTA
CCAAAGGCCACAGCCCTCACCTATGACCCCTATGTAAACTACTCCTCCCGCCATACCATAACCCAGCCCTTCTCCTACCA
CTCCCGCTACTTTACCCCCAAACCTGTCCTAGATTCCACTATTGATTACTTCCAACCAAACAACAAAAGAAATCAGCTGT
GGCTGAGACTACAAACTACTGGAAATGTAGACCACGTAGGCCTCGGCACTGCGTTCGAAAACAGTATATACGACCAG
GAATACAATATCCGTGTAACCATGTATGTACAATTCAGAGAATTTAATCTTAAAGACCCCCCACTTAACCCT--------

>GQ995582_pcv2b

ATGACGTATCCAAGGAGGCGTTACCGGAGAAGAAGACACCGCCCCCGCAGCCATCTTGGCCAGATCCTCCGCCGCCG
CCCCTGGCTCGTCCACCCCCGCCACCGTTACCGCTGGAGAAGGAAAAATGGCATCTTCAACACCCGCCTCTCCCGCACC
TTCGGATATACTATCAAGCGAACCACAGTCAAAACGCCCTCCTGGGCCGGTGACATGATGAGATTCAATATTAATGAC
TTTCTTCCCCCAGGAGGGGGCTCAAACCCCCGCTCTGTGCCCTTTGAATACTACAGAATAAGAAAGGTTAAGGTTGAT
TCCTGGCCCTGCTCCCCGATCACCCAGGTGAACAGGGGAGTGGGCTCCAGTGCTGTAATTTTAGATGATAACTTGGTA
CCAAAGGCCACAGCCCTCACCTATGACCCCTATGTAAAATACTCCTCCCGCCATACCATAACCCAGCCCTTCTCCTACCA
CTCCCGCTACTTTACCCCCAAACCTGTCCTAGATTCCACTATTGATTACTTCCAACCAAACAACAAAAGAAATCAGCTGT
GGCTGAGACTACAAACTACTGGAAATGTAGACCACGTAGGCCTCGGCACTGCATTCGAAAACAGTATATACGACCAG
GAATACAATATCCGTGTAACCATGTATGTACAATTCAGAGAATTTAATCTTAAAGACCCCCCACTTAACCCT--------

>HQ831540_pcv2b

ATGACGTATCCAAGGAGGCGTTACCGGAGAAGAAGACACCGCCCCCGCAGCCATCTTGGCCAGATCCTCCGCCGCCG
CCCCTGGCTCGTCCACCCCCGCCACCGTTACCGCTGGAGAAGGAAAAATGGCATCTTCAACACCCGCCTCTCCCGCACC
TTCGGATATACTATCAAGCGAACCACAGTCAAAACACCCTCCTGGGCGGTGGACATGATGAGATTCAATATTAATGAC
TTTCTTCCCCCAGGAGGGGGCTCAAACCCCCGCTCTGTGCCCTTTGAATACTACAGAATAAGAAAGGTTAAGGTTGAA
TTCTGGCCCTGCTCCCCGATCACCCAGGGTGACAGGGGAGTGGGCTCCAGTGCTGTTATTCTAGATGATAACTTTGTA
CCAAAGGCCACAGCCCTCACCTATGACCCCTATGTAAACTACTCCTCCCGCCATACCATAACCCAGCCCTTCTCCTACCA
CTCCCGCTACTTTACCCCCAAACCTGTCCTAGATTCCACTATTGATTACTTCCAACCAAACAACAAAAGAAACCAGCTGT
GGCTGAGACTACAAACTACTGGAAATGTAGACCACGTAGGCCTCGGCACTGCGTTCGAAAACAGTATATACGACCAG
GAATACAATATCCGTGTAACCATGTATGTACAATTCAGAGAATTTAATCTTAAAGACCCCCCACTTAACCCT--------

>GU247991_pcv2b

ATGACGTATCCAAGGAGGCGTTACCGAAGAAGAAGACACCGCCCCCGCAGCCATCTTGGCCAGATCCTCCGCCGCCG
CCCCTGGCTCGTCCACCCCCGCCACCGTTACCGCTGGAGAAGGAAAAATGGCATCTTCAACACCCGCCTCTCCCGCACC
TTCGGATATACTATCAAGCGAACCACAGTCAAAACGCCCTCCTGGGCAGTGGACATGATGAGATTCAATATTAATGAC
TTTCTTCCCCCAGGAGGGGGCTCAAACCCCCGCTCTGTGCCCTTTGAATACTACAGAATAAGAAAGGTTAAGGTTGAA
TTCTGGCCCTGCTCCCCGATCACCCAAGGTGACAGGGGAGTGGGCTCCAGTGCTGTTATTCTAGATGATAACTTTGTA
CCAAAGGCCACAGCCCTCACCTATGACCCCTATGTAAACTACTCCTCCCGCCATACCATAACCCAGCCCTTCTCCTACCA
CTCCCGCTACTTTACCCCCAAACCTGTCCTAGATTCCACTATTGATTACTTCCAACCAAACAACAAAAGAAATCAGCTGT
GGCTGAGACTACAAACTGCTGGAAATGTAGACCACGTAGGCCTCGGCACTGCGTTCGAAAACAGTATATACGACCAG
GAATACAATATCCGTGTAACCATGTATGTACAATTCAGAGAATTTAATCTTAAAGACCCCCCACTTAACCCT--------

>EU545547_pcv2b

ATGACGTATCCAAGGAGGCGTTACCGGAGAAGAAGACACCGCCCCCGCAGCCATCTTGGCCAGATCCTCCGCCGCCG
CCCCTGGCTCGTCCACCCCCGCCACCGTTACCGCTGGAGAAGGAAAAATGGCATCTTCAACACCCGCCTCTCCCGCACC
TTCGGATATACTATCAAGCGAACCACAGTCAAAACGCCCTCCTGGGCGGTGGACATGATGAGATTCAATATTAATGAC
TTTCTTCCCCCAGGAGGGGGCTCAAACCCCCGCTCTGTGCCCTTTGAATACTACAGAATAAGAAAGGTTAAGGTTGAA
TTCTGGCCCTGCTCCCCGATCACCCAGGGTGACAGGGGAGTGGGCTCCAGTGCTGTTATTCTAGATGATAACTTTGTA
CCAAAGGCCACAGCCCTCACCTATGACCCCTATGTAAACTACTCCTCCCGCCATACCATTACCCAGCCCTTCTCCTACCA
CTCCCGCTACTTTACCCCCAAACCTGTCCTAGATTCCACTATTGATTACTTCCAACCAAACAACAAAAGAAATCAGCTGT
GGCTGAGACTACAAACTGCTGGAAATGTAGACCACGTAGGCCTCGGCACTGCGTTCGAAAACAGTATATACGACCAG
GAATACAATATCCGTGTAACCATGTATGTACAATTCAGAGAATTTAATCTTAAAGACCCCCCACTTAACCCT--------

>FJ644924_pcv2b

ATGACGTATCCAAGGAGGCGGTACCGGAGAAGAAGACACCGCCCCCGCAGCCATCTTGGCCAGATCCTCCGCCGCCG
CCCCTGGCTCGTCCACCCCCGCCACCGTTACCGCTGGAGAAGGAAAAATGGCATCTTCAACACCCGCCTCTCCCGCACC
TTCGGATATACTATCAAGCGAACCACAGTCAAAACGCCCTCCTGGGCGGTGGACATGATGAGATTCAATATTAATGAC
TTTCTTCCCCCAGGAGGGGGCTCAAACCCCCGCTCTGTGCCCTTTGAATACTACAGAATAAGAAAGGTTAAGGTTGAA
TTCTGGCCCTGCTCCCCGATCACCCAGGGTGACAGGGGAGTGGGCTCCAGTGCTGTTATTCTAGATGATAACTTTGTA
CCAAAGGCCACAGCCCTCACCTATGACCCCTATGTAAACTACTCCTCCCGCCATACCATCACCCAGCCCTTCTCCTACCA
CTCCCGCTACTTTACCCCCAAACCTGTCCTAGATTCCACTATTGATTACTTCCAACCAAACAACAAAAGAAATCAGCTGT
GGCTGAGACTACAAACTGCTGGAAATGTAGACCACGTAGGCCTCGGCACTGCGTTCGAAAACAGTATATACGACCAG
GAATACAATATCCGTGTAACCATGTATGTACAATTCAGAGAATTTAATCTTAAAGACCCCCCACTTAACCCT--------

>FJ644555_pcv2b

ATGACGTATCCAAGGAGGCGTTACCGAAGAAGAAGACACCGCCCCCGCAGCCATCTTGGCCAGATCCTCCGCCGCCG
CCCCTGGCTCGTCCACCCCCGCCACCGTTACCGCTGGAGAAGGAAAAATGGCATCTTCAACACCCGCCTCTCCCGCACC
TTCGGATATACTATCAAGCGAACCACAGTCAAAACGCCCTCCTGGGCGGTGGACATGATGAGATTCAATATTAATGAC
TTTCTTCCCCCAGGAGGGGGCTCAAACCCCCGCTCTGTGCCCTTTGAATACTACAGAATAAGAAAGGTGAAGGTTGAA
TTCTGGCCCTGCTCCCCGATCACCCAGGGTGACAGGGGAGTGGGCTCCAGTGCTGTTATTCTAGATGATAACTTTGTA
ACAAAGGCCACAGCCCTCACCTATGACCCCTATGTAAACTACTCCTCCCGCCATACCATAACCCAGCCCTTCTCCTACCA
CTCCCGCTACTTTACCCCCAAACCTGTCCTAGATTCCACTATTGATTACTTCCAACCAAACAACAAAAGAAATCAGCTGT
GGCTGAGACTACAAACTGCTGGAAATGTAGACCACGTAGGCCTCGGCACTGCGTTCGAAAACAGTATATACGACCAG
GAATACAATATCCGTGTAACCATGTATGTACAATTCAGAGAATTTAATCTTAAAGACCCCCCACTTAACCCT--------

>EF467928_pcv2b

ATGACGTATCCAAGGAGGCGTTACCGTAGACGAAGACACCGCCCCCGCAGCCATCTTGGCCAGATCCTCCGCCGCCG
CCCCTGGCTCGTCCACCCCCGCCACCGTTACCGCTGGAGAAGGAAAAATGGCATCTTCAACACCCGCCTCTCCCGCACC
TTCGGATATACTATCAAGCGAACCACAGTCAGAACGCCCTCCTGGGCGGTGGACATGATGAGATTCAATATTAATGAC
TTTCTTCCCCCAGGAGGGGGCTCAAACCCCCGCTCTGTGCCCTTTGAATACTACAGAATAAGAAAGGTAAAGGTTGAA
TTCTGGCCCTGCTCCCCGATCACCCAGGGTGACAGGGGAGTGGGCTCCAGTGCTGTTATTCTAGATGATAACTTTGTA
ACAAAGGCCACAGCCCTCACCTATGACCCCTATGTAAACTACTCCTCCCGCCATACCATAACCCAGCCCTTCTCCTACCA
CTCCCGCTACTTTACCCCCAAACCTGTCCTAGATTCCACTATTGATTACTTCCAACCAAACAACAAAAGAAATCAGCTGT
GGCTGAGACTGCAAACTGCTGGAAATGTAGACCACGTAGGCCTCGGCACTGCGTTCGAAAACAGTATATACGACCAG
GAATACAATATCCGTGTAACCATGTATGTACAATTCAGAGAATTTAATCTTAAAGACCCCCCACTTAACCCA--------

>EF064149_pcv2b

ATGACGTATCCAAGGAGGCGTTACCGTAGACGAAGACACCGCCCCCGCAGCCATCTTGGCCAGATCCTCCGCCGCCG
CCCCTGGCTCGTCCACCCCCGCCACCGTTACCGCTGGAGATGGAAAAAAGGCATCTTCAACACCCGCCTCTCCCGCACC
TTCGGATATACTATCAAGCGAACCACAGTCAAAACGCCCTCCTGGGCGGTGGACATGATGAGATTCAATATTAATGAC
TTTCTTCCCCCAGGAGGGGGCTCAAACCCCCGCTCTGTGCCCTTTGAATACTACAGAATAAGAAAGGTTAAGGTTGAA
TTCTGGCCCTGCTCCCCGATCACCCAGGGTGACAGGGGAGTGGGCTCCAGTGCTGTTATTCTAGATGATAACTTTGTA
ACAAAGGCCACAGCCCTCACCTATGACCCCTATGTAAACTACTCCTCCCGCCATACCATAACCCAGCCCTTCTCCTACCA
CTCCCGCTACTTTACCCCCAAACCTGTCCTAGATTCCACTATTGATTACTTCCAACCAAACAACAAAAGAAATCAGCTGT
GGCTGAGACTACAAACTGCTGGAAATGTAGACCACGTAGGCCTCGGCACTGCGTTCGAAAACAGTATATACGACCAG
GAATACAATATCCGTGTAACCATGTATGTACAATTCAGAGAATTTAATCTTAAAGACCCCCCACTTACACCT--------

>HQ395028_pcv2b

ATGACGTATCCAAGGAGGCGTTTCCGCAGACGAAGACACCGCCCCCGCAGCCATCTTGGCCAGATCCTCCGCCGCCGC
CCCTGGCTCGTCCACCCCCGCCACCGTTACCGCTGGAGAAGGAAAAATGGCATCTTCAACACCCGCCTCTCCCGCACCT
TCGGATATACTATCAAGCGAACCACAGTCAAAACGCCCTCCTGGGCGGTGGACATGATGAGATTCAATATTAATGACT
TTCTTCCCCCAGGAGGGGGCTCAAACCCCCGCTCTGTGCCCTTTGAATACTACAGAATAAGAAAGGTTAAGGTTGAAT
TCTGGCCCTGCTCCCCGATCACCCAGGGTGACAGGGGAGTGGGCTCCAGTGCTGTTATTCTAGATGATAACTTTGTAA
CAAAGGCCACAGCCCTCACCTATGACCCCTATGTAAACTACTCCTCCCGCCATACCATAACCCAGCCCTTCTCCTACCAC
TCCCGCTACTTTACCCCCAAACCTGTCCTAGATTCCACTATTGATTACTTCCAACCAAACAACAAAAGAAATCAGCTGTG
GCTGAGACTACAAACTACTGGAAATGTAGACCACGTAGGCCTCGGCACTGCGTTCGAAAACAGTATATACGACCAGG
AATACAATATCCGTGTAACCATGTATGTACAATTCAGAGAATTTAATCTTAAAGACCCCCCACTTAACCCT--------

>HQ395046_pcv2b

ATGACGTATCCAAGGAGGCGTTTCCGCAGACGAAGACACCGCCCCCGCAGCCATCTTGGCCAGATCCTCCGACGCCG
CCCCTGGCTCGTCCACCCCCGCCACCGTTACCGCTGGAGAAGGAAAAATGGCATCTTCAACACCCGCCTCTCCCGCACC
TTCGGATATACTATCAAGCGAACCACAGTCAAAACGCCCTCCTGGGCGGTGGACATGATGAGATTCAATATTAATGAC
TTTCTTCCCCCAGGAGGGGGCTCAAACCCCCGCTCTGTGCCCTTTGAATACTACAGAATAAGAAAGGTTAAGGTTGAA
TTCTGGCCCTGCTCCCCGATCACCCAGGGTGACAGGGGAGTGGGCTCCAGTGCTGTTATTCTAGATGATAACTTTGTA
ACAAAGGCCACAGCCCTCACCTATGACCCCTATGTAAACTACTCCTCCCGCCATACCATAACCCAGCCCTTCTCCTACCA
CTCCCGCTACTTTACCCCCAAACCTGTCCTAGATTCCACTATTGATTACTTCCAACCAAACAACAAAAGAAATCAGCTGT
GGCTGAGACTACAAACTACTGGAAATGTAGACCACGTAGGCCTCGGCACTGCGTTCGAAAACAGTATATACGACCAG
GAATACAATATCCGTGTAACCATGTATGTACAATTCAGAGAATTTAATCTTAAAGACCCCCCACTTAACCCT--------

>JN382168_pcv2b

ATGACGTATCCAAGGAGGCGTTACCGCAGACGAAGACACCGCCCCCGCAGCCATCTTGGCCAGATCCTCCGCCGCCG
CCCCTGGCTCGTCCACCCCCGCCACCGTTACCGCTGGAGAAGGAAAAATGGCATCTTCAACACCCGCCTCTCCCGCACC
TTCGGATATACTATCAAGCGAACCACAGTCAAAACGCCCTCCTGGGCGGTGGACATGATGAGATTCAATATTAATGAC
TTTCTTCCCCCAGGAGGGGGCTCAAACCCCCGCTCTGTGCCCTTTGAATACTACAGAATAAGAAAGGTTAAGGTTGAA
TTCTGGCCCTGCTCCCCGATCACCCAGGGTGACAGGGGAGTGGGCTCCAGTGCTGTTATTCTAGATGATAACTTTGTA
CCAAAGGCCACAGCCCTCACCTATGACCCCTATGTAAACTACTCCTCCCGCCATACCATAACCCAGCCCCTCTCCTACCA
CTCCCGCTACTTTACCCCCAAACCTGTCCTAGATTCCACTATTGATTACTTCCAACCAAACAACAAAAGAAATCAGCTGT
GGATGAGACTACAAACTGCTGGAAATGTAGACCACGTAGGCCTCGGCACTGCGTTCGAAAACAGTATATACGACCAG
GAATACAATATCCGTGTAACCATGTATGTACAATTCAGAGAATTTAATCTTAAAGACCCCCCACTTAACCCT--------

>JN382169_pcv2b

ATGACGTATCCAAGGAGGCGTTACCGCAGACGAAGACACCGCCCCCGCAGCCATCTTGGCCAGATCCTCCGCCGCCG
CCCCTGGCTCGTCCACCCCCGCCACCGTTACCGCTGGAGAAGGAAAAATGGCATCTTCAACACCCGCCTCTCCCGCACC
TTCGGATATACTATCAAGCGAACCACAGTCAAAACGCCCTCCTGGGCGGTGGACATGATGAGATTCAATATTAATGAC
TTTCTTCCCCCAGGAGGGGGCTCAAACCCCCGCTCTGTGCCCTTTGAATACTACAGAATAAGAAAGGTTAAGGTTGAA
TTCTGGCCCTGCTCCCCGATCACCCAGGGTGACAGGGGAGTGGGCTCCAGTGCTGTTATTCTAGATGATAACTTTGTA
CCAAAGGCCACAGCCCTCACCTATGACCCCTATGTAAACTACTCCTCCCGCCATACCATAACCCAGCCCCTCTCCTACCA
CTCCCGCTACTTTACCCCCAAACCTGTCCTAGATTCCACTATTGATTACTTCCAACCAAACAACAAAAGAAATCAGCTGT
GGATGAGACTACAAACTGCTGGAAATGTAGACCACGTAGGCCTCGGCACTGCGTTCGAAAACAGTATATACGACCAG
GAATACAATATCCGTGTAACCATGTATGTACAATTCAGAGAATTTAATCTTAAAGACCCCCCACTTAACCCT--------

>AY424405_pcv2b

ATGACGTATCCAAGGAGGCGTTACCGGAGAAGAAGACACCGCCCCCGCAGCCATCTTGGCCAGATCCTCCGCCGCCG
CCCCTGGCTCGTCCACCCCCGCCACCGTTACCGCTGGAGAAGGAAAAATGGCATCTTCAACACCCGCCTCTCCCGCACC
TTCGGATATACTATCAAGCGAACCACAGTCAAAACGCCCTCCTGGGCGGTGGACATGATGAGATTCAATATTAATGAC
TTTCTTCCCCCAGGAGGGGGCTCAAACCCCCGCTCTGTGCCCTTTGAATACTACAGAATAAGAAAGGTTAAGGTTGAA
TTCTGGCCCTGCTCCCCGATCACCCAGGGTGACAGGGGAGTGGGCTCCAGTGCTGTTATTCTAGATGATAACTTTGTA
ACAAAGGCCACAGCCCTCACCTATGACCCCTATGTAAACTACTCCTCCCGCCATACCATAACCCAGCCCTTCTCCTACCA
CTCCCGCTACTTTACCCCCAAACCTGTCCTAGATTCCACTATTGATTACTTCCAACCAAACAACAAAAGAAATCAGCTGT
GGCTGAGACTACAAACTGCTGGAAATGTAGACCACGTAGGCCTCGGCACTGCGTTCGAAAACAGTATATACGACCAG
GAATACAATATCCGTGTAACCATGTATGTACAATTCAGAGAATTTAATCTTAAAGACCCCCCACTTAACCCT--------

>EU136715_pcv2b

ATGACGTATCCAAGGAGGCGTTACCGGAGAAGAAGACACCGCCCCCGCAGCCATCTTGGCCAGATCCTCCGCCGCCG
CCCCTGGCTCGTCCACCCCCGCCACCGTTACCGCTGGAGAAGGAAAAATGGCATCTTCAACACCCGCCTCTCCCGCACC
TTCGGATATACTATCAAGCGAACCACAGTCAAAACGCCCTCCTGGGCGGTGGACATGATGAGATTCAATATTAATGAC
TTTCTTCCCCCAGGAGGGGGCTCAAACCCCCGCTCTGTGCCCTTTGAATACTACAGAATAAGAAAGGTTAAGGTTGAA
TTCTGGCCCTGCTCCCCGATCACCCAGGGTGACAGGGGAGTGGGCTCCAGTGCTGTTATTCTAGATGATAACTTTGTA
ACAAAGGCCACAGCCCTCACCTATGACCCCTATGTAAACTACTCCTCCCGCCATACCATAACCCAGCCCTTCTCCTACCA
CTCCCGCTACTTTACCCCCAAACCTGTCCTAGATTCCACTATTGATTACTTCCAACCAAACAACAAAAGAAATCAGCTGT
GGCTGAGACTACAAACTGCTGGAAATGTAGACCACGTAGGCCTCGGCACTGCGTTCGAAAACAGTATATACGACCAG
GAATACAATATCCGTGTAACCATGTATGTACAATTCAGAGAATTTAATCTTAAAGACCCCCCACTTAACCCT--------

>AY484416_pcv2b

ATGACGTATCCAAGGAGGCGTTACCGGAGAAGAAGACACCGCCCCCGCAGCCATCTTGGCCAGATCCTCCGCCGCCG
CCCCTGGCTCGTCCACCCCCGCCACCGTTACCGCTGGAGAAGGAAAAATGGCATCTTCAACACCCGCCTCTCCCGCACC
TTCGGATATACTATCAAGCGAACCACAGTCAAAACGCCCTCCTGGGCGGTGGACATGATGAGATTCAATATTAATGAC
TTTCTTCCCCCAGGAGGGGGCTCAAACCCCCGCTCTGTGCCCTTTGAATACTACAGAATAAGAAAGGTTAAGGTTGAA
TTCTGGCCCTGCTCCCCGATCACCCAGGGTGACAGGGGAGTGGGCTCCAGTGCTGTTATTCTAGATGATAACTTTGTA
ACAAAGGCCACAGCCCTCACCTATGACCCCTATGTAAACTACTCCTCCCGCCATACCATAACCCAGCCCTTCTCCTACCA
CTCCCGCTACTTTACCCCCAAACCTGTCCTAGATTCCACTATTGATTACTTCCAACCAAACAACAAAAGAAATCAGCTGT
GGCTGAGACTACAAACTGCTGGAAATGTAGACCACGTAGGCCTCGGCACTGCGTTCGAAAACAGTATATACGACCAG
GAATACAATATCCGTGTAACCATGTATGTACAATTCAGAGAATTTAATCTTAAAGACCCCCCACTTAACCCT--------

>AY424404_pcv2b

ATGACGTATCCAAGGAGGCGTTACCGGAGAAGAAGACACCGCCCCCGCAGCCATCTTGGCCAGATCCTCCGCCGCCG
CCCCTGGCTCGTCCACCCCCGCCACCGTTACCGCTGGAGAAGGAAAAATGGCATCTTCAACACCCGCCTCTCCCGCACC
TTCGGATATACTATCAAGCGAACCACAGTCAAAACGCCCTCCTGGGCGGTGGACATGATGAGATTCAATATTAATGAC
TTTCTTCCCCCAGAGGGGGCGTCAAACCCCCGCTCTGTGCCCTTTGAATACTACAGAATAAGAAAGGTTAAGGTTGAA
TTCTGGCCCTGCTCCCCGATCACCCAGGGTGACAGGGGAGTGGGCTCCAGTGCTGTTATTCTAGATGATAACTTTGTA
ACAAAGGCCACAGCCCTCACCTATGACCCCTATGTAAACTACTCCTCCCGCCATACCATAACCCAGCCCTTCTCCTACCA
CTCCCGTTACTTTACCCCCAAACCTGTCCTAGATTCCACTATTGATTACTTCCAACCAAACAACAAAAGAAATCAGCTGT
GGCTGAGACTACAAACTGCTGGAAATGTAGACCACGTAGGCCTCGGCACTGCGTTCGAAAACAGTATATACGACCAG
GAATACAATATCCGTGTAACCATGTATGTACAATTCAGAGAATTTAATCTTAAAGACCCCCCACTTAACCCT--------

>EF190932_pcv2b

ATGACGTATCCAAGGAGGCGTTACCGGAGAAGAAGACACCGCCCCCGCAGCCATCTTGGCCAGATCCTCCGCCGCCG
CCCCTGGCTCGTCCACCCCCGCCACCGTTACCGCTGGAGAAGGAAAAATGGCATCTTCAACACCCGCCTCTCCCGCACC
TTCGGATATACTATCAAGCGAACCACAGTCAAAACGCCCTCCTGGGCGGTGGACATGATGAGATTCAATATTAATGAC
TTTCTTCCCCCAGGGGGGGGCTCAAACCCCCGCTCTGTGCCCTTTGAATACTACAGAATAAGAAAGGTTAAGGTTGAA
TTCTGGCCCTGCTCCCCGATCACCCAGGGTGACAGGGGAGTGGGCTCCAGTGCTGTTATTCTAGATGATAACTTTGTA
ACAAAGGCCACAGCCCTCACCTATGACCCCTATGTAAACTACTCCTCCCGCCATACCATAACCCAGCCCTTCTCCTACCA
CTCCCGCTACTTTACCCCCAAACCTGTCCTAGATTCGACTATTGATTACTTCCAACCAAACAACAAAAGAAATCAGCTGT
GGCTGAGACTACAAACTGCTGGAAATGTAGACCACGTAGGCCTCGGCACTGCGTTCGAAAACAGTATATACGACCAG
GAATACAATATCCGTGTAACCATGTATGTACAATTCAGAGAATTTAATCTTAAAGACCCCCCACTTAACCCT--------

>JQ866918_pcv2b

ATGACGTATCCAAGGAGGCGTTACCGGAGAAGAAGACACCGCCCCCGCAGCCATCTTGGCCAGATCCTCCGCCGCCG
CCCCTGGCTCGTCCACCCCCGCCACCGTTACCGCTGGAGAAGGAAAAATGGCATCTTCAACACCCGCCTCTCCCGCACC
TTCGGATATACTATCAAGCGAACCACAGTCAAAACGCCCTCCTGGGCGGTGGACATGATGAGATTCAATATTAATGAC
TTTCTTCCCCCAGGAGGGGGCTCAAACCCCCGCTCTGTGCCCTTTGAATACTACAGAATAAGAAAGGTTAAGGTTGAA
TTCTGGCCCTGCTCCCCGATCACCCAGGGTGACAGGGGAGTGGGCTCCAGTGCTGTTATTCTAGATGATAACTTTGTA
ACAAAGGCCACAGCCCTCACCTATGACCCCTATGTAAACTACTCCTCCCGCCATACCATAACCCAGCCCTTCTCCTACCA
CTCCCGCTACTTTACCCCCAAACCTGTCCTAGATTCCACTATTGATTACTTCCAACCAAACAACAAAAGAAATCAGCTGT
GGCTGAGACTACAAACTGCTGGAAATGTAGACCACGTAGGCCTCGGCACTGCGTTCGAAAACAGTATATACGACCAG
GAATACAATATCCGTGTAACCATGTACGTACAATTCAGAGAATTTAATCTTAAAGACCCCCCACTTAACCCT--------

>JQ866919_pcv2b

ATGACGAAATCAGGGAGGCGTTACCGGAGAAGAAGACACCGCCCCCGCAGCCATCTTGGCCAGATCCTCCGCCGCCG
CCCCTGGCTCGTCCACCCCCGCCACCGTTACCGCTGGAGAAGGAAAAATGGCATCTTCAACACCCGCCTCTCCCGCACC
TTCGGATATACTATCAAGCGAACCACAGTCAAAACGCCCTCCTGGGCGGTGGACATGATGAGATTCAATATTAATGAC
TTTCTTCCCCCAGGAGGGGGCTCAAACCCCCGCTCTGTGCCCTTTGAATACTACAGAATAAGAAAGGTTAAGGTTGAA
TTCTGGCCCTGCTCCCCGATCACCCAGGGTGACAGGGGAGTGGGCTCCAGTGCTGTTATTCTAGATGATAACTTTGTA
ACAAAGGCCACAGCCCTCACCTATGACCCCTATGTAAACTACTCCTCCCGCCATACCATAACCCAGCCCTTCTCCTACCA
CTCCCGCTACTTTACCCCCAAACCTGTCCTAGATTCCACTATTGATTACTTCCAACCAAACAACAAAAGAAATCAGCTGT
GGCTGAGACTACAAACTGCTGGAAATGTAGACCACGTAGGCCTCGGCACTGCGTTCGAAAACAGTATATACGACCAG
GAATACAATATCCGTGTAACCATGTACGTACAATTCAGAGAATTTAATCTTAAAGACCCCCCACTTAACCCC--------

>EF190925_pcv2b

ATGACGTATCCAAGGAGGCGTTACCGGAGAAGAAGACACCGCCCCCGCAGCCATCTTGGCCAAATCCTCCGCCGCCG
CCCCTGGCTCCTCCACCCCCGCCACCGTTACCGCTGGAGAAGGAAAAATGGCATCTTCAACACCCGCCTCTCCCGCACC
TTCGGATATACTATCAAGCGAACCACAGTCAAAACGCCCTCCTGGGCGGTGGACATGATGAGATTCAATATTAATGAC
TTTCTTCCCCCAGGAGGGGGCTCAAACCCCCGCTCTGTGCCCTTTGAATACTACAGAATAAGAAAGGTTAAGGTTGAA
TTCTGGCCCTGCTCCCCGATCACCCAGGGTGACAGGGGAGTGGGCTCCAGTGCTGTTATTCTAGATGATAACTTTGTA
ACAAAGGCCACAGCCCTCACCTATGACCCCTATGTAAACTACTCCTCCCGCCATACCATAACCCAGCCCTTCTCCTACCA
CTCCCGCTACTTTACCCCCAAACCTGTCCTAGATTCCACTATTGATTACTTCCAACCAAACAACAAAAGAAATCAGCTGT
GGCTGAGACTACAAACTGCTGGAAATGTAGACCACGTAGGCCTCGGCACTGCGTTCGAAAACAGTATATACGACCAG
GAATACAATATCCGTGTAACCATGTACGTACAATTCAGAGAATTTAATCTTAAAGACCCCCCACTTAACCCT--------

>EU684164_pcv2b

ATGACGTATCCAAGGAGGCGTTACCGGAGAAGAAGACACCGCCCCCGCAGCCATCTTGGCCAAATCCTCCGCCGCCG
CCCCTGGCTCGTCCACCCCCGCCACCGTTACCGCTGGAGAAGGAAAAATGGCATCTTCAATACCCGCCTCTCCCGCACC
TTCGGATATACTATCAAGCGAACCACAGTCAAAACGCCCTCCTGGGCGGTGGACATGATGAGATTCAATATTAATGAC
TTTCTTCCCCCAGGAGGGGGCTCAAACCCCCGCTCTGTGCCCTTTGAATACTACAGAATAAGAAAGGTTAAGGTTGAA
TTCTGGCCCTGCTCCCCGATCACCCAGGGTGACAGGGGAGTGGGCTCCAGTGCTGTTATTCTAGATGATAACTTTGTA
ACAAAGGCCACAGCCCTCACCTATGACCCCTATGTAAACTACTCCTCCCGCCATACCATAACCCAGCCCTTCTCCTACCA
CTCCCGCTACTTTACCCCCAAACCTGTCCTAGATTCCACTATTGATTACTTCCAACCAAACAACAAAAGAAATCAGCTGT
GGCTGAGACTACAAACTGCTGGAAATGTAGACCACGTAGGCCTCGGCACTGCGTTCGAAAACAGTATATACGACCAG
GAATACAATATCCGTGTAACCATGTATGTACAATTCAGAGAATTTAATCTTAAAGACCCCCCACTTAACCCT--------

>EU886637_pcv2b

ATGACGTATCCAAGGAGGCGTTACCGGAGAAGAAGACACCGCCCCCGCAGCCATCTTGGCCAGATCCTCCGCCGCCG
CCCCTGGCTCGTCCACCCCCGCCACCGTTACCGCTGGAGAAGGAAAAATGGCATCTTCAATACCCGCCTCTCCCGCACC
TTCGGATATACTATCAAGCGAACCACAGTCAAAACGCCCTCCTGGGCGGTGGACATGATGAGATTCAATATTAATGAC
TTTCTTCCCCCAGGAGGGGGCTCAAACCCCCGCTCTGTGCCCTTTGAATACTACAGAATAAGAAAGGTTAAGGTTGAA
TTCTGGCCCTGCTCCCCGATCACCCAGGGTGACAGGGGAGTGGGCTCCAGTGCTGTTATTCTAGATGATAACTTTGTA
ACAAAGGCCACAGCCCTCACCTATGACCCCTATGTAAACTACTCCTCCCGCCATACCATAACCCAGCCCTTCTCCTACCA
CTCCCGCTACTTTACCCCCAAACCTGTCCTAGATTCCACTATTGATTACTTCCAACCAAACAACAAAAGAAATCAGCTGT
GGCTGAGACTACAAACTGCTGGAAATGTAGACCACGTAGGCCTCGGCACTGCGTTCGAAAACAGTATATACGACCAG
GAATACAATATCCGTGTAACCATGTATGTACAATTCAGAGAATTTAATCTTAAAGACCCCCCACTTAACCCT--------

>EF190930_pcv2b

ATGACGTATCCAAGGAGGCGTTACCGGAGAAGAGGACACCGCCCCCGCAGCCATCTTGGCCAGATCCTCCGCCGCGG
CCCCTGGCTCCTCCACCCCCGCCACCGTTACCGCTGGAGAAGGAAAAATGGCATCTTCAACACCCGCTTCTCCCGCACC
TTCGGATATACTATCAAGCGAACCACAGTCAAAACGCCCTCCTGGGCGGTGGACATGATGAGATTCAATATTAATGAC
TTTCTTCCCCCAGGAGGGGGCTCAAACCCCCGCTCTGTGCCCTTTGAATACTACAGAATAAGAAAGGTTAAGGTTGAA
TTCTGGCCCTGCTCCCCGATCACCCAGGGTGACAGGGGAGTGGGCTCCAGTGCTGTTATTCTAGATGATAACTTTGTA
ACAAAGGCCACAGCCCTCACCTATGACCCCTATGTAAACTACTCCTCCCGCCATACCATAACCCAGCCCTTCTCCTACCA
CTCCCGTTACTTTACCCCCAAACCTGTCCTAGATTCCACTATTGATTACTTCCAACCAAACAACAAAAGAAATCAGCTGT
GGCTGAGACTACAAACTGCTGGAAATGTAGACCACGTAGGCCTCGGCACTGCGTTCGAAAACAGTATATACGACCAG
GAATACAATATCCGTGTAACCATGTATGTACAATTCAGAGAATTTAATCTTAAAGACCCCCCACTTAACCCT--------

>JF927983_pcv2b

ATGACGTATCCAAGGAGGCGTTACCGCAGAAGAGAACACCGCCCCCGCAGCCATCTTGGCCAGATCCTCCGCCGCCG
CCCCTGGCTCGTCCACCCCCGCCACCGTTACCGCTGGAGAAGGAAAAATGGCATCTTCAACACCCGCCTCTCCCGCACC
TTCGGATATACTATCAAGCGAACCACAGTCAAAACGCCCTCCTGGGCGGTGGACATGATGAGATTCAATATTAATGAC
TTTCTTCCCCCAGGAGGGGGCTCAAACCCCCGCTCTGTGCCCTTTGAATACTACAGAATAAGAAAGGTTAAGGTTGAA
TTCTGGCCCTGCTCCCCGATCACCCAGGGTGACAGGGGAGTGGGCTCCAGTGCTGTTATTCTAGATGATAACTTTGTA
ACAAAGGCCACAGCCCTCACCTATGACCCCTATGTAAACTACTCCTCCCGCCATACCATAACCCAGCCCTTCTCCTACCA
CTCCCGCTACTTTACCCCCAAACCTGTCCTAGATTCCACTATTGATTACTTCCAACCAAACAACAAAAGAAATCAGCTGT
GGCTGAGACTACAAACTGCTGGAAATGTAGACCACGTAGGCCTCGGCACTGCGTTCGAAAACAGTATATACGACCAG
GAATACAATATCCGTGTAACCATGTATGTACAATTCAGAGAATTTAATCTTAAAGACCCCCCACTTAACCCT--------

>GU325764_pcv2b

ATGACGTATCCAAGGAGGCGTTACCGGAGAAGAAGACACCGCCCCCGCAGCCATCTTGGACAGATCCTCAGCCGCCG
CCCCTGGCTCGTCCACCCCCGCCACCGTTACCGCTGGAGACGGAAAAATGGCATCTTCAACACCCGCCTCTCCCGCACC
TTCGGATATACTATCAAGCGAACCACAGTCAAAACGCCCTCCTGGGCGGTGGACATGATGAGATTCAATATTAATGAC
TTTCTTCCCCCAGGAGGGGGCTCAAACCCCCGCTCTGTGCCCTTTGAATACTACAGAATAAGAAAGGTTAAGGTTGAA
TTCTGGCCCTGCTCCCCGATCACCCAGGGTGACAGGGGAGTGGGCTCCAGTGCTGTTATTCTAGATGATAACTTTGTA
ACAAAGGCCACAGCCCTCACCTATGACCCCTATGTAAACTACTCCTCCCGCCATACCATAACCCAGCCCTTCTCCTACCA
CTCCCGCTACTTTACCCCCAAACCTGTCCTAGATTCCACTATTGATTACTTCCAACCAAACAACAAAAGAAATCAGCTGT
GGCTAAGACTACAAACTGCTGGAAATGTAGACCACGTAGGCCTCGGCACTGCGTTCGAAAACAGTATATACGACCAG
GAATACAATATCCGTGTAACCATGTATGTACAATTCAGAGAATTTAATCTTAAAGACCCCCCACTTAACCCT--------

>EF371548_pcv2b

ATGACGTATCCAAGGAGGCGTTACCGGAGAAGAAGACACCGCCCCCGCAGCCATCTTGGCCAGATCCTCCGCCGCCG
CCCCTGGCTCGTCCACCCCCGCCACCGTTACCGCTGGAGAAGGAAAAATGGCATCTTCAACACCCGCCTCTCCCGCACC
TTCGGATATACTATCAAGCGAACCACAGTCAAAACGCCCTCCTGGGCGGTGGACATGATGAGATTCAATATTAATGAC
TTTCTTCCCCCAGGAGGGGGCTCAAACCCCCGCTCTGTGCCCTTTGAATACTACAGAATAAGAAAGGTTAAGGTTGAA
TTCTGGCCCTGCTCCCCGATCACCCAGGGTGACAGGGGAGTGGGCTCCAGTGCTGTTATTCTAGATGATAACTTTGTA
ACAAAGGCCACAGCCCTCACCTATGACCCCTATGTAAACTACTCCTCCCGCCATACCATAACCCAGCCCTTCTCCTACCA
CTCCCGCTACTTTACCCCCAAACCTGTCCTAGATTCCACTATTGATTACTTCCAACCAAACAACAAAAGAAATCAGCTGT
GGCTAAGACTACAAACTGCTGGAAATATAGACCACGTAGGCCTCGGCACTGCGTTCGAAAACAGTATATACGACCAG
GAATACAATATCCGTGTAACCATGTATGTACAATTCAGAGAATTTAATCTTAAAGACCCCCCACTTAACCCT--------

>FJ644561_pcv2b

ATGACGTATCCAAGGAGGCGTTACCGGAGAAGAAGACACCGCCCCCGCAGCCATCTTGGCCAGATCCTCCGCCGCCG
CCCCTGGCTCGTCCACCCCCGCCACCGTTACCGCTGGAGAAGGAAAAATGGCATCTTCAACACCCGCCTCTCCCGCACC
TTCGGATATACTATCAAGCGAACCACAGTCAAAACGCCCTCCTGGGCGGTGGACATGATGAGATTCAATATTAATGAC
TTTCTTCCCCCAGGAGGGGGCTCAAACCCCCGCTCTGTGCCCTTTGAATACTACAGAATAAGAAAGGTTAAGGTTGAA
TTCTGGCCCTGCTCCCCGATCACCCAGGGTGACAGGGGAGTGGGCTCCAGTGCTGTTATTCTAGATGATAACTTTGTA
ACAAAGGCCACAGCCCTCACCTATGACCCCTATGTAAACTACTCCTCCCGCCATACCATAACCCAGCCCTTCTCCTACCA
CTCCCGCTACTTTACCCCCAAACCTGTCCTAGATTCCACTATTGATTACTTCCAACCAAACAACAAAAGAAATCAGCTGT
GGCTAAGACTACAAACTGCTGGAAATGTAGACCACGTAGGCCTCGGCACTGCGTTCGAAAACAGTATATACGACCAG
GAATACAATATCCGTGTAACCATGTATGTACAATTCAGAGAATTTAATCTTAAAGACCCCCCACTTAACCCT--------

>AB462389_pcv2b

ATGACGTATCCAAGGAGGCGTTACCGGAGAAGAAGACACCGCCCCCGCAGCCATCTTGGCCAGATCCTCCGCCGCCG
CCCCTGGCTCGTCCACCCCCGCCACCGTTACCGCTGGAGAAGGAAAAATGGCATCTTCAACACCCGCCTCTCCCGCACC
TTCGGATATACTATCAAGCGAACCACAGTCAAAACGCCCTCCTGGGCGGTGGACATGATGAGATTCAATATTAATGAC
TTTCTTCCCCCAGGAGGGGGCTCAAACCCCCGCTCTGTGCCCTTTGAATACTACAGAATAAGAAAGGTTAAGGTTGAA
TTCTGGCCCTGCTCCCCGATCACCCAGGGTGACAGGGGAGTGGGCTCCAGTGCTGTTATTCTAGATGATAACTTTGTA
ACAAAGGCCACAGCCCTCACCTATGACCCCTATGTAAACTACTCCTCCCGCCATACCATAACCCAGCCCTTCTCCTACCA
CTCCCGCTACTTTACCCCCAAACCTGTCCTAGATTCCACTATTGATTACTTCCAACCAAACAACAAAAGAAATCAGCTGT
GGCTAAGACTACAAACTGCTGGAAATGTAGACCACGTAGGCCTCGGCACTGCGTTCGAAAACAGTATATACGACCAG
GAATACAATATCCGTGTAACCATGTATGTACAATTCAGAGAATTTAATCTTAAAGACCCCCCACTTAAACCT--------

>JF928005_pcv2b

ATGACGTATCCAAGGAGGCGTTACCGGAGAAGAAGACACCGCCCCCGCAGCCATCTTGGCCAGATCCTCCGCCGCCG
CCCCTGGCTCCTCCACCCCCGCCACCGTTACCGCTGGAGAAGGAAAAATGGCATCTTCAACACCCGCCTCTCCCGCACC
TTCGGATATACTATCAAGCGAACCACAGTCAAAACGCCCTCCTGGGCGGTGGACATGATGAGATTCAATATTAATGAC
TTTCTTCCCCCAGGAGGGGGCTCAAACCCCCGCTCTGTGCCCTTTGAATACTACAGAATAAGAAAGGTTAAGGTTGAA
TTCTGGCCCTGCTCCCCGATCACCCAGGGTGACAGGGGAGTGGGCTCCAGTGCTGTTATTCTAGATGATAACTTTGTA
ACAAAGGCCACAGCCCTCACCTATGACCCCTATGTAAACTACTCCTCCCGCCATACCATAACCCAGCCCTTCTCCTACCA
CTCCCGCTACTTTACCCCCAAACCTGTCCTAGATTCCACTATTGATTACTTCCAACCAAACAACAAAAGAAATCAGCTGT
GGCTAAGACTACAAACTGCTGGAAATGTAGACCACGTAGGCCTCGGCACTGCGTTCGAAAACAGTATATACGACCAG
GAATACAATATCCGTGTAACCATGTATGTACAATTCAGAGAATTTAATCTTAAAGACCCCCCACTTAACCCT--------

>HM776446_pcv2b

ATGACGTATCCAAGGAGGCGTTACCGGAGAAGAAGACACCGCCCCCGCAGCCATCTTGGCCAGATCCTCCGCCGCCG
CCCCTGGCTCCTCCACCCCCGCCACCGTTACCGCTGGAGAAGGAAAAATGGCATCTTCAACACCCGCCTCTCCCGCACC
TTCGGATATACTATCAAGCGAACCACAGTCAAAACGCCCTCCTGGGCGGTGGACATGATGAGATTCAATATTAATGAC
TTTCTTCCCCCAGGAGGGGGCTCAAACCCCCGCTCTGTGCCCTTTGAATACTACAGAATAAGAAAGGTTAAGGTTGAA
TTCTGGCCCTGCTCCCCGATCACCCAGGGTGACAGGGGAGTGGGCTCCAGTGCTGTTATTCTAGATGATAACTTTGTA
ACAAAGGCCACAGCCCTCACCTATGACCCCTATGTAAACTACTCCTCCCGCCATACCATAACCCAGCCCTTCTCCTACCA
CTCCCGCTACTTTACCCCCAAACCTGTCCTAGATTCCACTATTGATTACTTCCGACCAAACAACAAAAGAAATCAGCTGT
GGCTGAGACTACAAACTGCTGGAAATGTAGACCACGTAGGCCTCGGCACTGCGTTCGAAAACAGTATATACGACCAG
GAATACAATATCCGTGTAACCATGTATGTACAATTCAGAGAATTTAATCTTAAAGACCCCCCACTTAACCCT--------

>AY651850_pcv2b

ATGACGTATCCAAGGAGGCGTTACCGGAGAAGAAGACACCGCCCCCGCAGCCATCTTGGCCAGATCCTCCGCCGCCG
CCCCTGGCTCCTCCACCCCCGCCACCGTTACCGCTGGAGAAGGAAAAATGGCATCTTCAACACCCGCCTCTCCCGCACC
TTCGGATATACTATCAAGCGAACCACAGTCAAAACGCCCTCCTGGGCGGTGGACATGATGAGATTCAATATTAATGAC
TTTCTTCCCCCAGGAGGGGGCTCAAACCCCCGCTCTGTGCCCTTTGAATACTACAGAATAAGAAAGGTTAAGGTTGAA
TTCTGGCCCTGCTCCCCGATCACCCAGGGTGACAGGGGAGTGGGCTCCAGTGCTGTTATTCTAGATGATAACTTTGTA
ACAAAGGCCACAGCCCTCACCTATGACCCCTATGTAAACTACTCCTCCCGCCATACCATAACCCAGCCCTTCTCCTACCA
CTCCCGCTACTTTACCCCCAAACCTGTCCTAGATTCCACTATTGATTACTTCCGACCAAACAACAAAAGAAATCAGCTGT
GGCTGAGACTACAAACTGCTGGAAATGTAGACCACGTAGGCCTCGGCACTGCGTTCGAAAACAGTATATACGACCAG
GAATACAATATCCGTGTAACCATGTATGTACAATTCAGAGAATTTAATCTTAAAGACCCCCCACTTAACCCT--------

>AB361585_pcv2b

ATGACGTATCCAAGGAGGCGTTACCGGAGAAGAAGACACCGCCCCCGCAGCCATCTTGGCCAGATCCTCCGCCGCCG
CCCCTGGCTCGTCCACCCCCGCCACCGTTACCGCTGGAGAAGGAAAAATGGCATCTTCAACACCCGCCTCTCCCGCACC
TTCGGATATACTATCAAGCGAACCACAGTCAAAACGCCCTCCTGGGCGGTGGACATGATGAGATTCAATATTAATGAC
TTTCTTCCCCCAGGAGGGGGCTCAAACCCCCGCTCTGTGCCCTTTGAATACTACAGAATAAGAAAGGTTAAGGTTGAA
TTCTGGCCCTGCTCCCCGATCACCCAGGGTGACAGGGGAGTGGGCTCCAGTGCTGTTATTCTAGATGATAACTTTGTA
ACAAAGGCCACAGCCCTCACCTATGACCCCTATGTAAACTACTCCTCCCGCCATACCATAACCCAGCCCTTCTCCTACCA
CTCCCGCTACTTTACCCCCGAACCTGTCCTAGATTCCACTATTGATTACTTCCGACCAAACAACAAAAGAAATCAGCTGT
GGCTGAGACTACAAACTGCTGGAAATGTAGACCACGTAGGCCTCGGCACTGCGTTCGAAAACAGTATATACGACCAG
GAATACAATATCCGTGTAACCATGTATGTACAATTCAGAGAATTTAATCTTAAAGACCCCCCACTTAACCCT--------

>FJ644920_pcv2b

ATGACGTATCCAAGGAGGCGTTACCGGAGAAGAAGACACCGCCCCCGCAGCCATCTTGGCCAGATCCTCCGCCGCCG
CCCCTGGCTCCTCCACCCCCGCCACCGTTACCGCTGGAGAAGGAAAAATGGCATCTTCAACACCCGCCTCTCCCGCACC
TTCGGATATACTATCAAGCGAACCACAGTCAAAACGCCCTCCTGGGCGGTGGACATGATGCGATTCAATATTAATGAC
TTTCTTCCCCCAGGAGGGGGCTCAAACCCCCGCTCTGTGCCCTTTGGATACTACAGAATAAGAAAGGTTAAGGTTGAA
TTCTGGCCCTGCTCCCCGATCACCCAGGGTGACAGGGGAGTGGGCTCCAGTGCTGTTATTCTAGATGATAACTTTGTA
ACAAAGGCCACAGCCCTCACCTATGACCCCTATGTAAACTACTCCTCCCGCCATACCATAACCCAGCCCTTCTCCTACCA
CTCCCGCTACTTTACCCCCAAACCTGTCCTAGATTCCACTATTGATTACTTCCAACCAAACAACAAAAGAAATCAGCTGT
GGCTGAGACTACAAACTGCTGGAAATGTAGACCACGTAGGCCTCGGCACTGCGTTCGAAAACAGTATATACGACCAG
GAATACAATATCCGTGTAACCATGTATGTACAATTCAGAGAATTTAATCTTAAAGACCCCCCACTTAACCCT--------

>FJ644921_pcv2b

ATGACGTATCCAAGGAGGCGTTACCGGAGAAGAAGACACCGCCCCCGCAGCCATCTTGGCCAGATCCTCCGCCGCCG
CCCCTGGCTCCTCCACCCCCGCCACCGTTACCGCTGGAGAAGGAAAAATGGCATCTTCAACACCCGCCTCTCCCGCACC
TTCGGATATACTATCAAGCGAACCACAGTCAAAACGCCCTCCTGGGCGGTGGACATGATGCGATTCAATATTAATGAC
TTTCTTCCCCCAGGAGGGGGCTCAAACCCCCGCTCTGTGCCCTTTGAATACTACAGAATAAGAAAGGTTAAGGTTGAA
TTCTGGCCCTGCTCCCCGATCACCCAGGGTGACAGGGGAGTGGGCTCCAGTGCTGTTATTCTAGATGATAACTTTGTA
ACAAAGGCCACAGCCCTCACCTATGACCCCTATGTAAACTACTCCTCCCGCCATACCATAACCCAGCCCTTCTCCTACCA
CTCCCGCTACTTTACCCCCAAACCTGTCCTAGATTCCACTATTGATTACTTCCAACCAAACAACAAAAGAAATCAGCTGT
GGCTGAGACTACAAACTGCTGGAAATGTAGACCACGTAGGCCTCGGCACTGCGTTCGAAAACAGTATATACGACCAG
GAATACAATATCCGTGTAACCATGTATGTACAATTCAGAGAATTTAATCTTAAAGACCCCCCACTTAACCCT--------

>DQ104422_pcv2b

ATGACGTATCCAAGGAGGCGTTACCGGAGAAGAAGACACCGCCCCCGCAGCCATCTTGGCCAGATCCTCCGCCGCCG
CCCCTGGCTCGTCCACCCCCGCCACCGTTACCGCTGGAGAAGGAAAAATGGCATCTTCAACACCCGCCTCTCCCGCACC
TTCGGATATACTATCAAGCGAACCACAGTCAAAACGCCCTCCTGGGCGGTGGACATGATGCGATTCAATATTAATGAC
TTTCTTCCCCCAGGAGGGGGCTCAAACCCCCGCTCTGTGCCCTTTGAATACTACAGAATAAGAAAGGTTAAGGTTGAA
TTCTGGCCCTGCTCCCCGATCACCCAGGGTGACAGGGGAGTGGGCTCCAGTGCTGTTATTCTAGATGATAACTTTGTA
ACAAAGGCCACAGCCCTCACCTATGACCCCTATGTAAACTACTCCTCCCGCCATACCATAACCCAGCCCTTCTCCTACCA
CTCCCGCTACTTTACCCCCAAACCTGTCCTAGATTCCACTATAGATTACTTCCAACCAAACAACAAAAGAAATCAGCTGT
GGCTGAGACTACAAACTGCTGGAAATGTAGACCACGTAGGCCTCGGCACTGCGTTCGAAAACAGTATATACGACCAG
GAATACAATATCCGTGTAACCATGTATGTACAATTCAGAGAATTTAATCTTAAAGACCCCCCACTTAACCCT--------

>EU545545_pcv2b

ATGACGTATCCAAGGAGGCGTTACCGGAGAAGAAGACACCGCCCCCGCAGCCATCTTGGCCAGATCCTCCGCCGCCG
CCCCTGGCTCCTCCACCCCCGCCACCGTTACCGCTGGAGAAGGAAAAATGGCATCTTCAACACCCGCCTCTCCCGCACC
TTCGGATATACTATCAAGCGAACCACAGTCAAAACGCCCTCCTGGGCGGTGGACATGATGAGATTCAATATTAATGAC
TTTCTTCCCCCAGGAGGGGGCTCAAACCCCCGCTCTGTGCCCTTTGAATACTACAGAATAAGAAAGGTTAAGGTTGAA
TTCTGGCCCTGCTCCCCGATCACCCAGGGTGACAGGGGAGTGGGCTCCAGTGCTGTTATTCTAGATGATAACTTTGTA
ACAAAAGCCACAGCCCTCACCTATGACCCCTATGTAAACTACTCCTCCCGCCATACCATAACCCAGCCCTTCTCCTACCA
CTCCCGCTACTTTACCCCCAAACCTGTCCTAGATTCCACTATTGATTACTTCCAACCAAACAACAAAATAAATCAGCTGT
GGCTGAGACTACAAACTGCTGGAAATGTAGACCACGTAGGCCTCGGCACTGCGTTCGAAAACAGTATATACGACCAG
GAATACAATATCCGTGTAACCATGTATGTACAATTCAGAGAATTTAATCTTAAAGACCCCCCACTTAACCCT--------

>AB462382_pcv2b

ATGACGTATCCAAGGAGGCGTTACCGGAGAAGAAGACACCGCCCCCGCAGCCATCTTGGCCAGATCCTCCGCCGCCG
CCCCTGGCTCGTCCACCCCCGCCACCGTTACCGCTGGAGAAGGAAAAATGGCATCTTCAACACCCGCCTCTCCCGCACC
TTCGGATATACTATCAAGCGAACCACAGTCAAAACGCCCTCCTGGGCGGTGGACATGATGAGATTCAATATTAATGAC
TTTCTTCCCCCAGGAGGGGGCTCAAACCCCCGCTCTGTGCCCTTTGAATACTACAGAATAAGAAAGGTTAAGGTTGAA
TTCTGGCCCTGCTCCCCGATCACCCAGGGTGACAGGGGAGTGGGCTCCAGTGCTGTTATTCTAGATGATAACTTTGTA
ACAAAAGCCACAGCCCTCACCTATGACCCCTATGTAAACTACTCCTCCCGCCATACCATAACCCAGCCCTTCTCCTACCA
CTCCCGCTACTTTACCCCCAAACCTGTCCTAGATTCCACTATTGATTACTTCCAACCAAACAACAAAAGAAATCAGCTGT
GGCTGAGACTACAAACTGCTGGAAATGTAGACCACGTAGGCCTCGGCACTGCGTTCGAAAACAGTATATACGACCAG
GAATACAATATCCGTGTAACCATGTATGTACAATTCAGAGAATTTAATCTTAAAGACCCCCCACTTAACCCT--------

>EU547458_pcv2b

ATGACGTATCCAAGGAGGCGTTACCGGAGAAGAAGACACCGCCCCCGCAGCCATCTTGGCCAGATCCTCCGCCGCCG
CCCCTGGCTCGTCCACCCCCGCCACCGTTACCGCTGGAGAAGGAAAAATGGCATCTTCAACACCCGCCTCTCCCGCACC
TTCGGATATACTATCAAGCGAACCACAGTCAAAACGCCCTCCTGGGCGGTGGACATGATGAGATTCAATATTAATGAC
TTTCTTCCCCCAGGAGGGGGCTCAAACCCCCGCTCTGTGCCCTTTGAATACTACAGAATAAGAAAGGTTAAGGTTGAA
TTCTGGCCCTGCTCCCCGATCACCCAGGGCGACAGGGGAGTGGGCTCCAGTGCTGTTATTCTAAATGATAACTTTGTA
ACAAAGGCCACAGCCCTCACCTATGACCCCTATGTAAACTACTCCTCCCGCCATACCATAACCCAGCCCTTCTCCTACCA
CTCCCGCTACTTTACCCCCAAACCTGTCCTAGATTCCACTATTGATTACTTCCAACCAAACAACAAAAGAAATCAGCTGT
GGCTGAGACTACAAACTGCTGGAAATGTAGACCACGTAGGCCTCGGCACTGCGTTCGAAAACAGTATATACGACCAG
GAATACAATATCCGTGTAACCATGTATGTACAATTCAGAGAATTTAATCTTAAAGACCCCCCACTTAACCCT--------

>DQ355153_pcv2b

ATGACGTATCCAAGGAGGCGTTACCGGAGAAGAAGACACCGCCCCCGCAGCCATCTTGGCCAGATCCTCCGCCGCCG
CCCCTGGCTCGTCCACCCCCGCCACCGTTACCGCTGGAGAAGGAAAAATGGCATCTTCAACACCCGCCTCTCCCGCACC
TTCGGATATACTATCAAGCGAACCACAGTCAAAACGCCCTCCTGGGCGGTGGACATGATGAGATTCAATATTAATGAC
TTTCTTCCCCCAGGAGGGGGCTCAAACCCCCGCTCTGTGCCCTTTGAATACTACAGAATAAGAAAGGTTAAGGTTGAA
TTCTGGCCCTGCTCCCCGATCACCCAGGGCGACAGGGGAGTGGGCTCCAGTGCTGTTATTCTAAATGATAACTTTGTA
ACAAAGGCCACAGCCCTCACCTATGACCCCTATGTAAACTACTCCTCCCGCCATACCATAACCCAGCCCTTCTCCTACCA
CTCCCGCTACTTTACCCCCAAACCTGTCCTAGATTCCACTATTGATTACTTCCAACCAAACAACAAAAGAAATCAGCTGT
GGCTGAGACTACAAACTGCTGGAAATGTAGACCACGTAGGCCTCGGCACTGCGTTCGAAAACAGTATATACGACCAG
GAATACAATATCCGTGTAACCATGTATGTACAATTCAGAGAATTTAATCTTAAAGACCCCCCACTTAACCCT--------

>AY686762_pcv2b

ATGACGTATCCAAGGAGGCGTTACCGGAGAAGAAGACACCGCCCCCGCAGCCATCTTGGCCAGATCCTCCGCCGCCG
CCCCTGGCTCGTCCACCCCCGCCACCGTTACCGCTGGAGAAGGAAAAATGGCATCTTCAACACCCGCCTCTCCCGCACC
TTCGGATATACTATCAAGCGAACCACAGTCAAAACGCCCTCCTGGGCGGTGGACATGATGAGATTCAATATTAATGAC
TTTCTTCCCCCAGGAGGGGGCTCAAACCCCCGCTCTGTGCCCTTTGAATACTACAGAATAAGAAAGGTTAAGGTTGAA
TTCTGGCCCTGCTCCCCGATCACCCAGGGCGACAGGGGAGTGGGCTCCAGTGCTGTTATTCTAGATGATAACTTTGTA
ACAAAGGCCACAGCCCTCACCTATGACCCCTATGTAAACTACTCCTCCCGCCATACCATAACCCAGCCCTTCTCCTACCA
CTCCCGCTACTTTACCCCCAAACCTGTCCTAGATTCCACTATTGATTACTTCCAACCAAACAACAAAAGAAATCAGCTGT
GGCTGAGACTACAAACTGCTGGAAATGTAGACCACGTAGGCCTCGGCACTGCGTTCGAAAACAGTATATACGACCAG
GAATACAATATCCGTGTAACCATGTATGTACAATTCAGAGAATTTAATCTTAAAGACCCCCCACTTAACCCT--------

>FJ644923_pcv2b

ATGACGTATCCAAGGAGGCGTTACCGGAGAAGAAGACACCGCCCCCGCAGCCATCTTGGCCAGATCCTCCGCCGCCG
CCCCTGGCTCCTCCACCCCCGCCACCGTTACCGCTGGAGAAGGAAAAATGGCATCTTCAACACCCGCCTCTCCCGCACC
TTCGGATATACTATCAAGCGAACCACAGTCAAAACGCCCTCCTGGGCGGTGGACATGATGAGATTCAATATTAATGAC
TTTCTTCCCCCAGGAGGGGGCTCAAACCCCCGCTCTGTGCCCTTTGAATACTACAGAATAAGAAAGGTTAAGGTTGAA
TTCTGGCCCTGCTCCCCGATCACCCAGGGCGACAGGGGAGTGGGCTCCAGTGCTGTTATTCTAGATGATAACTTTGTA
ACAAAGGCCACAGCCCTCACCTATGACCCCTATGTAAACTACTCCTCCCGCCATACCATAACCCAGCCCTTCTCCTACCA
CTCCCGCTACTTTACCCCCAAACCTGTCCTAGATTCCACTATTGATTACTTCCAACCAAACAACAAAAGAAATCAGCTGT
GGCTGAGACTACAAACTGCTGGAAATGTAGACCACGTAGGCCTCGGCACTGCGTTCGAAAACAGTATATACGACCAG
GAATACAATATCCGTGTAACCATGTATGTACAATTCAGAGAATTTAATCTTAAAGACCCCCCACTTAACCCT--------

>JN006455_pcv2b

ATGACGTATCCAAGGAGGCGTTACCGGAGAAGAAGACACCGCCCCCGCAGCCATCTTGGCCAGATCCTCCGCCGCCG
CCCCTGGCTCGTCCACCCCCGCCACCGTTACCGCTGGAGAAGGAAAAATGGCATCTTCAACACCCGCCTCTCCCGCACC
TTCGGATATACTATCAAGCGAACCACAGTCAAAACGCCCTCCTGGGCGGTGGACATGATGAGATTCAATATTAATGAC
TTTCTTCCCCCAGGAGGGGGCTCAAACCCCCGCTCTGTGCCCTTTGAATACTACAGAATAAGAAAGGTTAAGGTTGAA
TTCTGGCCCTGCTCCCCGATCACCCAGGGTGACAGGGGAGTGGGCTCCAGTGCTGTTATTCTAGATGATAACTTTGTA
ACAAAGGCCACAGCCCTCACCTATGACCCCTATGTAAACTACTCCTCCCGCCATACCATAACCCAGCCCTTCTCCTACCA
CTCCCGCTACTTTACCCCCAAACCTGTCCTAGATTCCACTATTGATTACTTCCAACCAAACAACAAAAGAAATCAGCTGT
GGCTGAGACTACAAACTGCTGGAAATGTAGACCACGTAGGCCTCGGCACTGCCTTCGAAAACAGTATATACGACCAG
GAATACAATATCCGTGTAACCATGTATGTACAATTCAGGGAATTTAATCTTAAAGACCCCCCACTTAACCCT--------

>JN006456_pcv2b

ATGACGTATCCAAGGAGGCGTTACCGGAGAAGAAGACACCGCCCCCGCAGCCATCTTGGCCAGATCCTCCGCCGCCG
CCCCTGGCTCGTCCACCCCCGCCACCGTTACCGCTGGAGAAGGAAAAATGGCATCTTCAACACCCGCCTCTCCCGCACC
TTCGGATATACTATCAAGCGAACCACAGTCAAAACGCCCTCCTGGGCGGTGGACATGATGAGATTCAATATTAATGAC
TTTCTTCCCCCAGGAGGGGGCTCAAACCCCCGCTCTGTGCCCTTTGAATACTACAGAATAAGAAAGGTTAAGGTTGAA
TTCTGGCCCTGCTCCCCGATCACCCAGGGTGACAGGGGAGTGGGCTCCAGTGCTGTTATTCTAGATGATAACTTTGTA
ACAAAGGCCACAGCCCTCACCTATGACCCCTATGTAAACTACTCCTCCCGCCATACCATAACCCAGCCCTTCTCCTACCA
CTCCCGCTACTTTACCCCCAAACCTGTCCTAGATTCCACTATTGATTACTTCCAACCAAACAACAAAAGAAATCAGCTGT
GGCTGAGACTACAAACTGCTGGAAATGTAGACCACGTAGGCCTCGGCACTGCCTTCGAAAACAGTATATACGACCAG
GAATACAATATCCGTGTAACCATGTATGTACAATTCAGGGAATTTAATCTTAAAGACCCCCCACTTAACCCT--------

>FJ598044_pcv2b

ATGACGTATCCAAGGAGGCGTTACCGGAGAAGAAGACACCGCCCCCGCAGCCATCTTGGCCAGATCCTCCGCCGCCG
CCCCTGGCTCGTCCACCCCCGCCACCGTTACCGCTGGAGAAGGAAAAATGGCATCTTCAACACCCGCCTCTCCCGCACC
TTCGGATATACTATCAAGCGAACCACAGTCAAAACGCCCTCCTGGGCGGTGGACATGATGAGATTCAATATTAATGAC
TTTCTTCCCCCAGGAGGGGGCTCAAACCCCCGCTCTGTGCCCTTTGAATACTACAGAATAAGAAAGGTTAAGGTTGAA
TTCTGGCCCTGCTCCCCGATCACCCAGGGTGACAGGGGAGTGGGCTCCAGTGCTGTTATTCTAGATGATAACTTTGTA
ACAAAGGCCACAGCCCTCACCTATGACCCCTATGTAAACTACTCCTCCCGCCATACCATAACCCAGCCCTTCTCCTACCA
CTCCCGCTACTTTACCCCCAAACCTGTCCTAGATTCCACTATTGATTACTTCCAATCAAACAACAAAAGAAATCAGCTGT
GGCTGAGACTACAAACTGCTGGAAATGTAGACCACGTAGGCCTCGGAACTGCGTTCGAAAACAGTATATACGACCAG
GAATACAATATCCGTGTAACCATGTATGTACAATTCCGGGAATTTAATCTTAAAGACCCCCCACTTAACCCT--------

>FJ644922_pcv2b

ATGACGTATCCAAGGAGGCGTTACCGGAGAAGAAGACACCGCCCCCGCAGCCATCTTGGCCAGATCCTCCGCCGCCG
CCCCTGGCTCCTGCACCCCCGCCACCGTTACCGCTGGAGAAGGAAAAATGGCATCTTCAACACCCGCCTCTCCCGCACC
TTCGGATATACTATCAAGCGAACCACAGTCAAAACGCCCTCCTGGGCGGTGGACATGATGAGATTCAATATTAATGAC
TTTCTTCCCCCAGGAGGGGGCTCAAACCCCCGCTCTGTGCCCTTTGAATACTACAGAATAAGAAAGGTTAAGGTTGAA
TTCTGGCCCTGCTCCCCGATCACCCAGGGTGACAGGGGAGTGGGCTCCAGTGCTGTTATTCTAGATGATAACTTTGTA
ACAAAGGCCACAGCCCTCACCTATGACCCCTATGTAAACTACTCCTCCCGCCATACCATAACCCAGCCCTTCTCCTACCA
CTCCCGCTACTTTACCCCCAAACCTGTCCTAGATTCCACTATTGATTACTTCCAACCAAACAACAAAAGAAATCAGCTGT
GGCTGAGACTACAAACTGCTGGAAATGTAGACCACGTAGGCCTCGGCACTGCGTTCGAAAACAGTATATACGACCAG
GAATACAATATCCGTGTAACCATGTATGTACAATTCAGGGAATTTAATCTTAAAGACCCCCCACTTAACCCT--------

>FJ667591_pcv2b

ATGACGTATCCAAGGAGGCGTTACCGGAGAAGAAGACACCGCCCCCGCAGCCATCTTGGCCAGATCCTCCGCCGCCG
CCCCTGGCTCCTCCACCCCCGCCACCGTTACCGCTGGAGAAGGAAAAATGGCATCTTCAACACCCGCCTCTCCCGCACC
TTCGGATATACTATCAAGCGAACCACAGTCAAAACGCCCCCCTGGGCGGTGGACATGATGAGATTCATTATTAATGAC
TTTCTTCCCCCAGGAGGGGGCTCAAACCCCCGCTCTGTGCCCTTTGAATACTACAGAATAAGAAAGGTTAAGGTTGAA
TTCTGGCCCTGCTCCCCGATCACCCAGGGTGACAGGGGAGTGGGCTCCAGTGCTGTTATTCTAGATGATAACTTTGTA
ACAAAGGCCACAGCCCTCACCTATGACCCCTATGTAAACTACTCCTCCCGCCATACCATAACCCAGCCCTTCTCCTACCA
CTCCCGCTACTTTACCCCCAAACCTGTCCTAGATTCCACTATTGATTACTTCCAACCAAACAACAAAAGAAATCAGCTCT
GGCTGAGACTACAAACTACTGGAAATGTAGACCACGTAGGCCTCGGCACTGCGTTCGAAAACAGTATATACGACCAG
GAATACAATATCCGTGTAACCATGTATGTACAATTCAGAGAATTTAATCTTAAAGACCCCCCACTTAACCCT--------

>FJ667593_pcv2b

ATGACGTATCCAAGGAGGCGTTACCGGAGAAGAAGACACCGCCCCCGCAGCCATCTTGGCCAGATCCTCCGCCGCCG
CCCCTGGCTCCTCCACCCCCGCCACCGTTACCGCTGGAGAAGGAAAAATGGCATCTTCAACACCCGCCTCTCCCGCACC
TTCGGATATACTATCAAGCGAACCACAGTCAAAACGCCCTCCTGGGCGGTGGACATGATGAGATTCAATATTAATGAC
TTTCTTCCCCCAGGAGGGGGCTCAAGCCCCCGCTCTGTGCCCTTTGAATACTACAGAATAAGAAAGGTTAAGGTTGAA
TTCTGGCCCTGCTCCCCGATCACCCAGGGTGACAGGGGAGTGGGCTCCAGTGCTGTTATTCTAGATGATAACTTTGTA
ACAAAGGCCACAGCCCTCACCTATGACCCCTATGTAAACTACTCCTCCCGCCATACCATAACCCAGCCCTTCTCCTACCA
CTCCCGCTACTTTACCCCCAAACCTGTCCTAGATTCCACTATTGATTACTTCCAACCAAACAACAAAAGAAATCAGCTCT
GGCTGAGACTACAAACTACTGGAAATGTAGACCACGTAGGCCTCGGCACTGCGTTCGAAAACAGTATATACGACCAG
GAATACAATATCCGTGTAACCATGTATGTACAATTCAGAGAATTTAATCTTAAAGACCCCCCACTTAACCCT--------

>FJ667592_pcv2b

ATGACGTATCCAAGGAGGCGTTACCGGAGAAGAAGACACCGCCCCCGCAGCCATCTTGGCCAGATCCTCCGCCGCCG
CCCCTGGCTCCTCCACCCCCGCCACCGTTACCGCTGGAGAAGGAAAAATGGCATCTTCAACACCCGCCTCTCCCGCACC
TTCGGATATACTATCAAGCGAACCACAGTCAAAACGCCCTCCTGGGCGGTGGACATGATGAGATTCAATATTAATGAC
TTTCTTCCCCCAGGAGGGGGCTCAAACCCCCGCTCTGTGCCCTTTGAATACTACAGAATAAGAAAGGTTAAGGTTGAA
TTCTGGCCCTGCTCCCCGATCACCCAGGGTGACAGGGGAGTGGGCTCCAGTGCTGTTATTCTAGATGATAACTTTGTA
ACAAAGGCCACAGCCCTCACCTATGACCCCTATGTAAACTACTCCTCCCGCCATACCATAACCCAGCCCTTCTCCTACCA
CTCCCGCTACTTTACCCCCAAACCTGTCCTAGATTCCACTATTGATTACTTCCAACCAAACAACAAAAGAAATCAGCTCT
GGCTGAGACTACAAACTACTGGAAATGTAGACCACGTAGGCCTCGGCACTGCGTTCGAAAACAGTATATACGACCAG
GAATACAATATCCGTGTAACCATGTATGTACAATTCAGAGAATTTAATCTTAAAGACCCCCCGCTTAACCCT--------

>FJ667587_pcv2b

ATGACGTATCCAAGGAGGCGTTACCGGAGAAGAAGACACCGCCCCCGCAGCCATCTTGGCCAGATCCTCCGCCGCCG
CCCCTGGCTCCTCCACCCCCGCCACCGTTACCGCTGGAGAAGGAAAAATGGCATCTTCAACACCCGCCTCTCCCGCACC
TTCGGATATACTATCAAGCGAACCACAGTCAAAACGCCCTCCTGGGCGGTGGACATGATGAGATTCAATATTAAGGAC
TTTCTTCCCCCAGGAGGGGGCTCAAACCCCCGCTCTGTGCCCTTTGAATACTACAGAATAAGAAAGGTTAAGGTTGAA
TTCTGGCCCTGCTCCCCGATCACCCAGGGTGACAGGGGAGTGGGCTCCAGTGCTGTTATTCTAGATGATAACTTTGTA
ACAAAGGCCACAGCCCTCACCTATGACCCCTATGTAAACTACTCCTCCCGCCATACCATAACCCAGCCCTTCTCCTACCA
CTCCCGCTACTTTACCCCCAAACCTGTCCTAGATTCCACTATTGATTACTTCCAACCAAACAACAAAAGAAATCAGCTCT
GGCTGAGACTACAAACTACTGGAAATGTAGACCACGTAGGCCTCGGCACTGCGTTCGAAAACAGTATATACGACCAG
GAATACAATATCCGTGTAACCATGTATGTACAATTCAGAGAATTTAATCTTAAAGACCCCCCACTTAACCCT--------

>FJ667589_pcv2b

ATGACGTATCCAAGGAGGCGTTACCGGAGAAGAAGACACCGCCCCCGCAGCCATCTTGGCCAGATCCTCCGCCGCCG
CCCCTGGCTCCTCCACCCCCGCCACCGTTACCGCTGGAGAAGGAAAAATGGCATCTTCAACACCCGCCTCTCCCGCACC
TTCGGATATACTATCAAGCGAACCACAGTCAAAACGCCCTCCTGGGCGGTGGACATGATGAGATTCAATATTAATGAC
TTTCTTCCCCCAGGAGGGGGCTCAAACCCCCGCTCTGTGCCCTTTGAATACCACAGAATAAGAAAGGTTAAGGTTGAA
TTCTGGCCCTGCTCCCCGATCACCCAGGGTGACAGGGGAGTGGGCTCCAGTGCTGTTATTCTAGATGATAACTTTGTA
ACAAAGGCCACAGCCCTCACCTATGACCCCTATGTAAACTACTCCTCCCGCCATACCATAACCCAGCCCTTCTCCTACCA
CTCCCGCTACTTTACCCCCAAACCTGTCCTAGATTCCACTATTGATTACTTCCAACCAAACAACAAAAGAAATCAGCTCT
GGCTGAGACTACAAACTACTGGAAATGTAGACCACGTAGGCCTCGGCACTGCGTTCGAAAACAGTATATACGACCAG
GAATACAATATCCGTGTAACCATGTATGTACAATTCAGAGAATTTAATCTTAAAGACCCCCCACTTAACCCT--------

>FJ667584_pcv2b

ATGACGTATCCAAGGAGGCGTTACCGGAGAAGAAGACACCGCCCCCGCAGCCATCTTGGCCAGATCCTCCGCCGCCG
CCCCTGGCTCCTCCACCCCCGCCACCGTTACCGCTGGAGAAGGAAAAATGGCATCTTCAACACCCGCCTCTCCCGCACC
TTCGGATATACTATCAAGCGAACCACAGTCAAAACGCCCTCCTGGGCGGTGGACATGATGAGATTCAATATTAATGAC
TTTCTTCCCCCAGGAGGGGGCTCAAACCCCCGCTCTGTGCCCTTTGAATACTACAGAATAAGAAAGGTTAAGGTTGAA
TTCTGGCCCTGCTCCCCGATCACCCAGGGTGACAGGGGAGTGGGCTCCAGTGCTGTTATTCTAGATGATAACTTTGTA
ACAAAGGCCACAGCCCTCACCTATGACCCCTATGTAAACTACTCCTCCCGCCATACCATAACCCAGCCCTTCTCCTACCA
CTCCCGCTACTTTACCCCCAAACCTGTCCTAGATTCCACTATTGATTACTTCCAACCAAACAACAAAAGAAATCAGCTCT
GGCTGAGACTACAAACTACTGGAAATGTAGACCACGTAGGCCTCGGCACTGCGTTCGAAAACAGTATATACGACCAG
GAATACAATATCCGTGTAACCATGTATGTACAATTCAGAGAATTTAATCTTAAAGACCCCCCACTTAACCCT--------

>EF190924_pcv2b

ATGACGTATCCAAGGAGGCGTTACCGGAGAAGAAGACACCGCCCCCGCAGCCATCTTGGCCAGATCCTCCGCCGCCG
CCCCTGGCTCCTCCACCCCCGCCACCGTTACCGCTGGAGAAGGAAAAATGGCATCTTCAACACCCGCCTCTCCCGCACC
TTCGGATATACTATCAAGCGAACCACAGTCAAAACGCCCTCCTGGGCGGTGGACATGATGAGATTCAATATTAATGAC
TTTCTTCCCCCAGGAGGGGGCTCAAACCCCCGCTCTGTGCCCTTTGAATACTACAGAATAAGAAAAGTTAAGGTTGAA
TTCTGGCCCTGCTCCCCGATCACCCAGGGTGACAGGGGAGTGGGCTCCAGTGCTGTTATTCTAGATGATAACTTTGTA
ACAAAGGCCACAGCCCTCACCTATGACCCCTATGTAAACTACTCCTCCCGCCATACCATAACCCAGCCCTTCTCCTACCA
CTCCCGCTACTTTACCCCCAAACCTGTCCTAGATTCCACTATTGATTACTTCCAACCAAACAACAAAAGAAATCAGCTCT
GGCTGAGACTACAAACTGTTGGAAATGTAGACCACGTAGGCCTCGGCACCGCGTTCGAAAACAGTATATACGACCAG
GAATACAATATCCGTGTAACCATGTATGTACAATTCAGAGAATTTAATCTTAAAGACCCCCCACTTAACCCT--------

>JX534236_pcv2b

ATGACGTATCCAAGGAGGCGTTACCGGAGAAGAAGACACCGCCCCCGCAGCCATCTTGGCCAGATCCTCCGCCGCCG
CCCCTGGCTCCTCCACCCCCGCCACCATTACCGCTGGAGAAGGAAAAATGGCATCTTCAACACCCGCCTCTCCCGCACC
TTCGGATATACTATCAAGCGAACCACAGTCAAAACGCCCTCCTGGGCGGTGGACATGATGAGATTCAATATTAATGAC
TTTCTTCCCCCAGGAGGGGGCTCAAACCCCCGCTCTGTGCCCTTTGAATACTACAGAATAAGAAAGGTTAAGGTTGAA
TTCTGGCCCTGCTCCCCGATCACCCAGGGTGACAGGGGAGTGGGCTCCACTGCTGTTATTCTAGATGATAACTTTGTA
ACAAAGGCCACAGCCCTCACCTATGACCCCTATGTAAACTACTCCTCCCGCCATACCATAACCCAGCCCTTCTCCTACCA
CTCCCGCTACTTTACCCCGAAACCTGTCCTAGATTCCACTATTGATTACTTCCAACCAAACAACAAAAGAAATCAGCTCT
GGCTGAGACTACAAACTGCTGGAAATGTAGACCACGTAGGCCTCGGCACTGCGTTCGAAAACAGTATATACGACCAG
GACTACAATATCCGTGTAACCATGTATGTACAATTCAGAGAATTTAATCTTAAAGACCCCCCCCTTAACCCT--------

>JX534237_pcv2b

ATGACGTATCCAAGGAGGCGTTACCGCAGAAGAAGACACCGCCCCCGCAGCCATCTTGGCCAGATCCTCCGCCGCCG
CCCCTGGCTCCTCCACCCCCGCCACCATTACCGCTGGAGAAGGAAAAATGGCATCTTCAACACCCGCCTCTCCCGCACC
TTCGGATATACTATCAAGCGAACCACAGTCAAAACGCCCTCCTGGGCGGTGGACATGATGAGATTTAATATTAATGAC
TTTCTTCCCCCAGGAGGGGGCTCAAACCCCCGCTCTGTGCCCTTTGAATACTACAGAATAAGAAAGGTTAAGGTTGAA
TTCTGGCCCTGCTCCCCGATCACCCAGGGTGACAGGGGAGTGGGCTCCAGTGCTGTTATTCTAGATGATAACTTTGTA
ACAAAGGCCACAGCCCTCACCTATGACCCCTATGTAAACTACTCCTCCCGCCATACCATAACCCAGCCCTTCTCCTACCA
CTCCCGCTACTTTACCCCCAAACCTGTCCTAGATTCCACTATTGATTACTTCCAACCAAACAACAAAAGAAATCAGCTGT
GGCTGAGACTACAAACTGCTGGAAATGTAGACCACGTAGGCCTCGGCACTGCGTTCGAAAACAGTATATACGACCAG
GAATACAATATCCGTGTAACCATGTATGTACAATTCAGAGAATTTAATCTTAAAGACCCCCCCCTTAACCCT--------

>EU656143_pcv2b

ATGACGTATCCAAGGAGGCGTTACCGGAGAAGAAGACACCGCCCCCGCAGCCATCTTGGCCAGATCCTCCGCCGCCG
CCCCTGGCTCCTCCACCCCCGCCACCATTACCGCTGGAGAAGGAAAAATGGCATCTTCAACACCCGCCTCTCCCGCACC
TTCGGATATACTATCAAGCGAACCACAGTCAAAACGCCCTCCTGGGCGGTGGACATGATGAGATTCAATATTAATGAC
TTTCTTCCCCCAGGAGGGGGCTCAAACCCCCGCTCTGTGCCCTTTGAATACTACAGAATAAGAAAGGTTAAGGTTGAA
TTCTGGCCCTGCTCCCCGATCACCCAGGGTGACAGGGGAGTGGGCTCCAGTGCTGTTATTCTAGATGATAACTTTGTA
ACAAAGGCCACAGCCCTCACCTATGACCCCTATGTAAACTACTCCTCCCGCCATACCATAACCCAGCCCTTCTCCTACCA
CTCCCGCTACTTTACCCCCAAACCTGTCCTAGATTCCACTATTGATTACTTCCAACCAAACAACAAAAGAAATCAGCTGT
GGCTGAGACTACAAACTGCTGGAAATGTAGACCACGTAGGCCTCGGCACTGCGTTCGAAAACAGTATATACGACCAG
GAATACAATATCCGTGTAACCATGTATGTACAATTCAGAGAATTTAATCTTAAAGACCCCCCCCTTAACCCT--------

>EU257515_pcv2b

ATGACGTATCCAAGGAGGCGTTACCGGAGAAGAAGACACCGCCCCCGCAGCCATCTTGGCCAGATCCTCCGCCGCCG
CCCCTGGCTCCTCCACCCCCGCCACCGTTACCCCTGGAGAAGGAAAAATGGCATCTTCAACACCCGCCTCTCCCGCACC
TTCGGATATACTATCAAGCGAACCACAGTCAAAACGCCCCCCTGGGCGGTGGACATGATGAGATTCAATATTAATGAC
TTTCTTCCCCCAGGAGGGGGCTCAAACCCCCGCTCTGTGCCCTTTGAATACTACAGAATAAGAAAGGTTAAGGTTGAA
TTCTGGCCCTGTTCCCCGATCACCCAGGGTGACAGGGGAGTGGGCTCCAGTGCTGTTATTCTAGATGATAACTTTGTA
ACAAAGGCCACAGCCCTCACCTATGACCCCTATGTAAACTACTCCTCCCGCCATACCATAACCCAACCCTTCTCCTACCA
CTCCCGCTACTTTACCCCCAAACCTGTCCTAGATTCCACTATTGATTACTTCCAACCAAACAACAAAAGAAATCTGCTGT
GGCTGAGACTACAAACTGCTGGAAATGTAGACCACGTAGGCCTCGGCACTGCGTTCGAAAACAGTATATACGACCAG
GAATACAATATCCGTGTAACCATGTATGTACAATTCAGAGAATTTAATCTTAAAGACCCCCCACTTAACCCT--------

>HQ693092_pcv2b

ATGACGTATCCAAGGAGGCGTTACCGGAGAAGAAGACACCGCCCCCGCAGCCATCTTGGCCAGATCCTCCGCCGCCG
CCCCTGGCTCCTCCACCCCCGCCACCGTTACCGCTGGAGAAGGAAAAATGGCATCTTCAACACCCGCCTCTCCCGCACC
TTCGGATATACTATCAAGCGAACCACAGTCAAAACGCCCCCCTGGGCGGTGGACATGATGAGATTCAATATTAATGAC
TTTCTTCCCCCAGGAGGGGGCTCAAACCCCCGCTCTGTGCCCTTTGAATACTACAGAATAAGAAAGGTTAAGGTTGAA
TTCTGGCCCTGCTCCCCGATCACCCAGGGTGACAGGGGAGTGGGCTCCAGTGCTGTTATTCTAGATGATAACTTTGTA
ACAAAGGCCACAGCCCTCACCTATGACCCCTATGTAAACTACTCCTCCCGCCATACCATAACCCAGCCCTTCTCCTACCA
CTCCCGCTACTTTACCCCCAAACCTATCCTAGATTCCACTATTGATTACTTCCAACCAAACAACAAAAGAAATCAGCTGT
GGCTGAGACTACAAACTGCTGGAAATGTAGACCACGTAGGCCTCGGCACTGCGTTCGAAAACAGTATATACGACCAG
GAATACAATATCCGTGTAACCATGTATGTACAATTCAGAGAATTTAATCTTAAAGACCCCCCCCTTAACCCT--------

>HM776438_pcv2b

ATGACGTATCCAAGGAGGCGTTACCGGAGAAGAAGACACCGCCCCCGCAGCCATCTTGGCCAGATCCTCCGCCGCCG
CCCCTGGCTCCTCCACCCCCGCCACCGTTACCGCTGGAGAAGGAAAAATGGCATCTTCAACACCCGCCTCTCCCGCACC
TTCGGGTATACTATCAAGCGAACCACAGTCAAAACGCCCTCCTGGGCGGTGGACATGATGAGATTCAATATTAATGAC
TTTCTTCCCCCAGGAGGGGGCTCAAACCCCCGCTCTGTGCCCTTTGAATACTACAGAATAAGAAAGGTTAAGGTTGAA
TTCTGGCCCTGCTCCCCGATCACCCAGGGTGACAGGGGAGTGGGCTCCAGTGCTGTTATTCTAGATGATAACTTTGTA
ACAAAGGCCACAGCCCTCACCTATGACCCCTATGTAAACTACTCCTCCCGCCATACCATAACCCAGCCCTTCTCCTACCA
CTCCCGCTACTTTACCCCCAAACCTGTCCTAGATTCCACTATTGATTACTTCCAACCAAACAACAAAAGAAATCAGCTGT
GGCTGAGACTACAAACTGCTGGAAATGTAGACCACGTAGGCCTCGGCACTGCGTTCGAAAACAGTATATACGACCAG
GACTACAATATCCGTATAACCATGTATGTACAATTCAGAGAATTTAATCTTAAAGACCCCCCACTTAACCCTAAG-----

>GQ174519_pcv2b

ATGACGTATCCAAGGAGGCGTTACCGGAGAAGAAGACACCGCCCCCGCAGCCATCTTGGCCAGATCCTCCGCCGCCG
CCCCTGGCTCCTCCACCCCCGCCACCGTTACCGCTGGAGAAGGAAAAATGGCATCTTCAACACCCGCCTCTCCCGCACC
TTCGGATATACTATCAAGCGAACCACAGTCAAAACGCCCTCCTGGGCGGTGGACATGATGAGATTCAATATTAATGAC
TTTCTTCCCCCAGGAGGGGGCTCAAACCCCCGCTCTGTGCCCTTTGAATACTACAGAATAAGAAAGGTTAAGGTTGAA
TTCTGGCCCTGCTCCCCGATCACCCAGGGTGACAGGGGAGTGGGCTCCAGTGCTGTTATTCTAGATGATAACTTTGTA
ACAAAGGCCACAGCCCTCACCTATGACCCCTATGTAAACTACTCCTCCCGCCATACCATAACCCAGCCCTTCTCCTACCA
CTCCCGCTACTTTACCCCCAAACCTGTCCTAGATTCCACTATTGATTACTTCCAACCAAACAACAAAAGAAATCAGCTGT
GGCTGAGACTACAAACTGCTGGAAATGTAGACCACGTAGGCCTCGGCACTGCGTTCGAAAACAGTATATACGACCAG
GAATACAATATCCGTGTAACCATGTATGTACAATTCAGAGAATTTAATCTTAAAGACCCCCCACTTAACCCT--------

>FJ384968_pcv2b

ATGACGTATCCAAGGAGGCGTTACCGGAGAAGAAGACACCGCCCCCGCAGCCATCTTGGCCAGATCCTCCGCCGCCG
CCCCTGGCTCCTCCACCCCCGCCACCGTTACCGCTGGAGAAGGAAAAATGGCATCTTCAACACCCGCCTCTCCCGCACC
TTCGGATATACTATCAAGCGAACCACAGTCAAAACGCCCTCCTGGGCGGTGGACATGATGAGATTCAATATTAATGAC
TTTCTTCCCCCAGGAGGGGGCTCAAACCCCCGCTCTGTGCCCTTTGAATACTACAGAATAAGAAAGGTTAAGGTTGAA
TTCTGGCCCTGCTCCCCGATCACCCAGGGTGACAGGGGAGTGGGCTCCAGTGCTGTTATTCTAGATGATAACTTTGTA
ACAAAGGCCACAGCCCTCACCTATGACCCCTATGTAAACTACTCCTCCCGCCATACCATAACCCAGCCCTTCTCCTACCA
CTCCCGCTACTTTACCCCCAAACCTGTCCTAGATTCCACTATTGATTACTTCCAACCAAACAACAAAAGAAATCAGCTGT
GGCTGAGACTACAAACTGCTGGAAATGTAGACCACGTAGGCCTCGGCACTGCGTTCGAAAACAGTATATACGACCAG
GAATACAATATCCGTGTAACCATGTATGTACAATTCAGAGAATTTAATCTTAAAGACCCCCCACTTAACCCT--------

>EF210106_pcv2b

ATGACGTATCCAAGGAGGCGTTACCGGAGAAGAAGACACCGCCCCCGCAGCCATCTTGGCCAGATCCTCCGCCGCCG
CCCCTGGCTCCTCCACCCCCGCCACCGTTACCGCTGGAGAAGGAAAAATGGCATCTTCAACACCCGCCTCTCCCGCACC
TTCGGATATACTATCAAGCGAACCACAGTCAAAACGCCCTCCTGGGCGGTGGACATGATGAGATTCAATATTAATGAC
TTTCTTCCCCCAGGAGGGGGCTCAAACCCCCGCTCTGTGCCCTTTGAATACTACAGAATAAGAAAGGTTAAGGTTGAA
TTCTGGCCCTGCTCCCCGATCACCCAGGGTGACAGGGGAGTGGGCTCCAGTGCTGTTATTCTAGATGATAACTTTGTA
ACAAAGGCCACAGCCCTCACCTATGACCCCTATGTAAACTACTCCTCCCGCCATACCATAACCCAGCCCTTCTCCTACCA
CTCCCGCTACTTTACCCCCAAACCTGTCCTAGATTCCACTATTGATTACTTCCAACCAAACAACAAAAGAAATCAGCTGT
GGCTGAGACTACAAACTGCTGGAAATGTAGACCACGTAGGCCTCAGCACTGCGTTCGAAAACAGTATATACGACCAG
GAATACAATATCCGTGTAACCATGTATGTACAATTCAGAGAATTTAATCTTAAAGACCCCCCACTTAACCCT--------

>AY578327_pcv2b

ATGACGTATCCAAGGAGGCGTTACCGGAGAAGAAGACACCGCCCCCGCAGCCATCTTGGCCAGATCCTCCGCCGCCG
CCCCTGGCTCCTCCACCCCCGCCACCGTTACCGCTGGAGAAGGAAAAATGGCATCTTCAACACCCGCCTCTCCCGCACC
TTCGGATATACTATCAAGCGAACCACAGTCAAAACGCCCTCCTGGGCGGTGGACATGATGAGATTCAATATTAATGAC
TTTCTTCCCCCAGGAGGGGGCTCAAACCCCCGCTCTGTGCCCTTTGAATACTACAGAATAAGAAAGGTTAAGGTTGAA
TTCTGGCCCTGCTCCCCGATCACCCAGGGTGACAGGGGAGTGGGCTCCAGTGCTGTTATTCTAGATGATAACTTTGTA
ACAAAGGCCACAGCCCTCACCTATGACCCCTATGTAAACTACTCCTCCCGCCATACCATAACCCAGCCCTTCTCCTACCA
CTCCCGCTACTTTACCCCCAAACCTGTCCTAGATTCCACTATTGATTACTTCCAACCAAACAACAAAAGAAATCAGCTGT
GGCTGAGACTACAAACTGCTGGAAATGTAGACCACGTAGGCCTCAGCACTGCGTTCGAAAACAGTATATACGACCAG
GAATACAATATCCGTGTAACCATGTATGTACAATTCAGAGAATTTAATCTTAAAGACCCCCCACTTAACCCT--------

>GU233804_pcv2b

ATGACGTATCCAAGGAGGCGTTACCGGAGAAGAAGACACCGCCCCCGCAGCCATCTTGGCCAGATCCTCCGCCGCCG
CCCCTGGCTCCTCCACCCCCGCCACCGTTACCGCTGGAGAAGGAAAAATGGCATCTTCAACACCCGCCTCTCCCGCACC
TTCGGATATACTATCAAGCGAACCACAGTCAAAACGCCCTCCTGGGCTGTGGACATGACGAGATTCAATATTAATGAC
TTTCTTCCCCCAGGAGGGGGCTCAAACCCCCGCTCTGTGCCCTTTGAATACTACAGAATAAGAAAGGTTAAGGTTGAA
TTCTGGCCCTGCTCCCCGACCACCCAGGGTGACAGGGGAGTGGGCTCCAGTGCTGTTATTCTAGATGATAACTTTGTA
ACAAAGGCCACAGCCCTCACCTATGACCCCTATGTAAACTACTCCTCCCGCCATACCATAACCCAGCCCTTCTCCTACCA
CTCCCGCTACTTTACCCCCAAACCTGTCCTAGATTCCACTATTGATTACTTCCAACCAAACAACAAAAGAAATCAGCTGT
GGCTGAGACTACAAACTGCTGGAAATGTAGACCACGTAGGCCTCGGCACTGCGTTCGAAAACAGTATATACGACCAG
GAATACAATATCCGTGTAACCATGTATGTACAATTCAGAGAATTTAATCTTAAAGACCCCCCACTTAACCCT--------

>AY556475_pcv2b

ATGACGTATCCAAGGAGGCGTTACCGGAGAAGAAGACACCGCCCCCGCAGCCATCTTGGCCAGATCCTCCGCCGCCG
CCCCTGGCTCCTCCACCCCCGCCACCGTTACCGCTGGAGAAGGAAAAATGGCATCTTCAACACCCGCCTCTCCCGCACC
TTCGGATATACTATCAAGCGAACCACAGTCAAAACGCCCTCCTGGGCGGTGGACATGATGAGATTCAATATTAATGAC
TTTCTTCCCCCAGGAGGGGGCTCAAACCCCCGCTCTGTGCCCTTTGAATACTACAGAATAAGAAAGGTTAAGGTTGAA
TTCTGGCCCTGCTCCCCGATCACCCAGGGTGACAGGGGAGTGGGCTCCAGTGCTGTTATTCTAGATGATAACTTTGTA
ACAAAGGCCACAGCCCTCACCTATGACCCCTATGTAAACTACTCCTCCCGCCATACCATAACCCAGCCCTTCTCCTACCA
CTCCCGCTACTTTACCCCCAAACCTGTCCTAGATTCCACTATTGATTACTTCCAACCAAACAACAAAAGAAATCAGCTGT
GGCTGAGACTACAAACTGCTGGAAATGTAGACCACGTAGGCCTCGGCACTGCGTTCGAAAACAGTATATACGACCAG
GAATACAATATCCGTGTAACCATGTATGTACAATTCAGAGAATTTAATCTTAAAGACCCCCCACTTAACCCT--------

>GU247990_pcv2b

ATGACGTATCCAAGGAGGCGTTACCGGAGAAGAAGACACCGCCCCCGCAGCCATCTTGGCCAGATCCTCCGCCGCCG
CCCCTGGCTCCTCCACCCCCGCCACCGTTACCGCTGGAGAAGGAAAAATGGCATCTTCAACACCCGCCTCTCCCGCACC
TTCGGATATACTATCAAGCGAACCACAGTCAAAACGCCCTCCTGGGCGGTGGACATGATGAGATTCAATATTAATGAC
TTTCTTCCCCCAGGAGGGGGCTCAAACCCCCGCTCTGTGCCCTTTGAATACTACAGAATAAGAAAGGTTAAGGTTGAA
TTCTGGCCCTGCTCCCCGATCACCCAGGGTGACAGGGGAGTGGGCTCCAGTGCTGTTATTCTAGATGATAACTTTGTG
ACAAAGGCCACAGCCCTCACCTATGACCCCTATGTAAACTACTCCTCCCGCCATACCATAACCCAGCCCTTCTCCTACCA
CTCCCGCTACTTTACCCCCAAACCTGTCCTAGATTCCACTATTGATTACTTCCAACCAAACAACAAAAGAAATCAGCTGT
GGCTGAGACTACAAACTGCTGGAAATGTAGACCACGTAGGCCTCGGCACTGCGTTCGAAAACAGTATATACGACCAG
GAATACAATATCCGTGTAACCATGTATGTACAATTCAGAGAATTTAATCTTAAAGACCCCCCACTTAACCCT--------

>EF190935_pcv2b

ATGACGTATCCAAGGAGGCGTTACCGGAGAAGAAGACACCGCCCCCGCAGCCATCTTGGCCAGATCCTCCGCCGCCG
CCCCTGGCTCCTCCACCCCCGCCACCGTTACCGCTGGAGAAGGAAAAATGGCATCTTCAACACCCGCCTCTCCCGCACC
TTCGGATATACTATCAAGCGAACCACAGTCAAAACGCCCTCCTGGGCGGTGGACATGATGAGATTCAATATTAATGAC
TTTCTTCCCCCAGGAGGGGGCTCAAACCCCCGCTCTGTGCCCTTTGAATACTACAGAATAAGAAAGGTTAAGGTTGAA
TTCTGGCCCTGCTCCCCGATCACCCAGGGTGACAGGGGAGTGGGCTCCAGTGCTGTTATTCTAGATGATAACTTTGTA
ACAAAGGCCACAGCCCTCACCTATGACCCCTATGTAAACTACTCATCCCGCCATACCATAACCCAGCCCTTCTCCTACCA
CTCCCGCTACTTTACCCCCAAACCTGTCCTAGATTCCACTATTGATTACTTCCAACCAAACAACAAAAGAAATCAGCTGT
GGCTGAGACTACAAACTGCTGGAAATGTAGACCACGTAGGCCTCGGCACTGCGTTCGAAAACAGTATATACGACCAG
GAATACAATATCCGTGTAACCATGTATGTACAATTCAGAGAATTTAATCTTAAAGACCCCCCACTTAACCCT--------

>JN989557_pcv2b

ATGACGTATCCAAGGAGGCGTTACCGGAGAAGAAGACACCGCCCCCGCAGCCATCTTGGCCAGATCCTCCGCCGCCG
CCCCTGGCTCCTCCACCCCCGCCACCGTTACCGCTGGAGAAGGAAAAATGGCATCTTCAACACCCGCCTCTCCCGCACC
TTCGGATATACTATCAAGCGAACCACAGTCAAAACGCCCTCCTGGGCGGTGGACATGATGAGATTCAATATTAATGAC
TTTCTTCCCCCAGGAAGGGGCTCAAACCCCCGCTCTGTGCCCTTTGAATACTACAGAATAAGAAAGGTTAAGGTTGAA
TTCTGGCCCTGCTCCCCGATCACCCAGGGTGACAGGGGAGTGGGCTCCAGTGCTGTTATTCTAGATGATAACTTTGTA
ACAAAGGCCACAGCCCTCACCTATGACCCCTATGTAAACTACTCCTCCCGCCATACCATAACCCAGCCCTTCTCCTACCA
CTCCCGCTACTTTACCCCCAAACCTGTCCTAGATTCCACTATTGATTACTTCCAACCAAACAACAAAAGAAATCAGCTGT
GGCTGAGACTACAAACTGCTGGAAATGTAGACCACGTAGGCCTCGGCACTGCGTTCGAAAACAGTATATACGACCAG
GAATACAATATCCGTGTAACCATGTATGTACAATTCAGAGAATTTAATCTTAAAGACCCCCCACTTAACCCT--------

>DQ195679_pcv2b

ATGACGTATCCAAGGAGGCGTTACCGGAGAAGAAGACACCGCCCCCGCAGCCATCTTGGCCAGATCCTCCGCCGCCG
CCCCTGGCTCCTCCACCCCCGCCACCGTTACCGCTGGAGAAGGAAAAATGGCATCTTCAACACCCGCCTCTCCCGCACC
TTCGGATATACTATCAAGCGAACCACAGTCAAAACGCCCTCCTGGGCGGTGGACATGATGAGATTCAATATTAATGAC
TTTCTTCCCCCAGGAGGGGGCTCAAACCCCCGCTCTGTGCCCTTTGAATACTACAGAATAAGAAAGGTTAAGGTTGAA
TTCTGGCCCTGCTCCCCGATCACCCAGGGTGACAGGGGAGTGGGCTCCAGTGCTGTTATTCTAGATGATAACTTTGTA
ACAAAGGCCACAGCCCTCACCTATGACCCCTATGTAAACTACTCCTCCCGCCATACCATAACCCAGCCCTTCTCCTACCA
CTCCCGCTACTTTACCCCCAAACCTGTCCTAGATTCCACTATTGATTACTTCCAACCAAACAACAAAAGAAATCAGCTGT
GGCTGAGACTACAAACTGCTGGAAATGTAGACCACGTAGGCCTCGGCACTGCGTTCGAAAACAGTATATATGACCAG
GAATACAATATCCGTGTAACCATGTATGTACAATTCAGAGAATTTAATCTTAAAGACCCCCCACTTAACCCT--------

>EF190926_pcv2b

ATGACGTATCCAAGGAGGCGTTACCGGAGAAGAAGACTCCGCCCCCGCAGCCATCTTGGCCAGATCCTCCGCCGCCG
CCCCTGGCTCCTCCACCCCCGCCACCGTTACCGCTGGAGAAGGAAAAATGGCATCTTCAACACCCGCCTCTCCCGCACC
TTCGGATATACTATCAAGCGAACCACAGTCAAAACGCCCTCCTGGGCGGTGGACATGATGAGATTCAATATTAATGAC
TTTCTTCCCCCAGGAGGGGGCTCAAACCCCCGCTCTGTGCCCTTTGAATACTACAGAATAAGAAAGGTTAAGGTTGAA
TTCTGGCCCTGCTCCCCGATCACCCAGGGTGACAGGGGAGTGGGCTCCAGTGCTGTTATTCTAGATGATAACTTTGTA
ACAAAGGCCACAGCCCTCACCTATGACCCCTATGTAAACTACTCCTCCCGCCATACCATAACCCAGCCCTTCTCCTACCA
CTCCCGCTACTTTACCCCCAAACCTGTCCTAGATTCCACTATTGATTACTTCCAACCAAACAACAAAAGAAATCAGCTGT
GGCTGAGACTACAAACTGCTGGAAATGTAGACCACGTAGGCCTCGGCACTGCGTTCGAAAACAGTATATACGACCAG
GAATACAATATCCGTGTAACCATGTATGTACAATTCAGAGAATTTAATCTTAAAGACCCCCCACTTAACCCT--------

>FJ644559_pcv2b

ATGACGTATCCAAGGAGGCGTTACCGGAGAAGAAGACACCGCCCCCGCAGCCATCTTGGCCAGATCCTCCGCCGCCG
CCCCTGGCTCCTCCACCCCCGCCACCGTTACCGCTGGAGAAGGAGAAATGGCATCTTCAACACCCGCCTCTCCCGCACC
TTCGGATATACTATCAAGCGAACCACAGTCAAAACGCCCTCCTGGGCGGTGGACATGATGAGATTCAATATTAATGAC
TTTCTTCCCCCAGGAGGGGGCTCAAACCCCCGCTCTGTGCCCTTTGAATACTACAGAATAAGAAAGGTTAAGGTTGAA
TTCTGGCCCTGCTCCCCGATCACCCAGGGTGACAGGGGAGTGGGCTCCAGTGCTGTTATTCTAGATGATAACTTTGTA
ACAAAGGCCACAGCCCTCACCTATGACCCCTATGTAAACTACTCCTCCCGCCATACCATAACCCAGCCCTTCTCCTACCA
CTCCCGCTACTTTACCCCCAAACCTGTCCTAGATTCCACTATTGATTACTTCCAACCAAACAACAAAAGAAATCAGCTGT
GGCTGAGACTACAAACTGCTGGAAATGTAGACCACGTAGGCCTCGGCACTGCGTTCGAAAACAGTATATACGACCAG
GAATACAATATCCGTGTAACCATGTATGTACAATTCAGAGAATTTAATCTTAAAGACCCCCCACTTAACCCT--------

>HQ113120_pcv2b

ATGACGTATCCAAGGAGGCGTTACCGGAGAAGAAGACACCGCCCCCGCAGCCATCTTGGCCAGATCCTCCGCCGCCG
CCCCTGGCTCCTCCACCCCCGCCACCGTTACCGCTGGAGAAGGAAAAATGGCATCTTCAACACCCGCCTCTCCCGCACC
TTCGGATATACTATCAAGCGAACCACAGTCAAAACGCCCTCCTGGGCGGTGGACATGATGAGATTCAATATTAATGAC
TTTCTTCCCCCAGGAGGGGGCTCAAACCCCCGCTCTGTGCCCTTTGAATACTACAGAATAAGAAAGGTTAAGGTTGAA
CTCTGGCCCTGCTCCCCGATCACCCAGGGTGACAGGGGAGTGGGCTCCAGTGCTGTTATTCTAGATGATAACTTTGTA
ACAAAGGCCACAGCCCTCACCTATGACCCCTATGTAAACTACTCCTCCCGCCATACCATAACCCAGCCCTTCTCCTACCA
CTCCCGCTACTTTACCCCCAAACCTGTCCTAGATTCCACTATTGATTACTTCCAACCAAACAACAAAAGAAATCAGCTGT
GGCTGAGACTACAAACTGCTGGAAATGTAGACCACGTAGGCCTCGGCACTGCGTTCGAAAACAGTATATACGACCAG
GAATACAATATCCGTGTAACCATGTATGTACAATTCAGAGAATTTAATCTTAAAGACCCCCCACTTAACCCT--------

>EF190943_pcv2b

ATGACGTATCCAAGGAGGCGTTACCGGAGAAGAAGACACCGCCCCCGCAGCCATCTTGGCCAGATCCTCCGCCGCCG
CCCCTGGCTCCTCCACCCCCGCCACCGTTACCGCTGGAGAAGGAAAAATGGCATCTTCAACACCCGCCTCTCCCGCACC
TTCGGATATACTATCAAGCGAACCACAGTCAAAACGCCCTCCTGGGCGGTGGACATGATGAGATTCAATATTAATGAC
TTTCTTCCCCCAGGAGGGGGCTCAAACCCCCGCTCTGTGCCCTTTGAATACTACAGAATAAGAAAGGTTAAGGTTGAA
TTCTGGCCCTGCTCCCCGATCACCCAGGGTGACAGGGGAGTGGGCTCCAGTGCTGTTATTCTAGATGATAACTTTGTA
ACAAAGGCCACAGCCCTCACCTATGACCCCTATGTAAACTACTCCTCCCGCCATACCATAACCCAGCCCTTCTCCTACCA
CTCCCGCTACTTTACCCCCAAACCTGTCCTAGATTCCACTATTGATTACTTCCAACCAAACAACAAAAGAAATCAGCTGT
GGCTGAGACTACAAACTGCTGGAAATGTAGACCACGTAGGCCTCGGCACTGCGTTCGAAAACAGTATATACGACCAG
GAATACAATATCCGTGTAACCATGTATGTACAATTCAGAGAATTTAATCTTAAAGACCCCCCACTTAACCCT--------

>EU521707_pcv2b

ATGACGTATCCAAGGAGGCGTTACCGGAGAAGAAGACACCGCCCCCGCAGCCATCTTGGCCAGATCCTCCGCCGCCG
CCCCTGGCTCCTCCACCCCCGCCACCGTTACCGCTGGAGAAGGAAAAATGGCATCTTCAACACCCGCCTCTCCCGCACC
TTCGGATATACTATCAAGCGAACCACAGTCAAAACGCCCTCCTGGGCGGTGGACATGATGAGATTCAATATTAATGAC
TTTCTTCCCCCAGGAGGGGGCTCAAACCCCCGCTCTGTGCCCTTTGAATACTACAGAATAAGAAAGGTTAAGGTTGAA
TTCTGGCCCTGCTCCCCGATCACCCAGGGTGACAGGGGAGTGGGCTCCAGTGCTGTTATTCTAGATGATAACTTTGTA
ACAAAGGCCGCAGCCCTCACCTATGACCCCTATGTAAACTACTCCTCCCGCCATACCATAACCCAGCCCTTCTCCTACCA
CTCCCGCTACTTTACCCCCAAACCTGTCCTAGATTCCACTATTGATTACTTCCAACCAAACAACAAAAGAAATCAGCTGT
GGCTGAGACTACAAACTGCTGGAAATGTAGACCACGTAGGCCTCGGCACTGCGTTCGAAAACAGTATATACGACCAG
GAATACAATATCCGTGTAACCATGTATGTACAATTCAGAGAATTTAATCTTAAAGACCCCCCACTTAACCCT--------

>EF190942_pcv2b

ATGACGTATCCAAGGAGGCGTTACCGGAGAAGAAGACACCGCCCCCGCAGCCATCTTGGCCAGATCCTCCGCCGCCG
CCCCTGGCTCCTCCACCCCCGCCACCGTTACCGCTGGAGAAGGAAAAATGGCATCTTCAACACCCGCCTCTCCCGCACC
TTCGGATATACTATCAAGCGAACCACAGTCAAAACGCCCTCCTGGGCGGTGGACATGATGAGATTCAATATTAATGAC
TTTCTTCCCCCAGGAGGGGGCTCAAACCCCCGCTCTGTGCCCTTTGAATACTACAGAATAAGAAAGGTTAAGGTTGAA
TTCTGGCCCTGCTCCCCGATCACCCAGGGTGACAGGGGAGTGGGCTCCAGTGCTGTTATTCTAGATGATAACTTTGTA
ACAAAGGCCACAGCCCTCACCTATGACCCCTATGTAAACTACTCCTCCCGCCATACCATAACCCAGCCCTTCTCCTACCA
CTCCCGCTACTTTACCCCCAAACCTGTCCTAGATTCCACTATTGATTACTTCCAACCAAACAACAAAAGAAATCAGCTGT
GGCTGAGACTACAAACTGCTGGAAATGTAGACCACGTAGGCCTCGGCACTGCGTTCGAAAACAGTATATACGACCAG
GAATACAATATCCGTGTAACCATGTATGTACAATTCAGAGAATTTAATCTTAAAGACCCCCCACTTAACCCT--------

>EF190937_pcv2b

ATGACGTATCCAAGGAGGCGTTACCGGAGAAGAAGACACCGCCCCCGCAGCCATCTTGGCCAGATCCTCCGCCGCCG
CCCCTGGCTCCTCCACCCCCGCCACCGTTACCGCTGGAGAAGGAAAAATGGCATCTTCAACACCCGCCTCTCCCGCACC
TTCGGATATACTATCAAGCGAACCACAGTCAAAACGCCCTCCTGGGCGGTGGACATGATGAGATTCAATATTAATGAC
TTTCTTCCCCCAGGAGGGGGCTCAAACCCCCGCTCTGTGCCCTTTGAATACTACAGAATAAGAAAGGTTAAGGTTGAA
TTCTGGCCCTGCTCCCCGATCACCCAGGGTGACAGGGGAGTGGGCTCCAGTGCTGTTATTCTAGATGATAACTTTGTA
ACAAAGGCCACAGCCCTCACCTATGACCCCTATGTAAACTACTCCTCCCGCCATACCATAACCCAGCCCTTCTCCTACCA
CTCCCGCTACTTTACCCCCAAACCTGTCCTAGATTCCACTATTGATTACTTCCAACCAAACAACAAAAGAAATCAGCTGT
GGCTGAGACTACAAACTGCTGGAAATGTAGACCACGTAGGCCTCGGCACTGCGTTCGAAAACAGTATATACGACCAG
GAATACAATATCCGTGTAACCATGTATGTACAATTCAGAGAATTTAATCTTAAAGACCCCCCACTTAACCCT--------

>EF190939_pcv2b

ATGACGTATCCAAGGAGGCGTTACCGGAGAAGAAGACACCGCCCCCGCAGCCATCTTGGCCAGATCCTCCGCCGCCG
CCCCTGGCTCCTCCACCCCCGCCACCGTTACCGCTGGAGAAGGAAAAATGGCATCTTCAACACCCGCCTCTCCCGCACC
TTCGGATATACTATCAAGCGAACCACAGTCAAAACGCCCTCCTGGGCGGTGGACATGATGAGATTCAATATTAATGAC
TTTCTTCCCCCAGGAGGGGGCTCAAACCCCCGCTCTGTGCCCTTTGAATACTACAGAATAAGAAAGGTTAAGGTTGAA
TTCTGGCCCTGCTCCCCGATCACCCAGGGTGACAGGGGAGTGGGCTCCAGTGCTGTTATTCTAGATGATAACTTTGTA
ACAAAGGCCACAGCCCTCACCTATGACCCCTATGTAAACTACTCCTCCCGCCATACCATAACCCAGCCCTTCTCCTACCA
CTCCCGCTACTTTACCCCCAAACCTGTCCTAGATTCCACTATTGATTACTTCCAACCAAACAACAAAAGAAATCAGCTGT
GGCTGAGACTACAAACTGCTGGAAATGTAGACCACGTAGGCCTCGGCACTGCGTTCGAAAACAGTATATACGACCAG
GAATACAATATCCGTGTAACCATGTATGTACAATTCAGAGAATTTAATCTTAAAGACCCCCCACTTAACCCT--------

>EU257512_pcv2b

ATGACGTATCCAAGGAGGCGTTACCGGAGAAGAAGACACCGCCCCCGCAGCCATCTTGGCCAGATCCTCCGCCGCCG
CCCCTGGCTCCTCCACCCCCGCCACCGTTACCGCTGGAGAAGGAAAAATGGCATCTTCAACACCCGCCTCTCCCGCACC
TTCGGATATACTATCAAGCGAACCACAGTCAAAACGCCCTCCTGGGCGGTGGACATGATGAGATTCAATATTAATGAC
TTTCTTCCCCCAGGAGGGGGCTCAAACCCCCGCTCTGTGCCCTTTGAATACTACAGAATAAGAAAGGTTAAGGTTGAA
TTCTGGCCCTGCTCCCCGATCACCCAGGGTGACAGAGGAGTGGGCTCCAGTGCTGTTATTCTAGATGATAACTTTGTA
ACAAAGGCCACAGCCCTCACCTATGACCCCTATGTAAACTACTCCTCCCGCCATACCATAACCCAGCCCTTCTCCTACCA
CTCCCGCTACTTTACCCCCAAACCTGTCCTAGATTCCACTATTGATTACTTCCAACCAAACAACAAAAGAAATCAGCTGT
GGCTGAGACTACAAACTGCTGGAAATGTAGACCACGTAGGCCTCGGCACTGCGTTCGAAAACAGTATATACGACCAG
GAATACAATATCCGTGTAACCATGTATGTACAATTCAGAGAATTTAATCTTAAAGACCCCCCACTTAACCCT--------

>EF190923_pcv2b

ATGACGTATCCAAGGAGGCGTTACCGGAGAAGAAGACACCGCCCCCGCAGCCATCTTGGCCAGATCCTCCGCCGCCG
CCCCTGGCTCCTCCACCCCCGCCACCGTTACCGCTGGAGAAGGAAAAATGGCATCTTCAACACCCGCCTCTCCCGCACC
TTCGGATATACTATCAAGCGAACCACAGTCAAAACGCCCTCCTGGGCGGTGGACATGATGAGATTCAATATTAATGAC
TTTCTTCCCCCAGGAGGGGGCTCAAACCCCCGCTCTGTGCCCTTTGAATACTACAGAATAAGAAAGGTTAAGGTTGAA
TTCTGGCCCTGCTCCCCGATCACCCAGGGTGACAGGGGAGTGGGCTCCAGTGCTGTTATTCTAGATGATAACTTTGTA
ACAAAGGCCACAGCCCTCACCTATGACCCCTATGTAAACTACTCCTCCCGCCATACCATAACCCAGCCCTTCTCCTACCA
CTCCCGCTACTTTACCCCCAAACCTGTCCTAGATTCCACTATTGATTACTTCCAACCAAACAACAAAAGAAATCAGCTGT
GGCTGAGACTACAAACTGCTGGAAATGTAGACCACGTAGGCCTCGGCACTGCGTTCGAAAACAGTATATACGACCAG
GAATACAATATCCGTGTAACCATGTATGTACAATTCAGAGAATTTAATCTTAAAGACCCCCCACTTAACCCT--------

>HQ113121_pcv2b

ATGACGTATCCAAGGAGGCGTTACCGGAGAAGAAGACACCGCCCCCGCAGCCATCTTGGCCAGATCCTCCGCCGCCG
CCCCTGGCTCCTCCACCCCCGCCACCGTTACCGCTGGAGAAGGAAAAATGGCATCTTCAACACCCGCCTCTCCCGCACC
TTCGGATATACTATCAAGCGAACCACAGTCAAAACGCCCTCCTGGGCGGTGGACATGATGAGATTCAATATTAATGAC
TTTCTTCCCCCAGGAGGGGGCTCAAACCCCCGCTCTGTGCCCTTTGAATACTACAGAATAAGAAAGGTTAAGGTTGAA
TTCTGGCCCTGCTCCCCGATCACCCAGGGTGACAGGGGAGTGGGCTCCAGTGCTGTTATTCTAGATGATAACTTTGTA
ACAAAGGCCACAGCCCTCACCTATGACCCCTATGTAAACTACTCCTCCCGCCATACCATAACCCAGCCCTTCTCCTACCA
CTCCCGCTACTTTACCCCCAAACCTGTCCTAGATTCCACTATTGATTACTTCCAACCAAACAACAAAAGAAATCAGCTGT
GGCTGAGACTACAAACTGCTGGAAATGTAGACCACGTAGGCCTCGGCACTGCGTTCGAAAACAGTATATACGACCAG
GAATACAATATCCGTGTAACCATGTATGTACAATTCAGAGAATTTAATCTTAAAGACCCCCCACTTAACCCT--------

>FJ384966_pcv2b

ATGACGTATCCAAGGAGGCGTTACCGGAGAAGAAGACACCGCCCCCGCAGCCATCTTGGCCAGATCCTCCGCCGCCG
CCCCTGGCTCCTCCACCCCCGCCACCGTTACCGCTGGAGAAGGAAAAATGGCATCTTCAACACCCGCCTCTCCCGCACC
TTCGGATATACTATCAAGCGAACCACAGTCAAAACGCCCTCCTGGGCGGTGGACATGATGAGATTCAATATTAATGAC
TTTCTTCCCCCAGGAGGGGGCTCAAACCCCCGCTCTGTGCCCTTTGAATACTACAGAATAAGAAAGGTTAAGGTTGAA
TTCTGGCCCTGCTCCCCGATCACCCAGGGTGACAGGGGAGTGGGCTCCAGTGCTGTTATTCTAGATGATAACTTTGTA
ACAAAGGCCACAGCCCTCACCTATGACCCCTATGTAAACTACTCCTCCCGCCATACCATAACCCAGCCCTTCTCCTACCA
CTCCCGCTACTTTACCCCCAAACCTGTCCTAGATTCCACTATTGATTACTTCCAACCAAACAACAAAAGAAATCAGCTGT
GGCTGAGACTACAAACTGCTGGAAATGTAGACCACGTAGGCCTCGGCACTGCGTTCGAAAACAGTATATACGACCAG
GAATACAATATCCGTGTAACCATGTATGTACAATTCAGAGAATTTAATCTTAAAGACCCCCCACTTAACCCT--------

>FJ384967_pcv2b

ATGACGTATCCAAGGAGGCGTTACCGGAGAAGAAGACACCGCCCCCGCAGCCATCTTGGCCAGATCCTCCGCCGCCG
CCCCTGGCTCCTCCACCCCCGCCACCGTTACCGCTGGAGAAGGAAAAATGGCATCTTCAACACCCGCCTCTCCCGCACC
TTCGGATATACTATCAAGCGAACCACAGTCAAAACGCCCTCCTGGGCGGTGGACATGATGAGATTCAATATTAATGAC
TTTCTTCCCCCAGGAGGGGGCTCAAACCCCCGCTCTGTGCCCTTTGAATACTACAGAATAAGAAAGGTTAAGGTTGAA
TTCTGGCCCTGCTCCCCGATCACCCAGGGTGACAGGGGAGTGGGCTCCAGTGCTGTTATTCTAGATGATAACTTTGTA
ACAAAGGCCACAGCCCTCACCTATGACCCCTATGTAAACTACTCCTCCCGCCATACCATAACCCAGCCCTTCTCCTACCA
CTCCCGCTACTTTACCCCCAAACCTGTCCTAGATTCCACTATTGATTACTTCCAACCAAACAACAAAAGAAATCAGCTGT
GGCTGAGACTACAAACTGCTGGAAATGTAGACCACGTAGGCCTCGGCACTGCGTTCGAAAACAGTATATACGACCAG
GAATACAATATCCGTGTAACCATGTATGTACAATTCAGAGAATTTAATCTTAAAGACCCCCCACTTAACCCT--------

>AY604430_pcv2b

ATGACGTATCCAAGGAGGCGTTACCGGAGAAGAAGACACCGCCCCCGCAGCCATCTTGGCCAGATCCTCCGCCGCCG
CCCCTGGCTCCTCCACCCCCGCCACCGTTACCGCTGGAGAAGGAAAAATGGCATCTTCAACACCCGCCTCTCCCGCACC
TTCGGATATACTATCAAGCGAACCACAGTCAAAACGCCCTCCTGGGCGGTGGACATGATGAGATTCAATATTAATGAC
TTTCTTCCCCCAGGAGGGGGCTCAAACCCCCGCTCTGTGCCCTTTGAATACTACAGAATAAGAAAGGTTAAGGTTGAA
TTCTGGCCCTGCTCCCCGATCACCCAGGGTGACAGGGGAGTGGGCTCCAGTGCTGTTATTCTAGATGATAACTTTGTA
ACAAAGGCCACAGCCCTCACCTATGACCCCTATGTAAACTACTCCTCCCGCCATACCATAACCCAGCCCTTCTCCTACCA
CTCCCGCTACTTTACCCCCAAACCTGTCCTAGATTCCACTATTGATTACTTCCAACCAAACAACAAAAGAAATCAGCTGT
GGCTGAGACTACAAACTGCTGGAAATGTAGACCACGTAGGCCTCGGCACTGCGTTCGAAAACAGTATATACGACCAG
GAATACAATATCCGTGTAACCATGTATGTACAATTCAGAGAATTTAATCTTAAAGACCCCCCACTTAACCCT--------

>EF190933_pcv2b

ATGACGTATCCAAGGAGGCGTTACCGGAGAAGAAGACACCGCCCCCGCAGCCATCTTGGCCAGATCCTCCGCCGCCG
CCCCTGGCTCCTCCACCCCCGCCACCGTTACCGCTGGAGAAGGAAAAATGGCATCTTCAACACCCGCCTCTCCCGCACC
TTCGGATATACTATCAAGCGAACCACAGTCAAAACGCCCTCCTGGGCGGTGGACATGATGAGATTCAATATTAATGAC
TTTCTTCCCCCAGGAGGGGGCTCAAACCCCCGCTCTGTGCCCTTTGAATACTACAGAATAAGAAAGGTTAAGGTTGAA
TTCTGGCCCTGCTCCCCGATCACCCAGGGTGACAGGGGAGTGGGCTCCAGTGCTGTTATTCTAGATGATAACTTTGTA
ACAAAGGCCACAGCCCTCACCTATGACCCCTATGTAAACTACTCCTCCCGCCATACCATAACCCAGCCCTTCTCCTACCA
CTCCCGCTACTTTACCCCCAAACCTGTCCTAGATTCCACTATTGATTACTTCCAACCAAACAACAAAAGAAATCAGCTGT
GGCTGAGACTACAAACTGCTGGAAATGTAGACCACGTAGGCCTCGGCACTGCGTTCGAAAACAGTATATACGACCAG
GAATACAATATCCGTGTAACCATGTATGTACAATTCAGAGAATTTAATCTTAAAGACCCCCCACTTAACCCT--------

>GU083582_pcv2b

ATGACGTATCCAAGGAGGCGTTACCGGAGAAGAAGACACCGCCCCCGCAGCCATCTTGGCCAGATCCTCCGCCGCCG
CCCCTGGCTCCTCCACCCCCGCAACCGTTACCGCTGGAGAAGGAAAAATGGCATCTTCAACACCCGCCTCTCCCGCACC
TTCGGATATACTATCAAGCGAACCACAGTCAAAACGCCCTCCTGGGCGGTGGACATGATGAGATTCAATATTAATGAC
TTTATTCCCCCAGGAGGGGGCACAAACCCCCGCTCTGTGCCCTTTGAATACTACAGAATAAGAAAGGTTAAGGTTGAA
TTCTGGCCCTGCTCCCCGATCACCCAGGGTGACAGGGGAGTGGGCTCCAGTGCTGTTATTCTAGATGATAACTTTGTA
CCAAAGACCACAGCCCTCACCTATGACCCCTATGTAAACTACTCCTCCCGCCATACCATAACCCAGCCCTTCTCCTACCA
CTCCCGCTACTTTACCCCCAAACCTGTCCTAGATTCCACTATTGATTACTTCCAACCAAACAACAAAAGAAATCAGCTGT
GGCTGAGACTACAAACTGCTGGAAATGTAGACCACGTAGGCCTCGGCACTGCGTTCGAAAACAGTATATACGACCAG
GAATACAATATCCGTGTAACCATGTATGTACAATTCAGAGAATTTAATCTTAAAGACCCCCCACTTAAACCT--------

>FJ644926_pcv2b

ATGACGTATCCAAGGAGGCGTTACCGGAGAAGAAGACACCGCCCCCGCAGCCATCTTGGCCAGATCCTCCGCCGCCG
CCCCTGGCTCCTCCACCCCCGCCACCGTTACCGCTGGAGAAGGAAAAATGGCATCTTCAACACCCGCCTCTCCCGCACC
TTCGGATATACTATCAAGCGAACCACAGTCAAAACGCCCTCCTGGGCGGTGGACATGATGAGATTCAATATTAATGAC
TTTATTCCCCCAGGAGGGGGCTCAAACCCCCGCTCTGTGCCCTTTGAATACTACAGAATAAGAAAGGTTAAGGTTGAA
TTCTGGCCCTGCTCCCCGATCACCCAGGGTGACAGGGGAGTGGGCTCCAGTGCTGTTATTCTAGATGATAACTTTGTA
ACAAAGGCCACAGCCCTCACCTATGACCCCTATGTAAACTACTCCTCCCGCCATACCATAACCCAGCCCTTCTCCTACCA
CTCCCGCTACTTTACCCCCAAACCTGTCCTAGATTCCACTATTGATTACTTCCAACCAAACAACAAAAGAAATCAGCTGT
GGCTGAGGCTACAAACTGCTGGAAATGTAGACCACGTAGGCCTCGGCACTGCGTTCGAAAACAGTATATACGACCAG
GAATACAATATCCGTGTAACCATGTATGTACAATTCAGAGAATTTAATCTTAAAGACCCCCCACTTAACCCT--------

>FJ384965_pcv2b

ATGACGTATCCAAGGAGGCGTTACCGGAGAAGAAGACACCGCCCCCGCAGCCATCTTGGCCAGATCCTCCGCCGCCG
CCCCTGGCTCCTCCACCCCCGCCACCGTTACCGCTGGAGAAGGAAAAATGGCATCTTCAACACCCGCCTCTCCCGCACC
TTCGGATATACTATCAAGCGAACCACAGTCAAAACGCCCTCCTGGGCGGTGGACATGATGAGATTCAATATTAATGAC
TTTCTTCCCCCAGGAGGGGGCTCAAACCCCCGCTCTGTGCCCTTTGAATACTACAGAATAAGAAAGGTTAAGGTTGAA
TTCTGGCCCTGCTCCCCGATCACCCAGGGTGACAGGGGAGTGGGCTCCAGTGCTGTTATTCTAGATGATAACTTTGTA
ACAAAGGCCACAGCCCTCACCTATGACCCCTATGTAAACTACTCCTCCCGCCATACCATAACCCAGCCCTTCTCCTACCA
CTCCCGCTACTTTACCCCCAAACCTGTCCTAGATTCCACTATTGATTACTTCCAACCAAACAACAAAAGAAATCAGCTGT
GGCTGAGACTACAAACTGCTGGAAATGTAGACCACGTAGGCCTCGGCACTGCGTTCGAAAACAGTATATACGACCAG
GAATACAATATCCGTGTAACCATGTATGTACAATTCAGAGAATTTAATCTTAAAGACCCCCCACTTAACCCT--------

>EU257514_pcv2b

ATGACGTATCCAAGGAGGCGTTACCGGAGAAGAAGACACCGCCCCCGCAGCCATCTTGGCCAGATCCTCCGCCGCCG
CCCCTGGCTCCTCCACCCCCGCCACCGTTACCGCTGGAGAAGGAAAAATGGCATCTTCAACACCCGCCTCTCCCGCACC
TTCGGATATACTATCAAGCGAACCACAGTCAAAACGCCCTCCTGGGCGGTGGACATGATGAGATTCAATATTAATGAC
TTTCTTCCCCCAGGAGGGGGCTCAAACCCCCGCTCTGTGCCCTTTGAATACTACAGAATAAGAAAGGTTAAGGTTGAA
TTCTGGCCCTGCTCCCCGATCACCCAGGGTGACAGGGGAGTGGGCTCCAGTGCTGTTATTCTAGATGATAACTTTGTA
ACAAAGGCCACAGCCCTCACCTATGACCCCTATGTAAACTACTCCTCCCGCCATACCATAACCCAGCCCTTCTCCTACCA
CTCCCGCTACTTTACCCCCAAACCTGTCCTAGATTCCACTATTGATTACTTCCAACCAAACAACAAAAGAAATCAGCTGT
GGCTGAGACTACAAACTGCTGGAAATGTAGACCACGTAGGCCTCGGCACTGCGTTCGAAAACAGTATATACGACCAG
GAATACAATATCCGTGTAACCATGTATGTACAATTCAGAGAATTTAATCTTAAAGACCCCCCACTTAACCCT--------

>EF197987_pcv2b

ATGACGTATCCAAGGAGGCGTTACCGGAGAAGAAGACACCGCCCCCGCAGCCATCTTGGCCAGATCCTCCGCCGCCG
CCCCTGGCTCCTCCACCCCCGCCACCGTTACCGCTGGAGAAGGAAAAATGGCATCTTCAACACCCGCCTCTCCCGCACC
TTCGGATATACTATCAAGCGAACCACAGTCAAAACGCCCTCCTGGGCGGTGGACATGATGAGATTCAATATTAATGAC
TTTCTTCCCCCAGGAGGGGGCTCAAACCCCCGCTCTGTGCCCTTTGAATACTACAGAATAAGAAAGGTTAAGGTTGAA
TTCTGGCCCTGCTCCCCGATCACCCAGGGTGACAGGGGAGTGGGCTCCAGTGCTGTTATTCTAGATGATAACTTTGTA
ACAAAGGCCACAGCCCTCACCTATGACCCCTATGTAAACTACTCCTCCCGCCATACCATAACCCAGCCCTTCTCCTACCA
CTCCCGCTACTTTACCCCCAAACCTGTCCTAGATTCCACTATTGATTACTTCCAACCAAACAACAAAAGAAATCAGCTGT
GGCTGAGACTACAAACTGCTGGAAATGTAGACCACGTAGGCCTCGGCACTGCGTTCGAAAACAGTATATACGACCAG
GAATACAATATCCGTGTAACCATGTATGTACAATTCAGAGAATTTAATCTTAAAGACCCCCCACTTAACCCT--------

>AY686764_pcv2b

ATGACGTATCCAAGGAGGCGTTACCGGAGAAGAAGACACCGCCCCCGCAGCCATCTTGGCCAGATCCTCCGCCGCCG
CCCCTGGCTCCTCCACCCCCGCCACCGTTACCGCTGGAGAAGGAAAAATGGCATCTTCAACACCCGCCTCTCCCGCACC
TTCGGATATACTATCAAGCGAACCACAGTCAAAACGCCCTCCTGGGCGGTGGACATGATGAGATTCAATATTAATGAC
TTTCTTCCCCCAGGAGGGGGCTCAAACCCCCGCTCTGTGCCCTTTGAATACTACAGAATAAGAAAGGTTAAGGTTGAA
TTCTGGCCCTGCTCCCCGATCACCCAGGGTGACAGGGGAGTGGGCTCCAGTGCTGTTATTCTAGATGATAACTTTGTA
ACAAAGGCCACAGCCCTCACCTATGACCCCTATGTAAACTACTCCTCCCGCCATACCATAACCCAGCCCTTCTCCTACCA
CTCCCGCTACTTTACCCCCAAACCTGTCCTAGATTCCACTATTGATTACTTCCAACCAAACAACAAAAGAAATCAGCTGT
GGCTGAGACTACAAACTGCTGGAAATGTAGACCACGTAGGCCTCGGCACTGCATTCGAAAACAGTATATACGACCAG
GAATACAATATCCGTGTAACCATGTATGTACAATTCAGAGAATTTAATCTTAAAGACCCCCCACTTAACCCT--------

>HM009329_pcv2b

ATGACGTATCCAAGGAGGCGTTACCGGAGAAGAAGACACCGCCCCCGCAGCCATCTTGGCCAGATCCTCCGCCGCCG
CCCCTGGCTCATCCACCCCCGCCACCGTTACCGCTGGAGAAGGAAAAATGGCATCTTCAACACCCGCCTCTCCCGCACC
TTCGGATATACTATCAAGCGAACCACAGTCAAAACGCCCTCCTGGGCGGTGGACATGATGAGATTCAATATTAATGAC
TTTCTTCCCCCAGGAGGGGGCTCAAACCCCCGCTCTGTGCCCTTTGAATACTACAGAATAAGAAAGGTTAAGGTTGAA
TTCTGGCCCTGCTCCCCGATCACCCAGGGTGACAGGGGAGTGGGCTCCAGTGCTGTTATTCTAGATGATAACTTTGTA
ACAAAGGCCACAGCCCTCACCTATGACCCCTATGTAAACTACTCCTCCCGCCATACCATAACCCAGCCCTTCTCCTACCA
CTCCCGCTACTTTACCCCCAAACCTGTCCTAGATTCCACTATTGATTACTTCCAACCAAACAACAAAAGAAATCAGCTGT
GGCTGAGACTACAAACTGCTGGAAATGTAGACCACGTAGGCCTCGGCACTGCATTCGAAAACAGTATATACGACCAG
GAATACAATATCCGTGTAACCATGTATGTACAATTCAGAGAATTTAATCTTAAAGACCCCCCACTTAACCCT--------

>FJ384969_pcv2b

ATGACGTATCCAAGGAGGCGTTACCGGAGAAGAAGACACCGCCCCCGCAGCCATCTTGGCCAGATCCTCCGCCGCCG
CCCCTGGCTCCTCCACCCCCGCCACCGTTACCGCTGGAGAAGGAAAAATGGCATCTTCAACACCCGCCTCTCCCGCACC
TTCGGATATACTATCAAGCGAACCACAGTCAAAACACCCTCCTGGGCGGTGGACATGATGAGATTCAATATTAATGAC
TTTCTTCCCCCAGGAGGGGGCTCAAACCCCCGCTCTGTGCCCTTTGAATACTACAGAATAAGAAAGGTTAAGGTTGAA
TTCTGGCCCTGCTCCCCGATCACCCAGGGTGACAGGGGAGTGGGCTCCAGTGCTGTTATTCTAGATGATAACTTTGTA
ACAAAGGCCACAGCCCTCACCTATGACCCCTATGTAAACTACTCCTCCCGCCATACCATAACCCAGCCCTTCTCCTACCA
CTCCCGCTACTTTACCCCCAAACCTGTCCTAGATTCCACTATTGATTACTTCCAACCAAACAACAAAAGAAATCAGCTGT
GGCTGAGACTACAAACTGCTGGAAATGTAGACCACGTAGGCCTCGGCACTGCTTTCGAAAACAGTATATACGACCAG
GAATACAATATCCGTGTAACCATGTATGTACAATTCAGAGAATTTAATCTTAAAGACCCCCCACTTAACCCT--------

>JN989556_pcv2b

ATGACGTATCCAAGGAGGCGTTACCGGAGAAGAAGACACCGCCCCCGCAGCCATCTTGGCCAGATCCTCCGCCGCCG
CCCCTGGCTCCTCCACCCCCGCCACCGTTACCGCTGGAGAAGGAAAAATGGCATCTTCAACACCCGCCTCTCCCGCACC
TTCGGATATACTATCAAGCGAACCACAGTCAAAACACCCTCCTGGGCGGTGGACATGATGAGATTCAATATTAATGAC
TTTCTTCCCCCAGGAGGGGGCTCAAACCCCCGCTCTGTGCCCTTTGAATACTACAGAATAAGAAAGGTTAAGGTTGAA
TTCTGGCCCTGCTCCCCGATCACCCAGGGTGACAGGGGAGTGGGCTCCAGTGCTGTTATTCTAGATGATAACTTTGTA
ACAAAGGCCACAGCCCTCACCTATGACCCCTATGTAAACTACTCCTCCCGCCATACCATAACCCAGCCCTTCCCCTACCA
CTCCCGCTACTTTACCCCCAAACCTGTCCTAGATTCCACTATTGATTACTTCCAACCGAACAACAAAAGAAATCAGCTGT
GGCTGAGACTACAAACTGCTGGAAATGTAGACCACGTAGGCCTCGGCACTGCTTTCGAAAACAGTATATACGACCAG
GAATACAATATCCGTGTAACCATGTATGTACAATTCAGAGAATTTAATCTTAAAGACCCCCCACTTAACCCT--------

>JN989553_pcv2b

ATGACGTATCCAAGGAGGCGTTACCGGAGAAGAAGACACCGCCCCCGCAGCCATCTTGGCCAGATCCTCCGCCGCCG
CCCCTGGCTCCTCCACCCCCGCCACCGTTACCGCTGGAGAAGGAAAAATGGCATCTTCAACACCCGCCTCTCCCGCACC
TTCGGATATACTATCAAGCGAACCACAGTCAAAACACCCTCCTGGGCGGTGGACATGATGAGATTCAATATTAATGAC
TTTCTTCCCCCAGGAGGGGGCTCAAACCCCCGCTCTGTGCCCTTTGAATGCTACAGAATAAGAAAGGTTAAGGTTGAA
TTCTGGCCCTGCTCCCCGATCACCCAGGGTGACAGGGGAGTGGGCTCCAGTGCTGTTATTCTAGATGATAACTTTGTA
ACAAAGGCCACAGCCCTCACCTATGACCCCTATGTAAACTACTCCTCCCGCCATACCATAACCCAGCCCTTCTCCTACCA
CTCCCGCTACTTTACCCCCAAACCTGTCCTAGATTCCACTATTGATTACTTCCAACCAAACAACAAAAGAAATCAGCTGT
GGCTGAGACTACAAACTGCTGGAAATGTAGACCACGTAGGCCTCGGCACTGCTTTCGAAAACGGTATATACGACCGG
GAATACAATATCCGTGTAACCATGTATGTACGATTCAGAGAATTTAATCTTAAAGACCCCCCACTTAACCCT--------

>JN989554_pcv2b

ATGACGTATCCAAGGAGGCGTTACCGGAGAAGAAGACACCGCCCCCGCAGCCATCTTGGCCAGATCCTCCGCCGCCG
CCCCTGGCTCCTCCACCCCCGCCACCGTTACCGCTGGAGAAGGAAAAATGGCATCTTCAACACCCGCCTCTCCCGCACC
TTCGGATATACTATCAAGCGAACCACAGTCAAAACACCCTCCTGGGCGGTGGACATGATGAGATTCAATATTAATGAC
TTTCTTCCCCCAGGAGGGGGCTCAAACCCCCGCTCTGTGCCCTTTGAATACTACAGAATAAGAAAGGTTAAGGTTGAA
TTCTGGCCCTGCTCCCCGATCACCCAGGGTGACAGGGGAGTGGGCTCCAGTGCTGTTATTCTAGATGATAACTTTGTA
ACAAAGGCCACAGCCCTCACCTATGACCCCTATGTAAACTACTCCTCCCGCCATACCATAACCCAGCCCTTCTCCTACCA
CTCCCGCTACTTTACCCCCAAACCTGTCCTAGATTCCACTATTGATTACTTCCAACCAAACAACAAAAGAAATCAGCTGT
GGCTGAGACTACAAACTGCTGGAAATGTAGACCACGTAGGCCTCGGCACTGCTTTCGAAAACAGTATATACGACCGG
GAATACAATATCCGTGTAACCATGTATGTACAATTCAGAGAATTTAATCTTAAAGACCCCCCACTTAACCCT--------

>EU545544_pcv2b

ATGACGTATCCAAGGAGGCGTTACCGGAGAAGAAGACACCGCCCCCGCAGCCATCTTGGCCAGATCCTCCGCCGCCG
CCCCTGGCTCATCCACCCCCGCCACCGTTACCGCTGGAGAAGGAAAAATGGCATCTTCAACACCCGCCTCTCCCGCACC
TTCGGATATACTATCAAGCGAACCACAGTCAAAACGCCCTCCTGGGCGGTGGACATGATGAGATTCAATATTAATGAC
TTTCTTCCCCCAGGAGGGGGCTCAAACCCCCGCTCTGTGCCCTTTGAATACTACAGAATAAGAAAGGTTAAGGTTGAA
TTCTGGCCCTGCTCCCCGATCACCCAGGGTGACAGGGGAGTGGGCTCCAGTGCTGTTATTCTAGATGATAACTTTGTA
ACAAAGGCCACAGCCCTCACCTATGACCCCTATGTAAACTACTCCTCCCGCCATACCATAACCCAGCCCTTCTCCTACCA
CTCCCGCTACTTTACCCCCAAACCTGTCCTAGATTCCACTATTGATTACTTCCAACCAAACAACAAAAGAAATCAGCTGT
GGCTGAGACTACAAACTGCTGGAAATGTAGACCACGTAGGCCTCGGCACTGCGTTCGAAAACAGTATATACGACCAG
GAATACAATATCCGTGTAACCATGTATGTACAATTCAGAGAATTTAATCTTAAAGACCCCCCACTTAACCCT--------

>EU545543_pcv2b

ATGACGTATCCAAGGAGGCGTTACCGGAGAAGAAGACACCGCCCCCGCAGCCATCTTGGCCAGATCCTCCGCCGCCG
CCCCTGGCTCATCCACCCCCGCCACCGTTACCGCTGGAGAAGGAAAAATGGCATCTTCAACACCCGCCTCTCCCGCACC
TTCGGATATACTATCAAGCGAACCACAGTCAAAACGCCCTCCTGGGCGGTGGACATGATGAGATTCAATATTAATGAC
TTTCTTCCCCCAGGAGGGGGCTCAAACCCCCGCTCTGTGCCCTTTGAATACTACAGAATAAGAAAGGTTAAGGTTGAA
TTCTGGCCCTGCTCCCCGATCACCCAGGGTGACAGGGGAGTGGGCTCCAGTGCTGTTATTCTAGATGATAACTTTGTA
ACAAAGGCCACAGCCCTCACCTATGACCCCTATGTAAACTACTCCTCCCGCCATACCATAACCCAGCCCTTCTCCTACCA
CTCCCGCTACTTTACCCCCAAACCTGTCCTAGATTCCACTATTGATTACTTCCAACCAAACAACAAAAGAAATCAGCTGT
GGCTGAGACTACAAACTGCTGGAAATGTAGACCACGTAGGCCTCGGCACTGCGTTCGAAAACAGTATATACGACCAG
GAATACAATATCCGTGTAACCATGTATGTACAATTCAGAGAATTTAATCTTAAAGACCCCCCACTTAACCCT--------

>DQ201641_pcv2b

ATGACGTATCCAAGGAGGCGTTACCGGAGAAGAAGACACCGCCCCCGCAGCCATCTTGGCCAGATCCTCCGCCGCCG
CCCCTGGCTCATCCACCCCCGCCACCGTTACCGCTGGAGAAGGAAAAATGGCATCTTCAACACCCGCCTCTCCCGCACC
TTCGGATATACTATCAAGCGAACCACAGTCAAAACGCCCTCCTGGGCGGTGGACATGATGAGATTCAATATTAATGAC
TTTCTTCCCCCAGGAGGGGGCTCAAACCCCCGCTCTGTGCCCTTTGAATACTACAGAATAAGAAAGGTTAAGGTTGAA
TTCTGGCCCTGCTCCCCGATCACCCAGGGTGACAGGGGAGTGGGCTCCAGTGCTGTTATTCTGGATGATAACTTTGTA
ACAAAGGCCACAGCCCTCACCTATGACCCCTATGTAAACTACTCCTCCCGCCATACCATAACCCAGCCCTTCTCCTACCA
CTCCCGCTACTTTACCCCCAAACCTGTCCTAGATTCCACTATTGATTACTTCCAACCAAACAACAAAAGAAATCAGCTGT
GGCTGAGACTACAAACTGCTGGAAATGTAGACCACGTAGGCCTCGGCACTGCGTTCGAAAACAGTATATACGACCAG
GAATACAATATCCGTGTAACCATGTATGTACAATTCAGAGAATTTAATCTTAAAGACCCCCCACTTAACCCT--------

>JN662675_pcv2b

ATGACGTATCCAAGGAGGCGTTACCGGAGAAGAAGACACCGCCCCCGCAGCCATCTTGGCCAGATCCTCCGCCGCCG
CCCCTGGCTCGTCCACCCCCGCCACCGTTACCGCTGGAGAAGGAAAAATGGCATCTTCAACACCCGCCTCTCCCGCACC
TTCGGATATACTGTCAAGGCTACCACAGTCAGAACGCCCTCCTGGGCGGTGGACATGATGAGATTCAATATTAATGAC
TTTCTTCCCCCAGGAGGGGGCTCAAACCCCCGCTCTGTGCCCTTTGAATACTACAGAATAAGAAAGGTTAAGGTTGAA
TTCTGGCCCTGCTCCCCGATCACCCAGGGTGACAGGGGAGTGGGCTCCAGTGCTGTTATTCTAGATGATAACTTTGTA
ACAAAGGCCACAGCCCTCACCTATGACCCCTATGTAAACTACTCCTCCCGCCATACCATACCCCAGCCCTTCTCCTACCA
CTCCCGCTACTTTACCCCCAAACCTGTCCTAGATTCCACTATTGATTACTTCCAACCAAACAACAAAAGAAATCAGCTGT
GGATGAGACTACAAACTGCTGGAAATGTAGACCACGTAGGCCTCGGCACTGCGTTCGAAAACAGTAAATACGACCAG
GAATACAATATCCGTGTAACCATGTATGTACAATTCAGAGAATTTAATCTTAAAGACCCCCCACTTAAACCT--------

>JN662673_pcv2b

ATGACGTATCCAAGGAGGCGTTACCGGAGAAGAAGACACCGCCCCCGCAGCCATCTTGGCCAGATCCTCCGCCGCCG
CCCCTGGCTCGTCCACCCCCGCCACCGTTACCGCTGGAGAAGGAAAAATGGCATCTTCAACACCCGCCTCTCCCGCACC
TTCGGATATACTGTCAAGGCTACCACAGTCAGAACGCCCTCCTGGGCGGTGGACATGATGAGATTCAATATTAATGAC
TTTCTTCCCCCAGGAGGGGGCTCAAACCCCCGCTCTGTGCCCTTTGAATACTACAGAATAAGAAAGGTTAAGGTTGAA
TTCTGGCCCTGCTCCCCGATCACCCAGGGTGACAGGGGAGTGGGCTCCAGTGCTGTTATTCTAGATGATAACTTTGTA
ACAAAGGCCACAGCCCTCACCTATGACCCCTATGTAAACTACTCCTCCCGCCATACCATACCCCAGCCCTTCTCCTACCA
CTCCCGCTACTTTACCCCCAAACCTGTCCTAGATTCCACTATTGATTACTTCCAACCAAACAACAAAAGAAATCAGCTGT
GGATGAGACTACAAACTGCTGGAAATGTAGACCACGTAGGCCTCGGCACTGCGTTCGAAAACAGTAAATACGACCAG
GAATACAATATCCGTGTAACCATGTATGTACAATTCAGAGAATTTAATCTTAAAGACCCCCCACTTAAACCT--------

>AY321982_pcv2b

ATGACGTATCCAAGGAGGCGTTACCGGAGAAGAAGACACCGCCCCCGCAGCCATCTTGGCCAGATCCTCCGCCGCCG
CCCCTGGCTCGTCCACCCCCGCCACCGTTACCGCTGGAGAAGGAAAAATGGCATCTTCAACACCCGCCTCTCCCGCACC
TTCGGATATACTGTCAAGGCTACCACAGTCAGAACGCCCTCCTGGGCGGTGGACATGATGAGATTCAATATTAATGAC
TTTCTTCCCCCAGGAGGGGGCTCAAACCCCCGCTCTGTGCCCTTTGAATACTACAGAATAAGAAAGGTTAAGGTTGAA
TTCTGGCCCTGCTCCCCGATCACCCAGGGTGACAGGGGAGTGGGCTCCAGTGCTGTTATTCTAGATGATAACTTTGTA
ACAAAGGCCACAGCCCTCACCTATGACCCCTATGTAAACTACTCCTCCCGCCATACCATACCCCAGCCCTTCTCCTACCA
CTCCCGCTACTTTACACCCAAACCTGTCCTAGATTCCACTATTGATTACTTCCAACCAAACAACAAAAGAAATCAGCTGT
GGATGAGACTACAAACTGCTGGAAATGTAGACCACGTAGGCCTCGGCACTGCGTTCGAAAACAGTAAATACGACCAG
GAATACAATATCCGTGTAACCATGTATGTACAATTCAGAGAATTTAATCTTAAAGACCCCCCACTTAACCCT--------

>AY321983_pcv2b

ATGACGTATCCAAGGAGGCGTTACCGGAGAAGAAGACACCGCCCCCGCAGCCATCTTGGCCAGATCCTCCGCCGCCG
CCCCTGGCTCGTCCACCCCCGCCACCGTTACCGCTGGAGAAGGAAAAATGGCATCTTCAACACCCGCCTCTCCCGCACC
TTCGGATATACTGTCAAGGCTACCACAGTCAGAACGCCCTCCTGGGCGGTGGACATGATGAGATTCAATATTAATGAC
TTTCTTCCCCCAGGAGGGGGCTCAAACCCCCGCTCTGTGCCCTTTGAATACTACAGAATAAGAAAGGTTAAGGTTGAA
TTCTGGCCCTGCTCCCCGATCACCCAGGGTGACAGGGGAGTGGGCTCCAGTGCTGTTATTCTAGATGATAACTTTGTA
ACAAAGGCCCCAGCCCTCACCTATGACCCCTATGTAAACTACTCCTCCCGCCATACCATAACCCAGCCCTTCTCCTACCA
CTCCCGCTACTTTACCCCCAAACCTGTCCTAGATTCCACTATTGATTACTTCCAACCAAACAACAAAAGAAATCAGCTGT
GGATGAGACTACAAACTGCTGGAAATGTAGACCACGTAGGCCTCGGCACTGCGTTCGAAAACAGTATATACGACCAG
GAATACAATATCCGTGTAACCATGTATGTACAATTCAGAGAATTTAATCTTAAAGACCCCCCACTTAAACCT--------

>AY322003_pcv2b

ATGACGTATCCAAGGAGGCGTTACCGGAGAAGAAGACACCGCCCCCGCAGCCATCTTGGCCAGATCCTCCGCCGCCG
CCCCTGGCTCGTCCACCCCCGCCACCGTTACCGCTGGAGAAGGAAAAATGGCATCTTCAACACCCGCCTCTCCCGCACC
TTCGGATATACTATCAAGGCTACCACAGTCAGAACGCCCTCCTGGGCGGTGGACATGATGAGATTCAATATTAATGAC
TTTCTTCCCCCAGGAGGGGGCTCAAACCCCCGCTCTGTGCCCTTTGAATACTACAGAATAAGAAAGGTTAAGGTTGAA
TTCTGGCCCTGCTCCCCGATCACCCAGGGTGACAGGGGAGTGGGCTCCAGTGCTGTTATTCTAGATGATAACTTTGTA
ACAAAGGCCACAGCCCTCACCTATGACCCCTATGTAAACTACTCCTCCCGCCATACCATAACCCAGCCCTTCTCCTACCA
CTCCCGCTACTTTACCCCCAAACCTGTACTAGATTCCACTATTGATTACTTCCAACCAAACAACAAAAGAAATCAGCTGT
GGATGAGACTACAAACTGCTGGAAATGTAGACCACGTAGGCCTCGGCACTGCGTTCGAAAACAGTATATACGACCAG
GAATACAATATCCGTGTAACCATGTATGTACAATTCAGAGAATTTAATCTTAAAGACCCCCCACTTAAACCT--------

>HQ591367_pcv2b

ATGACGTATCCAAGGAGGCGTTTCCGCAGACGAAGACACCGCCCCCGCAGCCATCTTGGCCAAATCCTCCGCCGCCGC
CCCTGGCTCGTCCACCCCCGCCACCGTTACCGCTGGAAAAGGAAAAATGGCATCTTCAACACCCGCCTCTCCCGCACCT
TCGGATATACTGTCAAGGCTACCACAGTCAACACGCCCTCCTGGGCGGTGGACATGATGAGATTCAATATTGACGACT
TTGTTCCCCCAGGAGGGGGGTCCAACCCCCGCTCTGTGCCCTTTGAATACTACAGAATAAGAAAGGTTAAGGTTGAAT
TCTGGCCCTGCTCCCCGATCACCCAGGGTGACAGGGGAGTGGGCTCCAGTGCTGTTATTCTAGATGATAACTTTGTAC
CAAAGGCCACAGCCCTCACCTATGACCCCTATGTAAACTACTCCTCCCGCCATACCATAACCCAGCCCTTCTCCTACCAC
TCCCGCTACTTTACCCCCAAACCTGTCCTAGATTCCACTATTGATTACTTCCAACCAAACAACAAAAGAAATCAGCTGTG
GATGAGACTACAAACTGCTGGAAATGTAGACCACGTAGGCCTCGGCACTGCGTTCGAAAACAGTATATACGACCAGG
AATACAATATCCGTGTAACCCTGTATGTACAATTCAGAGAATTTAATCTTAAAGACCCCCCACTTAACCCT--------

>DQ218420_pcv2b

ATGACGTATCCAAGGAGGCGTTACCGGAGAAGAAGACACCGCCCCCGCAGCCATCTTGGCCAGATCCTCCGCCGCCG
CCCCTGGCTCGTCCACCCCCGCCACCGTTACCGCTGGAGAAGGAAAAATGGCATCTTCAACACGCGCCTCTCCCGCAC
CATCGGTTATACTGTCAAGGCTACCACAGTCAGAACGCCCTCCTGGGCGGTGGACATGATGAGATTCAATATTAATGA
CTTTCTTCCCCCAGGAGGGGGCTCAAACCCCCGCTCTGTGCCCTTTGAATACTACAGAATAAGAAAGGTTAAGGTTGA
ATTCTGGCCCTGCTCCCCGATCACCCAGGGTGACAGGGGAGTGGGCTCCAGTGCTGTTATTCTAGATGATAACTTTGT
AACAAAGGCCACAGCCCTCACCTATGACCCCTATGTAAACTACTCCTCCCGCCATACCATAACCCAGCCCTTCTCCTACC
ACTCCCGCTACTTTACCCCCAAACCTGTCCTAGATTCCACTATTGATTACTTCCAACCAAACAACAAAAGAAATCAGCTG
TGGCTGAGACTACAAACTGCTGGAAATGTAGACCACATAGGCCTCGGCACTGCGTTCGAAAACAGTATATACGACCA
GGAATACAATATCCGTGTAACCATGTATGTACAATTCAGAGAATTTAGTCTTAAAGACCCCCCACTTAACCCT--------

>DQ218421_pcv2b

ATGACGTATCCAAGGAGGCGTTACCGGAGAAGAAGACACCGCCCCCGCAGCCATCTTGGCCAGATCCTCCGCCGCCG
CCCCTGGCTCGTCCACCCCCGCCACCGTTACCGCTGGAGAAGGAAAAATGGCATCTTCAACACGCGCCTCTCCCGCAC
CATCGGTTATACTGTCAAGGCTACCACAGTCAGAACGCCCTCCTGGGCGGTGGACATGATGAGATTCAATATTAATGA
CTTTCTTCCCCCAGGAGGGGGCTCAAACCCCCGCTCTGTGCCCTTTGAATACTACAGAATAAGAAAGGTTAAGGTTGA
ATTCTGGCCCTGCTCCCCGATCACCCAGGGTGACAGGGGAGTGGGCTCCAGTGCTGTTATTCTAGATGATAACTTTGT
AACAAAGGCCACAGCCCTCACCTATGACCCCTATGTAAACTACTCCTCCCGCCATACCATAACCCAGCCCTTCTCCTACC
ACTCCCGCTACTTTACCCCCAAACCTGTCCTAGATTCCACTATTGATTACTTCCAACCAAACAACAAAAGAAATCAGCTG
TGGCTGAGACTACAAACTGCTGGAAATGTAGACCACATAGGCCTCGGCACTGCGTTCGAAAACAGTATATACGACCA
GGAATACAATATCCGTGTAACCATGTATGTACAATTCAGAGAATTTAGTCTTAAAGACCCCCCACTTAACCCT--------

>DQ218419_pcv2b

ATGACGTATCCAAGGAGGCGTTACCGAAGACGAAGACACCGCCCCCGCAGCCATCTTGGCCAAATCCTCCGCCGCCG
CCCCTGGCTCGTCCACCCCCGCCACCATTACCGCTGGAGAAGGAAAAATGGCATCTTCAACACCCGCCTCTCCCGCACC
ATCGGTTATACTGTCAAGGCTACCACAGTCAGAACGCCCTCCTGGGCGGTGGACATGATGAGATTCAATATTAATGAT
TTTCTTCCCCCAGGAGGGGGCTCAAACCCCCGCTCTGTGCCCTTTGAATACTACAGAATAAGAAAGGTTAAGGTTGAA
TTCTGGCCCTGCTCCCCCATCACCCAGGGTGACAGGGGAGTGGGCTCCAGTGCTGTTATTCTAGATGATAACTTTGTA
ACAAAGGCCACAGCCCTCACCTATGACCCCTATGTAAACTACTCCTCCCGCCATACCATAACCCAGCCCTTCTCCTACCA
CTCCCGCTACTTTACCCCCAAACCTGTCCTAGATTCCACTATTGATTACTTCCAACCAAACAACAAAAGAAATCAGCTGT
GGCTGAGACTACAAACTGCTGGAAATGTAGACCACGTAGGCCTCGGCACTGCGTTCGAAAACAGTATATACGACCAG
GAATACAATATCCGTGTAACCATGTATGTACAATTCAGAGAATTTAATCTTAAAGACCCCCCACTTAACCCT--------

>HM003570_pcv2b

ATGACGTATCCAAGGAGGCGTTACCGCAGAAGAAGACACCGCCCCCGCAGCCATCTTGGCCAGATCCTCCGCCGCCG
CCCCTGGCTCGTCCACCCCCGCCACCGTTACCGCTGGAGAAGGAAAAATGGCATCTTCAACACCCGCCTCTCCCGCACC
TTCGGATATACTATCAAGAGAACCACAGTCAGAACGCCCTCCTGGGCGGTGGACATGATGAGATTCAATATTAATGAC
TTTCTTCCCCCAGGAGGGGGCTCAAACCCCCGCTCTGTGCCCTTTGAATACTACAGAATAAGAAAGGTTAAGGTTGAA
TTCTGGCCCTGCTCCCCGATCACCCAGGGTGACAGGGGAGTGGGCTCCAGTGCTGTTATTCTAGATGATAACTTTGTA
ACAAAGGCCACAGCCCTCACCTATGACCCCTATGTAAACTACTCCTCCCGCCATACCATAACCCAGCCCTTCTCCTACCA
CTCCCGCTACTTTACCCCCAAACCTGTCCTAGATTCCACTATTGATTACTTCCAACCAAACAACAAAAGAAATCAGCTGT
GGCTGAGACTACAAACTGCTGGAAATGTAGACCACGTAGGCCTCGGCACTGCGTTCGAAAACAGTATATACGACCAG
GAATACAATATCCGTGTAACCATGTATGTACAATTCAGAGAATTTAATCTTAAAGACCCCCCACTTAACCCT--------

>JN006454_pcv2b

ATGACGTATCCAAGGAGGCGTTACCGGAGAAGAAGACACCGCCCCCGCAGCCATCTTGGCCAGATCCTCCGCCGCCG
CCCCTGGCTCGTCCACCCCCGCCACCGTTACCGCTGGAGAAGGAAAAATGGCATCTTCAACACCCGCCTCTCCCGCACC
TTCGGATATACTATCAAGAGAACCACAGTCAAAACGCCCTCCTGGGCGGTGGACATGATGAGATTCAATATTAATGAC
TTTCTTCCCCCAGGAGGGGGCTCAAACCCCCGCTCTGTGCCCTTTGAATACTACAGAATAAGAAAGGTTAAGGTTGAA
TTCTGGCCCTGCTCCCCGATCACCCAGGGTGACAGGGGAGTGGGCTCCAGTGCTGTTATTCTAGATGATAACTTTGTA
ACAAAGGCCACAGCCCTCACCTATGACCCCTATGTAAACTACTCCTCCCGCCATACCATAACCCAGCCCTTCTCCTACCA
CTCCCGCTACTTTACCCCCAAACCTGTCCTAGATTCCACTATTGATTACTTCCAACCAAACAACAAAAGAAATCAGCTGT
GGCTGAGACTACAAACTACTGGAAATGTAGACCACGTAGGCCTCGGCACTGCGTTCGAAAACAGTATATACGACCAG
GAATACAATATCCGTGTAACCATGTATGTACAATTCAGAGAATTTAATCTTAAAGACCCCCCACTTAACCCT--------

>EF524529_pcv2b

ATGACGTATCCAAGGAGGCGTTACCGGAGAAGAAGACACCGCCCCCGCAGCCATCTTGGCCAGATCCTCCGCCGCCG
CCCCTGGCTCGTCCACCCCCGCCACCGTTACCGCTGGAGAAGGAAAAATGGCATCTTCAACACCCGCCTCTCCCGCACC
TTCGGATATACTATCAAGAGAACCACAGTCAAAACGCCCTCCTGGGCGGTGGACATGATGAGATTCAATATTAATGAC
TTTATTCCCCCAGGAGGGGGCTCAAACCCCCGCTCTGTGCCCTTTGAATACTACAGAATAAGAAAGGTTAAGGTTGAA
TTCTGGCCCTGCTCCCCGATCACCCAGGGTGACAGGGGAGTGGGCTCCAGTGCTGTGATTCTAGATGATAACTTTGTA
ACAAAGGCCACAGCCCTCACCTATGACCCCTATGTAAACTACTCCTCCCGCCATACCATAACCCAGCCCTTCTCCTACCA
CTCCCGCTACTTTACCCCCAAACCTGTCCTAGATTCCACTATTGATTACTTCCAACCAAACAACAAAAGAAATCAGCTGT
GGCTGAGACTACAAACTACTGGAAATGTAGACCACGTAGGCCTCGGCACTGCGTTCGAAAACAGTATATACGACCAG
GAATACAATATCCGTGTAACCATGTATGTACAATTCAGAGAATTTAATCTTAAAGACCCCCCACTTAACCCT--------

>JN006453_pcv2b

ATGACGTATCCAAGGAGGCGTTACCGGAGAAGAAGACACCGCCCCCGCAGCCATCTTGGCCAGATCCTCCGCCGCCG
CCCCTGGCTCGTCCACCCCCGCCACCGTTACCGCTGGAGAAGGAAAAATGGCATCTTCAACACCCGCCTCTCCCGCACC
TTCGGATATACTATCAAGAGAACCACAGTCAAAACGCCCTCCTGGGCGGTGGACATGATGAGATTCAATATTAATGAC
TTTCTTCCCCCAGGAGGGGGCTCAAACCCCCGCTCTGTGCCCTTTGAATACTACAGAATAAGAAAGGTTAAGGTTGAA
TTCTGGCCCTGCTCCCCGATCACCCAGGGTGACAGGGGAGTGGGCTCCAGTGCTGTTATTCTAGATGATAACTTTGTA
ACAAAGGCCACAGCCCTCACCTATGACCCCTATGTAAACTACTCCTCCCGCCATACCATAACCCAGCCCTTCTCCTACCA
CTCCCGCTACTTTACCCCCAAACCTGTCCTAGATTCCACTATTGATTACTTCCAACCAAACAACAAAAGAAATCAGCTGT
GGCTGAGACTACAAACTACTGGAAATGTAGACCACGTAGGCCTCGGCACTGCGTTCGAAAACAGTATATACGACCAG
GAATACAATATCCGTGTAACCATGTATGTACAATTCAGAGAATTTAATCTTAAAGACCCCCCACTTAACCCT--------

>HQ378163_pcv2b

ATGACGTATCCAAGGAGGCGTTACCGGAGAAGAAGACACCGCCCCCGCAGCCATCTTGGCCAGATCCTCCGCCGCCG
CCCCTGGCTCGTCCACCCCCGCCACCGTTACCGCTGGAGAAGGAAAAATGGCATCTTCAACACCCGCCTCTCCCGCACC
TTCGGATATACTATCAAGAGAACCACAGTCAGAACGCCCTCCTGGGCGGTGGACATGATGAGATTCAATATTAATGAC
TTTCTTCCCCCAGGAGGGGGCTCAAACCCCCGCTCTGTGCCCTTTGAATACTACAGAATAAGAAAGGTTAAGGTTGAA
TTCTGGCCCTGCTCCCCGATCACCCAGGGTGACAGGGGAGTGGGCTCCAGTGCTGTTATTCTAGATGATAACTTTGTA
ACAAAGGCCACAGCCCTCACCTATGACCCCTATGTAAACTACTCCTCCCGCCATACCATACCCCAGCCCTTCTCCTACCA
CTCCCGCTACTTTACCCCCAAACCTGTCCTAGATTCCACTATTGATTACTTCCAACCAAACAACAAAAGAAATCAGCTGT
GGCTGAGACTACAAACTACTGGAAATGTAGACCACGTAGGCCTCGGCACTGCGTTCGAAAACAGTATATACGACCAG
GAATACAATATCCGTGTAACCATGTATGTACAATTCAGAGAATTTAATCTTAAAGACCCCCCACTTAACCCT--------

>HM776448_pcv2b

ATGACGTATCCAAGGAGGCGTTACCGGAGAAGAAGACACCGCCCCCGCAGCCATCTTGGCCAGATCCTCCGCCGCCG
CCCCTGGCTCGTCCACCCCCGCCACCGTTACCGCTGGAGAAGGAAAAATGGCATCTTCAACACCCGCCTCTCCCGCACC
TTCGGATATACTATCAAGAAAACCACAGTCAGAACGCCCTCCTGGGCGGTGGACATGATGAGATTCAATATTAATGAC
TTTCTTCCCCCAGGAGGGGGCTCAAACCCCCGCTCTGTGCCCTTTGAATACTACAGAATAAGAAAGGTTAAGGTTGAA
TTCTGGCCCTGCTCCCCGATCACCCAGGGTGACAGGGGAGTGGGCTCCAGTGCTGTTATTCTAGATGATAACTTTGTA
ACAAAGGCCACAGCCCTCACCTATGACCCCTATGTAAACTACTCCTCCCGCCATACCATAACCCAGCCCTTCTCCTACCA
CTCCCGCTACTTTACCCCCAAACCTGTCCTAGATTCCACTATTGATTACTTCCAACCAAACAACAAAAGAAATCAGCTGT
GGCTGAGACTACAAACTACTGGAAATGTAGACCACGTAGGCCTCGGCACTGCGTTCGAAAACAGTATATACGACCAG
GAATACAATATCCGTGTAACCATGTATGTACAATTCAGAGAATTTAATCTTAAAGACCCCCCACTTAACCCT--------

>JF317566_pcv2b

ATGACGTATCCAAGGAGGCGTTACCGGAGAAGAAGACACCGCCCCCGCAGCCATCTTGGCCAGATCCTCCGCCGCCG
CCCCTGGCTCGTCCACCCCCGCCACCGTTACCGCTGGAGAAGGAAAAATGGCATCTTCAACACCCGCCTCTCCCGCACC
TTCGGATATACTATCAAGAAAACCACAGTCAGAACGCCCTCCTGGGCGGTGGACATGATGAGATTCAATATTAATGAC
TTTCTTCCCCCAGGAGGGGGCTCAAACCCCCGCTCTGTGCCCTTTGAATACTACAGAATAAGAAAGGTTAAGGTTGAA
TTCTGGCCCTGCTCCCCGATCACCCAGGGTGACAGGGGAGTGGGCTCCAGTGCTGTTATTCTAGATGATAACTTTGTA
ACAAAGGCCACAGCCCTCACCTATGACCCCTATGTAAACTACTCCTCCCGCCATACCATAACCCAGCCCTTCTCCTACCA
CTCCCGCTACTTTACCCCCAAACCTGTCCTAGATTCCACTATTGATTACTTCCAACCAAACAACAAAAGAAATCAGCTGT
GGCTGAGACTACAAACTACTGGAAATGTAGACCACGTAGGCCTCGGCACTGCGTTCGAAAACAGTATATACGACCAG
GAATACAATATCCGTGTAACCATGTATGTACAATTCAGAGAATTTAATCTTAAAGACCCCCCACTTAACCCT--------

>JX945575_pcv2b

ATGACGTATCCAAGGAGGCGTTACCGGAGAAGAAGACACCGCCCCCGCAGCCATCTTGGCCAGATCCTCCGCCGCCG
CCCCTGGCTCGTCCACCCCCGCCACCGTTACCGCTGGAGAAGGAAAAATGGCATCTTCAACACCCGCCTCTCCCGCACC
TTCGGATATACTATCAAGAAAACCACAGTCAGAACGCCCTCCTGGGCGGTGGACATGATGAGATTCAATATTAATGAC
TTTCTTCCCCCAGGAGGGGGCTCCAACCCCCGCTCTGTGCCCTTTGAATACTACAGAATAAGAAAGGTTAAGGTTGAA
TTCTGGCCCTGCTCCCCGATCACCCAGGGTGACAGGGGAGTGGGCTCCAGTGCTGTTATTCTAGATGATAACTTTGTA
ACAAAGGCCACAGCCCTCACCTATGACCCCTATGTAAACTACTCCTCCCGCCATACCATAACCCAGCCCTTCTCCTACCA
CTCCCGCTACTTTACCCCCAAACCTGTCCTAGATTCCACTATTGATTACTTCCAACCAAACAACAAAAGAAATCAGCTGT
GGCTGAGACTACAAACTACTGGAAATGTAGACCACGTAGGCCTCGGCACTGCGTTCGAAAACAGTATATACGACCAG
GAATACAATATCCGTGTAACCATGTATGTACAATTCAGAGAATTTAATCTTAAAGACCCCCCACTTAACCCT--------

>JX948784_pcv2b

ATGACGTATCCAAGGAGGCGTTACCGGAGAAGAAGACACCGCCCCCGCAGCCATCTTGGCCAGATCCTCCGCCGCCG
GCCCTGGCTCGTCCACCCCCGCCACCGTTACCGCTGGAGAAGGAAAAATGGCATCTTCAACACCCGCCTCTCCCGCAC
CTTCGGATATACTATCAAGAAAACCACAGTCAGAACGCCCTCCTGGGCGGTGGACATGATGAGATTCAATATTAATGA
CTTTCTTCCCCCAGGAGGGGGCTCAAACCCCCGCTCTGTGCCCTTTGAATACTACAGAATAAGAAAGGTTAAGGTTGA
ATTCTGGCCCTGCTCCCCGATCACCCAGGGTGACAGGGGAGTGGGCTCCAGTGCTGTTATTCTAGATGATAACTTTGT
AACAAAGGCCACAGCCCTCACCTATGACCCCTATGTAAACTACTCCTCCCGCCATACCATAACCCAGCCCTTCTCCTACC
ACTCCCGCTACTTTACCCCCAAACCTGTCCTAGATTCCACTATTGATTACTTCCAACCAAACAACAAAAGAAATCAGCTA
TGGCTGAGACTACAAACTACTGGAAATGTAGACCACGTAGGCCTCGGCACTGCGTTCGAAAACAGTATATACGACCA
GGAATACAATATCCGTGTAACCATGTATGTACAATTCAGAGAATTTAATCTTAAAGACCCCCCACTTAACCCT--------

>EU503038_pcv2b

ATGACGTATCCAAGGAGGCGTTACCGGAGAAGAAGACACCGCCCCCGCAGCCATCTTGGCCAGATCCTCCGCCGCCG
CCCCTGGCTCGTCCACCCCCGCCACCGTTACCGCTGGAGAAGGAAAAATGGCATCTTCAACACCCGCCTCTCCCGCACC
TTCGGATATACTATCAAGAGAACCACAGTCAAAACGCCATCCTGGGCGGTGGACATGATGAGATTTAATATTAATGAC
TTTCTTCCCCCAGGAGGGGGGACAAACCCCCGCTCTGTGCCCTTTGAATACTACAGAATAAGAAAGGTTAAGGTTGAA
TTCTGGCCCTGCTCCCCAATCACCCAGGGTGACAGGGGAGTGGGCTCCAGTGCTGTTATTCTAGATGATAACTTTGTA
ACAAAGGCCACAGCCCTCACCTATGACCCCTATGTAAACTACTCCTCCCGCCATACCATAACCCAGCCCTTCTCCTACCA
CTCCCGCTACTTTACCCCCAAACCTGTCCTAGATTCCACTATTGATTACTTCCAACCAAACAACAAAAGAAATCAGCTGT
GGCTGAGACTACAAACTGCTGGAAATGTAGACCACGTAGGCCTCGGCACTGCGTTCGAAAACAGTAAATACGACCAG
GAATACAATATCCGTGTAACCATGTATGTGCAATTCAGAGAATTTAATCTTAAAGACCCCCCACTTAACCCT--------

>EU503035_pcv2b

ATGACGTATCCAAGGAGGCGTTACCGGAGAAGAAGACACCGCCCCCGCAGCCATCTTGGCCAGATCCTCCGCCGCCG
CCCCTGGCTCGTCCACCCCCGCCACCGTTACCGCTGGAGAAGGAAAAATGGCATCTTCAACACCCGCCTCTCCCGCACC
TTCGGATATACTATCAAGAGAACCACAGTCAAAACGCCATCCTGGGCGGTGGACATGATGAGATTCAATATTAATGAC
TTTCTTCCCCCAGGAGGGGGCTCAAACCCCCGCTCTGTGCCCTTTGAATACTACAGAATAAGAAAGGTTAAGGTTGAA
TTCTGGCCCTGCTCCCCGATCACCCAGGGTGACAGGGGAGTGGGCTCCAGTGCTGTTATTCTAGATGATAACTTTGTA
ACAAAGGCCACAGCCCTCACCTATGACCCCTATGTAAACTACTCCTCCCGCCATACCATAACCCAGCCCTTCTCCTACCA
CTCCCGCTACTTTACCCCCAAACCTGTCCTAGATTCCACTATTGATTACTTCCAACCAAACAACAAAAGAAATCGGCTGT
GGCTGAGACTACAAACTGCTGGAAATGTAGACCACGTAGGCCTCGGCACTGCGTTCGAAAACAGTATATACGACCAG
GAATACAATATCCGTGTAACCATGTATGTGCAATTCAGAGAATTTAATCTTAAAGACCCCCCACTTAACCCT--------

>AY536756_pcv2b

ATGACGTATCCAAGGAGGCGTTACCGGAGAAGAAGACACCGCCCCCGCAGCCATCTTGGCCAGATCCTCCGCCGCCG
CCCCTGGCTCGTCCACCCCCGCCACCGTTACCGCTGGAGAAGGAAAAATGGCATCTTCAACACCCGCCTCTCCCGCACC
TTCGGATATACTATCAAGAGAACCACAGTCAAAACGCCATCCTGGGCGGTGGACATGATGAGATTCAATATTAATGAC
TTTCTTCCCCCAGGAGGGGGCTCAAACCCCCGCTCTGTGCCCTTTGAATACTACAGAATAAGAAAGGTTAAGGTTGAA
TTCTGGCCCTGCTCCCCGATCACCCAGGGTGACAGGGGAGTGGGCTCCAGTGCTGTTATTCTAGATGATAACTTTGTA
ACAAAGGCCACAGCCCTCACCTATGACCCCTATGTAAACTACTCCTCCCGCCATACCATAACCCAGCCCTTCTCCTACCA
CTCCCGCTACTTTACCCCCAAACCTGTCCTAGATTCCACTATTGATTACTTCCAACCAAACAACAAAAGAAATCAGCTGT
GGCTGAGACTACAAACTGCTGGAAATGTAGACCACGTAGGCCTCGGCACTGCGTTCGAAAACAGTATATACGACCAG
GAATACAATATCCGTGTAACCATGTATGTGCAATTCAGAGAATTTAATCTTAAAGACCCCCCACTTAACCCT--------

>EU503032_pcv2b

ATGACGTATCCAAGGAGGCGTTACCGGAGAAGAAGACACCGCCCCCGCAGCCATCTTGGCCAGATCCTCCGCCGCCG
CCCCTGGCTCGTCCACCCCCGCCACCGTTACCGCTGGAGAAGGAAAAATGGCATCTTCAACACCCGCCTCTCCCGCACC
TTCGGATATACTATCAAGAGAACCACAGTCAAAACGCCATCCTGGGCGGTGGACATGATGAGATTCAATATTAATGAC
TTTCTTCCCCCAGGAGGGGGCTCAAACCCCCGCTCTGTGCCCTTTGAATACTACAGAATAAGAAAGGTTAAGGTTGAA
TTCTGGCCCTGCTCCCCGATCACCCAGGGTGACAGGGGAGTGGGCTCCAGTGCTGTTATTCTAGATGATAACTTTGTA
ACAAAGGCCACAGCCCTCACCTATGACCCCTATGTAAACTACTCCTCCCGCCATACCATAACCCAGCCCTTCTCCTACCA
CTCCCGCTACTTTACCCCCAAACCTGTCCTAGATTCCACTATTGATTACTTCCAACCAAACAACAAAAGAAATCAGCTGT
GGCTGAGACTACAAACTGCTGGAAATGTAGACCACGTAGGCCTCGGCACTGCGTTCGAAAACAGTATATACGACCAG
GAATACAATATCCGTGTAACCATGTATGTGCAATTCAGAGAATTTAATCTTAAAGACCCCCCACTTAACCCT--------

>EU503037_pcv2b

ATGACGTATCCAAGGAGGCGTTACCGGAGAAGAAGACACCGCCCCCGCAGCCATCTTGGCCAGATCCTCCGCCGCCG
CCCCTGGCTCATCCACCCCCGCCACCGTTACCGCTGGAGAAGGAAAAATGGCATCTTCAACACCCGCCTCTCCCGCACC
TTCGGATATACTGTCAAGAGAACCACAGTCAAAACGCCATCCTGGGCGGTGGACATGATGAGATTCAATATTAATGAC
TTTCTTCCCCCAGGAGGGGGCTCAAACCCCCGCTCTGTGCCCTTTGAATACTACAGAATAAGAAAGGTTAAGGTTGAA
TTCTGGCCCTGCTCCCCGATCACCCAGGGTGACAGGGGAGTGGGCTCCAGTGCTGTTATTCTAGATGATAACTTTGTA
ACAAAGGCCACAGCCCTCACCTATGACCCCTATGTAAACTACTCCTCCCGCCATACCATAACCCAGCCCTTCTCCTACCA
CTCCCGCTACTTTACCCCCAAACCTGTCCTAGATTCCACTATTGATTACTTCCAACCAAACAACAAAAGAAATCAGCTGT
GGCTGAGACTACAAACTGCTGGAAATGTAGACCACGTAGGCCTCGGCACTGCGTTCGAAAACAGTATATACGACCAG
GAATACAATATCCGTGTAACCATGTATGTGCAATTCAGAGAATTTAATCTTAAAGACCCCCCACTTAACCCT--------

>EU503033_pcv2b

ATGACGTATCCAAGGAGGCGTTACCGGAGAAGAAGACACCGCCCCCGCAGCCATCTTGGCCAGATCCTCCGCCGCCG
CCCCTGGCTCCTCCACCCCCGCCACCGTTACCGTTGGAGAAGGAAAAATGGCATCTTCAACACCCGCCTCTCCCGCACC
TTCGGATATACTGTCAAGAGAACCACAGTCGAAACGCCATCCTGGGCGGTGGACATGATGAGATTCAATATTAATGAC
TTTCTTCCCCCAGGAGGGGGCTCAAACCCCCGCACTGTGCCCTTTGAATACTACAGAATAAGAAAGGTTAAGGTTGAA
TTCTGGCCCTGCTCCCCGATCACCCAGGGTGACAGGGGAGTGGGCTCCAGTGCTGTTATTCTAGATGATAACTTTGTA
ACAAAGGCCACAGCCCTCACCTATGACCCCTATGTAAACTACTCCTCCCGCCATACCATAACCCAGCCCTTCTCCTACCA
CTCCCGCTACTTTACCCCCAAACCTGTCCTAGATTCCACTATTGATTACTTCCAACCAAACAACAAAAGAAATCAGCTTT
GGCTGAGACTACAAACTGCTGGAAATGTAGACCACGTAGGCCTCGGCACTGCGTTCGAAAACAGTATATACGACCAG
GAATACAATATCCGTGTAACCATGTATGTGCAATTCAGAGAATTTAATCTTAAAGACCCCCCACTTAACCCT--------

>EU503031_pcv2b

ATGACGTATCCAAGGAGGCGTTACCGGAGAAGAAGACACCGCCCCCGCAGCCATCTTGGCCAGATCCTCCGCCGCCG
CCCCTGGCTCGTCCACCCCCGCCACCGTTACCGCTGGAGAAGGAAAAATGGCATCTTCAACACCCGCCTCTCCCGCACC
TTCGGATATACTGTCAAGAGAACCACAGTCAGAACGCCATCCTGGGCGGTGGACATGATGAGATTCAATATTAATGAC
TTTCTTCCCCCAGGAGGGGGCTCAAACCCCCGCTCTGTGCCCTTTGAATACTACAGAATAAGAAAGGTTAAGGTTGAA
TTCTGGCCCTGCTCCCCGATCACCCAGGGTGACAGGGGAGTGGGCTCCAGTGCTGTTATTCTAGATGATAACTTTGTA
ACAAAGGCCACAGCCCTCACCTATGACCCCTATGTAAACTACTCCTCCCGCCATACCATAACCCAGCCCTTCTCCTACCA
CTCCCGCTACTTTACCCCCAAACCTGTCCTAGATTCCACTATTGATTACTTCCAACCAAACAACAAAAGAAATCAGCTGT
GGCTGAGACTACAAACTGCTGGAAATGTAGACCACGTAGGCCTCGGCACTGCGTTCGAAAACAGTATATACGACCAG
GAATACAATATCCGTGTAACCATGTATGTGCAATTCAGAGAATTTAATCTTAAAGACCCCCCACTTAACCCT--------

>EU503034_pcv2b

ATGACGTATCCAAGGAGGCGTTACCGAAGAAGAAGACACCGCCCCCGCAGCCATCTTGGCCAGATCCTCCGCCGCCG
CCCCTGGCTCATCCACCCCCGCCACCCTTACCGCTGGAGAAGGAAAAATGGCATCTTCAACACCCGCCTCTCCCGCACC
TTCGGATATACTATCAAGAGAACCACAGTCAGAACGCCATCCTGGGCGGTGGACATGATGAGATTCAATATTAATGAC
TTTCTTCCCCCAGGAGGGGGCTCAAACCCCCGCTCTGTGCCCTTTGAATACTACAGAATAAGAAAGGTTAAGGTTGAA
TTCTGGCCCTGCTCCCCGATCACCCAGGGTGACAGGGGAGTGGGCTCCAGTGCTGTTATTCTAGATGATAACTTTGTA
ACAAAGGCCACAGCCCTCACCTATGACCCCTATGTAAACTACTCCTCCCGCCATACCATAACCCAGCCCTTCTCCTACCA
CTCCCGCTACTTTACCCCCAAACCTGTCCTAGATTCCACTATTGATTACTTCCAACCAAACAACAAAAGAAATCAGCTGT
GGCTGAGACTACAAACTGCTGGAAATGTAGACCACGTAGGCCTCGGCACTGCGTTCGAAAACAGTATATACGACCAG
GAATACAATATCCGTGTAACCATGTATGTGCAATTCAGAGAATTTAATCTTAAAGACCCCCCACTTAACCCT--------

>EU503039_pcv2b

ATGACGTATCCAAGGAGGCGTTACCGAAGAAGAAGACACCGCCCCCGCAGCCATCTTGGCCAGATCCTCCGCCGCCG
CCCCTGGCTCGTCCACCCCCGCCACCGTTACCGCTGGAGAAGGAAAAATGGCATCTTCAACACCCGCCTCTCCCGCACC
TTCGGATATACTGTCAAGAGAACCACAGTCAGAACGCCATCCTGGGCGGTGGACATGATGAGATTCAATATTAATGAC
TTTCTTCCCCCAGGAGGGGGCTCAAACCCCCGCTCTGTGCCCTTTGAATACTACAGAATAAGAAAGGTTAAGGTTGAA
TTCTGGCCCTGCTCCCCGATCACCCAGGGTGACAGGGGAGTGGGCTCCAGTGCTGTTATTCTAGATGATAACTTTGTA
ACAAAGGCCACAGCCCTCACCTATGACCCCTATGTAAACTACTCCTCCCGCCATACCATAACCCAGCCCTTCTCCTACCA
CTCCCGCTACTTTACCCCCAAACCTGTCCTAGATTCCACTATTGATTACTTCCAACCAAACAACAAAAGAAATCAGCTCT
GGCTGAGACTACAAACTGCTGGAAATGTAGACCACGTAGGCCTCGGCACTGCGTTCGAAAACAGTATATACGACCAG
GAATACAATATCCGTGTAACCATGTATGTGCAATTCAGAGAATTTAATCTTAAAGACCCCCCACTTAACCCT--------

>HM009334_pcv2b

ATGACGTATCCAAGGAGGCGTTACCGGAGAAGAAGACACCGCCCCCGCAGCCATCTTGGCCAGATCCTCCGCCGCCG
CCCCTGGCTCGTCCACCCCCGCCACCGTTACCGCTGGAGAAGGAAAAATGGCATCTTCAACACCCGCCTCTCCCGCACC
TTCGGATATACTATCAAGAGAACCACAGTTAAAACTCCCTCCTGGGCGGTGGACATGATGAGATTCAATATTAATGAC
TTTCTTCCCCCAGGAGGGGGCTCAAACCCCCGCTCTGTGCCCTTTGAATACTACAGAATAAGAAAGGTTAAGGTTGAA
TTCTGGCCCTGCTCCCCGATCACCCAGGGTGACAGGGGAGTGGGCTCCAGTGCTGTTATTCTAGATGATAACTTTGTA
ACAAAGGCCACAGCCCTCACCTATGACCCCTATGTAAACTACTCCTCCCGCCATACCATAACCCAGCCCTTCTCCTACCA
CTCCCGCTACTTTACCCCCAAACCTGTCCTAGATTCCACTATTGATTACTTCCAACCAAACAACAAAAGAAATCAGCTGT
GGCTGAGACTACAAACTGCTGGAAATGTAGACCACGTAGGCCTCGGCACTGCGTTCGAAAACAGTATATACGACCAG
GAATACAATATCCGTGTAACCATGTATGTACAATTCAGAGAATTTAATCTTAAAGACCCCCCACTTAACCCT--------

>FJ644925_pcv2b

ATGACGTATCCAAGGAGGCGTTACCGGAGAAGAAGACACCGCCCCCGCAGCCATCTTGGCCAGATCCTCCGCCGCCG
CCCCTGGCTCGTCCACCCCCGCCACCGTTACCGCTGGAGAAGGAAAAATGGCATCTTCAACACCCGCCTCTCCCGCACC
TTCGGATATACTATCAAGAGAACCACAGTCAAAACGCCCTCCTGGGCGGTGGACATGATGAGATTCAATATTAATGAC
TTTCTTCCCCCAGGAGGGGGCTCAAACCCCCGCTCTGTGCCCTTTGAATACTACAGAATAAGAAAGGTTAAGGTTGAA
TTCTGGCCCTGCTCCCCGATCACCCAGGGTGACAGGGGAGTGGGCTCCAGTGCTGTTATTCTAGATGATAACTTTGTA
ACAAAGGCCACAGCCCTCACCTATGACCCCTATGTAAACTACTCCTCCCGCCATACCATAACCCAGCCCTTCTCCTACCA
CTCCCGCTACTTTACCCCCAAACCTGTCCTAGATTCCACTATTGATTACTTCCAACCAAACAACAAAAGAAATCAGCTGT
GGCTGAGACTACAAACTGCTGGAAATGTAGACCACGTAGGCCTCGGCACTGCGTTCGAAAACAGTATATACGACCAG
GAATACAATATCCGTGTAACCATGTATGTACAATTCAGAGAATTTAATCTTAAAGACCCCCCACTTAACCCT--------

>JN382171_pcv2b

ATGACGTATCCAAGGAGGCGTTACCGGAGAAGAAGACACCGCCCCCGCAGCCATCTTGGCCAGATCCTCCGCCGCCG
CCCCTGGGTCGCCCACCCCCGCCACCGTTACCGCTGGAGAAGGAAAAATGGCATCTTCAACACCCGCCTCTCCCGCAC
CTTCGGATATACTATCAAGAGAACCACAGTCAAAACGCCCTCCTGGGCGGTGGACATGATGAGATTCAATATTAATGA
CTTTCTTCCCCCAGGAGGGGGCTCAAACCCCCGCTCTGTGCCCTTTGAATACTACAGAATAAGAAAGGTTAAGGTTGA
ATTCTGGCCCTGCTCCCCGATCACCCAGGGTGACAGGGGAGTGGGCTCCAGTGCTGTTATTCTAGATGATAACTTTGT
AACAAAGGCCACAGCCCTCACCTATGACCCCTATGTAAACTACTCCTCCCGCCATACCATAACCCAGCCCTTCTCCTACC
ACTCCCGCTACTTTACCCCCAAACCTGTCCTAGATTCCACTATTGATTACTTCCAACCAAACAACAAAAGAAATCAGCTG
TGGCTGAGACTACAAACTGCTGGAAATGTAGACCACGTAGGCCTCGGCACTGCGTTCGAAAACAGTATATACGACCA
GGAATACAATATCCGTGTAACCATGTATGTACAATTCAGAGAATTTAATCTTAAAGACCCCCCACTTAACCCT--------

>AY691679_pcv2b

ATGACGTATCCAAGGAGGCGTTACCGGAGAAGAAGACACCGCCCCCGCAGCCATCTTGGCCAGATCCTCCGCCGCCG
CCCCTGGCTCGTCCACCCCCGCCACCGTTACCGCTGGAGAAGGAAAAATGGCATCTTCAACACCCGCCTCTCCCGCACC
TTCGGGTATACTATCAAGAGAACCACAGTCAAAACGCCCTCCTGGGCGGTGGACATGATGAGATTCAATATTAATGAC
TTTCTTCCCCCAGGAGGGGGCTCAAACCCCCGCTCTGTGCCCTTTGAATACTACAGAATAAGAAAGGTTAAGGTTGAA
TTCTGGCCCTGCTCCCCGATCACCCAGGGTGACAGGGGAGTGAGCTCCAGTGCTGTTATTCTAGATGATAACTTTGTA
ACAAAGGCCACAGCCCTCACCTATGACCCCTATGTAAACTACTCCTCCCGCCATACCATAACCCAGCCCTTCTCCTACCA
CTCCCGCTACTTTACCCCCAAACCTGTCCTAGATTCCACTATTGATTACTTCCAACCAAACAACAAAAGAAATCAGCTGT
GGCTGAGACTACAAACTGCTGGAAATGTAGACCACGTAGGCCTCGGCACTGCGTTCGAAAACAGTATATACGACCAG
GAATACGATATCCGTGTAACCATGTATGTACAATTCAGAGAATTTAATCTTAAAGACCCCCCACTTAACCCT--------

>HQ395036_pcv2b

ATGACGTATCCAAGGAGGCGTTACCGGAGAAGAAGACACCGCCCCCGCAGCCATCTTGGCCAGATCCTCCGCCGCCG
CCCCTGGCTCCTCCACCCCCGCCACCGTTACCGCTGGAGAAGGAAAAATGGCATCTTCAACACCCGCCTCTCCCGCACC
TTCGGATATACTATCAAGAGAACCACAGTCAAAACGCCCTCCTGGGCGGTGGACATGATGAGATTCAATATTAATGAC
TTTCTTCCCCCAGGAGGGGGCTCAAACCCCCGCTCTGTGCCCTTTGAATACTACAGAATAAGAAAGGTTAAGGTTGAA
TTCTGGCCCTGCTCCCCGATCACCCAGGGTGACAGGGGAGTGGGCTCCAGTGCTGTTATTCTAGATGATAACTTTGTA
ACAAAGGCCACAGCCCTCACCTATGACCCCTATGTAAACTACTCCTCCCGCCATACCATAACCCAGCCCTTCTCCTACCA
CTCCCGCTACTTTACCCCCAAACCTGTCCTAGATTCCACTATTGATTACTTCCAACCAAACAACAAAAGAAATCAGCTGT
GGCTGAGACTGCAAACTGCTGGAAATGTAGACCACGTAGGCCTCGGCACTGCGTTCGAAAACAGTATATACGACCAG
GAATACAATATCCGTGTAACCATGTATGTACAATTCAGAGAATTTAATCTTAAAGACCCCCCACTTAACCCT--------

>AY321987_pcv2b

ATGACGTATCCAAGGAGGCGTTACCGGAGAAGAAGACACCGCCCCCGCAGCCATCTTGGCCAGATCCTCCGCCGCCG
CCCCTGGCTCCTCCACCCCCGCCACCGTTACCGCTGGAGAAGGAAAAATGGCATCTTCAACACCCGCCTCTCCCGCACC
TTCGGATATACTGTCAAGCGAACCACAGTCAAAACGCCCTCCTGGGCGGTGGACATGATGAGATTCAATATTAATGAC
TTTCTTCCCCCAGGAGGGGGCTCAAACCCCCGCTCTGTGCCCTTTGAATACTACAGAATAAGAAAGGTTAAGGTTGAA
TTCTGGCCCTGCTCCCCGATCACCCAGGGTGACAGGGGAGTGGGCTCCAGTGCTGTTATTCTAGATGATAACTTTGTA
ACAAAGGCCACAGCCCTCACCTATGACCCCTATGTAAACTACTCCTCCCGCCATACCATAACCCAGCCCTTCTCCTACCA
CTCCCGCTACTTTACCCCCAAACCTGTCCTAGATTCCACTATTGATTACTTCCAACCAAACAACAAAAGAAATCAGCTGT
GGCTGAGACTACAAACTGCTGGAAATGTAGACCACGTAGGCCTCGGCACTGCGTTCGAAAACAGTATATACGACCAG
GAATACAATATCCGTGTAACCATGTATGTACAATTCAGAGAATTTAATCTTAAAGACCCCCCACTTAACCCT--------

>EF675236_pcv2b

ATGACGTATCCAAGGAGGTGTTACCGGAGAAGAAGACACCGCCCCCGCAGCCATCTTGGCCAGATCCTCCGCCGCCA
CCCCTGGCTCGTCCACCCCCGCCACCGTTACCGCTGGAGAAGGAAAAATGGCATCTTTAACTCCCGCCTCTCCCGCACC
TTCAGATATACTGTCAAGCGAACCACAGTTAAAACGCCCTCCTGGGCGGTGGACATGATGAGATTCAATATTAATGAC
TTTCTTCCCCCAGGAGGGGGCTCAAACCCCCGCTCTGTGCCCTTTGAATACTACAGAATAAGAAAGGTTAAGGTTGAA
TTCTGGCCCTGCTCCCCGATCACCCAGGGTGACAGGGGAGTGGGCTCCAGTGCTGTTATTCTAGATGATAACTTTGTA
ACAAAGGCCACAGCCCTCACCTATGACCCCTATGTAAACTACTCCTCCCGCCATACCATAACCCAGCCCTTCTCCTACCA
CTCCCGCTACTTTACCCCCAAACCTGTCCTAGATTCCACTATTGATTACTTCCAACCAAACAACAAAAGAAATCAGCTGT
GGCTGAGACTACAAACTGCTGGAAATGTAGACCACGTAGGCCTCGGCACTGCGTTCGAAACCAGTATATACGACCAG
GAATACAATATCCGTGTAACCATGTATGTACAATTCAGAGAATTTAATCTTAAAGACCCCCCACTT--------------

>EF675242_pcv2b

ATGACGTATCCAAGGAGGTGTTACCGGAGAAGAAGACACCGCCCCCGCAGCCATCTTGGCCAGATCCTCCGCCGCCA
CCCCTGGCTCGTCCACCCCCGCCACCGTTACCGCTGGAGAAGGAAAAATGGCATCTTTAACTCCCGCCTCTCCCGCACC
TTCAGATATACTGTCAAGCGAACCACAGTTAAAACGCCCTCCTGGGCGGTGGACATGATGAGATTCAATATTAATGAC
TTTCTTCCCCCAGGAGGGGGCTCAAACCCCCGCTCTGTGCCCTTTGAATACTACAGAATAAGAAAGGTTAAGGTTGAA
TTCTGGCCCTGCTCCCCGATCACCCAGGGTGACAGGGGAGTGGGCTCCAGTGCTGTTATTCTAGATGATAACTTTGTA
ACAAAGGCCACAGCCCTCACCTATGACCCCTATGTAAACTACTCCTCCCGCCATACCATAACCCAGCCCTTCTCCTACCA
CTCCCGCTACTTTACCCCCAAACCTGTCCTAGATTCCACTATTGATTACTTCCAACCAAACAACAAAAGAAATCAGCTGT
GGCTGAGACTACAAACTGCTGGAAATGTAGACCACGTAGGCCTCGGCACTGCGTTCGAAACCAGTATATACGACCAG
GAATACAATATCCGTGTAACCATGTATGTACAATTCAGAGAATTTAATCTTAAAGACCCCCCACTT--------------

>EF675237_pcv2b

ATGACGTATCCAAGGAGGCGTTACCGGAGAAGAAGACACCGCCCCCGCAGCCATCTTGGCCAGATCCTCCGCCGCCG
CCCCTGGCTCGTCCACCCCCGCCACCGTTACCGCTGGAGAAGGAAAAATGGCATCTTCAACACCCGCCTCTCCCGCACC
TTCGGATATACTGTCAAGCGAACCACAGTCAAAACGCCCTCCTGGGCGGTGGACATGATGAGATTCAATATTAATGAC
TTTCTTCCCCCAGGAGGGGGCTCAAACCCCCGCTCTGTGCCCTTTGAATACTACAGAATAAGAAAGGTTAAGGTTGAA
TTCTGGCCCTGCTCCCCGATCACCCAGGGTGACAGGGGAGTGGGCTCCAGTGCTGTTATTCTAGATGATAACTTTGTA
ACAAAGGCCACAGCCCTCACCTATGACCCCTATGTAAACTACTCCTCCCGCCATACCATAACCCAGCCCTTCTCCTACCA
CTCCCGCTACTTTACCCCCAAACCTGTCCTAGATTCCACTATTGATTACTTCCAACCAAACAACAAAAGAAATCAGCTGT
GGCTGAGACTACAAACTGCTGGAAATGTAGACCACGTAGGCCTCGGCACTGCGTTCGAAAACAGTATATACGACCAG
GAATACAATATCCGTGTAACCATGTATGTACAATTCAGAGAATTTAATCTTAAAGACCCCCCACTTAACCCT--------

>EF675239_pcv2b

ATGACGTATCCAAGGAGGCGTTACCGGAGAAGAAGACACCGCCCCCGCAGCCATCTTGGCCAGATCCTCCGCCGCCG
CCCCTGGCTCGTCCACCCCCGCCACCGTTACCGCTGGAGAAGGAAAAATGGCATCTTCAACACCCGCCTCTCCCGCACC
TTCGGATATACTGTCAAGCGAACCACAGTCAAAACGCCCTCCTGGGCGGTGGACATGATGAGATTCAATATTAATGAC
TTTCTTCCCCCAGGAGGGGGCTCAAACCCCCGCTCTGTGCCCTTTGAATACTACAGAATAAGAAAGGTTAAGGTTGAA
TTCTGGCCCTGCTCCCCGATCACCCAGGGTGACAGGGGAGTGGGCTCCAGTGCTGTTATTCTAGATGATAACTTTGTA
ACAAAGGCCACAGCCCTCACCTATGACCCCTATGTAAACTACTCCTCCCGCCATACCATAACCCAGCCCTTCTCCTACCA
CTCCCGCTACTTTACCCCCAAACCTGTCCTAGATTCCACTATTGATTACTTCCAACCAAACAACAAAAGAAATCAGCTGT
GGCTGAGACTACAAACTGCTGGAAATGTAGACCACGTAGGCCTCGGCACTGCGTTCGAAAACAGTATATACGACCAG
GAATACAATATCCGTGTAACCATGTATGTACAATTCAGAGAATTTAATCTTAAAGACCCCCCACTTAACCCT--------

>HM003569_pcv2b

ATGACGTATCCAAGGAGGCGTTACCGGAGAAGAAGACACCGCCCCCGCAGCCATCTTGGCCAGATCCTCCGCCGCCG
ACCCTGGCTCGTCCACCCCCGCCACCGTTACCGCTGGAGAAGGAAAAATGGCATCTTCAACACCCGCCTCTCCCGCACC
TTCGGATATACTGTCAAGCGAACCACAGTCAAAACGCCCTCCTGGGCGGTGGACATGATGAGATTCAATATTAATGAC
TTTCTTCCCCCAGGAGGGGGCTCAAACCCCCGCTCTGTGCCCTTTGAATACTACAGAATAAGAAAGGTTAAGGTTGAA
TTCTGGCCCTGCTCCCCGATCACCCAGGGTGACAGGGGAGTGGGCTCCAGTGCTGTTATTCTAGATGATAACTTTGTA
ACAAAGGCCACAGCCCTCACCTATGACCCCTATGTAAACTACTCCTCCCGCCATACCATAACCCAGCCCTTCTCCTACCA
CTCCCGCTACTTTACCCCCAAACCTGTCCTAGATTCCACTATTGATTACTTCCAACCAAACAACAAAAGAAATCAGCTGT
GGCTGAGACTACAAACTGCTGGAAATGTAGACCACGTAGGCCTCGGCACTGCGTTCGAAAACAGTATATACGACCAG
GAATACAATATCCGTGTAACCATGTATGTACAATTCAGAGAATTTAATCTTAAAGACCCCCCACTTAACCCT--------

>EF421969_pcv2b

ATGACGTATCCAAGGAGGCGTTACCGGAGAAGAAGACACCGCCCCCGCAGCCATCTTGGCCAGATCCTCCGCCGCCG
CCCCTGGCTCGTCCACCCCCGCCACCGTCACCGCTGGAGAAGGAAAAATGGCATCTTCAACACCCGCCTCTCCCGCAC
CTTCGGATATACTGTCAAGCGAACCACAGTCAAAACGCCCTCCTGGGCGGTGGACGTGATGAGATTCAATATTAATGA
CTTTCTTCCCCCAGGAGGGGGCTCAAACCCCCGCTCTGTGCCCTTTGAATACTACAGAATAAGAAAGGTTAAGGTTGA
ATTCTGGCCCTGCTCCCCGATCACCCAGGGTGACAGGGGAGTGGGCTCCAGTGCTGTTATTCTAGATGATAACTTTGT
AACAAAGGCCACAGCCCTCACCTATGACCCCTATGTAAACTACTCCTCCCGCCATACCATAACCCAGCCCTTCTCCTACC
ACTCCCGCTACTTTACCCCCAAACCTGTCCTAGATTCCACTATTGATTACTTCCAACCAAACAACAAAAGAAATCAGCTG
TGGCTGAGACTACAAACTGCTGGAAATGTAGACCACGTAGGCCTCGGCACTGCGTTCGAAAACAGTATATACGACCA
GGAATACAATATCCGTGTAACCATGTATGTACAATTCAGAGAATTTAATCTTAAAGACCCCCCACTTAACCCT--------

>EF524515_pcv2b

ATGACGTATCCAAGGAGGCGTTACCGGAGAAGAAGACACCGCCCCCGCAGCCATCTTGGCCAGATCCTCCGCCGCCG
CCCCTGGCTCGTCCACCCCCGCCACCGTTACCGCTGGAGAAGGAAAAATGGCATCTTCAACACCCGCCTCTCCCGCACC
TTCGGATATACTGTCAAGCGAACCACAGTCAAAACGCCCTCCTGGGCGGTGGACATGATGAGATTCAATATTAATGAC
TTTCTTCCCCCAGGAGGGGGCTCAAACCCCCGCTCTGTGCCCTTTGAATACTACAGAATAAGAAAGGTTAAGGTTGAA
TTCTGGCCCTGCTCCCCGATCACCCAGGGTGACAGGGGAGTGGGCTCCAGTGCTGTTATTCTAGATGATAACTTTGTA
ACAAAGGCCACAGCCCTCACCTATGACCCCTATGTAAACTACTCCTCCCGCCATACCATACCCCAGCCCTTCTCCTACCA
CTCCCGCTACTTTACCCCCAAACCTGTCCTAGATTCCACTATTGATTACTTCCAACCAAACAACAAAAGAAATCAGCTGT
GGCTGAGACTACAAACTACTGGAAATGTAGACCACGTAGGCCTCGGCACTGCGTTCGAAAACAGTATATACGACCAG
GAATACAATATCCGTGTAACCATGTATGTACAATTCAGAGAATTTAATCTTAAAGACCCCCCACTTAACCCT--------

>AF055394_pcv2b

ATGACGTATCCAAGGAGGCGTTACCGGAGAAGAAGACACCGCCCCCGCAGCCATCTTGGCCAGATCCTCCGCCGCCG
CCCCTGGCTCGTCCACCCCCGCCACCGTTACCGCTGGAGAAGGAAAAATGGCATCTTCAACACCCGCCTCTCCCGCACC
TTCGGATATACTGTCAAGCGAACCACAGTCAAAACGCCCTCCTGGGCGGTGGACATGATGAGATTCAATATTAATGAC
TTTCTTCCCCCAGGAGGGGGCTCAAACCCCCGCTCTGTGCCCTTTGAATACTACAGAATAAGAAAGGTTAAGGTTGAA
TTCTGGCCCTGCTCCCCGATCACCCAGGGTGACAGGGGAGTGGGCTCCAGTGCTGTTATTCTAGATGATAACTTTGTA
ACAAAGGCCACAGCCCTCACCTATGACCCCTATGTAAACTACTCCTCCCGCCATACCATAACCCAGCCCTTCTCCTACCA
CTCCCGCTACTTTACCCCCAAACCTGTCCTAGATTCCACTATTGATTACTTCCAACCAAACAACAAAAGAAATCAGCTGT
GGCTGAGACTACAAACTACTGGAAATGTAGACCACGTAGGCCTCGGCACTGCGTTCGAAAACAGTATATACGACCAG
GAATACAATATCCGTGTAACCATGTATGTACAATTCAGAGAATTTAATCTTAAAGACCCCCCACTTAACCCT--------

>AY484412_pcv2b

ATGACGTATCCAAGGAGGCGTTACCGGAGAAGAAGACACCGCCCCCGCAGCCATCTTGGCCAGATCCTCCGCCGCCG
CCCCTGGCTCGTCCACCCCCGCCACCGTTACCGCTGGAGAAGGAAAAATGGCATCTTCAACACCCGCCTCTCCCGCACC
TTCGGATATACTGTCAAGCGAACCACAGTCAGAACGCCCTCCTGGGCGGTGGACATGATGAGATTCAATATTAATGAC
TTTCTTCCCCCAGGAGGGGGCTCAAACCCCCGCTCTGTGCCCTTTGAATACTACAGAATAAGAAAGGTTAAGGTTGAA
TTCTGGCCCTGCTCCCCGATCACCCAGGGTGACAGGGGAGTGGGCTCCAGTGCTGTTATTCTAGATGATAACTTTGTA
ACAAAGGCCACAGCCCTCACCTATGACCCCTATGTAAACTACTCCTCCCGCCATACCATAACCCAGCCCTTCTCCTACCA
CTCCCGCTACTTTACCCCCAAACCTGTCCTAGATTCCACTATTGATTACTTCCAACCAAACAACAAAAGAAATCAGCTGT
GGCTGAGACTACAAACTACTGGAAATGTAGACCACGTAGGCCTCGGCACTGCGTTCGAAAACAGTATATACGACCAG
GAATACAATATCCGTGTAACCATGTATGTACAATTCAGAGAATTTAATCTTAAAGACCCCCCACTTAACCCT--------

>AY484408_pcv2b

ATGACGTATCCAAGGAGGCGTTACCGGAGAAGAAGACACCGCCCCCGCAGCCATCTTGGCCAGATCCTCCGCCGCCG
CCCCTGGCTCATCCACCCCCGCCACCGTTACCGCTGGAGAAGGAAAAATGGCATCTTCAACACCCGCCTCTCCCGCACC
TTCGGATATACTGTCAAGCGAACCACAGTCAGAACGCCCTCCTGGGCGGTGGACATGATGAGATTCAATATTAATGAC
TTTCTTCCCCCAGGAGGGGGCTCAAACCCCCGCTCTGTGCCCTTTGAATACTACAGAATAAGAAAGGTTAAGGTTGAA
TTCTGGCCCTGCTCCCCGATCACCCAGGGTGACAGGGGAGTGGGCTCCAGTGCTGTTATTCTAGATGATAACTTTGTA
ACAAAGGCCACAGCCCTCACCTATGACCCCTATGTAAACTACTCCTCCCGCCATACCATAACCCAGCCCTTCTCCTACCA
CTCCCGCTACTTTACCCCCAAACCTGTCCTAGATTCCACTATTGATTACTTCCAACCAAACAACAAAAGAAATCAGCTGT
GGCTGAGACTACAAACTGCTGGAAATGTAGACCACGTAGGCCTCGGCACTGCGTTCGAAAACAGTATATACGACCAG
GAATACAATATCCGTGTAACCATGTATGTACAATTCAGAGAATTTAATCTTAAAGACCCCCCACTTAACCCT--------

>AY321988_pcv2b

ATGACGTATCCAAGGAGGCGTTACCGGAGAAGAAGACACCGCCCCCGCAGCCATCTTGGCCAGATCCTCCGCCGCCG
CCCCTGGCTCGTCCACCCCCGCCACCGTTACCGCTGGAGAAGGAAAAATGGCATCTTCAACACCCGCCTCTCCCGCACC
TTCGGATATACTGTGAAGAGAACCACAGTCAGAACGCCCTCCTGGGCGGTGGACATGATGAGATTCAATATTAATGAC
TTTCTTCCCCCAGGAGGGGGCTCAAACCCCCGCTCTGTGCCCTTTGAATACTACAGAATAAGAAAGGTTAAGGTTGAA
TTCTGGCCCTGCTCCCCGATCACCCAGGGTGACAGGGGAGTGGGCTCCAGTGCTGTTATTCTAGATGATAACTTTGTA
ACAAAGGCCACAGCCCTCACCTATGACCCCTATGTAAACTACTCCTCCCGCCATACCATAACCCAGCCCTTCTCCTACCA
CTCCCGCTACTTTACCCCCAAACCTGTCCTAGATTCCACTATTGATTACTTCCAACCAAACAACAAAAGAAATCAGCTGT
GGCTGAGACTACAAACTGCTGGAAATGTAGACCACGTAGGCCTCGGCACTGCGTTCGAAAACAGTATATACGACCAG
GAATACAATATCCGTGTAACCATGTATGTACAATTCAGAGAATTTAATCTTAAAGACCCCCCACTTAACCCT--------

>AY321989_pcv2b

ATGACGTATCCAAGGAGGCGTTACCGGAGAAGAAGACACCGCCCCCGCAGCCATCTTGGCCAGATCCTCCGCCGCCG
CCCCTGGCTCGTCCACCCCCGCCACCGTTACCGCTGGAGAAGGAAAAATGGCATCTTCAACACCCGCCTCTCCCGCACC
TTCGGATATACTGTGAAGCGAACCACAGTCAGAACGCCCTCCTGGGCGGTGGACATGATGAGATTCAATATTAATGAC
TTTCTTCCCCCAGGAGGGGGCTCAAACCCCCGCTCTGTGCCCTTTGAATACTACAGAATAAGAAAGGTTAAGGTTGAA
TTCTGGCCCTGCTCCCCGATCACCCAGGGTGACAGGGGAGTGGGCTCCAGTGCTGTTATTCTAGATGATAACTTTGTA
ACAAAGGCCACAGCCCTCACCTATGACCCCTATGTAAACTACTCCTCCCGCCATACCATAACCCAGCCCTTCTCCTACCA
CTCCCGCTACTTTACCCCCAAACCTGTCCTAGATTCCACTATTGATTACTTCCAACCAAACAACAAAAGAAATCAGCTGT
GGCTGAGACTACAAACTGCTGGAAATGTAGACCACGTAGGCCTCGGCACTGCGTTCGAAAACAGTATATACGACCAG
GAATACAATATCCGTGTAACCATGTATGTACAATTCAGAGAATTTAATCTTAAAGACCCCCCACTTAACCCT--------

>HQ378159_pcv2b

ATGACGTATCCAAGGAGGCGTTACCGGAGAAGAAGACACCGCCCCCGCAGCCATCTTGGCCAGATCCTCCGCCGCCG
CCCCTGGCTCGTCCACCCCCGCCACCGTTACCGCTGGAGAAGGAAAAATGGCATCTTCAACACCCGCCTCTCCCGCACC
TTCGGATATACTGTCAAGCGAACCACAGTCAGAACGCCCTCCTGGGCGGTGGACATGATGAGATTCAATATTAATGAC
TTTCTTCCCCCAGGAGGGGGCTCAAACCCCCGCTCTGTGCCCTTTGAATACTACAGAATAAGAAAGGTTAAGGTTGAA
TTCTGGCCCTGCTCCCCGATCACCCAGGGTGACAGGGGAGTGGGCTCCAGTGCTGTTATTCTAGATGATAACTTTGTA
ACAAAGGCCACAGCCCTCACCTATGACCCCTATGTAAACTACTCCTCCCGCCATACCATAACCCAGCCCTTCTCCTACCA
CTCCCGCTACTTTACCCCCAAACCTGTCCTAGATTCCACTATTGATTACTTCCAACCAAACAACAAAAGAAATCAGCTGT
GGCTGAGACTACAAACTGCTGGAAATGTAGACCACGTAGGCCTCGGCACTGCGTTCGAAAACAGTATATACGACCAG
GAATACAATATCCGTGTAACCATGTATGTACAATTCAGAGAATTTAATCTTAAAGACCCCCCACTTAACCCT--------

>HM038016_pcv2b

ATGACGTATCCAAGGAGGCGTTACCGGAGAAGAAGACACCGCCCCCGCAGCCATCTTGGCCAGATCCTCCGCCGCCG
CCCCTGGCTCGTCCACCCCCGCCACCGTTACCGCTGGAGAAGGAAAAATGGCATCTTCAACACCCGCCTCTCCCGCACC
TTCGGATATACTGTCAAGCGAACCACAGTCAGAACGCCCTCCTGGGCGGTGGACATGATGAGATTCAATATTAATGAC
TTTCTTCCCCCAGGAGGGGGCTCAAACCCCCGCTCTGTGCCCTTTGAATACTACAGAATAAGAAAGGTTAAGGTTGAA
TTCTGGCCCTGCTCCCCGATCACCCAGGGTGACAGGGGAGTGGGCTCCAGTGCTGTTATTCTAGATGATAACTTTGTA
ACAAAGGCCACAGCCCTCACCTATGACCCCTATGTAAACTACTCCTCCCGCCATACCATAACCCAGCCCTTCTCCTACCA
CTCCCGCTACTTTACCCCCAAACCTGTCCTAGATTCCACTATTGATTACTTCCAACCAAACAACAAAAGAAATCAGCTGT
GGCTGAGACTACAAACTGCTGGAAATGTAGACCACGTAGGCCTCGGCACTGCGTTCGAAAACAGTATATACGACCAG
GAATACAATATCCGTGTAACCATGTATGTACAATTCAGAGAATTTAATCTTAAAGACCCCCCACTTAACCCT--------

>EU283329_pcv2b

ATGACGTATCCAAGGAGGCGTTACCGGAGAAGAAGACACCGCCCCCGCAGCCATCTTGGCCAGATCCTCCGCCGCCG
CCCCTGGCTCGTCCACCCCCGCCACCGTTACCGCTGGAGAAGGAAAAATGGCATCTTCAACACCCGCCTCTCCCGCACC
TTCGGATATACTGTCAAGCGAACCACAGTCAGAACGCCCTCCTGGGCGGTGGACATGATGAGATTCAATATTAATGAC
TTTCTTCCCCCAGGAGGGGGCTCAAACCCCCGCTCTGTGCCCTTTGAATACTACAGAATAAGAAAGGTTAAGGTTGAA
TTCTGGCCCTGCTCCCCGATCACCCAGGGTGACAGGGGAGTGGGCTCCAGTGCTGTTATTCTAGATGATAACTTTGTA
ACAAAGGCCACAGCCCTCACCTATGACCCCTATGTAAACTACTCCTCCCGCCATACCATAACCCAGCCCTTCTCCTACCA
CTCCCGCTACTTTACCCCCAAACCTGTCCTAGATTCCACTATTGATTACTTCCAACCAAACAACAAAAGAAATCAGCTGT
GGCTGAGACTACAAACTGCTGGAAATGTAGACCACGTAGGCCTCGGCACTGCGTTCGAAAACAGTATATACGACCAG
GAATACAATATCCGTGTAACCATGTATGTACAATTCAGAGAATTTAATCTTAAAGACCCCCCACTTAACCCT--------

>DQ220733_pcv2b

ATGACGTATCCAAGGAGGCGTTACCGCAGAAGAAGACACCGCCCCCGCAGCCATCTTGGCCAGATCCTCCGCCGCCG
CCCCTGGCTCGTCCACCCCCGCCACCGTTACCGTTGGAGAAGGAAAAATGGCATCTTCAACACCCGCCTATCCCGCACC
TTCGGATATACTGTCAAGCGAACCACAGTCAGAACGCCCTCCTGGGCGGTGGACATGATGAGATTCAATATTAATGAC
TTTCTTCCCCCAGGAGGGGGCTCAAACCCCCGCTCTGTGCCCTTTGAATACTACAGAATAAGAAAGGTTAAGGTTGAA
TTCTGGCCCTGCTCCCCCATCACCCAGGGTGACAGGGGAGTGGGCTCCAGTGCTGTTATTCTAGATGATAACTTTGTA
ACAAAGGCCACAGCCCTCACCTATGACCCCTATGTAAACTACTCCTCCCGCCATACCATACCCCAACCCTTCTCCTACCA
CTCCCGCTACTTTACCCCCAAACCTGTCCTAGATTCCACTATTGATTACTTCCAACCAAACAACAAAAGAAATCAGCTGT
GGCTGAGACTACAAACTGCTGGAAATGTAGACCACGTAGGCCTCGGCACTGCATTCGAAAACAGTATATACGACCAG
GAATACAATATCCGTGTAACCATGTATGTACAATTCAGAGAATTTAATCTTAAAGACCCCCCACTTAACCCT--------

>EU909688_pcv2b

ATGACGTATCCAAGGAGGCGTTACCGGAGAAGAAGACACCGCCCCCGCAGCCATCTTGGCCAGATCCTCCGCCGCCG
CCCCTGGCTCGTCCACCCCCGCCACCGTTACCGCTGGAGAAGGAAAAATGGCATCTTCAACACCCGCCTCTCCCGCACC
TTCGGATATACTGTCAAGCGAACCACAGTCAGAACGCCCTCCTGGGCGGTGGACATGATGAGATTCAATATTAATGAC
TTTCTTCCCCCAGGAGGGGGCTCAAACCCCCGCTCTGTGCCCTTTGAATACTACAGAATAAGAAAGGTTAAGGTTGAA
TTCTGGCCCTGCTCCCCGATCACCCAGGGTGACAGGGGAGTGGGCTCCAGTGCTGTTATTCTAGATGATAACTTTGTA
ACAAAGGCCACAGCCCTCACCTATGACCCCTATGTAAACTACTCCTCCCGCCATACCATACCCCAGCCCTTCTCCTACCA
CTCCCGCTACTTTACCCCCAAACCTGTCCTAGATTCCACTATTGATTACTTCCAACCAAACAACAAAAGAAATCAGCTGT
GGCTGAGACTACAAACTGCTGGAAATGTAGACCACGTAGGCCTCGGCATTGCGTTCGAAAACAGTATATACGACCAG
GAATACAATATCCGTGTAACCATGTATGTACAATTCAGAGAATTTAATCTTAAAGACCCCCCACTTAACCCT--------

>AF201897_pcv2b

ATGACGTATCCAAGGAGGCGTTACCGGAGAAGAAGACACCGCCCCCGCAGCCATCTTGGCCAGATCCTCCGCCGCCG
CCCCTGGCTCGTCCACCCCCGCCACCGTTACCGCTGGAGAAGGAAAAATGGCATCTTCAACACCCGCCTCTCCCGCACC
TTCGGATATACTGTCAAGCGAACCACAGTCAGAACGCCCTCCTGGGCGGTGGACATGATGAGATTCAATATTAATGAC
TTTCTTCCCCCAGGAGGGGGCTCAAACCCCCGCTCTGTGCCCTTTGAATACTACAGAATAAGAAAGGTTAAGGTTGAA
TTCTGGCCCTGCTCCCCGATCACCCAGGGTGACAGGGGAGTGGGCTCCAGTGCTGTTATTCTAGATGATAACTTTGTA
ACAAAGGCCACAGCCCTCACCTATGACCCCTATGTAAACTACTCCTCCCGCCATACCATAACCCAGCCCTTCTCCTACCA
CTCCCGCTACTTTACCCCCAAACCTGTCCTAGATTCCACTATTGATTACTTCCAACCAAACAACAAAAGAAATCAGCTGT
GGCTGAGACTACAAACTGCTGGAAATGTAGACCACGTAGGCCTCGGCACTGCGTTCGAAAACAGTATATACGACCAG
GAATACAATATCCGTGTAACCATGTATGTACAATTCAGAGAATTTAATCTTAAAGACCCCCCACTTAACCCT--------

>AY484413_pcv2b

ATGACGTATCCAAGGAGGCGTTACCGGAGAAGAAGACACCGCCCCCGCAGCCATCTTGGCCAGATCCTCCGCCGCCG
CCCCTGGCTCGTCCACCCCCGCCACCGTTACCGCTGGAGAAGGAAAAATGGCATCTTCAACACCCGCCTCTCCCGCACC
TTCGGATATACTGTCAAGCGAACCACAGTCAGAACGCCCTCCTGGGCGGTGGACATGATGAGATTCAATATTAATGAC
TTTCTTCCCCCAGGAGGGGGCTCAAACCCCCGCTCTGTGCCCTTTGAATACTACAGAATAAGAAAGGTTAAGGTTGAA
TTCTGGCCCTGCTCCCCGATCACCCAGGGTGACAGGGGAGTGGGCTCCAGTGCTGTTATTCTAGATGATAACTTTGTA
ACAAAGGCCACAGCCCTCACCTATGACCCCTATGTAAACTACTCCTCCCGCCATACCATAACCCAGCCCTTCTCCTACCA
CTCCCGCTACTTTACCCCCAAACCTGTCCTAGATTCCACTATTGATTACTTCCAACCAAACAACAAAAGAAATCAGCTGT
GGCTGAGACTACAAACTGCTGGAAATGTAGACCACGTAGGCCTCGGCACTGCGTTCGAAAACAGTATATACGACCAG
GAATACAATATCCGTGTAACCATGTATGTACAATTCAGAGAATTTAATCTTAAAGACCCCCCACTTAACCCT--------

>DQ220737_pcv2b

ATGACGTATCCAAGGAGGCGTTACCGGAGAAGAAGACACCGCCCCCGCAGCCATCTTGGCCAGATCCTCCGCCGCCG
CCCCTGGCTCGTCCACCCCCGCCACCGTTACCGCTGGAGAAGGAAAAATGGCATCTTCAACACCCGCCTCTCCCGCACC
TTCGGATATACTGTCAAGCGAACCACAGTCAGAACGCCCTCCTGGGCGGTGGACATGATGAGATTCAATATTAATGAC
TTTCTTCCCCCAGGAGGGGGCTCAAACCCCCGCTCTGTGCCCTTTGAATACTACAGAATAAGAAAGGTTAAGGTTGAA
TTCTGGCCCTGCTCCCCGATCACCCAGGGTGACAGGGGAGTGGGCTCCAGTGCTGTTATTCTAGATGATAACTTTGTA
ACAAAGGCCACAGCCCTCACCTATGACCCCTATGTAAACTACTCCTCCCGCCATACCATAACCCAGCCCTTCTCCTACCA
CTCCCGCTACTTTACCCCCAAACCTGTCCTAGATTCCACTATTGATTACTTCCAACCAAACAACAAAAGAAATCAGCTGT
GGCTGAGACTACAAACTGCTGGAAATGTAGACCACGTAGGCCTCGGCACTGCGTTCGAAAACAGTATATACGACCAG
GAATACAATATCCGTGTAACCATGTATGTACAATTCAGAGAATTTAATCTTAAAGACCCCCCACTTAACCCT--------

>AY484411_pcv2b

ATGACGTATCCAAGGAGGCGTTACCGGAGAAGAAGACACCGCCCCCGCAGCCATCTTGGCCAGATCCTCCGCCGCCG
CCCCTGGCTCGTCCACCCCCGCCACCGTTACCGCTGGAGAAGGAAAAATGGCATCTTCAACACCCGCCTCTCCCGCACC
TTCGGATATACTGTCAAGCGAACCACAGTCAGAACGCCCTCCTGGGCGGTGGACATGATGAGATTCAATATTAATGAC
TTTCTTCCCCCAGGAGGGGGCTCAAACCCCCGCTCTGTGCCCTTTGAATACTACAGAATAAGAAAGGTTAAGGTTGAA
TTCTGGCCCTGCTCCCCGATCACCCAGGGTGACAGGGGAGTGGGCTCCAGTGCTGTTATTCTAGATGATAACTTTGTA
ACAAAGGCCACAGCCCTCACCTATGACCCCTATGTAAACTACTCCTCCCGCCATACCATAACCCAGCCCTTCTCCTACCA
CTCCCGCTACTTTACCCCCAAACCTGTCCTAGATTCCACTATTGATTACTTCCAACCAAACAACAAAAGAAATCAGCTGT
GGCTGAGACTACAAACTGCTGGAAATGTAGACCACGTAGGCCTCGGCACTGCGTTCGAAAACAGTATATACGACCAG
GAATACAATATCCGTGTAACCATGTATGTACAATTCAGAGAATTTAATCTTAAAGACCCCCCACTTAACCCT--------

>AY256460_pcv2b

ATGACGTATCCAAGGAGGCGTTACCGGAGAAGAAGAAACCGCCCCCGCAGCCATCTTGGCCAGATCCTCCGCCGCCG
CCCCTGGCTCGTCCACCCCCGCCACCGTTACCGCTGGAGAAGGAAAAATGGCATCTTCAACACCCGCCTCTCCCGCACC
TTCGGATATACTGTCAAGCGAACCACAGTCAGAACGCCCTCCTGGGCGGTGGACATGATGAGATTCAATATTAATGAC
TTTCTTCCCCCAGGAGGGGGCTCAAACCCCCGCTCTGTGCCCTTTGAATACTACAGAATAAGAAAGGTTAAGGTTGAA
TTCTGGCCCTGCTCCCCGATCACCCAGGGTGACAGGGGAGTGGGCTCCAGTGCTGTTATTCTAGATGATAACTTTGTA
ACAAAGGCCACAGCCCTCACCTATGACCCCTATGTAAACTACTCCTCCCGCCATACCATAACCCAGCCCTTCTCCTACCA
CTCCCGCTACTTTACCCCCAAACCTGTCCTAGATTCCACTATTGATTACTTCCAACCAAACAACAAAAGAAATCAGCTGT
GGCTGAGACTACAAACTGCTGGAAATGTAGACCACGTAGGCCTCGGCACTGCGTTCGAAAACAGTATATACGACCAG
GAATACAATATCCGTGTAACCATGTATGTACAATTCAGAGAATTTAATCTTAAAGACCCCCCACTTAACCCT--------

>AY916791_pcv2b

ATGACGTATCCAAGGAGGCGTTACCGGAGAAGAAGAAACCGCCCCCGCAGCCATCTTGGCCAGATCCTCCGCCGCCG
CCCCTGGCTCGTCCACCCCCGCCACCGTTACCGCTGGAGAAGGAAAAATGGCATCTTCAACACCCGCCTCTCCCGCACC
TTCGGATATACTGTCAAGCGAACCACAGTCAGAACGCCCTCCTGGGCGGTGGACATGATGAGATTCAATATTAATGAC
TTTCTTCCCCCAGGAGGGGGCTCAAACCCCCGCTCTGTGCCCTTTGAATACTACAGAATAAGAAAGGTTAAGGTTGAA
TTCTGGCCCTGCTCCCCGATCACCCAGGGTGACAGGGGAGTGGGCTCCAGTGCTGTTATTCTAGATGATAACTTTGTA
ACAAAGGCCACAGCCCTCACCTATGACCCCTATGTAAACTACTCCTCCCGCCATACCATAACCCAGCCCTTCTCCTACCA
CTCCCGCTACTTTACCCCCAAACCTGTCCTAGATTCCACTATTGATTACTTCCAACCAAACAACAAAAGAAATCAGCTGT
GGCTGAGACTACAAACTGCTGGAAATGTAGACCACGTAGGCCTCGGCACTGCGTTCGAAAACAGTATATACGACCAG
GAATACAATATCCGTGTAACCATGTATGTACAATTCAGAGAATTTAATCTTAAAGACCCCCCACTTAACCCA--------

>AY613906_pcv2b

ATGACGTATCCAAGGAGGCGTTACCGGAGAAGAAGACACCGCCCCCGCTGCCATCTTGGCCAGATCCTCCGCCGCCG
CCCCTGGCTCGTCCACCCCCGCCACCGTTACCGCTGGAGAAGGAAAAATGGCATCTTCAACACCCGCCTCTCCCGCACC
TTCGGATATACTGTCAAGCGAACCACAGTCAGAACGCCCTCCTGGGCGGTGGACATGATGAGATTCAATATTAATGAC
TTTCTTCCCCCAGGAGGGGGCTCAAACCCCCGCTCTGTGCCCTTTGAATACTACAGAATAAGAAAGGTTAAGGTTGAA
TTCTGGCCCTGCTCCCCGATCACCCAGGGTGACAGGGGAGTGGGCTCCAGTGCTGTTATTCTAGATGATAACTTTGTA
ACAAAGGCCACAGCCCTCACCTATGACCCCTATGTAAACTACTCCTCCCGCCATACCATAACCCAGCCCTTCTCCTACCA
CTCCCGCTACTTTACCCCAAAACCTGTCCTAGATTCCACTATTGATTACTTCCAACCAAACAACAAAAGAAATCAGCTGT
GGCTGAGACTACAAACTGCTGGAAATGTAGACCACGTAGGCCTCGGCACTGCGTTCGAAAACAGTATATACGACCAG
GAATACAATATCCGTGTAACCACGTATGTACAATTCAGAGAATTTAATCTTAAAGACCCCCCACTTAACCCCTTA-----

>DQ997815_pcv2b

ATGACGTATCCAAGGAGGCGTTACCGGAGAAGAAGACACCGCCCCCGCAGCCATCTTGGCCAGATCCTCCGCCGCCG
CCCCTGGCTCGTCCACCCCCGCCACCGTTACCGCTGGAGACGGAAAAATGGCATCTTCAACACCCGCCTCTCCCGCACC
TTCGGATATACTGTCAAGCGAACCACAGTCAGAACGCCCTCCTGGGCGGTGGACATGATGAGATTCAATATTAATGAC
TTTCTTCCCCCAGGAGGGGGCTCAAACCCCCGCTCTGTGCCCTTTGAATACTACAGAATAAGAAAGGTTAAGGTTGAA
TTCTGGCCCTGCTCCCCGATCACCCAGGGTGACAGGGGAGTGGGCTCCAGTGCTGTTATTCTAGATGATAACTTTGTA
ACAAAGGCCACAGCCCTCACCTATGACCCCTATGTAAACTACTCCTCCCGCCATACCATAACCCAGCCCTTCTCCTACCA
CTCCCGCTACTTTACCCCAAAACCTGTCCTAGATTCCACTATTGATTACTTCCAACCAAACAACAAAAGAAATCAGCTGT
GGCTGAGACTACAAACTGCTGGAAATGTAGACCACGTAGGCCTCGGCACTGCGTTCGAAAACAGTATATACGACCAG
GAATACAATATCCGTGTAACCATGTATGTACAATTCAGAGAATTTAATCTTAAAGACCCCCCACTTAACCCT--------

>HM776444_pcv2b

ATGACGTATCCAAGGAGGCGTTACCGGAGAAGAAGACACCGCCCCCGCAGCCATCTTGGCCAGATCCTCCGCCGCCG
CCCCTGGCTCGTCCACCCCCGCCACCGTTACCGCTGGAGAAGGAAAAATGGCATCTTCAACACCCGCCTCTCCCGCACC
TTCGGATATACTGTCAAGCGAACCACAGTCAGAACGCCCTCCTGGGCGGTGGACATGATGAGATTCAATATTAATGAC
TTTCTTCCCCCAGGAGGGGGCTCAAACCCCCGCTCTGTGCCCTTTGAATACTACAGAATAAGAAAGGTTAAGGTTGAA
TTCTGGCCCTGCTCCCCGATCACCCAGGGTGACAGGGGAGTGGGCTCCAGTGCTGTTATTCTAGATGATAACTTTGTA
ACAAAGGCCACAGCCCTCACCTATGACCCCTATGTAAACTACTCCTCCCGCCATACCATAACCCAGCCCTTCTCCTACCA
CTCCCGCTACTTTACCCCCAAACCTGTCCTAGATTCCACTATTGATTACTTCCAACCAAACAACAAAAGAAATCAGCTGT
GGCTGAGACTACAAACTGCTGGAAATGTAGACCACGTAGGCCTCGGCACTGCGTTCGAAAACAGTATATACGACCAG
GAATACAATATCCGTGTAACCATGTATGTACAATTCAGAGAATTTAATCTTAAAGACCCCCCACTTAACCCT--------

>EF493841_pcv2b

ATGACGTATCCAAGGAGGCGTTACCGGAGAGGAAGACGCCGCCCCCGCAGCCATCTTGGCCAGATCCTCCGCCGCCG
CCCCTGGCTCGTCCACCCCCGCCACCGTTACCGCTGGAGAAGGAAAAATGGCATCTTCAACACCCGCCTCTCCCGCACC
TTCGGATATACTGTCAAGCGAACCACAGTCAGAACGCCCTCCTGGGCGGTGGACATGATGAGATTCAATATTAATGAC
TTTCTTCCCCCAGGAGGGGGCTCAAACCCCCGCTCTGTGCCCTTTGAATACTACAGAATAAGAAAGGTTAAGGTTGAA
TTCTGGCCCTGCTCCCCGATCACCCAGGGTGACAGGGGAGTGGGCTCCAGTGCTGTTATTCTAGATGATAACTTTGTA
ACAAAGGCCACAGCCCTCACCTATGACCCCTATGTAAACTACTCCTCCCGCCATACCATAACCCAGCCCTTCTCCTACCA
CTCCCGCTACTTTACCCCCAAACCTGTCCTAGATTCCACTATTGATTACTTCCAACCAAACAACAAAAGAAATCAGCTGT
GGCTGAGACTACAAACTGCTGGAAATGTAGACCACGTAGGCCTCGGCACTGCGTTCGAAAACAGTATATACGACCAG
GAATACAATATCCGTGTAACCATGTATGTACAATTCAGAGAATTTAATCTTAAAGACCCCCCACTTAACCCT--------

>EF493838_pcv2b

ATGACGTATCCAAGGAGGCGTTACCGGAGAAGAAGACGCCGCCCCCGCAGCCATCTTGGCCAGATCCTCCGCCGCCG
CCCCTGGCTCGTCCACCACCGCCACCGTTACCGCTGGAGAAGGAAAAATGGCATCTTCAACACCCGCCTCTCCCGCACC
TTCGGATATACTGTCAAGCGAACCACAGTCAGAACGCCCTCCTGGGCGGTGGACATGATGAGATTCAATATTAATGAC
TTTCTTCCCCCAGGAGGGGGCTCAAACCCCCGCTCTGTGCCCTTTGAATACTACAGAATAAGAAAGGTTAAGGTTGAA
TTCTGGCCCTGCTCCCCGATCACCCAGGGTGACAGGGGAGTGGGCCCCAGTGCTGTTATTCTAGATGATAACTTTGTA
ACAAAGGCCACAGCCCTCACCTATGACCCCTATGTAAACTACTCCTCCCGCCATACCATAACCCAGCCCTTCTCCTACCA
CTCCCGCTACTTTACCCCCAAACCTGTCCTAGATTCCACTATTGATTACTTCCAACCAAACAACAAAAGAAATCAGCTGT
GGCTGAGACTACAAACTGCTGGAAATGTAGACCACGTAGGCCTCGGCACTGCGTTCGAAAACAGTATATACGACCAG
GAATACAATATCCGTGTAACCATGTATGTACAATTCAGAGGATTTAATCTTAAAGACCCCCCACTTAACCCT--------

>EU346945_pcv2b

ATGACGTATCCAAGGAGGCGTTACCGGAGAAGAAGACGCCGCCCCCGCAGCCATCTTGGCCAGATCCTCCGCCGCCG
CCCCTGGCTCGTCCACCCCCGCCACCGTTACCGCTGGAGAAGGAAAAATGGCATCTTCAACACCCGCCTCTCCCGCACC
TTCGGATATACTGTCAAGCGAACCACAGTCAGAACGCCCTCCTGGGCGGTGGACATGATGAGATTCAATATTAATGAC
CTTCTTCCCCCAGGAGGGGGCTCAAACCCCCGCTCTGTGCCCTTTGAATACTACAGAATAAGAAAGGTTAAGGTTGAA
TTCTGGCCCTGCTCCCCGATCACCCAGGGTGACAGGGGAGTGGGCTCCAGTGCTGTTATTCTAGATGATAACTTTGTA
ACAAAGGCCACAGCCCTCACCTATGACCCCTATGTAAACTACTCCTCCCGCCATACCATAACCCAGCCCTTCTCCTACCA
CTCCCGCTACTTTACCCCCAAACCTGTCCTAGATTCCACTATTGATTACTTCCAACCAAACATCAAAAGAAATCAGCTGT
GGCTGAGACTACAAACTGCTGGAAATGTAGACCACGTAGGCCTCGGCACTGCGTTCGAAAACAGTATATACGACCAG
GAATACAATATCCGTGTAACCATGTATGTACAATTCAGAGAATTTAATCTTAAAGACCCCCCACTTAACCCT--------

>EF493842_pcv2b

ATGACGTATCCAAGGAGGCGTTACCGGAGAAGAAGACGCCGCCCCCGCAGCCATCTTGGCCAGATCCTCCGCCGCCG
CCCCTGGCTCGTCCACCCCCGCCACCGTTACCGCTGGAGAAGGAAAAATGGCATCTTCAACACCCGCCTCTCCCGCACC
TTCGGATATACTGTCAAGCGAACCACAGTCAGAACGCCCTCCTGGGCGGTGGACATGATGAGATTCAATATTAATGAC
TTTCTTCCCCCAGGAGGGGGCTCAAACCCCCGCTCTGTGCCCTTTGAATACTACAGAATAAGAAAGGTTAAGGTTGAA
TTCTGGCCCTGCTCCCCGATCACCCAGGGTGACAGGGGAGTGGGCTCCAGTGCTGTTATTCTAGATGATAACTTTGTA
ACAAAGGCCACAGCCCTCACCTATGACCCCTATGTAAACTACTCCTCCCGCCATACCATAACCCAGCCCTTCTCCCACCA
CTCCCGCTACTTTACCCCCAAACCTGTCCTAGATTCCACTATTGATTACTTCCAACCAAACAACAAAAGAAATCAGCTGT
GGCTGAGACTACAAACTGCTGGAAATGTAGACCACGTAGGCCTCGGCACTGCGTTCGAAAACAGTATATACGACCAG
GAATACAATATCCGTGTAACCATGTATGTACAATTCAGAGAATTTAATCTTAAAGACCCCCCACTTAACCCT--------

>EF493840_pcv2b

ATGACGTATCCAAGGAGGCGTTACCGGAGAAGAAGACGCCGCCCCCGCAGCCATCTTGGCCAGATCCTCCGCCGCCG
CCCCTGGCTCGTCCACCCCCGCCACCGTTACCGCTGGAGAAGGAAAAATGGCATCTTCAACACCCGCCTCTCCCGCACC
TTCGGATATACTGTCAAGCGAACCACAGTCAGAACGCCCTCCTGGGCGGTGGACATGATGAGATTCAATATTAATGAC
TTTCTTCCCCCAGGAGGGGGCTCAAACCCCCGCTCTGTGCCCTTTGAATACTACAGAATAAGAAAGGTTAAGGTTGAA
TTCTGGCCCTGCTCCCCGATCACCCAGGGTGACAGGGGAGTGGGCTCCAGTGCTGTTATTCTAGATGATAACTTTGTA
ACAAAGGCCACAGCCCTCACCTATGACCCCTATGTAAACTACTCCTCCCGCCATACCATAACCCAGCCCTTCTCCTACCA
CTCCCGCTACTTTACCCCCAAACCTGTCCTAGATTCCACTATTGATTACTTCCAACCAAACAACAAAAGAAATCAGCTGT
GGCTGAGACTACAAACTGCTGGAAATGTAGACCACGTAGGCCTCGGCACTGCGTTCGAAAACAGTATATACGACCAG
GAATACAATATCCGTGTAACCATGTATGTACAATTCAGAGAATTTAATCTTAAAGACCCCCCACTTAACCCT--------

>AY288133_pcv2b

ATGACGTATCCAAGGAGGCGTTACCGGAGAAGAAGACACCGCCCCCGCAGCCATCTTGGCCAGATCCTCCGCCGCCG
CCCCTGGCTCGTCCACCCCCGCCGCCGTTACCGCTGGAGAAGGAAAAATGGCATCTTCAACACCCGCCTCTCCCGCAC
CTTAGGATATACTGTCAAGCGAACCACAGTCAGAACGCCCTCCTGGGCGGTGGACATGATGAGATTCAATATTAATGA
CTTTCTTCCCCCAGGAGGGGGCTCAAACCCCCGCTCTGTGCCCTTTGAATACTACAGAATAAGAAAGGTTAAGGTTGA
ATTCTGGCCCTGCTCCCCGATCACCCAGGGTGACAGAGGAGTGGGCTCCAGTGCTGTTATTCTAGATGATAACTTTGT
AACAAAGGCCACAGCCCTCACCTATGACCCCTATGTAAACTACTCCTCCCGCCATACCATAACCCAGCCCTTCTCCTACC
ACTCCCGCTACTTTACCCCCAAACCTGTCCTAGATTCCACTATTGATTACTTCCAACCAAACAACAAAAGAAATCAGCTG
TGGCTGAGACTACAAACTGCTGGAAATGTAGACCACGTAGGCCTCGGCACTGCGTTCGAAAACAGTATATACGACCA
GGAATACAACATCCGTGTAACCATGTATGTACAATTCAGAGAATTTAATCTTAAAGACCCCCCACTTAACCCT--------

>EF421968_pcv2b

ATGACGTATCCAAGGAGGCGTTACCGGAGAAGAAGACGCCGCCCCCGCAGCCATCTTGGCCAGATCCTCCGCCGCCG
CCCCTGGCTCGTCCACCCCCGCCGCCGTTACCGCTGGAGAAGGAAAAATGGCATCTTCAACACCCGCCTCTCCCGCAC
CTTCGGATATACTGTCAAGCGAACCACAGTCAGAACGCCCTCCTGGGCGGTGGACATGATGAGATTCAATATCAATGA
CTTTCTTCCCCCAGGAGGGGGCTCAAACCCCCGCTCTGTGCCCTTTGAATACTACAGAATAAGAAAGGTTAAGGTTGA
ATTCTGGCCCTGCTCCCCGATCACCCAGGGTGACAGGGGAGTGGGCTCCAGTGCTGTTATTCTAGATGATAACTTTGT
AACAAAGGCCACAGCCCTCACCTATGACCCCTATGTAAACTACTCCTCCCGCCATACCATAACCCAGCCCTTCTCCTACC
ACTCCCGCTACTTTACCCCCAAACCTGTCCTAGATTCCACTATTGATTACTTCCAACCAAACAACAAAAGAAATCAGCTG
TGGCTGAGACTACAAACTGCTGGAAATGTAGACCACGTAGGCCTCGGCACTGCGTTCGAAAACAGTATATACGACCA
GGAATACAATATCCGTGTAACCATGTATGTACAATTCAGAGAATTTAATCTTAAAGACCCCCCACTTAACCCT--------

>EU366325_pcv2b

ATGACGTATCCAAGGAGGCGTTACCGGAGAAGAAGACACCGCCCCCGCAGCCATCTTGGCCAGATCCTCCGCCGCCG
CCCCTGGCTCGTCCACCCCCGCCACCGTTACCGCTGGAGAAGGAAAAATGGCATCTTCAACACCCGCCTCTCCCACACC
TTCGGATATACTGTCAAGCGAACCACAGTCAGAACGCCCTCCTGGGCGGTGGACATGATGAGATTCAATATTAATGAC
TTTCTTCCCCCAGGAGGGGGCTCAAACCCCCGCTCTGTGCCCTTTGAATACTACAGAATAAGAAAGGTTAAGGTTGAA
TTCTGGCCCTGCTCCCCGATCACCCAGGGTGACAGGGGAGTGGGCTCCAGTGCTGTTATTCTAGATGATAACTTTGTA
ACAAAGGCCACAGCCCTCACCTATGACCCCTATGTAAACTACTCCTCCCGCCATACCATAACCCAGCCCTTCTCCTACCA
CTCCCGCTACTTTACCCCCAAACCTGTCCTAGATTCCACTATTGATTACTTCCAACCAAACAACAAAAGAAATCAGCTGT
GGCTGAGACTACAAACTGCTGGAAATGTAGACCACGTAGGCCTCGGCACTGCGTTCGAAAACAGTATATACGACCAG
GAATACAATATCCGTGTAACCATGTATGTACAATTCAGAGAATTTAATCTTAAAGACCCCCCACTTAACCCT--------

>EF421970_pcv2b

ATGACGTATCCAAGGAGGCGTTACCGGAGAAGAAGACACCGCCCCCGCAGCCATCTTGGCCAGATCCTCCGCCGCCG
CCCCTGGCTCGTCCACCCCCGCCACCGTTACCGCTGGAGAAGGAAAAATGGCATCTTCAACACCCGCCTCTCCCGCACC
TTCGGATATACTGTCAAGCGAACCACAGTCAGAACGCCCTCCTGGGCGGTGGACATGATGAGATTCAATATTAATGAC
TTTCTTCCCCCAGGAGGGGGCTCAAACCCCCGCCCTGTGCCCTTTGAATACTACAGAATAAGAAAGGTTAAGGTTGAA
CTCTGGCCCTGCTCCCCGATCACCCAGGGTGACAGGGGAGTGGGCTCCAGTGCTGTTATTCTAGATGATAACTTTGTA
ACAAAGGCCACAGCCCTCACCTATGACCCCTATGTAAACTACTCCTCCCGCCATACCATAACCCAGCCCTTCTCCTACCA
CTCCCGCTACTTTACCCCCAAACCTGTCCTAGATTCCACTATTGACTACTTCCAACCAAACAACAAAAGAAATCCGCTGT
GGCTGAGACTACAAACTGCTGGAAATGTAGACCACGTAGGCCTCGGCACTGCGTTCGAAAACAGTATATACGACCAG
GAATACAATATCCGTGTAACCATGTATGTACAATTCAGAGAATTTAATCTTAAAGACCCCCCACTTAACCCT--------

>EF421971_pcv2b

ATGACGTATCCAAGGAGGCGTTACCGGAGAAGAAGACACCGCCCCCGCAGCCATCTTGGCCAGATCCTCCGCCGCCG
CCCCTGGCTCGTCCACCCCCGCCACCGTTACCGCTGGAGAAGGAAAAATGGCATCTTCAACACCCGCCTCTCCCGCACC
TTCGGATATACTGTCAAGCGAACCACAGTCAGAACGCCCTCCTGGGCGGTGGACATGATGAGATTCAATATTAATGAC
TTTCTTCCCCCAGGAGGGGGCTCAAACCCCCGCTCTGTGCCCTTTGAATACTACAGAATAAGAAAGGTTAAGGTTGAA
TTCTGGCCCTGCTCCCCGATCACCCAGGGTGACAGGGGAGTGGGCTCCAGTGCTGTTATTCTAGATGATAACTTTGTA
ACAAAGGCCACAGCCCTCACCTATGACCCCTATGTAAACTACTCCTCCCGCCATACCATAACCCAGCCCTTCTCCTACCA
CTCCCGCTACTTTACCCCCAAACCTGTCCTAGATTCCACTATTGACTACTTCCAACCAAACAACAAAAGAAATCAGCTGT
GGCTGAGACTACAAACTGCTGGAAATGTAGACCACGTAGGCCTCGGCACTGCGTTCGAAAACAGTATATACGACCAG
GAATACAATATCCGTGTAACCATGTATGTACAATTCAGAGAATTTAATCTTAAAGACCCCCCACTTAACCCT--------

>DQ220738_pcv2b

ATGACGTATCCAAGGAGGCGTTACCGGAGAAGAAGACACCGCCCCCGCAGCCATCTTGGCCAGATCCTCCGCCGCCG
CCCCTGGCTCGTCCACCCCCGCCACCGTTACCGCTGGAGAAGGAAAAATGGCATCTTCAACACCCGCCTCTCCCGCACC
TTCGGATATACTGTCAAGCGAACCACAGTCAGAACGCCCTCCTGGGCAGTGGACATGATGAGATTCAATATTAATGAC
TTTCTTCCCCCAGGAGGGGGCTCAAACCCCCGCTCTGTGCCCTTTGAATACTACAGAATAAGAAAGGTTAAGGTTGAA
TTCTGGCCCTGCTCCCCGATCACCCAGGGTGACAGGGGAGTGGGCTCCAGTGCTGTTATTCTAGATGATAACTTTGTA
ACAAAGGCCACAGCCCTCACCTATGACCCCTATGTAAACTACTCCTCCCGCCATACCATAACCCAGCCCTTCTCCTACCA
CTCCCGCTACTTTACCCCCAAACCTGTCCTAGATTCCACTATTGATTACTTCCAACCAAACAACAAAAGAAATCAGCTGT
GGCTGAGACTACAAACTGCTGGAAATGTAGACCACGTAGGCCTCGGCACTGCGTTCGAAAACAGTATATACGACCAG
GAATACAATATCCGTGTAACCATGTATGTACAATTCAGAGAATTTAATCTTAAAGACCCCCCACTTAACCCT--------

>EF452350_pcv2b

ATGACGTATCCAAGGAGGCGTTACCGGAGAAGAAGACACCGCCCCCGCAGCCATCTTGGCCAGATCCTCCGCCGCCG
CCCCTGGCTCGTCCACCCCCGCCACCGTTACCGCTGGAGAAGGAAAAATGGCATCTTCAACACCCGCCTCTCCCGCACC
TTCGGATATACTGTCAAGCGAACCACAGTCAGAACGCCCTCCTGGGCAGTGGACATGATGAGATTCAATATTAATGAC
TTTCTTCCCCCAGGAGGGGGCTCAAACCCCCGCTCTGTGCCCTTTGAATACTACAGAATAAGAAAGGTTAAGGTTGAA
TTCTGGCCCTGCTCCCCGATCACACAGGGTGACAGGGGAGTGGGCTCCAGTGCTGTTATTCTAGATGATAACTTTGTA
ACAAAGGCCACAGCCCTCACCTATGACCCCTATGTAAACTACTCCTCCCGCCATACCATAACCCAGCCCTTCTCCTACCA
CTCCCGCTACTTTACCCCCAAACCTGTCCTAGATTCCACTATTGATTACTTCCAACCAAACAACAAAAGAAATCAGCTGT
GGCTGAGACTACAAACTGCTGGAAATGTAGACCACGTAGGCCTCGGCACTGCGTTCGAAAACAGTATATACGACCAG
GAATACAATATCCGTGTAACCATGTATGTACAATTCAGAGAATTTAATCTTAAAGACCCCCCACTTAACCCT--------

>EF619971_pcv2b

ATGACGTATCCAAGGAGGCGTTACCGGAGAAGAAGACACCGCCCCCGCAGCCATCTTGGCCAGATCCTCCGCCGCCG
CCCCTGGCTCGTCCACCCCCGCCACCGTTACCGCTGGAGAAGGAAAAATGGCATCTTCAACACCCGCCTCTCCCGCACC
TTCGGATATACTGTCAAGCGAACCACAGTCAGAACGCCCTCCTGGGCAGTGGACATGATGAGATTCAATATTAATGAC
TTTCTTCCCCCAGGAGGGGGCTCAAACCCCCGCTCTGTGCCCTTTGAATACTACAGAATAAGAAAGGTTAAGGTTGAA
TTCTGGCCCTGCTCCCCGATCACACAGGGTGACAGGGGAGTGGGCTCCAGTGCTGTTATTCTAGATGATAACTTTGTA
ACAAAGGCCACAGCCCTCACCTATGACCCCTATGTAAACTACTCCTCCCGCCATACCATAACCCAGCCCTTCTCCTACCA
CTCCCGCTACTTTACCCCCAAACCTGTCCTAGATTCCACTATTGATTACTTCCAACCAAACAACAAAAGAAATCAGCTGT
GGCTGAGACTACAAACTGCTGGAAATGTAGACCACGTAGGCCTCGGCACTGCGTTCGAAAACAGTATATACGACCAG
GAATACAATATCCGTGTAACCATGTATGTACAATTCAGAGAATTTAATCTTAAAGACCCCCCACTTAACCCT--------

>HQ378161_pcv2b

ATGACGTATCCAAGGAGGCGTTACCGGAGAAGAAGACACCGCCCCCGCAGCCATCTTGGCCAGATCCTCCGCCGCCG
CCCCTGGCTCGTCCACCCCCGCCACCGTTACCGCTGGAGAAGGAAAAATGGCATCTTCAACACCCGCCTCTCCCGCACC
TTCGGATATACTGTCAAGCGAACCACAGTCAGAACGCCCTCCTGGGCGGTGGACATGATGAGATTCAATATTAATGAC
TTTCTTCCCCCAGGAGGGGGCTCAAACCCCCGCTCTGTGCCCTTTGAATACTACAGAATAAGAAAGGTTAAGGTTGAA
TTCTGGCCCTGCTCCCCGATCACCCAGGGTGACAGGGGAGTGGGCTCCAGTGCTGTTATTCTAGATGATAACTTTGTA
ACAAAGGCCACAGCCCTCACCTATGACCCCTATGTAAACTACTCCTCCCGCCATACCATAACCCAGCCCTTCTCCTACCA
CTCCCGCTACTTTACCCCCAAACCTGTCCTAGATTCCACTATTGATTACTTCCAACCAAACAACAAAAGAAATCAGCTGT
GGCTGAGACTACAAACTGCTGGAAATGTAGACCACGTAGGCCTCGGCACTGCGTTCGAAAACAGTATATACGACCAG
GAATACAATATCCGTGTAACCATGTATGTACAATTCAGAGAATTTAATCTTAAAGACCCCCCACTTAACCCT--------

>JN662684_pcv2b

ATGACGTATCCAAGGAGGCGTTACCGGAGAAGAAGACACCGCCCCCGCAGCCATCTTGGCCAGATCCTCCGCCGCCG
CCCCTGGCTCGTCCACCCCCGCCACCGTTACCGCTGGAGAAGGAAAAATGGCATCTTCAACACCCGCCTCTCCCGCACC
TTCGGATATACTGTCAAGCGAACCACAGTCAGAACGCCCTCCTGGGCGGTGGACATGATGAGATTCAATATTAATGAC
TTTCTTCCCCCAGGAGGGGGCTCAAACCCCCGCTCTGTGCCCTTTGAATACTACAGAATAAGAAAGGTTAAGGTTGAA
TTCTGGCCCTGCTCCCCGATCACCCAGGGTGACAGGGGAGTGGGCTCCAGTGCTGTTATTCTAGATGATAACTTTGTA
ACAAAGGCCACAGCCCTCACCTATGACCCCTATGTAAACTACTCCTCCCGCCATACCATAACCCAGCCCTTCTCCTACCA
CTCCCGCTACTTTACCCCCAAACCTGTCCTAGATTCCACTATTGATTACTTCCAACCAAACAACAAAAGAAATCAGCTGT
GGCTGAGACTACAAACTGCTGGAAATGTAGACCACGTAGGCCTCGGCACTGCGTTCGAAAACAGTATATACGACCAG
GAATACAATATCCGTGTAACCATGTATGTACAATTCAGAGAATTTAATCTTAAAGACCCCCCACTTAACCCT--------

>DQ915586_pcv2b

ATGACGTATCCAAGAAGGCGTTACCGAACAAGAAGACACCGCCCCCGCAGCCATCTTGGCCAGATCCTCCGCCGCCGC
CCCTGGCTCGTCCACCCCCGCCACCGTTACCGCTGGAGAAGGAAAAATGGCATCTTCAACACCCGCCTCTCCCGCACCT
TCGGATATACTGTCAAGCGAACCACAGTCAGAACGCCCTCCTGGGCGGTGGACATGATGAGATTCAATATTAATGACT
TTCTTCCCCCAGGAGGGGGCTCAAACCCCCGTTCTGTGCCCTTTGAATACTACAGAATAAGAAAGGTTAAGGTTGAAT
TCTGGCCTTGTTCGCGGATCACCCAGGGTGAGAGGGGAGTGGGCTCCACTGCTGTTATTTTAGATGATAACTTTGTAA
CAAAGGCCACACCCCTCTCCTATGACCCCTATGTAAACTACTCCTCCCGCCATACCATAACCCAGCCCTTCTCCTCCCCC
TCCCGGTGCTTTACCCCCAAACCTGTCCTAGATTCGACTATTGATTACTTCCAACCAAACAACAAAAGAAACCAGCTGG
GGTTGAGACTACAAACTGCTGGAAATGTAGACCACGTAGGCCTCGGCGCTGTGTTCGAAAACAGTATATACGACCAG
GAATACAATATCCGTGTAACCATGTATGTACAATTCAGAGAATTTAATTTTAAAGACCCCCCACTTAACCCT--------

>DQ915587_pcv2b

ATGACGTATCCAAGAAGGCGTTACCGAACAAGAAGACACCGCCCCCGCAGCCATCTTGGCCAGATCCTCCGCCGCCGC
CCCTGGCTCGTCCACCCCCGCCACCGTTACCGCTGGAGAAGGAAAAATGGCATCTTCAACACCCGCCTCTCCCGCACCT
TCGGATATACTGTCAAGCGAACCACAGTCAGAACGCCCTCCTGGGCGGTGGACATGATGAGATTCAATATTAATGACT
TTCTTCCCCCAGGAGGGGGCTCAAACCCCCGTTCTGTGCCCTTTGAATACTACAGAATAAGAAAGGTTAAGGTTGAAT
TCTGGCCTTGTTCGCGGATCACCCAGGGTGAGAGGGGAGTGGGCTCCACTGCTGTTATTTTAGATGATAACTTTGTAA
CAAAGGCCACACCCCTCTCCTATGACCCCTATGTAAACTACTCCTCCCGCCATACCATAACCCAGCCCTTCTCCTCCCCC
TCCCGGTGCTTTACCCCCAAACCTGTCCTAGATTCGACTATTGATTACTTCCAACCAAACAACAAAAGAAACCAGCTGG
GGTTGAGACTACAAACTGCTGGAAATGTAGACCACGTAGGCCTCGGCGCTGTGTTCGAAAACAGTATATACGACCAG
GAATACAATATCCGTGTAACCATGTATGTACAATTCAGAGAATTTAATTTTAAAGACCCCCCACTTAACCCT--------

>DQ915585_pcv2b

ATGACGTATCCAAGAAGGCGTTACCGCACAAGAAGACACCGCCCCCGCAGCCATCTTGGCCAGATCCTCCGCCGCCGC
CCCTGGCTCGTCCACCCCCGCCACCGTTACCGCTGGAGAAGGAAAAATGGCATCTTCAACACCCGCCTCTCCCGCACCT
TCGGATATACTGTCAAGCGAACCACAGTCAGAACGCCCTCCTGGGCGGTGGACATGATGAGATTCAATATTAATGACT
TTCTTCCCCCAGGAGGGGGTCAAACCCCCGTTCTGTGCCCTTTGAATACTACAGAATAAGAAAGGTTAAGGTTGAAT
TCTGGCCTTGTTCGCGGATCACCCAGGGTGAGAGGGGAGTGGGCTCCACTGCTGTTATTCTAGATGATAACTTTGTAA
CAAAGGCCACACCCCTCTCCTATGACCCCTATGTAAACTACTCCTCCCGCCATACCATAACCCAGCCCTTCTCCTCCCCC
TCCCGGTGCTTTACCCCCAAACCTGTCCGATCTTCGACTATTGATTACTTCCAACCAAACAACAAAAGAAATCAGCTGG
GGTTGAGACTACAAACTGCTGGAAATGTAGACCACGTAGGCCTCGGCGCTGTGTCAGAAAACAGTATATACGACCAG
GAATACAATATCCGTGTAACCATGTATGTACAATTCAGAGAATTTAATCTTAAAGACCCCCCACTTAACCCT--------

>AJ623306_pcv2b

ATGACGTATCCAAGGAGGCGTTACCGAAGAAGAAGACACCGCCCCCGCAGCCATCTTGGCCAGATCCTCCGCCGCCG
CCCCTGGCTCGTCCACCCCCGCCACCGTTACCGCTGGAGAAGGAAAAATGGCATCTTCAACACCCGCCTCTCCCGCACC
TTCGGATATACTGTCAAGCGAACCACAGTCAGAACGCCCTCCTGGGCGGTGGACATGATGAGATTCAATATTAATGAC
TTTCTTCCCCCAGGAGGGGGCTCAAACCCCCGCTCTGTGCCCTTTGAATACTACAGAATAAGAAAGGTTAAGGTTGAA
TTCTGGCCCTGCTCCCCGATCACCCAGGGTGACAGGGGAGTGGGCTCCAGTGCTGTTATTCTAGATGATAACTTTGTA
ACAAAGGCCACAGCCCTCACCTATGACCCCTATGTAAACTACTCCTCCCGCCATACCATAACCCAGCCCTTCTCCTACCA
CTCCCGGTACTTTACCCCCAAACCTGTCCTAGATTCCACTATTGATTACTTCCAACCAAACAACAAAAGAAACCAGCTGT
GGCTGAGACTACAAACTGCTGGAAATGTAGACCACGTAGGCCTCGGCACTGCGTTCGAAAACAGTATATACGACCAG
GAATACAATATCCGTGTAACCATGTATGTACAATTCAGAGAATTTAATTTTAAAGACCCCCCACTTAACCCT--------

>AF201311_pcv2b

ATGACGTATCCAAGGAGGCGTTACCGAAGAAGAAGACACCGCCCCCGCAGCCATCTTGGCCAGATCCTCCGCCGCCG
CCCCTGGCTCGTCCACCCCCGCCACCGTTACCGCTGGAGAAGGAAAAATGGCATCTTCAACACCCGCCTCTCCCGCACC
TTCGGATATACTGTCAAGCGAACCACAGTCAGAACGCCCTCCTGGGCGGTGGACATGATGAGATTCAATATTAATGAC
TTTCTTCCCCCAGGAGGGGGCTCAAACCCCCGCTCTGTGCCCTTTGAATACTACAGAATAAGAAAGGTTAAGGTTGAA
TTCTGGCCCTGCTCCCCGATCACCCAGGGTGACAGGGGAGTGGGCTCCAGTGCTGTTATTTTAGATGATAACTTTGTA
ACAAAGGCCACAGCCCTCACCTATGACCCCTATGTAAACTACTCCTCCCGCCATACCATAACCCAGCCCTTCTCCTACCA
CTCCCGGTACTTTACCCCCAAACCTGTCCTAGATTCCACTATTGATTACTTCCAACCAAACAACAAAAGAAACCAGCTGT
GGCTGAGACTACAAACTGCTGGAAATGTAGACCACGTAGGCCTCGGCACTGCGTTCGAAAACAGTATATACGACCAG
GAATACAATATCCGTGTAACCATGTATGTACAATTCAGAGAATTTAATTTTAAAGACCCCCCACTTAACCCT--------

>AY122275_pcv2b

ATGACGTATCCAAGGAGGCGTTACCGAAGAAGAAGACACCGCCCCCGCAGCCATCTTGGCCAGATCCTCCGCCGCCG
CCCCTGGCTCGTCCACCCCCGCCACCGTTACCGCTGGAGAAGGAAAAATGGCATCTTCAACACCCGCCTCTCCCGCACC
TTCGGATATACTGTCAAGCGAACCACAGTCAGAACGCCCTCCTGGGCGGTGGACATGATGAGATTCAATATTAATGAC
TTTCTTCCCCCAGGAGGGGGCTCAAACCCCCGCTCTGTGCCCTTTGAATACTACAGAATAAGAAAGGTTAAGGTTGAA
TTCTGGCCCTGCTCCCCGATCACCCAGGGTGACAGGGGAGTGGGCTCCAGTGCTGTTATTTTAGATGATAACTTTGTA
ACAAAGGCCACAGCCCTCACCTATGACCCCTATGTAAACTACTCCTCCCGCCATACCATAACCCAGCCCTTCTCCTACCA
CTCCCGGTACTTTACCCCCAAACCTGTCCTAGATTCCACTATTGATTACTTCCAACCAAACAACAAAAGAAACCAGCTGT
GGCTGAGACTACAAACTGCTGGAAATGTAGACCACGTAGGCCTCGGCACTGCGTTCGAAAACAGTATATACGACCAG
GAATACAATATCCGTGTAACCATGTATGTACAATTCAGAGAATTTAATTTTAAAGACCCCCCACTTAACCCT--------

>EF421967_pcv2b

ATGACGTATCCAAGGAGGCGTTACCGGAGAAGAAGACACCGCCCCCGCAGCCATCTTGGCCAGATCCTCCGCCGCCG
CCCCTGGCTCGTCCACCCCCGCCACCGTTACCGCTGGAGAAGGAAAAATGGCATCTTCAACACCCGCCTCTCCCGCACC
TTCGGATATACTGTCAAGCGAACCACAGTCAGAACGCCCTCCTGGGCGGTGGACATGATGAGATTCAATATTAATGAC
TTTCTTCCCCCAGGAGGGGGCTCAAACCCCCGCTCTGTGCCCTTTGAATACTACAGAATAAGAAAGGTTAAGGTTGAA
TTCTGGCCCTGCTCCCCGATCACCCAGGGTGACAGGGGAGTGGGCTCCAGGGCTGTTATTCTAGATGATAACTTTGTA
ACAAACGCCCCAGTCCTCACCTATGACCCCTATGTAAACTACTCCTCCCGCCATACCATAACCCAGCCCTTCTCCTACCA
CTCCCGCTACGTTACCCCCAAACCTGTCCTACATTCCACTATTGATTACTTCCAACCAAACAACAAAAGAAATCAGCTGT
GGCTGAGACTACAAACTGCTGGTAATGTAGACCACGTAGGCCTTGGCACTGGGTTCGAAAACAGTATATACGCCCAG
GAATACAATATTCGTGTAACCAGGGATGTACAATTCAGAGAATTTTATTTTAAAGACCCCCCCTTAACCCT--------

>AY613854_pcv2b

ATGACGTATCCAAGGAGGCGTTACCGGAGAAGAAGACACCGCCCCCGCAGCCATCTTGGCCAGATCCTCCGCCGCCG
CCCCTGGCTCGTCCACCCCCGCCACCGTTACCGCTGGAGAAGGAAAAATGGCATCTTCAACACCCGCCTCTCCCGCACC
TTCGGATATACTGTCAAGCGAACCACAGTCAGAACGCCCTCCTGGGCGGTGGACATGATGAGATTCAATATTAATGAC
TTTCTTCCCCCAGGAGGGGGCTCAAACCCCCGCTCTGTGCCCTTTGAATACTACAGAATAAGAAAGGTTAAGGTTGAA
TTCTGGCCCTGCTCCCCGATCACCCAGGGTGACAGGGGAGTGGGCTCCAGTGCTGTTATTCTAGATGATAACTTTGTA
ACAAAGGCCACAGCCCTCACCTATGACCCCTATGTAAACTACTCCTCCCGCCATACCATAACCCAGCCCTTCTCCTACCC
CTCCCGCTACTTTACCCCCAAACCTGTCCTAGATTCCACTATTGATTACTTCCAACCAAACAACAAAAGAAATCAGCTGT
GGCTGAGACTACAAACTGCTGGAAATGTAGACCACGTAGGCCTCGGCACTGCGTTCGAAAACAGTATATACGACCAG
GAATACAATATCCGTGTAACCATGTATGTACAATTCAGAGAATTTAATCTTAAAGACCCCCCACTTAACCCT--------

>AB462387_pcv2b

ATGACGTATCCAAGGAGGCGTTACCGGAGAAGAAGACACCGCCCCCACAGCCATCTTGGCCAGATCCTCCGCCGCCG
CCCCTGGCTCGTCCACCCCCGCCACCGTTACCGCTGGAGAAGGAAAAATGGCATCTTCAACACCCGCCTCTCCCGCACC
TTCGGATATACTGTCAAGCGAACCACAGTCAGAACGCCCTCCTGGGCGGTGGACATGATGAGATTCAATATTAATGAC
TTTCTTCCCCCAGGAGGGGGCTCAAACCCCCGCTCTGTGCCCTTTGAATACTACAGAATAAGAAAGGTTAAGGTTGAA
TTCTGGCCCTGCTCCCCGATCACCCAGGGTGACAGGGGAGTGGGCTCCAGTGCTGTTATTCTAGATGATAACTTTGTA
ACAAAGGCCACAGCCCTCACCTATGACCCCTATGTAAACTACTCCTCCCGCCATACCATAACCCAGCCCTTCTCCTACCA
CTCCCGCTACTTTACCCCCAAACCTGTCCTAGATTCCACTATTGATTACTTCCAACCAAACAACAAAAGAAATCAGCTGT
GGCTGAGACTACAAACTGCTGGAAATGTAGACCACGTAGGCCTCGGCACTGCGTTCGAAAACAGTATATACGACCAG
GAATACAATATCCGTGTAACCATGTATGTACAATTCAGAGAATTTAATCTTAAAGACCCCCCACTTAAACCT--------

>FJ905469_pcv2b

ATGACGTATCCAAGGAGGCGTTACCGGAGAAGAAGACACCGCCCCCGCAGCCATCTTGGCCAGATCCTCCGCCGCCG
CCCCTGGCTCGTCCACCCCCGCCACCGTTACCGCTGGAGAAGGAAAAATGGCATCTTCAACACCCGCCTCTCCCGCACC
TTCGGATATACTGTCAAGCGAACCACAGTGAGAACACCCTCCTGGGCGGTGGACATGATGAGATTCAATATTAATGAC
TTTCTTCCCCCAGGAGGGGGCTCAAACCCCCGCTCTGTGCCCTTTGAATACTACAGAATAAGAAAGGTTAAGGTTGAA
TTCTGGCCCTGCTCCCCGATCACCCCCGGTGACAGGGGAGTGGGCTCCAGTGCTGTTATTCTAGATGATAACTTTGTAA
CAAAGGCCACAGCCCTCACCTATGACCCCTATGTAAACTACTCCTCCCGCCATACCATAACCCAGCCCTTCTCCTACCAC
TCCCGCTACTTTACCCCCAAACCTGTCCTAGATTCCACTATTGATTACTTCCAACCAAACAACAAAAGAAACCAGCTATG
GCTGAGACTACAAACTGCTGGAAATGTAGACCACGTAGGCCTCGGCACTGCGTTCGAAAACAGTATATACGACCAGG
AATACAATATCCGTGTAACCATGTATGTACAATTCAGAGAATTTAATCTTAAAGACCCCCCACTTAACCCT--------

>JF317573_pcv2b

ATGACGTATCCAAGGAGGCGTTACCGGAGAAGAAGACACCGCCCCCGCAGCCATCTTGGCCAGATCCTCCGCCGCCG
CCCCTGGCTCGTCCACCCCCGCCACCGTTACCGCTGGAGAAGGAAAAATGGCATCTTCAACACCCGCCTCTCCCGGAC
CTTCGGATATACTGTCAAGCGAACCACAGTGAGAACACCCTCCTGGGCGGTGGACATGATGAGATTCAATATTAATGA
CTTTCTTCCCCCAGGAGGGGGCTCCAACCCCCGCTCTGTGCCCTTTGAATACTACAGAATAAGAAAGGTTAAGGTTGA
ATTCTGGCCCTGCTCCCCGATCACCCAGGGTGACAGGGGAGTGGGCTCCAGTGCTGTTATTCTAGATGATAACTTTGT
AACAAAGGCCACAGCCCTCACCTATGACCCCTATGTAAACTACTCCTCCCGCCATACCATAACCCAGCCCTTCTCCTACC
ACTCCCGCTACTTTACCCCCAAACCTGTCCTAGATTCCACTATTGATTACTTCCAACCAAACAACAAAAGAAACCAGCTG
TGGCTGAGACTACAAACTGCTGGAAATGTAGACCACGTAGGCCTCGGCACTGCGTTCGAAAACAGTATATACGACCA
GGAATACAATATCCGTGTAACCATGTATGTACAATTCAGAGAATTTAATCTTAAAGACCCCCCACTTAACCCT--------

>FJ905462_pcv2b

ATGACGTATCCAAGGAGGCGTTACCGGAGAAGAAGACACCGCCCCCGCAGCCATCTTGGCCAGATCCTCCGCCGCCG
CCCCTGGCTCGTCCACCCCCGCCACCGTTACCGCTGGAGAAGGAAAAATGGCATCTTCAACACCCGCCTCTCCCGCACC
TTCGGATATACTGTCAAGCGAACCACAGTGAGAACGCCCTCCTGGGCGGTGGACATGATGAGATTCAATATTAATGAC
TTTCTTCCCCCAGGAGGGGGCTCAAACCCCCGCTCTGTGCCCTATGAATACTACAGAATAAGAAAGGTTAAGGTTGAA
TTCTGGCCCTGCTCCCCGATCACCCAGGGTGACAGGGGAGTGGGCTCCAGTGCTGTTATTCTAGATGATAACTTTGTA
ACAAAGGCCACAGCCCTCACCTATGACCCCTATGTAAACTACTCCTCCCGCCATACCATAACCCAGCCCTTCTCCTACCA
CTCCCGCTACTTTACCCCCAAACCTGTCCTAGATTCCACTATTGATTACTTCCAACCAAACAACAAAAGAAACCAGCTGT
GGCTGAGACTGCAAACTGCTGGAAATGTAGACCACGTAGGCCTCGGCACTGCGTTCGAAAACAGTATATACGACCAG
GAATACAATATCCGTGTAACCATGTATGTACAATTCAGAGAATTTAATCTTAAAGACCCCCCACTTAAACCT--------

>AY321985_pcv2b

ATGACGTATCCAAGGAGGCGTTACCGGAGAAGAAGACACCGCCCCCGCAGCCATCTTGGCCAGATCCTCCGCCGCCG
CCCCTGGCTCGTCCACCCCCGCCACCGTTACCGCTGGAGAAGGAAAAATGGCATCTTCAACACCCGCCTCTCCCGCACC
TTCGGATATACTGTCAAGCGAACCACAGTCAGAACGCCCTCCTGGGCGGTGGACATGATGAGATTCAATATTAATGAC
TTTCTTCCCCCAGGAGGGGGCTCAAACCCCCGCTCTGTGCCCTTTGAATACTACAGAATAAGAAAGGTTAAGGTTGAA
TTCTGGCCCTGCTCCCCGATCACCCAGGGTGACAGGGGAGTGGGCTCCAGTGCTGTTATTCTAGATGATAACTTTGTA
ACAAAGGCCACAGCCCTCACCTATGACCCCTATGTAAACTACTCCTCCCGCCATACCATAACCCAGCCCTTCTCCTACCA
CTCCCGCTACTTTACCCCCAAACCTGTCCTAGATTCCACTATTGATTACTTCCAACCAAACAACAAAAGAAACCAGCTGT
GGCTGAGACTACAAACTGCTGGAAATGTAGACCACGTAGGCCTCGGCACTGCGTTCGAAAACAGTATATACGACCAG
GAATACAATATCCGTGTAACCATGTATGTACAATTCAGAGAATTTAATCTTAAAGACCCCCCACTTAACCCT--------

>EU547456_pcv2b

ATGACGTATCCAAGGAGGCGTTACCGGAGAAGAAGACACCGCCCCCGCAGCCATCTTGGCCAGATCCTCCGCCGCCG
CCCCTGGCTCGTCCACCCCCGCCACCGTTACCGCTGGAGAAGGAAAAATGGCATCTTCAACACCCGCCTCTCCCGCACC
TTCGGATATACTGTCAAGCGAACCACAGTCAGAACGCCCTCCTGGGCGGTGGACATGATGAGATTCAATATTAATGAC
TTTCTTCCCCCAGGAGGGGGCTCAAACCCCCGCTCTGTGCCCTTTGAATACTACAGAATAAGAAAGGTTAAGGTTGAA
TTCTGGCCCTGCTCCCCGATCACCCAGGGTGACAGGGGAGTGGGCTCCAGTGCTGTTATTCTAGATGATAACTTTGTA
ACAAAGGCCACAGCCCTCACCTATGACCCCTATGTAAACTACTCCTCCCGCCATACCATAACCCAGCCCTTCTCCTACCA
CTCCCGCTACTTTACCCCCAAACCTGTCCTAGATTCCACTATTGATTACTTCCAACCAAACAACAAAAGAAACCAGCTGT
GGCTGAGACTACAAACTGCTGGAAATGTAGACCACGTAGGCCTCGGCACTGCGTTCGAAAACAGTATATACGACCAG
GAATACAATATCCGTGTAACCATGTATGTACAATTCAGAGAATTTAATCTTAAAGACCCCCCACTTAACCCT--------

>AF055393_pcv2b

ATGACGTATCCAAGGAGGCGTTACCGGAGAAGAAGACACCGCCCCCGCAGCCATCTTGGCCAGATCCTCCGCCGCCG
CCCCTGGCTCGTCCACCCCCGCCACCGTTACCGCTGGAGAAGGAAAAATGGCATCTTCAACACCCGCCTCTCCCGCACC
TTCGGATATACTGTCAAGCGAACCACAGTCAGAACGCCCTCCTGGGCGGTGGACATGATGAGATTCAATATTAATGAC
TTTCTTCCCCCAGGAGGGGGCTCAAACCCCCGCTCTGTGCCCTTTGAATACTACAGAATAAGAAAGGTTAAGGTTGAA
TTCTGGCCCTGCTCCCCGATCACCCAGGGTGACAGGGGAGTGGGCTCCAGTGCTGTTATTCTAGATGATAACTTTGTA
ACAAAGGCCACAGCCCTCACCTATGACCCCTATGTAAACTACTCCTCCCGCCATACCATAACCCAGCCCTTCTCCTACCA
CTCCCGCTACTTTACCCCCAAACCTGTCCTAGATTCCACTATTGATTACTTCCAACCAAACAACAAAAGAAACCAGCTGT
GGCTGAGACTACAAACTGCTGGAAATGTAGACCACGTAGGCCTCGGCACTGCGTTCGAAAACAGTATATACGACCAG
GAATACAATATCCGTGTAACCATGTATGTACAATTCAGAGAATTTAATCTTAAAGACCCCCCACTTAACCCT--------

>EF565357_pcv2b

ATGACGTATCCAAGGAGGCGTTACCGGAGAAGAAGACACCGCCCCCGCAGCCATCTTGGCCAGATCCTCCGCCGCCG
CCCCTGGCTCGTCCACCCCCGCCACCGTTACCGCTGGAGAAGGAAAAATGGCATCTTCAACACCCGCCTCTCCCGCACC
TTCGGATATACTGTCAAGCGAACCACAGTCAGAACGCCCTCCTGGGCGGTGGACATGATGAGATTCAATATTAATGAC
TTTCTTCCCCCAGGAGGGGGCTCAAACCCCCGCTCTGTGCCCTTTGAATACTACAGAATAAGAAAGGTTAAGGTTGAA
TTCTGGCCCTGCTCCCCGATCACCCAGGGTGACAGGGGAGTGGGCTCCAGTGCTGTTATTCTAGATGATAACTTTGTA
ACAAAGGCCACAGCCCTCACCTATGACCCCTATGTAAACTACTCCTCCCGCCATACCATAACCCAGCCCTTCTCCTACCA
CTCCCGCTACTTTACCCCCAAACCTGTCCTAGATTCCACTATTGATTACTTCCAACCAAACAACAAAAGAAACCAGCTGT
GGCTGAGACTACAAACTGCTGGAAATGTAGACCACGTAGGCCTCGGCACTGCGTTCGAAAACAGTATATACGACCAG
GAATACAATATCCGTGTAACCATGTATGTACAATTCAGAGAATTTAATCTTAAAGACCCCCCACTTAACCCT--------

>AY321986_pcv2b

ATGACGTATCCAAGGAGGCGTTACCGGAGAAGAAGACACCGCCCCCGCAGCCATCTTGGCCAGATCCTCCGCCGCCG
CCCCTGGCTCGTCCACCCCCGCCACCGTTACCGCTGGAGAAGGAAAAATGGCATCTTCAACACCCGCCTCTCCCGCACC
TTCGGATATACTGTCAAGCGAACCACAGTCAGAACGCCCTCCTGGGCGGTGGACATGATGAGATTCAATATTAATGAC
TTTCTTCCCCCAGGAGGGGGCTCAAACCCCCGCTCTGTGCCCTTTGAATACTACAGAATAAGAAAGGTTAAGGTTGAA
TTCTGGCCCTGCTCCCCGATCACCCAGGGTGACAGGGGAGTGGGCTCCAGTGCTGTTATTCTAGATGATAACTTTGTA
ACAAAGGCCACAGCCCTCACCTATGACCCCTATGTAAACTACTCCTCCCGCCATACCATAACCCAGCCCTTCTCCTACCA
CTCCCGCTACTTTACCCCCAAACCTGTCCTAGATTCCACTATTGATTACTTCCAACCAAACAACAAAAGAAACCAGCTGT
GGCTGAGACTACAAACTGCTGGAAATGTAGACCACGTAGGCCTCGGCACTGCGTTCGAAAACAGTATATACGACCAG
GAATACAATATCCGTGTAACCATGTATGTACAATTCAGAGAATTTAATCTTAAAGACCCCCCACTTAACCCT--------

>AY321984_pcv2b

ATGACGTATCCAAGGAGGCGTTACCGGAGAAGAAGACACCGCCCCCGCAGCCATCTTGGCCAGATCCTCCGCCGCCG
CCCCTGGCTCGTCCACCCCCGCCACCGTTACCGCTGGAGAAGGAAAAATGGCATCTTCAACACCCGCCTCTCCCGCACC
TTCGGATATACTGTCAAGCGAACCACAGTCAGAACGCCCTCCTGGGCGGTGGACATGATGAGATTCAATATTAATGAC
TTTCTTCCCCCAGGAGGGGGCTCAAACCCCCGCTCTGTGCCCTTTGAATACTACAGAATAAGAAAGGTTAAGGTTGAA
TTCTGGCCCTGCTCCCCGATCACCCAGGGTGACAGGGGAGTGGGCTCCAGTGCTGTTATTCTAGATGATAACTTTGTA
ACAAAGGCCACAGCCCTCACCTATGACCCCTATGTAAACTACTCCTCCCGCCATACCATAACCCAGCCCTTCTCCTACCA
CTCCCGCTACTTTACCCCCAAACCTGTCCTAGATTCCACTATTGATTACTTCCAACCAAACAACAAAAGAAACCAGCTGT
GGCTGAGACTACAAACTGCTGGAAATGTAGACCACGTAGGCCTCGGCACTGCGTTCGAAAACAGTATATACGACCAG
GAATACAATATCCGTGTAACCATGTATGTACAATTCAGAGAATTTAATCTTAAAGACCCCCCACTTAACCCT--------

>FN687856_pcv2b

ATGACGTATCCAAGGAGGCGTTACCGGAGAAGAAGACACCGCCCCCGCAGCCATCTTGGCCAGATCCTCCGCCGCCG
CCCCTGGCTCGTCCACCCCCGCCACCGTTACCGCTGGAGAAGAAAGAATGGCATCTTCAACACCCGCCTCTCCCGCACC
TTCGGATATACTGTCAAGCGAACCACAGTCAGAACGCCCTCCTGGGCGGTGGACATGATGAGATTCAATATTAATGAC
TTTCTTCCCCCAGGAGGGGGCTCAAACCCCCGCTCTGTGCCCTTTGAATACTACAGAATAAGAAAGGTTAAGGTTGAA
TTCTGGCCCTGCTCCCCGATCACCCAGGGTGACAGGGGAGTGGGCTCCAGTGCTGTTATTCTAGATGATAACTTTGTA
ACAAAGGCCACAGCCCTCACCTATGACCCCTATGTAAACTACTCCTCCCGCCATACCATAACCCAGCCCTTCTCCTACCA
CTCCCGCTACTTTACCCCCAAACCTGTCCTAGATTCCACTATTGATTACTTCCAACCAAACAACAAAAGAAACCAGCTGT
GGCTGAGACTACAAACTGCTGGAAATGTAGACCACGTAGGCCTCGGCACTGCGTTCGAAAACAGTATATACGACCAG
GAATACAATATCCGTGTAACCATGTATGTACAATTCAGAGAATTTAATCTTAAAGACCCCCCACTTAACCCT--------

>EU545550_pcv2b

ATGACGTATCCAAGGAGGCGTTACCGGAGAAGAAGACACCGCCCCCGCAGCCATCTTGGCCAGATCCTCCGCCGCCG
CCCCTGGCTCGTCCACCCCCGCCACCGTTACCGCTGGAGAAGGAAAAATGGCATCTTCAACACCCGCCTCTCCCGCACC
TTCGGATATACTGTCAAGCGAACCACAGTCAGAACGCCCTCCTGGGCGGTGGACATGATGAGATTCAATATTAATGAC
TTTCTTCCCCCAGGAGGGGGCTCAAACCCCCGCTCTGTGCCCTTTGAATACTACAGAATAAGAAAGGTCAAGGTTGAA
TTCTGGCCCTGCTCCCCGATCACCCAGGGTGACAGGGGAGTGGGCTCCAGTGCTGTTATTCTAGATGATAACTTTGTA
ACAAAGGCCACAGCCCTCACCTATGACCCCTATGTAAACTACTCCTCCCGCCATACCATAACCCAGCCCTTCTCCTACCA
CTCCCGCTACTTTACCCCCAAACCTGTCCTAGATTCCACTATTGATTACTTCCAACCAAACAACAAAAGAAACCAGCTGT
GGCTGAGACTACAAACTGCTGGAAATGTAGACCACGTAGGCCTCGGCACTGCGTTCGAAAACAGTATATACGACCAG
GAATACAATATCCGTGTTACCATGTATGTACAATTCAGAGAATTTAATCTTAAAGACCCCCCACTTAACCCT--------

>HM755880_pcv2d

ATGACGTATCCAAGGAGGCGTTTCCGCAGACGAAGACACCGCCCCCGCAGCCATCTTGGCCAGATCCTCCGCTGCCGC
CCCTGGCTCGTCCACCCCCGCCACCGTTACCGCTGGAGAAGGAAAAATGGCATCTTCAACACCCGCCTCTCCCGCACCA
TAGGTTATACTGTCAAGAAAACCACAGTCAGAACGCCCTCCTGGAATGTGGACATGATGAGATTTAATATTAATGATT
TTCTTCCCCCAGGAGGGGGCTCAAACCCCCTCACTGTGCCCTTTGAATACTACAGAATAAGGAAGGTTAAGGTTGAAT
TCTGGCCCTGCTCCCCAATCACCCAGGGTGACAGGGGAGTGGGCTCCACTGCTGTTATTTTAGATGATAACTTTGTAAC
AAAGGCCAATGCCCTAACCTATGACCCCTATGTAAACTACTCCTCCCGCCATACCATAACCCAGCCCTTCTCCTACCACT
CCCGGTACTTTACCCCGAAACCTGTCCTTGATAGGACAATCGATTACTTCCAACCCAATAACAAAAGAAATCAACTCTG
GCTGAGACTACAAACTACTGGAAATGTAGACCATGTAGGCCTCGGCACTGCGTTCGAAAACAGTATATACGACCAGG
ACTACAATATCCGTATAACCATGTATGTACAATTCAGAGAATTTAATCTTAAAGACCCCCCACTTAACCCT--------

>HM535640_pcv2d

ATGACGTATCCAAGGAGGCGTTTCCGCAGACGAAGACACCGCCCCCGCAGCCATCTTGGCCAGATCCTCCGCTGCCGC
CCCTGGCTCGTCCACCCCCGCCACCGTTACCGCTGGAGAAGGAAAAATGGCATCTTCAACACCCGCCTCTCCCGCACCA
TAGGTTATACTGTCAAGAAAACCACAGTCAGAACGCCCTCCTGGAATGTGGACATGATGAGATTTAATATTAATGATT
TTCTTCCCCCAGGAGGGGGCTCAAACCCCCTCACTGTGCCCTTTGAATACTACAGAATAAGGAAGGTTAAGGTTGAAT
TCTGGCCCTGCTCCCCAATCACCCAGGGTGACAGGGGAGTGGGCTCCACTGCTGTTATTCTAGATGATAACTTTGTAA
CAAAGGCCAATGCCCTAACCTATGACCCCTATGTAAACTACTCCTCCCGCCATACCATAACCCAGCCCTTCTCCTACCAC
TCCCGGTACTTTACCCCGAAACCTGTCCTTGATAGGACAATCGATTACTTCCAACCCAATAACAAAAGAAATCAACTCT
GGCTGAGACTACAAACTACTGGAAATGTAGACCATGTAGGCCTCGGCACTGCGTTCGAAAACAGTATATACGACCAG
GACTACAATATCCGTATAACCATGTATGTACAATTCAGAGAATTTAATCTTAAAGACCCCCCACTTAACCCT--------

>HM755881_pcv2d

ATGACGTATCCAAGGAGGCGTTTCCGCAGACGAAGACACCGCCCCCGCAGCCATCTTGGCCAGATCCTCCGCTGCCGC
CCCTGGCTCGTCCACCCCCGCCACCGTTACCGCTGGAGAAGGAAAAATGGCATCTTCAACACCCGCCTCTCCCGCACCA
TGGGTTATACTGTCAAGAAAACCACAGTCAGAACGCCCTCCTGGAATGTGGACATGATGAGATTTAATATTAATGATT
TTCTTCCCCCAGGAGGGGGCTCAAACCCCCTCACTGTGCCCTTTGAATACTACAGAATAAGGAAGGTTAAGGTTGAAT
TCTGGCCCTGCTCCCCAACCACCCAGGGTGACAGGGGAGTGGGCTCCACTGCTGTTATTCTAGATGATAACTTTGTAA
CAAAGGCCAATGCCCTAACCTATGACCCCTATGTAAACTACTCCTCCCGCCATACCATAACCCAGCCCTTCTCCTACCAC
TCCCGGTACTTTACCCCGAAACCTGTCCTTGATAGGACAATCGATTACTTCCAACCCAATAACAAAAGAAATCAACTCT
GGCTGAGACTACAAACTACTGGAAATGTAGACCATGTAGGCCTCGGCACTGCGTTCGAAAACAGTATATACGACCAG
GACTACAATATCCGTATAACCATGTATGTACAATTCAGAGAATTTAATCTTAAAGACCCCCCACTTAACCCT--------

>JF272498_pcv2d

ATGACGTATCCAAGGAGGCGTTTCCGCAGACGAAGACACCGCCCCCGCAGCCATCTTGGCCAGATCCTCCGCCGCCGC
CCCTGGCTCGTCCACCCCCGCCACCGTTACCGCTGGAGAAGGAAAAATGGCATCTTCAACACCCGCCTCTCCCGCGCC
ATAGGTTATACTGTCAAGAAAACCACAGTCAGAACGCCCTCCTGGAATGTGGACATGATGAGATTTAATATTAATGAT
TTTCTTCCCCCAGGAGGGGGCTCAAACCCCCTCACTGTGCCCTTTGAATACTACAGAATAAGGAAGGTTAAGGTTGAA
TTCTGGCCCTGCTCCCCAATCACCCAGGGTGACAGGGGAGTGGGCTCCACTGCTGTTATTCTAGATGATAACTTTGTAA
CAAAGGCCAATGCCCTAACCTATGACCCCTATGTAAACTACTCCTCCCGCCATACCATAACCCAGCCCTTCTCCTACCAC
TCCCGGTACTTTACCCCGAAACCTGTCCTTGATAGGACAATCGATTACTTCCAACCCAATAACAAAAGAAATCAACTCT
GGCTGAGACTACAAACTACTGGAAATGTAGACCATGTAGGCCTCGGCACTGCGTTCGAAAACAGTATATACGACCAG
GACTACAATATCCGTATAACCATGTATGTACAATTCAGAGAATTTAATCTTAAAGACCCCCCACTTAACCCTAAG-----

>JX912914_pcv2d

ATGACGTATCCAAGGAGGCGTTTCCGCAGACGAAGACACCGCCCCCGCAGCCATCTTGGCCAGATCCTCCGCCGCCGC
CCCTGGCTCGTCCACCCCCGCCACCGTTACCGCTGGAGAAGGAAAAATGGCATCTTCAACACCCGCCTCTCCCGCACCA
TCGGTTATACTGTCAAGAAAACCACAGTCAGAACGCCCTCCTGGAATGTGGACATGATGAGATTTGATATTAATGATT
TTCTTCCCCCAGGAGGGGGCTCAAACCCCCTCACTGTACCCTTTGAATACTACAGAATAAGGAAGGTTAAGGTTGAAT
TCTGGCCCTGCTCCCCAATCACCCAGGGTGACAGGGGAGTGGGCTCCACTGCTGTTATTCTAGATGATAACTTTGTAA
CAAAGGCCAATGCCCTAACCTATGACCCCTATGTAAACTACTCCTCCCGCCATACCATAACCCAGCCCTTCTCCTACCAC
TCCCGGTACTTTACCCCGAAACCTGTCCTTGATAGGACAATCGATTACTTCCAACCCAATAACAAAAGAAATCAACTCT
GGCTGAGACTACAAACTACTGGAAATGTAGACCATGTAGGCCTCGGCACTGCGTTCGAAAACAGTATATACGACCAG
GACTACAATATCCGTATAACCATGTATGTACAATTCAGAGAATTTAATCTTAAAGACCCCCCACTTAACCCTAAG-----

>JX204386_pcv2d

ATGACGTATCCAAGGAGGCGTTTCCGCAGACGAAGACACCGCCCCCGCAGCCATCTTGGCCAGATCCTCCGCCGCCGC
CCCTGGCTCGTCCACCCCCGCCACCGTTACCGCTGGAGAAGGAAAAATGGCATCTTCAACACCCACCTCTCCCGCACCA
TAGGTTATACTGTCAAGAAAACCACAGTCAGAACACCCTCCTGGAATGTGGACATGATGAGATTTAATATTAATGATTT
TCTTCCCCCAGGAGGGGGCTCAAACCCCCTCACTGTACCCTTTGAATACTACAGAATAAGGAAGGTTAAGGTTGAATT
CTGGCCCTGCTCCCCAATCACCCAGGGTGACAGGGGAGTGGGCTCCACTGCTGTTATTCTAGATGATAACTTTGTAAC
AAAGGCCAATGCCCTAACCTATGACCCCTATGTAAACTACTCCTCCCGCCATACCATAACCCAGCCCTTCTCCTACCACT
CCCGGTACTTTACCCCGAAACCTGTCCTTGATAGGACAATCGATTACTTCCAACCCAATAACAAAAGAAATCAACTCTG
GCTGAGACTACAAACTACTGGAAATGTAGACCATGTAGGCCTCGGCACTGCGTTCGAAAACAGTATATACGACCAGG
ACTACAATATCCGTATAACCATGTATGTACAATTCAGAGAATTTAATCTTAAAGACCCCCCACTTAACCCTAAG-----

>KC800639_pcv2d

ATGACGTATCCAAGGAGGCGTTTCCGCAGACGAAGACACCGCCCCCGCAGCCATCTTGGCCAGATCCTCCGCCGCCGC
CCCTGGCTCGTCCACCCCCGCCACCGTTACCGCTGGAGAAGGAAAAATGGCATCTTCAACACCCGCCTCTCCCGCACCA
TCGGTTATACTGTCAAGAAAACCACAGTCAGAACGCCCTCCTGGAATGTGGACATGATGAGATTTAATATTAATGATT
TTCTTCCCCCAGGAGGGGGCTCAAACCCCCTCACTGTGCCCTTTGAATACTACAGAATAAGGAAGGTTAAGGTTGAAT
TCTGGCCCTGCTCCCCAATCACCCAGGGTGACAGGGGAGTGGGCTCCACTGCTGTTATTCTAGATGATAACTTTGTAA
CAAAGGCCAATGCCCTAACCTATGACCCCTATGTAAACTACTCCTCCCGCCATACCATAACGGAGGCCTTCTCCTACCA
CTACCGGTACTTTACCCCGAAGCCTGTCCTTGATAGGACAATCGATTACTTCCAACCCAATAACAAAAGAAATCAACTC
TGGCTGAGACTACAAACTACTGGAAATGTAGACCATGTAGGCCTCGGCACTGCGTTCGAAAGCAGTATATACGACCA
GGACTGCAATATCCGTATAACCATGTATGTACAATTCAGAGAATTTAATCTTAAAGACCCCCCACTTAACCCT--------

>HQ395033_pcv2d

ATGACGTATCCAAGGAGGCGTTTCCGCAGACGAAGACACCGCCCCCGCAGCCATCTTGGCCAGATCCTCCGACGCCG
CCCCTGGCTCGTCCACCCCCGCCACCGTTACCGCTGGAGAAGGAAAAATGGCATCTTCAACACCCGCCTCTCCCGCACC
ATCGGTTATACTGTCAAGAAAACCACAGTCAGAACGCCCTCCTGGAATGTGGACATGATGAGATTTAATATTAATGAT
TTTCTTCCCCCAGGAGGGGGCTCAAACCCCCTCACTGTGCCCTTTGAATACTACAGAATAAGGAAGGTTAAGGTTGAA
TTCTGGCCTTGCTCCCCAATCACCCAGGGTGACAGGGGAGTGGGCTCCACTGCTGTTATTCTAGATGATAACTTTGTAA
CAAAGGCCAATGCCCTAACCTATGACCCCTATGTAAACTACTCCTCCCGCCATACCCTAACCCAGCCCTTCTCCTACCAC
TCCCGGTACTTTACCCCGAAACCTGTCCTTGATAGGACAATCGATTACTTCCAACCCAATAACAAAAGAAATCAACTCT
GGCTGAGACTACAAACTACTGGAAATGTAGACCATGTAGGCCTCGGCACTGCGTTCGAAAACAGTATATACGACCAG
GACTACAATATCCGTATAACCATGTATGTACAATTCAGAGAATTTAATCTTAAAGACCCCCCACTTAACCCTAAA-----

>JN411100_pcv2d

ATGACGTATCCAAGGAGGCGTTTCCGCAGACGAAGACACCGCCCCCGCAGCCATCTTGGCCAGATCCTCCGCCGCCGC
CCCTGGCTCGTCCACCCCCGCCACCGTTACCGCTGGAGAAGGAAAAATGGCATCTTCAACACCCGCCTCTCCCGCACCA
TCGGTTATACTGTCAAGAAAACCACAGTCAGAACGCCCTCCTGGAATGTGGACATGATGAGATTTAATATTAATGATT
TTCTTCCCCCGGGAGGGGGCTCAAACCCCCTCACTGTGCCCTTTGAATACTACAGAATAAGGAAGGTTAAGGTTGAAT
TCTGGCCCTGCTCCCCAATCACCCAGGGTGACAGGGGAGTGGGCTCCACTGCTGTTATTCTAGATGATAACTTTGTAA
CAAAGGCCAATGCCCTAACCTATGACCCCTATGTAAACTACTCCTCCCGCCATACCATAACCCAGCCCTTCTCCTACCAC
TCCCGGTACTTTACCCCGAAACCTGTCCTTGATAGGACAATCGATTACTTCCAACCCAATAACAAAAGAAATCAACTCT
GGCTGAGACTACAAACTACTGGAAATGTAGACCATGTAGGCCTCGGCACTGCGTTCGAAAACAGTATATACGACCAG
GACTACAACATCCGTATAACCATGTATGTACAATTCAGAGAATTTAATCTTAAAGACCCCCCACTTAACCCTAAG-----

>JF718784_pcv2d

ATGACGTATCCAAGGAGGCGTTTCCGCAGACGAAGACACCGCCCCCGCAGCCATCTTGCCCAGATCCTCCGCCGCCGC
CCCTGGCTCGTCCACCCCCGCCACCGTTACCGCTGGAGAAGGAAAAATGGCATCTTCAACACCCGCCTCTCCCGCACCA
TCGGTTATACTGTCAAGAAAACCACAGTCAGAACGCCCTCCTGGAATGTGGACATGATGAGATTTAATATTAATGATT
TTCTTCCCCCAGGAGGGGGCTCAAACCCCCTCACTGTGCCCTTTGAATACTACAGAATAAGGAAGGTTAAGGTTGAAT
TCTGGCCCTGCTCCCCAATCACCCAGGGTGACAGGGGAGTGGGCTCCACTGCTGTTATTCTAGATGGTAACTTTGTAA
CAAAGGCCAATGCCCTAACCTATGACCCCTATGTAAACTACTCCTCCCGCCATACCATAACCCAGCCCTTCTCCTACCAC
TCCCGGTACTTTACCCCGAAACCTGTCCTTGATAGGACAATCGATTACTTCCAACCCAATAACAAAAGAAATCAACTCT
GGCTGAGACTACAAACTACTGGAAATGTAGACCATGTAGGCCTCGGCACTGCGTTCGAAAACAGTATATACGACCAG
GACTACAACATCCGTATAACCATGTATGTACAATTCAGAGAATTTAATCTTAAAGACCCCCCACTTAACCCTAAG-----

>JX406419_pcv2d

ATGACGTATCCAAGGAGGCGTTTCCGCAGACGAAGACACCGCCCCCGCAGCCATCTTGGCCAGATCCTCCGCCGCCGC
CCCTGGCTCGTCCACCCCCGCCACCGTTACCGCTGGAGAAGGAAAAATGGCATCTTCAACACCCGCCTCTCCCGCACCA
TCGGTTATACTGTCAAGAAAACCACAGTCAGAACGCCCTCCTGGAATGTGGACATGATGAGATTTAATATTAATGATT
TTCTTCCCCCAGGAGGGGGCTCAAACCCCCTCACTGTGCCCTTTGAATACTACAGAATAAGGAAGGTTAAGGTTGAAT
TCTGGCCCTGCTCCCCAATCACCCAGGGTGACAGGGGAGTGGGCTCCACTGCTGTTATTCTAGATGATAACTTTGTAA
CAAAGGCCAATGCCCTAACCTATGACCCCTATGTAAACTACTCCTCCCGCCATACCATAACCCAGCCCTTCTCCTACCAC
TCCCGGTACTTCACCCCGAAACCCGTCCTTGATAGGACAATCGATTACTTCCAACCCAATAACAAAAGAAATCAACTCT
GGCTGAGACTACAAACTACTGGAAATGTAGACCATGTAGGCCTCGGCACTGCGTTCGAAAACAGTATATGCGACCAG
GACTACAATATCCGTATAACCATGTATGTACAATTCAGAGAATTTAATCTTAAAGACCCCCCACTTAACCCTAAG-----

>KC473168_pcv2d

ATGACGTATCCAAGGAGGCGTTTCCGCAGACGAAGACACCGCCCCCGCAGCCATCTTGGCCAGATCCTCCGCCGCCGC
CCCTGGCTCGTCCACCCCCGCCACCGTTACCGCTGGAGAAGGAAAAATGGCATCTTCAACACCCGCCTCTCCCGCACCA
TCGGTTATACTGTCAAGAAAACCACAGTCAGAACGCCCTCCTGGAATGTGGACATGATGAGATTTAATATTAATGATT
TTCTTCCCCCAGGAGGGGGCTCAAACCCCCTCACTGTGCCCTTTGAATACTACAGAATAAGGAAGGTAAAGGTTGAAT
TCTGGCCCTGCTCCCCAATCACCCAGGGTGACAGGGGAGTGGGCTCCACTGCTGTTATTCTAGATGATAACTTTGTAA
CAAAGGCCAATGCCCTAACCTATGACCCCTATGTAAACTACTCCTCCCGCCATACCATAACCCAGCCCTTCTCCTACCAC
TCCCGGTACTTTACCCCGAAACCTGTCCTTGATAGGACAATCGATTACTTCCAACCCAATAACAAAAGAAATCAACTCT
GGCTGAGACTACAAACTACTGGAAATGTAGACCATGTAGGCCTCGGCACTGCGTTCGAAAACAGTATATACGACCAG
GACTACAATATCCGTATAACCATGTATGTACAATTCAGAGAATTTAATCTTAAAGACCCCCCACTTAACCCTAAG-----

>FJ870972_pcv2d

ATGACGTATCCAAGGAGGCGTTTCCGCAGACGAAGACACCGCCCCCGCAGCCATCTTGGCCAGATCCTCCGCCGCCGC
CCCTGGCTCGTCCACCCCCGCCACCGTTACCGCTGGAGAAGGAAAAATAGCATCTTCAACACCCGCCTCTCCCGCACCA
TCGGTTATACTGTCAAGAAAACCACAGTCAGAACGCCCTCCTGGAATGTGGACATGATGAGATTTAATATTAATGATT
TTCTTCCCCCAGGAGGGGGCTCAAACCCCCTCACTGTGCCCTTTGAATACTACAGAATAAGGAAGGTAAAGGTTGAAT
TCTGGCCCTGCTCCCCAATCACCCAGGGTGACAGGGGAGTGGGCTCCACTGCTGTTATTCTAGATGATAACTTTGTAA
CAAAGGCCAATGCCCTAACCTATGACCCCTATGTAAACTACTCCTCCCGCCATACCATAACCCAGCCCTTCTCCTACCAC
TCCCGGTACTTTACCCCGAAACCTGTCCTTGATAGGACAATCGATTACTTCCAACCCAATAACAAAAGAAATCAACTCT
GGCTGAGACTACAAACTACTGGAAATGTAGACCATGTAGGCCTCGGCACTGCGTTCGAAAACAGTATATACGACCAG
GACTACAATATCCGTATAACCATGTATGTACAATTCAGAGAATTTAATCTTAAAGACCCCCCACTTAACCCTAAG-----

>FJ598045_pcv2d

ATGACGTATCCAAGGAGGCGTTTCCGCAGACGAAGACACCGCCCCCGCAGCCATCTTGGCCAGATCCTCCGCCGCCGC
CCCTGGCTCGTCCACCCCTGCCACCGTTACCGCTGGAGAAGGAAAAATGGCATCTTCAACACCCGCCTCTCCCGCACCA
TCGGTTATACTGTCAAGAAAACCACAGTCAGAACGCCCTCCTGGAATGTGGACATGATGGGGTTTAATATTAATGATT
TTCTTCCCCCAGGAGGGGGCTCAAACCCCCTCACTGTGCCCTTTGAATACTACAGAATAAGGAAGGTAAAGGTTGAAT
TCTGGCCCTGCTCCCCAATCACCCAGGGTGACAGGGGAGTGGGCTCCACTGCTGTTATTCTAGATGATAACCTTGTAA
CAAAGGCCAATGCCCTAACCTATGACCCCTATGTAAACTACTCCTCCCGCCATACCATAACCCAGCCCTTCTCCTACCAC
TCCCGGTACTTTACCCCGAAACCAGTCCTTGATAGGACAATCGATTACTTCCAACCCAATAACAAAAGAAATCAACTCT
GGCTGAGACTACAAACTACTGGAAATGTAGACCATGTAGGCCTCGGCACTGCGTTCGAAAACAGTATATACGACCAG
GACTACAATATCCGTATAACCATGTATGTACAATTCAGAGAATTTAATCTTAAAGACCCCCCACTTAACCCTAAG-----

>FJ870971_pcv2d

ATGACGTATCCAAGGAGGCGTTTCCGCAGACGAAGACACCGCCCCCGCAGCCATCTTGGCCAGATCCTCCGCCGCCGC
CCCTGGCTCGTCCACCCCTGCCACCGTTACCGCTGGAGAAGGAAAAATGGCATCTTCAACACCCGCCTCTCCCGCACCA
TCGGTTATACTGTCAAGAAAACCACAGTCAGAACGCCCTCCTGGAATGTGGACATGATGGGGTTTAATATTAATGATT
TTCTTCCCCCAGGAGGGGGCTCAAACCCCCTCACTGTGCCCTTTGAATACTACAGAATAAGGAAGGTAAAGGTTGAAT
TCTGGCCCTGCTCCCCAATCACCCAGGGTGACAGGGGAGTGGGCTCCACTGCTGTTATTCTAGATGATAACTTTGTAA
CAAAGGCCAATGCCCTAACCTATGACCCCTATGTAAACTACTCCTCCCGCCATACCATAACCCAGCCCTTCTCCTACCAC
TCCCGGTACTTTACCCCGAAACCAGTCCTTGATAGGACAATCGATTACTTCCAACCCAATAACAAAAGAAATCAACTCT
GGCTGAGACTACAAACTACTGGAAATGTAGACCATGTAGGCCTCGGCACTGCGTTCGAAAACAGTATATACGACCAG
GACTACAATATCCGTATAACCATGTATGTACAATTCAGAGAATTTAATCTTAAAGACCCCCCACTTAACCCTAAG-----

>HQ113118_pcv2d

ATGACGTATCCAAGGAGGCGTTTCCGCAGACGAAGACACCGCCCCCGCAGCCATCTTGGCCAGATCCTCCGCCGCCGC
CCCTGGCTCGTCCACCCCCGCCACCGTTACCGCTGGAGAAGGAAAAATGGCATCTTCAACACCCGCCTCTCCCGCACCA
TCGGTTATACTGTCAAGAAAACCACAGTCAGAACGCCCTCCTGGAATGTGCACATGATGAGATTTAATATTAATGATTT
TCTTCCCCCAGGAGGGGGCTCAAACCCCCTCACTGTGCCCTTTGAATACTACAGAATAAGGAAGGTTAAGGTTGAATT
CTGGCCCTGCTCCCCAATCACCCAGGGTGACAGGGGAGTGGGCTCCACTGCTGTTATTCTAGATGATAACTTTGTAAC
AAAGGCCAATGCCCTAACCTATGACCCCTATGTAAACTACTCCTCCCGCCATACCATAACCCAGCCCTTCTCCTACCACT
CCCGGTACTTTACCCCGAAACCTGTCCTTGATAGGACAATCGATTACTTCCAACCCAATAACAAAAGAAATCAACTCTG
GCTGAGACTACAAACTACTGGAAATGTAGACCATGTAGGCCTCGGCACTGCGTTCGAAAACAGTATATACGACCAGG
ACTACAATATCCGTATAACCATGTATATACAATTCAGAGAATTTAATCTTAAAGACCCCCCACTTAACCCTAAG-----

>JN615187_pcv2d

ATGACGTATCCAAGGAGGCGTTTCCGCAGACGAAGACACCGCCCCCGCAGCCATCTTGGCCAGATCCTCCGCCGCCGC
CCCTGGCTCGTCCACCCCCGCCACCGTTACCGCTGGAGAAGGAAAAATGGCATCTTCAACACCCGCCTCTCCCGCACCA
TCGGTTATACTGTCAAGAAAACCACAGTCAGAACGCCCTCCTGGAATGTGGACATGATGAGATTTAATATTAATGATT
TTCTTCCCCCAGGAGGGGGCTCAAACCCCCTCACTGTGCCCTTTGAATACTACAGAATAAGGAAGGTTAAGGTTGAAT
TCTGGCCCTGCTCCCCAATCACCCAGAGTGACAGGGGAGTGGGCTCCACTGCTGTTATTCTAGATGATAACTTTGTAAC
AAAGGCCAATGCCCTAACCTATGACCCCTATGTAAACTACTCCTCCCGCCATACCATAACCCAGCCCTTCTCCTACCACT
CCCGGTACTTTACCCCGAAACCTGTCCTTGATAGGACAATCGATTACTTCCAACCCAATAACAAAAGAAATCAACTCTG
GCTGAGACTACAAACTACTGGAAATGTCGACCATGTAGGCCTCGGCACTGCGTTCGAAAACAGTATATACGACCAGG
ACTACAATATCCGTATAACCATGTATGTACAATTCAGAGAATTTAATCTTAAAGACCCCCCACTTAACCCTAAG-----

>JN660055_pcv2d

ATGACGTATCCAAGGAGGCGTTTCCGCAGACGAAGACACCGCCCCCGCAGCCATCTTGGCCAGATACTCCGCCGCCG
CCCCTGGCTCGTCCACCCCCGCCACCGTTACCGCTGGAGAAGGAAAAATGGCATCTTCAACACCCGCCTCTCCCGCACC
ATCGGTTATACTGTCAAGAAAACCACAGTCAGAACGCCCTCCTGGAATGTGGACATGATGAGATTTAATATTAATGAT
TTTCTTCCCCCAGGAGGGGGCTCAAACCCCCTCACTGTGCCCTTTGAATACTACAGAATAAGGAAGGTTAAGGTTGAA
TTCTGGCCCTGCTCCCCAATCACCCAGGGTGACAGGGGAGTGGGCTCCACTGCTGTTATTCTAGATGATAACTTTGTAA
CAAAGGCCAATGCCCTAACCTATGACCCCTATGTAAACTACTCCTCCCGCCATACCATAACCCAGCCCTTCTCGTACCAC
TCCCGGTACTTTACCCCGAAACCTGTCCTTGATAGGACAATCGATTACTTCCAACCCAATAACAAAAGAAATCAACTCT
GGCTGAGACTACAAACTACTGGAAATGTAGACCATGTAGGCCTCGGCACTGCGTTCGAAAACAGTATATACGACCAG
GACTACAATATCCGTATAACCATGTATGTACAATTCAGAGAATTTAATCTTAAAGACCCCCCACTTAACCCTAAG-----

>HQ395043_pcv2d

ATGACGTATCCAAGGAGGCGTTTCCGCAGACGAAGACACCGCCCCCGCAGCCATCTTGGCCAGATCCTCCGCCGCCGC
CCCTGGCTCGTCCACCCCCGCCACCGTTACCGCTGGAGAAGGAAAAATGGCATCTTCAACACCCGCCTCTCCCGCACCA
TCGGTTATACTGTCAAGAAAACCACAGTCAGAACGCCCTCCTGGAATGTGGACATGATGAGATTTAATATTAATGATT
TTCTTCCCCCAGGAGGGGGCTCAAACCCCCTCACTGTGCCCTTTGAATACTACAGAATAAGGAAGGTTAAGGTTGAAT
TCTGGCCCTGCTCCCCAATCACCCAGGGTGACAGGGGAGTGGGCTCCACTGCTGTTATTCTAGATGATAACTTTGTAA
CAAAGGCCAATGCCCTAACCTATGACCCCTATGTAAACTACTCCTCCCGCCATACCATAACCCAGCCCTTCTCCTACCAC
TCCCGGTACTTTACCCCGAAACCTGTCCTTGATAGGACAATCGATTACTTCCAACCCAATAACAAAAGAAATCAACTCT
GGCTAAGACTACAAACTACTGGAAATGTAGACCATGTAGGCCTCGGCACTGCGTTCGAAAACAGTATATACGACCAG
GACTACAATATCCGTATAACCATGTATGTACAATTCAGAGAATTTAATCTTAAAGACCCCCCACTTAACCCTAAG-----

>HQ395061_pcv2d

ATGACGTATCCAAGGAGGCGTTTCCGCAGACGAAGACACCGCCCCCGCAGCCATCTTGGCCAGATCCTCCGCCGCCGC
CCCTGGCTCGTCCACCCCCGCCACCGTTACCGCTGGAGAAGGAAAAATGGCATCTTCAACACCCGCCTCTCCCGCACCA
TCGGTTATACTGTTAAGAAAACCACAGTCAGAACGCCCTCCTGGAATGTGGACATGATGAGATTTAATATTAATGATTT
TCTTCCCCCAGGAGGGGGCTCAAACCCCCTCACTGTGCCCTTTGAATACTACAGAATAAGGAAGGTTAAAGTTGAATT
CTGGCCCTGCTCCCCAATCACCCAGGGTGACAGGGGAGTGGGCTCCACTGCTGTTATTCTAGATGATAACTTTGTAAC
AAAGGCCAATGCCCTAACCTATGACCCCTATGTAAACTACTCCTCCCGCCATACCATAACCCAGCCCTTCTCCTACCACT
CCCGGTACTTTACCCCGAAACCTGTCCTTGATAGGACAATCGATTACTTCCAACCCAATAACAAAAGAAATCAACTCTG
GCTAAGACTACAAACTACTGGAAATGTAGACCATGTAGGCCTCGGCACTGCGTTCGAAAACAGTATATACGACCAGG
ACTACAATATCCGTATAACCATGTATGTACAATTCAGAGAATTTAATCTTAAAGACCCCCCACTTAACCCTAAG-----

>FJ870970_pcv2d

ATGACGTATCCAAGGAGGCGTTTCCGCAGACGAAGACACCGCCCCCGCAGCCATCTTGGCCAGATCCTCCGCCGCCGC
CCCTGGCTCGTCCACCCCCGCCACCGTTACCGCTGGAGAAGGAAAAATGGCATCTTCAACACCCGCCTCTCCCGCACCA
TCGGTTATACTGTCAAGAAAACCACAGTCAGAACGCCCTCCTGGAATGTGGACATGATGAGATTTAATATTAATGATT
TTCTTCCCCCAGGAGGGGGCTCAAACCCCCTCACTGTGCCCTTTGAATACTACAGAATAAGGAAGGTTAAGGTTGAAT
TCTGGCCCTGCTCCCCAATCACCCAGGGTGACAGGGGAGTGGGCTCCACTGCTGTTATTCTAGATGATAACTTTGTAA
CAAAGGCCAATGCCCTAACCTATGACCCCTATGTAAACTACTCCTCCCGCCATACCATAACCCAGCCCTTCTCCTACCAC
TCCCGGTACTTTACCCCGAAACCTGTCCTTGATAGGACAATCGATTACTTCCAACCCAATAACAAAAGAAATCAACTCT
GGCTAAGACTACAAACTACTGGAAATGTAGATCATGTAGGCCTCGGCACTGCGTTCGAAAACAGTATATACGACCAG
GACTACAATATCCGTATAACCATGTATGTACAATTCAGAGAATTTAATCTTAAAGACCCCCCACTTAACCCTAAG-----

>HM027580_pcv2d

ATGACGTATCCAAGGAGGCGTTTCCGCAGACGAAGACACCGCCCCCGCAGCCATCTTGGCCAGATCCTCCGCCGCCGC
CCCTGGCTCGTCCACCCCCGCCACCGTTACCGCTGGAGAAGGAAAAATGGCATCTTCAACACCCGCCTCTCCCGCACCA
TCGGTTATACTGTCAAGAAAACCACAGTCAGAACGCCCTCCTGGAATGTGGACATGATGAGATTTAATATTAATGATT
TTCTTCCCCCAGGAGGGGGCTCAAACCCCCTCACTGTGCCCTTTGAATACTACAGAATAAGGAAGGTTAAGGTTGAAT
TCTGGCCCTGCTCCCCAATCACCCAGGGTGACAGGGGAGTGGGCTCCACTGCTGTTATTCTAGATGATAACTTTGTAA
CAAAGGCGAATGCCCTAACCTATGACCCCCATGTAAACTACTCCTCCCGCCATACCATAACCCAGCCCTTCTCCTACCAC
TCCCGGTACTTTACCCCGAAACCTGTCCTTGATAGGACAATCGATTACTTCCAACCCAATAACAAAAGAAATCAACTCT
GGCTGAGACTACAAACTACTGGAAATGTAGACCATGTAGGCCTCGGCACTGCGTTCGAAAACAGTATATACGACCAG
GACTACAATATCCGTATAACCATGTATGTACAATTCAGAGAATTTAATCTTAAAGACCCCCCACTTAACCCTAAG-----

>JF927988_pcv2d

ATGACGTATTCAATGAGGCGTTTCCGCAGACGAAGACACCGCCCCCGCAGCCATCTTGGCCAGATCCTCCGCCGCCGC
CCCTGGCTCGTCCACCCCCGCCACCGTTACCGCTGGAGAAGGAAAAATGGCATCTTCAACACCCGCCTCTCCCGCACCA
TCGGTTATACTGTCAAGAAAACCACAGTCAGAACGCCCTCCTGGAATGTGGACATGATGAGATTTAATATTAATGATT
TTCTTCCCCCAGGAGGGGGCTCAAACCCCCTCACTGTGCCCTTTGAATACTACAGAATAAGGAAGGTTAAGGTTGAAT
TCTGGCCCTGCTCCCCAATCACCCAGGGTGACAGGGGAGTGGGCTCCACTGCTGTTATTCTAGATGATAACTTTGTAA
CAAAGGCCAATGCCCTAACCTATGACCCCTATGTAAACTACTCCTCCCGCCATACCATAACCCAGCCCTTCTCCTACCAC
TCCCGGTACTTTACCCCGAAACCTGTCCTTGATAGGACAATCGATTACTTCCAACCCAATAACAAAAGAAATCAACTCT
GGCTGAGACTACAAACTACTGGAAATGTAGACCATGTAGGCCTCGGCACTGCGTTCGAAAACAGTATATACGACCAG
GACTACAATATCCGTATAACCATGTATGTACAATTCAGAGAATTTAATCTTAAAGACCCCCCACTTAACCCTAAG-----

>JN411095_pcv2d

ATGACGTATCCAAGGAGGCGTTTCCGCAGACGAAGACACCGCCCCCGCAGCCATCTTGGCCAGATCCTCCGCCGCCGC
CCCTGGCTCGTCCACCCCCGCCACCGTTACCGCTGGAGAAGGAAAAATGGCATCTTCAACACCCGCCTCTCCCGCACCA
TCGGTTATACTGTCAAGAAAACCACAGTCAGAACGCCCTCCTGGAATGTGGACATGATGAGATTTAATATTAATGATT
TTCTTCCCCCAGGAGGGGGCTCAAACCCCCTCACTGTGCCCTTTGAATACTACAGAATAAGGAAGGTTAAGGTTGAAT
TCTGGCCCTGCTCCCCAATCACCCAGGGTGACAGGGGAGTGGGCTCCACTGCTGTTATTCTAGATGATAACTTTGTAA
CAAAGGCCAATGCCCTAACCTATGACCCCTATGTAAACTACTCCTCCCGCCATACCATAACCCAGCCCTTCTCCTACCAC
TCCCGGTACTTTACCCCGAAACCTGTCCTTGATAGGACAATCGATTACTTCCAACCCAATAACAAAAGAAATCAACTCT
GGCTGAGACTACAAACTACTGGAAATGTAGACCATGTAGGCCTCGGCACTGCGTTCGAAAACAGTATATACGACCAG
GACTACAATATCCGTATAACGATGTATGTACAATTCAGAGAATTTAATCTTAAAGACCCCCCACTTAACCCTAAG-----

>JN411096_pcv2d

ATGACGTATCCAAGGAGGCGTTTCCGCAGACGAAGACACCGCCCCCGCAGCCATCTTGGCCAGATCCTCCGCCGCCGC
CCCTGGCTCGTCCACCCCCGCCACCGTTACCGCTGGAGAAGGAAAAATGGCATCTTCAACACCCGCCTCTCCCGCACCA
TCGGTTATACTGTCAAGAAAACCACAGTCAGAACGCCCTCCTGGAATGTGGACATGGTGAGATTTAATATTAATGATT
TTCTTCCCCCAGGAGGGGGCTCAAACCCCCTCACTGTGCCCTTTGAATACTACAGAATAAGGAAGGTTAAGGTTGAAT
TCTGGCCCTGCTCCCCAATCACCCAGGGTGACAGGGGAGTGGGCTCCACTGCTGTTATTCTAGATGATAACTTTGTAA
CAAAGGCCAATGCCCTAACCTATGACCCCTATGTAAACTACTCCTCCCGCCATACCATAACCCAGCCCTTCTCCTACCAC
TCCCGGTACTTTACCCCGAAACCTGTCCTTGATAGGACAATCGATTACTTCCAACCCAATAACAAAAGAAATCAACTCT
GGCTGAGACTACAAACTACTGGAAATGTAGACCATGTAGGCCTCGGCACTGCGTTCGAAAACAGTATATACGACCAG
GACTACAATATCCGTATAACGATGTATGTACAATTCAGAGAATTTAATCTTAAAGACCCCCCACTTAACCCTAAG-----

>GQ359009_pcv2d

ATGACGTATCCAAGGAGGCGTTTCCGCGGACGAAGACACCGCCCCCGCAGCCATCTTGGCCAGATCCTCCGCCGCCG
CCCCTGGCTCGTCCACCCCCGCCACCGTTACCGCTGGAGAAGGAAAAATGGCATCTTCAACACCCGCCTCTCCCGCACC
ATCGGTTATACTGTCAAGAAAACCACAGTCAGAACGCCCTCCTGGAATGTGGACATGATGAGATTTAATATTAATGAT
TTTCTTCCCCCAGGAGGGGGCTCAAACCCCCTCACTGTGCCCTTTGAATACTACAGAATAAGGAAGGTTAAGGTTGAA
TTCTGGCCCTGCTCCCCAATCACCCAGGGTGACAGGGGAGTGGGCTCCACTGCTGTTATTCTAGATGATAACTTTGTAA
CAAAGGCCAATGCCCTAACCTATGACCCCTATGTAAACTACTCCTCCCGCCATACCATAACCCAGCCCTTCTCCTACCAC
TCCCGGTACTTTACCCCGAAACCTGTCCTTGATAGGACAATCGATTACTTCCAACCCAATAACAAAAGAAATCAACTCT
GGCTGAGACTACAAACTACTGGAAATGTAGACCATGTAGGCCTCGGCACTGCGTTCGAAAACAGTATATACGACCAG
GACTACAATATCCGTATAACGATGTATGTACAATTCAGAGAATTTAATCTTAAAGACCCCCCACTTAACCCTAAG-----

>JX948781_pcv2d

ATGACGTATCCAAGGAGGCGTTTCCGCAGACGAAGACACCGCCCCCGCAGCCATCTTGGCCAGATCCTCCGCCGCCGC
CCCTGGCTCGTCCACCCCCGCCACCGTTACCGCTGGAGAAGGAAAAATGGCATCTTCAACACCCGCCTCTCCCGCACCA
TCGGTTATACTGTCAAGAAAACCACAGTCAGAACGCCCTCCTGGAATGTGGACATGATGAGATTTAATATTAATGATT
TTCTTCCCCCAGGAGGGGGCTCAAACCCCCTCACTGTGCCCTTTGAATACTACAGAATAAGGAAGGTTAAGGTTGAAT
TCTGGCCCTGCTCCCCAATCACCCAGGGTGGCAGGGGAGTGGGCTCCACTGCTGTTATTCTAGATGATAACTTTGTAA
CAAAGGCCAATGCCCTAACCTATGACCCCTATGTAAACTACTCCTCCCGCCATACCATAACCCAGCCCTTCTCCTACCAC
TCCCGGTACTTTACCCCGAAACCTGTCCTTGATAGGACAATCGATTACTTCCAACCCAATAACAAAAGAAATCAACTCT
GGCTGAGACTACAAACTACTGGAAATGTAGACCATGTAGGCCTCGGCACTGCGTTCGAAAACAGTATATACGACCAG
GACTACAATATCCGTATAACCATGTATGTACAATTCAGAGAATTTAATCTTAAAGACCCCCCACTTAACCCTAAG-----

>HQ395050_pcv2d

ATGACGTATCCGAGGAGGCGTTTCCGCAGACGAAGACACCGCCCCCGCAGCCATCTTGGCCAGATCCTCCGCCGCCG
CCCCTGGCTCGTCCACCCCCGCCACCGTTACCGCTGGAGAAGGAAAAATGGCATCTTCAACACCCGCCTCTCCCGCACC
ATCGGTTATACTGTCAAGAAAACCACAGTCAGAACGCCCTCCTGGAATGTGGACATGATGAGATTTAATATTAATGAT
TTTCTTCCCCCAGGAGGGGGCTCAAACCCCCTCACTGTGCCCTTTGAATACTACAGAATAAGGAAGGTTAAGGTTGAA
TTCTGGCCCTGCTCCCCAATCACCCAGGGTGACAGGGGAGTGGGCTCCACTGCTGTTATTCTAGATGATAACTTTGTAA
CAAAGGCCAATGCCCTAACCTATGACCCCTATGTAAACTACTCCTCCCGCCATACCATAACCCAGCCCTTCTCCTACCAC
TCCCGGTACTTTACCCCGAAACCTGTCCTTGATAGGACAATCGATTACTTCCAACCCAATAACAAAAGAAATCAACTCT
GGCTGAGACTACAAACTACTGGAAATGTAGACCATGTAGGCCTCGGCACTGCGTTCGAAAACAGTATATACGACCAG
GACTACAATATCCGTATAACCATGTATGTACAATTCAGAGAATTTAATCTTAAAGACCCCCCACTTAACCCTAAG-----

>HQ113119_pcv2d

ATGACGTATCCAAGGAGGCGTTTCCGCAGACGAAGACACCGCCCCCGCAGCCATCTTGGCCAGATCCTCCGCCGCCGC
CCCTGGCTCGTCCACCCCCGCCACCGTTACCGCTGGAGAAGGAAAAATGGCATCTTCAACACCCGCCTCTCCCGCACCA
TCGGTTATACTGTCAAGAAAACCACAGTCAGAACGCCCTCCTGGAATGTGGACATGATGAGATTTAATATTAATGATT
TTCTTCCCCCAGGAGGGGGCTCAAACCCCCTCACTGTGCCCTTTGAATACTACAGAATAAGGAAGATTAAGGTTGAAT
TCTGGCCCTGCTCCCCAATCACCCAGGGTGACAGGGGAGTGGGCTCCACTGCTGTTATTCTAGATGATAACTTTGTAA
CAAAGGCCAATGCCCTAACCTATGACCCCTATGTAAACTACTCCTCCCGCCATACCATAACCCAGCCCTTCTCCTACCAC
TCCCGGTACTTTACCCCGAAACCTGTCCTTGATAGGACAATCGATTACTTCCAACCCAATAACAAAAGAAATCAACTCT
GGCTGAGACTACAAACTACTGGAAATGTAGACCATGTAGGCCTCGGCACTGCGTTCGAAAACAGTATATACGACCAG
GACTACAATATCCGTATAACCATGTATGTACAATTCAGAGAATTTAATCTTAAAGACCCCCCACTTAACCCTAAG-----

>HM142899_pcv2d

ATGACGTATCCAAGGAGGCGTTTCCGCAGACGAAGACACCGCCCCCGCAGCCATCTTGGCCAGATCCTCCGCCGCCGC
CCCTGGCTCGTCCACCCCCGCCACCGTTACCGCTGGAGAAGGAAAAATGGCATCTTCAACACCCGCCTCTCCCGCACCA
TCGGTTATACTGTCAAGAAAACCACAGTCAGAACGCCCTCCTGGAATGTGGACATGATGAGATTTAATATTAATGATT
TTCTTCCCCCAGGAGGGGGCTCAAACCCCCTCACTGTGCCCTTTGAATACTACAGAATAAGGAAGGTTAAGGTTGAAT
TCTGGCCCTGCTCCCCAATCACCCAGGGTGACAGGGGAGTGGGCTCCACTGCTGTTATTCTAGATGATAACTTTGTAA
CAAAGGCCAATGCCCTAACCTATGACCCCTATGTAAACTACTCCTCCCGCCATACCATAACCCAGCCCTTCTCCTACCAC
TCCCGGTACTTTACCCCGAAACCTGTCCTTGATAGGACAATCGATTACTTCCAACCCAATAACAAAAGAAATCAACTCT
GGCTGAGACTACAAACTACTGGAAATATAGACCATGTAGGCCTCGGCACTGCGTTCGAAAACAGTATATACGACCAG
GACTACAATATCCGTATAACCATGTATGTACAATTCAGAGAATTTAATCTTAAAGACCCCCCACTTAACCCTAAG-----

>HQ395029_pcv2d

ATGACGTATCCAAGGAGGCGTTTCCGCAGACGAAGACACCGCCCCCGCAGCCATCTTGGCCAGATCCTCCGCCGCCGC
CCCTGGCTCGTCCACCCCCGCCACCGTTACCGCTGGAGAAGGAAAAATGGCATCTTCAACACCCGCCTCTCCCGCACCA
TCGGTTATACTGTCAAGAAAACCACAGTCAGAACGCCCTCCTGGAATGTGGACATGATGAGATTTAATATTAATGATT
TTCTTCCCCCAGGAGGGGGCTCAAACCCCCTCACTGTGCCCTTTGAATACTACAGAATAAGGAAGGTTAAGGTTGAAT
TCTGGCCCTGCTCCCCAATCACCCAGGGTGACAGGGGAGTGGGCTCCACTGCTGTTATTCTAGATGATAACTTTGTAA
CAAAGGCCAATGCCCTAACCTATGACCCCTATGTAAACTACTCCTCCCGCCATACCATAACCCAGCCCTTCTCCTACCAC
TCCCGGTACTTTACCCCGAAACCTGTCCTTGATAGGACAATCGATTACTTCCAACCCAATAACAAAAGAAATCAACTCT
GGCTGAGACTACAAACTACTGGAAATGTAGACCATGTAGGCCTCGGCACTGCGTTCGAAAACAGTATATACGACCAG
GACTACAATATCCGTATAACCATGTATGTACAATTCAGAGAATTTAACCTTAAAGACCCCCCACTTAACCCTAAG-----

>HQ395048_pcv2d

ATGACGTATCCAAGGAGGCGTTTCCGCAGACGAAGACACCGCCCCCGCAGCCATCTTGGCCAGATCCTCCGCCGCCGC
CCCTGGCTCGTCCACCCCCGCCACCGTTACCGCTGGAGAAGGAAAAATGGCATCTTCAACACCCGCCTCTCCCGCACCA
TCGGTTATACTGTCAAGAAAACCACAGTCAGAACGCCCTCCTGGAATGTGGACATGATGAGATTTAATATTAATGATT
TTCTTCCCCCAGGAGGGGGCTCAAACCCCCTCACTGTGCCCTTTGAATACTACAGAATAAGGAAGGTTAAGGTTGAAT
TCTGGCCCTGCTCCCCAATCACCCAGGGTGACAGGGGAGTGGGCTCCACTGCTGTTATTCTAGATGATAACTTTGTAA
CAAAGGCCAATGCCCTAACCTATGACCCCTATGTAAACTACTCCTCCCGCCATACCATAACCCAGCCCTTCTCCTACCAC
TCCCGGTACTTTACCCCGAAACCTGTCCTTGATAGGACAATCGATTACTTCCAACCCAATAACAAAAGAAATCAACTCT
GGCTGAGACTACAAACTACTGGAAATGTAGACCATGTAGGCCTCGGCACTGCGTTCGAAAACAGTATATACGACCAG
GACTACAATATCCGTATAACCATGTATGTACAATTCAGAGAATTTAACCTTAAAGACCCCCCACTTAACCCTAAG-----

>JX982222_pcv2d

ATGACGTATCCAAGGAGGCGTTTCCGCAGACGAAGACACCGCCCCCGCAGCCATCTTGGCCAGATCCTCCGCCGCCGC
CCCTGGCTCGTCCACCCCCGCCACCGTTACCGCTGGAGAAGGAAAAATGGCATCTTCAACACCCGCCTCTCCCGCACCA
TCGGTTATACTGTCAAGAAAACCACAGTCAGAACGCCCTCCTGGAATGTGGACATGATGAGATTTAATATTAATGATT
TTCTTCCCCCAGGAGGGGGATCAAACCCCCTCACTGTGCCCTTTGAATACTACAGAATAAGGAAGGTTAAGGTTGAAT
TCTGGCCCTGCTCCCCAATCACCCAGGGTGACAGGGGAGTGGGCTCCACTGCTGTTATTCTAGATGATAACTTTGTAA
CAAAGGCCAATGCCCTAACCTATGACCCCTATGTAAACTACTCCTCCCGCCATACCATAACCCAGCCCTTCTCCTACCAC
TCCCGGTACTTTACCCCGAAACCTGTCCTTGATAGGACAATCGATTACTTCCAACCCAATAACAAAAGAAATCAACTCT
GGCTGAGACTACAAACTACTGGAAATGTAGACCATGTAGGCCTCGGCACTGCGTTCGAAAACAGTATATACGACCAG
GACTACAATATCCGTATAACCATGTATGTACAATTCAGAGAATTTAACCTTAAAGACCCCCCACTTAACCCTAAG-----

>JF683394_pcv2d

ATGACGTATCCAAGGAGGCGTTTCCGCAGACGAAGACACCGCCCCCGCAGCCATCTTGGCCAGATCCTCCGCCGCCGC
CCCTGGCTCGTCCACCCCCGCCACCGTTACCGCTGGAGAAGGAAAAATGGCATCTTCAACACCCGCCTCTCCCGCACCA
TCGGTTATACTGTCAAGAAAACCACAGTCAGAACGCCCTCCTGGAATGTGGACATGATGAGATTTAATATTAATGATT
TTCTTCCCCCAGGAGGGGGCTCAAACCCCCTCACTGTGCCCTTTGAATACTACAGAATAAGGAAGGTTAAGGTTGAAT
TCTGGCCCTGCTCCCCAATCACCCAGGGTGACAGGGGAGTGGGCTCCACTGCTGTTATTCTAGATGATAACTTTGTAA
CAAAGGCCAATGCCCTAACCTATGACCCCTATGTAAACTACTCCTCCCGCCATACCATAACCCAGCCCTTCTCCTACCAC
TCCCGGTACTTTACCCCGAAACCTGTCCTTGATAGGACAATCGATTACTTCCAACCCAATAACAAAAGAAATCAACTCT
GGCTGAGACTACAAACTACTGGAAATGTAGACCATGTAGGCCTCGGCACTGCGTTCGAAAACAGTATATACGACCAG
GACTACAATATCCGTATAACCATGTATGTACAATTCAGAGAATTTAATCTTAAAGACCACCCACTTAACCCTAAG-----

>EF675230_pcv2d

ATGACGTATCCAAGGAGGCGTTTCCGCAGACGAAGACACCGCCCCCGCAGCCATCTTGGCCAGATCCTCCGCCGCCGC
CCCTGGCTCGTCCACCCCCGCCACCGTTACCGCTGGAGAAGGAAAAATGGCATCTTCAACACCCGCCTCTCCCGCACCA
TCGGTTATACTGTCAAGAAAACGACAGTCAGAACGCCCTCCTGGAATGTGGACATGATGAGATTTAATATTAATGATT
TTCTTCCCCCAGGAGGGGGCTCAAACCCCCTCACTGTGCCCTTTGAATACTACAGAATAAGGAAGGTTAAGGTTGAAT
TCTGGCCCTGCTCCCCAATCACCCAGGGTGACAGGGGAGTGGGCTCCACTGCTGTTATTCTAGATGATAACTTTGTAA
CAAAGGCCAATGCCCTAACCTATGACCCCTATGTAAACTACTCCTCCCGCCATACCATAACCCAGCCCTTCTCCTACCAC
TCCCGGTACTTTACCCCGAAACCTGTCCTTGATAGGACAATCGATTACTTCCAACCCAATAACAAAAGAAATCAACTCT
GGCTGAGACTACAAACTACTGGAAATGTAGACCATGTAGGCCTCGGCACTGCGTTCGAAAACAGTATATACGACCAG
GACTACAATATCCGTATAACCATGTATGTACAATTCAGAGAATTTAATCTTAAAGACCCCCCACTTAACCCTAAG-----

>FJ426398_pcv2d

ATGACGTATCCAAGGAGGCGTTTCCGCAGACGAAGACACCGCCCCCGCAGCCATCTTGGCCAGATCCTCCGCCGCCGC
CCCTGGCTCGTCCACCCCCGCCACCGTTACCGCTGGAGAAGGAAAAATGGCATCTTCAACACCCGCCTCTCCCGCACCA
TCGGTTATACTGTCAAGAAAACGACAGTCAGAACGCCCTCCTGGAATGTGGACATGATGAGATTTAATATTAATGATT
TTCTTCCCCCAGGAGGGGGCTCAAACCCCCTCACTGTGCCCTTTGAATACTACAGAATAAGGAAGGTTAAGGTTGAAT
TCTGGCCCTGCTCCCCAATCACCCAGGGTGACAGGGGAGTGGGCTCCACTGCTGTTATTCTAGATGATAACTTTGTAA
CAAAGGCCAATGCCCTAACCTATGACCCCTATGTAAACTACTCCTCCCGCCATACCATAACCCAGCCCTTCTCCTACCAC
TCCCGGTACTTTACCCCGAAACCTGTCCTTGATAGGACAATCGATTACTTCCAACCCAATAACAAAAGAAATCAACTCT
GGCTGAGACTACAAACTACTGGAAATGTAGACCATGTAGGCCTCGGCACTGCGTTCGAAAACAGTATATACGACCAG
GACTACAATATCCGTATAACCATGTATGTACAATTCAGAGAATTTAATCTTAAAGACCCCCCACTTAACCCTAAG-----

>HQ395025_pcv2d

ATGACGTATCCAAGGAGGCGTTTCCGCAGACGAAGACACCGCCCCCGCAGCCATCTTGGCCAGATCCTCCGCCGCCGC
CCCTGGCTCGTCCACCCCCGCCACCGTTACCGCTGGAGAAGGAAAAATGGCATCTTCAACACCCGCCTCTCCCGCACCA
TCGGTTATACTGTCAAGAAAACCACAGTCAGAACGCCCTCCTGGAATGTGGACATGATGAGATTTAATATTAATGATT
TTCTTCCCCCAGGAGGGGGCTCAAACCCCCTCACTGTGCCCTTTGAATACTACAGAATAAGGAAGGTTAAGGTTGAAT
TCTGGCCCTGCTCCCCAATCACCCAGGGTGACAGGGGAGTGGGCTCCACTGCTGTTATTCTAGATGATAACTTTGTAA
CAAAGGCCAATGCCCTAACCTATGACCCCTATGTAAACTACTCCTCCCGCCATACCATAACCCAGCCCTTCTCCTACCAC
TCCCGGTACTTTACCCCGAAACCTGTCCTTGATAGGACAATCGATTACTTCCAACCCAATAACAAAAGAAATCAACTCT
GGCTGAGACTACAAACTACTGGAAACGTAGACCATGTAGGCCTCGGCACTGCGTTCGAAAACAGTATATACGACCAG
GACTACAATATCCGTATAACCATGTATGTACAATTCAGAGAATTTAATCTTAAAGACCCCCCACTTAACCCTAAG-----

>GQ359010_pcv2d

ATGACGTATCCAAGGAGGCGTTTCCGCAGACGAAGACACCGCCCCCGCAGCCATCTTGGCCAGATCCTCCGCCGCCGC
CCCTGGCTCGTCCACCCCCGCCACCGTTACCGCTGGAGAAGGAAAAATGGCATCTTCAACACCCGCCTCTCCCGCACCA
TCGGTTATACTGTCAAGAAAACCACAGTCAGAACGCCCTCCTGGAATGTGGACATGATGAGATTTAATATTAATGATT
TTCTTCCCCCAGGAGGGGGCTCAAATCCCCTCACTGTGCCCTTTGAATACTACAGAATAAGGAAGGTTAAGGTTGAAT
TCTGGCCCTGCTCCCCAATCACCCAGGGTGACAGGGGAGTGGGCTCCACTGCTGTTATTCTAGATGATAACTTTGTAA
CAAAGGCCAATGCCCTAACCTATGACCCCTATGTAAACTACTCCTCCCGCCATACCATAACCCAGCCCTTCTCCTACCAC
TCCCGGTACTTTACCCCGAAACCTGTCCTTGATAGGACAATCGATTACTTCCAACCCAATAACAAAAGAAATCAACTCT
GGCTGAGACTACAAACTACTGGAAATGTAGACCATGTAGGCCTCGGCACTGCGTTCGAAAACAGTATATACGACCAG
GACTACAATATCCGTATAACCATGTATGTACAATTCAGAGAATTTAATCTTAAAGACCCCCCACTTAACCCTAAG-----

>HM776449_pcv2d

ATGACGTATCCAAGGAGGCGTTTCCGCAGACGAAGACACCGCCCCCGCAGCCATCTTGGCCAGATCCTCCGCCGCCGC
CCCTGGCTCGTCCACCCCCGCCACCGTTACCGCTGGAGAAGGAAAAATGGCATCTTCAACACCCGCCTCTCCCGCACCA
TCGGTTACACTGTCAAGAAAACCACAGTCAGAACGCCCTCCTGGAATGTGGACATGATGAGATTTAATATTAATGATT
TTCTTCCCCCAGGAGGGGGCTCAAACCCCCTCACTGTGCCCTTTGAATACTACAGAATAAGGAAGGTTAAGGTTGAAT
TCTGGCCCTGCTCCCCAATCACCCAGGGTGACAGGGGAGTGGGCTCCACTGCTGTTATTCTAGATGATAACTTTGTAA
CAAAGGCCAATGCCCTAACCTATGACCCCTATGTAAACTACTCCTCCCGCCATACCATAACCCAGCCCTTCTCCTACCAC
TCCCGGTACTTTACCCCGAAACCTGTCCTTGATAGGACAATCGATTACTTCCAACCCAATAACAAAAGAAATCAACTCT
GGCTGAGACTACAAACTACTGGAAATGTAGACCATGTAGGCCTCGGCACTGCGTTCGAAAACAGTATATACGACCAG
GACTACAATATCCGTATAACCATGTATGTACAATTCAGAGAATTTAATCTTAAAGACCCCCCACTTAACCCTAAG-----

>GU083583_pcv2d

ATGACGTATCCAAGGAGGCGTTTCCGCAGACGAAGACACCGCCCCCGCAGCCATCTTGGCCAGATCCTCCGCCGCCGC
CCCTGGCTCGTCCACCCCCGCCACCGTTACCGCTGGAGAAGGAAAAATGGCATCTTCAACACCCGCCTCTCCCGCACCA
TCGGTTATACTGTCAAGAAAACCACAGTCAGAACGCCCTCCTGGAATGTGGACATGATGAGATTTAATATTAATGATT
TTCTTCCCCCAGGAGGGGGCTCAAACCCCCTCACTGTGCCCTTTGAATACTACAGAATAAGGAAGGTTAAGGTTGAAT
TCTGGCCCTGCTCCCCAATCACCCAGGGTGACAGGGGAGTGGGCTCCACTGCTGTTATTCTAGATGATAACTTTGTAA
CAAAGGCCAATGCCCTAACCTATGACCCCTATGTAAACTACTCCTCCCGCCATACCATAACCCAGCCCTTCTCCTACCAC
TCCCGGTACTTTACCCCGAAACCTGTCCTTGATAGGACAATCGATTACTTCCAACCCAATAACAAAAGAAATCAACTCT
GGCTGAGACTACAAACTACTGGAAATGTAGACCATGTAGGTCTCGGCACTGCGTTCGAAAACAGTATATACGACCAG
GACTACAATATCCGTATAACCATGTATGTACAATTCAGAGAATTTAATCTTAAAGACCCCCCACTTAACCCTAAG-----

>HM776453_pcv2d

ATGACGTATCCAAGGAGGCGTTTCCGCAGACGAAGACACCGCCCCCGCAGCCATCTTGGCCAGATCCTCCGCCGCCGC
CCCTGGCTCGTCCACCCCCGCCACCGTTACCGCTGGAGAAGGAAAAATGGCATCTTCAACACCCGCCTCTCCCGCACCA
TCGGTTATACTGTCAAGAAAACCACAGTCAGAACGCCCTCCTGGAATGTGGACATGATGAGATTTAATATTAATGATT
TTCTTCCCCCAGGAGGGGGCTCAAACCCCCTCACTGTGCCCTTTGAATACTACAGAATAAGGAAGGTTAAGGTTGAAT
TCTGGCCCTGCTCCCCAATCACCCAGGGTGACAGGGGAGTGGGCTCCACTGCTGTTATTCTAGATGATAACTTTGTAA
CAAAGGCCAATGCCCTAACCTATGACCCCTATGTAAACTACTCCTCCCGCCATACCATAACCCAGCCCTTCTCCTACCAC
TCCCGGTACTTTACCCCGAAACCTGTCCTTGATAGGACAATCGATTACTTCCAACCCAATAACAAAAGAAATCAACTCT
GGCTGAGACTACAAACTACTGGAAATGTAGACCATGTAGGCCTCGGCACTGCCTTCGAAAACAGTATATACGACCAG
GACTACAATATCCGTATAACCATGTATGTACAATTCAGAGAATTTAATCTTAAAGACCCCCCACTTAACCCTAAG-----

>KC473166_pcv2d

ATGACGTATCCAAGGAGGCGTTTCCGCAGACGAAGACACCGCCCCCGCAGCCATCTTGGCCAGATCCTCCGCCGCCGC
CCCTGGCTCGTCCACCCCCGCCACCGTTACCGCTGGAGAAGGAAAAATGGCATCTTCAACACCCGCCTCTCCCGCACCA
TCGGTTATACTGTCAAGAAAACCACAGTCAGAACGCCCTCCTGGAATGTGGACATGATGAGATTTAATATTAATGATT
TTCTTCCCCCAGGAGGGGGCTCAAACCCCCTCACTGTGCCCTTTGAATACTACAGAATAAGGAAGGTTAAGGTTGAAT
TCTGGCCCTGCTCCCCAATCACCCAGGGTGACAGGGGAGTGGGCTCCACTGCTGTTATTCTAGATGATAACTTTGTAA
CAAAGGCCAATGCCCTAACCTATGACCCCTATGTAAACTACTCCTCCCGCCATACCATAACCCAGCCCTTCTCCTACCAC
TCCCGGTACTTTACCCCGAAACCTGTCCTTGATAGGACAATCGATTACTTCCAACCCAATAACAAAAGAAATCAACTCT
GGCTGAGACTACAAACTACTGGAAATGTAGACCATGTAGGCCTCGGCACTGCTTTCGAAAACAGTATATACGACCAG
GACTACAATATCCGTATAACCATGTATGTACAATTCAGAGAATTTAATCTTAAAGACCCCCCACTTAACCCTAAG-----

>HQ395041_pcv2d

ATGACGTATCCAAGGAGGCGTTTCCGCAGACGAAGACACCGCCCCCGCAGCCATCTTGGCCAGATCCTCCGCCGCCGC
CCCTGGCTCGTCCACCCCCGCCACCGTTACCGCTGGAGAAGGAAAAATGGCATCTTCAACACCCGCCTCTCCCGCACCA
TCGGTTATACTGTCAAGAAAACCACAGTCAGAACGCCCTCCTGGAATGTGGACATGATGAGATTTAATATTAATGATT
TTCTTCCCCCAGGAGGGGGCTCAAACCCCCTCACTGTGCCCTTTGAATACTACAGAATAAGGAAGGTTAAGGTTGAAT
TCTGGCCCTGCTCCCCAATCACCCAGGGTGACAGGGGAGTGGGCTCCACTGCTGTTATTCTAGATGATAACTTTGTAA
CAAAGGCCAATGCCCTAACCTATGACCCCTATGTAAACTACTCCTCCCGCCATACCATAACCCAGCCCTTCTCCTACCAC
TCCCGGTATTTTACCCCGAAACCTGTCCTTGATAGGACAATCGATTACTTCCAACCCAATAACAAAAGAAATCAACTCT
GGCTGAGACTACAAACTACTGGAAATGTAGACCATGTAGGCCTCGGCACTGCGTTCGAAAACAGTATATACGACCAG
GACTACAATATCCGTATAACCATGTATGTACAATTCAGAGAATTTAATCTTAAAGACCCCCCACTTAACCCTAAG-----

>KC527542_pcv2d

ATGACGTATCCAAGGAGGCGTTTCCGCAGACGAAGACACCGCCCCCGCAGCCATCTTGGCCAGATCCTCCGCCGCCGC
CCCTGGCTCGTCCACCCCCGCCACCGTTACCGCTGGAGAAGGAAAAATGGCATCTTCAACACCCGCCTCTCCCGCACCA
TCGGTTATACTGTCAAGAAAACCACAGTCAGAACGCCCTCCTGGAATGTGGACATGATGAGATTTAATATTAATGATT
TTCTTCCCCCAGGAGGGGGCTCAAACCCCCTCACTGTGCCCTTTGAATACTACAGAATAAGGAAGGTTAAGGTTGAAT
TCTGGCCCTGCTCCCCAATCACCCAGGGTGACAGGGGAGTGGGCTCCACTGCTGTTATTCTAGATGATAACTTTGTAA
CAAAGGCCAATGCCCTAACGTATGACCCCTATGTAAACTACTCCTCCCGCCATACCATAACCCAGCCCTTCTCCTACCAC
TCCCGGTACTTTACCCCGAAACCTGTCCTTGATAGGACAATCGATTACTTCCAACCCAATAACAAAAGAAATCAACTCT
GGCTGAGACTACAAACTACTGGAAATGTAGACCATGTAGGCCTCGGCACTGCGTTCGAAAACAGTATATACGACCAG
GACTACAATATCCGTATAACCATGTATGTACAATTCAGAGAATTTAATCTTAAAGACCCCCCACTTAACCCTAAG-----

>HM535641_pcv2d

ATGACGTATCCAAGGAGGCGTTTCCGCAGACGAAGACACCGCCCACGCAGCCATCTTGGCCAGATCCTCCGCCGCCG
CCCCTGGCTCGTCCACCCCCGCCACCGTTACCGCTGGAGAAGGAAAAATGGCATCTTCAACACCCGCCTCTCCCGCACC
ATCGGTTATACTGTCAAGAAAACCACAGTCAGAACGCCCTCCTGGAATGTGGACATGATGAGATTTAATATTAATGAT
TTTCTTCCCCCAGGAGGGGGCTCAAACCCCCTCACTGTGCCCTTTGAATACTACAGAATAAGGAAGGTTAAGGTTGAA
TTCTGGCCCTGCTCCCCAATCACCCAGGGTGACAGGGGAGTGGGCTCCACTGCTGTTATTCTAGATGATAACTTTGTCA
CAAAGGCCAATGCCCTAACCTATGACCCCTATGTAAACTACTCCTCCCGCCATACCATAACCCAGCCCTTCTCCTACCAC
TCCCGGTACTTTACCCCGAAACCTGTCCTTGATAGGACAATCGATTACTTCCAACCCAATAACAAAAGAAATCAACTCT
GGCTGAGACTACAAACTACTGGAAATGTAGACCATGTAGGCCTCGGCACTGCGTTCGAAAACAGTATATACGACCAG
GACTACAATATCCGTATAACCATGTATGTACAATTCAGAGAATTTAATCTTAAAGACCCCCCACTTAACCCT--------

>HM142895_pcv2d

ATGACGTATCCAAGGAGGCGTTTCCGCAGACGAAGACACCGCCCACGCAGCCATCTTGGCCAGATCCTCCGCCGCCG
CCCCTGGCTCGTCCACCCCCGCCACCGTTACCGCTGGAGAAGGAAAAATGGCATCTTCAACACCCGCCTCTCCCGCACC
ATCGGCTATACTGTCAAGAAAACCACAGTCAGAACGCCCTCCTGGAATGTGGACATGATGAGATTTAATATTAATGAT
TTTCTTCCCCCAGGAGGGGGCTCAAACCCCCTCACTGTGCCCTTTGAATACTACAGAATAAGGAAGGTTAAGGTTGAA
TTCTGGCCCTGCTCCCCAATCACCCAGGGTGACAGGGGAGTGGGCTCCACTGCTGTTATTCTAGATGATAACTTTGTAA
CAAAGGCCAATGCCCTAACATATGACCCCTATGTAAACTACTCCTCCCGCCATACCATAACCCAGCCCTTCTCCTACCAC
TCCCGGTACTTTACCCCGAAACCTGTCCTTGATAGGACAATCGATTACTTCCAACCCAATAACAAAAGAAATCAACTCT
GGCTGAGACTACAAACTACTGGAAATGTAGACCATGTAGGCCTCGGCACTGCGTTCGAAAACAGTATATACGACCAG
GACTACAATATCCGTATAACCATGTATGTACAATTCAGAGAATTTAATCTTAAAGACCCCCCACTTAACCCTAAG-----

>GU001710_pcv2d

ATGACGTATCCAAGGAGGCGTTTCCGCAGACGAAGACACCGCCCCCGCAGCCATCTTGGCCAGATCCTCCGCCGCCGC
CCCTGGCTCGTCCACCCCCGCCACCGTTACCGCTGGAGAAGGAAAAATGGCATCTTCAACACCCGCCTCTCCCGCACCA
TCGGTTATACTGTCAAGAAAACCACAGTCAGAACGCCCTCCTGGAATGTGGACATGATGAGATTTAATATTAATGATT
TTCTTCCCCCAGGAGGGGGCTCAAACCCCCTCACTGTGCCCTTTGAATACTACAGAATAAGGAAGGTTAAGGTTGAAT
TCTGGCCCTGCTCCCCAATCACCCAGGGTGACAGGGGAGTGGGCTCCACTGCTGTTATTCTAGATGATAACTTTGTAA
CAAAGGCCAATGCCCTAACCTATGACCCCTATGTAAACTACTCCTCCCGCCATACCATAACCCAGCCCTTCTCCTACCAC
TCCCGGTACTTTACCCCGAAACCTGTCCTTGATAGGACAATCGATTACTTCCAACCCAATAACAAAAGAAACCAACTCT
GGCTGAGACTACAAACTACTGGAAATGTAGACCATGTAGGCCTCGGCACTGCGTTCGAAAACAGTATATACGACCAG
GACTACAATATCCGTATAACCATGTATGTACAATTCAGAGAATTTAATCTTAAAGACCCCCCACTTAACCCTAAG-----

>JX948783_pcv2d

ATGACGTATCCAAGGAGGCGTTTCCGCAGACGAAGACACCGCCCCCGCAGCCATCTTGGCCAGATCCTCCGCCGCCGC
CCCTGGCTCGTCCACCCCCGCCACCGTTACCGCTGGAGAAGGAAAAATGGCATCTTCAACACCCGCCTCTCCCGCACCA
TCGGTTATACTGTCAAGAAAACCACAGTCAGAACGCCCTCCTGGAATGTGGACATGATGAGATTTAATATTAATGATT
TTCTTCCCCCAGGAGGGGGCTCAAACCCCCTCACTGTGCCCTTTGAATACTACAGAATAAGGAAGGTTAAGGTTGAAT
TCTGGCCCTGCTCCCCAATCACCCAGGGTGACAGGGGAGTGGGCTCCACTGCTGTTATTCTAGATGATAACTTTGTAA
CAAAGGCCAATGCCCTAACCTATGACCCCTATGTAAACTACTCCTCCCGCCATACCATAACCCAGCCCTTCTCCTACCAC
TCCCGGTACTTTACCCCGAAACCTGTCCTTGATAGGACAATCGATTACTTCCAACCCAATAACAAAAGAAATCAACTCT
GGCTGAGACTACAAACTACTGGAAATGTAGACCATGTAGGCCTCGGCACTGCGTTCGAAAACAGTATATACGACCAG
GACTACAATATCCGTATAACCATGTATGTACAATTCAGAGAATTTAATCTTAAAGACCCCCCACTTAACCCTAAG-----

>JX948777_pcv2d

ATGACGTATCCAAGGAGGCGTTTCCGCAGACGAAGACACCGCCCCCGCAGCCATCTTGGCCAGATCCTCCGCCGCCGC
CCCTGGCTCGTCCACCCCCGCCACCGTTACCGCTGGAGAAGGAAAAATGGCATCTTCAACACCCGCCTCTCCCGCACCA
TCGGTTATACTGTCAAGAAAACCACAGTCAGAACGCCCTCCTGGAATGTGGACATGATGAGATTTAATATTAATGATT
TTCTTCCCCCAGGAGGGGGCTCAAACCCCCTCACTGTGCCCTTTGAATACTACAGAATAAGGAAGGTTAAGGTTGAAT
TCTGGCCCTGCTCCCCAATCACCCAGGGTGACAGGGGAGTGGGCTCCACTGCTGTTATTCTAGATGATAACTTTGTAA
CAAAGGCCAATGCCCTAACCTATGACCCCTATGTAAACTACTCCTCCCGCCATACCATAACCCAGCCCTTCTCCTACCAC
TCCCGGTACTTTACCCCGAAACCTGTCCTTGATAGGACAATCGATTACTTCCAACCCAATAACAAAAGAAATCAACTCT
GGCTGAGACTACAAACTACTGGAAATGTAGACCATGTAGGCCTCGGCACTGCGTTCGAAAACAGTATATACGACCAG
GACTACAATATCCGTATAACCATGTATGTACAATTCAGAGAATTTAATCTTAAAGACCCCCCACTTAACCCTAAG-----

>JX948769_pcv2d

ATGACGTATCCAAGGAGGCGTTTCCGCAGACGAAGACACCGCCCCCGCAGCCATCTTGGCCAGATCCTCCGCCGCCGC
CCCTGGCTCGTCCACCCCCGCCACCGTTACCGCTGGAGAAGGAAAAATGGCATCTTCAACACCCGCCTCTCCCGCACCA
TCGGTTATACTGTCAAGAAAACCACAGTCAGAACGCCCTCCTGGAATGTGGACATGATGAGATTTAATATTAATGATT
TTCTTCCCCCAGGAGGGGGCTCAAACCCCCTCACTGTGCCCTTTGAATACTACAGAATAAGGAAGGTTAAGGTTGAAT
TCTGGCCCTGCTCCCCAATCACCCAGGGTGACAGGGGAGTGGGCTCCACTGCTGTTATTCTAGATGATAACTTTGTAA
CAAAGGCCAATGCCCTAACCTATGACCCCTATGTAAACTACTCCTCCCGCCATACCATAACCCAGCCCTTCTCCTACCAC
TCCCGGTACTTTACCCCGAAACCTGTCCTTGATAGGACAATCGATTACTTCCAACCCAATAACAAAAGAAATCAACTCT
GGCTGAGACTACAAACTACTGGAAATGTAGACCATGTAGGCCTCGGCACTGCGTTCGAAAACAGTATATACGACCAG
GACTACAATATCCGTATAACCATGTATGTACAATTCAGAGAATTTAATCTTAAAGACCCCCCACTTAACCCTAAG-----

>KC860786_pcv2d

ATGACGTATCCAAGGAGGCGTTTCCGCAGACGAAGACACCGCCCCCGCAGCCATCTTGGCCAGATCCTCCGCCGCCGC
CCCTGGCTCGTCCACCCCCGCCACCGTTACCGCTGGAGAAGGAAAAATGGCATCTTCAACACCCGCCTCTCCCGCACCA
TCGGTTATACTGTCAAGAAAACCACAGTCAGAACGCCCTCCTGGAATGTGGACATGATGAGATTTAATATTAATGATT
TTCTTCCCCCAGGAGGGGGCTCAAACCCCCTCACTGTGCCCTTTGAATACTACAGAATAAGGAAGGTTAAGGTTGAAT
TCTGGCCCTGCTCCCCAATCACCCAGGGTGACAGGGGAGTGGGCTCCACTGCTGTTATTCTAGATGATAACTTTGTAA
CAAAGGCCAATGCCCTAACCTATGACCCCTATGTAAACTACTCCTCCCGCCATACCATAACCCAGCCCTTCTCCTACCAC
TCCCGGTACTTTACCCCGAAACCTGTCCTTGATAGGACAATCGATTACTTCCAACCCAATAACAAAAGAAATCAACTCT
GGCTGAGACTACAAACTACTGGAAATGTAGACCATGTAGGCCTCGGCACTGCGTTCGAAAACAGTATATACGACCAG
GACTACAATATCCGTATAACCATGTATGTACAATTCAGAGAATTTAATCTTAAAGACCCCCCACTTAACCCTAAG-----

>GU574204_pcv2d

ATGACGTATCCAAGGAGGCGTTTCCGCAGACGAAGACACCGCCCCCGCAGCCATCTTGGCCAGATCCTCCGCCGCCGC
CCCTGGCTCGTCCACCCCCGCCACCGTTACCGCTGGAGAAGGAAAAATGGCATCTTCAACACCCGCCTCTCCCGCACCA
TCGGTTATACTGTCAAGAAAACCACAGTCAGAACGCCCTCCTGGAATGTGGACATGATGAGATTTAATATTAATGATT
TTCTTCCCCCAGGAGGGGGCTCAAACCCCCTCACTGTGCCCTTTGAATACTACAGAATAAGGAAGGTTAAGGTTGAAT
TCTGGCCCTGCTCCCCAATCACCCAGGGTGACAGGGGAGTGGGCTCCACTGCTGTTATTCTAGATGATAACTTTGTAA
CAAAGGCCAATGCCCTAACCTATGACCCCTATGTAAACTACTCCTCCCGCCATACCATAACCCAGCCCTTCTCCTACCAC
TCCCGGTACTTTACCCCGAAACCTGTCCTTGATAGGACAATCGATTACTTCCAACCCAATAACAAAAGAAATCAACTCT
GGCTGAGACTACAAACTACTGGAAATGTAGACCATGTAGGCCTCGGCACTGCGTTCGAAAACAGTATATACGACCAG
GACTACAATATCCGTATAACCATGTATGTACAATTCAGAGAATTTAATCTTAAAGACCCCCCACTTAACCCTAAG-----

>GU938302_pcv2d

ATGACGTATCCAAGGAGGCGTTTCCGCAGACGAAGACACCGCCCCCGCAGCCATCTTGGCCAGATCCTCCGCCGCCGC
CCCTGGCTCGTCCACCCCCGCCACCGTTACCGCTGGAGAAGGAAAAATGGCATCTTCAACACCCGCCTCTCCCGCACCA
TCGGTTATACTGTCAAGAAAACCACAGTCAGAACGCCCTCCTGGAATGTGGACATGATGAGATTTAATATTAATGATT
TTCTTCCCCCAGGAGGGGGCTCAAACCCCCTCACTGTGCCCTTTGAATACTACAGAATAAGGAAGGTTAAGGTTGAAT
TCTGGCCCTGCTCCCCAATCACCCAGGGTGACAGGGGAGTGGGCTCCACTGCTGTTATTCTAGATGATAACTTTGTAA
CAAAGGCCAATGCCCTAACCTATGACCCCTATGTAAACTACTCCTCCCGCCATACCATAACCCAGCCCTTCTCCTACCAC
TCCCGGTACTTTACCCCGAAACCTGTCCTTGATAGGACAATCGATTACTTCCAACCCAATAACAAAAGAAATCAACTCT
GGCTGAGACTACAAACTACTGGAAATGTAGACCATGTAGGCCTCGGCACTGCGTTCGAAAACAGTATATACGACCAG
GACTACAATATCCGTATAACCATGTATGTACAATTCAGAGAATTTAATCTTAAAGACCCCCCACTTAACCCTAAG-----

>HM038017_pcv2d

ATGACGTATCCAAGGAGGCGTTTCCGCAGACGAAGACACCGCCCCCGCAGCCATCTTGGCCAGATCCTCCGCCGCCGC
CCCTGGCTCGTCCACCCCCGCCACCGTTACCGCTGGAGAAGGAAAAATGGCATCTTCAACACCCGCCTCTCCCGCACCA
TCGGTTATACTGTCAAGAAAACCACAGTCAGAACGCCCTCCTGGAATGTGGACATGATGAGATTTAATATTAATGATT
TTCTTCCCCCAGGAGGGGGCTCAAACCCCCTCACTGTGCCCTTTGAATACTACAGAATAAGGAAGGTTAAGGTTGAAT
TCTGGCCCTGCTCCCCAATCACCCAGGGTGACAGGGGAGTGGGCTCCACTGCTGTTATTCTAGATGATAACTTTGTAA
CAAAGGCCAATGCCCTAACCTATGACCCCTATGTAAACTACTCCTCCCGCCATACCATAACCCAGCCCTTCTCCTACCAC
TCCCGGTACTTTACCCCGAAACCTGTCCTTGATAGGACAATCGATTACTTCCAACCCAATAACAAAAGAAATCAACTCT
GGCTGAGACTACAAACTACTGGAAATGTAGACCATGTAGGCCTCGGCACTGCGTTCGAAAACAGTATATACGACCAG
GACTACAATATCCGTATAACCATGTATGTACAATTCAGAGAATTTAATCTTAAAGACCCCCCACTTAACCCTAAG-----

>HM038030_pcv2d

ATGACGTATCCAAGGAGGCGTTTCCGCAGACGAAGACACCGCCCCCGCAGCCATCTTGGCCAGATCCTCCGCCGCCGC
CCCTGGCTCGTCCACCCCCGCCACCGTTACCGCTGGAGAAGGAAAAATGGCATCTTCAACACCCGCCTCTCCCGCACCA
TCGGTTATACTGTCAAGAAAACCACAGTCAGAACGCCCTCCTGGAATGTGGACATGATGAGATTTAATATTAATGATT
TTCTTCCCCCAGGAGGGGGCTCAAACCCCCTCACTGTGCCCTTTGAATACTACAGAATAAGGAAGGTTAAGGTTGAAT
TCTGGCCCTGCTCCCCAATCACCCAGGGTGACAGGGGAGTGGGCTCCACTGCTGTTATTCTAGATGATAACTTTGTAA
CAAAGGCCAATGCCCTAACCTATGACCCCTATGTAAACTACTCCTCCCGCCATACCATAACCCAGCCCTTCTCCTACCAC
TCCCGGTACTTTACCCCGAAACCTGTCCTTGATAGGACAATCGATTACTTCCAACCCAATAACAAAAGAAATCAACTCT
GGCTGAGACTACAAACTACTGGAAATGTAGACCATGTAGGCCTCGGCACTGCGTTCGAAAACAGTATATACGACCAG
GACTACAATATCCGTATAACCATGTATGTACAATTCAGAGAATTTAATCTTAAAGACCCCCCACTTAACCCTAAG-----

>HM102350_pcv2d

ATGACGTATCCAAGGAGGCGTTTCCGCAGACGAAGACACCGCCCCCGCAGCCATCTTGGCCAGATCCTCCGCCGCCGC
CCCTGGCTCGTCCACCCCCGCCACCGTTACCGCTGGAGAAGGAAAAATGGCATCTTCAACACCCGCCTCTCCCGCACCA
TCGGTTATACTGTCAAGAAAACCACAGTCAGAACGCCCTCCTGGAATGTGGACATGATGAGATTTAATATTAATGATT
TTCTTCCCCCAGGAGGGGGCTCAAACCCCCTCACTGTGCCCTTTGAATACTACAGAATAAGGAAGGTTAAGGTTGAAT
TCTGGCCCTGCTCCCCAATCACCCAGGGTGACAGGGGAGTGGGCTCCACTGCTGTTATTCTAGATGATAACTTTGTAA
CAAAGGCCAATGCCCTAACCTATGACCCCTATGTAAACTACTCCTCCCGCCATACCATAACCCAGCCCTTCTCCTACCAC
TCCCGGTACTTTACCCCGAAACCTGTCCTTGATAGGACAATCGATTACTTCCAACCCAATAACAAAAGAAATCAACTCT
GGCTGAGACTACAAACTACTGGAAATGTAGACCATGTAGGCCTCGGCACTGCGTTCGAAAACAGTATATACGACCAG
GACTACAATATCCGTATAACCATGTATGTACAATTCAGAGAATTTAATCTTAAAGACCCCCCACTTAACCCTAAG-----

>HM027579_pcv2d

ATGACGTATCCAAGGAGGCGTTTCCGCAGACGAAGACACCGCCCCCGCAGCCATCTTGGCCAGATCCTCCGCCGCCGC
CCCTGGCTCGTCCACCCCCGCCACCGTTACCGCTGGAGAAGGAAAAATGGCATCTTCAACACCCGCCTCTCCCGCACCA
TCGGTTATACTGTCAAGAAAACCACAGTCAGAACGCCCTCCTGGAATGTGGACATGATGAGATTTAATATTAATGATT
TTCTTCCCCCAGGAGGGGGCTCAAACCCCCTCACTGTGCCCTTTGAATACTACAGAATAAGGAAGGTTAAGGTTGAAT
TCTGGCCCTGCTCCCCAATCACCCAGGGTGACAGGGGAGTGGGCTCCACTGCTGTTATTCTAGATGATAACTTTGTAA
CAAAGGCCAATGCCCTAACCTATGACCCCTATGTAAACTACTCCTCCCGCCATACCATAACCCAGCCCTTCTCCTACCAC
TCCCGGTACTTTACCCCGAAACCTGTCCTTGATAGGACAATCGATTACTTCCAACCCAATAACAAAAGAAATCAACTCT
GGCTGAGACTACAAACTACTGGAAATGTAGACCATGTAGGCCTCGGCACTGCGTTCGAAAACAGTATATACGACCAG
GACTACAATATCCGTATAACCATGTATGTACAATTCAGAGAATTTAATCTTAAAGACCCCCCACTTAACCCTAAG-----

>HM142894_pcv2d

ATGACGTATCCAAGGAGGCGTTTCCGCAGACGAAGACACCGCCCCCGCAGCCATCTTGGCCAGATCCTCCGCCGCCGC
CCCTGGCTCGTCCACCCCCGCCACCGTTACCGCTGGAGAAGGAAAAATGGCATCTTCAACACCCGCCTCTCCCGCACCA
TCGGTTATACTGTCAAGAAAACCACAGTCAGAACGCCCTCCTGGAATGTGGACATGATGAGATTTAATATTAATGATT
TTCTTCCCCCAGGAGGGGGCTCAAACCCCCTCACTGTGCCCTTTGAATACTACAGAATAAGGAAGGTTAAGGTTGAAT
TCTGGCCCTGCTCCCCAATCACCCAGGGTGACAGGGGAGTGGGCTCCACTGCTGTTATTCTAGATGATAACTTTGTAA
CAAAGGCCAATGCCCTAACCTATGACCCCTATGTAAACTACTCCTCCCGCCATACCATAACCCAGCCCTTCTCCTACCAC
TCCCGGTACTTTACCCCGAAACCTGTCCTTGATAGGACAATCGATTACTTCCAACCCAATAACAAAAGAAATCAACTCT
GGCTGAGACTACAAACTACTGGAAATGTAGACCATGTAGGCCTCGGCACTGCGTTCGAAAACAGTATATACGACCAG
GACTACAATATCCGTATAACCATGTATGTACAATTCAGAGAATTTAATCTTAAAGACCCCCCACTTAACCCTAAG-----

>HM161711_pcv2d

ATGACGTATCCAAGGAGGCGTTTCCGCAGACGAAGACACCGCCCCCGCAGCCATCTTGGCCAGATCCTCCGCCGCCGC
CCCTGGCTCGTCCACCCCCGCCACCGTTACCGCTGGAGAAGGAAAAATGGCATCTTCAACACCCGCCTCTCCCGCACCA
TCGGTTATACTGTCAAGAAAACCACAGTCAGAACGCCCTCCTGGAATGTGGACATGATGAGATTTAATATTAATGATT
TTCTTCCCCCAGGAGGGGGCTCAAACCCCCTCACTGTGCCCTTTGAATACTACAGAATAAGGAAGGTTAAGGTTGAAT
TCTGGCCCTGCTCCCAATCACCCAGGGTGACAGGGGAGTGGGCTCCACTGCTGTTATTCTAGATGATAACTTTGTAA
CAAAGGCCAATGCCCTAACCTATGACCCCTATGTAAACTACTCCTCCCGCCATACCATAACCCAGCCCTTCTCCTACCAC
TCCCGGTACTTTACCCCGAAACCTGTCCTTGATAGGACAATCGATTACTTCCAACCCAATAACAAAAGAATCAACTCT
GGCTGAGACTACAAACTACTGGAAATGTAGACCATGTAGGCCTCGGCACTGCGTTCGAAAACAGTATATACGACCAG
GACTACAATATCCGTATAACCATGTATGTACAATTCAGAGAATTTAATCTTAAAGACCCCCCACTTAACCCTAAG-----

>HQ113117_pcv2d

ATGACGTATCCAAGGAGGCGTTTCCGCAGACGAAGACACCGCCCCCGCAGCCATCTTGGCCAGATCCTCCGCCGCCGC
CCCTGGCTCGTCCACCCCCGCCACCGTTACCGCTGGAGAAGGAAAAATGGCATCTTCAACACCCGCCTCTCCCGCACCA
TCGGTTATACTGTCAAGAAAACCACAGTCAGAACGCCCTCCTGGAATGTGGACATGATGAGATTTAATATTAATGATT
TTCTTCCCCCAGGAGGGGGCTCAAACCCCCTCACTGTGCCCTTTGAATACTACAGAATAAGGAAGGTTAAGGTTGAAT
TCTGGCCCTGCTCCCAATCACCCAGGGTGACAGGGGAGTGGGCTCCACTGCTGTTATTCTAGATGATAACTTTGTAA
CAAAGGCCAATGCCCTAACCTATGACCCCTATGTAAACTACTCCTCCCGCCATACCATAACCCAGCCCTTCTCCTACCAC
TCCCGGTACTTTACCCCGAAACCTGTCCTTGATAGGACAATCGATTACTTCCAACCCAATAACAAAAGAATCAACTCT
GGCTGAGACTACAAACTACTGGAAATGTAGACCATGTAGGCCTCGGCACTGCGTTCGAAAACAGTATATACGACCAG
GACTACAATATCCGTATAACCATGTATGTACAATTCAGAGAATTTAATCTTAAAGACCCCCCACTTAACCCTAAG-----

>JN006448_pcv2d

ATGACGTATCCAAGGAGGCGTTTCCGCAGACGAAGACACCGCCCCCGCAGCCATCTTGGCCAGATCCTCCGCCGCCGC
CCCTGGCTCGTCCACCCCCGCCACCGTTACCGCTGGAGAAGGAAAAATGGCATCTTCAACACCCGCCTCTCCCGCACCA
TCGGTTATACTGTCAAGAAAACCACAGTCAGAACGCCCTCCTGGAATGTGGACATGATGAGATTTAATATTAATGATT
TTCTTCCCCCAGGAGGGGGCTCAAACCCCCTCACTGTGCCCTTTGAATACTACAGAATAAGGAAGGTTAAGGTTGAAT
TCTGGCCCTGCTCCCAATCACCCAGGGTGACAGGGGAGTGGGCTCCACTGCTGTTATTCTAGATGATAACTTTGTAA
CAAAGGCCAATGCCCTAACCTATGACCCCTATGTAAACTACTCCTCCCGCCATACCATAACCCAGCCCTTCTCCTACCAC
TCCCGGTACTTTACCCCGAAACCTGTCCTTGATAGGACAATCGATTACTTCCAACCCAATAACAAAAGAATCAACTCT
GGCTGAGACTACAAACTACTGGAAATGTAGACCATGTAGGCCTCGGCACTGCGTTCGAAAACAGTATATACGACCAG
GACTACAATATCCGTATAACCATGTATGTACAATTCAGAGAATTTAATCTTAAAGACCCCCCACTTAACCCTAAG-----

>JF899334_pcv2d

ATGACGTATCCAAGGAGGCGTTTCCGCAGACGAAGACACCGCCCCCGCAGCCATCTTGGCCAGATCCTCCGCCGCCGC
CCCTGGCTCGTCCACCCCCGCCACCGTTACCGCTGGAGAAGGAAAAATGGCATCTTCAACACCCGCCTCTCCCGCACCA
TCGGTTATACTGTCAAGAAAACCACAGTCAGAACGCCCTCCTGGAATGTGGACATGATGAGATTTAATATTAATGATT
TTCTTCCCCCAGGAGGGGGCTCAAACCCCCTCACTGTGCCCTTTGAATACTACAGAATAAGGAAGGTTAAGGTTGAAT
TCTGGCCCTGCTCCCAATCACCCAGGGTGACAGGGGAGTGGGCTCCACTGCTGTTATTCTAGATGATAACTTTGTAA
CAAAGGCCAATGCCCTAACCTATGACCCCTATGTAAACTACTCCTCCCGCCATACCATAACCCAGCCCTTCTCCTACCAC
TCCCGGTACTTTACCCCGAAACCTGTCCTTGATAGGACAATCGATTACTTCCAACCCAATAACAAAAGAATCAACTCT
GGCTGAGACTACAAACTACTGGAAATGTAGACCATGTAGGCCTCGGCACTGCGTTCGAAAACAGTATATACGACCAG
GACTACAATATCCGTATAACCATGTATGTACAATTCAGAGAATTTAATCTTAAAGACCCCCCACTTAACCCTAAG-----

>JF928002_pcv2d

ATGACGTATCCAAGGAGGCGTTTCCGCAGACGAAGACACCGCCCCCGCAGCCATCTTGGCCAGATCCTCCGCCGCCGC
CCCTGGCTCGTCCACCCCCGCCACCGTTACCGCTGGAGAAGGAAAAATGGCATCTTCAACACCCGCCTCTCCCGCACCA
TCGGTTATACTGTCAAGAAAACCACAGTCAGAACGCCCTCCTGGAATGTGGACATGATGAGATTTAATATTAATGATT
TTCTTCCCCCAGGAGGGGGCTCAAACCCCCTCACTGTGCCCTTTGAATACTACAGAATAAGGAAGGTTAAGGTTGAAT
TCTGGCCCTGCTCCCCAATCACCCAGGGTGACAGGGGAGTGGGCTCCACTGCTGTTATTCTAGATGATAACTTTGTAA
CAAAGGCCAATGCCCTAACCTATGACCCCTATGTAAACTACTCCTCCCGCCATACCATAACCCAGCCCTTCTCCTACCAC
TCCCGGTACTTTACCCCGAAACCTGTCCTTGATAGGACAATCGATTACTTCCAACCCAATAACAAAAGAATCAACTCT
GGCTGAGACTACAAACTACTGGAAATGTAGACCATGTAGGCCTCGGCACTGCGTTCGAAAACAGTATATACGACCAG
GACTACAATATCCGTATAACCATGTATGTACAATTCAGAGAATTTAATCTTAAAGACCCCCCACTTAACCCTAAG-----

>JN119257_pcv2d

ATGACGTATCCAAGGAGGCGTTTCCGCAGACGAAGACACCGCCCCCGCAGCCATCTTGGCCAGATCCTCCGCCGCCGC
CCCTGGCTCGTCCACCCCCGCCACCGTTACCGCTGGAGAAGGAAAAATGGCATCTTCAACACCCGCCTCTCCCGCACCA
TCGGTTATACTGTCAAGAAAACCACAGTCAGAACGCCCTCCTGGAATGTGGACATGATGAGATTTAATATTAATGATT
TTCTTCCCCCAGGAGGGGGCTCAAACCCCCTCACTGTGCCCTTTGAATACTACAGAATAAGGAAGGTTAAGGTTGAAT
TCTGGCCCTGCTCCCCAATCACCCAGGGTGACAGGGGAGTGGGCTCCACTGCTGTTATTCTAGATGATAACTTTGTAA
CAAAGGCCAATGCCCTAACCTATGACCCCTATGTAAACTACTCCTCCCGCCATACCATAACCCAGCCCTTCTCCTACCAC
TCCCGGTACTTTACCCCGAAACCTGTCCTTGATAGGACAATCGATTACTTCCAACCCAATAACAAAAGAATCAACTCT
GGCTGAGACTACAAACTACTGGAAATGTAGACCATGTAGGCCTCGGCACTGCGTTCGAAAACAGTATATACGACCAG
GACTACAATATCCGTATAACCATGTATGTACAATTCAGAGAATTTAATCTTAAAGACCCCCCACTTAACCCTAAG-----

>JN411094_pcv2d

ATGACGTATCCAAGGAGGCGTTTCCGCAGACGAAGACACCGCCCCCGCAGCCATCTTGGCCAGATCCTCCGCCGCCGC
CCCTGGCTCGTCCACCCCCGCCACCGTTACCGCTGGAGAAGGAAAAATGGCATCTTCAACACCCGCCTCTCCCGCACCA
TCGGTTATACTGTCAAGAAAACCACAGTCAGAACGCCCTCCTGGAATGTGGACATGATGAGATTTAATATTAATGATT
TTCTTCCCCCAGGAGGGGGCTCAAACCCCCTCACTGTGCCCTTTGAATACTACAGAATAAGGAAGGTTAAGGTTGAAT
TCTGGCCCTGCTCCCCAATCACCCAGGGTGACAGGGGAGTGGGCTCCACTGCTGTTATTCTAGATGATAACTTTGTAA
CAAAGGCCAATGCCCTAACCTATGACCCCTATGTAAACTACTCCTCCCGCCATACCATAACCCAGCCCTTCTCCTACCAC
TCCCGGTACTTTACCCCGAAACCTGTCCTTGATAGGACAATCGATTACTTCCAACCCAATAACAAAAGAATCAACTCT
GGCTGAGACTACAAACTACTGGAAATGTAGACCATGTAGGCCTCGGCACTGCGTTCGAAAACAGTATATACGACCAG
GACTACAATATCCGTATAACCATGTATGTACAATTCAGAGAATTTAATCTTAAAGACCCCCCACTTAACCCTAAG-----

>HQ395022_pcv2d

ATGACGTATCCAAGGAGGCGTTTCCGCAGACGAAGACACCGCCCCCGCAGCCATCTTGGCCAGATCCTCCGCCGCCGC
CCCTGGCTCGTCCACCCCCGCCACCGTTACCGCTGGAGAAGGAAAAATGGCATCTTCAACACCCGCCTCTCCCGCACCA
TCGGTTATACTGTCAAGAAAACCACAGTCAGAACGCCCTCCTGGAATGTGGACATGATGAGATTTAATATTAATGATT
TTCTTCCCCCAGGAGGGGGCTCAAACCCCCTCACTGTGCCCTTTGAATACTACAGAATAAGGAAGGTTAAGGTTGAAT
TCTGGCCCTGCTCCCCAATCACCCAGGGTGACAGGGGAGTGGGCTCCACTGCTGTTATTCTAGATGATAACTTTGTAA
CAAAGGCCAATGCCCTAACCTATGACCCCTATGTAAACTACTCCTCCCGCCATACCATAACCCAGCCCTTCTCCTACCAC
TCCCGGTACTTTACCCCGAAACCTGTCCTTGATAGGACAATCGATTACTTCCAACCCAATAACAAAAGAATCAACTCT
GGCTGAGACTACAAACTACTGGAAATGTAGACCATGTAGGCCTCGGCACTGCGTTCGAAAACAGTATATACGACCAG
GACTACAATATCCGTATAACCATGTATGTACAATTCAGAGAATTTAATCTTAAAGACCCCCCACTTAACCCTAAG-----

>HQ395023_pcv2d

ATGACGTATCCAAGGAGGCGTTTCCGCAGACGAAGACACCGCCCCCGCAGCCATCTTGGCCAGATCCTCCGCCGCCGC
CCCTGGCTCGTCCACCCCCGCCACCGTTACCGCTGGAGAAGGAAAAATGGCATCTTCAACACCCGCCTCTCCCGCACCA
TCGGTTATACTGTCAAGAAAACCACAGTCAGAACGCCCTCCTGGAATGTGGACATGATGAGATTTAATATTAATGATT
TTCTTCCCCCAGGAGGGGGCTCAAACCCCCTCACTGTGCCCTTTGAATACTACAGAATAAGGAAGGTTAAGGTTGAAT
TCTGGCCCTGCTCCCCAATCACCCAGGGTGACAGGGGAGTGGGCTCCACTGCTGTTATTCTAGATGATAACTTTGTAA
CAAAGGCCAATGCCCTAACCTATGACCCCTATGTAAACTACTCCTCCCGCCATACCATAACCCAGCCCTTCTCCTACCAC
TCCCGGTACTTTACCCCGAAACCTGTCCTTGATAGGACAATCGATTACTTCCAACCCAATAACAAAAGAAATCAACTCT
GGCTGAGACTACAAACTACTGGAAATGTAGACCATGTAGGCCTCGGCACTGCGTTCGAAAACAGTATATACGACCAG
GACTACAATATCCGTATAACCATGTATGTACAATTCAGAGAATTTAATCTTAAAGACCCCCCACTTAACCCTAAG-----

>HQ395032_pcv2d

ATGACGTATCCAAGGAGGCGTTTCCGCAGACGAAGACACCGCCCCCGCAGCCATCTTGGCCAGATCCTCCGCCGCCGC
CCCTGGCTCGTCCACCCCCGCCACCGTTACCGCTGGAGAAGGAAAAATGGCATCTTCAACACCCGCCTCTCCCGCACCA
TCGGTTATACTGTCAAGAAAACCACAGTCAGAACGCCCTCCTGGAATGTGGACATGATGAGATTTAATATTAATGATT
TTCTTCCCCCAGGAGGGGGCTCAAACCCCCTCACTGTGCCCTTTGAATACTACAGAATAAGGAAGGTTAAGGTTGAAT
TCTGGCCCTGCTCCCCAATCACCCAGGGTGACAGGGGAGTGGGCTCCACTGCTGTTATTCTAGATGATAACTTTGTAA
CAAAGGCCAATGCCCTAACCTATGACCCCTATGTAAACTACTCCTCCCGCCATACCATAACCCAGCCCTTCTCCTACCAC
TCCCGGTACTTTACCCCGAAACCTGTCCTTGATAGGACAATCGATTACTTCCAACCCAATAACAAAAGAAATCAACTCT
GGCTGAGACTACAAACTACTGGAAATGTAGACCATGTAGGCCTCGGCACTGCGTTCGAAAACAGTATATACGACCAG
GACTACAATATCCGTATAACCATGTATGTACAATTCAGAGAATTTAATCTTAAAGACCCCCCACTTAACCCTAAG-----

>HQ395052_pcv2d

ATGACGTATCCAAGGAGGCGTTTCCGCAGACGAAGACACCGCCCCCGCAGCCATCTTGGCCAGATCCTCCGCCGCCGC
CCCTGGCTCGTCCACCCCCGCCACCGTTACCGCTGGAGAAGGAAAAATGGCATCTTCAACACCCGCCTCTCCCGCACCA
TCGGTTATACTGTCAAGAAAACCACAGTCAGAACGCCCTCCTGGAATGTGGACATGATGAGATTTAATATTAATGATT
TTCTTCCCCCAGGAGGGGGCTCAAACCCCCTCACTGTGCCCTTTGAATACTACAGAATAAGGAAGGTTAAGGTTGAAT
TCTGGCCCTGCTCCCCAATCACCCAGGGTGACAGGGGAGTGGGCTCCACTGCTGTTATTCTAGATGATAACTTTGTAA
CAAAGGCCAATGCCCTAACCTATGACCCCTATGTAAACTACTCCTCCCGCCATACCATAACCCAGCCCTTCTCCTACCAC
TCCCGGTACTTTACCCCGAAACCTGTCCTTGATAGGACAATCGATTACTTCCAACCCAATAACAAAAGAAATCAACTCT
GGCTGAGACTACAAACTACTGGAAATGTAGACCATGTAGGCCTCGGCACTGCGTTCGAAAACAGTATATACGACCAG
GACTACAATATCCGTATAACCATGTATGTACAATTCAGAGAATTTAATCTTAAAGACCCCCCACTTAACCCTAAG-----

>HM776439_pcv2d

ATGACGTATCCAAGGAGGCGTTTCCGCAGACGAAGACACCGCCCCCGCAGCCATCTTGGCCAGATCCTCCGCCGCCGC
CCCTGGCTCGTCCACCCCCGCCACCGTTACCGCTGGAGAAGGAAAAATGGCATCTTCAACACCCGCCTCTCCCGCACCA
TCGGTTATACTGTCAAGAAAACCACAGTCAGAACGCCCTCCTGGAATGTGGACATGATGAGATTTAATATTAATGATT
TTCTTCCCCCAGGAGGGGGCTCAAACCCCCTCACTGTGCCCTTTGAATACTACAGAATAAGGAAGGTTAAGGTTGAAT
TCTGGCCCTGCTCCCCAATCACCCAGGGTGACAGGGGAGTGGGCTCCACTGCTGTTATTCTAGATGATAACTTTGTAA
CAAAGGCCAATGCCCTAACCTATGACCCCTATGTAAACTACTCCTCCCGCCATACCATAACCCAGCCCTTCTCCTACCAC
TCCCGGTACTTTACCCCGAAACCTGTCCTTGATAGGACAATCGATTACTTCCAACCCAATAACAAAAGAAATCAACTCT
GGCTGAGACTACAAACTACTGGAAATGTAGACCATGTAGGCCTCGGCACTGCGTTCGAAAACAGTATATACGACCAG
GACTACAATATCCGTATAACCATGTATGTACAATTCAGAGAATTTAATCTTAAAGACCCCCCACTTAACCCTAAG-----

>HM776440_pcv2d

ATGACGTATCCAAGGAGGCGTTTCCGCAGACGAAGACACCGCCCCCGCAGCCATCTTGGCCAGATCCTCCGCCGCCGC
CCCTGGCTCGTCCACCCCCGCCACCGTTACCGCTGGAGAAGGAAAAATGGCATCTTCAACACCCGCCTCTCCCGCACCA
TCGGTTATACTGTCAAGAAAACCACAGTCAGAACGCCCTCCTGGAATGTGGACATGATGAGATTTAATATTAATGATT
TTCTTCCCCCAGGAGGGGGCTCAAACCCCCTCACTGTGCCCTTTGAATACTACAGAATAAGGAAGGTTAAGGTTGAAT
TCTGGCCCTGCTCCCCAATCACCCAGGGTGACAGGGGAGTGGGCTCCACTGCTGTTATTCTAGATGATAACTTTGTAA
CAAAGGCCAATGCCCTAACCTATGACCCCTATGTAAACTACTCCTCCCGCCATACCATAACCCAGCCCTTCTCCTACCAC
TCCCGGTACTTTACCCCGAAACCTGTCCTTGATAGGACAATCGATTACTTCCAACCCAATAACAAAAGAATCAACTCT
GGCTGAGACTACAAACTACTGGAAATGTAGACCATGTAGGCCTCGGCACTGCGTTCGAAAACAGTATATACGACCAG
GACTACAATATCCGTATAACCATGTATGTACAATTCAGAGAATTTAATCTTAAAGACCCCCCACTTAACCCTAAG-----

>JF683392_pcv2d

ATGACGTATCCAAGGAGGCGTTTCCGCAGACGAAGACACCGCCCCCGCAGCCATCTTGGCCAGATCCTCCGCCGCCGC
CCCTGGCTCGTCCACCCCCGCCACCGTTACCGCTGGAGAAGGAAAAATGGCATCTTCAACACCCGCCTCTCCCGCACCA
TCGGTTATACTGTCAAGAAAACCACAGTCAGAACGCCCTCCTGGAATGTGGACATGATGAGATTTAATATTAATGATT
TTCTTCCCCCAGGAGGGGGCTCAAACCCCCTCACTGTGCCCTTTGAATACTACAGAATAAGGAAGGTTAAGGTTGAAT
TCTGGCCCTGCTCCCCAATCACCCAGGGTGACAGGGGAGTGGGCTCCACTGCTGTTATTCTAGATGATAACTTTGTAA
CAAAGGCCAATGCCCTAACCTATGACCCCTATGTAAACTACTCCTCCCGCCATACCATAACCCAGCCCTTCTCCTACCAC
TCCCGGTACTTTACCCCGAAACCTGTCCTTGATAGGACAATCGATTACTTCCAACCCAATAACAAAAGAATCAACTCT
GGCTGAGACTACAAACTACTGGAAATGTAGACCATGTAGGCCTCGGCACTGCGTTCGAAAACAGTATATACGACCAG
GACTACAATATCCGTATAACCATGTATGTACAATTCAGAGAATTTAATCTTAAAGACCCCCCACTTAACCCTAAG-----

>JF927989_pcv2d

ATGACGTATCCAAGGAGGCGTTTCCGCAGACGAAGACACCGCCCCCGCAGCCATCTTGGCCAGATCCTCCGCCGCCGC
CCCTGGCTCGTCCACCCCCGCCACCGTTACCGCTGGAGAAGGAAAAATGGCATCTTCAACACCCGCCTCTCCCGCACCA
TCGGTTATACTGTCAAGAAAACCACAGTCAGAACGCCCTCCTGGAATGTGGACATGATGAGATTTAATATTAATGATT
TTCTTCCCCCAGGAGGGGGCTCAAACCCCCTCACTGTGCCCTTTGAATACTACAGAATAAGGAAGGTTAAGGTTGAAT
TCTGGCCCTGCTCCCCAATCACCCAGGGTGACAGGGGAGTGGGCTCCACTGCTGTTATTCTAGATGATAACTTTGTAA
CAAAGGCCAATGCCCTAACCTATGACCCCTATGTAAACTACTCCTCCCGCCATACCATAACCCAGCCCTTCTCCTACCAC
TCCCGGTACTTTACCCCGAAACCTGTCCTTGATAGGACAATCGATTACTTCCAACCCAATAACAAAAGAATCAACTCT
GGCTGAGACTACAAACTACTGGAAATGTAGACCATGTAGGCCTCGGCACTGCGTTCGAAAACAGTATATACGACCAG
GACTACAATATCCGTATAACCATGTATGTACAATTCAGAGAATTTAATCTTAAAGACCCCCCACTTAACCCTAAG-----

>GQ915288_pcv2d

ATGACGTATCCAAGGAGGCGTTTCCGCAGACGAAGACACCGCCCCCGCAGCCATCTTGGCCAGATCCTCCGCCGCCGC
CCCTGGCTCGTCCACCCCCGCCACCGTTACCGCTGGAGAAGGAAAAATGGCATCTTCAACACCCGCCTCTCCCGCACCA
TCGGTTATACTGTCAAGAAAACCACAGTCAGAACGCCCTCCTGGAATGTGGACATGATGAGATTTAATATTAATGATT
TTCTTCCCCCAGGAGGGGGCTCAAACCCCCTCACTGTGCCCTTTGAATACTACAGAATAAGGAAGGTTAAGGTTGAAT
TCTGGCCCTGCTCCCCAATCACCCAGGGTGACAGGGGAGTGGGCTCCACTGCTGTTATTCTAGATGATAACTTTGTAA
CAAAGGCCAATGCCCTAACCTATGACCCCTATGTAAACTACTCCTCCCGCCATACCATAACCCAGCCCTTCTCCTACCAC
TCCCGGTACTTTACCCCGAAACCTGTCCTTGATAGGACAATCGATTACTTCCAACCCAATAACAAAAGAATCAACTCT
GGCTGAGACTACAAACTACTGGAAATGTAGACCATGTAGGCCTCGGCACTGCGTTCGAAAACAGTATATACGACCAG
GACTACAATATCCGTATAACCATGTATGTACAATTCAGAGAATTTAATCTTAAAGACCCCCCACTTAACCCTAAG-----

>JX535297_pcv2d

ATGACGTATCCAAGGAGGCGTTTCCGCAGACGAAGACACCGCCCCCGCAGCCATCTTGGCCAGATCCTCCGCCGCCGC
CCCTGGCTCGTCCACCCCCGCCACCGTTACCGCTGGAGAAGGAAAAATGGCATCTTCAACACCCGCCTCTCCCGCACCA
TCGGTTATACTGTCAAGAAAACCACAGTCAGAACGCCCTCCTGGAATGTGGACATGATGAGATTTAATATTAATGATT
TTCTTCCCCCAGGAGGGGGCTCAAACCCCCTCACTGTGCCCTTTGAATACTACAGAATAAGGAAGGTTAAGGTTGAAT
TCTGGCCCTGCTCCCCAATCACCCAGGGTGACAGGGGAGTGGGCTCCACTGCTGTTATTCTAGATGATAACTTTGTAA
CAAAGGCCAATGCCCTAACCTATGACCCCTATGTAAACTACTCCTCCCGCCATACCATAACCCAGCCCTTCTCCTACCAC
TCCCGGTACTTTACCCCGAAACCTGTCCTTGATAGGACAATCGATTACTTCCAACCCAATAACAAAAGAATCAACTCT
GGCTGAGACTACAAACTACTGGAAATGTAGACCATGTAGGCCTCGGCACTGCGTTCGAAAACAGTATATACGACCAG
GACTACAATATCCGTATAACCATGTATGTACAATTCAGAGAATTTAATCTTAAAGACCCCCCACTTAACCCTAAG-----

>JQ181600_pcv2d

ATGACGTATCCAAGGAGGCGTTTCCGCAGACGAAGACACCGCCCCCGCAGCCATCTTGGCCAGATCCTCCGCCGCCGC
CCCTGGCTCGTCCACCCCCGCCACCGTTACCGCTGGAGAAGGAAAAATGGCATCTTCAACACCCGCCTCTCCCGCACCA
TCGGTTATACTGTCAAGAAAACCACAGTCAGAACGCCCTCCTGGAATGTGGACATGATGAGATTTAATATTAATGATT
TTCTTCCCCCAGGAGGGGGCTCAAACCCCCTCACTGTGCCCTTTGAATACTACAGAATAAGGAAGGTTAAGGTTGAAT
TCTGGCCCTGCTCCCCAATCACCCAGGGTGACAGGGGAGTGGGCTCCACTGCTGTTATTCTAGATGATAACTTTGTAA
CAAAGGCCAATGCCCTAACCTATGACCCCTATGTAAACTACTCCTCCCGCCATACCATAACCCAGCCCTTCTCCTACCAC
TCCCGGTACTTTACCCCGAAACCTGTCCTTGATAGGACAATCGATTACTTCCAACCCAATAACAAAAGAATCAACTCT
GGCTGAGACTACAAACTACTGGAAATGTAGACCATGTAGGCCTCGGCACTGCGTTCGAAAACAGTATATACGACCAG
GACTACAATATCCGTATAACCATGTATGTACAATTCAGAGAATTTAATCTTAAAGACCCCCCACTTAACCCTAAG-----

>JX535296_pcv2d

ATGACGTATCCAAGGAGGCGTTTCCGCAGACGAAGACACCGCCCCCGCAGCCATCTTGGCCAGATCCTCCGCCGCCGC
CCCTGGCTCGTCCACCCCCGCCACCGTTACCGCTGGAGAAGGAAAAATGGCATCTTCAACACCCGCCTCTCCCGCACCA
TCGGTTATACTGTCAAGAAAACCACAGTCAGAACGCCCTCCTGGAATGTGGACATGATGAGATTTAATATTAATGATT
TTCTTCCCCCAGGAGGGGGCTCAAACCCCCTCACTGTGCCCTTTGAATACTACAGAATAAGGAAGGTTAAGGTTGAAT
TCTGGCCCTGCTCCCCAATCACCCAGGGTGACAGGGGAGTGGGCTCCACTGCTGTTATTCTAGATGATAACTTTGTAA
CAAAGGCCAATGCCCTAACCTATGACCCCTATGTAAACTACTCCTCCCGCCATACCATAACCCAGCCCTTCTCCTACCAC
TCCCGGTACTTTACCCCGAAACCTGTCCTTGATAGGACAATCGATTACTTCCAACCCAATAACAAAAGAATCAACTCT
GGCTGAGACTACAAACTACTGGAAATGTAGACCATGTAGGCCTCGGCACTGCGTTCGAAAACAGTATATACGACCAG
GACTACAATATCCGTATAACCATGTATGTACAATTCAGAGAATTTAATCTTAAAGACCCCCCACTTAACCCTAAG-----

>JX948778_pcv2d

ATGACGTATCCAAGGAGGCGTTTCCGCAGACGAAGACACCGCCCCCGCAGCCATCTTGGCCAGATCCTCCGCCGCCGC
CCCTGGCTCGTCCACCCCCGCCACCGTTACCGCTGGAGAAGGAAAAATGGCATCTTCAACACCCGCCTCTCCCGCACCA
TCGGTTATACTGTCAAGAAAACCACAGTCAGAACGCCCTCCTGGAATGTGGACATGATGAGATTTAATATTAATGATT
TTCTTCCCCCAGGAGGGGGCTCAAACCCCCTCACTGTGCCCTTTGAATACTACAGAATAAGGAAGGTTAAGGTTGAAT
TCTGGCCCTGCTCCCCAATCACCCAGGGTGACAGGGGAGTGGGCTCCACTGCTGTTATTCTAGATGATAACTTTGTAA
CAAAGGCCAATGCCCTAACCTATGACCCCTATGTAAACTACTCCTCCCGCCATACCATAACCCAGCCCTTCTCCTACCAC
TCCCGGTACTTTACCCCGAAACCTGTCCTTGATAGGACAATCGATTACTTCCAACCCAATAACAAAAGAATCAACTCT
GGCTGAGACTACAAACTACTGGAAATGTAGACCATGTAGGCCTCGGCACTGCGTTCGAAAACAGTATATACGACCAG
GACTACAATATCCGTATAACCATGTATGTACAATTCAGAGAATTTAATCTTAAAGACCCCCCACTTAACCCTAAG-----

>JX982221_pcv2d

ATGACGTATCCAAGGAGGCGTTTCCGCAGACGAAGACACCGCCCCCGCAGCCATCTTGGCCAGATCCTCCGCCGCCGC
CCCTGGCTCGTCCACCCCCGCCACCGTTACCGCTGGAGAAGGAAAAATGGCATCTTCAACACCCGCCTCTCCCGCACCA
TCGGTTATACTGTCAAGAAAACCACAGTCAGAACGCCCTCCTGGAATGTGGACATGATGAGATTTAATATTAATGATT
TTCTTCCCCCAGGAGGGGGCTCAAACCCCCTCACTGTGCCCTTTGAATACTACAGAATAAGGAAGGTTAAGGTTGAAT
TCTGGCCCTGCTCCCCAATCACCCAGGGTGACAGGGGAGTGGGCTCCACTGCTGTTATTCTAGATGATAACTTTGTAA
CAAAGGCCAATGCCCTAACCTATGACCCCTATGTAAACTACTCCTCCCGCCATACCATAACCCAGCCCTTCTCCTACCAC
TCCCGGTACTTTACCCCGAAACCTGTCCTTGATAGGACAATCGATTACTTCCAACCCAATAACAAAAGAAATCAACTCT
GGCTGAGACTACAAACTACTGGAAATGTAGACCATGTAGGCCTCGGCACTGCGTTCGAAAACAGTATATACGACCAG
GACTACAATATCCGTATAACCATGTATGTACAATTCAGAGAATTTAATCTTAAAGACCCCCCACTTAACCCTAAG-----

>KC473167_pcv2d

ATGACGTATCCAAGGAGGCGTTTCCGCAGACGAAGACACCGCCCCCGCAGCCATCTTGGCCAGATCCTCCGCCGCCGC
CCCTGGCTCGTCCACCCCCGCCACCGTTACCGCTGGAGAAGGAAAAATGGCATCTTCAACACCCGCCTCTCCCGCACCA
TCGGTTATACTGTCAAGAAAACCACAGTCAGAACGCCCTCCTGGAATGTGGACATGATGAGATTTAATATTAATGATT
TTCTTCCCCCAGGAGGGGGCTCAAACCCCCTCACTGTGCCCTTTGAATACTACAGAATAAGGAAGGTTAAGGTTGAAT
TCTGGCCCTGCTCCCCAATCACCCAGGGTGACAGGGGAGTGGGCTCCACTGCTGTTATTCTAGATGATAACTTTGTAA
CAAAGGCCAATGCCCTAACCTATGACCCCTATGTAAACTACTCCTCCCGCCATACCATAACCCAGCCCTTCTCCTACCAC
TCCCGGTACTTTACCCCGAAACCTGTCCTTGATAGGACAATCGATTACTTCCAACCCAATAACAAAAGAAATCAACTCT
GGCTGAGACTACAAACTACTGGAAATGTAGACCATGTAGGCCTCGGCACTGCGTTCGAAAACAGTATATACGACCAG
GACTACAATATCCGTATAACCATGTATGTACAATTCAGAGAATTTAATCTTAAAGACCCCCCACTTAACCCTAAG-----

>JX679498_pcv2d

ATGACGTATCCAAGGAGGCGTTTCCGCAGACGAAGACACCGCCCCCGCAGCCATCTTGGCCAGATCCTCCGCCGCCGC
CCCTGGCTCGTCCACCCCCGCCACCGTTACCGCTGGAGAAGGAAAAATGGCATCTTCAACACCCGCCTCTCCCGCACCA
TCGGTTATACTGTCAAGAAAACCACAGTCAGAACGCCCTCCTGGAATGTGGACATGATGAGATTTAATATTAATGATT
TTCTTCCCCCAGGAGGGGGCTCAAACCCCCTCACTGTGCCCTTTGAATACTACAGAATAAGGAAGGTTAAGGTTGAAT
TCTGGCCCTGCTCCCCAATCACCCAGGGTGACAGGGGAGTGGGCTCCACTGCTGTTATTCTAGATGATAACTTTGTAA
CAAAGGCCAATGCCCTAACCTATGACCCCTATGTAAACTACTCCTCCCGCCATACCATAACCCAGCCCTTCTCCTACCAC
TCCCGGTACTTTACCCCGAAACCTGTCCTTGATAGGACAATCGATTACTTCCAACCCAATAACAAAAGAAATCAACTCT
GGCTGAGACTACAAACTACTGGAAATGTAGACCATGTAGGCCTCGGCACTGCGTTCGAAAACAGTATATACGACCAG
GACTACAATATCCGTATAACCATGTATGTACAATTCAGAGAATTTAATCTTAAAGACCCCCCACTTAACCCTAAG-----

>HM776447_pcv2d

ATGACGTATCCAAGGAGGCGTTTCCGCAGACGAAGACACCGCCCCCGCAGCCATCTTGGCCAGATCCTCCGCCGCCGC
CCCTGGCTCGTCCACCCCCGCCACCGTTACCGCTGGAGAAGGAAAAATGGCATCTTCAACACCCGCCTCTCCCGCACCA
TCGGTTATACTGTCAAGAAAACCACGGTCAGAACGCCCTCCTGGAATGTGGACATGATGAGATTTAATATTAATGATT
TTCTTCCCCCAGGAGGGGGCTCAAACCCCCTCACTGTGCCCTTTGAATACTACAGAATAAGGAAGGTTAAGGTTGAAT
TCTGGCCCTGCTCCCCAATCACCCAGGGTGACAGGGGAGTGGGCTCCACTGCTGTTATTCTAGATGATAACTTTGTAA
CAAAGGCCAATGCCCTAACCTATGACCCCTATGTAAACTACTCCTCCCGCCATACCATAACCCAGCCCTTCTCCTACCAC
TCCCGGTACTTTACCCCGAAACCTGTCCTTGATAGGACAATCGATTACTTCCAACCCAATAACAAAAGAAATCAACTCT
GGCTGAGACTACAAACTACTGGAAATGTAGACCATGTAGGCCTCGGCACTGCGTTCGAAAACAGTATATACGACCAG
GACTACAATATCCGTATAACCATGTATGTACAATTCAGAGAATTTAATCTTAAAGACCCCCCACTTAACCCTAAG-----

>JX406425_pcv2d

ATGACGTATCCAAGGAGGCGTTTCCGCAGACGAAGACACCGCCCCCGCAGCCATCTTGGCCAGATCCTCCGCCGCCGC
CCCTGGCTCGTCCACCCCCGCCACCGTTACCGCTGGAGAAGGAAAAATGGCATCTTCAACACCCGCCTCTCTCGCACCA
TCGGTTATACTGTCAAGAAAACCACAGTCAGAACGCCCTCCTGGAATGTGGACATGATGAGATTTAATATTAATGATT
TTCTTCCCCCAGGAGGGGGCTCAAACCCCCTCACTGTGCCCTTTGAATACTACAGAATAAGGAAGGTTAAGGTTGAAT
TCTGGCCCTGCTCCCCAATCACCCAGGGTGACAGGGGAGTGGGCTCCACTGCTGTTATTCTAGATGATAACTTTGTAA
CAAAGGCCAATGCCCTAACCTATGACCCCTATGTAAACTACTCCTCCCGCCATACCATAACCCAGCCCTTCTCCTACCAC
TCCCGGTACTTTACCCCGAAACCTGTCCTTGATAGGACAATCGATTACCTCCAACCCAATAACAAAAGAAATCAACTCT
GGCTGAGACTACAAACTACTGGAAATGTAGACCATGTAGGCCTCGGCACTGCGTTCGAAAACAGTATATACGACCAG
GACTACAATATCCGTATAACCATGTATGTACAATTCAGAGAATTTAATCTTAAAGACCCCCCACTTAACCCTAAG-----

>KC533812_pcv2d

ATGACGTATCCAAGGAGGCGTTTCCGCAGACGAAGACACCGCCCCCGCAGCCATCTTGGCCAGATCCTCCGCCGCCGC
CCCTGGCTCGTCCACCCCCGCCACCGTTACCGCTGGAGAAGGAAAAATGGCATCTTCAACACCCGCCTCTCCCGCACCA
TCGGTTATACTGTCAAGAAAACCACGGTCAGAACGCCCTCCTGGAATGTGGACATGATGAGATTTAATATTAATGATT
TTCTTCCCCCAGGAGGGGGCTCAAACCCCCTCACTGTGCCCTTTGAATACTACAGAATAAGGAAGGTTAAGGTTGAAT
TCTGGCCCTGCTCCCCAATCACCCAGGGTGACAGGGGAGTGGGCTCCACTGCTGTTATTCTAGATGATAACTTTGTAA
CAAAGGCCAATGCCCTAACCTATGACCCCTATGTAAACTACTCCTCCCGCCATACCATAACCCAGCCCTTCTCCTACCAC
TCCCGGTACTTTACCCCAAAACCTGTCCTTGATAGGACAATCGATTACCTCCAACCCAATAACAAAAGAAATCAACTCT
GGCTGAGACTACAAACTACTGGAAATGTAGACCATGTAGGCCTCGGCACTGCGTTCGAAAACAGTATATACGACCAG
GACTACAATATCCGTATAACCATGTATGTACAATTCAGAGAATTTAATCTTAAAGACCCCCCACTTAACCCTAAA-----

>HM038031_pcv2d

ATGACGTATCCAAGGAGGCGTTTCCGCAGACGAAGACACCGCCCCCGCAGCCATCTTAGCCAGATCCTCCGCCGCCGC
CCCTGGCTCGTCCACCCCCGCCACCGTTACCGCTGGAGAAGGAAAAATGGCATCTTCAACACCCGCCTCTCCCGCACCA
TCGGTTATACTGTCAAGAAAACCACAGTCAGAACGCCCTCCTGGAATGTGGACATGATGAGATTTAATATTAATGATT
TTCTTCCCCCAGGAGGGGGCTCAAACCCCCTCACTGTGCCCTTTGAATACTACAGAATAAGGAAGGTTAAGGTTGAAT
TCTGGCCCTGCTCCCCAATCACCCAGGGTGACAGGGGAGTGGGCTCCACTGCTGTTATTCTAGATGATAACTTTGTAA
CAAAGGCCAATGCCCTAACCTATGACCCCTATGTAAACTACTCCTCCCGCCATACCATAACCCAGCCCTTCTCCTACCAC
TCCCGGTACTTTACCCCGAAACCTGTCCTTGATAGGACAATCGATTACTTCCAACCCAATAACAAAAGAAATCAACTCT
GGCTGAGACTACAAACTACTGGAAATGTAGACCATGTAGGCCTCGGCACTGCGTTCGAAAACAGTATATACGACCAG
GACTACAATATCCGTATAACCATGTATGTACAATTCAGAGAATTTAATCTTAAAGACCCCCCACTTAACCCTAAG-----

>JX948785_pcv2d

ATGACGTATCCAAGGAGGCGTTTCCGCAGACGAAGACACCGCCCCCGCAGCCATCTTAGCCAGATCCTCCGCCGCCGC
CCCTGGCTCGTCCACCCCCGCCACCGTTACCGCTGGAGAAGGAAAAATGGCATTTTCAACACCCGCCTCTCCCGCACCA
TCGGTTATACTGTCAAGAAAACCACAGTCAGAACGCCCTCCTGGAATGTGGACATGATGAGATTTAATATTAATGATT
TTCTTCCCCCAGGAGGGGGCTCAAACCCCCTCACTGTGCCCTTTGAATACTACAGAATAAGAAAGGTTAAGGTTGAAT
TCTGGCCCTGCTCCCCAATCACCCAGGGTGACAGGGGAGTGGGCTCCACTGCTGTTATTCTAGATGATAACTTTGTAA
CAAAGGCCAATGCCCTAACCTATGACCCCTATGTAAACTACTCCTCCCGCCATACCATAACCCAGCCCTTCTCCTACCAC
TCCCGGTACTTTACCCCGAAACCTGTCCTTGATAGGACAATCGATTACTTCCAACCCAATAACAAAAGAAATCAACTCT
GGCTGAGACTACAAACTACTGGAAATGTAGACCATGTAGGCCTCGGCACTGCATTCGAAAACAGTATATACGACCAG
GACTACAATATCCGTATAACCATGTATGTACAATTCAGAGAATTTAATCTTAAAGACCCCCCACTTAACCCTAAG-----

>GU252370_pcv2d

ATGACGTATCCAAGGAGGCGTTTCCGCAGACGAAGACACCGCCCCCGCAGCCATCTTGGCCAGATCCTCCGCCGCCGC
CCCTGGCTCGTCCACCCCCGCCACCGTTACCGCTGGAGAAGGAAAAATGGCATTTTCAACACCCGCCTCTCCCGCACCA
TCGGTTATACTGTCAAGAAAACCACAGTAAGAACGCCCTCCTGGAATGTGGACATGATGAGATTTAATATTAATGATT
TTCTTCCCCCAGGAGGGGGCTCAAACCCCCTCACTGTGCCCTTTGAATACTACAGAATAAGGAAGGTTAAGGTTGAAT
TCTGGCCCTGCTCCCCAATCACCCAGGGTGACAGGGGAGTGGGCTCCACTGCTGTTATTCTAGATGATAACTTTGTAA
CAAAGGCCAATGCCCTAACCTATGACCCCTATGTAAACTACTCCTCCCGCCATACCATAACCCAGCCCTTCTCCTACCAC
TCCCGGTACTTTACCCCGAAACCTGTCCTTGATAGGACAATCGATTACTTCCAACCCAATAACAAAAGAAATCAACTCT
GGCTGAGACTACAAACTACTGGAAATGTAGACCATGTAGGCCTCGGCACTGCGTTCGAAAACAGTATATACGACCAG
GACTACAATATCCGTATAACCATGTATGTACAATTCAGAGAATTTAATCTTAAAGACCCCCCACTTAACCCTAAG-----

>JX982219_pcv2d

ATGACGTATCCAAGGAGGCGTTTCCGCAGACGAAGACACCGCCCCCGCAGCCATCTTGGCCAGATCCTCCGCCGGGG
GCCCGGGCTCGTCCACCCCCGCCACCGTTACCGCTGGAGAAGGAAAAATGGCATCTTCAACACCCGCCTCTCCCGCAC
CATCGGTTATACTGTCAAGAAAACCACAGTCAGAACGCCCTCCTGGAATGTGGACATGATGAGATTTAATATTAATGA
TTTTCTTCCCCCAGGAGGGGGCTCAAACCCCCTCACTGTGCCCTTTGAATACTACAGAATAAGAAAGGTTAAGGTTGA
ATTCTGGCCCTGCTCCCCAATCACCCAAGGTGACAGGGGAGTGGGCTCCACTGCTGTTATTCTAGATGATAACTTTGTA
ACAAAGGCCAATGCCCTAACCTATGACCCCTATGTAAACTACTCCTCCCGCCATACCATAACCCAGCCCTTCTCCTACCA
CTCACGGTACTTTACCCCGAAACCTGTCCTTGATAGGACAATCGATTACTTCCAACCCAATAACAAAAGAAATCAACTC
TGGCTGAGACTACAAACTACTGGAAATGTAGACCATGTAGGCCTCGGCACTGCGTTCGAAAACAGTATATACGACCA
GGACTACAATATCCGTATAACCATGTATGTACAATTCAGAGAATTTAATCTTAAAGACCCCCCACTTAACCCTAAG-----

>FJ870973_pcv2d

ATGACGTATCCAAGGAGGCGTTTCCGCAGACGAAGACACCGCCCCCGCAGCCATCTTGGCCAGATCCTCCGCCGCCGC
CCCTGGCTCGTCCACCCCCGCCACCGTTACCGCTGGAGAAGGAAAAATGGCATCTTCAACACCCGCCTCTCCCGCACCA
TCGGTTATACTGTCAAGAAAACCACAGTCAGAACGCCCTCCTGGAATGTGGACATGATGAGATTTAATATTAATGATT
TTCTTCCCCCAGGAGGGGGCTCAAACCCCCTCACTGTGCCCTTTGAATACTACAGAATAAGGAAGGTTAAGGTTGAAT
TCTGGCCCTGCTCCCCAATCACCCAAGGTGACAGGGGAGTGGGCTCCACTGCTGTTATTCTAGATGATAACTTTGTAAC
AAAGGCCAATGCCCTAACCTATGACCCATATGTAAACTACTCCTCCCGCCATACCATAACCCAGCCCTTCTCCTACCACT
CCCGGTACTTTACCCCGAAACCTGTCCTTGATAGGACAATCGATTACTTCCAACCCAATAACAAAAGAAATCAACTCTG
GCTGAGACTACAAACTACTGGAAATGTAGACCATGTAGGCCTCGGCACTGCGTTCGAAAACAGTATATACGACCAGG
ACTACAATATCCGTATAACCATGTATGTACAATTCAGAGAATTTAATCTTAAAGACCCCCCACTTAACCCTAAG-----

>KC800645_pcv2d

ATGACGTATCCAAGGAGGCGTTTCCGCAGACGAAGACACCGCCCCCGCAGCCATCTTGGCCATATCCTCCGCCGCCGC
CCCTGGCTCGTCCACCCCCGCCACCGTTACCGCTGGAGAAGGAAAAATGGCATCTTCAACACCCGCCTCTCCCGCACCA
TCGGTTATACTGTCAAGAAAACCACAGTCAGAACGCCCTCCTGGAATGTGGACATGATGAGATTTAATATTAATGATT
TTCTTCCCCCAGGAGGGGGCTCAAACCCCCTCACTGTGCCCTTTGAATACTACAGAATAAGGAAGGTTAAGGTTGAAT
TCTGGCCCTGCTCCCCCATCACCCAAGGTGACAGGGGAGTAGGCTCCACTGCTGTTATTCTAGATGATAACTTTGTAAC
AAAGGCCAATGCCCTAACCTATGACCCCTATGTAAACTACTCCTCCCGCCATACCATAACCCAGCCCTTCTCCTACCACT
CCCGGTACTTTACCCCGAAACCTGTCCTTGATAGGACAATCGATTACTTCCAACCCAATAACAAAAGAAATCAACTCTG
GCTGAGACTACAAACTACTGGAAATGTAGACCATGTAGGCCTCGGCACTGCGTTCGAAAACAGTATATACGACCAGG
ACTACAATATCCGTATAACCATGTATGTACAATTCAGAGAATTTAATCTTAAAGACCCCCCACTTAACCCT--------

>HQ693093_pcv2d

ATGACGTATCCAAGGAGGCGTTTCCGCAGACGAAGATACCGCCCCCGCAGCCATCTTGGCCAGATCCTCCGCCGCCGC
CCCTGGCTCGTCCACCCCCGCCACCGTTACCGCTGGAGAAGGAAAAATGGCATCTTCAACACCCGCCTCTCCCGCACCA
TCGGTTATACTGTCAAGAAAACCACAGTCAGAACCCCCTCCTGGAATGTGGACATGATGAGATTTAATATTAATGATTT
TCTTCCCCCAGGAGGGGGCTCAAACCCCCTCACTGTGCCCTTTGAATACTACAGAATAAGGAAGGTTAAGGTTGAATT
CTGGCCCTGCTCCCCAATCACCCAGGGTGACAGGGGAGTGGGCTCCACTGCTGTTATTCTAGATGATAACTTTGTAAC
AAAGGCCAATGCCCTAACCTATGACCCCTATGTAAACTACTCCTCCCGCCATACCATAACCCAGCCCTTCTCCTACCACT
CCCGGTACTTTACCCCGAAACCTGTCCTTGATAGGACAATCGATTACTTCCAACCCAATAACAAAAGAAATCAACTCTG
GCTGAGACTACAAACTACTGGAAATGTAGACCATGTAGGCCTCGGCACTGCGTTCGAAAACAGTATATACGACCAGG
ACTACAATATCCGTATAACCATGTATGTACAATTCAGAGAATTTAATCTTAAAGACCCCCCACTTAACCCTAAG-----

>GU325769_pcv2d

ATGACGTATCCAAGGAGGCGTTTCCGCAGACGAAGACACCGCCCCCGCAGCCATCTTGGCCAGATCCTCCGCCGCCGC
CCCTGGCTCGTCCACCCCCGCCACCGTTACCGCTGGAGAAGGAAAAATGGCATCTTCAACACCCGCCTCTCCCGCACCA
TCGGTTATACTGTCAAGAAAACCACAGTCAGAACCCCCTCCTGGAATGTGGACATGATGAGATTTAATATTAATGATTT
TCTTCCCCCAGGAGGGGGCTCAAACCCCCTCACTGTGCCCTTTGAATACTACAGAATAAGAAAGGTTAAGGTTGAATT
CTGGCCCTGCTCCCCAATCACCCAGGGTGACAGGGGAGTGGGCTCCACTGCTGTTATTCTAGATGATAACTTTGTAAC
AAAGGCCAATGCCCTAACCTATGACCCCTATGTAAACTACTCCTCCCGCCATACCATAACCCAGCCCTTCTCCTACCACT
CCCGGTACTTTACCCCGAAACCTGTCCTTGATAGGACAATCGATTACTTCCAACCCAATAACAAAAGAAATCAACTCTG
GCTGAGACTCCAAACTACTGGAAATGTAGACCATGTAGGCCTCGGCACTGCGTTCGAAAACAGTATATACGACCAGG
ACTACAATATCCGTATAACCATGTATGAACAATTCAGAGAATTTAATCTTAAAGACCCCCCACTTAACCCTAAG-----

>HM776437_pcv2d

ATGACGTATCCAAGGAGGCGTTTCCGCAGACGAAGACACCGCCCCCGCAGCCATCTTGGCCAGATCCTCCGCCGCCGC
CCCTGGCTCGTCCACCCCCGCCACCGTTACCGCTGGAGAAGGAAAAATGGCATCTTCAACACCCGCCTCTCCCGCACCA
TCGGTTATACTGTCAAGAAAACCACAGTCAGAACCCCCTCCTGGAATGTGGACATGATGAGATTTAATATTAATGATTT
TCTTCCCCCAGGAGGGGGCTCAAACCCCCTCACTGTGCCCTTTGAATACTACAGAATAAGGAAGGTTAAGGTTGAATT
CTGGCCCTGCTCCCCAATCACCCAGGGTGACAGGGGAGTGGGCTCCACTGCTGTTATTCTAGATGATAACTTTGTAAC
AAAGGCCAATGCCCTAACCTATGACCCCTATGTAAACTACTCCTCCCGCCATACCATAACCCAGCCCTTCTCCTACCACT
CCCGGTACTTTACCCCGAAACCTGTCCTTGATAGGACAATCGATTACTTCCAACCCAATAACAAAAGAAATCAACTCTG
GCTGAGACTCCAAACTACTGGAAATGTAGACCATGTAGGCCTCGGCACTGCGTTCGAAAACAGTATATACGACCAGG
ACTACAATATCCGTATAACCATGTATGTACAATTCAGAGAATTTAATCTTAAAGACCCCCCACTTAACCCTAAG-----

>KC800641_pcv2d

ATGACGTATCCAAGGAGGCGTTTCCGCAGACGAAGACACCGCCCCCGCAGCCATCTTGGCCAGATCCTCCGCCGCCGC
CCCTGGCTCGTCCACCCCCGCCACCGTTACCGCTGGAGAAGGAAAAATGGCATCTTCAACACCCGCCTCTCCCGCACCA
TCGGTTATACTGTCAAGAAAACCACAGTCAGAACCCCCTCCTGGAATGTGGACATGATGAGATTTAATATTAATGATTT
TCTTCCCCCAGGAGGGGGCTCAAACCCCCTCACTGTGCCCTTTGAATACTACAGAATAAGGAAGGTTAAGGTTGAATT
CTGGCCCTGCTCCCCAATCACCCAGGGTGACAGGGGAGTGGGCTCCACTGCTGTTATTCTAGATGATAACTTTGTAAC
AAAGGCCAATGCCCTAACCTATGACCCCTATGTAAACTACTCCTCCCGCCATACCATAACCCAGCCCTTCTCCTACCACT
CCCGGTACTTTACCCCGAAACCTGTCCTTGATAGCACAATCGATTACTTCCAACCCAATAACAAAAGAAATCAACTCTG
GCTGAGACTCCAAACTACTGGAAATGTAGACCATGTAGGCCTCGGCACTGCGTTCGAAAACAGTATATACGACCAGG
ACTACAATATCCGTATAACCATGTATGTACAATTCAGAGAATTTAATCTTAAAGACCCCCCACTTAACCCT--------

>KC800642_pcv2d

ATGACGTATCCAAGGAGGCGTTTCCGCAGACGAAGACACCGCCCCCGCAGCCATCTTGGCCAGATCCTCCGCCGCCGC
CCCTGGCTCGTCCACCCCCGCCACCGTTACCGCTGGAGAAGGAAAAATGGCATCTTCAACACCCGCCTCTCCCGCACCA
TCGGTTATACTGTCAAGAAAACCACAGTCAGAACCCCCTCCTGGAATGTGGACATGATGAGATTTAATATTAATGATTT
TCTTCCCCCAGGAGGGGGCTCAAACCCCCTCACTGTGCCCTTTGAATACTACAGAATAAGGAAGGTTAAGGTTGAATT
CTGGCCCTGCTCCCCAATCACCCAGGGTGACAGGGGAGTGGGCTCCACTGCTGTTATTCTAGATGATAACTTTGTAAC
AAAGGCCAATGCCCTAACCTATGACCCCTATGTAAACTACTCCTCCCGCCATACCATAACCCAGCCCTTCTCCTACCACT
CCCGGTACTTTACCCCGAAACCTGTCCTTGATAGCACAATCGATTACTTCCAACCCAATAACAAAAGAAATCAACTCTG
GCTGAGACTCCAAACTACTGGAAATGTAGACCATGTAGGCCTCGGCACTGCGTTCGAAAACAGTATATACGACCAGG
ACTACAATATCCGTATAACCATGTATGTACAATTCAGAGAATTTAATCTTAAAGACCCCCCACTTAACCCT--------

>KC800644_pcv2d

ATGACGTATCCAAGGAGGCGTTTCCGCAGACGAAGACACCGCCCCCGCAGCCATCTTGGCCAGATCCTCCGCCGCCGC
CCCTGGCTCGTCCACCCCCGCCACCGTTACCGCTGGAGAAGGAAAAATGGCATCTTCAACACCCGCCTCTCCCGCACCA
TCGGTTATACTGTCAAGAAAACCACAGTCAGAACGCCCTCCTGGAATGTGGACATGATGAGATTTAATATTAATGATT
TTCTTCCCCCAGGAGGGGGCTCAAACCCCCTCACTGTGCCCTTTGAATACTACAGAATAAGGAAGGTTAAGGTTGAAT
TCTGGCCCTGCTCCCCAATCACCCAGGGTGACAGGGGAGTGGGCTCCACTGCTGTTATTCTAGATGATAACTTTGTAA
CAAAGGCCAATGCCCTAACCTATGACCCCTATGTAAACTACTCCTCCCGCCATACCATAACCCAGCCCTTCTCCTACCAC
TCCCGGTACTTTACCCCGAAACCTGTCCTTGATAGGACAATCGATTACTTCCAACCCAATAACAAAAGAAATCAACTCT
GGCTGAGACTACAAACTACTGGAAATGTAGACCATGTAGGCCTCGGCACTGCGTTCGAAAACAGTATATACGACCAG
GACTACAATATCCGTATAACCATGTATGTACAATTCAGAGAATTTAATCTTAAAGACCCCCCACTTAACCCT--------

>JQ809463_pcv2d

ATGACGTATCCAAGGAGGCGTTTCCGCAGACGAAGACACCGCCCCCGCAGCCATCTTGGCCAGATCCTCCGCCGCCGC
CCCTGGCTCGTCCACCCCCGCCACCGTTACCGCTGGAGAAGGAAAAATGGCATCTTCAACACCCGCCTCTCCCGCACCA
TCGGTTATACTGTCAAGAAAACCACAGTCAGAACGCCCTCCTGGAATGTGGACATGATGAGATTTAATATTAATGATT
TTCTTCCCCCAGGAGGGGGCTCAAACCCCCTCACTGTGCCCTTTGAATACTACAGAATAAGGAAGGTTAAGGTTGAAT
TCTGGCCCTGCTCCCCAATCACCCAGGGTGACAGGGGAGTGGGCTCCACTGCTGTTATTCTAGATGATAACTTTGTAA
CAAAGGCCAATGCCCTAACCTATGACCCCTATGTAAACTACTCCTCCCGCCATACCATAACCCAGCCCTTCTCCTACCAC
TCCCGGTACTTTACCCCGAAACCTGTCCTTGATAGGACAATCGATTACTTCCAACCCAATAACAAAAGAAATCAACTCT
GGCTGAGACTACAAACTACTGGAAATGTAGACCATGTAGGCCTCGGCACTGCGTTCGAAAACAGTATATACGACCAG
GACTACAATATCCGTATAACCATGTATGTACAATTCAGAGAATTTAATCTTAAAGACCCCCCACTTAACCCTAGG-----

>JQ809464_pcv2d

ATGACGTATCCAAGGAGGCGTTTCCGCAGACGAAGACACCGCCCCCGCAGCCATCTTGGCCAGATCCTCCGCCGCCGC
CCCTGGCTCGTCCACCCCCGCCACCGTTACCGCTGGAGAAGGAAAAATGGCATCTTCAACACCCGCCTCTCCCGCACCA
TCGGTTATACTGTCAAGAAAACCACAGTCAGAACGCCCTCCTGGAATGTGGACATGATGAGATTTAATATTAATGATT
TTCTTCCCCCAGGAGGGGGCTCAAACCCCCTCACTGTGCCCTTTGAATACTACAGAATAAGGAAGGTTAAGGTTGAAT
TCTGGCCCTGCTCCCCAATCACCCAGGGTGACAGGGGAGTGGGCTCCACTGCTGTTATTCTAGATGATAACTTTGTAA
CAAAGGCCAATGCCCTAACCTATGACCCCTATGTAAACTACTCCTCCCGCCATACCATAACCCAGCCCTTCTCCTACCAC
TCCCGGTACTTTACCCCGAAACCTGTCCTTGATAGGACAATCGATTACTTCCAACCCAATAACAAAAGAAATCAACTCT
GGCTGAGACTACAAACTACTGGAAATGTAGACCATGTAGGCCTCGGCACTGCGTTCGAAAACAGTATATACGACCAG
GACTACAATATCCGTATAACCATGTATGTACAATTCAGAGAATTTAATCTTAAAGACCCCCCACTTAACCCTAGG-----

>JF317586_pcv2d

ATGACGTATCCAAGGAGGCGTTTCCGCAGACGAAGACACCGCCCCCGCAGCCATCTTGGCCAGATCCTCCGCCGCCGC
CCCTGGCTCGTCCACCCCCGCCACCGTTACCGCTGGAGAAGGAAAAATGGCATCTTCAACACCCGCCTCTCCCGCACCA
TCGGTTATACTGTCAAGAAAACCACAGTCAGAACGCCCTCCTGGAATGTGGACATGATGAGATTTAATATTAATGATT
TTCTTCCCCCAGGAGGGGGCTCAAACCCCCTCACTGTGCCCTTTGAATACTACAGAATAAGGAAGGTGAAGGTTGAAT
TCTGGCCCTGCTCCCCCATCACCCAGGGTGACAGGGGAGTGGGCTCCACTGCTGTTATTCTAGATGATAACTTTGTAAC
AAAGGCCAATGCCCTAACCTATGACCCCTATGTAAACTACTCCTCCCGCCATACCATAACCCAGCCCTTCTCCTACCACT
CCCGGTACTTTACCCCGAAACCTGTCCTTGATAGGACAATCGATTACTTCCAACCCAATAACAAAAGAAATCAACTCTG
GCTGAGACTACAAACTACTGGAAATGTAGACCATGTAGGCCTCGGCACTGCGTTCGAAAACAGTATATACGACCAGG
ACTACAATATCCGTATAACCATGTATGTACAATTCAGAGAATTTAATCTTAAAGACCCCCCACTTAAACCC--------

>JX682407_pcv2d

ATGACGTATCCAAGGAGGCGTTTCCGCAGACGAAGACACCGCCCCCGCAGCCATCTTGGCCAGATCCTCCGCCGCCGC
CCCTGGCTCGTCCACCCCCGCCACCGTTACCGCTGGAGAAGGAAAAATGGCATCTTCAACACCCGCCTCTCCCGCACCA
TCGGTTATACTGTCAAGAAAACCACAGTCAGAACGCCCTCCTGGAATGTGGACATGATGAGATTTAATATTAATGATT
TTCTTCCCCCAGGAGGGGGCTCAAACCCCCTCACTGTGCCCTTTGAATACTACAGAATAAGAAAGGTTAAGGTTGAAT
TCTGGCCCTGCTCCCCAATCACCCAGGGTGACAGGGGAGTGGGCTCCACTGCTGTTATACTAGATGATAACTTTGTAA
CAAAGGCCAATGCCCTAACCTATGACCCCTATGTAAACTACTCCTCCCGCCATACCATAACCCAGCCCTTCTCCTACCAC
TCCCGGTACTTTACCCCGAAACCTGTCCTTGATAGGACAATCGATTACTTCCAACCCAATAACAAAAGAAATCAACTCT
GGCTGAGACTACAAACTACTGGAAATGTAGACCATGTAGGCCTCGGCACTGCGTTCGAAAACAGTATCTACGACCAG
GACTACAATATCCGTATAACCATGTATGTACAATTCAGAGAATTTAATCTTAAAGACCCCCCACTTAACCCTAAG-----

>HQ395038_pcv2d

ATGACGTATCCAAGGAGGCGTTTCCGCAGACGAAGACACCGCCCCCGCAGCCATCTTGGCCAGATCCTCCGCCGCCGC
CCCTGGCTCGTCCACCCCCGCCACCGTTACCGCTGGAGAAGGAAAAATGGCATCTTCAACACCCGCCTCTCCCGCACCA
TCGGTTATACTGTCAAGAAAACCACAGTCAGAACGCCCTCCTGGAATGTGGACATGATGAGATTTAATATTAATGATT
TTCTTCCCCCAGGAGGGGGCTCAAACCCCCTCACTGTGCCCTTTGAATACTACAGAATAAGGAAGGTTAAGGTTGAAT
TCTGGCCCTGCTCCCCAATCACCCAGGGTGACAGGGGAGTGGGCTCCACTGCTGTTATTCTAGATGATAACTTTGTAA
CAAAGGCCAATGCCCTAACCTATGACCCCTATGTAAACTACTCCTCCCGCCATACCATAACCCAGCCCTTCTCCTACCAC
TCCCGGTACTTTACCCCGAAACCTGTCCTTGATAGGACAATCGATTACTTCCAACCCAATAACAAAAGAAATCAACTCT
GGCTGAGACTACAAACTACTGGAAATGTAGACCATGTAGGCCTCGGCACTGCGTTCGAAAACAGTATCTACGACCAG
GACTACAATATCCGTATAACCATGTATGTACAATTCAGAGAATTTAATCTTAAAGACCCCCCACTTAACCCTAAG-----

>KC249977_pcv2d

ATGACGTATCCAAGGAGGCGTTTCCGCAGACGAAGACACCGCCCCCGCAGCCATCTTGGCCAGATCCTCCGCCGCCGC
CCCTGGCTCGTCCACCCCCGCCACCGTTACCGCTGGAGAAGGAAAAATGGCATCTTCAACACCCGCCTCTCCCGCACCA
TCGGTTATACTGTCAAGAAAACCACAGTCAGAACGCCCTCCTGGAATGTGGACATGATGAGATTTAATATTAATGATT
TTCTTCCCCCAGGAGGGGGCTCAAACCCCCTCACTGTGCCCTTTGAATACTACAGAATAAGGAAGGTTAAGGTTGAAT
TCTGGCCCTGCTCCCCAATCACCCAGGGTGACAGGGGAGTGGGCTCCACTGCTGTTATTCTAGATGATAACTTTGTAA
CAAAGGCCAATGCCCTAACCTATGACCCCTATGTAAACTACTCCTCCCGCCATACCATAACCCAGCCCTTCTCCTACCAC
TCCCGGTACTTTACCCCGAAACCTGTCCTTGATAGGACAATCGATTACTTCCAACCCAATAACAAAAGAAATCAACTCT
GGCTGAGACTACAAACTACTGGAAATGTAGACCATGTAGGCCTCGGCACTGCGTTCGAAAACAGTATCTACGACCAG
GACTACAATATCCGTATAACCATGTATGTACAATTCAGAGAATTTAATCTTAAAGACCCCCCACTTAACCCTAAG-----

>JN119255_pcv2d

ATGACGTATCCAAGGAGGCGTTTCCGCAGACGAAGACACCGCCCCCGCAGCCATCTTGGCCAGATCCTCCGCCGCCGC
CCCTGGCTCGTCCACCCCCGCCACCGTTACCGCTGGAGAAGGAAAAATGGCATCTTCAACACCCGCCTCTCCCGCACCA
TCGGTTATACTGTCAAGAAAACCACAGTCAGAACGCCCTCCTGGAATGTGGACATGATGAGATTTAATATTAATGATT
TTCTTCCCCCAGGAGGGGGCTCAAACCCCCTCACTGTGCCCTTTGAATACTACAGAATAAGGAAGGTTAAGGTTGAAT
TCTGGCCCTGCTCCCCAATCACCCAGGGTGACAGGGGAGTGGGCTCCACTGCTGTTATTCTAGATGATAACTTTGTAA
CAAAGGCCAATGCCCTAACCTATGACCCCTATGTAAACTACTCCTCCCGCCATACCATAACCCAGCCCTTCTCCTACCAC
TCCCGGTACTTTACCCCGAAACCTGTCCTTGATGGGACAATCGATTACTTCCAACCCAATAACAAAAGAAATCAACTCT
GGCTGAGACTACAAACTACTGGAAATGTAGACCATGTAGGCCTCGGCACTGCGTTCGAAAACAGTATATACGACCAG
GACTACAATATCCGTATAACCATGTATGTACAATTCAGAGAATTTAATCTTAAAGACCCCCCACTTAACCCTAAG-----

>JX912915_pcv2d

ATGACGTATCCAAGGAGGCGTTTCCGCAGACGAAGACACCGCCCCCGCAGCCATCTTGGCCAGATCCTCCGCCGCCGC
CCCTGGCTCGTCCACCCCCGCCACCGTTACCGCTGGAGAAGGAAAAATGGCATCTTCAACACCCGCCTCTCCCGCACCA
TCGGTTATACTGTCAAGAAAACCACAGTCAGAACGCCCTCCTGGAATGTGGACATGATGAGATTTAATATTAATGATT
TTCTTCCCCCAGGAGGGGGCTCAAACCCCCTCACTGTGCCCTTTGAATACTACAGAATAAGGAAGGTTAAGGTTGAAT
TCTGGCCCTGCTCCCCAATCACCCAGGGTGACAGGGGAGTGGGCTCCACTGCTGTTATTCTAGATGATAACTTTGTAA
CAAAGGCCAATGCCCTAACCTATGACCCCTATGTAAACTACTCCTCCCGCCATACCATAACCCAGCCCTTCTCCTACCAC
TCCCGGTACTTTACCCCGAAACCTGTCCTTGATGGGACAATCGATTACTTCCAACCCAATAACAAAAGAAATCAACTCT
GGCTGAGACTACAAACTACTGGAAATGTAGACCATGTAGGCCTCGGCACTGCGTTCGAAAACAGTATATACGACCAG
GACTACAATATCCGTATAACCATGTATGTACAATTCAGAGAATTTAATCTTAAAGACCCCCCACTTAACCCTAAG-----

>JN411099_pcv2d

ATGACGTATCCAAGGAGGCGTTTCCGCAGACGAAGACACCGCCCCCGCAGCCATCTTGGCCAGATCCTCCGCCGCCGC
CCCTGGCTCGTCCACCCCCGCCACCGTTACCGCTGGAGAAGGAAAAATGGCATCTTCAACACCCGCCTCTCCCGCACCA
TCGGTTATACTGTCAAGAAAACCACAGTCAGAACGCCCTCCTGGAATGTGGACATGATGAGATTTAATATTAATGATT
TTCTTCCCCCAGGAGGGGGCTCAAACCCCCTCACTGTGCCCTTTGAATACTACAGAATAAGGAAGGTTAAGGTTGAAT
TCTGGCCCTGCTCCCCAATCACCCAGGGTGACAGGGGAGTGGGCTCCACTGCTGTTATTCTAGATGATAACTTTGTAG
CAAAGGCCAATGCCCTAACCTATGACCCCTATGTAAACTACTCCTCCCGCCATACCATAACCCAGCCCTTCTCCTACCAC
TCCCGGTACTTTACCCCGAAACCTGTCCTTGATGGGACAATCGATTACTTCCAACCCAATAACAAAAGAAATCAACTCT
GGCTGAGACTACAAACTACTGGAAATGTAGACCATGTAGGCCTCGGCACTGCGTTCGAAAACAGTATATACGACCAG
GACTACAATATCCGTATAACCATGTATGTACAATTCAGAGAATTTAACCTTAAAGACCCCCCACTTAACCCTAAG-----

>JF683402_pcv2d

ATGACGTATTCAAGGAGGCGTTTCCGCAGACGAAGACACCGCCCCCGCAGCCATCTTGGCCAGATCCTCCGCCGCCGC
CCCTGGCTCGTCCACCCCCGCCACCGTTACCGCTGGAGAAGGAAAAATGGCATCTTCAACACCCGCCTCTCCCGCACCA
TCGGTTATACTGTCAAGAAAACCACAGTCAGAACGCCCTCCTGGAATGTGGACATGATGAGATTTAATATTAATGATT
TTCTTCCCCCAGGAGGGGGCTCAAACCCCCTCACTGTGCCCTTTGAATACTACAGAATAAGGAAGGTTAAGGTTGAAT
TCTGGCCCTGCTCCCCAATCACCCAGGGTGACAGGGGAGTGGGCTCCACTGCTGTTATTCTAGATGATAACTTTGTAG
CAAAGGCCAATGCCCTAACCTATGACCCCTATGTAAACTACTCCTCCCGCCATACCATAACCCAGCCCTTCTCCTACCAC
TCCCGGGACTTTACCCCGAAACCTGAACTTGATGGGACAATCGATTACTTCCAACCCAATAACAAAAGAAATCAACTCT
GGCTGAAACTACACACTACTGGAAATGTAGACCATGTAGGCCTCGGCACTGCGTTCGAAAACAGTATATACGACCAG
GACTACAATATCCGTATAACCATGTATGTACAATTCAGAGAATTTAATCTTAAAGACCCCCCACTTAACCCTAAG-----

>KC800646_pcv2d

ATGACGTATCCAAGGAGGCGTTTCCGCAGACGAAGACACCGCCCCCGCAGCCATCTTGGCCAGATCCTCCGCCGCCGC
CCCTGGCTCGTCCACCCCCGCCACCGTTACCGCTGGAGAAGGAAAAATGGCATCTTCAACACCCGCCTCTCCCGCACCA
TCGGTTATACTGTCAAGAAAACCACAGTCAGAACACCCTCCTGGAATGTGGACATGATGAGATTTAATATTAATGATTT
TCTTCCCCCAGGAGGGGGCTCAAACCCCCTCACTGTGCCCTTTGAATACTACAGAATAAGGAAGGTTAAGGTTGAATT
CTGGCCCTGCTCCCCAATCACCCAGGGTGACAGGGGAGTGGGCTCCACTGCTGTTATTCTAGATGATAACTTTGTAAC
AAAGGCCAATGCCCTAACCTATGACCCCTATGTAAACTACTCCTCCCGCCATACCATAACCCAGCCCTTCTCCTACCACT
CCCGGTACTTTACCCCGAAACCTGTCCTTGATGGGACAATCGATTACTTCCAACCCAATAACAAAAGAAATCAACTCTG
GCTGAGACTACAAACTACTGGAAATGTAGACCATGTAGGCCTGGGCACTGCGTTCGAAAACAGTAAATACGACCAGG
ACTACAATATCCGTGTAAGCGTGTATGGACAGGTGGGAGAATTTAGGGTGGAAGACCCCCCACTTAACCCT--------

>HM142896_pcv2d

ATGACGTATCCAAGGAGGCGTTTCCGCAGACGAAGACACCGCCCCCGCAGCCATCTTGGCCAGATCCTCCGCCGCCGC
CCCTGGCTCGTCCACCCCCGCCACCGTTACCGCTGGAGAAGGAAAAATGGCATCTTCAACACCCGCCTCTCCCGCACCA
TCGGTTATACTGTCAAGAAAACCACAGTCAGAACTCCCTCCTGGAATGTGGACATGATGAGATTTAATATTAATGATTT
TCTTCCCCCAGGAGGGGGCTCAAACCCCCTCACTGTGCCCTTTGAATACTACAGAATAAGGAAGGTTAAGGTTGAATT
CTGGCCCTGCTCCCCAATCACCCAGGGTGACAGGGGAGTGGGCTCCACTGCTGTTATTCTAGATGATAACTTTGTAAC
AAAGGCCAATGCCCTAACCTATGACCCCTATGTAAACTACTCCTCCCGCCATACCATAACACAGCCCTTCTCCTACCACT
CCCGGTACTTTACCCCGAAACCTGTCCTTGATGGGACAATCGATTACTTCCAACCCAATAACAAAAGAAATCAACTCTG
GCTGAGACTACAAACTACTGGAAATGTAGACCATGTAGGCCTCGGCACTGCGTTCGAAAACAGTATATACGACCAGG
ACTACAATATCCGTATAACCATGTATGTACAATTCAGAGAATTTAATCTTAAAGACCCCCCACTTAACCCTAAG-----

>HM161710_pcv2d

ATGACGTATCCAAGGAGGCGTTTCCGCAGACGAAGACACCGCCCCCGCAGCCATCTTGGCCAGATCCTCCGCCGCCGC
CCCTGGCTCGTCCACCCCCGCCACCGTTACCGCTGGAGAAGGAAAAATGGCATCTTCAACACCCGCCTCTCCCGCACCA
TCGGTTATACTGTCAAGAAAACCACAGTCAGAACTCCCTCCTGGAATGTGGACATGATGAGATTTAATATTAATGATTT
TCTTCCCCCAGGAGGGGGCTCAAACCCCCTCACTGTGCCCTTTGAATACTACAGAATAAGGAAGGTTAAGGTTGAATT
CTGGCCCTGCTCCCCAATCACCCAGGGTGACAGGGGAGTGGGCTCCACTGCTGTTATTCTAGATGATAACTTTGTAAC
AAAGGCCAATGCCCTAACCTATGACCCCTATGTAAACTACTCCTCCCGCCATACCATAACACAGCCCTTCTCCTACCACT
CCCGGTACTTTACCCCGAAACCTGTCCTTGATGGGACAATCGATTACTTCCAACCCAATAACAAAAGAAATCAACTCTG
GCTGAGACTACAAACTACTGGAAATGTAGACCATGTAGGCCTCGGCACTGCGTTCGAAAACAGTATATACGACCAGG
ACTACAATATCCGTATAACCATGTATGTACAATTCAGAGAATTTAATCTTAAAGACCCCCCACTTAACCCTAAG-----

>KC800643_pcv2d

ATGACGTATCCAAGGAGGCGTTTCCGCAGACGAAGACACCGCCCCCGCAGCCATCTTGGCCAGATCCTCCGCCGCCGC
CCCTGGCTCGTCCACCCCCGCCACCGTTACCGCTGGAGAAGGAAAAATGGCATCTTCAACACCCGCCTCTCCCGCACCA
TCGGTTATACTGTCAAGAAAACCACAGTCAGAACGCCCTCCTGGAATGTGGACATGATGAGATTTAATATTAATGATT
TTCTTCCCCCAGGAGGGGGCTCAAACCCCCTCACTGTGCCCTTTGAATACTACAGAATAAGGAAGGTTAAGGTTGAAT
TCTGGCCCTGCTCCCCAATCACCCAGGGTGACAGGGGAGTGGGCTCCACTGCTGTTATTCTAGATGATAACTTTGTAA
CAAAGGCCAATGCCCTAACCTATGACCCCTATGTAAACTACTCCTCCCGCCATACCATAACCCAGCCCTTCTCCTACCAC
TCCCGGTACTTTACCCCGAAACCTGTCCTTGATAGTACAATCGATTACTTCCAACCCAATAACAAAAGAAATCAACTCT
GGCTGAGACTACAAACTACTGGAAATGTAGACCATGTAGGCCTCGGCACTGCGTTCGAAAACAGTATATACGACCAG
GACTACAATATCCGTATAACCATGTATGTACAATTCAGAGAATTTAATCTTAAAGACCCCCCACTTAACCCT--------

>JF928004_pcv2d

ATGACGTATCCAAGGAGGCGTTTCCGCAGACGAAGACACCGCCCCCGCAGCCATCTTGGCCAGATCCTCCGCCGCCGC
CCCTGGCTCGTCCACCCCCGCCACCGTTACCGCTGGAGAAGGAAAAATGGCATCTTCAACACCCGCCTCTCCCGCACCA
TCGGTTATACTGTCAAGAAAACCACAGTCAGAACGCCCTCCTGGAATGTGGACATGATGAGATTTAATATTAATGATT
TTCTTCCCCCAGGAGGGGGCTCAAACCCCCTCACTGTGCCCTTTGAATACTACAGAATAAGGAAAGTTAAGGTTGAAT
TCTGGCCCTGCTCCCCAATCACGCAGGGTGACAGGGGAGTGGGCTCCACTGCTGTTATTCTAGATGATAACTTTGTAA
CAAAGGCCAATGCCCTAACCTATGACCCCTATGTAAACTACTCCTCCCGCCATACCATAACCCAGCCCTTCTCCTACCAC
TCCCGATACTTTACCCCGAAACCTGTCCTTGATAGGACAATCGATTACTTCCAACCCAATAACAAAAGAAATCAACTCT
GGCTGAGACTACAAACTACTGGAAATGTAGACCATGTAGGCCTCGGCACTGCGTTCGAAAACAGTATATACGACCAG
GACTACAATATCCGTATAACCATGTATGTACAATTCAGAGAATTTAATCTTAAAGACCCCCCACTTAACCCTAAG-----

>JF928006_pcv2d

ATGACGTATCCAAGGAGGCGTTTCCGCAGACGAAGACACCGCCCCCGCAGCCATCTTGGCCAGATCCTCCGCCGCCGC
CCCTGGCTCGTCCACCCCCGCCACCGTTACCGCTGGAGAAGGAAAAATGGCATCTTCAACACCCGCCTCTCCCGCACCA
TCGGTTATACTGTCAAGAAAACCACAGTCAGAACGCCCTCCTGGAATGTGGACATGATGAGATTTAATATTAATGATT
TTCTTCCCCCAGGAGGGGGCTCAAACCCCCTCACTGTGCCCTTTGAATACTACAGAATAAGGAAAGTTAAGGTTGAAT
TCTGGCCCTGCTCCCCAATCACGCAGGGTGACAGGGGAGTGGGCTCCACTGCTGTTATTCTAGATGATAACTTTGTAA
CAAAGGCCAATGCCCTAACCTATGACCCCTATGTAAACTACTCCTCCCGCCATACCATAACCCAGCCCTTCTCCTACCAC
TCCCGATACTTTACCCCGAAACCTGTCCTTGATAGGACAATCGATTACTTCCAACCCAATAACAAAAGAAATCAACTCT
GGCTGAGACTACAAACTACTGGAAATGTAGACCATGTAGGCCTCGGCACTGCGTTCGAAAACAGTATATACGACCAG
GACTACAATATCCGTATAACCATGTATGTACAATTCAGAGAATTTAATCTTAAAGACCCCCCACTTAACCCTAAG-----

>HQ395055_pcv2d

ATGACGTATCCAAGGAGGCGTTTCCGCAGACGAAGACACCGCCCCCGCAGCCATCTTGGCCAGATCCTCCGCCGCCGC
CCCTGGCTCGTCCACCCCCGCCACCGTTACCGCTGGAGAAGGAAAAATGGCATCTTCAACACCCGCCTCTCCCGCACCA
TCGGTTATACTGTCAAGAAAACCACAGTCAGAACGCCCTCCTGGAATGTGGACATGATGAGATTTAATATTAATGATT
TTCTTCCCCCGGGAGGGGGCTCAAACCCCCTCACTGTGCCCTTTGAATACTACAGAATAAGGAAGGTTAAGGTTGAAT
TCTGGCCCTGCTCCCCAATCACCCAGGGTGACAGGGGAGTGGGCTCCACTGCTGTTATTCTAGATGATAACTTTGTAA
CAAAGGCAAATGCCCTAACCTATGACCCCTATGTAAACTACTCCTCCCGCCATACCATAACCCAGCCCTTCTCCTACCAC
TCCCGATACTTTACCCCGAAACCTGTCCTTGATAGGACAATCGATTACTTCCAACCCAATAACAAAAGAAATCAACTCT
GGCTGAGACTACAAACTACTGGAAATGTAGACCATGTAGGCCTCGGCACTGCGTTCGAAAACAGTATATACGACCAG
GACTACAATATCCGTATAACCATGTATGTACAATTCAGAGAATTTAATCTTAAAGACCCCCCACTTAACCCTAAG-----

>JX982224_pcv2d

ATGACGTATCCAAGGAGGCGTTTCCGCAGACGAAGACACCGCCCCCGCAGCCATCTTGGCCAGATCCTCCGCCGCCGC
CCCTGGCTCGTCCACCCCCGCCACCGTTACCGCTGGAGAAGGAAAAATGGCATCTTCAACACCCGCCTCTCCCGCACCA
TCGGTTATACTGTCAAGAAAACCACAGTCAGAACGCCCTCCTGGAATGTGGACATGATGAGATTTAATATTAATGATT
TTCTTCCCCCAGGAGGGGGCTCAAACCCCCTCACTGTGCCCTTTGAATACTACAGAATAAGGAAGGTTAAGGTTGAAT
TCTGGCCCTGCTCCCCAATCACCCAGGGTGACAGGGGAGTGGGCTCCACTGCTGTTATTCTAGATGATAACTTTGTAA
CAAAGGCCAATGCCCTAACCTATGACCCCTATGTAAACTACTCCTCCCGCCATACCATAACCCAGCCCTTCTCCTACCAC
TCCCGATACTTTACCCCGAAACCTGTCCTTGATAGGACAATCGATTACTTCCAACCCAATAACAAAAGAAATCAACTCT
GGCTGAGACTACAAACTACTGGAAATGTAGACCATGTAGGCCTCGGCACTGCGTTCGAAAACAGTATATACGACCAG
GACTACAATATCCGTATAACCATGTATGTACAATTCAGAGAATTTAATCTTAAAGACCCCCCACTTAACCCTAAG-----

>HQ650833_pcv2d

ATGACGTATCCAAGGAGGCGTTTCCGCAGACGAAGACACCGCCCCCGCAGTCATCTTGGCCAGATCCTCCGCCGCCGC
CCCTGGCTCGTCCACCCCCGCCACCGTTACCGCTGGAGAAGGAAAAATGGCATCTTCAACACCCGCCTCTCCCGCACCA
TCGGTTATACTGTCAAGAAAACCACAGTCAGAACGCCCTCCTGGAATGTGGACATGATGAGATTTAATATTAATGATT
TTCTTCCCCCAGGAGGGGGCTCAAACCCCCTCACTGTGCCCTTTGAATACTACAGAATAAGGAAGGTTAAGGTTGAAT
TCTGGCCCTGCTCCCCAATCACCCAGGGTGACAGGGGAGTGGGCTCCACTGCTGTTATTCTAGATGATAACTTTGTAA
CAAAGGCCAATGCCCTAACCTATGACCCCTATGTAAACTACTCCTCCCGCCATACCATAACCCAGCCCTTCTCCTACCAC
TCCCGATACTTTACCCCGAAACCTGTCCTTGATGGGACAATCGATTACTTCCAACCCAATAACAAAAGAAATCAACTCT
GGCTGAGACTACAAACTACTGGAAATGTAGACCATGTAGGCCTCGGCACTGCGTTCGAAAACAGTATATACGACCAG
GACTACAATATCCGTATAACCATGTATGTACAATTCAGAGAATTTAATCTTAAAGACCCCCCACTTAACCCTAAG-----

>JX406422_pcv2d

ATGACGTATCCAAGGAGGCGTTTCCGCAGACGAAGACACCGCCCCCGCAGCCATCTTGGCCAGATCCTCCGCCGCCGC
CCCTGGCTCGTCCACCCCCGCCACCGTTACCGCTGGAGAAGGAAAAATGGCATCTTCAACACCCGCCTCTCCCGCACCA
TCGGTTATACTGTCAAGAAAACCACAGTCAGAACGCCCTCCTGGAATGTGGACATGATGAGATTTAATATTAATGATT
TTCTTCCCCCAGGAGGGGGCTCAAACCCCCTCACTGTGCCCTTTGAATACTACAGAATAAGAAAGGTTAAGGTTGAAT
TCTGGCCCTGCTCCCCAATCACCCAGGGTGACAGGGGAGTGGGCTCCACTGCTGTTATTCTAGATGATAACTTTGTAA
CAAAGGCCAATGCCCTAACCTATGACCCCTATGTAAACTACTCCTCCCGCCATACCATAACCCAGCCCTTCTCCTACCAC
TCCCGATACTTTACACCGAAACCTGTCCTTGATAGGACAATCGATTACTTCCAACCCAATAACAAAAGAAATCAACTCT
GGCTGAGACTACAAACTACTGGAAATGTAGACCATGTAGGCCTCGGCACTGCGTTCGAAAACAGTATATACGACCAG
GACTACAATATCCGTATAACCATGTATGTACAATTTAGAGAATTTAATCTTAAAGACCCCCCACTTAACCCTAAG-----

>HQ395059_pcv2d

ATGACGTATCCAAGGAGGCGTTTCCGCAGACGAAGACACCGCCCCCGCAGCCATCTTGGCCAGATCCTCCGCCGCCGC
CCCTGGCTCGTCCACCCCCGCCACCGTTACCGCTGGAGAAGGAAAAATGGCATCTTCAACACCCGCCTCTCCCGCACCA
TCGGTTATACTGTCAAGAAAACCACAGTCAGAACGCCCTCCTGGAATGTGGACATGATGAGATTTAATATTAATGATT
TTCTTCCCCCAGGAGGGGGCTCAAACCCCCTCACTGTGCCCTTTGAATACTACAGAATAAGGAAGGTTAAGGTTGAAT
TCTGGCCCTGCTCCCCAATCACCCAGGGTGACAGGGGAGTGGGCTCCACTGCTGTTATTCTAGATGATAACTTTGTAA
CAAAGGCCAATGCCCTAACCTATGACCCCTATGTAAACTACTCCTCCCGCCATACCATAACCCAGCCCTTCTCCTACCAC
TCCCGATACTTTACCCCGAAACCTGTCCTTGATAGGACAATCGATTACTTCCAACCCAATAACAAAAGAAATCAACTCT
GGCTGAGACTACAAACTACTGGAAATGTAGACCATGTAGGCCTCGGCACTGCGTTCGAAAACAGTATATACGACCAG
GACTACAATATCCGTATAACCATGTATGTACAATTTAGAGAATTTAATCTTAAAGACCCCCCACTTAACCCTAAG-----

>HQ395053_pcv2d

ATGACGTATCCAAGGAGGCGTTTCCGCAGACGAAGACACCGCCCCCGCAGCCATCTTGGCCAGATCCTCCGCCGCCGC
CCCTGGCTCGTCCACCCCCGCCACCGTTACCGCTGGAGAAGGAAAAATGGCATCTTCAACACCCGCCTCTCCCGCACCA
TCGGTTATACTGTCAAGAAAACCACAGTCAGAACGCCCTCCTGGAATGTGGACATGATGAGATTTAATATTAATGATT
TTCTTCCCCCAGGAGGGGGCTCAAACCCCCTCACTGTGCCCTTTGAATACTACAGAATAAGGAAGGTTAAGGTTGAAT
TCTGGCCCTGCTCCCCAATCACCCAGGGTGACAGGGGAGTGGGCTCCACTGCTGTTATTCTAGATGATAACTTTGTAA
CAAAGGCCAATGCCCTAACCTATGACCCCTATGTAAACTACTCCTCCCGCCATACCATAACCCAGCCCTTCTCCTACCAC
TCCCGATACTTTACCCCAAAACCTGTCCTTGATAGGACAATCGATTACTTCCAACCCAATAACAAAAGAAATCAACTCT
GGCTGAGACTACAAACTACTGGAAATGTAGACCATGTAGGCCTCGGCACTGCGTTCGAAAACAGTATATACGACCAG
GACTACAATATCCGTATAACCATGTATGTACAATTCAGAGAATTTAATCTTAAAGACCCCCCACTTAACCCTAAG-----

>HQ395021_pcv2d

ATGACGTATCCAAGGAGGCGTTTCCGCAGACGAAGACACCGCCCCCGCAGCCATCTTGGCCAGATCCTCCGCCGCCGC
CCCTGGCTCGTCCACCCCCGCCACCGTTACCGCTGGAGAAGGAAAAATGGCATCTTCAACACCCGCCTCTCCCGCACCA
TCGGTTATACTGTCAAGAAAACCACAGTCAGAACGCCCTCCTGGAATGTGGACATGATGAGATTTAATATTAATGATT
TTCTTCCCCCAGGAGGGGGCTCAAACCCCCTCACTGTGCCCTTTGAATACTACAGAATAAGGAAGGTTAAGGTTGAAT
TCTGGCCCTGCTCCCCAATCACCCAGGGTGACAGGGGAGTGGGCTCCACTGCTGTTATTCTAGATGATAACTTTGTAA
CAAAGGCCAATGCCCTAACCTATGACCCCTATGTAAACTACTCCTCCCGCCATACCATAACCCAGCCCTTCTCCTACCAC
TCCCGGTACTTTACCCCAAAACCTGTCCTTGATAGGACAATCGATTACTTCCAACCCAATAACAAAAGAAATCAACTCT
GGCTGAGACTACAAACTACTGGAAATGTAGACCATGTGGGCCTCGGCACTGCGTTCGAAAACAGTATATACGACCAG
GACTACAATATCCGTATAACCATGTATGTACAATTCAGAGAATTTAATCTTAAAGACCCCCCACTTAACCCTAAG-----

>JQ653449_pcv2d

ATGACGTATCCAAGGAGGCGTTTCCGCAGACGAAGACACCGCCCCCGCAGCCATCTTGGCCAGATCCTCCGCCGCCGC
CCCTGGCTCGTCCACCCCCGCCTCCGTTACCGCTGGAGAAGGAAAAATGGCATCTTCAACACCCGCCTCTCCCGCACCA
TCGGTTATACTGTCAAGAAAACCACAGTCAGAACGCCCTCCTGGAATGTGGACATGATGAGATTTAATATTAATGATT
TTCTTCCCCCAGGAGGGGGCTCAAACCCCCTCACTGTGCCCTTTGAATACTACAGAATAAGGAAGGTTAAGGTTGAAT
TCTGGCCCTGCTCCCCAATCACCCAGGGTGACAGGGGAGTGGGCTCCACTGCTGTTATTCTAGATGATAACTTTGTAA
CAAAGGCCAATGCCCTAACCTATGACCCCTATGTAAACTACTCCTCCCGCCATACCATAACCCAGCCCTTCTCCTACCAC
TCCCGGTACTTTACCCCAAAACCTGTCCTTGATAGGACAATCGATTACTTCCAACCCAATAACAAAAGAAATCAACTCT
GGCTGAGACTACAAACTACTGGAAATGTAGACCATGTAGGCCTCGGCACTGCGTTCGAAAACAGTATATACGACCAG
GACTACAATATCCGTATAACCATGTATGTACAATTCAGAGAATTTAATCTTAAAGACCCCCCACTTAACCCTAAG-----

>HM776441_pcv2d

ATGACGTATCCAAGGAGGCGTTTCCGCAGACGAAGACACCGCCCCCGCAGCCATCTTGGCCAGATCCTCCGCCGCCGC
CCCTGGCTCGTCCACCCCCGCCACCGTTACCGCTGGAGAAGGAAAAATGGCATCTTCAACACCCGCCTCTCCCGCACCA
TCGGTTATACTGTCAAGAAAACCACAGTCAGAACGCCCTCCTGGAATGTGGACATGATGAGATTTAATATTAATGATT
TTCTTCCCCCAGGAGGGGGCTCAAACCCCCTCACTGTGCCCTTTGAATACTACAGAATAAGGAAGGTTAAGGTTGAAT
TCTGGCCCTGCTCCCCAATCACACAGGGTGACAGGGGAGTGGGCTCCACTGCTGTTATTCTAGATGATAACTTTGTAA
CAAAGGCCAATGCCCTAACCTATGACCCCTATGTAAACTACTCCTCCCGCCATACCATAACCCAGCCCTTCTCCTACCAC
TCCCGGTACTTTACCCCAAAACCTGTCCTTGATAGGACAATCGATTACTTCCAACCCAATAACAAAAGAAATCAACTCT
GGCTGAGACTACAAACTACTGGAAATGTAGACCATGTAGGCCTCGGCACTGCGTTCGAAAACAGTATATACGACCAG
GACTACAATATCCGTATAACCATGTATGTACAATTCAGAGAATTTAATCTTAAAGACCCCCCACTTAACCCTAAG-----

>JQ413808_pcv2d

ATGACGTATCCAAGGAGGCGTTTCCGCAGACGAAGACACCGCCCCCGCAGCCATCTTGGCCAGATCCTCCGCCGCCGC
CCCTGGCTCGTCCACCCCCGCCACCGTTACCGCTGGAGAAGGAAAAATGGCATCTTCAACACCCGCCTCTCCCGCACCA
TCGGTTATACTGTCAAGAAAACCACAGTCAGAACGCCCTCCTGGAATGTGGACATGATGAGATTTAATATTAATGATT
TTCTTCCCCCAGGAGGGGGCTCAAACCCCCTCACTGTGCCCTTTGAATACTACAGAATAAGGAAGGTTAAGGTTGAAT
TCTGGCCCTGCTCCCCAATCACACAGGGTGACAGGGGAGTGGGCTCCACTGCTGTTATTCTAGATGATAACTTTGTAA
CAAAGGCCAATGCCCTAACCTATGACCCCTATGTAAACTACTCCTCCCGCCATACCATAACCCAGCCCTTCTCCTACCAC
TCCCGGTACTTTACCCCGAAACCTGTCCTTGATAGGACAATCGATTACTTCCAACCCAATAACAAAAGAAATCAACTCT
GGCTGAGACTACAAACTACTGGAAATGTAGACCATGTAGGCCTCGGCACTGCGTTCGAAAACAGTATATACGACCAG
GACTACAATATCCGTATAACCATGTATGTACAATTCAGAGAATTTAATCTTAAAGACCCCCCACTTAACCCTAAG-----

>HQ395024_pcv2d

ATGACGTATCCAAGGAGGCGTTTCCGCAGACGAAGACACCGCCCCCGCAGCCATCTTGGCCAGATCCTCCGCCGCCGC
CCCTGGCTCGTCCACCCCCGCCACCGTTACCGCTGGAGAAGGAAAAATGGCATCTTCAACACCCGCCTCTCCCGCACCA
TCGGTTATACTGTCAAGAAAACCACAGTCAGAACGCCCTCCTGGAATGTGGACATGATGAGATTTAATATTAATGATT
TTCTTCCCCCAGGAGGGGGCTCAAACCCCCTCACTGTGCCCTTTGAATACTACAGAATAAGGAAGGTTAAGGTTGAAT
TCTGGCCCTGCTCCCCAATCACACAGGGTGACAGGGGAGTGGGCTCCACTGCTGTTATTCTAGATGATAACTTTGTAA
CAAAGGCCAATGCCCTAACCTATGACCCCTATGTAAACTACTCCTCCCGCCATACCATAACCCAGCCCTTCTCCTACCAC
TCCCGGTACTTTACCCCGAAACCTGTCCTTGATAGGACAATCGATTACTTCCAACCCAATAACAAAAGAAATCAACTCT
GGCTGAGACTACAAACTACTGGAAATGTAGACCATGTAGGCCTCGGCACTGCATTCGAAAACAGTATATACGACCAG
GACTACAATATCCGTATAACCATGTATGTACAATTCAGAGAATTTAATCTTAAAGACCCCCCACTTAACCCTAAG-----

>GQ359011_pcv2d

ATGACGTATCCAAGGAGGCGTTTCCGCAGACGAAGACACCGCCCCCGCAGCCATCTTGGCCAGATCCTCCGCCGCCGC
CCCTGGCTCGTCCACCCCCGCCACCGTTACCGCTGGAGAAGGAAAAATGGCATCTTCAACACCCGCCTCTCCCGCACCA
TCGGTTATACTGTCAAGAAAACCACAGTCAGAACGCCCTCCTGGAATGTGGACATGATGAGATTTAATATTAATGATT
TTCTTCCCCCAGGAGGGGGCTCAAACCCCCTCACTGTGCCCTTTGAATACTACAGAATAAGGAAGGTTAAGGTTGAAT
TCTGGCCCTGCTCCCCAATCACACAGGGTGACAGGGGAGTGGGCTCCACTGCTGTTATTCTAGATGATAACTTTGTAA
CAAAGGCCAATGCCCTAACCTATGACCCTTATGTAAACTACTCCTCCCGCCATACCATAACCCAGCCCTTCTCCTACCAC
CCCCGGTACTTTACCCCGAAACCTGTCCTTGATAGGACAATCGATTACTTCCAACCCAATAACAAAAGAAATCAACTCT
GGCTGAGACTACAAACTACTGGAAATGTAGACCATGTAGGCCTCGGCACTGCGTCCGAAAACAGTATATACGACCAG
GACTACAATATCCGTATAACCATGTATGTACAATTCAGAGAATTTAATCTTAAAGACCCCCCACTTAACCCTAAG-----

>KC533811_pcv2d

ATGACGTATCCAAGGAGGCGTTTCCGCAGACGAAGACACCGCCCCCGCAGCCATCTTGGCCAGATCCTCCGCCGCCGC
CCCTGGCTCGTCCACCCCCGCCACCGTTACCGCTGGAGAAGGAAAAATGGCATCTTCAACACCCGCCTCTCCCGCACCA
TCGGTTATACTGTCAAGAAAACCACAGTCAGAACGCCCTCCTGGAATGTGGACATGATGAGATTTAATATTAATGATT
TTCTTCCCCCAGGAGGGGGCTCAAACCCCCTCACTGTGCCGTTTGAATACTACAGAATAAGGAAGGTTAAGGTTGAAT
TCTGGCCCTGCTCCCCAATCACACAGGGTGGCAGGGGAGTGGGCTCCACTGCTGTTATTCTAGATGATAACTTTGTAA
CAAAGGCCAATGCCCTAACCTATGACCCCTATGTAAACTACTCCTCCCGCCATACCATAACCCAGCCCTTCTCCTACCAC
TCCCGGTACTTCACCCCGAAACCTGTCCTTGATAGGACAATCGATTACTTCCAACCCAATAACAAAAGAAATCAACTCT
GGCTGAGACTACAAACTACTGGAAATGTAGACCATGTAGGCCTCGGCACTGCGTTCGAAAACAGTATATACGACCAG
GACTACAATATCCGTATAACCATGTATGTACAATTCAGAGAATTTAATCTTAAAGACCCCCCACTTAACCCTAAG-----

>JX406421_pcv2d

ATGACGTATCCAAGGAGGCGTTTCCGCAGACGAAGACACCGCCCCCGCAGCCATCTTGGCCAGATCCTCCGCCGCCGC
CCCTGGCTCGTCCACCCCCGCCACCGTTACCGCTGGAGAAGGAAAAATGGCATCTTCAACACCCGCCTCTCCCGCACCA
TCGGTTATACTGTCAAGAAAACCACAGTCAGAACGCCCTCCTGGAATGTGGACATGATGAGATTTAATATTAATGATT
TTCTTCCCCCAGGAGGGGGCTCAAACCCCCTCACTGTGCCCTTTGAATACTACAGAATAAGGAAGGTTAAGGTTGAAT
TCTGGCCCTGCTCCCCAATCACACAGGGTGACAGGGGAGTGGGCTCCACTGCTGTTATTCTAGATGATAACTTTGTAA
CAAAGGCCAATGCCCTAACCTATGACCCCTATGTAAACTACTCCTCCCGCCATACCATAACCCAGCCCTTCTCCTACCAC
TCCCGGTACTTCACCCCGAAACCTGTCCTTGATAGGACAATCGATTACTTCCAACCCAATAACAAAAGAAATCAACTCT
GGCTGAGACTACAAACTACTGGAAATGTAGACCATGTAGGCCTCGGCACTGCGTTCGAAAACAGTATATACGACCAG
GACTACAATATCCGTATAACCATGTATGTACAATTCAGAGAATTTAATCTTAAAGACCCCCCACTTAACCCTAAG-----

>JX982228_pcv2d

ATGACGTATCCAAGGAGGCGTTTCCGCAGACGAAGACACCGCCCCCGCAGCCATCTTGGCCAGATCCTCCGCCGCCGC
CCCTGGCTAGTCCACCCCCGCCACCGTTACCGCTGGAGAAGGAAAAATGGCATCTTCAACACCCGCCTCTCCCGCACC
ATCGGTTATACTGTCAAGAAAACCACAGTCAGAACGCCCTCCTGGAATGTGGACATGATGAGATTTAATATTAATGAT
TTTCTTCCCCCAGGAGGGGGCTCAAACCCCCTCACTGTGCCCTTTGAATACTACAGAATAAGAAAGGTTAAGGTTGAA
TTCTGGCCCTGCTCCCCAATCACCCAGGGTGACAGGGGAGTGGGCTCCACTGCTGTTATTCTAGATGATAACTTTGTAA
CAAAGGCCAATGCCCTAACCTATGACCCCTATGTAAACTACTCCTCCCGCCATACCATAACCCAGCCCTTCTCCTACCAC
TCCCGGTACTTTACCCCGAAACCTGTCCTTGATAGGACAATCGATTACTTCCAACCCAATAACAAAAGAAATCAACTCT
GGCTGAGACTACAAACTACTGGAAATGTAGACCATGTAGGCCTCGGCACTGCGTTCGAAAACAGTATATACGACCAG
GACTACAATATCCGTATAACCATGTATGTACAATTCAGAGAATTTAATCTTAAAGACCCCCCACTTAACCCTAAG-----

>JX948770_pcv2d

ATGACGTATCCAAGGAGGCGTTTCCGCAGACGAAGACACCGCCCCCGCAGCCATCTTGGCCAGATCCTCCGCCGCCGC
CCCTGGCTAGTCCACCCCCGCCACCGTTACCGCTGGAGAAGGAAAAATGGCATCTTCAACACCCGCCTCTCCCGCACC
ATCGGTTATACTGTCAAGAAAACCACAGTCAGAACGCCCTCCTGGAATGTGGACATGATGAGATTTAATATTAATGAT
TTTCTTCCCCCAGGAGGGGGCTCAAACCCCCTCACTGTGCCCTTTGAATACTACAGAATAAGGAAGGTTAAGGTTGAA
TTCTGGCCCTGCTCCCCAATCACCCAGGGTGACAGGGGAGTGGGCTCCACTGCTGTTATTCTAGATGATAACTTTGTAA
CAAAGGCCAATGCCCTAACCTATGACCCCTATGTAAACTACTCCTCCCGCCATACCATAACCCAGCCCTTCTCCTACCAC
TCCCGGTACTTTACCCCGAAACCTGTCCTTGATACGACAATCGATTACTTCCAACCCAATAACAAAAGAAATCAACTCT
GGCTGAGACTACAAACTACTGGAAATGTAGACCATGTAGGCCTCGGCACTGCGTTCGAAAACAGTATATACGACCAG
GACTACAATATCCGTATAACCATGTATGTACAATTCAGAGAATTTAATCTTAAAGACCCCCCACTTAACCCTAAG-----

>JX948776_pcv2d

ATGACGTATCCAAGGAGGCGTTTCCGCAGACGAAGACACCGCCCCCGCAGCCATCTTGGCCAGATCCTCCGCCGCCGC
CCCTGGCTAGTCCACCCCCGCCACCGTTACCGCTGGAGAAGGAAAAATGGCATCTTCAACACCCGCCTCTCCCGCACC
ATCGGTTATACTGTCAAGAAAACCACAGTCAGAACGCCCTCCTGGAATGTGGACATGATGAGATTTAATATTAATGAT
TTTCTTCCCCCAGGAGGGGGCTCAAACCCCCTCACTGTGCCCTTTGAATACTACAGAATAAGGAAGGTTAAGGTTGAA
TTCTGGCCCTGCTCCCCAATCACCCAGGGTGACAGGGGAGTGGGCTCCACTGCTGTTATTCTAGATGATAACTTTGTAA
CAAAGGCCAATGCCCTAACCTATGACCCCTATGTAAACTACTCCTCCCGCCATACCATAACCCAGCCCTTCTCCTACCAC
TCCCGGTACTTTACCCCGAAACCTGTCCTTGATACGACAATCGATTACTTCCAACCCAATAACAAAAGAAATCAACTCT
GGCTGAGACTACAAACTACTGGAAATGTAGACCATGTAGGCCTCGGCACTGCGTTCGAAAACAGTATATACGACCAG
GACTACAATATCCGTATAACCATGTATGTACAATTCAGAGAATTTAATCTTAAAGACCCCCCACTTAACCCTAAG-----

>JQ390467_pcv2d

ATGACGTATCCAAGGAGGCGTTACCGCAGACGAAGACACCGCCCCCGCAGCCATCTTGGCCAGATCCTCCGCCGCCG
CCCCTGGCTAGTCCACCCCCGCCACCGTTACCGCTGGAGAAGGAAAAATGGCATCTTCAACACCCGCCTCTCCCGCACC
ATCGGTTATACTGTCAAGAAAACCACAGTCAGAACGCCCTCCTGGAATGTGGACATGATGAGATTTAATATTAATGAT
TTTCTTCCCCCAGGAGGGGGCTCAAGCCCCCTCACTGTGCCCTTTGAATACTACAGAATAAGGAAGGTTAAGGTTGAA
TTCTGGCCCTGCTCCCCAATCACCCAGGGTGACAGGGGAGTGAGCTCCACTGCTGTTATTCTAGATGATAACTTTGTAA
CAAAGGCCAATGCCCTAACCTATGACCCCTATGTAAACTACTCCTCCCGCCATACCATAACCCAGCCCTTCTCCTACCAC
TCCCGGTACTTTACCCCGAAACCTGTCCTTGATAGGACAATCGATTACTTCCAACCCAATAACAAAAGAAATCAACTCT
GGCTGAGACTACAAACTACTGGAAATGTAGACCATGTAGGCCTCGGCACTGCGTTCGAAAACAGTATATACGACCAG
GACTACAATATCCGTATAACCATGTATGTACAATTCAGAGAATTTAATCTTAAAGACCCCCCACTTAACCCTAAG-----

>JX948782_pcv2d

ATGACGTATCCAAGGAGGCGTTTCCGCAGACGAAGACACCGCCCCCGCAGCCATCTTGGCCAGATCCTCCGCCGCCGC
CCCTGGCTAGTCCACCCCCGCCACCGTTACCGCTGGAGAAGGAAAAATGGCATCTTCAACACCCGCCTCTCCCGCACC
ATCGGTTATACTGTCAAGAAAACCACAGTCAGAACGCCCTCCTGGAATGTGGACATGATGAGATTTAATATTAATGAT
TTTCTTCCCCCAGGAGGGGGCTCAAACCCCCTCACTGTGCCCTTTGAATACTACAGAATAAGGAAGGTTAAGGTTGAA
TTCTGGCCCTGCTCCCCAATCACCCAGGGTGACAGGGGAGTGGGCTCCACTGCTGTTATTCTAGATGATAACTTTGTAA
CAAAGGCCAATGCCCTAACCTATGACCCCTATGTAAACTACTCCTCCCGCCATACCATAACCCAGCCCTTCTCCTACCAC
TCCCGGTACTTTACCCCGAAACCTGTCCTTGATAGGACAATCGATTACTTCCAACCCAATAACAAAAGAAATCAACTCT
GGCTGAGACTACAAACTACTGGAAATGTAGACCATGTAGGCCTCGGCACTGCGTTCGAAAACAGTATATACGACCAG
GACTACAATATCCGTATAACCATGTATGTACAATTCAGAGAATTTAATCTTAAAGACCCCCCACTTAACCCTAAG-----

>JX948771_pcv2d

ATGACGTATCCAAGGAGGCGTTTCCGCAGACGAAGACACCGCCCCCGCAGCCATCTTGGCCAGATCCTCCGCCGCCGC
CCCTGGCTAGTCCACCCCCGCCACCGTTACCGCTGGAGAAGGAAAAATGGCATCTTCAACACCCGCCTCTCCCGCACC
ATCGGTTATACTGTCAAGAAAACCACAGTCAGAACGCCCTCCTGGAATGTGGACATGATGAGATTTAATATTAATGAT
TTTCTTCCCCCAGGAGGGGGCTCAAACCCCCTCACTGTGCCCTTTGAATACTACAGAATAAGGAAGGTTAAGGTTGAA
TTCTGGCCCTGCTCCCCAATCACCCAGGGTGACAGGGGAGTGGGCTCCACTGCTGTTATTCTAGATGATAACTTTGTAA
CAAAGGCCAATGCCCTAACCTATGACCCCTATGTAAACTACTCCTCCCGCCATACCATAACCCAGCCCTTCTCCTACCAC
TCCCGGTACTTTACCCCGAAACCTGTCCTTGATAGGACAATCGATTACTTCCAACCCAATAACAAAAGAAATCAACTCT
GGCTGAGACTACAAACTACTGGAAATGTAGACCATGTAGGCCTCGGCACTGCGTTCGAAAACAGTATATACGACCAG
GACTACAATATCCGTATAACCATGTATGTACAATTCAGAGAATTTAATCTTAAAGACCCCCCACTTAACCCTAAG-----

>JX406420_pcv2d

ATGACGTATCCAAGGAGGCGTTTCCGCAGACGAAGACACCGCCCCCGCAGCCATCTTGGCCAGATCCTCCGCCGCCGC
CCCTGGCTAGTCCACCCCCGCCACCGTTACCGCTGGAGAAGGAAAAATGGCATCTTCAACACCCGCCTCTCCCGCACC
ATCGGTTATACTGTCAAGAAAACCACAGTCAGAACGCCCTCCTGGAATGTGGACATGATGAGATTTAATATTAATGAT
TTTCTTCCCCCAGGAGGGGGCTCAAACCCCCTCACTGTGCCCTTTGAATACTACAGAATAAGGAAGGTTAAGGTTGAA
TTCTGGCCCTGCTCCCCAATCACCCAGGGTGACAGGGGAGTGGGCTCCACTGCTGTTATTCTAGATGATAACTTTGTAA
CAAAGGCCAATGCCCTAACCTATGACCCCTATGTAAACTACTCCTCCCGCCATACCATAACCCAGCCCTTCTCCTACCAC
TCCCGGTACTTTACCCCGAAACCTGTCCTTGATAGGACAATCGATTACTTCCAACCCAATAACAAAAGAAATCAACTCT
GGCTGAGACTACAAACTACTGGAAATGTAGACCATGTAGGCCTCGGCACTGCGTTCGAAAACAGTATATACGACCAG
GACTACAATATCCGTATAACCATGTATGTACAATTCAGAGAATTTAATCTTAAAGACCCCCCACTTAACCCTAAG-----

>FJ712215_pcv2d

ATGACGTATCCAAGGAGGCGTTTCCGCAGACGAAGACACCGCCCCCGCAGCCATCTTGGCCAGATCCTCCGCCGCCGC
CCCTGGCTCGTCCACCCCCGCCACCGTTACCGCTGGAGAAGGAAAAATGGCATCTTCAACGCCCGCCTCTCCCGCACC
ATCGGTTATACTGTCAAGAAAACCACAGTCAGAACGCCCTCCTGGAATGTGGACATGATGAGATTTAATATTAATGAT
TTTCTTCCCCCAGGAGGGGGCTCAAACCCCCTCACTGTGCCCTTTGAATACTACAGAATAAGGAAGGTTAAGGTTGAA
TTCTGGCCCTGCTCCCCAATCACCCGGGGTGACAGGGGAGTGGGCTCCACTGCTGTTATTCTAGATGATAACTTTGTA
ACAAAGGCCAATGCCCTAACCTATGACCCCTATGTAAACTACTCCTCCCGCCATACCATAACCCAGCCCTTCTCCTACCA
CTCCCGGTACTTTACCCCGAAACCTGTCCTTGATAGGACAATCGATTACTTCCAACCCAATAACAAAAGAAATCAACTC
TGGCTGAGACTACAAACTACTGGAAATGTAGACCACGTAGGCCTCGGCACTGCGTTCGAAAACAGTATATACGACCA
GGACTACAATATCCGTATAACCATGTATGTACAATTCAGAGAATTTAATCTTAAAGACCCCCCACTTAACCCTAAG-----

>JX948786_pcv2d

ATGACGTATCCAAGGAGGCGTTTCCGCAGACGAAGACACCGCCCCCGCAGCCATCTTGGCCAGATCCTCCGCCGCCGC
CCCTGGCTCGTCCACCCCCGCCACCGTTACCGCTGGAGAAGGAAAAATGGCATCTTCAACACCCGCCTCTCCCGCACCA
TCGGTTATACTGTCAAGAAAACCACAGTCAGAACGCCCTCCTGGAATGTGGACATGATGAGATTTAATATTAATGATT
TTCTTCCCCCAGGAGGGGGCTCAAACCCCCTCACTGTGCCCTTTGAATACTACAGAATAAGGAAGGTTAAAGTTGAAT
TCTGGCCCTGCTCCCCAATCACCCAGGGTGACAGGGGAGTGGGCTCCACTGCTGTTATTCTAGATGATAACTTTGTAA
CAAAGGCCAATGCCCTAACCTATGACCCCTATGTAAACTACTCCTCCCGCCATACCATAACCCAGCCCTTCTCCTACCAC
TCCCGGTACTTTACCCCCAAACCTGTCCTTGATAGGACAATCGATTACTTCCAACCCAATAACAAAAGAAATCAACTCT
GGCTGAGACTACAAACTACTGGAAATGTAGACCATGTAGGCCTCGGCACTGCGTTCGAAAACAGTATATACGACCAG
GACTACAATATCCGTATAACCATGTATGTACAATTCAGAGAATTTAATCTTAAAGACCCCCCACTTAACCCTAAG-----

>HQ395042_pcv2d

ATGACGTATCCAAGGAGGCGTTTCCGCAGACGAAGACACCGCCCCCGCAGCCATCTTGGCCAGATCCTCCGCCGCCGC
CCCTGGCTCGTCCACCCCCGCCACCGTTACCGCTGGAGAAGGAAAAATGGCATCTTCAACACCCGCCTCTCCCGCACCA
TCGGTTATACTGTCAAGAAAACCACAGTCAGAACGCCCTCCTGGAATGTGGACATGATGAGATTTAATATTAACGACT
TTCTTCCCCCAGGAGGGGGCTCAAACCCCCTCACTGTGCCCTTTGAATACTACAGAATAAGGAAGGTTAAGGTTGAAT
TCTGGCCCTGCTCCCCAATCACCCAGGGTGACAGGGGAGTGGGCTCCACTGCTGTTATTCTAGATGATAACTTTGTAA
CAAAGGCCAATGCCCTAACCTATGACCCCTATGTAAACTACTCCTCCCGCCATACCATAACCCAGCCCTTCTCCTACCAC
TCCCGGTACTTTACCCCGAAACCTGTCCTTGATAGGACAATCGATTACTTCCAACCCAATAACAAAAGAAATCAACTCT
GGCTGAGACTACAAACTACTGGAAATGTAGACCATGTAGGCCTCGGCACTGCGTTCGAAAACAGTATATACGACCAG
GACTACAATATCCGTATAACCATGTATGTACAATTCAGAGAATTTAATCTTAAAGACCCCCCACTTAACCCTAAG-----

>KC800640_pcv2d

ATGACGTATCCAAGGAGGCGTTTCCGCAGACGAAGACACCGCCCCCGCAGCCATCTTGGCCAGATCCTCCGCCGCCGC
CCCTGGCTCGTCCACCCCCGCCACCGTTACCGCTGGAGAAGGAAAAATGGCATCTTCAACACCCGCCTCTCCCGCACCA
TCGGATATACTGTCAAGAAAACCACAGTCAGAACGCCCTCCTGGAATGTGGACATGATGAGATTTAATATTAATGATT
TTCTTCCCCCAGGAGGGGGCTCAAACCCCCTCACTGTGCCCTTTGAATACTACAGAATAAGGAAGGTTAAGGTTGAAT
TCTGGCCCTGCTCCCCAATCACCCAGGGTGACAGGGGAGTGGGCTCCACTGCTGTTATTCTAGATGATAACTTTGTAA
CAAAGGCCAATGCCCTAACCTATGACCCCTATGTAAACTACTCCTCCCGCCATACCATAACCCAGCCCTTCTCCTACCAC
TCCCGGTACTTTACCCCGAAACCTGTCCTTGATAGGACAATCGATTACTTCCAACCCAATAACAAAAGAAATCAACTCT
GGCTGAGACTACAAACTACTGGAAATGTAGACCATGTAGGCCTCGGCACTGCGTTCGAAAACAGTATATACGACCAG
GACTACAATATCCGTATAACCATGTATGTACAATTCAGAGAATTTAATCTTAAAGACCCCCCACTTAACCCT--------

>JX982227_pcv2d

ATGACGTATCCAAGGAGGCGTTTCCGCAGACGAAGACACCGCCCCCGCAGCCATCTTGGCCAGATCCTCCGCCGCCGC
CCCTGGCTCGTCCACCCCCGCCACCGTTACCGCTGGAGAAGGAAAAATGGCATCTTCAACACCCGCCTCTCCCGCACCA
TCGGTTATACTGTCAAGAAAACCACAGTCAGAACGCCCTCCTGGAATGTGGACATGATGAGATTTAATATTAATGATT
TTCTTCCCCCAGGAGGGGGCTCAAACCCCCTCACTGTGCCCTTTGAATACTACAGAATAAGAAAGGTTAAGGTTGAAT
TCTGGCCCTGCTCCCCAATCACCCAGGGTGACAGGGGAGTGGGCTCCACTGCTGTTATTCTAGATGATAACTTTGTAA
CAAAGGCCAATGCCCTAACCTATGACCCCTATGTAAACTACTCCTCCCGCCATACCATAACCCAGCCCTTCTCCTACCAC
TCCCGGTACTTTACCCCGAAACCTGTCCTTGATGGGACAATCGATTACTTCCAACCCAATAACAAAAGAAATCAACTCT
GGCTGAGACTACAAACTACTGGAAATGTAGACCATGTAGGCCTCGGCACTGCGTTCGAAAACAGTATATACGACCAG
GACTACAATATCCGTATAACCATGTATGTACAATTCAGAGAATTTAATCTTAAAGACCCCCCACTTAACCCTAAG-----

>GU325762_pcv2d

ATGACGTATCCAAGGAGGCGTTTCCGCAGACGAAGACACCGCCCCCGCAGCCATCTTGGCCAGATCCTCCGCCGCCGC
CCCTGGCTCGTCCACCCCCGCCACCGTTACCGCTGGAGAAGGAAAAATGGCATCTTCAACACCCGCCTCTCCCGCACCA
TCGGTTATACTGTCAAGAAAACCACAGTCAGAACGCCCTCCTGGAATGTGGACATGATGAGATTTAATATTAATGATT
TTCTTCCCCCAGGAGGGGGCTCAAACCCCCTCACTGTGCCCTTTGAATACTACAGAATAAGAAAGGTTAAGGTTGAAT
TCTGGCCCTGCTCCCCAATCACCCAGGGTGACAGGGGAGTGGGCTCCACTGCTGTTATTCTAGATGATAACTTTGTAA
CAAAGGCCAATGCCCTAACCTATGACCCCTATGTAAACTACTCCTCCCGCCATACCATAACCCAGCCCTTCTCCTACCAC
TCCCGGTACTTTACCCCGAAACCTGTCCTTGATAGGACAATCGATTACTTCCAACCCAATAACAAAAGAAATCAACTCT
GGCTGAGACTACAAACTACTGGAAATGTAGACCATGTAGGCCTCGGCACTGCGTTCGAAAACAGTATATACGACCAG
GACTACAATATCCGTATAACCATGTATGTACAATTCAGAGAATTTAATCTTAAAGACCCCCCACTTAACCCTAAG-----

>KC153106_pcv2d

ATGACGTATCCAAGGAGGCGTTTCCGCAGACGAAGACACCGCCCCCGCAGCCATCTTGGCCAGATCCTCCGCCGCCGC
CCCTGGCTCGTCCACCCCCGCCACCGTTACCGCTGGAGAAGGAAAAATGGCATCTTCAACACCCGCCTCTCCCGCACCA
TCGGTTATACTGTCAAGAAAACCACAGTCAGAACGCCCTCCTGGAATGTGGACATGATGAGATTTAATATTAATGATT
TTCTTCCCCCAGGAGGGGGCTCAAACCCCCTCACTGTGCCCTTTGAATACTACAGAATAAGAAAGGTTAAGGTTGAAT
TCTGGCCCTGCTCCCCAATCACCCAGGGTGACAGGGGAGTGGGCTCCACTGCTGTTATTCTAGATGATAACTTTGTAA
CAAAGGCCAATGCCCTAACCTATGACCCCTATGTAAACTACTCCTCCCGCCATACCATAACCCAGCCCTTCTCCTACCAC
TCCCGGTACTTTACCCCGAAACCTGTCCTTGATAGGACAATCGATTACTTCCAACCCAATAACAAAAGAAATCAACTCT
GGCTGAGACTACAAACTACTGGAAATGTAGACCATGTAGGCCTCGGCACTGCGTTCGAAAACAGTATATACGACCAG
GACTACAATATCCGTATAACCATGTATGTACAATTCAGAGAATTTAATCTTAAAGACCCCCCACTTAACCCTAAG-----

>GU325763_pcv2d

ATGACGTATCCAAGGAGGCGTTTCCGCAGACGAAGACACCGCCCCCGCAGCCATCTTGGCCAGATCCTCCGCCGCCGC
CCCTGGCTCGTCCACCCCCGCCACCGTTACCGCTGGAGAAGGAAAAATGGCATCTTCAACACCCGCCTCTCCCGCACCA
TCGGTTATACTGTCAAGAAAACCACAGTCAGAACGCCCTCCTGGAATGTGGACATGATGAGATTTAATATTAATGATT
TTCTTCCCCCAGGAGGGGGCTCAAACCCCCTCACTGTGCCCTTTGAATACTACAGAATAAGAAAGGTTAAGGTTGAAT
TCTGGCCCTGCTCCCCAATCACCCAGGGTGACAGGGGAGTGGGCTCCACTGCTGTTATTCTAGATGATAACTTTGTAA
CAAAGGCCAATGCCCTAACCTATGACCCCTATGTAAACTACTCCTCCCGCCATACCATAACCCAGCCCTTCTCCTACCAC
TCCCGGTACTTTACCCCGAAACCTGTCCTTGATAGGACAATCGATTACTTCCAACCCAATAACAAAAGAAATCAACTCT
GGCTGAGACTACAAACTACTGGAAATGTAGACCATGTAGGCCTCGGCACTGCGTTCGAAAACAGTATATACGACCAG
GACTACAATATCCGTATAACCATGTATGTACAATTCAGAGAATTTAATCTTAAAGACCCCCCACTTAACCCTAAG-----

>FJ644929_pcv2d

ATGACGTATCCAAGGAGGCGTTTCCGCAGACGAAGACACCGCCCCCGCAGCCATCTTGGCCAGATCCTCCGCCGCCGC
CCCTGGCTCGTCCACCCCCGCCACCGTTACCGCTGGAGAAGGAAAAATGGCATCTTCAACACCCGCCTCTCCCGCACCA
TCGGTTATACTGTCAAGAAAACCACAGTCAGAACGCCCTCCTGGAATGTGGACATGATGAGATTTAATATTAATGATT
TTCTTCCCCCAGGAGGGGGCTCAAACCCCCTCACTGTGCCCTTTGAATACTACAGAATAAGAAAGGTTAAGGTTGAAT
TCTGGCCCTGCTCCCCAATCACCCAGGGTGACAGGGGAGTGGGCTCCACTGCTGTTATTCTAGATGATAACTTTGTAA
CAAAGGCCAATGCCCTAACCTATGACCCCTATGTAAACTACTCCTCCCGCCATACCATAACCCAGCCCTTCTCCTACCAC
TCCCGGTACTTTACCCCGAAACCTGTCCTTGATAGGACAATCGATTACTTCCAACCCAATAACAAAAGAAATCAACTCT
GGCTGAGACTACAAACTACTGGAAATGTAGACCATGTAGGCCTCGGCACTGCGTTCGAAAACAGTATATACGACCAG
GACTACAATATCCGTATAACCATGTATGTACAATTCAGAGAATTTAATCTTAAAGACCCCCCACTTAACCCTAAG-----

>GU325768_pcv2d

ATGACGTATCCAAGGAGGCGTTTCCGCAGACGAAGACACCGCCCCCGCAGCCATCTTGGCCAGATCCTCCGCCGCCGC
CCCTGGCTCGTCCACCCCCGCCACCGTTACCGCTGGAGAAGGAAAAATGGCATCTTCAACACCCGCCTCTCCCGCACCA
TCGGTTATACTGTCAAGAAAACCACAGTCAGAACGCCCTCCTGGAATGTAGACATGATGAGATTTAATATTAATGATTT
TCTTCCCCCAGGAGGGGGCTCAAACCCCCTCACTGTGCCCTTTGAATACTACAGAATAAGAAAGGTTAAGGTTGAATT
CTGGCCCTGCTCCCCAATCACCCAGGGTGACAGGGGAGTGGGCTCCACTGCTGTTATTCTAGATGATAACTTTGTAAC
AAAGGCCAATGCCCTAACCTATGACCCCTATGTAAACTACTCCTCCCGCCATACCATAACCCAGCCCTTCTCCTACCACT
CCCGGTACTTTACCCCGAAACCTGTCCTTGATAGGACAATCGATTACTTCCAACCCAATAACAAAAGAAATCAACTCTG
GCTGAGACTACAAACTACTGGAAATGTAGACCATGTAGGCCTCGGCACTGCGTTCGAAAACAGTATATACGACCAGG
ACTACAATATCCGTATAACCATGTATGTACAATTCAGAGAATTTAATCTTAAAGACCCCCCACTTAACCCTAAG-----

>GU325767_pcv2d

ATGACGTATCCAAGGAGGCGTTTCCGCAGACGAAGACACCGCCCCCGCAGCCATCTTGGCCAGATCCTCCGCCGCCGC
CCCTGGCTCGTCCACCCCCGCCACCGTCACCGCTGGAGAAGGAAAAATGGCATCTTCAACACCCGCCTCTCCCGCACC
ATCGGTTATACTGTCAAGAAAACCACAGTCAGAACGCCCTCCTGGAATGTGGACATGATGAGATTTAATATTAATGAT
TTTCTTCCCCCAGGAGGGGGCTCAAACCCCCTCACTGTGCCCTTTGAATACTACAGAATAAGAAAGGTTAAGGTTGAA
TTCTGGCCCTGCTCCCCAATCACCCAGGGTGACAGGGGAGTGGGCTCCACTGCTGTTATTCTAGATGATAACTTTGTAA
CAAAGGCCAATGCCCTAACCTATGACCCCTATGTAAACTACTCCTCCCGCCATACCATAACCCAGCCCTTCTCCTACCAC
TCCCGGTACTTTACCCCGAAACCTGTCCTTGATAGGACAATCGATTACTTCCAACCCAATAACAAAAGAAATCAACTCT
GGCTGAGACTACAAACTACTGGAAATGTAGACCATGTAGGCCTCGGCACTGCCTTCGAAAACAGTATATACGACCAG
GACTACAATATCCGTATAACCATGTATGTACAATTCAGAGAATTTAATCTTAAAGACCCCCCACTTAACCCTAAG-----

>GU124593_pcv2d

ATGACGTATCCAAGGAGGCGTTTCCGCAGACGAAGACACCGCCCCCGCAGCCATCTTGGCCAGATCCTCCGCCGCCGC
CCCTGGCTCGTCCACCCCCGCCACCGTTACCGCTGGAGAAGGAAAAATGGCATCTTCAACACCCGCCTCTCCCGCACCA
TCGGTTATACTGTCAAGAAAACCACAGTCAGAACGCCCTCCTGGAATGTGGACATGATGAGATTTAATATTAATGATT
TTCTTCCCCCAGGAGGGGGCTCAAACCCCCTCACTGTGCCCTTTGAATACTACAGAATAAGAAAGGTTAAGGTTGAAT
TCTGGCCCTGCTCCCCAATCACCCAGGGTGACAGGGGAGTGGGCTCCACTGCTGTTATTCTAGATGATAACTTTGTAA
CAAAGGCCAATGCCCTAACCTATGACCCCTATGTAAACTACTCCTCCCGCCATACCATAACCCAGCCCTTCTCCTACCAC
TCCCGGTACTTTACCCCGAAACCTGTCCTTGATAGGACAATCGATTACTTCCAACCCAATAACAAAAGAAATCAACTCT
GGCTGAGACTACAAACTACTGGAAATGTAGACCATGTAGGCCTCGGCACTGCGTTCGAAAACAGTATATACGACCAG
GACTATAATATCCGTATAACCATGTATGTACAATTCAGAGAATTTAATTTTAAAGACCCCCCACTTAACCCTAAG-----

>JX982220_pcv2d

ATGACGTATCCAAGGAGGCGTTTCCGCAGACGAAGACACCGCCCCCGCAGCCATCTTGGCCAGATCCTCCGCCGCCGC
CCCTGGCTCGTCCACCCCCGCCACCGTTACCGCTGGAGAAGGAAAAATGGCATCTTCAACACCCGCCTCTCCCGCACCA
TCGGTTATACTGTCAAGAAAACCACAGTCAGAACGCCCTCCTGGAATGTGGACATGATGAGATTTAATATTAATGATT
TCCTTCCCCCAGGAGGGGGCTCAAACCCCCTCACTGTGCCCTTTGAATACTACAGAATAAGAAAGGTTAAGGTTGAAT
TCTGGCCCTGCTCCCCAATCACCCAGGGTGACAGGGGAGTGGGCTCCACTGCTGTTATTCTAGATGATAACTTTGTAA
CAAAGGCCAATGCCCTAACCTATGACCCCTATGTAAACTACTCCTCCCGCCATACCATAACCCAGCCCTTCTCCTACCAC
TCCCGGTACTTTACCCCGAAACCTGTCCTTGATAGGACAATCGATTACTTCCAACCCAATACCAAAAGAAATCAACTCT
GGCTGAGACTACAAACTACTGGAAATGTAGACCATGTAGGCCTCGGCACTGCGTTCGAAAACAGTATATACGACCAG
GACTACAATATCCGTATAACCATGTATGTACAATTCAGAGAATTTAATCTTAAAGACCCCCCACTTAACCCTAAG-----

>KC447454_pcv2d

ATGACGTATCCAAGGAGGCGTTTCCGCAGACGAAGACACCGCCCCCGCAGCCATCTTGGCCAGATCCTCCGCCGCCGC
CCCTGGCTCGTCCACCCCCGCCACCGTTACCGCTGGAGAAGGAAAAATGGCATCTTCAACACCCGCCTCTCCCGCACCA
TCGGTTATACTGTCAAGAAAACCACAGTCAGAACGCCCTCCTGGAATGTGGACATGATGAGATTTAATATTAATGATT
TTCTTCCCCCAGGAGGGGGCTCAAACCCCCTCACTGTGCCCTTTGAATACTACAGAATAAGAAAGGTTAAGGTTGAAT
TCTGGCCCTGCTCCCCAATCACCCAGGGTGACAGGGGAGTGGGTTCCACTGCTGTTATTCTAGATGATAACTTTGTAAC
AAAGGCCAATGCCCTAACCTATGACCCCTATGTAAACTACTCCTCCCGCCATACCATAACCCAGCCCTTCTCCTACCACT
CCCGGTACTTTACCCCGAAACCTGTCCTTGATAGGACAATCGATTACTTCCAACCCAATAACAAAAGAAATCAACTCTG
GCTGAGACTACAAACTACTGGAAATGTAGACCATGTAGGCCTCGGCACTGCGTTCGAAAACAGTATATACGACCAAG
ACTACAATATCCGTATAACCATGTATGTACAATTCAGAGAATTTAATCTTAAAGACCCCCCACTTAACCCTAAG-----

>GU325770_pcv2d

ATGACGTATCCAAGGAGGCGTTTCCGCAGACGAAGACACCGCCCCCGCAGCCATCTTGGCCAGATCCTCCGCCGCCGC
CCCTGGCTCGTCCACCCCCGCCACCGTTACCGCTGGAGAAGGAAAAATGGCATCTTCAACACCCGCCTCTCCCGCACCA
TCGGTTATACTGTCAAGAAAACCACAGTAAGAACGCCCTCCTGGAATGTGGACATGATGAGATTTAATATTAATGATT
TTCTTCCCCCAGGAGGGGGCTCAAACCCCCTCACTGTGCCCTTTGAATACTACAGAATAAGAAAGGTTAAGGTTGAAT
TCTGGCCCTGCTCCCCAATCACCCAGGGTGACAGGGGAGTGGGCTCCACTGCTGTTATTCTAGATGATAACTTTGTAA
CAAAGGCCAATGCCCTAACCTATGACCCCTATGTAAACTACTCCTCCCGCCATACCATAACCCAGCCCTTCTCCTACCAC
TCCCGGTACTTTACCCCGAAACCTGTCCTTGATAGGACAATCGATTACTTCCAACCCAATAACAAAAGAAATCAACTCT
GGCTGAGACTACAAACTACTGGAAATGTAGACCATGTAGGCCTCGGCACTGCGTTCGAAAACAGTATATACGACCAG
GACTACAATATCCGTATAACCATGTATGTACAATTCAGAGAATTTAATCTTAAAGACCCACCACTTAACCCTAAG-----

>GU325760_pcv2d

ATGACGTATCCAAGGAGGCGTTTCCGCAGACGAAGACGCCGCCCCCGCAGCCATCTTGGCCAGATCCTCCGCCGCCG
CCCCTGGCTCGTCCACCCCCGCCACCGTTACCGCTGGAGAAGGAAAAATGGCATCTTCAACACCCGCCTCTCCCGCACC
ATCGGTTATACTGTCAAGAAAACCACAGTCAGAACGCCCTCCTGGAATGTGGACATGATGAGATTTAATATTAATGAT
TTTCTTCCCCCAGGAGGGGGCTCAAACCCCCTCACTGTGCCCTTTGAATACTACAGAATAAGAAAGGTTAAGGTTGAA
TTCTGGCCCTGCTCCCCAATCACCCAGGGTGACAGGGGAGTGGGCTCCACTGCTGTTATTCTAGATGATAACTTTGTAA
CAAAGGCCAATGCCCTAACCTATGACCCCTATGTAAACTACTCCTCCCGCCATACCATAACCCAGCCCTTCTCCTACCAC
TCCCGGTACTTTACCCCGAAACCTGTCCTTGATAGGACTATCGATTACTTCCAACCCAATAACAAAAGAAATCAACTCT
GGCTGAGACTACAAACTACTGGAAATGTAGACCATGTAGGCCTCGGCACTGCGTTCGAAAACAGTATATACGACCAG
GACTACAATATCCGTATAACCATGTATGTACAATTCAGAGAATTTAATCTTAAAGACCCCCCACTTAACCCTAAG-----

>FJ712216_pcv2d

ATGACGTATCCAAGGAGGCGTTTCCGCAGACGAAGACACCGCCCCCGCAGCCATCTTGGCCAGATCCTCCGCCGCCGC
CCCTGGCTCGTCCACCCCCGCCACCGTTACCGCTGGAGAAGGAAAAATGGCATCTTCAACACCCGCCTCTCCCGCACCA
TCGGTTATACTGTCAAAAAAACCACAGTCAGAACGCCCTCCTGGAATGTGGACATGATGAGATTTAATATTAATGATTT
TCTTCCCCCAGGAGGGGGCTCAAACCCCCTCACTGTGCCCTTTGAATACTACAGAATAAGAAAGGTTAAGGTTGAATT
CTGGCCCTGCTCCCCAATCACCCAGGGTGACAGGGGAGTGGGCTCCACTGCTGTTATTCTAGATGATAACTTTGTAAC
AAAGGCCAATGCCCTAACCTATGACCCCTATGTAAACTACTCCTCCCGCCATACCATAACCCAGCCCTTCTCCTACCACT
CCCGGTACTTTACCCCGAAACCTGTCCTTGATAGGACAATCGATTACTTCCAACCAAATAACAAAAGAAATCAACTCTG
GCTGAGACTACAAACTACTGGAAATGTAGACCATGTAGGCCTCGGCACTGCGTTCGAAAACAGTATATACGACCAGG
ACTACAATATCCGTATAACCATGTATGTACAATTCAGAGAATTTAATCTTAAAGACCCCCCACTTAACCCTAAG-----

>JX948775_pcv2d

ATGACGTATCCAAGGAGGCGTTTCCGCAGACGAAGACACCGCCCCCGCAGCCATCTTGGCCAGATCCTCCGCCGCCGC
CCCTGGCTCGTCCACCCCCGCCACCGTTACCGCTGGAGAAGGAAAAATGGCATCTTCAACACCCGCCTCTCCCGCACCA
TCGGTTATACTGTCAAGAAAACCACAGTCAGAACGCCCTCCTGGAATGTGGACATGATGAGATTTAATATTAATGATT
TTCTTCCCCCAGGAGGGGGCTCAAACCCCCTCACTGTGCCCTTTGAATACTACAGAATAAGAAAGGTTAAGGTTGAAT
TCTGGCCCTGCTCCCCAATCACCCAGGGTGACAGGGGAGTGGGCTCCACTGCTGTTATTCTAGATGATAACTTTGTAA
CAAAGGCCAATGCCCTAACCTATGACCCCTATGTAAACTACTCCTCCCGCCATACCATAACCCAGCCCTTCTCCTACCAC
TCCCGGTACTTTACCCCCAAACCTGTCCTTGATAGGACAATCGATTACTTCCAACCCAATAACAAAAGAAATCAACTCT
GGCTGAGACTACAAACTACTGGAAATGTAGACCATGTAGGCCTCGGCACTGCGTTCGAAAACAGTATATACGACCAG
GACTACAATATCCGTATAACCATGTATGTACAATTCAGAGAATTTAATCTTAAAGACCCCCCACTTAACCCTAAG-----

>KC800637_pcv2d

ATGACGTATCCAAGGAGGCGTTTCCGCAGACGAAGACACCGCCCCCGCAGCCATCTTGGCCAGATCCTCCGCCGCCGC
CCCTGGCTCGTCCACCCCCGCCACCGTTACCGCTGGAGAAGGAAAAATGGCATCTTCAACACCCGCCTCTCCCGCACCA
TCGGATATACTGTCAAGAAAACCACAGTCAGAACGCCCTCCTGGAATGTGGACATGATGAGATTTAATATTAATGATT
TTCTTCCCCCAGGAGGGGGCTCAAACCCCCTCACTGTGCCCTTTGAATACTACAGAATAAGGAAGGTTAAGGTTGAAT
TCTGGCCCTGCTCCCCAATCACCCAGGGTGACAGGGGAGTGGGCTCCACTGCTGTTATTCTAGATGATAACTTTGTAA
CAAAGGCCAATGCCCTAACCTATGACCCCTATGTAAACTACTCCTCCCGCCATACCATAACCCAGCCCTTCTCCTACCAC
TCCCGGTACTTTACCCCGAAACCTGTCCTTGATAGCACAATCGATTACTTCCAACCCAATAACAAAAGAAATCAACTCT
GGCTGAGACTACAAACTACTGGAAATGTAGACCATGTAGGCCTCGGCACTGCGTTCGAAAACAGTATATACGACCAG
GACTACAATATCCGTATAACCATGTATGTACAATTCAGAGAATTTAATCTTAAAGACCCCCCACTTAACCCT--------

>KC800638_pcv2d

ATGACGTATCCAAGGAGGCGTTTCCGCAGACGAAGACACCGCCCCCGCAGCCATCTTGGCCAGATCCTCCGCCGCCGC
CCCTGGCTCGTCCACCCCCGCCACCGTTACCGCTGGAGAAGGAAAAATGGCATCTTCAACACCCGCCTCTCCCGCACCA
TCGGTTATACTGTCAAGAAAACCACAGTCAGAACGCCCTCCTGGGCGGTGGACATGTGGAGATTTAATATTAATGATT
TTCTTCCCCCAGGAGGGGGCTCAAACCCCCTCACTGTGCCCTTTGAATACTACAGAATAAGGAAGGTTAAGGTTGAAT
TCTGGCCCTGCTCCCCAATCACCCAGGGTGACAGGGGAGTGGGCTCCACTGCTGTTATTCTAGATGATAACTTTGTAA
CAAAGGCCAATGCCCTAACCTATGACCCCTATGTAAACTACTCCTCCCGCCATACCATAACCCAGCCCTTCTCCTACCAC
TCCCGGTACTTTACCCCGAAACCTGTCCTTGATAGGACAATCGATTACTTCCAACCCAATAACAAAAGAAATCAACTCT
GGCTGAGACTACAAACTACTGGAAATGTAGACCATGTAGGCCTCGGCACTGCGTTCGAAAACAGTATATACGACCAG
GACTACAATATCCGTATAACCATGTATGTACAATTCAGAGAATTTAATCTTAAAGACCCCCCACTTAACCCT--------

>JX982226_pcv2d

ATGACGTATCAAAGGAGGCGTTTCCGCAGACGAAGACACCGCCCCCGCAGCCATCTTGGCCAGATCCTCCGCCGCCG
CCCCTGGCTCGTCCACCCCCGCCACCGTTACCGCTGGAGAAGGAAAAATGGCATCTTCAACACCCGCCTCTCCCGCACC
ATCGGTTATACTGTCAAGAAAACCACAGTCAAAACGCCCTCCTGGACGGTGGACATGATGAGATTTAATATTAATGAT
TTTCTTCCCCCAGGAGGGGGCTCAAACCCCCTCATGGGGCCCTTTGAATACTACAGAATAAGAAAGGTTAAGGTTGAA
TTCTGGCCCTGCTCCCCAATCACCCAGGGTGACAGGGGAGTGGGCTCCACTGCTGTTATTCTAGATGATAACTTTGTAA
CAAAGGCCAATGCCCTAACCTATGACCCCTATGTAAACTACTCCTCCCGCCATACCATAACCCAGCCCTTCTCCTACCAC
TCCCGGTACTTTACCCCGAAACCTGTCCTTGATAGGACAATCGATTACTTCCAACCCAATAACAAAAGAAATCAACTCT
GGCTTAGACTACAAACTACTGGGAATGTAGACCATGTAGGCCTCGGCACTGCGTTCGAAAACAGTATATACGACCAG
GACTACAATATCCGTATAACCATGTATGTACAATTCAGAGAATTTAATCTTAAAGACCCCCCACTTAACCCTAAG-----

>JN382188_pcv2d

ATGACGTATCCAAGGAGGCGTTACCGGAGAAGAAGACACCGCCCCCGCAGCCATCTTGGCCAGATCCTCCGCCGCCG
CCCCTGGCTCGTCCACCCCCGCCACCGTTACCGCTGGAGAAGGAAAAATGGCATCTTCAACACCCGCCTCTCCCGCACC
ATCGGTTATACTGTCAAGAAAACCACAGTCAGAACGCCCTCCTGGAATGTGGACATGATGAGATTTAATATTAATGAT
TTTCTTCCCCCAGGAGGGGGCTCAAACCCCCTCACTGTGCCCTTTGAATACTACAGAATAAGGAAGGTTAAGGTTGAA
TTCTGGCCCTGCTCCCCAATCACCCAGGGTGACAGGGGAGTGGGCTCCACTGCTGTTATTCTAGATGATAACTTTGTAA
CAAAGGCCAATGCCCTAACCTATGACCCCTATGTAAACTACTCCTCCCGCCATACCATAACCCAGCCCTTCTCCTACCAC
TCCCGGTACTTTACCCCGAAACCTGTCCTTGATAGGACAATCGATTACTTCCAACCCAATAACAAAAGAAATCAACTCT
GGCTGAGACTACAAACTACTGGAAATGTAGACCATGTAGGCCTCGGCACTGCGTTCGAAAACAGTATATACGACCAG
GACTACAATATCCGTGTAACCATGTATGTACAATTCAGAGAATTTAATCTTAAAGACCCCCCACTTAACCCT--------

>HQ395037_pcv2d

ATGACGTATCCAAGGAGGCGTTACCGGAGAAGAAGACACCGCCCCCGCAGCCATCTTGGCCAGATCCTCCGCCGCCG
CCCCTGGCTCCTCCACCCCCGCCACCGTTACCGCTGGAGAAGGAAAAATGGCATCTTCAACACCCGCCTCTCCCGCACC
ATCGGTTATACTGTCAAGAAAACCACAGTCAGAACGCCCTCCTGGAATGTGGACATGATGAGATTTAATATTAATGAT
TTTCTTCCCCCAGGAGGGGGCTCAAACCCCCTCACTGTGCCCTTTGAATACTACAGAATAAGGAAGGTTAAGGTTGAA
TTCTGGCCCTGCTCCCCAATCACCCAGGGTGACAGGGGAGTGGGCTCCACTGCTGTTATTCTAGATGATAACTTTGTAA
CAAAGGCCAATGCCCTAACCTATGACCCCTATGTAAACTACTCCTCCCGCCATACCATAACCCAGCCCTTCTCCTACCAC
TCCCGGTACTTTACCCCGAAACCTGTCCTTGATAGGACAATCGATTACTTCCAACCCAATAACAAAAGAAATCAACTCT
GGCTGAGACTACAAACTACTGGAAATGTAGACCATGTAGGCCTCGGCACTGCGTTCGAAAACAGTATATACGACCAG
GACTACAATACCCGTATAACCATGTATGTACAATTCAGAGAATTTAACCTTAAAGACCCCCCACTTAACCCTAAG-----

>JQ002671_pcv2d

ATGACGTATCCAAGGAGGCGTTACCGGAGAAGAAGACACCGCCCCCGCAGCCATCTTGGCCAGATCCTCCGCCGCCG
CCCCTGGCTCGTCCACCCCCGCCACCGTTACCGCTGGAGAAGGAAAAATGGCATCTTCAACACCCGCCTCTCCCGCACC
ATCGGTTATACTGTCAAGAAAACCACAGTCAGAACGCCCTCCTGGAATGTGGACATGATGAGATTTAATATTAATGAT
TTTCTTCCCCCAGGAGGGGGCTCAAACCCCCTCACTGTGCCCTTTGAATACTACAGAATAAGGAAGGTTAAGGTTGAA
TTCTGGCCCTGCTCCCCAATCACCCAGGGTGACAGGGGAGTGGGCTCCACTGCTGTTATTCTAGATGATAACTTTGTAA
CAAAGGCCAATGCCCTAACCTATGACCCCTATGTAAACTACTCCTCCCGCCATACCATAACCCAGCCCTTCTCCTACCAC
TCCCGGTACTTTACCCCGAAACCTGTCCTTGATAGGACAATCGATTACTTCCAACCCAATAACAAAAGAAATCAACTCT
GGCTGAGACTACAAACTACTGGAAATGTAGACCATGTAGGCCTCGGCACTGCGTTCGAAAACAGTATATACGACCAG
GACTACAATATCCGTATAACCATGTATGTACAATTCAGAGAATTTAATCTTAAAGACCCCCCACTTAACCCAAAG-----

>JF927985_pcv2d

ATGACGTATCCAAGGAGGCGTTACCGGAAAAGAAGACACCGCCCCCGCAGCCATCTTGGCCAGATCCTCCGCCGCCG
CCCCTGGCTCGTCCACCCCCGCCACCGTTACCGCTGGAGAAGGAAAAATGGCATCTTCAACACCCGCCTCTCCCGCACC
ATCGGTTATACTGTCAAGAAAACCACAGTCAGAACGCCCTCCTGGAATGTGGACATGATGAGATTTAATATTAATGAT
TTTCTTCCCCCAGGAGGGGGCTCAAACCCCCTCACTGTGCCCTTTGAATACTACAGAATAAGGAAGGTTAAGGTTGAA
TTCTGGCCCTGCTCCCCAATCACCCAGGGTGACAGGGGAGTGGGCTCCACTGCTGTTATTCTAGATGATAACTTTGTAA
CAAAGGCCAATGCCCTAACCTATGACCCCTATGTAAACTACTCCTCCCGCCATACCATAACCCAGCCCTTCTCCTACCAC
TCCCGGTACTTTACCCCGAAACCTGTCCTTGATAGGACAATCGATTACTTCCAACCCAATAACAAAAGAAATCAACTGT
GGCTGAGACTACAAACTACTGGAAATGTAGACCATGTAGGCCTCGGCACTGCGTTCGAAAACAGTATATACGACCAG
GACTACAATATCCGTATAACCATGTATGTACAATTCAGAGAATTTAATCTTAAAGACCCCCCACTTAACCCTAAG-----

>JF927984_pcv2d

ATGACGTTTCCAAGGAGGCGTTACCGAAGAAGAAGACACCGCCCCCGCAGCCATCTTGGCCAGATCCTCCGCCGCCG
CCCCTGGCTCGTCCACCCCCGCCACCGTTACCGCTGGAGAAGGAAAAATGGCATCTTCAACACCCGCCTCTCCCGCACC
ATCGGTTATACTGTCAAGAAAACCACAGTCAGAACGCCCTCCTGGAATGTGGACATGATGAGATTTAATATTAATGAT
TTTCTTCCCCCAGGAGGGGGCTCAAACCCCCTCACTGTGCCCTTTGAATACTACAGAATAAGGAAGGTTAAGGTTGAA
TTCTGGCCCTGCTCCCCAATCACCCAGGGTGACAGGGGAGTGGGCTCCACTGCTGTTATTCTAGATGATAACTTTGTAA
CAAAGGCCAATGCCCTAACCTACGACCCCTATGTAAACTACTCCTCCCGCCATACCATAACCCAGCCCTTCTCCTACCAC
TCCCGGTACTTTACCCCGAAACCTGTCCTTGATAGGACAATCGATTACTTCCAACCCAATAACAAAAGAAATCAACTGT
GGCTGAGACTACAAACTACTGGAAATGTAGACCATGTAGGCCTCGGCACTGCGTTCGAAAACAGTATATACGACCAG
GACTACAATATCCGTATAACCATGTATGTACAATTCAGAGAATTTAATCTTAAAGACCCCCCACTTAACCCTAAA-----

>JF683408_pcv2d

ATGACGTATTCCAGGAGGCGTTTCCGCAGAAGAAGACACCGCCCCCGCAGCCATCTTGGCCAGATCCTCCGCCGCCGC
CCCTGGCTCGTCCACCCCCGCCACCGTTACCGCTGGAGAAGGAAAAATGGCATCTTCAACACCCGCCTCTCCCGCACCA
TCGGTTATACTGTGAAGAAAACCACAGTCAGAACGCCCTCCTGGAATGTGGACATGATGAGATTTAATATTAATGATT
TTCTTCCCCCAGGAGGGGGCTCAAACCCCCTCACTGTGCCCTTTGAATACTACAGAATAAGGAAGGTTAAGGTTGAAT
TCTGGCCCTGCTCCCCAATCACCCAGGGTGACAGGGGAGTGGGCTCCACTGCTGTTATTCTAGATGATAACTTTGTAA
CAAAGGCCAATGCCCTAACCTATGACCCCTATGTAAACTACTCCTCCCGCCATACCATAACCCAGCCCTTCTCCTACCAC
TCCCGGTACTTTACCCCGAAACCTGTCCTTGATAGGACAATCGATTACTTCCAACCCAATAACAAAAGAAATCAACTCT
GGCTGAGACTACAAACCTCTGCAAATGTAGACCACGTAGGCCTCGGCACTGCGTTCGAAAACAGTAAATACGACCAG
GACTACAATATCCGTATAACCATGTATGTACAATTCAGAGAATTTAATCTTAAAGACCCCCCACTTAACCCTAAA-----

>JN176181_pcv2d

ATGACGTATCCAAGGAGGCGTTTCCGCAGAAGAAGACACCGCCCCCGCAGCCATCTTGGCCAGATCCTCCGCCGCCG
CCCCTGGCTCGTCCACCCCCGCCACCGTTACCGCTGGAGAAGGAAAAATGGCATCTTCAACACCCGCCTCTCCCGCACC
ATCGGTTATACTGTCAAGAAAACCACAGTCAGAACGCCCTCCTGGAATGTGGACATGATGAGATTTAACATTAATGAT
TTTCTTCCCCCAGGAGGGGGCTCAAACCCCCTCACTGTGCCCTTTGAATACTACAGAATAAGGAAGGTTAAGGTTGAA
TTCTGGCCCTGCTCCCCAATCACCCAGGGTGACAGGGGAGTGGGCTCCACTGCTGTTATTCTAGATGATAACTTTGTAA
CAAAGGCCAATGCCCTAACCTATGACCCCTATGTAAACTACTCCTCCCGCCATACCATAACCCAGCCCTTCTCCTACCAC
TCCCGGTACTTTACCCCGAAACCTGTCCTTGATGGGACAATCGATTACTTCCAACCCAATAACAAAAGAAATCAACTGT
GGCTGAGACTACAAACTACTGGAAATGTAGACCATGTAGGCCTCGGCACTGCGTTCGAAAACAGTATATACGACCAG
GACTACAATATCCGTATAACCATGTATGTACAATTCAGAGAATTTAATCTTAAAGACCCCCCACTTAACCCTAAG-----

>JF683404_pcv2d

ATGACGTATCCAAGGAGGCGTTTCCGCAGAAGAAGACACCGCCCCCGCAGCCATCTTGGCCAGATCCTCCGCCGCCG
CCCCTGGCTCGTCCACCCCCGCCACCGTTACCGCTGGAGAAGGAAAAATGGCATCTTCAACACCCGCCTCTCCCGCACC
ATCGGTTATACTGTCAAGAAAACCACAGTCAGAACGCCCTCCTGGAATGTGGACATGATGAGATTTAATATTAATGAT
TTTCTTCCCCCAGGAGGGGGCTCAAACCCCCTCACTGTGCCCTTTGAATACTACAGAATAAGGAAGGTTAAGGTTGAA
TTCTGGCCCTGCTCCCCAATCACCCAGGGTGACAGGGGAGTGGGCTCCACTGCTGTTATTCTAGATGATAACTTTGTAA
CAAAGGCCAATGCCCTAACCTATGACCCCTATGTAAACTACTCCTCCCGCCATACCATAACCCAGCCCTTCTCCTACCAC
TCCCGGTACTTTACCCCGAAACCTGTCCTTGATAGGACAATCGATTACTTCCAACCCAATAACAAAAGAAATCAACTGT
GGCTGAGACTACAAACTACTGGAAATGTAGACCATGTAGGCCTCGGCACTGCGTTCGAAAACAGTATATACGACCAG
GACTACAATATCCGTATAACCATGTATGTACAATTCAGAGAATTTAATCTTAAAGACCCCCCACTTAACCCTAAG-----

>JF683399_pcv2d

ATGACGTATCCAAGGAGGCGTTTCCGCAGAAGAAGACACCGCCCCCGCAGCCATCTTGGCCAGATCCTCCGCCGCCG
CCCCTGGCTCGTCCACCCCCGCCACCGTTACCGCTGGAGAAGGAAAAATGGCATCTTCAACACCCGCCTCTCCCGCACC
ATCGGTTATACTGTCAAGAAAACCACAGTCAGAACGCCCTCCTGGAATGTGGACATGATGAGATTTAATATTAATGAT
TTTCTTCCCCCAGGAGGGGGCTCAAACCCCCTCACTGTGCCCTTTGAATACTACAGAATAAGGAAGGTTAAGGTTGAA
TTCTGGCCCTGCTCCCCAATCACCCAGGGTGACAGGGGAGTGGGCTCCACTGCTGTTATTCTAGATGATAACTTTGTAA
CAAAGGCCAATGCCCTAACCTATGACCCCTATGTAAACTACTCCTCCCGCCATACCATAACCCAGCCCTTCTCCTACCAC
TCCCGGTACTTTACCCCGAAACCTGTCCTTGATAGGACAATCGATTACTTCCAACCCAATAACAAAAGAAATCAACTGT
GGCTGAGACTACAAACTACTGGAAATGTAGACCATGTAGGCCTCGGCACTGCGTTCGAAAACAGTATATACGACCAG
GACTACAATATCCGTATAACCATGTATGTACAATTCAGAGAATTTAATCTTAAAGACCCCCCACTTAACCCTAAG-----

>JF683401_pcv2d

ATGACGTATCCAAGGAGGCGTTTCCGCAGAAGAAGACACCGCCCCCGCAGCCATCTTGGCCAGATCCTCCGCCGCCG
CCCCTGGCTCGTCCACCCCCGCCACCGTTACCGCTGGAGAAGGAAAAATGGCATCTTCAACACCCGCCTCTCCCGCACC
ATCGGTTATACTGTCAAGAAAACCACAGTCAGAACGCCCTCCTGGAATGTGGACATGATGAGATTTAATATTAATGAT
TTTCTTCCCCCAGGAGGGGGCTCAAACCCCCTCACTGTGCCCTTTGAATACTACAGAATAAGGAAGGTTAAGGTTGAA
TTCTGGCCCTGCTCCCCAATCACCCAGGGTGACAGGGGAGTGGGCTCCACTGCTGTTATTCTAGATGATAACTTTGTAA
CAAAGGCCAATGCCCTAACCTATGACCCCTATGTAAACTACTCCTCCCGCCATACCATAACCCAGCCCTTCTCCTACCAC
TCCCGGTACTTTACCCCGAAACCTGTCCTTGATAGGACAATCGATTACTTCCAACCCAATAACAAAAGAAATCAACTGT
GGCTGAGACTACAAACTACTGGAAATGTAGACCATGTAGGCCTCGGCACTGCGTTCGAAAACAGTATATACGACCAG
GACTACAATATCCGTATAACCATGTATGTACAATTCAGAGAATTTAATCTTAAAGACCCCCCACTTAACCCTAAG-----

>JF683389_pcv2d

ATGACGTATCCAAGGAGGCGTTTCCGCAGAAGAAGACACCGCCCCCGCAGCCATCTTGGCCAGATCCTCCGCCGCCG
CCCCTGGCTCGTCCACCCCCGCCACCGTTACCGCTGGAGAAGGAAAAATGGCATCTTCAACACCCGCCTCTCCCGCACC
ATCGGTTATACTGTCAAGAAAACCACAGTCAGAACGCCCTCCTGGAATGTGGACATGATGAGATTTAATATTAATGAT
TTTCTTCCCCCAGGAGGGGGCTCAAACCCCCTCACTGTGCCCTTTGAATACTACAGAATAAGGAAGGTTAAGGTTGAA
TTCTGGCCCTGCTCCCCAATCACCCAGGGTGACAGGGGAGTGGGCTCCACTGCTGTTATTCTAGATGATAACTTTGTAA
CAAAGGCCAATGCCCTAACCTATGACCCCTATGTAAACTACTCCTCCCGCCATACCATAACCCAGCCCTTCTCCTACCAC
TCCCGGTACTTTACCCCGAAACCTGTCCTTGATAGGACAATCGATTACTTCCAACCCAATAACAAAAGAAATCAACTGT
GGCTGAGACTACAAACTACTGGAAATGTAGACCATGTAGGCCTCGGCACTGCGTTCGAAAACAGTATATACGACCAG
GACTACAATATCCGTATAACCATGTATGTACAATTCAGAGAATTTAATCTTAAAGACCCCCCACTTAACCCTAAG-----

>JF927986_pcv2d

ATGACGTATTCAAGGAGGCGTTTCCGCAGAAGAAGACACCGCCCCCGCAGCCATCTTGGCCAGATCCTCCGCCGCCGC
CCCTGGCTCGTCCACCCCCGCCACCGTTACCGCTGGAGAAGGAAAAATGGCATCTTCAACACCCGCCTCTCCCGCACCA
TCGGTTATACTGTCAAGAAAACCACAGTCAGAACGCCCTCCTGGAATGTGGACATGATGAGATTTAATATTAATGATT
TTCTTCCCCCAGGAGGGGGCTCAAACCCCCTCACTGTGCCCTTTGAATACTACAGAATAAGGAAGGTTAAGGTTGAAT
TCTGGCCCTGCTCCCCAATCACCCAGGGTGACAGGGGAGTGGGCTCCACTGCTGTTATTCTAGATGATAACTTTGTAA
CAAAGGCCAATGCCCTAACCTATGACCCCTATGTAAACTACTCCTCCCGCCATACCATAACCCAGCCCTTCTCCTACCAC
TCCCGGTACTTTACCCCGAAACCTGTCCTTGATAGGACAATCGATTACTTCCAACCCAATAACAAAAGAAATCAACTGT
GGCTGAGACTACAAACTACTGGAAATGTAGACCATGTAGGCCTCGGCACTGCGTTCGAAAACAGTATATACGACCAG
GACTACAATATCCGTATAACCATGTATGTACAATTCAGAGAATTTAATCTTAAAGACCCCCCACTTAACCCTAAG-----

>JF683405_pcv2d

ATGACGTATCCAAGGAGGCGTTTCCGCAGAAGAAGACACCGCCCCCGCAGCCATCTTGGCCAGATCCTCCGCCGCCG
CCCCTGGCTCGTCCACCCCCGCCACCGTTACCGCTGGAGAAGGAAAAATGGCATCTTCAACACCCGCCTCTCCCGCACC
ATCGGTTATACTGTCAAGAAAACCACAGTCAGAACTCCCTCCTGGAATGTGGACATGATGAGATTTAATATTAATGATT
TTCTTCCCCCAGGAGGGGGCTCAAACCCCCTCACTGTGCCCTTTGAATACTACAGAATAAGGAAGGTTAAGGTTGAAT
TCTGGCCCTGCTCCCCAATCACCCAGGGTGACAGGGGAGTGGGCTCCACTGCTGTTATTCTAGATGATAACTTTGTAA
CAAAGGCCAATGCCCTAACCTATGACCCCTATGTAAACTACTCCTCCCGCCATACCATAACCCAGCCCTTCTCCTACCAC
TCCCGGTACTTTACCCCGAAACCTGTCCTTGATAGGACAATCGATTACTTCCAACCCAATAACAAAAGAAATCAACTGT
GGCTGAGACTACAAACTACTGGAAATGTAGACCATGTAGGCCTCGGCACTGCGTTCGAAAACAGTATATACGACCAG
GACTACAATATCCGTATAACCATGTATGTACAATTCAGAGAATTTAATCTTAAAGACCCCCCACTTAACCCTAAG-----

>FJ644931_pcv2d

ATGACGTATCCAAGGAGGCGTTTCCGCAGACGAAGACACCGCCCCCGCAGCCATCTTGGCCAGATCCTCCGCCGCCGC
CCCTGGCTCGTCCACCCCCGCCACCGTTACCGCTGGAGAAGGAAAAATGGCATCTTCAACACCCGCCTCTCCCGCACCA
TCGGTTATACTGTCAAGAAAACCACAGTCAGAACGCCCTCCTGGAATGTGGACATGATGAGATTTAATATTAATGACT
TTCTTCCGCCAGGAGGGGGCTCAAACCCCCGCTCTGTGCCCTTTGAATACTACAGAATAAGAAAGGTTAAGGTTGAAT
TCTGGCCCTGCTCCCCAATCACCCAGGGTGACAGGGGAGTGGGCTCCACTGCTGTTATTCTAGATGATAACTTTGTAA
CAAAGGCCAATGCCCTAACCTATGACCCCTATGTAAACTACTCCTCCCGCCATACCATAACCCAGCCCTTCTCCTACCAC
TCCCGATACTTTACCCCGAAACCTGTCCTTGATAGGACAATCGATTACTTCCAACCCAATAACAAAAGAAATCAACTCT
GGCTGAGACTACAAACTACTGGAAATGTAGACCATGTAGGCCTCGGCACTGCGTTCGAAAACAGTATATACGACCAG
GACTACAATATCCGTATAACCATGTATGTACAATTCAGAGAATTTAATCTTAAAGACCCCCCACTTAACCCTAAG-----

>FJ948168_pcv2d

ATGACGTATCCAAGGAGGCGTTTCCGCAGACGAAGACACCGCCCCCGCAGCCATCTTGGCCAGATCCTCCGCCGCCGC
CCCTGGCTCGTCCACCCCCGCCACCGTTACCGCTGGAGAAGGAAAAATGGCATCTTCAACACCCGCCTCTCCCGCACCT
TCGGTTATACTGTCAAGAAAACCACAGTCAGAACGCCCTCCTGGGCGGTGGACATGATGAGATTTAATATTAATGATT
TTCTTCCCCCAGGAGGGGGCTCAAACCCCCGCACTGTGCCCTTTGAATACTACAGAATAAGGAAGGTTAAGGTTGAAT
TCTGGCCCTGCTCCCCAATCACCCAGGGTGACAGGGGAGTGGGCTCCACTGCTGTTATTCTAGATGATAACTTTGTAA
CAAAGGCCAATGCCCTAACCTATGACCCCTATGTAAACTACTCCTCCCGCCATACCATAACCCAGCCCTTCTCCTACCAC
TCCCGGTACTTTACCCCGAAACCTGTCCTTGATAGGACAATCGATTACTTCCAACCCAATAACAAAAGAAATCAACTCT
GGCTGAGACTACAAACTACTGGAAATGTAGACCATGTAGGCCTCGGCACTGCGTTCGAAAACAGTATATACGACCAG
GACTACAATATCCGTATAACCATGTATGTACAATTCAGAGAATTTAATCTTAAAGACCCCCCACTTAACCCTAAG-----

>GU808525_pcv2d

ATGACGTATCCAAGGAGGCGTTACCGAAGACGAAGACACCGCCCCCGCAGCCATCTTGGCCAGATCCTCCGCCGCCG
CCCCTGGCTCGTCCACCCCCGCCACCGTTACCGCTGGAGAAGGAAAAATGGCATCTTCAACACCCGCCTCTCCCGCACC
TTCGGATACACTGTCAAGGCTACCACAGTCAGAACGCCCTCCTGGGCGGTGGACATGATGAGATTTAATATTAATGAT
TTTCTTCCCCCAGGAGGGGGCTCAAACCCCCTCACTGTGCCCTTTGAATACTACAGAATAAGGAAGGTTAAGGTTGAA
TTCTGGCCCTGCTCCCCAATCACCCAGGGTGACAGGGGAGTGGGCTCCACTGCTGTTATTCTAGATGATAACTTTGTAA
CAAAGGCCAATGCCCTAACCTATGACCCCTATGTAAACTACTCCTCCCGCCATACCATAACCCAGCCCTTCTCCTACCAC
TCCCGCTATTTTACCCCCAAACCTGTCCTTGATAGGACAATCGATTACTTCCAACCCAATAACAAAAGAAATCAACTCTG
GCTGAGACTACAAACTACTGGAAATGTAGACCATGCAGGCCTCGGCACTGCGTTCGAAAACAGTATATACGACCAGG
ACTACAATATCCGTGTAACCATGTATGTACAATTCAGAGAATTTAATCTTAAAGACCCCCCACTTAACCCTAAG-----

>AY484410_pcv2d

ATGACGTATCCAAGGAGGCGTTACCGAAGACGAAGACACCGCCCCCGCAGCCATCTTGGCCAGATCCTCCGCCGCCG
CCCCTGGCTCGTCCACCCCCGCCACCGTTACCGCTGGAGAAGGAAAAATGGCATCTTCAACACCCGCCTCTCCCGCACC
TTCGGATATACTGTCAAGGCTACCACAGTCAGAACGCCCTCCTGGGCGGTGGACATGATGAGATTTAATATTAATGAT
TTTCTTCCCCCAGGAGGGGGCTCAAACCCCCTCACTGTGCCCTTTGAATACTACAGAATAAGGAAGGTTAAGGTTGAA
TTCTGGCCCTGCTCCCCAATCACCCAGGGTGACAGGGGAGTGGGCTCCACTGCTGTTGTTCTAGATGATAACTTTGTA
ACAAAGGCCAATGCCCTAACCTATGACCCCTATGTAAACTACTCCTCCCGCCATACCATACCCCAACCCTTCTCCTACCA
CTCCCGCTACTTCACCCCCAAACCTGTCCTTGATAGGACAATCGATTACTTCCAACCCAATAACAAAAGAAATCAACTCT
GGCTGAGACTGCAAACTACTGCAAATGTAGACCATGTAGGCCTCGGCACTGCCTTCGAAAACAGTAAATACGACCAG
GACTACAATATCCGTGTAACCATGTATGTACAATTCAGAGAATTTAATCTTAAAGACCCCCCACTTAAACCTAAG-----

>AY510375_pcv2d

ATGACGTATCCAAGGAGGCGTTACCGAAGACGAAGACACCGCCCCCGCAGCCATCTTGGCCAAATCCTCCGCCGCCG
CCCCTGGCTCGTCCACCCCCGCCACCGTTACCGCTGGAGAAGGAAAAATGGCATCTTCAACAGCCGCCTCTCCCGCAC
CATCGGTTATACTGTCAAGGCTACCACAGTCAGAACGCCCTCCTGGGCGGTGGACATGATGAGATTTAATATTAATGA
TTTTCTTCCCCCAGGAGGGGGCTCAAACCCCCTCACTGTGCCCTTTGAATACTACAGAATAAGGAAGATTAAGGTTGA
ATTCTGGCCATGCTCCCCAATCACCCAGGGTGACAGGGGAGTGGGCTCCACTGCTGTTATTCTAGATGATAACTTTGT
AACAAAGGCCAATGCCCTAACCTATGACCCCTATGTAAACTACTCCTCCCGCCATACCATACCCCAGCCCTTCTCCTACC
ACTCCCGCTATTTCACCCCCAAACCTGTCCTTGATAGGACAATCGATTACTTCCAACCCAATAACAAAAGAAATCAACTC
TGGCTGAGACTACAAACTACTGGAAATGTAGACCATGTAGGCCTCGGCACTGCGTTCGAAAACAGTAAATACGACCA
GGACTACAATATCCGTATAACCATGTATGTACAATTCAGAGAATTTAATCTTAAAGACCCCCCACTTAACCCTAAG-----

>FJ158604_pcv2d

ATGACGTATCCAAGGAGGCGTTACCGAAGACGAAGACACCGCCCCCGCAGCCATCTTGGCCAAATCCTCCGCCGCCG
CCCCCGGCTCGTCCACCCCCGCCACCGTTACCGCTGGAGAAGGAAAAATGGCATCTTCAACACCCGCCTCTCCCGCAC
CATCGGTTATACTGTCAAGGCTACCACAGTCAGAACGCCCTCCTGGGCGGTGGACATGATGAGATTTAATATTAATGA
TTTTCTTCCCCCAGGAGGGGGCTCAAACCCCCTCACTGTGCCCTTTGAATACTACAGAATAAGGAAGATTAAGGTTGA
ATTCTGGCCCTGCTCCCCAATCACCCAGGGTGACAGGGGAGTGGGCTCCACTGCTGTTATTCTAGATGATAACTTTGTA
ACAAAGGCCAATGCCCTAACCTATGACCCCTATGTAAACTACTCCTCCCGCCATACCATACCCCAGCCCTTCTCCTACCA
CTCCCGCTATTTCACCCCCAAACCTGTCCTTGATAGGACAATCGATTACTTCCAACCCAATAACAAAAGAAATCAACTCT
GGCTGAGACTACAAACTACTGGAAATGTAGACCATGTAGGCCTCGGCACTGCGTTCGAAAACAGTAAATACGACCAG
GACTACAATATCCGTATAACCATGTATGTACAATTCAGAGAATTTAATCTTAAAGACCCCCCACTTAACCCTAAG-----

>FJ158607_pcv2d

ATGACGTATCCAAGGAGGCGTTACCGAAGACGAAGACACCGCCCCCGCAGCCATCTTGGCCAAATCCTCCGCCGCCG
CCCCTGGCTCGTCCACCCCCGCCACCGTTACCGCTGGAGAAGGAAAAATGGCATCTTCAACACCCGCCTCTCCCGCACC
ATCGGTTATACTGTCAAGGCTACCACAGTCAGAACGCCCTCCTGGGCGGTGGACATGATGAGATTTAATATTAATGAT
TTTCTTCCCCCAGGAGGGGGATCAAACCCCCTCACTGTGCCCTTTGAATACTACAGAATAAGGAAGATTAAGGTTGAA
TTCTGGCCCTGCTCCCCAATCACCCAGGGTGACAGGGGAGTGGGCTCCACTGCTGTTATTCTAGATGATAACTTTGTAA
CAAAGGCCAATGCCCTAACCTATGACCCCTATGTAAACTACTCCTCCCGCCATACCATACCCCAGCCCTTCTCCTACCAC
TCCCGCTATTTCACCCCCAAACCTGTCCTTGATAGGACAATCGATTACTTCCAACCCAATAACAAAAGAAATCAACTCTG
GCTGAGACTACAAACTACTGGAAATGTAGACCATGTAGGCCTCGGCACTGCGTTCGAAAACAGTAAATACGACCAGG
ACTACAATATCCGTATAACCATGTATGTACAATTCAGAGAATTTAATCTTAAAGACCCCCCACTTAACCCTAAG-----

>AY181947_pcv2d

ATGACGTATCCAAGGAGGCGTTACCGAAGACGAAGACACCGCCCCCGCAGCCATCTTGGCCAAATCCTCCGCCGCCG
CCCCTGGCTCGTCCACCCCCGCCACCATTACCGCTGGAGAAGGAAAAATGGCATCTTCAACACCCGCCTCTCCCGCACC
ATCGGTTATACTGTCAAGGCTACCACAGTCAGAACGCCCTCCTGGGCGGTGGACATGATGAGATTTAATATTAATGAT
TTTCTTCCCCCAGGAGGGGGCTCAAACCCCCTCACTGTGCCCTTTGAATACTACAGAATAAGGAAGATTAAGGTTGAA
TTCTGGCCCTGCTCCCCAATCACCCAGGGTGACAGGGGAGTGGGCTCCACTGCTGTTATTCTAGATGATAACTTTGTAA
CAAAGGCCACAGCCCTAACCTATGACCCCTATGTAAACTACTCCTCCCGCCATACCATACCCCAGCCCTTCTCCTACCAC
TCCCGCTATTTCACCCCCAAACCTGTCCTTGATAGGACAATCGATTACTTCCAACCCAATAACAAAAGAAATCAACTCTG
GCTGAGACTACAAACTACTGGAAATGTAGACCATGTAGGCCTCGGCACTGCGTTCGAAAACAGTAAATACGACCAGG
ACTACAATATCCGTATAACCATGTATGTACAATTCAGAGAATTTAATCTTAAAGACCCCCCACTTAACCCTAAG-----

>AY686763_pcv2d

ATGACGTATCCAAGGAGGCGTTACCGAAGACGAAGACACCGCCCCCGCAGCCATCTTGGCCAAATCCTCCGCCGCCG
CCCCTGGCTCGTCCACCCCCGCCTCCGTTACCGCTGGAGAAGGAAAAATGGCATCTTCAACACCCGCCTCTCCCGCACC
ATCGGTTATACTGTCAAGGCTACCACAGTCAGAACGCCCTCCTGGGCGGTGGACATGATGAGATTTAATATTAATGAT
TTTCTTCCCCCAGGAGGGGGCTCAAACCCCCTCACTGTGCCCTTTGAATACTACAGAATAAGAAAGGTTAAGGTTGAA
TTCTGGCCCTGCTCCCCAATCACCCAGGGTGACAGGGGAGTGGGCTCCACTGCTGTTATTCTAGATGATAACTTTGTAA
CAAAGGCCAATGCCCTAACCTATGACCCCTATGTAAACTACTCCTCCCGCCATACCATACCCCAGCCCTTCTCCTACCAC
TCCCGCTATTTCACCCCCAAACCTGTCCTTGATAGGACAATCGATTACTTCCAACCCAATAACAAAAGAAATCAACTCTG
GCTGAGACTACAAACTACTGGAAATGTAGACCATGTAGGCCTCGGCACTGCGTTCGAAAACAGTAAATACGACCAGG
ACTACAATATCCGTATAACCATGTATGTACAATTCAGAGAATTTAATCTTAAAGACCCCCCACTTAACCCTAAG-----

>AY686765_pcv2d

ATGACGAATCCTAGGAGGCGTTACCGAAGACGAAGACACCGCCCCCGCAGCCATCTTGGCCAAATCCTCCGCCGCCG
CCCCTGGCTCGTCCACCCCCGCCACCGTTACCGCTGGAGAAGGAAAAATGGCATCTTCAACACCCGCCTCTCCCGCACC
ATCGGTTATACTGTCAAGGCTACCACAGTCAGAACGCCCTCCTGGGCGGTGGACATGATGAGATTTAATATCAATGAT
TTTCTTCCCCCAGGAGGGGGCTCAAACCCCCTCACTGTACCCTTTGAATACTACAGAATAAGAAAGGTTAAGGTTGAA
TTCTGGCCCTGCTCCCCAATCACGCAGGGTGACAGGGGAGTGGGCTCCACTGCTGTTATTCTAGATGATAACTTTGTA
ACAAAGGCCAATGCCCTAACCTATGACCCCTATGTAAACTACTCCTCCCGCCATACCATACCCCAGCCCTTCTCCTACCA
CTCCCGCTATTTCACCCCCAAACCTGTCCTTGATAGGACAATCGATTACTTCCAACCCAATAACAAAAGAAATCAACTCT
GGCTGAGACTACAAACTACTGGAAATGTAGACCATGTAGGCCTCGGCACTGCGTTCGAAAACAGTAAATACGACCAG
GACCACAATATCCGTATAACCATGTATGTGCAATTCAGAGAATTTAATCTTAAAGACCCCCCACTTAACCCTAAG-----

>AY556476_pcv2d

ATGACGTATCCAAGGAGGCGTTACCGAAGACGAAGACACCGCCCCCGCAGCCATCTTGGCCAAATCCTCCGCCGCCG
CCCCTGGCTCGTCCACCCCCGCCACCGTTACCGCTGGAGAAGGAAAAATGGCATCTTCAACACCCGCCTCTCCCGCACC
ATCGGTTATACTGTCAAGGCTACCACAGTCAGAACGCCCTCCTGGGCGGTGGACATGATGAGATTTAATATTAATGAT
TTTCTTCCCCCAGGAGGGGGCTCAAACCCCCTCACTGTGCCCTTTGAATACTACAGAATAAGAAAGGTTAAGGTTGAA
TTCTGGCCCTGCTCCCCAATCACCCAGGGTGACAGGGGAGTGGGCTCCACTGCTGTTATTCTAGATGATAACTTTGTAA
CAAAGGCCAATGCCCTAACCTATGACCCCTATGTAAACTACTCCTCCCGCCATACCATACCCCAGCCCTTCTCCTACCAC
TCCCGCTATTTCACCCCCAAACCTGTCCTTGATAGGACAATCGATTACTTCCAACTCAATAACAAAAGAAATCAACTCTG
GCTGAGACTACAAACTACTGGAAATGTAGACCATGTAGGCCTCGGCACTGCGTTCGAAAACAGTAAATACGACCAGG
ACTACAATATCCGTATAACCATGTATGTACAATTCAGAGAATTTAATCTTAAAGACCCCCCACTTAACCCTAAG-----

>HQ378158_pcv2d

ATGACGTATCCAAGGAGGCGTTACCGAAGACGAAGACACCGCCCCCGCAGCCATCTTGGCCAGATCCTCCGCCGCCG
CCCCTGGCTCGTCCACCCCCGCCACCGTTACCGCTGGAGAAGGAAAAATGGCATCTTCAACACCCGCCTCTCCCGCACC
ATCGGTTATACTGTCAAGGCTACCACAGTCAGAACGCCCTCCTGGAATGTGGACATGATGAGATTTAATATTAATGAT
TTTCTTCCCCCAGGAGGGGGCTCAAACCCCCTCACTGTGCCCTTTGAATACTACAGAATAAGGAAGGTTAAGGTTGAA
TTCTGGCCCTGCTCCCCAATCACCCAGGGTGACAGGGGAGTGGGCTCCACTGCTGTTATTCTAGATGATAACTTTGTAA
CAAAGGCCACAGCCCTAACCTATGACCCCTATGTAAACTACTCCTCCCGCCATACCATACCCCAACCCTTCTCCTACCAC
TCCCGCTACTTCACCCCCAAACCTGTCCTTGATAGGACAATCGATTACTTCCAACCCAATAACAAAAGAAATCAACTCT
GGCTGAGACTACAAACTACTGGAAATGTAGACCATGTAGGCCTCGGCACTGCGTTCGAAAACAGTATATACGACCAG
GACTACAATATCCGTATAACCATGTATGTACAATTCAGAGAATTTAATCTTAAAGACCCCCCACTTAACCCTAAG-----

>JN006445_pcv2d

ATGACGTATCCAAGGAGGCGTTTCCGCAGACGAAGACACCGCCCCCGCAGCCATCTTGGCCAGATCCTCCGCCGCCGC
CCCTGGCTCGTCCACCCCCGCCACCGTTACCGCTGGAGAAGGAAAAATGGCATCTTCAACACCCGCCTCTCCCGCACCA
TCGGTTATACTGTCAAGAAAACCACAGTCAGAACGCCCTCCTGGAATGTGGACATGATGAGATTTAATATTAATGATT
TTCTTCCCCCAGGAGGGGGCTCAAACCCCCTCACTGTGCCCTTTGAATACTACAGAATAAGGAAGGTTAAGGTTGAAT
TCTGGCCCTGCTCCCCAATCACCCAGGGTGACAGGGGAGTGGGCTCCACTGCTGTTATTCTAGATGATAACTTTGTAA
CAAAGGCCAATGCCCTAACCTATGACCCCTATGTAAACTACTCCTCCCGCCATACCATACCCCAGCCCTTCTCCTACCAC
TCCCGCTATTTCACCCCCAAACCTGTCCTTGATAGGACAATCGATTACTTCCAACCCAATAACAAAAGAAATCAACTCTG
GCTGAGACTACAAACTACTGGAAATGTAGACCATGTAGGCCTCGGCACTGCGTTCGAAAACAGTATATACGACCAGG
ACTACAATATCCGTATAACCATGTATGTACAATTCAGAGAATTTAATCTTAAAGACCCCCCACTTAACCCTAAG-----

>HQ378157_pcv2d

ATGACGTATCCAAGGAGGCGTTACCGCAGACGAAGACACCGCCCCCGCAGCCATCTTGGCCAGATCCTCCGCCGCCG
CCCCTGGCTCGTCCACCCCCGCCACCGTTACCGCTGGAGAAGGAAAAATGGCATCTTCAACACCCGCCTCTCCCGCACC
ATCGGTTATACTGTCAAGGCTACCACAGTCAGAACGCCCTCCTGGAATGTGGACATGATGAGATTTAATATTAATGAT
TTTCTTCCCCCAGGAGGGGGCTCAAACCCCCTCACTGTGCCCTTTGAATACTACAGAATAAGGAAGGTTAAGGTTGAA
TTCTGGCCCTGCTCCCCAATCACCCAGGGTGACAGGGGAGTGGGCTCCACTGCTGTTATTCTAGATGATAACTTTGTAA
CAAAGGCCAATGCCCTAACCTATGACCCCTATGTAAACTACTCCTCCCGCCATACCATACCCCAGCCCTTCTCCTACCAC
TCCCGGTACTTTACCCCGAAACCTGTCCTTGATAGGACAATCGATTACTTCCAACCCAATAACAAAAGAAATCAACTCT
GGCTGAGACTACAAACTACTGGAAATGTAGACCATGTAGGCCTCGGCACTGCGTTCGAAAACAGTATATACGACCAG
GACTACAATATCCGTATAACCATGTATGTACAATTCAGAGAATTTAATCTTAAAGACCCCCCACTTAACCCTAAG-----

>AY556473_pcv2d

ATGACGTATCCAAGGAGGCGTTACCGAAGACGAAGACACCGCCCCCGCAGCCATCTTGGCCAGATCCTCCGCCGCCG
CCCCTGGCTCGTCCACCCCCGCCACCGTTACCGCTGGAGAAGGAAAAATGGCATCTTCAACACCCGCCTCTCCCGCACC
ATCGGTTATACTGTCAAAGCTACCACAGTAAGAACGCCCTCCTGGAATGTGGACATGATGAGATTTAATATTAATGAT
TTTCTTCCCCCAGGAGGGGGCTCAAACCCCCTCACTGTGCCCTTTGAATACTACAGAATAAGAAAGGTTAAGGTTGAA
TTCTGGCCCTGCTCCCCAATCACCCAGGGTGACAGGGGAGTGGGCTCCACTGCTGTTATTCTAGATGATAACTTTGTAA
CAAAGGCCAATGCCCTAACCTATGACCCCTATGTAAACTACTCCTCCCGCCATACCATACCCCAGCCCTTCTCCTACCAC
TCCCGCTATTTCACCCCCAAACCTGTCCTTGATAGGACAATCGATTACTTCCAACCCAATAACAAAAGAAATCAACTCTG
GCTGAGACTACAAACTACTGGAAATGTAGACCATGTAGGCCTCGGCACTGCGTTCGAAAACAGTATATACGACCAGG
ACTACAATATCCGTATAACCATGTATGTACAATTCAGAGAATTTAATCTTAAAGACCCCCCACTTAACCCTAAG-----

>EF990645_pcv2d

ATGACGTATCCAAGGAGGCGTTACCGAAGACGAAGACACCGCCCCCGCAGCCATCTTGGCCAGATCCTCCGCCGCCG
CCCCTGGCTCGTCCACCCCCGCCACCGTTACCGCTGGAGAAGGAAAAATGGCATCTTCAACACCCGCCTCTCCCGCACC
ATCGGTTATACTGTCAAAGCTACCACAGTCAGAACGCCCTCCTGGAATGTGGACATGATGAGATTTAATATTAATGATT
TTCTTCCCCCAGGAGGGGGCTCAAACCCCCTCACTGTGCCCTTTGAATACTACAGAATAAGGAAGGTTAAGGTTGAAT
TCTGGCCCTGCTCCCCAATCACCCAGGGTGACAGGGGAGTGGGCTCCACTGCTGTTATTCTAGATGATAACTTTGTAA
CAAAGGCCAATGCCCTAACCTATGACCCCTATGTAAACTACTCCTCCCGCCATACCATACCCCAGCCCTTCTCCTACCAC
TCCCGCTATTTCACCCCCAAACCTGTCCTTGATAGGACAATCGATTACTTCCAACCCAATAACAAAAGAAATCAACTCTG
GCTGAGACTACAAACTACTGGAAATGTAGACCATGTAGGACTCGGCACTGCCTTCGAAAACAGTAAATACGACCAGG
ACTACAATATCCGTATAACCATGTATGTACAATTCAGAGAATTTAATCTTAAAGACCCCCCACTTAACCCTAAG-----

>EU909686_pcv2d

ATGACGTATCCAAGGAGGCGTTACCGAAGACGAAGACACCGCCCCCGCAGCCATCTTGGCCAGATCCTCCGCCGCCG
CCCCTGGCTCGTCCACCCCCGCCACCGTTACCGCTGGAGAAGGAAAAATGGCATCTTCAACACCCGCCTCTCCCGCACC
ATCGGTTATACTGTCAAAGCTACCACAGTCAGAACGCCCTCCTGGAATGTGGACATGATGAGATTTAATATTAATGATT
TTCTTCCCCCAGGAGGGGGCTCAAACCCCCTCACTGTGCCCTTTGAATACTACAGAATAAGGAAGGTTAAGGTTGAAT
TCTGGCCCTGCTCCCCAATCACCCAGGGTGACAGGGGAGTGGGCTCCACTGCTGTTATTCTAGATGATAACTTTGTAA
CAAAGGCCAATGCCCTAACCTATGACCCCTATGTAAACTACTCCTCCCGCCATACCATACCCCAGCCCTTCTCCTACCAC
TCCCGCTATTTCACCCCCAAACCTGTCCTTGATAGGACAATCGATTACTTCCAACCCAATAACAAAAGAAATCAACTCTG
GCTGAGACTACAAACTACTGGAAATGTAGACCATGTAGGACTCGGCACTGCCTTCGAAAACAGTAAATACGACCAGG
ACTACAATATCCGTATAACCATGTATGTACAATTCAGAGAATTTAATCTTAAAGACCCCCCACTTAACCCTAAG-----

>HQ395044_pcv2d

ATGACGTATCCAAGGAGGCGTTACCGAAGACGAAGACACCGCCCCCGCAGCCATCTTGGCCAGATCCTCCGCCGCCG
CCCCTGGCTCGTCCACCCCCGCCACCGTTACCGCTGGAGAAGGAAAAATGGCATCTTCAACACCCGCCTCTCCCGCACC
ATCGGTTATACTGTCAAAGCTACCACAGTAAGAACGCCCTCCTGGAATGTGGACATGATGAGATTTAATATTAATGAT
TTTCTTCCCCCAGGAGGGGGCTCAAACCCCCTCACTGTGCCCTTTGAATACTACAGAATAAGGAAGGTTAAGGTTGAA
TTCTGGCCCTGCTCCCCAATCACCCAGGGTGACAGGGGAGTGGGCTCCACTGCTGTTATTCTAGATGATAACTTTGTAA
CAAAGGCCAATGCCCTAACCTATGACCCCTATGTAAACTACTCCTCCCGCCATACCATACCCCAGCCCTTCTCCTACCAC
TCCCGCTATTTCACCCCCAAACCTGTCCTTGATAGGACAATCGATTACTTCCAACCCAATAACAAAAGAAATCAACTCTG
GCTGAGACTACAAACTACTGGAAATGTAGACCATGTAGGGCTCGGCACTGCGTTCGAAAACAGTATATACGACCAGG
AATACAATATCCGTATAACCATGTATGTACAATTCAGAGAATTTAATCTTAAAGACCCCCCACTTAACCCTAAG-----

>EF675241_pcv2d

ATGACGTATCCAAGGAGGCGTTACCGAAGGAGAAGACACCGCCCCCGCAGCCATCTTGGCCAGATCCTCCGCCGCCG
CCCCTGGCTCGTCCACCCCCGCCACCGTTACCGCTGGAGAAGGAAAAATGGCATCTTCAACACCCGCCTCTCCCGCACC
ATCGGTTATACTGTCAAAGCTACCACAGTAAGAACGCCCTCCTGGAATGTGGACATGATGAGATTTAATATTAATGAT
TTTCTTCCCCCAGGAGGGGGCTCAAACCCCCTCACTGTGCCCTTTGAATACTACAGAATAAGGAAGGTTAAGGTTGAA
TTCTGGCCCTGCTCCCCAATCACCCAGGGTGACAGGGGAGTGGGCTCCACTGCTGTTATTCTAGATGATAACTTTGTAA
CAAAGGCCAATGCCCTAACCTATGACCCCTATGTAAACTACTCCTCCCGCCATACCATACCCCAGCCCTTCTCCTACCAC
TCCCGCTATTTCACCCCCAAACCTGTCCTTGATAGGACAATCGATTACTTCCAACCCAATAACAAAAGAAATCAACTCTG
GCTGAGACTACAAACTACTGGAAATGTAGACCATGTAGGCCTCGGCACTGCGTTCGAAAACAGTATATACGACCAGG
ACTACAATATCCGTATAACCATGTATGTACAATTCAGAGAATTTAATCTTAAAGACCCCCCACTTAACCCTAAG-----

>AY943819_pcv2d

ATGACGTATCCAAGGAGGCGTTACCGAAGACGAAGACACCGCCCCCGCAGCCATCTTGGCCAGATCCTCCGCCGCCG
CCCCTGGCTCGTCCACCCCCGCCACCGTTACCGCTGGAGAAGGAAAAATGGCATCTTCAACACCCGCCTCTCCCGCACC
ATCGGTTATACTGTCAAAGCTACCACAGTAAGAACGCCCTCCTGGAATGTGGACATGATGAGATTTAATATTAATGAT
TTTCTTCCCCCAGGAGGGGGCTCAAACCCCCTCACTGTGCCCTTTGAATACTACAGAATAAGGAAGGTTAAGGTTGAA
TTCTGGCCCTGCTCCCCAATCACCCAGGGTGACAGGGGAGTGGGCTCCACTGCTGTTATTCTAGATGATAACTTTGTAA
CAAAGGCCAATGCCCTAACCTATGACCCCTATGTAAACTACTCCTCCCGCCATACCATACCCCAGCCCTTCTCCTACCAC
TCCCGCTATTTCACCCCCAAACCTGTCCTTGATAGGACAATCGATTACTTCCAACCCAATAACAAAAGAAATCAACTCTG
GCTGAGACTACAAACTACTGGAAATGTAGACCATGTAGGCCTCGGCACTGCGTTCGAAAACAGTATATACGACCAGG
ACTACAATATCCGTATAACCATGTATGTACAATTCAGAGAATTTAATCTTAAAGACCCCCCACTTAACCCTAAG-----

>AY291317_pcv2d

ATGACGTATCCAAGGAGGCGTTACCGAAGACGAAGACACCGCCCCCGCAGCCATCTTGGCCAGATCCTCCGCCGCCG
CCCCTGGCTCGTCCACCCCCGCCACCGTTACCGCTGGAGAAGGAAAAATGGCATCTTCAACACCCGCCTCTCCCGCACC
ATCGGTTATACTGTCAAAGCTACCACAGTAAGAACGCCCTCCTGGAATGTGGACATGATGAGATTTAATATTAATGAT
TTTCTTCCCCCAGGAGGGGGCTCAAACCCCCTCACTGTGCCCTTTGAATACTACAGAATAAGGAAGGTTAAGGTTGAA
TTCTGGCCCTGCTCCCCAATCACCCAGGGTGACAGGGGAGTGGGCTCCACTGCTGTTATTCTAGATGATAACTTTGTAA
CAAAGGCCAATGCCCTAACCTATGACCCCTATGTAAACTACTCCTCCCGCCATACCATACCCCAGCCCTTCTCCTACCAC
TCCCGCTATTTCACCCCCAAACCTGTCCTTGATAGGACAATCGATTACTTCCAACCCAATAACAAAAGAAATCAACTCTG
GCTGAGACTACAAACTACTGGAAATGTAGACCATGTAGGCCTCGGCACTGCGTTCGAAAACAGTATATACGACCAGG
ACTACAATATCCGTATAACCATGTATGTACAATTCAGAGAATTTAATCTTAAAGACCCCCCACTTAACCCTAAG-----

>EF524539_pcv2d

ATGACGTATCCAAGGAGGCGTTACCGAAGACGAAGACACCGCCCCCGCAGCCATCTTGGCCAGATCCTCCGCCGCCG
CCCCTGGCTCGTCCACCCCCGCCACCGTTACCGCTGGAGAAGGAAAAATGGCATCTTCAACACCCGCCTCTCACGCACC
ATCGGTTATACTGTCAAAGCTACCACAGTAAGAACGCCCTCCTGGAATGTGGACATGATGAGATTTAATATTAATGAT
TTTCTTCCCCCAGGAGGGGGCTCAAACCCCCTCACTGTGCCCTTTGAATACTACAGAATAAGGAAGGTTAAGGTTGAA
TTCTGGCCCTGCTCCCCAATCACCCAGGGTGACAGGGGAGTGGGCTCCACTGCTGTTATTCTAGATGATAACTTTGTAA
CAAAGGCCAATGCCCTAACCTATGACCCCTATGTAAACTACTCCTCCCGCCATACCATACCCCAGCCCTTCTCCTACCAC
TCACGCTATTTCACCCCCAAACCTGTCCTTGATAGGACAATCGATTACTTCCAACCCAATAACAAAAGAAATCAACTCT
GGCTGAGACTACAAACTACTGGAAATGTAGACCATGTAGGCCTCGGCACTGCGTTCGAAAACAGTATATACGACCAG
GACTACAATATCCGTATAACCATGTATGTACAATTCAGAGAATTTAATCTTAAAGACCCCCCACTTAACCCTAAG-----

>AY682994_pcv2d

ATGACGTATCCAAGGAGGCGTTACCGAAGACGAAGACACCGCCCCCGCAGCCATCTTGGCCAGATCCTCCGCCGCCG
CCCCTGGCTCGTCCACCCCCGCCACCGTTACCGCTGGAGAAGGAAAAATGGCATCTTCAACACCCGCCTCTCCCGCACC
ATCGGTTATACTGTCAAAGCTACCACAGTAAGAACGCCCTCCTGGAATGTGGACATGATGAGATTTAATATTAATGAT
TTTCTTCCCCCAGGAGGGGGCTCAAACCCCCTCACTGTGCCCTTTGAATACTACAGAATAAGGAAGGTTAAGGTTGAA
TTCTGGCCCTGCTCCCCAATCACCCAGGGTGACAGGGGAGTGGGCTCCACTGCTGTTATTCTAGATGATAACTTTGTAA
CAAAGGCCAATGCCCTAACCTATGACCCCTATGTAAACTACTCCTCCCGCCATACCATACCCCAGCCCTTCCCCTACCAC
TCCCGCTATTTCACCCCCAAACCTGTCCTTGATAGGACAATCGATTACTTCCAACCCAATAACAAAAGAAATCAACTCTG
GCTGAGACTGCAAACTACTGGAAATGTAGACCATGTAGGCCTCGGCACTGCGTTCGAAAACAGTATATACGACCAGG
ACTACAATATCCGTATAACCATGTATGTACAATTCAGAGAATTTAATCTTAAAGACCCCCCACTTAACCCTAAG-----

>AY682996_pcv2d

ATGACGTATCCAAGGAGGCGTTACCGAAGGCGAAGACACCGCCCCCGCAGCCATCTTGGCCAGATCCTCCGCCGCCG
CCCCTGGCTCGTCCACCCCCGCCACCGTTACCGCTGGAGAAGGAAAAATGGCATCTTCAACACCCGCCTCTCCCGCACC
ATCGGTTATACTGTCGAAGCTACCACAGTAAGAACGCCCTCCTGGAATGTGGACATGATGAGATTTAATATTAATGAT
TTTCTTCCCCCAGGAGGGGGCTCAAACCCCCTCACTGTGCCCTTTGAATACTACAGAATAAGGAAGGTTAAGGTTGAA
TTCTGGCCCTGCTCCCCAATCACCCAGGGTGACAGGGGAGTGGGCTCCACTGCTGTTATTCTAGATGATAACTTTGTAA
CAAAGGCCAATGCCCTAACCTATGACCCCTATGTAAACTACTCCTCCCGCCATACCATACCCCAGCCCTTCCCCTACCAC
TCCCGCTATTTCACCCCCAAACCTGTCCTTGATAGGACAATCGATTACTTCCAACCCAATAACAAAAGAAATCAACTCTG
GCTGAGACTGCAAACTACTGGAAATGTAGACCATGTAGGCCTCGGCACTGCGTTCGAAAACAGTATATACGACCAGG
ACTACAATATCCGTATAACCATGTATGTACAATTCAGAGAATTTAATCTTAAAGACCCCCCACTTAACCCTAAG-----

>AY682991_pcv2d

ATGACGTATCCAAGGAGGCGTTACCGAAGACGAAGACACCGCCCCCGCAGCCATCTTGGCCAGATCCTCCGCCGCCG
CCCCTGGCTCGTCCACCCCCGCCACCGTTACCGCTGGAGAAGGAAAAATGGCATCTTCAACACCCGCCTCTCCCGCACC
ATCGGTTATACTGTCAAAGCTACCACAGTAAGAACGCCCTCCTGGAATGTGGACATGATGAGATTTAATATTAATGAT
TTTCTTCCCCCAGGAGGGGGCTCAAACCCCCTCACTGTGCCCTTTGAATACTACAGAATAAGGAAGGTTAAGGTTGAA
TTCTGGCCCTGCTCCCCAATCACCCAGGGTGACAGGGGAGTGGGCTCCACTGCTGTTATTCTAGATGATAACTTTGTAA
CAAAGGCCAATGCCCTAACCTATGACCCCTATGTAAACTACTCCTCCCGCCATACCATACCCCAGCCCTTCTCCTACCAC
TCCCGCTATTTCACCCCCAAACCTGTCCTTGATAGGACAATCGATTACTTCCAACCCAATAACAAAAGAAATCAACTCTG
GCTGAGACTGCAAACTACTGGAAATGTAGACCATGTAGGCCTCGGCACTGCGTTCGAAAACAGTATATACGACCAGG
ACTACAATATCCGTATAACCATGTATGTACAATTCAGAGAATTTAATCTTAAAGACCCCCCACTTAACCCTAAG-----

>AB462384_pcv2d

ATGACGTATCCAAGGAGGCGTTACCGAAGACGAAGGCACCGCCCCCGCAGCCATCTTGGCCAGATCCTCCGCCGCCG
CCCCTGGCTCGTCCACCCCCGCCACCGTTACCGCTGGAGAAGGAAAAATGGCATCTTCAACACCCGCCTCTCCCGCACC
ATCGGTTATACTGTCAAAGCTACCACAGTAAGAACACCCTCCTGGAATGTGGACATGATGAGATTTAATATTAATGATT
TTCTTCCCCCAGGAGGGGGCTCAAACCCCCTCACTGTGCCCTTTGAATACTACAGAATAAGGAAGGTTAAGGTTGAAT
TCTGGCCCTGCTCCCCAATCACCCAGGGTGACAGGGGAGTGGGCTCCACTGCTGTTATTCTAGATGATAACTTTGTAA
CAAAGGCCAATGCCCTAACCTATGACCCCTATGTAAACTACTCCTCCCGCCATACCATACCCCAGCCCTTCTCCTACCAC
TCCCGCTATTTCACCCCCAAACCTGTCCTTGATAGGACAATCGATTACTTCCAACCCAATAACAAAAGAAATCAACTCTG
GCTGAGACTACAAACTACTGGAAATGTAGACCATGTAGGCCTCGGCACTGCGTTCGAAAACAGTATATACGACCAGG
ACTACAATATCCGTATAACCATGTATGTACAATTCAGAGAATTTAATCTTAAAGACCCCCCACTTAACCCTAAG-----

>FJ644560_pcv2d

ATGACGTATCCAAGGAGGCGTTACCGAAGACGAAGACACCGCCCCCGCAGCCATCTTGGCCAGATCCTCCGCCGCCG
CCCCTGGCCCGTCCACCCCCGCCACCGTTACCGCTGGAGAAGGAAAAATGGCATCTTCAACACCCGCCTCTCCCGCAC
CATCGGTTATACTGTCAAAGCTACCACAGTAAGAACGCCCTCCTGGAATGTGGACATGATGAGATTTAATATTGATGA
TTTTCTTCCCCCAGGAGGGGGCTCAAACCCCCTCACTGTGCCCTTTGAATACTACAGAATAAGGAAGGTTAAGGTTGA
ATTCTGGCCCTGCTCCCCAATCACCCAGGGTGACAGGGGAGTGGGCTCCACTGCTGTTATTCTAGATGATAACTTTGTA
ACAAAGGCCAATGCCCTAACCTATGACCCCTATGTAAACTACTCCTCCCGCCATACCATACCCCAGCCCTTCTCCTACCA
CTCCCGCTATTTCACCCCCAAACCTGTCCTTGATAGGACAATCGATTACTTCCAACCCAATAACAAAAGAAATCAACTCT
GGCTGAGACTACAAACTACTGGAAATGTAGACCATGTAGGCCTCGGCACTGCGTTCGAAAACAGTATATACGACCAG
GACTACAATATCCGTATAACCATGTATGTACAATTCAGAGAATTTAATCTTAAAGACCCCCCACTTAACCCTAAG-----

>FJ667588_pcv2d

ATGACGTATCCAAGGAGGCGTTACCGAAGACGAAGACACCGCCCCCGCAGCCATCTTGGCCAGATCCTCCGCCGCCG
CCCCTGGCTCGTCCACCCCCGCCACCGTTACCGCTGGAGAAGGAAAAATGGCATCTTCAACACCCGCCTCTCCCGCACC
ATCGGTTATACTGTAAAAGCTACCACAGTAAGAACGCCCTCCTGGAATGTGGACATGATGAGATTTAATATTAATGAT
TTTCTTCCCCCAGGAGGGGGCTCAAACCCCCTCACTGTCCCCTTTGAATACTACAGAATAAGGAAGGTTAAGGTTGAA
TTCTGGCCCTGCTCCCCAATCACCCAGGGTGACAGGGGAGTGGGCTCCACTGCTGTTATTCTAGATGATAACTTTGTAA
CAAAGGCCAATGCCCTAACCTATGACCCCTATGTAAACTACTCCTCCCGCCATACCATACCCCAGCCCTTCTCCTACCAC
TCCCGCTATTTCACCCCCAAACCTGTCCTTGATAGGACAATCGATTACTTCCAACCCAATAACAAAAGAAATCAACTCTG
GCTGAGACTACAAACTACTGGAAATGTAGACCATGTAGGCCTCGGCACTGCGTTCGAAAACAGTATATACGACCAGG
ACTACAATATCCGTATAACCATGTATGTACAATTCAGAGAATTTAATCTTAAAGACCCCCCACTTAAACCTAAG-----

>FJ594471_pcv2d

ATGACGTATCCAAGGAGGCGTTACCGAAGACGAAGACACCGCCCCCGCAGCCATCTTGGCCAGATCCTCCGCCGCCG
CCCCTGGCTCGTCCACCCCCGCCACCGTTACCGCTGGAGAAGGAAAAATGGCATCTTCAACACCCGCCTCTCCCGCACC
ATCGGTTATACTGTAAAAGCTACCACAGTAAGAACGCCCTCCTGGAATGTGGACATGATGAGATTTAATATTAATGAT
TTTCTTCCCCCAGGAGGGGGCTCAAACCCCCTCACTGTCCCCTTTGAATACTACAGAATAAGGAAGGTTAAGGTTGAA
TTCTGGCCCTGCTCCCCAATCACCCAGGGTGACAGGGGAGTGGGCTCCACTGCTGTTATTCTAGATGATAACTTTGTAA
CAAAGGCCAATGCCCTAACCTATGACCCCTATGTAAACTACTCCTCCCGCCATACCATACCCCAGCCCTTCTCCTACCAC
TCCCGCTATTTCACCCCCAAACCTGTCCTTGATAGGACAATCGATTACTTCCAACCCAATAACAAAAGAAATCAACTCTG
GCTGAGACTACAAACTACTGGAAATGTAGACCATGTAGGCCTCGGCACTGCGTTCGAAAACAGTATATACGACCAGG
ACTACAATATCCGTATAACCATGTATGTACAATTCAGAGAATTTAATCTTAAAGACCCCCCACTTAAACCTAAG-----

>FJ667582_pcv2d

ATGACGTATCCAAGGAGGCGTTACCGAAGACGAAGACACCGCCCCCGCAGCCATCTTGGCCAGATCCTCCGCCGCCG
CCCCTGGCTCGTCCACCCCCGCCACCGTTACCGCTGGAGAAGGAAAAATGGCATCTTCAACACCCGCCTCTCCCGCACC
ATCGGTTATACTGTAAAAGCTACCACAGTAAGAACGTCCTCCTGGAATGTGGACATGATGAGATTTAATATTAATGAT
TTTCTTCCCCCAGGAGGGGGCTCAAACCCCCTCACTGTCCCCTTTGAATACTACAGAATAAGGAAGGTTAAGGTTGAA
TTCTGGCCCTGCTCCCCAATCACCCAGGGTGACAGGGGAGTGGGCTCCACTGCTGTTATTCTAGATGATAACTTTGTAA
CAAAGGCCAATGCCCTAACCTATGACCCCTATGTAAACTACTCCTCCCGCCATACCATACCCCAGCCCTTCTCCTACCAC
TCCCGCTATTTCACCCCCAAACCTGTCCTTGATAGGACAATCGATTACTTCCAACCCAATAACAAAAGAAATCAACTCTG
GCTGAGACTACAAACTACTGGAAATGTAGACCATGTAGGCCTCGGCACTGCGTTCGAAAACAGTATATACGACCAGG
ACTACAATATCCGTATAACCATGTATGTACAATTCAGAGAATTTAATCTTAAAGACCCCCCACTTAAACCTAAG-----

>FJ667586_pcv2d

ATGACGTATCCAAGGAGGCGTTACCGAAGACGAAGACACCGCCCCCGCAGCCATCTTGGCCAGATCCTCCGCCGCCG
CCCCTGGCTCGTCCACCCCCGCCACCGTTACCGCTGGAGAAGGAAAAATGGCATCTTCAACACCCGCCTCTCCCGCACC
ATCGGTTATACTGTAAAAGCTACCACAGTAAGAACGCCCTCCTGGAATGTGGACATGATGGGATTTAATATTAATGAT
TTTCTTCCCCCAGGAGGGGGCTCAAACCCCCTCACTGTCCCCTTTGAATACTACAGAATAAGGAAGGTTAAGGTTGAA
TTCTGGCCCTGCTCCCCAATCACCCAGGGTGACAGGGGAGTGGGCTCCACTGCTGTTATTCTAGATGATAACTTTGTAA
CAAAGGCCAATGCCCTAACCTATGACCCCTATGTAAACTACTCCTCCCGCCATACCATACCCCAGCCCTTCTCCTACCAC
TCCCGCTATTTCACCCCCAAACCTGTCCTTGATAGGACAATCGATTACTTCCAACCCAATAACAAAAGAAATCAACTCTG
GCTGAGACTACAAACTACTGGAAATGTAGACCATGTAGGCCTCGGCACTGCGTTCGAAAACAGTATATACGACCAGG
ACTACAATATCCGTATAACCATGTATGTACAATTCAGAGAATTTAATCTTAAAGACCCCCCACTTAAACCTAAG-----

>FJ667585_pcv2d

ATGACGCATCCAAGGAGGCGTTACCGAAGACGAAGACACCGCCCCCGCAGCCATCTTGGCCAGATCCTCCGCCGCCG
CCCCTGGCTCGTCCACCCCCGCCACCGTTACCGCTGGAGAAGGAAAAATGGCATCTTCAACACCCGCCTCTCCCGCACC
ATCGGTTATACTGTAAAAGCTACCACAGTAAGAACGCCCTCCTGGAATGTGGACATGATGAGATTTAATATTAATGAT
TTTCTTCCCCCAGGAGGGGGCTCAAACCCCCTCACTGTCCCCTTTGAATACTACAGAATAAGGAAGGTTAAGGTTGAA
TTCTGGCCCTGCTCCCCAATCACCCAGGGTGACAGGGGAGTGGGCTCCACTGCTGTTATTCTAGATGATAACTTTGTAA
CAAAGGCCAATGCCCTAACCTATGACCCCTATGTAAACTACTCCTCCCGCCATACCATACCCCAGCCCTTCTCCTACCAC
TCCCGCTATTTCACCCCCAAACCTGTCCTTGATAGGACAATCGATTACTTCCAACCCAATAACAAAAGAAATCAACTCTG
GCTGAGACTACAAACTACTGGAAATGTAGACCATGTAGGCCTCGGCACTGCGTTCGGAAACAGTATATACGACCAGG
ACTACAATATCCGTATAACCATGTATGTACAATTCAGAGAATTTAATCTTAAAGACCCCCCACTTAAACCTAAG-----

>FJ667590_pcv2d

ATGACGTATCCAAGGAGGCGTTACCGAAGACGAAGACACCGCCCCCGCAGCCATCTTGGCCAGATCCTCCGCCGCCG
CCCCTGGCTCGTCCACCCCCGCCACCGTTACCGCTGGAGAAGGAAAAATGGCATCTTCAACACCCGCCTCTCCCGCACC
ATCGGTTATACTGTAAAAGCTACCACAGTAAGAACGCCCTCCTGGAATGTGGACATGATGAGATTTAATATTAATGAT
TTTCTTCCCCCAGGAGGGGGCTCAAACCCCCTCACTGTCCCCTTTGAATACTACAGAATAAGGAAGGTTAAGGTTGGA
TTCTGGCCCTGCTCCCCAATCACCCAGGGTGACAGGGGGGTGGGCTCCACTGCTGTTATTCTAGATGATAACTTTGTA
ACAAAGGCCAATGCCCTAACCTATGACCCCTATGTAAACTACTCCTCCCGCCATACCATACCCCAGCCCTTCTCCTACCA
CTCCCGCTATTTCACCCCCAAACCTGTCCTTGATAGGACAATCGATTACTTCCAACCCAATAACAAAAGAAATCAACTCT
GGCTGAGACTACAAACTACTGGAAATGTAGACCATGTAGGCCTCGGCACTGCGTTCGAAAACAGTATATACGACCAG
GACTACAATATCCGTATAACCATGTATGTACAATTCAGAGAATTTAATCTTAAAGACCCCCCACTTAAACCTAAG-----

>FJ667583_pcv2d

ATGACGTATCCAAGGAGGCGTTACCGAAGACGAAGACACCGCCCCCGCAGCCATCTTGGCCAGATCCTCCGTCGCCG
CCCCTGGCACGTCCACCCCCGCCACCGTTACCGCTGGAGAAGGAAAAATGGCATCTTCAACACCCGCCTCTCCCGCAC
CATCGGTTATACTGTAAAAGCTACCACAGTAAGAACGCCCTCCTGGAATGTGGACATGATGAGATTTAATATTAATGA
TTTTCTTCCCCCAGGAGGGGGCTCAAACCCCCTCACTGTCCCCTTTGAATACTACAGAATAAGGAAGGTTAAGGTTGA
ATTCTGGCCCTGCTCCCCAATCACCCAGGGTGACAGGGGAGTGGGCTCCACTGCTGTTATTCTAGATGATAACTTTGTA
ACAAAGGCCAATGCCCTAACCTATGACCCCTATGTAAACTACTCCTCCCGCCATACCATACCCCAGCCCTTCTCCTACCA
CTCCCGCTATTTCACCCCCAAACCTGTCCTTGATAGGACAATCGATTACTTCCAACCCAATAACAAAAGAAATCAACTCT
GGCTGAGACTACAAACTACTGGAAATGTAGACCATGTAGGCCTCGGCACTGCGTTCGAAAACAGTACATACGACCAG
GACTACAATATCCGTATAACCATGTATGTACAATTCAGAGAATTTAATCTTAAAGACCCCCCACTTAAACCTAAG-----

>EU148505_pcv2c

ATGACGTATCCAAGGAGGCGTTACCGGAGAAGAAGACACCGCCCCCGCAGCCATCTTGGCCATATCCTCCGCCGCCG
CCCCTGGCTCGTCCACCCCCGCCACCGCTACCGTTGGAGAAGGAAAAATGGAATCTTCAATGCCCGCCTCTCCCGCTCC
TTTGTTTATACCGTTAATGCCTCACAGGTCTCACCACCCTCTTGGGCGGTGGACATGATGAGATTTAATATTAATCAATT
TCTTCCCCCAGGAGGGGGCTCAAACCCCCTCACTGTGCCCTTTGAATACTACAGAATAAGAAAGGTTAAAGTGGAATT
CTTTGCCAGATCCCCCATCACCCAAGGTGACAGGGGAGTGGGCTCCACTGCTGTAATTCTAGATGATAACTTTGTAAC
AAAGGCCACAGCCCTAACCTATGACCCCTATGTAAACTACTCCTCCCGCCATACCATAACCCAGCCCTTCTCCTACCACT
CCCGCTACTTTACCCCCAAACCCGTCCTTGATTCCACTATTGATTACTTCCAACCAAATAACAAAAGAAATCAGCTGTGG
ATGAGACTACAAACTACTGGAAATGTAGACCATGTAGGCCTCGGACACGCCTTTCAAAACAGTACAAATGCCCAGGCC
TACAATGTCCGTGTAACCATGTATGTACAATTCAGAGAATTTAATCTTAAAGACCCCCCACTTAACCCTAAG-----

>EU148503_pcv2c

ATGACGTATCCAAGGAGGCGTTACCGGAGAAGAAGACACCGCCCCCGCAGCCATCTTGGCCATATCCTCCGCCGCCG

CCCCTGGCTCGTCCACCCCCGCCACCGCTACCGTTGGAGAAGGAAAAATGGAATCTTCAATGCCCGCCTCTCCCGCTCC

TTTGTTTATACCGTTAATGCCTCACAGGTCTCACCACCCTCTTGGGCGGTGGACATGATGAGATTTAATATTAACCAATT

TCTTCCCCCAGGAGGGGGCTCAAACCCCCTCACTGTGCCCTTTGAATACTACAGAATAAGAAAGGTTAAAGTGGAATT

CTTTGCAAGATCCCCCATCACCCAAGGTGACAGGGGAGTGGGCTCCACTGCTGTTATTCTAAATGATAACTTTGTAACA

AAGGCCACAGCCCTAACCTATGACCCCTATGTAAACTACTCCTCCCGCCATACCATAACCCAACCCTTCTCCTACCACTC

CCGCTACTTTACCCCCAAACCTGTCCTTGATTCCACTATTGATTACTTCCAACCAAATAACAAAAGAAATCAGCTGTGGA

TGAGACTACAAACTACTGGAAATGTAGACCATGTAGGCCTCGGACACGCCTTTCAAAACAGTACAAATGCCCAGGCCT

ACAATGTCCGTGTAACCATGTATGTACAATTCAGAGAATTTAATCTTAAAGACCCCCCACTTAACCCTAAG-----

>EU148504_pcv2c

ATGACGTATCCAAGGAGGCGTTACCGGAGAAGAAGACACCGCCCCCGCAGCCATCTTGGCCATATCCTCCGCCGCCG

CCCCTGGCTCGTCCACCCCCGCCACCGCTACCGTTGGAGAAGGAAAAATGGAATCTTCAATGCCCGCCTCTCCCGCTCC

TTTGTTTATACCGTTAATGCCTCACAGGTCTCACCACCCTCTTGGGCGGTGGACATGATGAGATTTAATATTAACCAATT

TCTTCCCCCAGGAGGGGGCTCAAACCCCCTCACTGTGCCCTTTGAATACTACAGAATAAGAAAGGTTAAAGTGGAATT

CTTTGCCAGATCCCCCATCACCCAAGGTGACAGGGGAGTGGGCTCCACTGCTGTTATTCTAGATGATAACTTTGTAACC

AAGGCCACAGCCCTTACCTATGACCCCTATGTAAACTACTCCTCCCGCCATACCATAACCCAACCCTTCTCCTACCACTC

CCGCTACTTTACCCCCAAACCTGTCCTTGATTCCACTATTGATTACTTCCAACCAAATAACAAAAGAAATCAGCTGTGGA

TGAGACTACAAACTACTGGAAATGTAGACCATGTAGGCCTCGGACACGCCTTTCAAAACAGTACAAATGCCCAGGCCT

ACAATGTCCGTGTAACCATGTATGTACAATTCAGAGAATTTAATCTTAAAGACCCCCCACTTAACCCTAAG-----
